# Supplementary material for: The IRE1α/XBP1 signaling axis drives myoblast fusion in adult skeletal muscle
Source: EMBO Rep. 2024 Jul 9;25(8):3627–50. doi: 10.1038/s44319-024-00197-4 (PMC11316051; doi:10.1038/s44319-024-00197-4)
Supplement: Supplementary file 1 — Table EV1 [file 44319_2024_197_MOESM1_ESM.docx]

**Table EV1.** List of primers used for PCR/qRT-PCR analysis.

| **Gene Name** | **Forward primer (5'-3')** | **Reverse primer (5'-3')** |
| --- | --- | --- |
| M-cadherin | TGGGCAGTCCCTGAGCCCAAA | TCCAGCGTGGCATTGAGGTACA |
| N-cadherin | CAGCAGATTTCAAGGTGGACGA | TCCTGGGTTTCTTTGTCTTGGG |
| BID-Integrin | CATCCCAATTGTAGCAGGCG | GAGACCAGCTTTACGTCCATAG |
| Caveolin-3 | GACCCCAAGAACATCAATGAGGAC | AGAAGGAGATACAGGCGAACAGGA |
| Myoferlin | CTACCAGAATGAGAATCGCTACCC | TACTCCCAGCCTTTCTCATCCA |
| ADAM12 | GGGCAAGAAGGCATAAGAGAGAGA | TGGTGAATGGGTCCTGGCTTAT |
| Myomaker | TATACTCCGGTCCCATAGGC | ATGCTCTTGTCGGGGTACAG |
| Myomerger-L | ACCAGCTTTCATGCCAGAAG | ATGTCTTGGGAGCTCAGTCG |
| Myomerger-S | CAGGAGGGCAAGAAGTTCAG | ATGTCTTGGGAGCTCAGTCG |
| IL-4 | GGATGTGCCAAACGTCCTC | GAGTTCTTCTTCAAGCATGGAG |
| IL-6 | CCTTCTTGGGACTGATGCTGG | GCCTCCGACTTGTGAAGTGGT |
| Nephronectin | CCAGAACAACTCCACTACCACCAA | CTGGGTCGTCCTTTACTTCCTCAT |
| Wnt3 | TGGGCCTGTCTTGGACAAA | GCGATGGCATGCACGAA |
| Wnt4 | CTGGAGAAGTGTGGCTGTGA | GGACGTCCACAAAGGACTGT |
| Wnt5a | GGCATCAAGGAATGCCAGTA | GTACGTGAAGGCCGTCTCTC |
| Wnt11 | GTAGGGCCTTCGCTGACAT | CGATGGTGTGACTGATGGTG |
| Fzd2 | CATCTCCATCCCGCTGTGCA | AGCACAGGAAGAAGCGCAGCTC |
| Fzd4 | GGCTACAACGTGACCAAGATGCC | GCACATTGGCACATAAACCGAAC |
| Fzd6 | GCGGCGTTTGCTTCGTT | CACAGAGGCAGAAGGACGAAGT |
| Axin-2 | TTTGGCACAGCTAGAGGAAG | TGGCTCTTTGTGATCTTCTGG |
| β-actin | CAGGCATTGCTGACAGGATG | TGCTGATCCACATCTGCTGG |
| Binding site 1 | GGTGACAGTCACGAAAGACC | GCCAGGTCCCTTTCCAAAT |
| Binding site 2 | CAGTTAAGGTCAAGCCTGGT | CACTCACCTTGTTCCTCCTC |
| Hspa5 | TGGTGGCATGGACCAATCAG | CGCCGACTCGCCTTATATAC |
| Dnajb9 | GAGCCGACCTACACGAAAC | AGGACCAAACGGCAACAA |
| Mymk promoter cloning | GGTACCGGTACCTGTCTCTTAT  GACAAATGTCCTCT | GATATCCTCGAGACTGGTTTCTT  TCCCCCAT |
