# Supplementary material for: The IRE1α/XBP1 signaling axis drives myoblast fusion in adult skeletal muscle
Source: EMBO Rep. 2024 Jul 9;25(8):3627–50. doi: 10.1038/s44319-024-00197-4 (PMC11316051; doi:10.1038/s44319-024-00197-4)
Supplement: Supplementary file 2 — Table EV2 [file 44319_2024_197_MOESM2_ESM.docx]

**Table EV2.** Mean TPM values for expression of various genes in control cultures.

| **Gene** | **Mean TPM values** | **Gene** | **Mean TPM values** |
| --- | --- | --- | --- |
| Hspa5 | 929.948 | Uba6 | 11.305 |
| Sdf2l1 | 105.930 | Pcm1 | 38.873 |
| Selenos | 150.429 | Akap9 | 18.635 |
| H13 | 46.874 | Golgb1 | 29.634 |
| Tmem129 | 41.276 | Atm | 22.331 |
| Derl1 | 144.536 | Fam168b | 58.945 |
| Man1b1 | 29.816 | Cul4b | 42.882 |
| Ccdc47 | 72.318 | Cul9 | 2.797 |
| Pomt2 | 7.664 | Ikzf1 | 0.045 |
| Rnf185 | 45.056 | Psme4 | 98.860 |
| Derl2 | 15.596 | Cenpf | 2.526 |
| Wfs1 | 137.619 | Atr | 2.190 |
| Dnajb9 | 64.526 | Xrn1 | 4.485 |
| Usp14 | 52.249 | Rictor | 4.565 |
| Uggt1 | 26.879 | Kmt2c | 4.851 |
| Syvn1 | 36.732 | Ubr2 | 26.618 |
| Sec13 | 108.220 | Birc6 | 15.102 |
| Ecpas | 43.393 | Huwe1 | 34.763 |
| Ube4a | 25.775 | Vps13c | 5.553 |
| Sel1l | 49.417 | Vps13d | 9.412 |
| Edem3 | 91.547 | Mtor | 28.880 |
| Hspa13 | 19.262 | Dicer1 | 13.641 |
| Ube2j1 | 76.995 | Ube4b | 65.523 |
| Dnajc10 | 42.990 | Notch1 | 17.609 |
| Nploc4 | 93.187 | Irf4 | 0.011 |
| Tmem182 | 190.196 | Mstn | 33.855 |
| Cxcl10 | 69.998 |  |  |
| Cflar | 6.785 |  |  |
| Cdon | 103.104 |  |  |
| Neo1 | 24.070 |  |  |
| Mymk | 2789.945 |  |  |
| Mapk14 | 53.559 |  |  |
| Ehd2 | 41.089 |  |  |
| Mymx | 594.790 |  |  |
