# Supplementary figures and images for: The IRE1α/XBP1 signaling axis drives myoblast fusion in adult skeletal muscle (part 1 of 4)
Source: EMBO Rep. 2024 Jul 9;25(8):3627–50. doi: 10.1038/s44319-024-00197-4 (PMC11316051; doi:10.1038/s44319-024-00197-4)

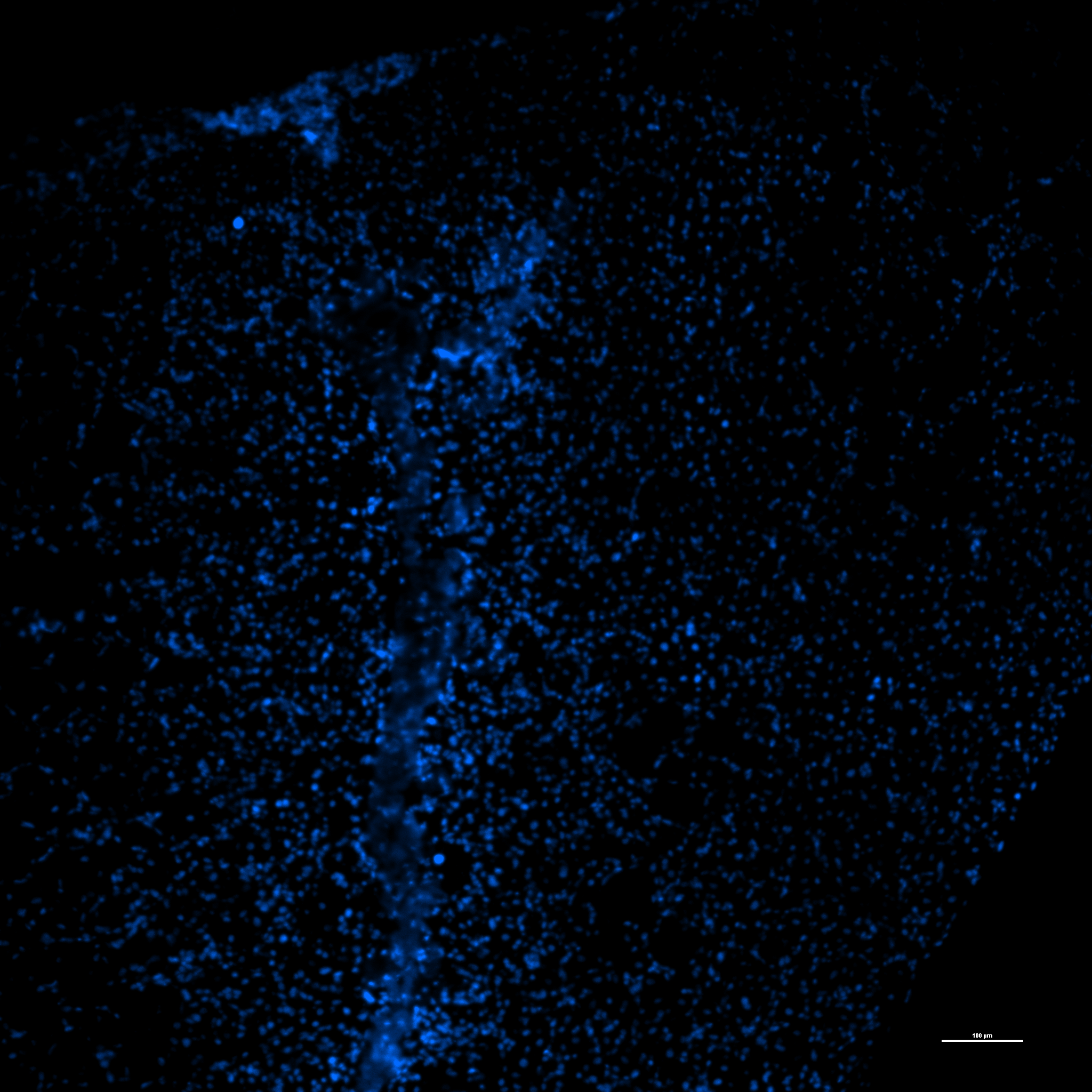

Supplement: Supplementary file 4 — Source data Fig. 1 [file 44319_2024_197_MOESM4_ESM.zip › Figure 1/1A/Injured_DAPI.tif]

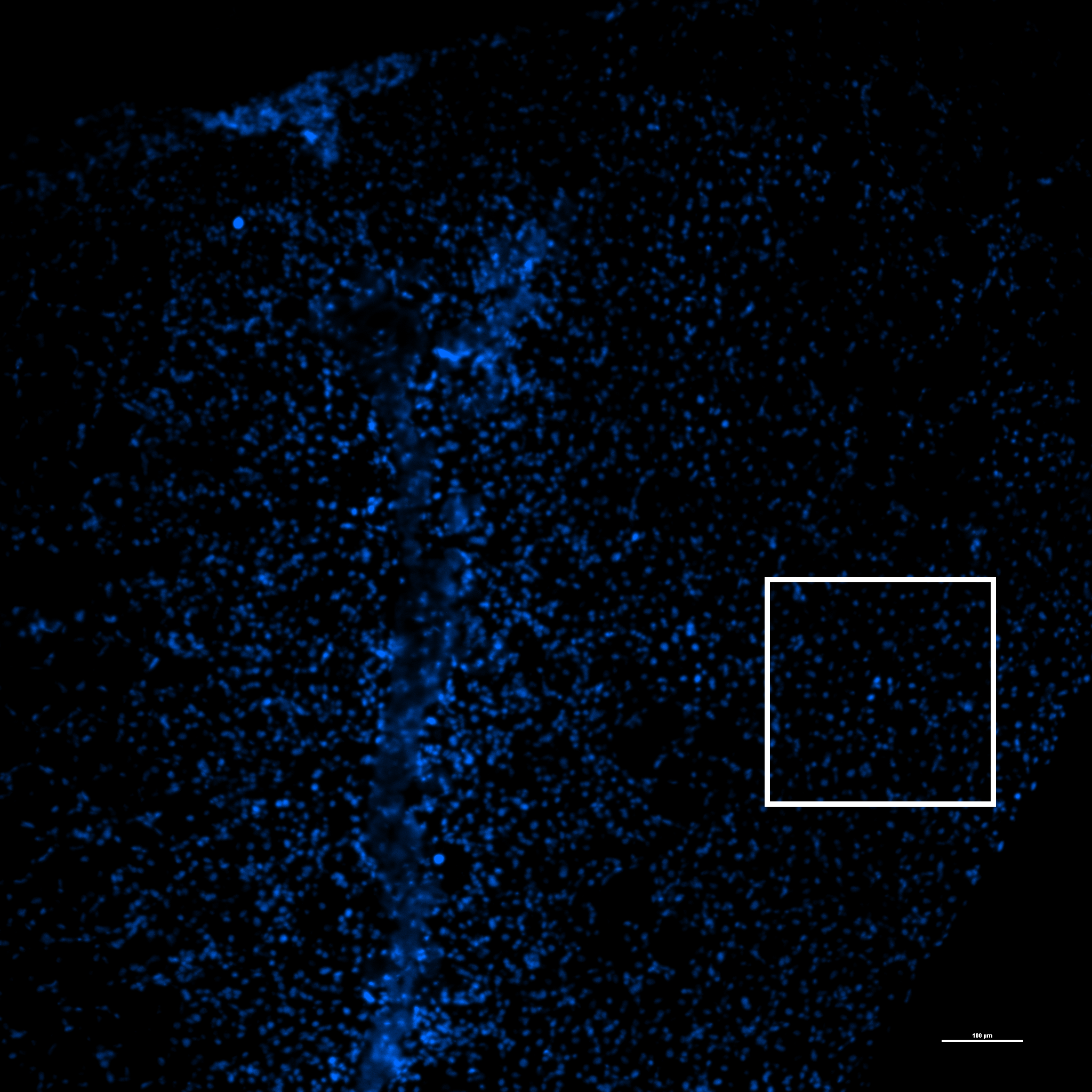

Supplement: Supplementary file 4 — Source data Fig. 1 [file 44319_2024_197_MOESM4_ESM.zip › Figure 1/1A/Injured_DAPI_with_box.tif]

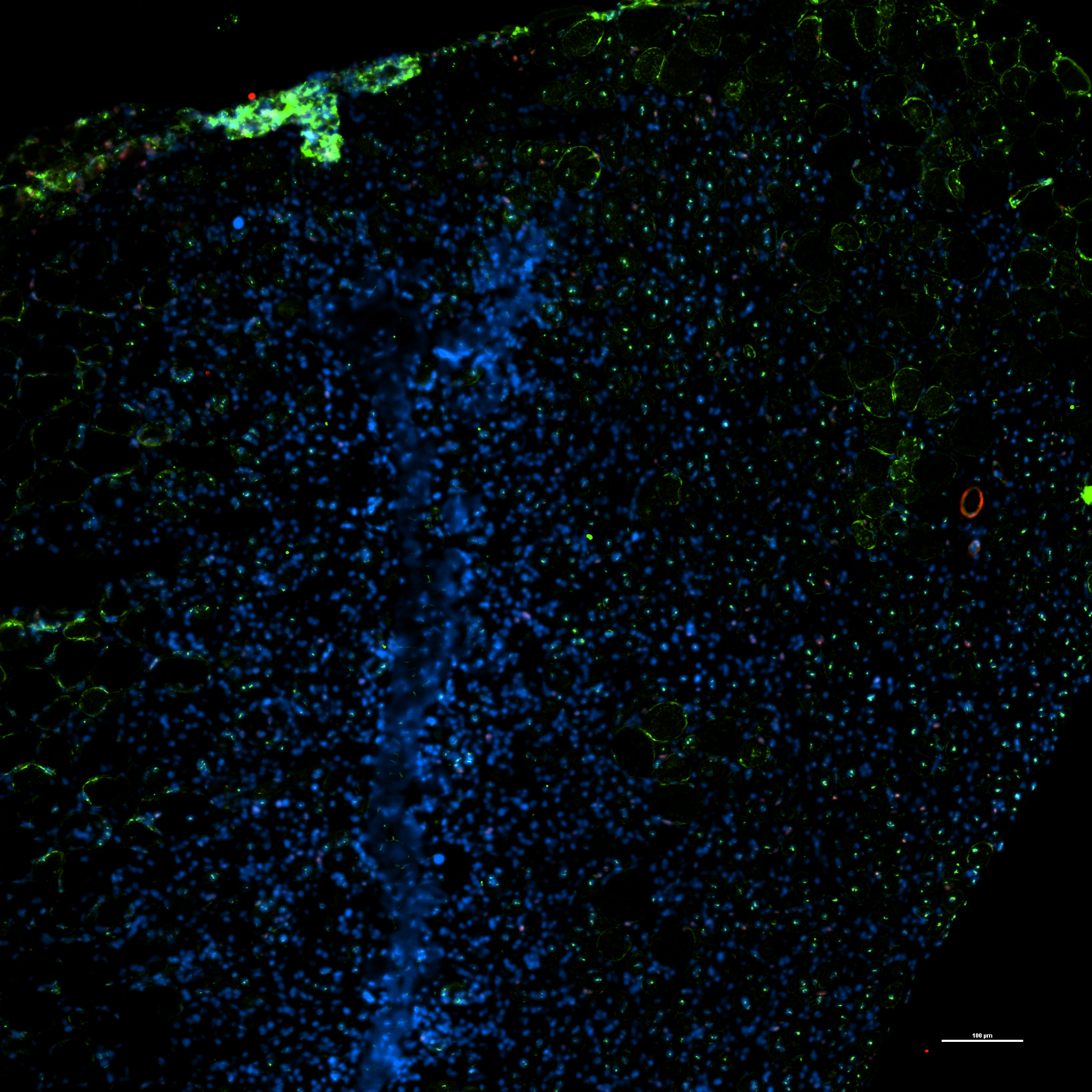

Supplement: Supplementary file 4 — Source data Fig. 1 [file 44319_2024_197_MOESM4_ESM.zip › Figure 1/1A/Injured_Merged.tif]

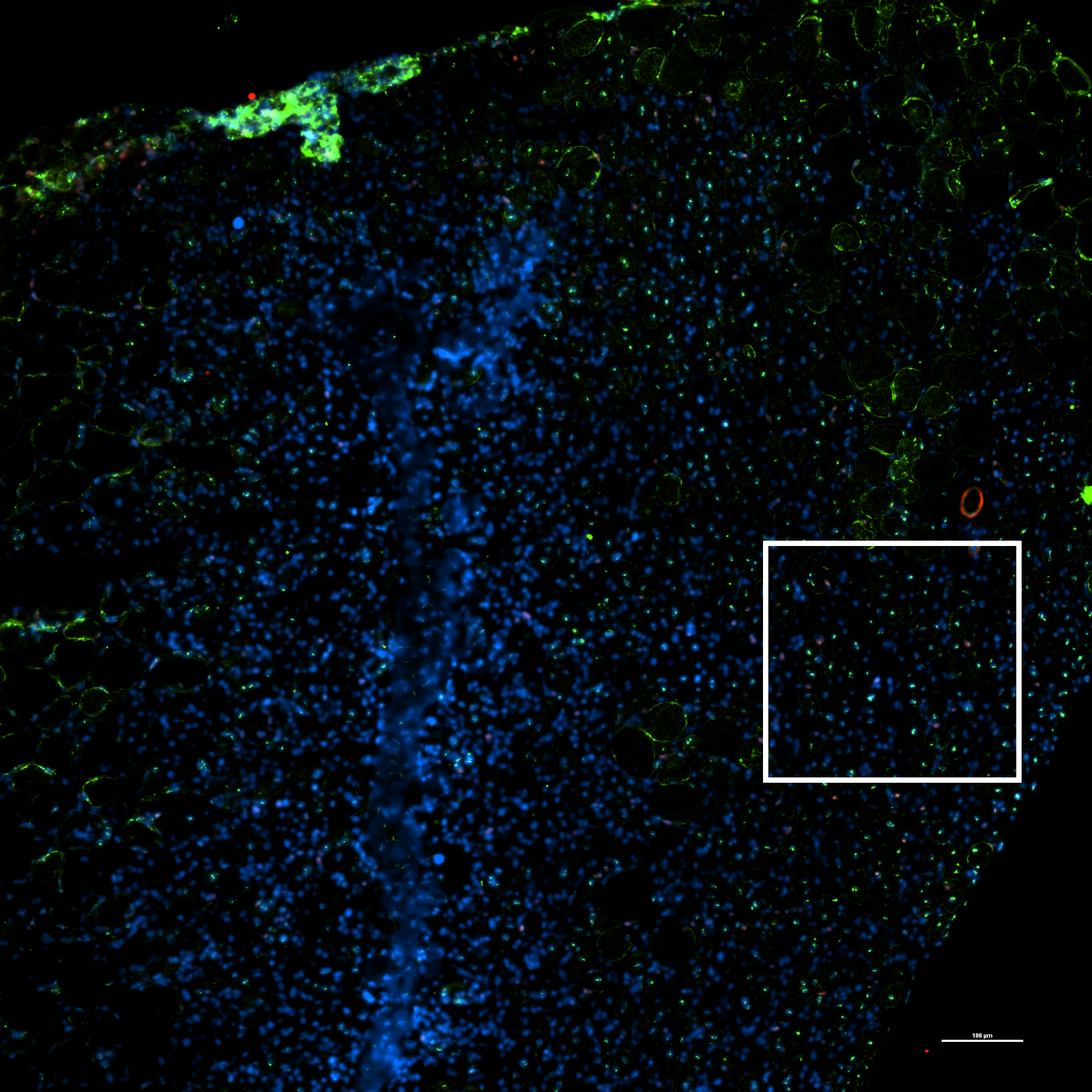

Supplement: Supplementary file 4 — Source data Fig. 1 [file 44319_2024_197_MOESM4_ESM.zip › Figure 1/1A/Injured_Merged_with_box.tif]

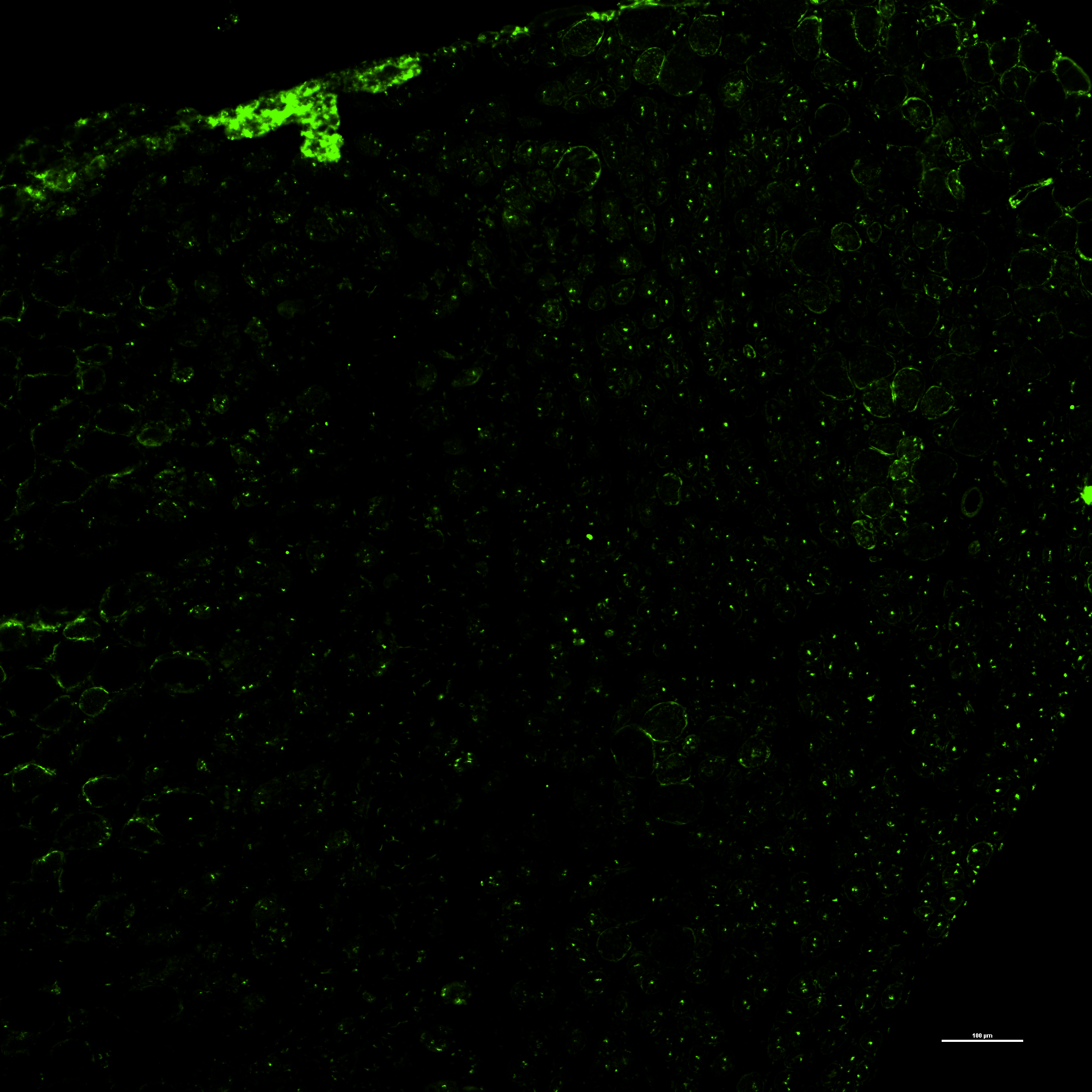

Supplement: Supplementary file 4 — Source data Fig. 1 [file 44319_2024_197_MOESM4_ESM.zip › Figure 1/1A/Injured_p-IRE1a.tif]

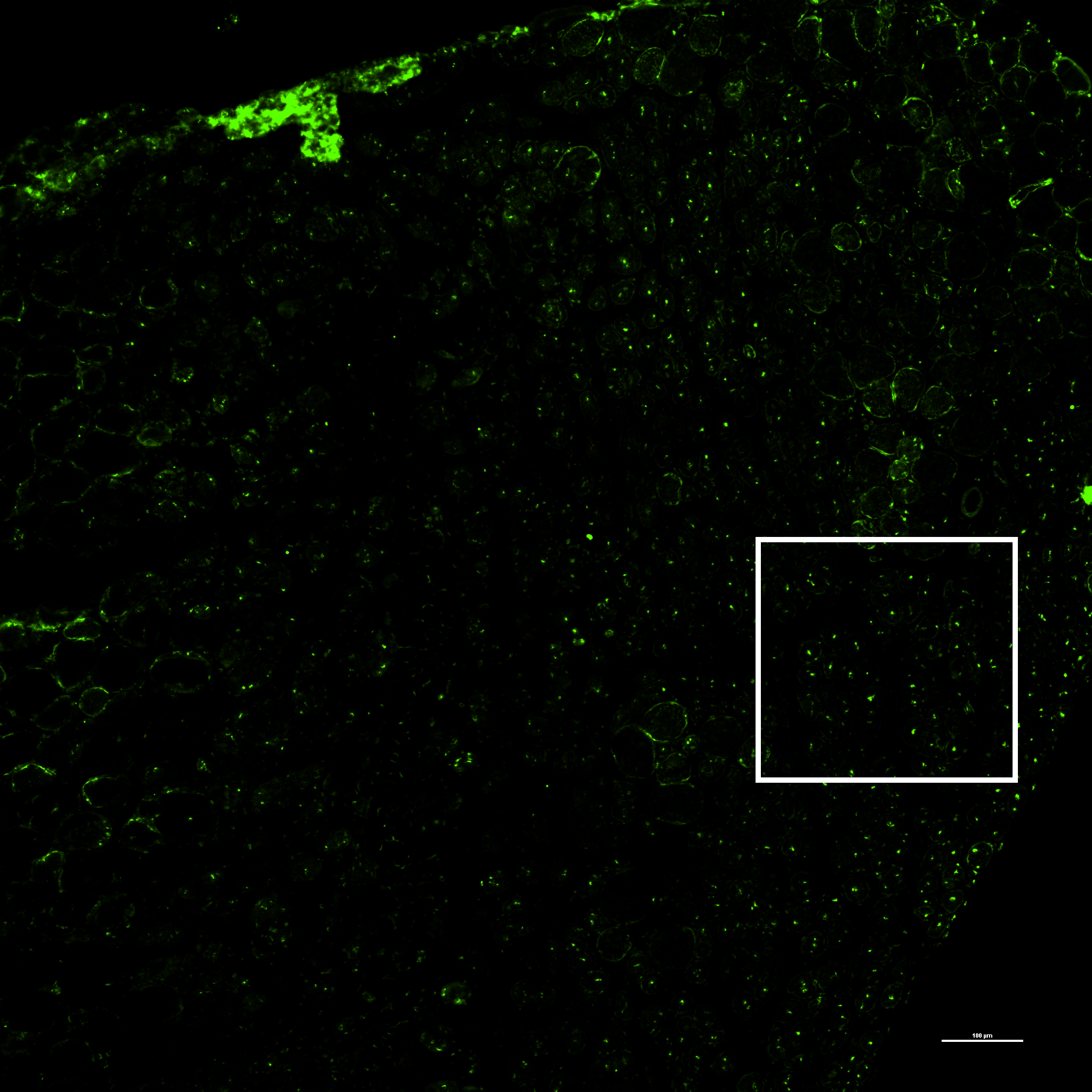

Supplement: Supplementary file 4 — Source data Fig. 1 [file 44319_2024_197_MOESM4_ESM.zip › Figure 1/1A/Injured_p-IRE1a_with_box.tif]

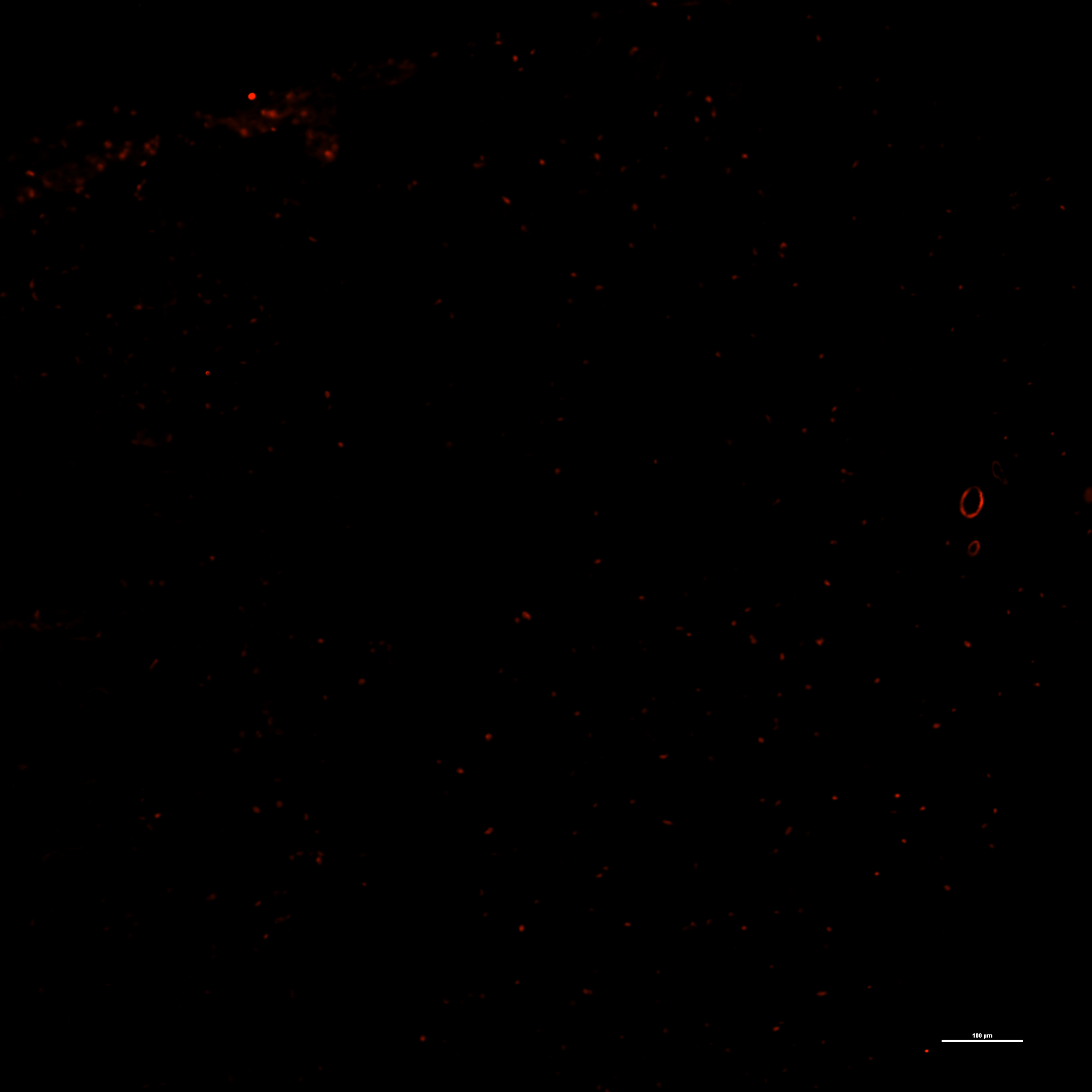

Supplement: Supplementary file 4 — Source data Fig. 1 [file 44319_2024_197_MOESM4_ESM.zip › Figure 1/1A/Injured_Pax7.tif]

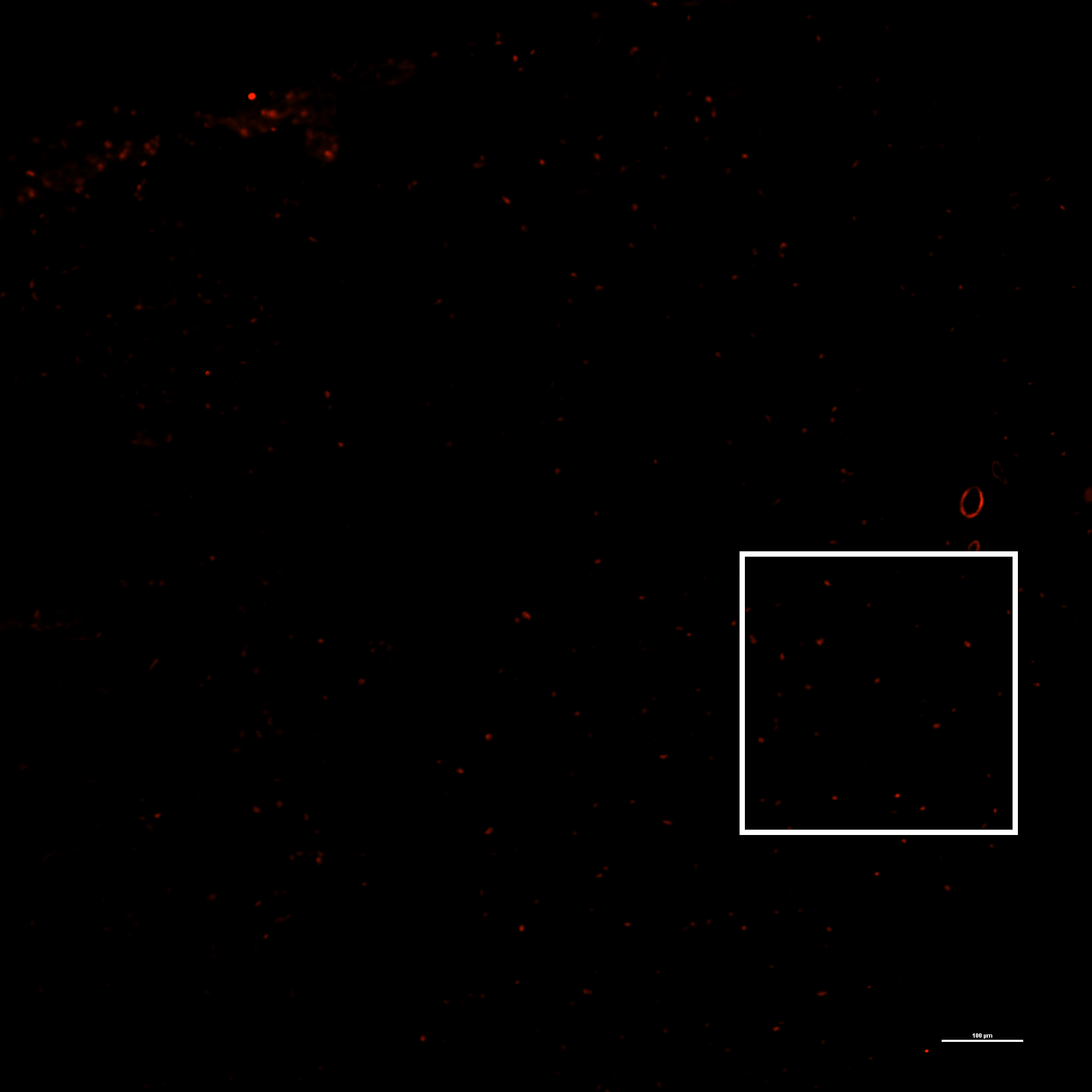

Supplement: Supplementary file 4 — Source data Fig. 1 [file 44319_2024_197_MOESM4_ESM.zip › Figure 1/1A/Injured_Pax7_with_box.tif]

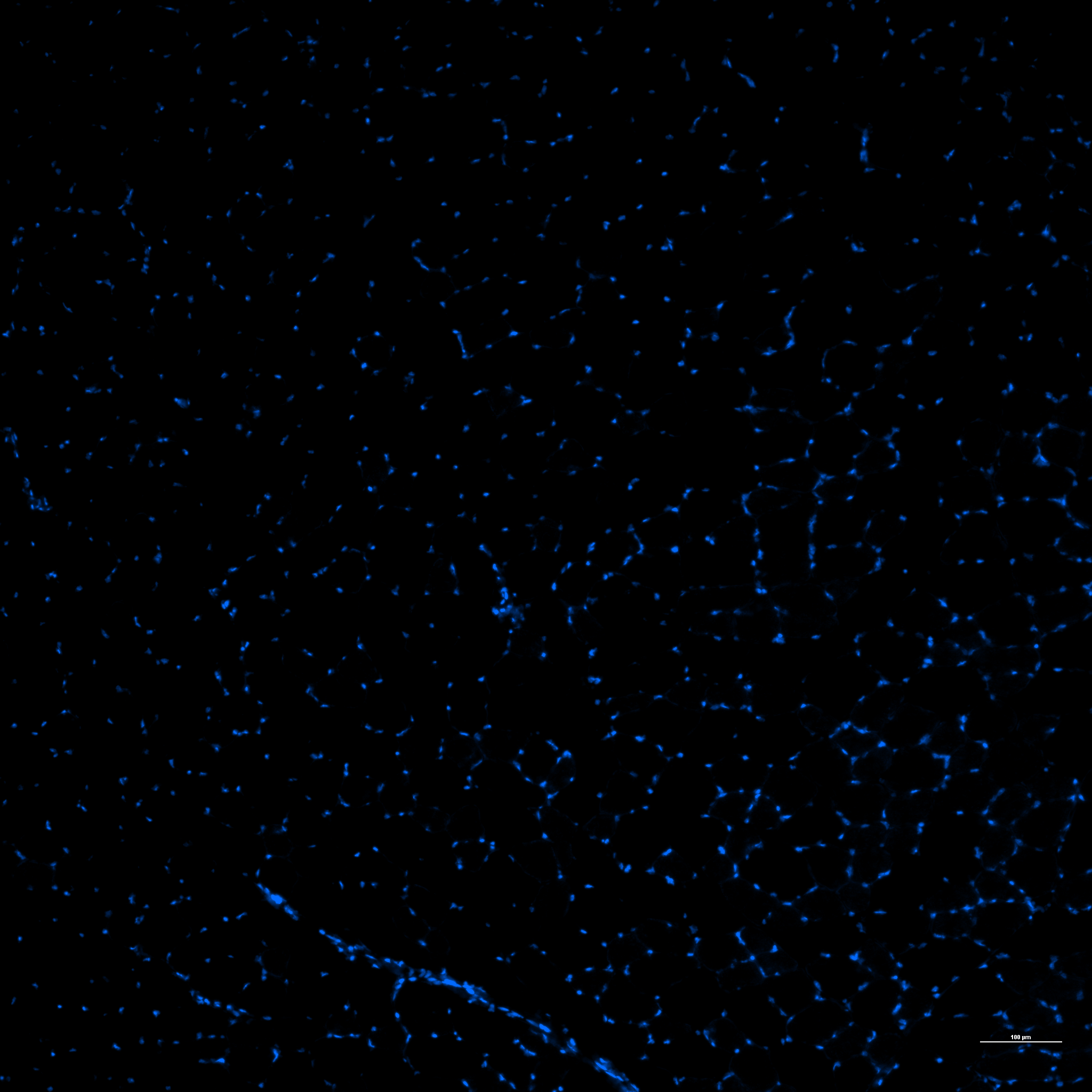

Supplement: Supplementary file 4 — Source data Fig. 1 [file 44319_2024_197_MOESM4_ESM.zip › Figure 1/1A/Uninjured_DAPI.tif]

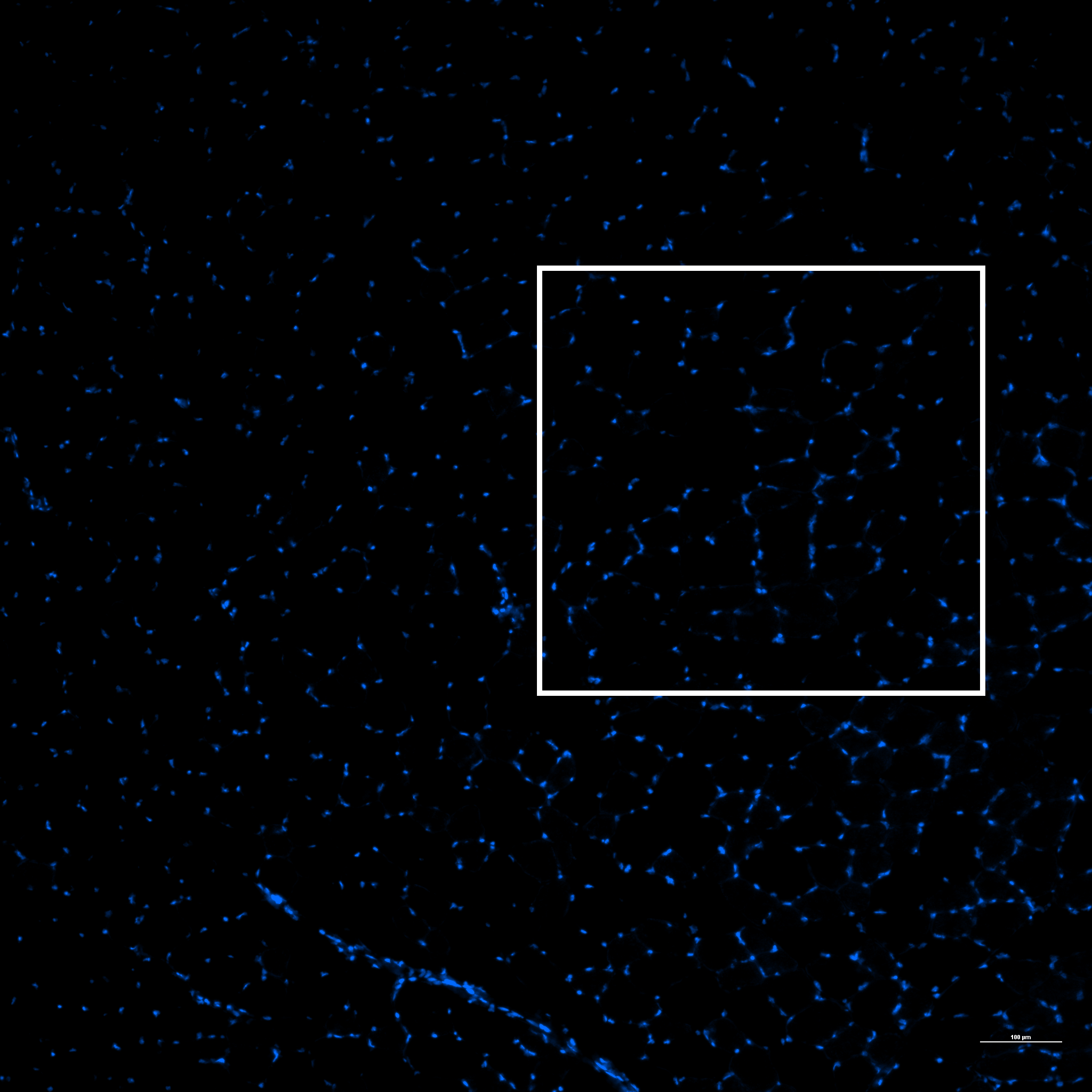

Supplement: Supplementary file 4 — Source data Fig. 1 [file 44319_2024_197_MOESM4_ESM.zip › Figure 1/1A/Uninjured_DAPI_with_box.tif]

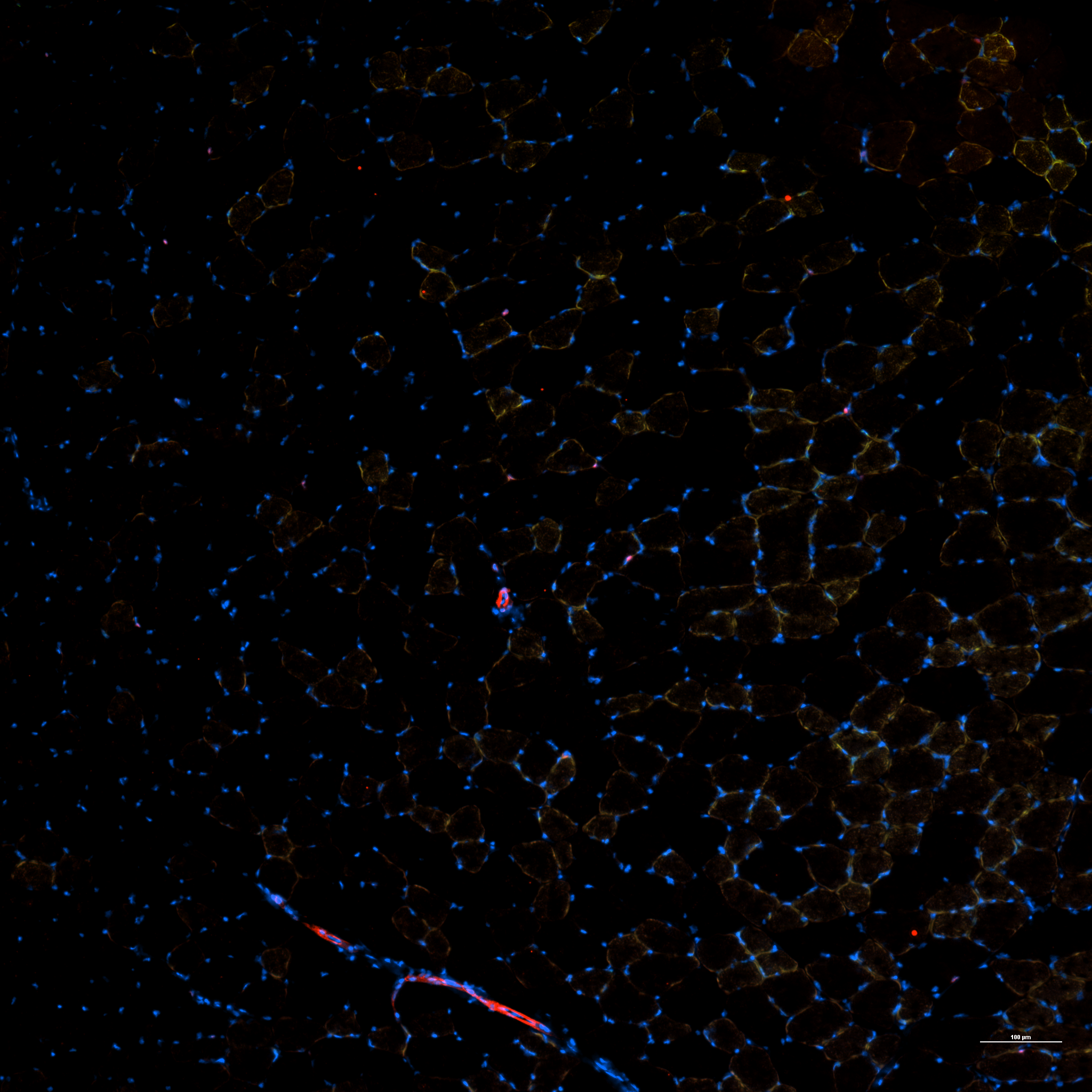

Supplement: Supplementary file 4 — Source data Fig. 1 [file 44319_2024_197_MOESM4_ESM.zip › Figure 1/1A/Uninjured_Merged.tif]

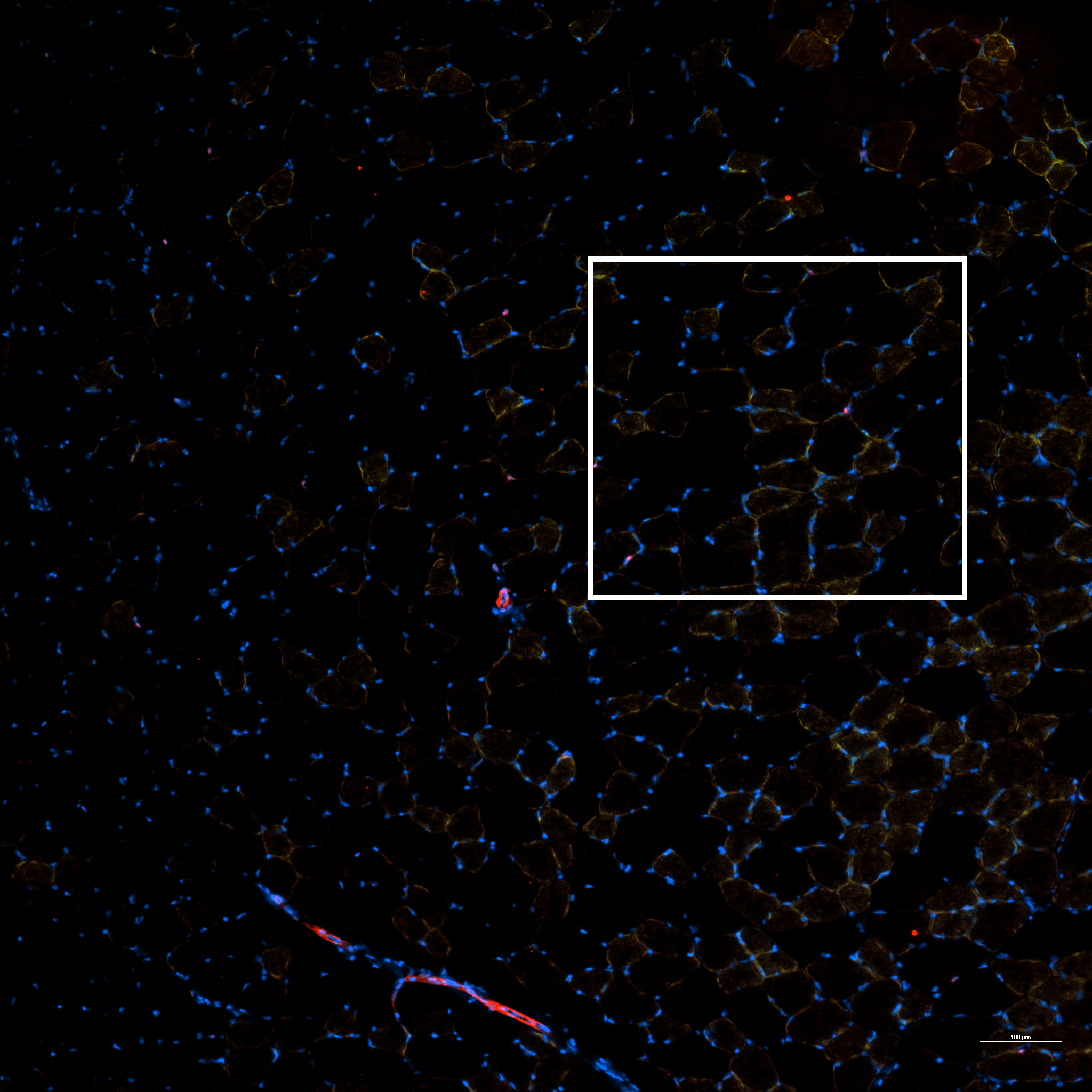

Supplement: Supplementary file 4 — Source data Fig. 1 [file 44319_2024_197_MOESM4_ESM.zip › Figure 1/1A/Uninjured_Merged_with_box.tif]

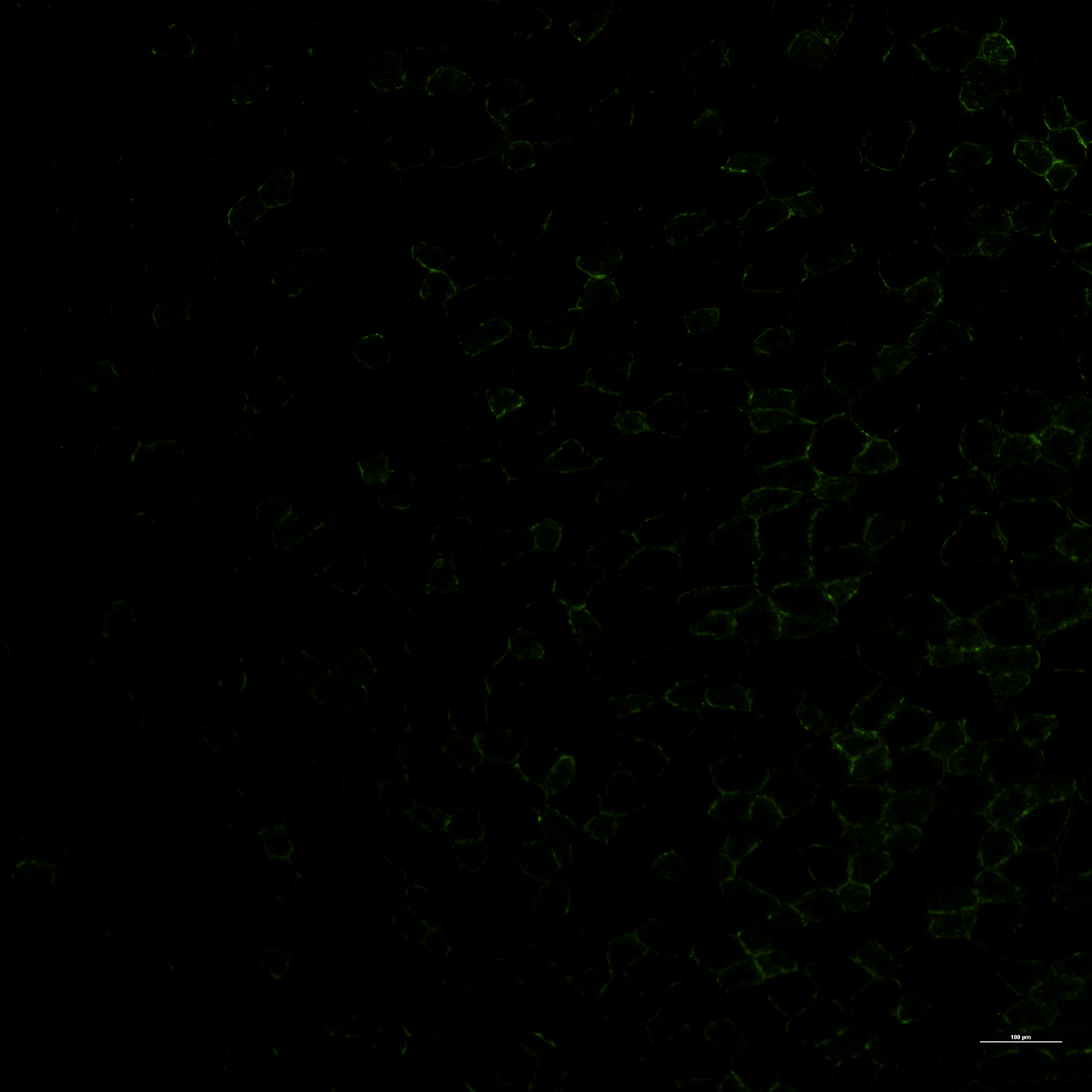

Supplement: Supplementary file 4 — Source data Fig. 1 [file 44319_2024_197_MOESM4_ESM.zip › Figure 1/1A/Uninjured_p-IRE1a.tif]

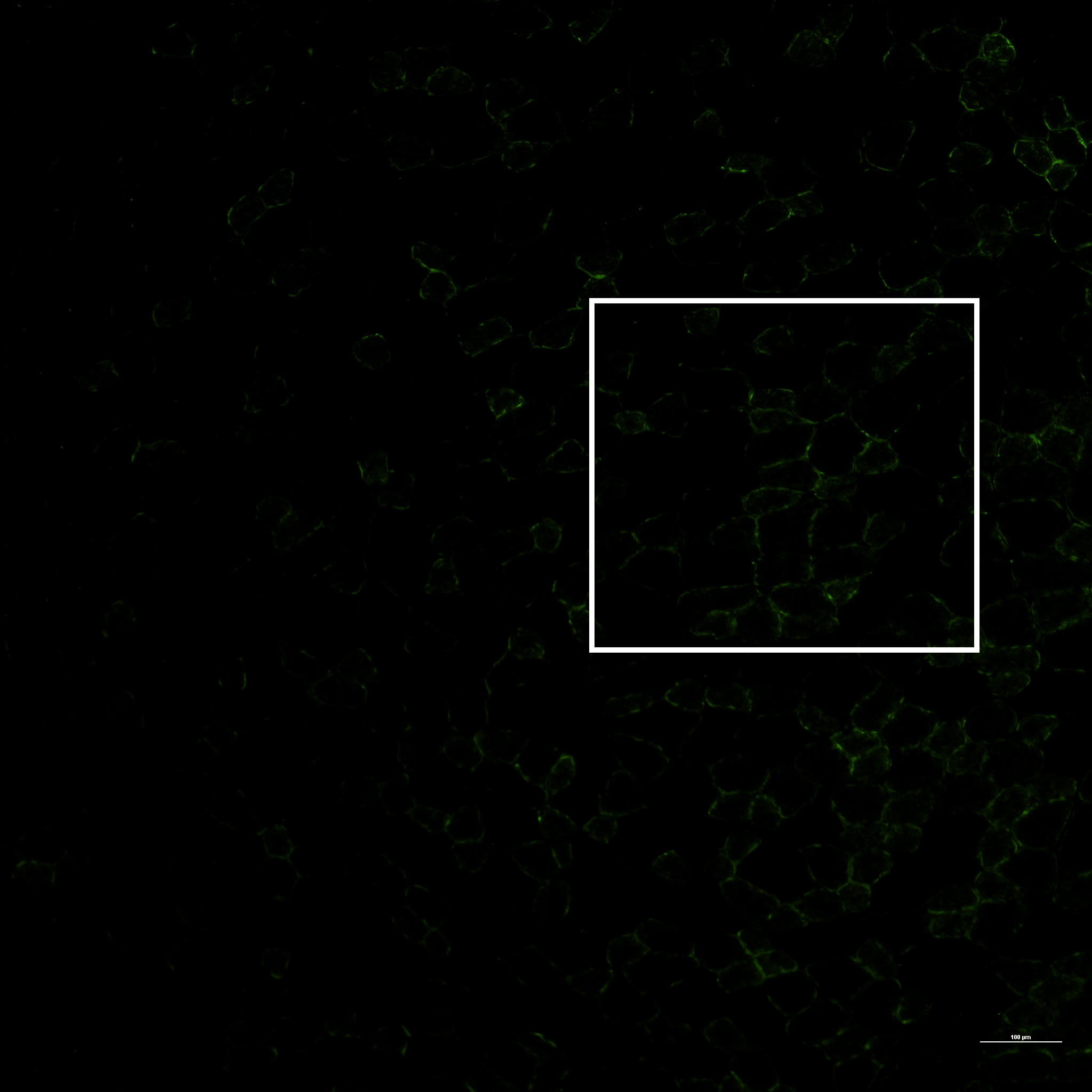

Supplement: Supplementary file 4 — Source data Fig. 1 [file 44319_2024_197_MOESM4_ESM.zip › Figure 1/1A/Uninjured_p-IRE1a_with_box.tif]

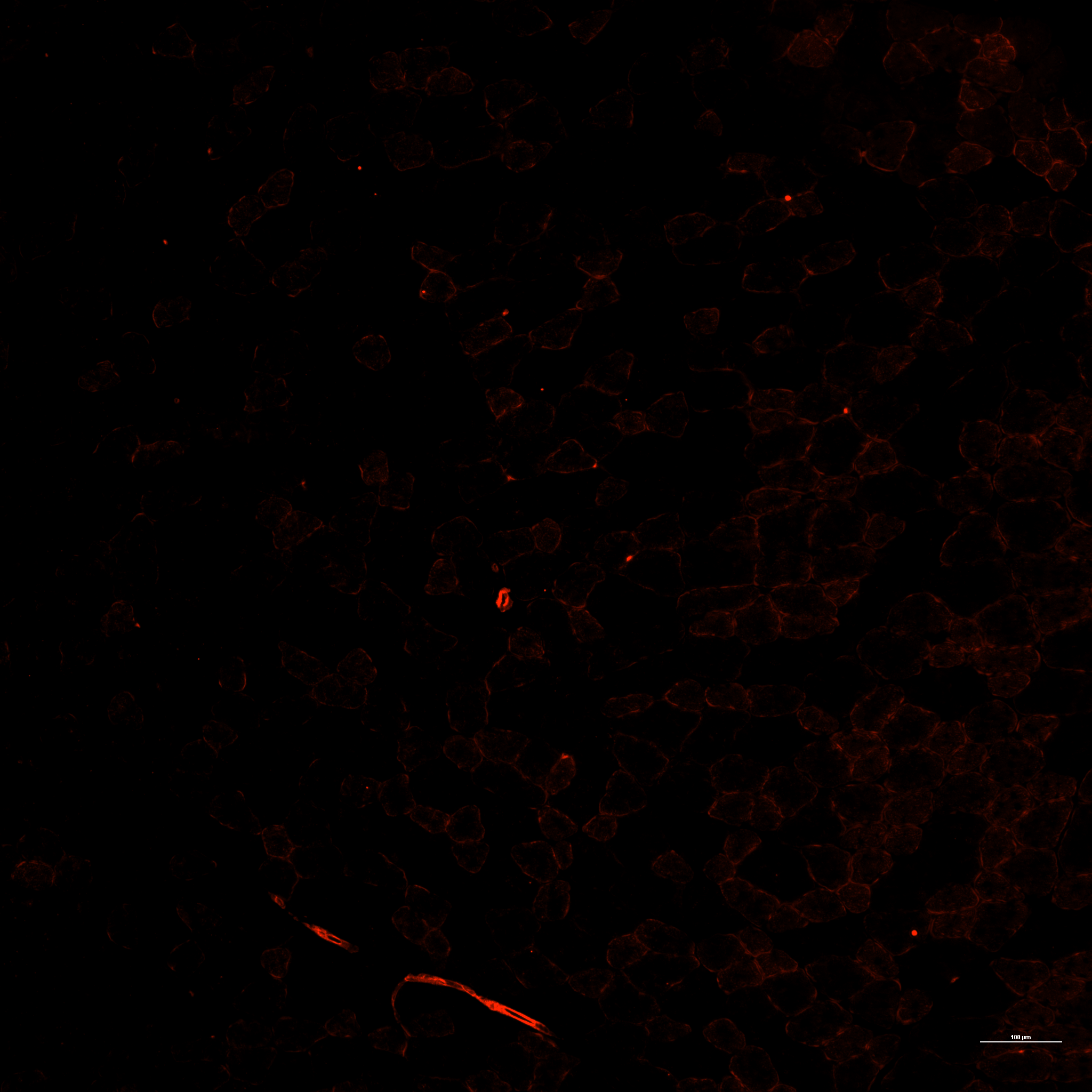

Supplement: Supplementary file 4 — Source data Fig. 1 [file 44319_2024_197_MOESM4_ESM.zip › Figure 1/1A/Uninjured_Pax7.tif]

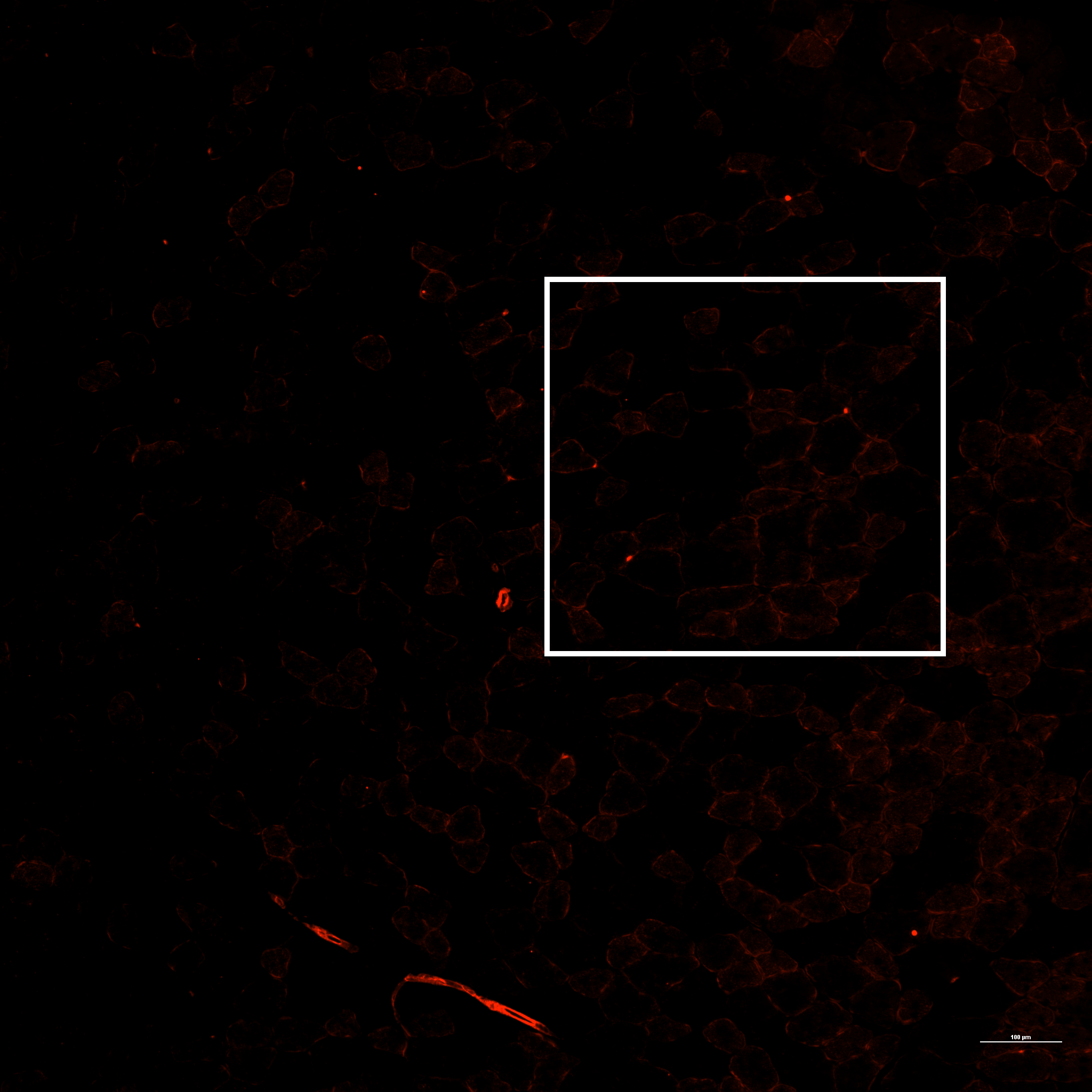

Supplement: Supplementary file 4 — Source data Fig. 1 [file 44319_2024_197_MOESM4_ESM.zip › Figure 1/1A/Uninjured_Pax7_with_box.tif]

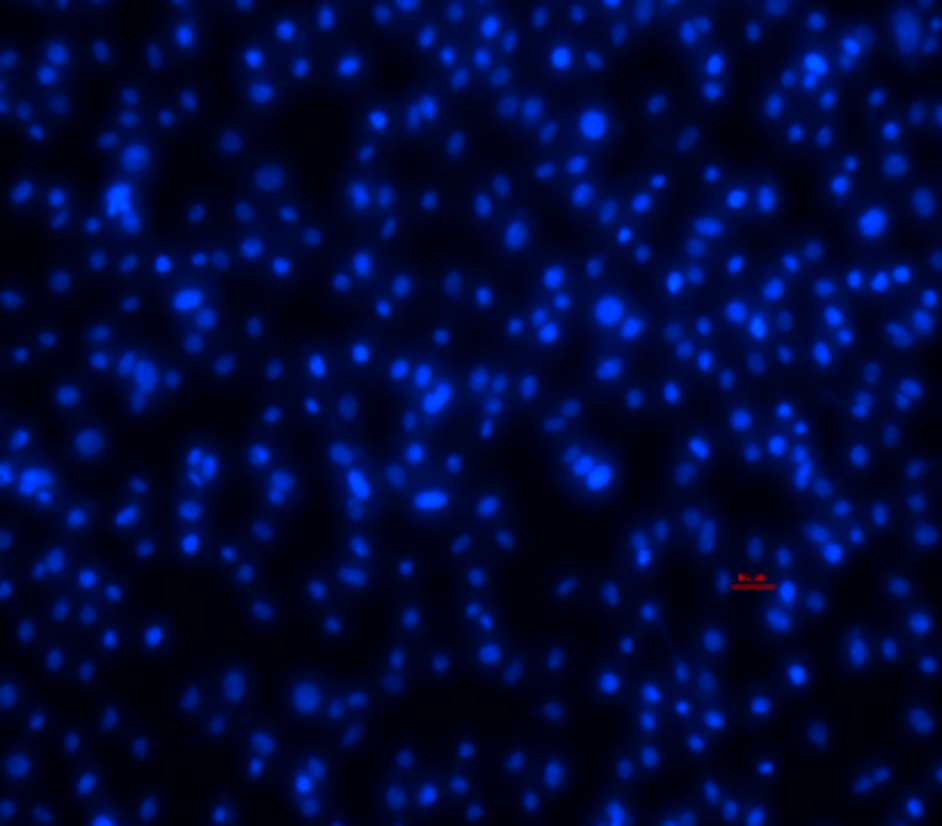

Supplement: Supplementary file 4 — Source data Fig. 1 [file 44319_2024_197_MOESM4_ESM.zip › Figure 1/1C/Staining-DAPI-Lower Panel.tif]

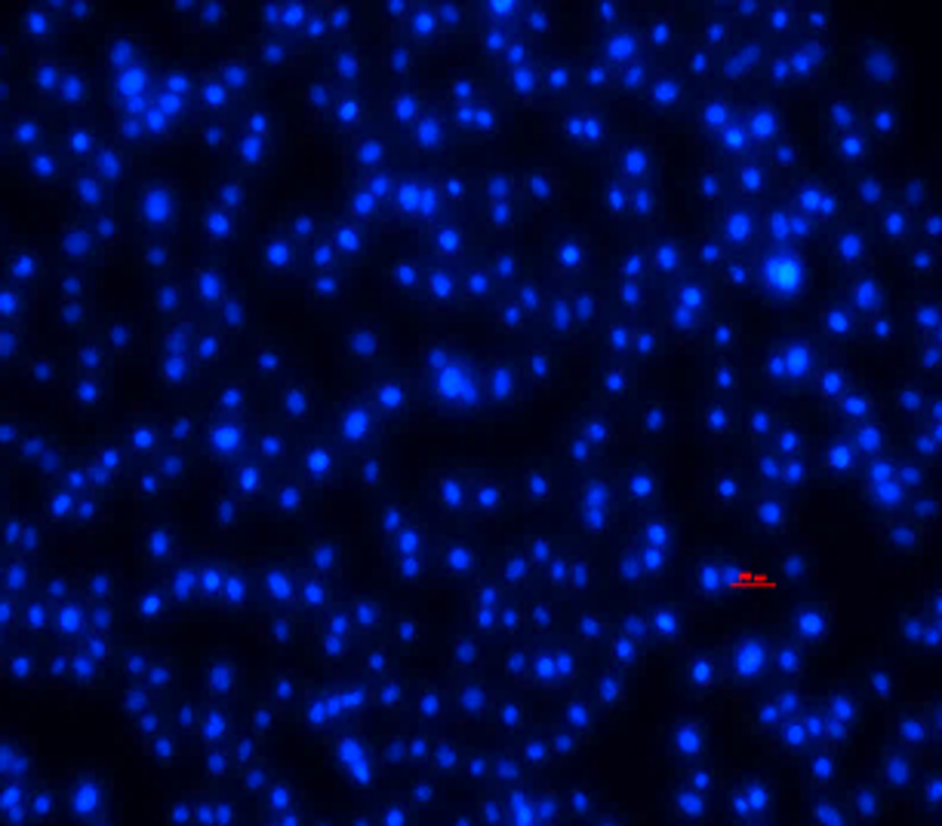

Supplement: Supplementary file 4 — Source data Fig. 1 [file 44319_2024_197_MOESM4_ESM.zip › Figure 1/1C/Staining-DAPI-Upper Panel.tif]

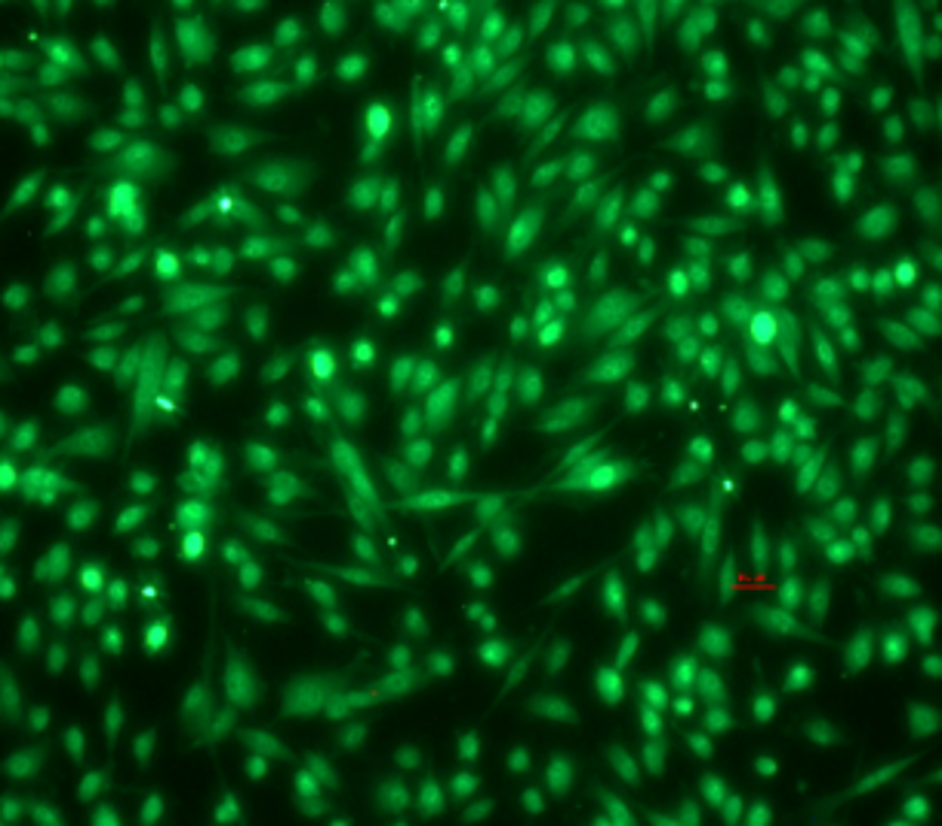

Supplement: Supplementary file 4 — Source data Fig. 1 [file 44319_2024_197_MOESM4_ESM.zip › Figure 1/1C/Staining-IRE1a-Lower Panel.tif]

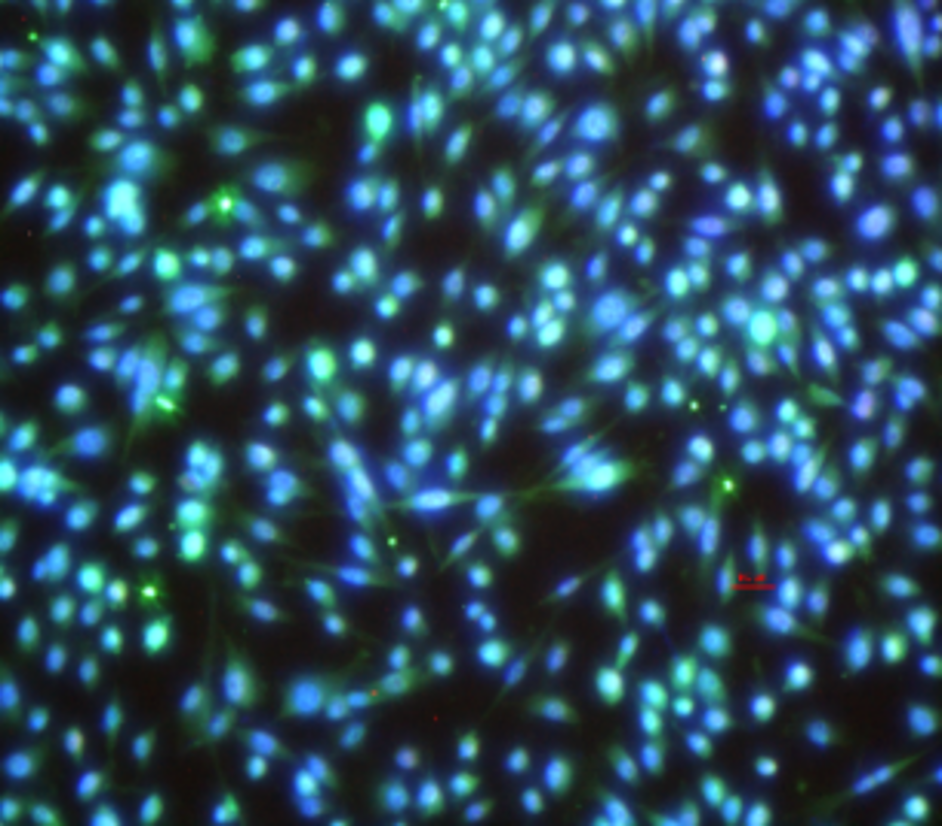

Supplement: Supplementary file 4 — Source data Fig. 1 [file 44319_2024_197_MOESM4_ESM.zip › Figure 1/1C/Staining-Merged-Lower Panel.tif]

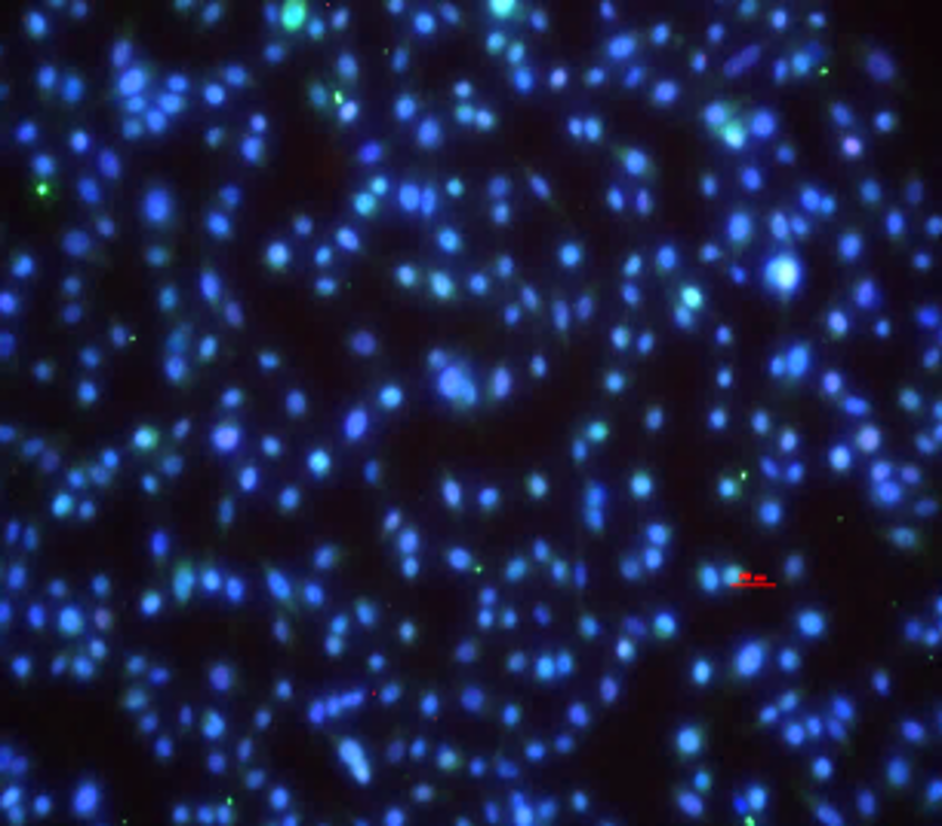

Supplement: Supplementary file 4 — Source data Fig. 1 [file 44319_2024_197_MOESM4_ESM.zip › Figure 1/1C/Staining-Merged-Upper Panel.tif]

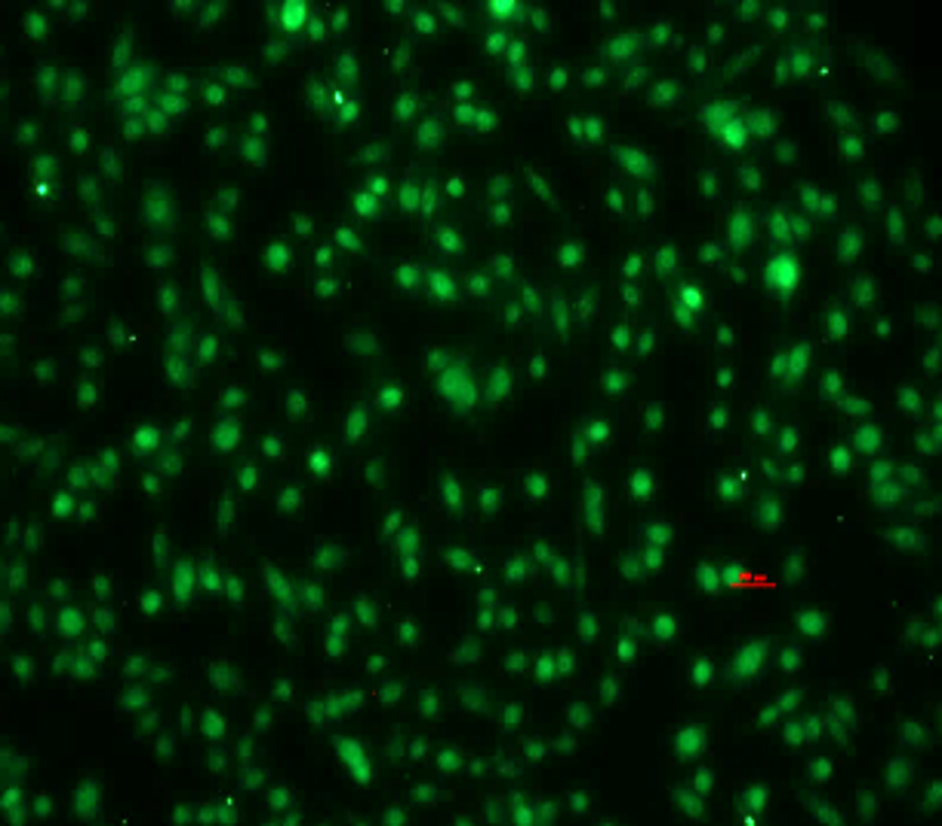

Supplement: Supplementary file 4 — Source data Fig. 1 [file 44319_2024_197_MOESM4_ESM.zip › Figure 1/1C/Staining-p-IRE1a-Upper Panel.tif]

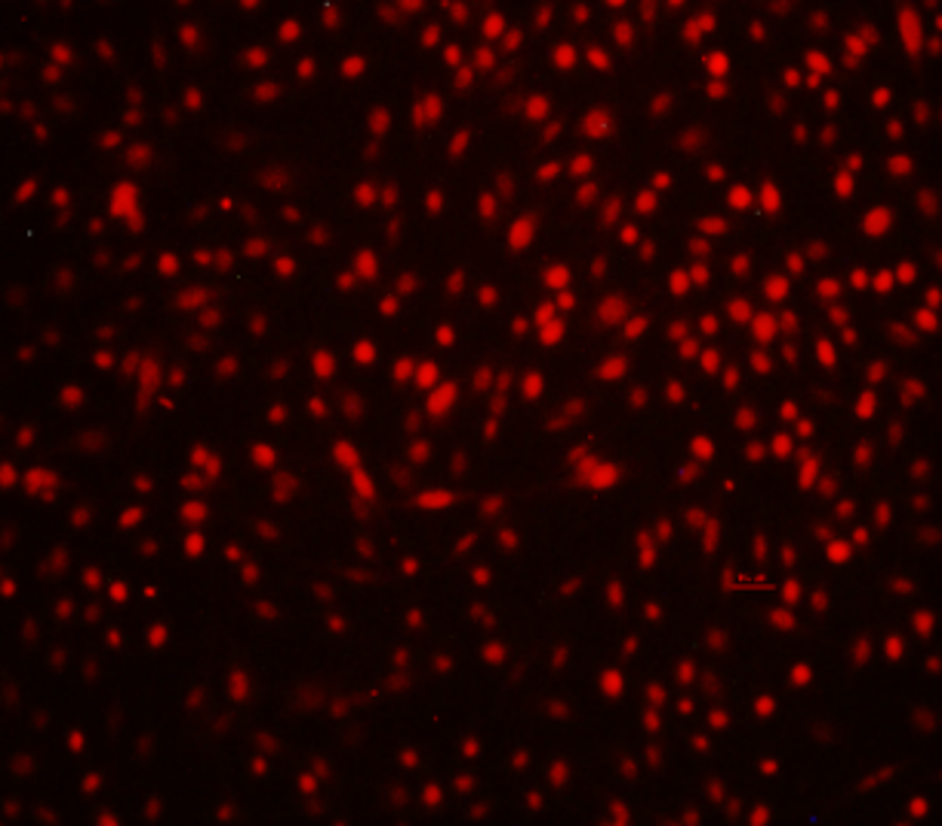

Supplement: Supplementary file 4 — Source data Fig. 1 [file 44319_2024_197_MOESM4_ESM.zip › Figure 1/1C/Staining-Pax7-Lower Panel.tif]

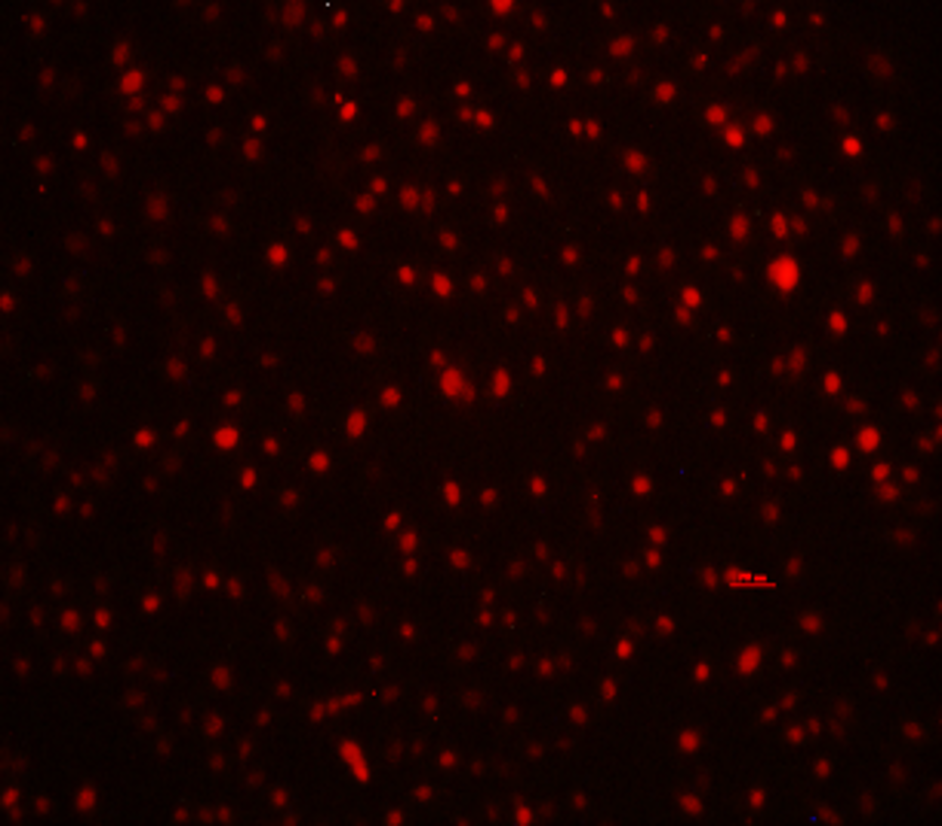

Supplement: Supplementary file 4 — Source data Fig. 1 [file 44319_2024_197_MOESM4_ESM.zip › Figure 1/1C/Staining-Pax7-Upper Panel.tif]

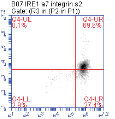

Supplement: Supplementary file 4 — Source data Fig. 1 [file 44319_2024_197_MOESM4_ESM.zip › Figure 1/1D/IRE1a.tif]

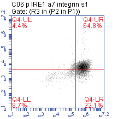

Supplement: Supplementary file 4 — Source data Fig. 1 [file 44319_2024_197_MOESM4_ESM.zip › Figure 1/1D/p-IRE1a.tif]

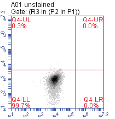

Supplement: Supplementary file 4 — Source data Fig. 1 [file 44319_2024_197_MOESM4_ESM.zip › Figure 1/1D/Unstained.tif]

## Slide 1
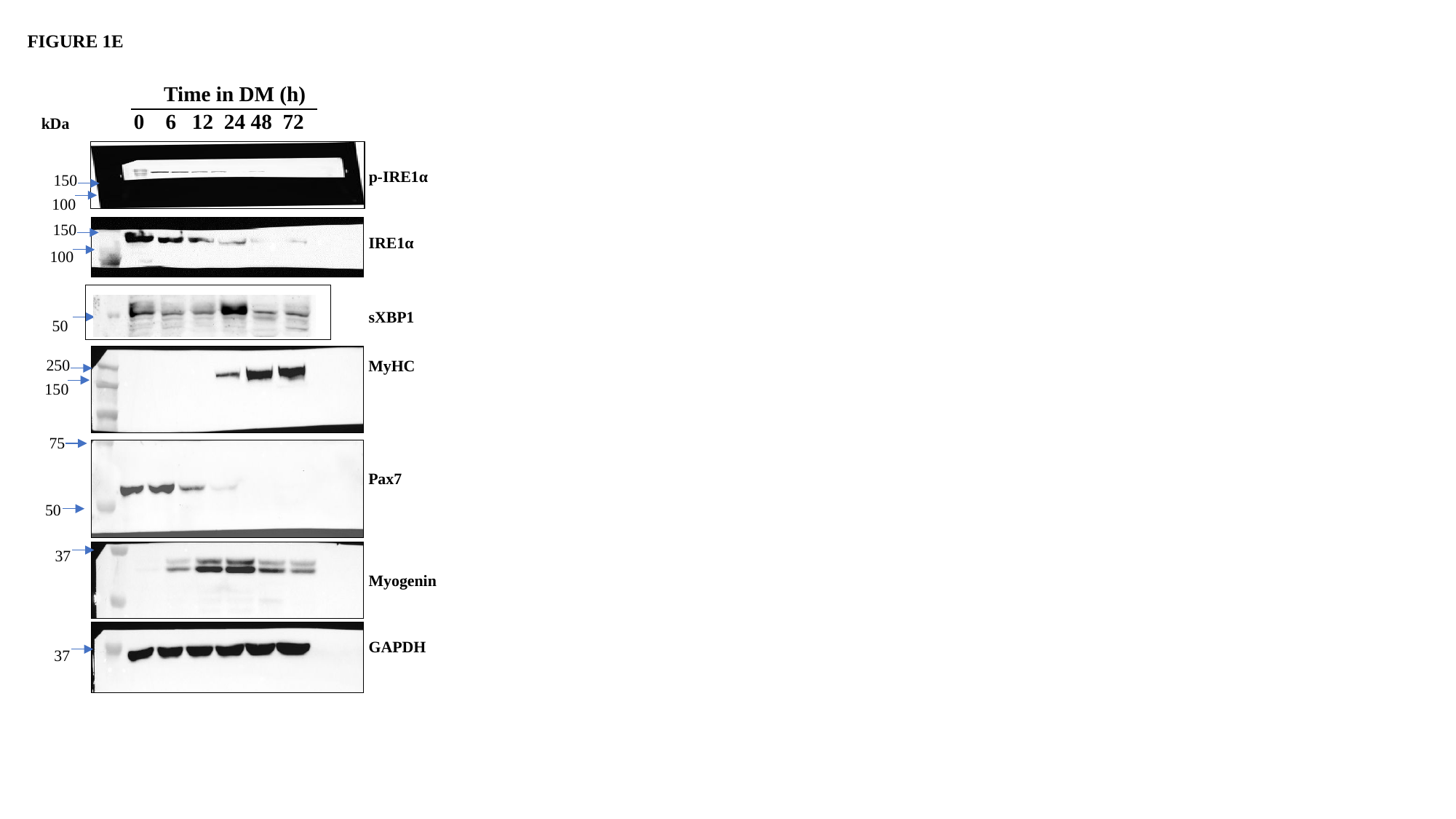

FIGURE 1E
Time in DM (h)
 0 6 12 24 48 72
kDa
p-IRE1α
150
100
150
IRE1α
100
sXBP1
50
250
MyHC
150
75
Pax7
50
37
Myogenin
GAPDH
37

Supplement: Supplementary file 4 — Source data Fig. 1 [file 44319_2024_197_MOESM4_ESM.zip › Figure 1/1E/Western blot with annotation.pptx]

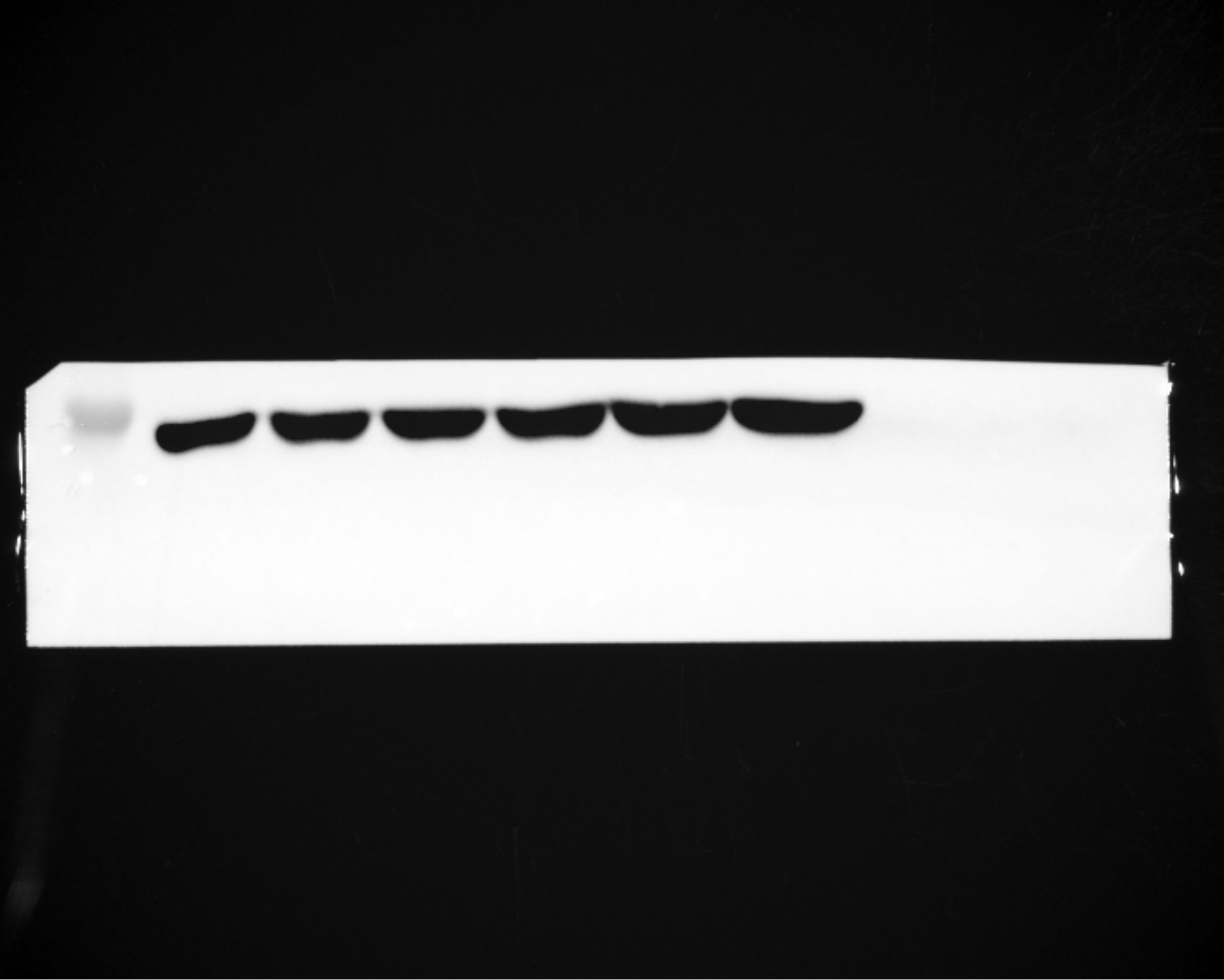

Supplement: Supplementary file 4 — Source data Fig. 1 [file 44319_2024_197_MOESM4_ESM.zip › Figure 1/1E/Western-GAPDH.tif]

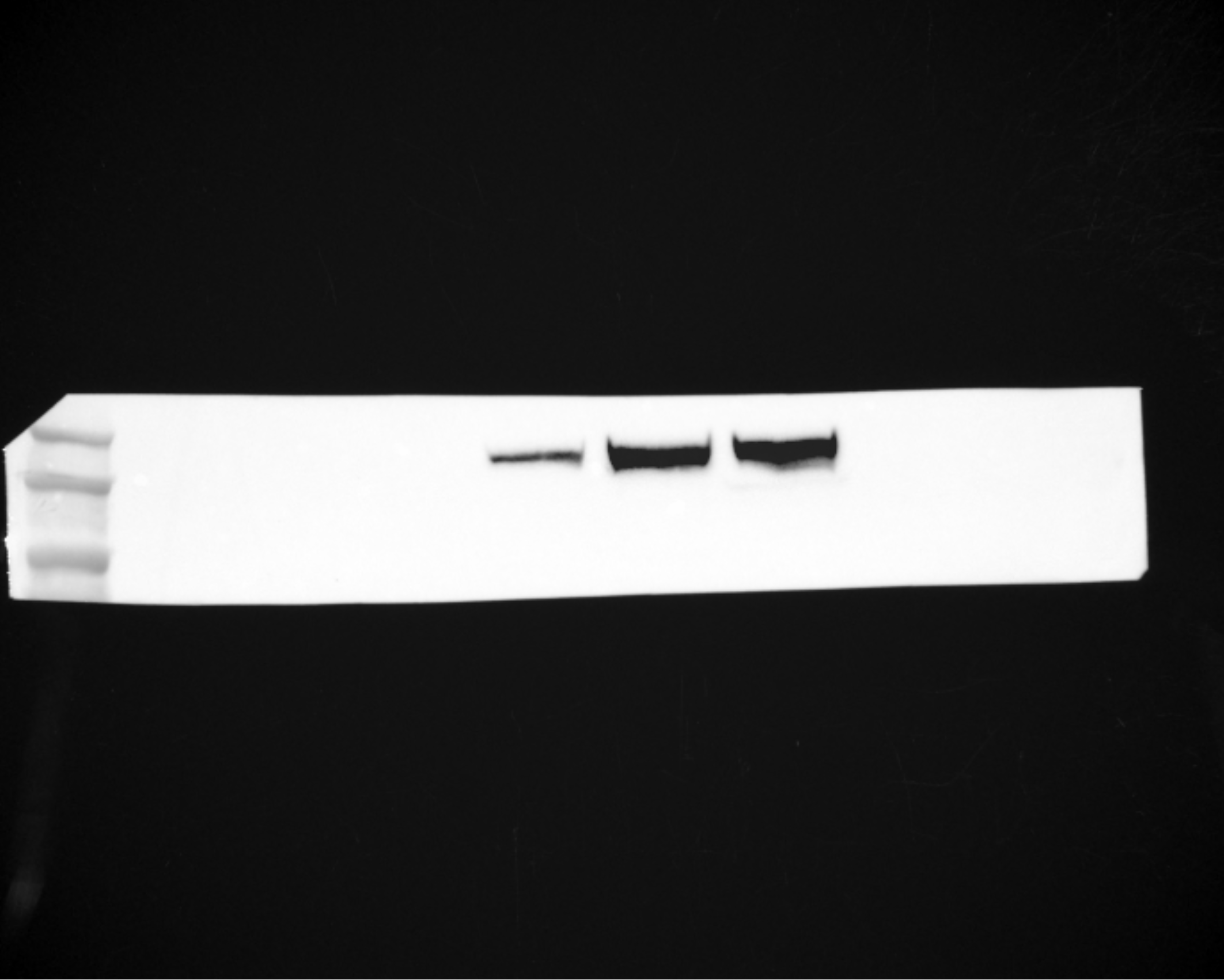

Supplement: Supplementary file 4 — Source data Fig. 1 [file 44319_2024_197_MOESM4_ESM.zip › Figure 1/1E/Western-MyHC.tif]

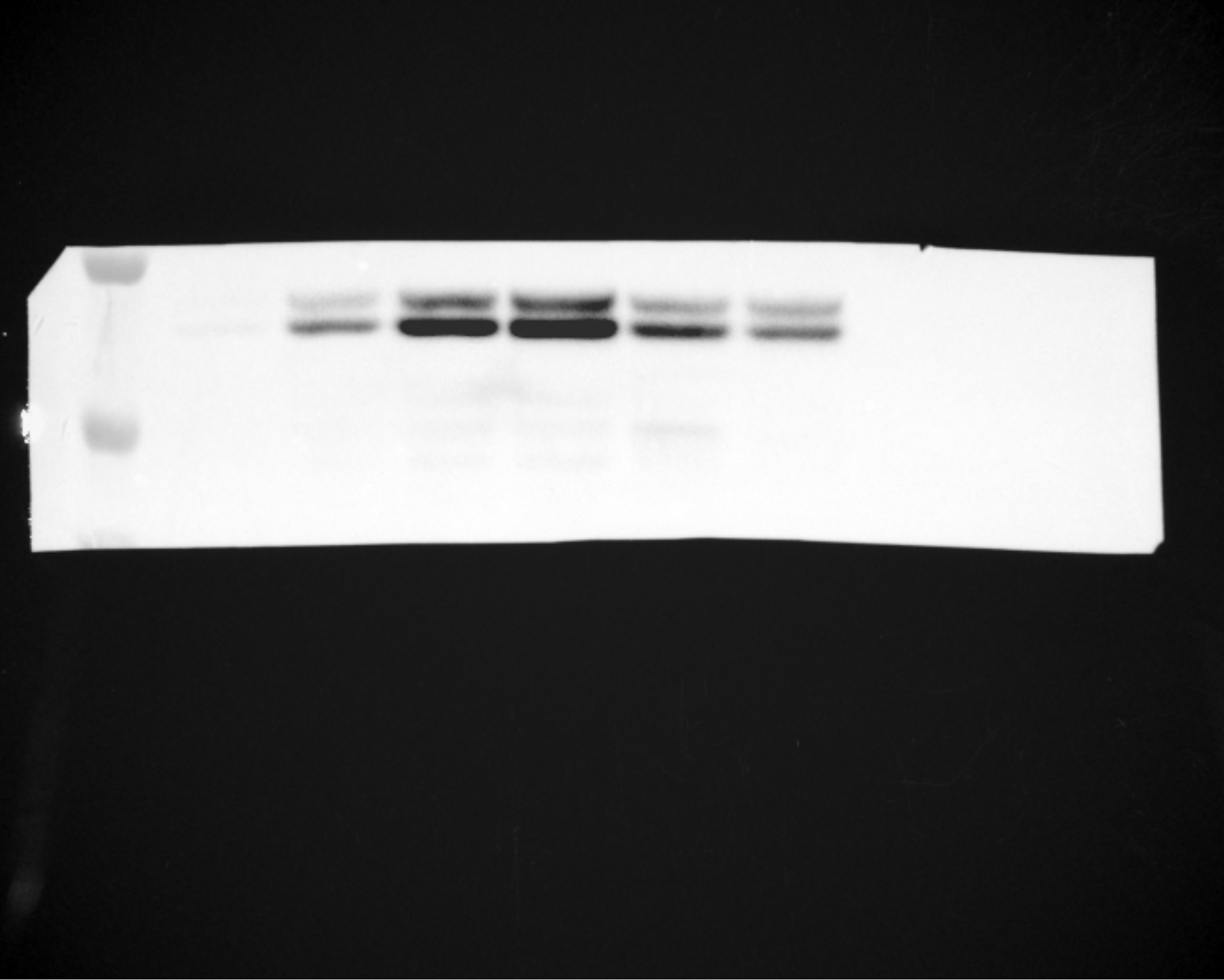

Supplement: Supplementary file 4 — Source data Fig. 1 [file 44319_2024_197_MOESM4_ESM.zip › Figure 1/1E/Western-Myogenin.tif]

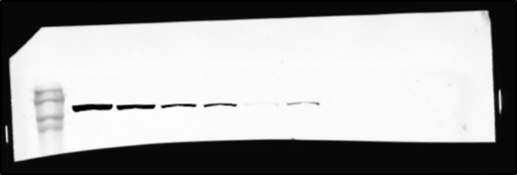

Supplement: Supplementary file 4 — Source data Fig. 1 [file 44319_2024_197_MOESM4_ESM.zip › Figure 1/1E/Western-p-IRE1.tif]

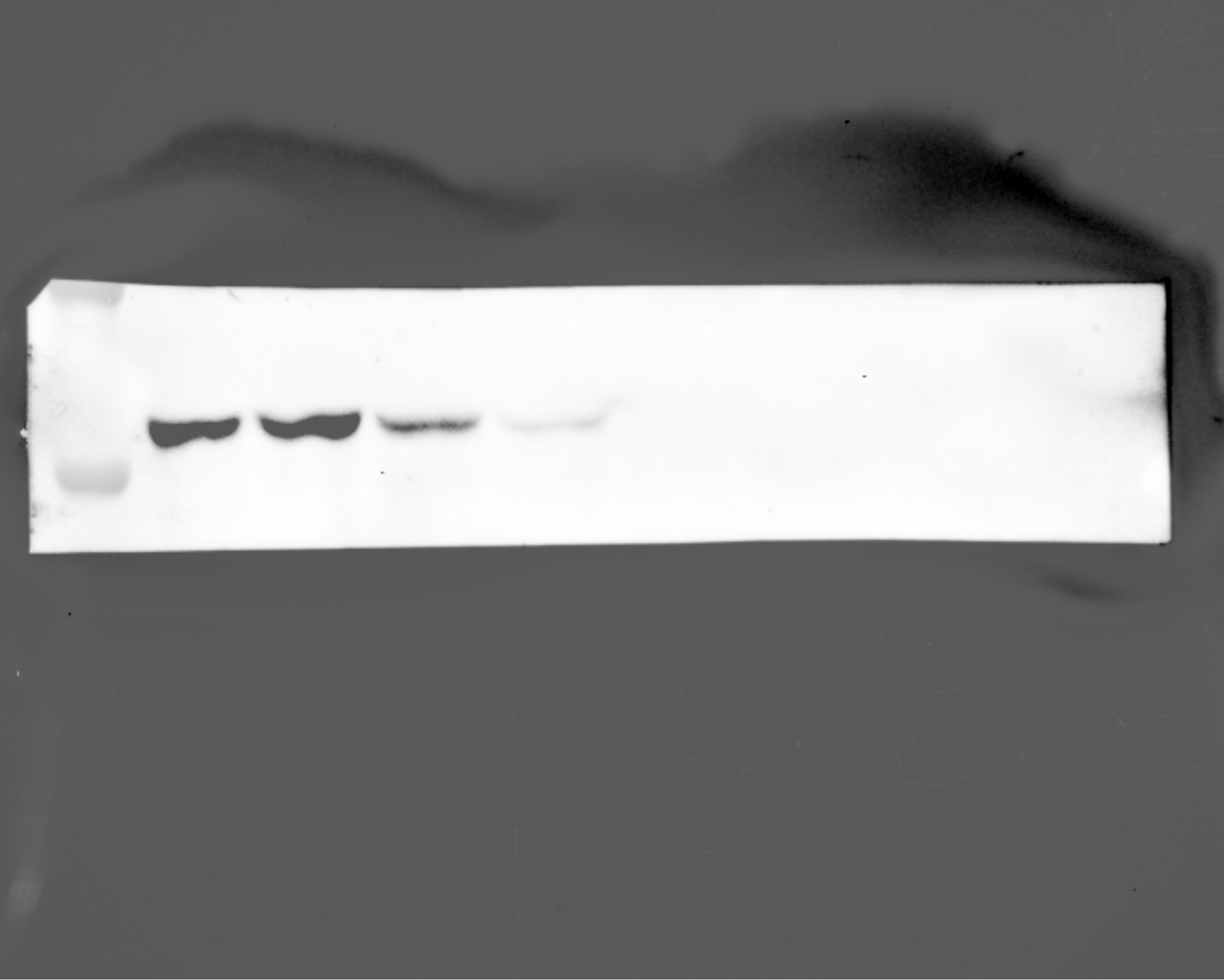

Supplement: Supplementary file 4 — Source data Fig. 1 [file 44319_2024_197_MOESM4_ESM.zip › Figure 1/1E/Western-Pax7.tif]

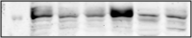

Supplement: Supplementary file 4 — Source data Fig. 1 [file 44319_2024_197_MOESM4_ESM.zip › Figure 1/1E/Western-sXBP1.tif]

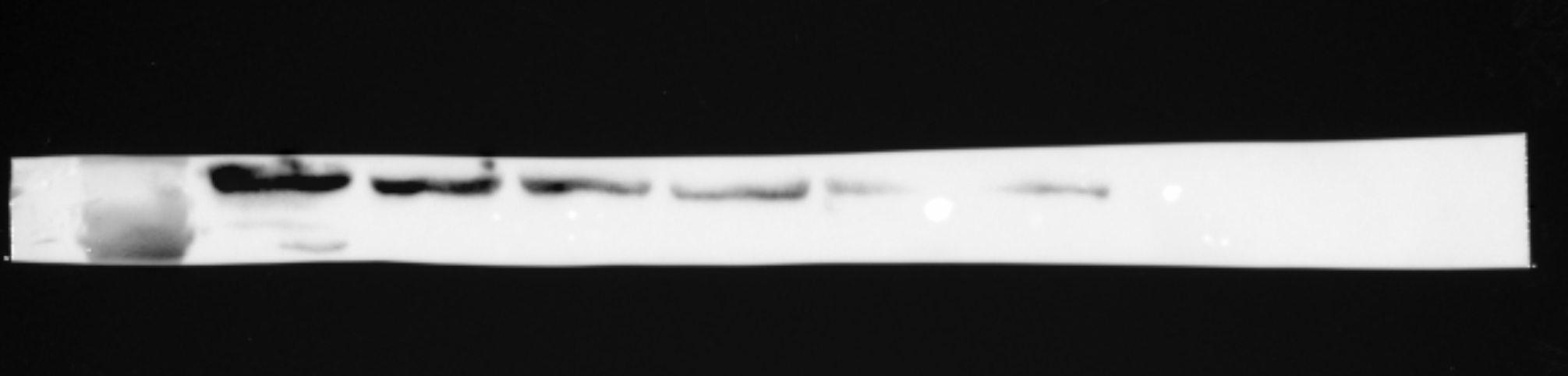

Supplement: Supplementary file 4 — Source data Fig. 1 [file 44319_2024_197_MOESM4_ESM.zip › Figure 1/1E/Western-Total IRE1a.tif]

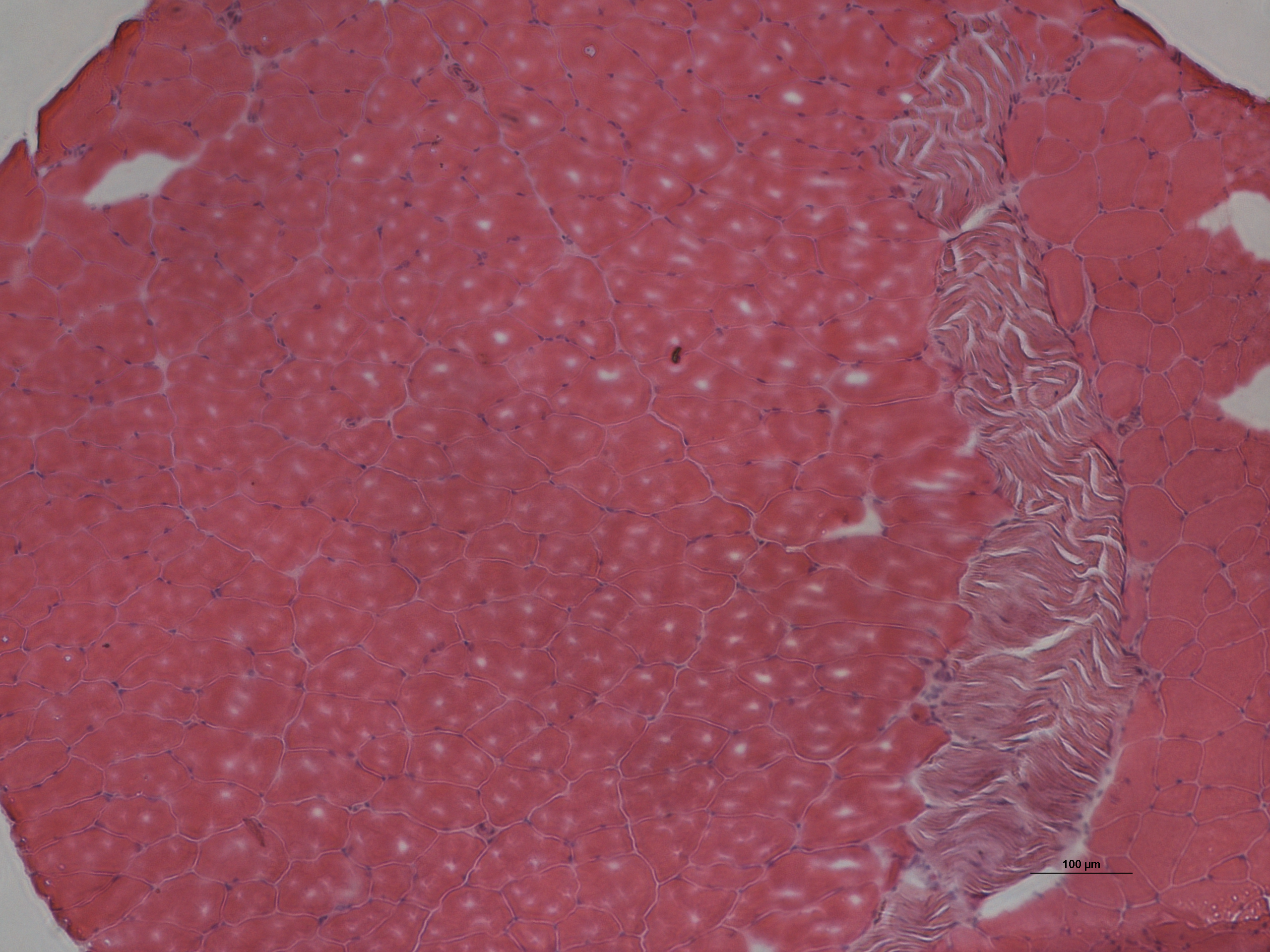

Supplement: Supplementary file 5 — Source data Fig. 2 [file 44319_2024_197_MOESM5_ESM.zip › Figure 2/2F/HE staining 0 and 5 days/Ern1 fl fl 0 day_1.tif]

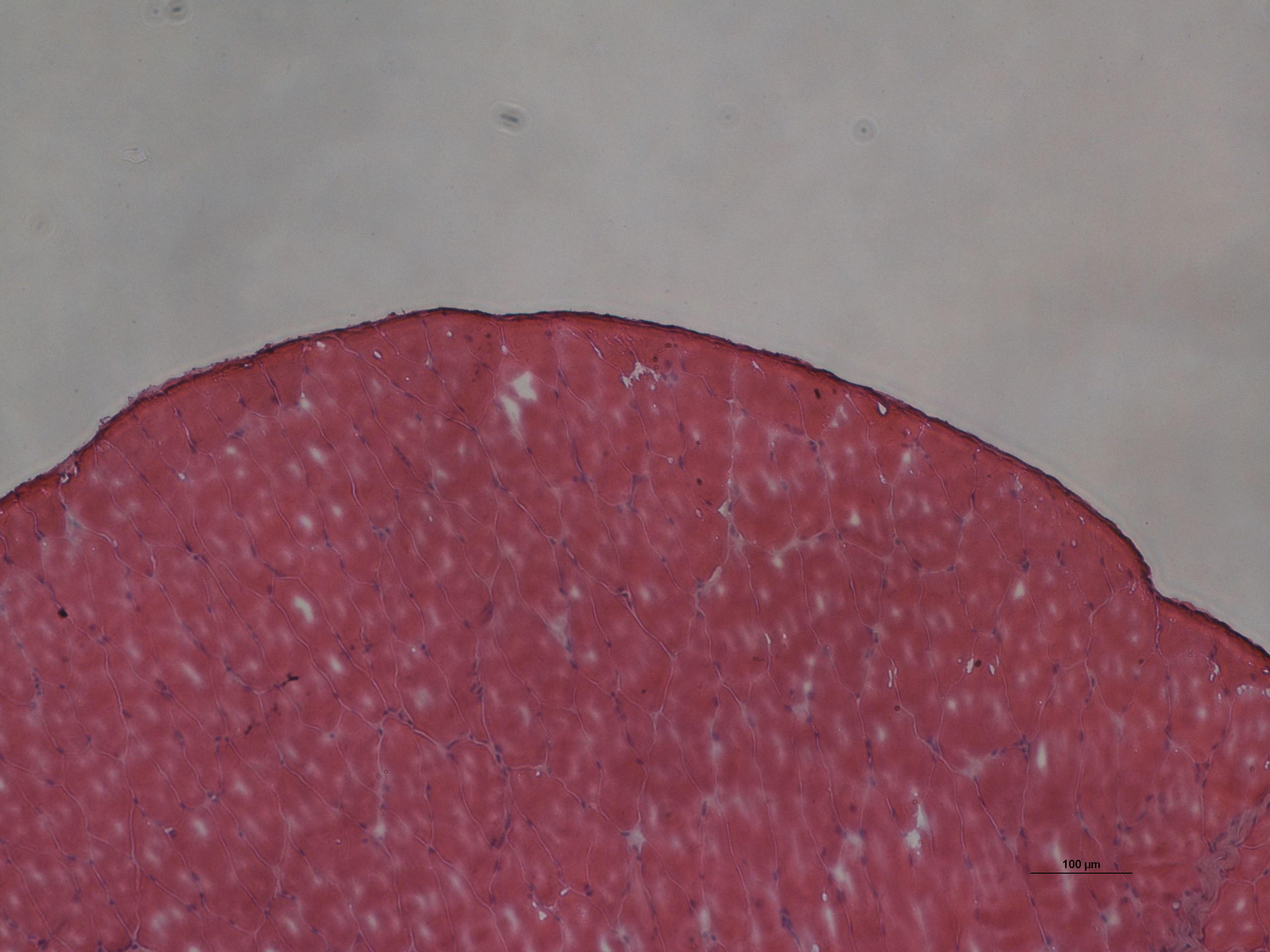

Supplement: Supplementary file 5 — Source data Fig. 2 [file 44319_2024_197_MOESM5_ESM.zip › Figure 2/2F/HE staining 0 and 5 days/Ern1 fl fl 0 day_2.tif]

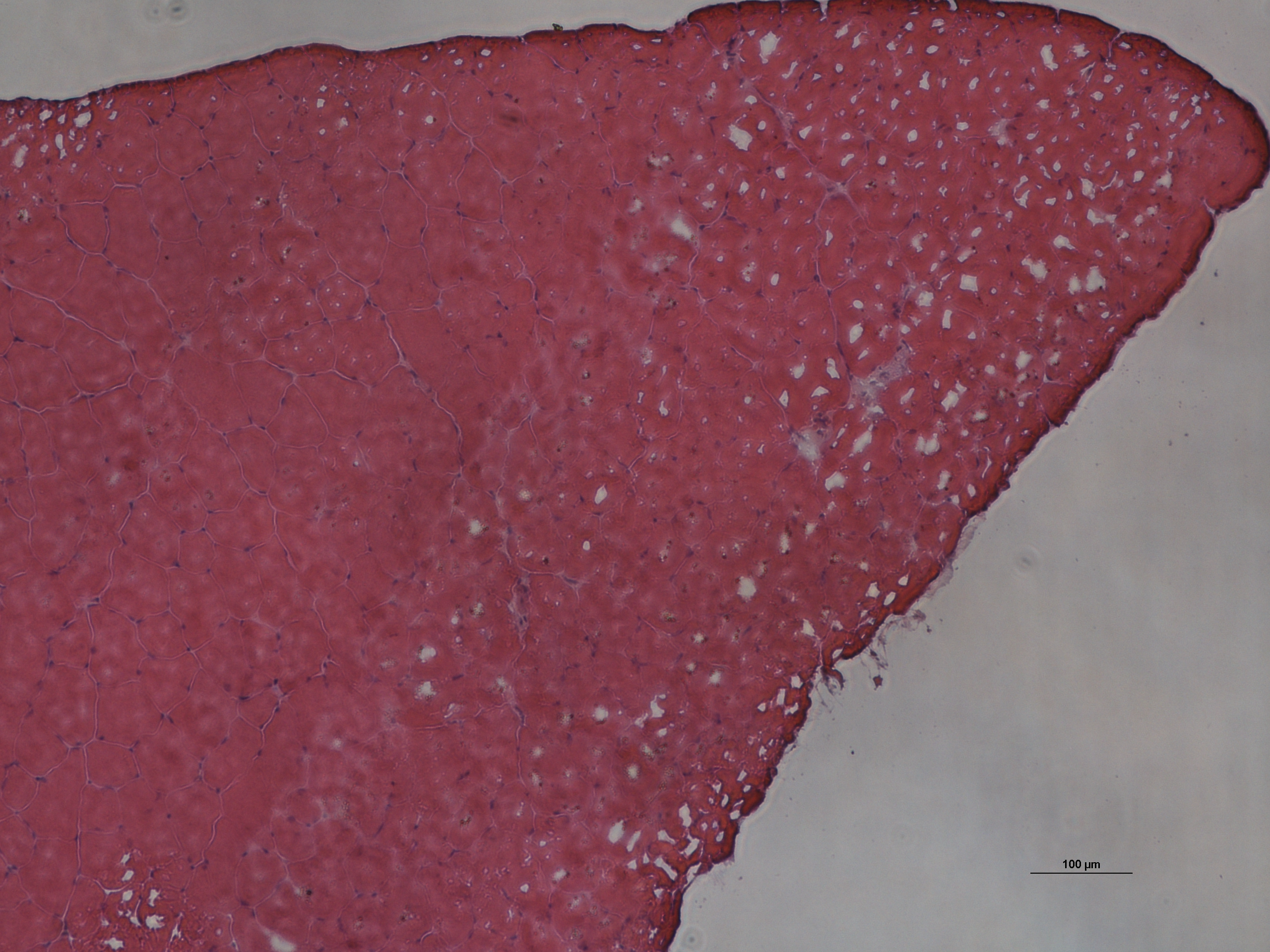

Supplement: Supplementary file 5 — Source data Fig. 2 [file 44319_2024_197_MOESM5_ESM.zip › Figure 2/2F/HE staining 0 and 5 days/Ern1 fl fl 0 day_3.tif]

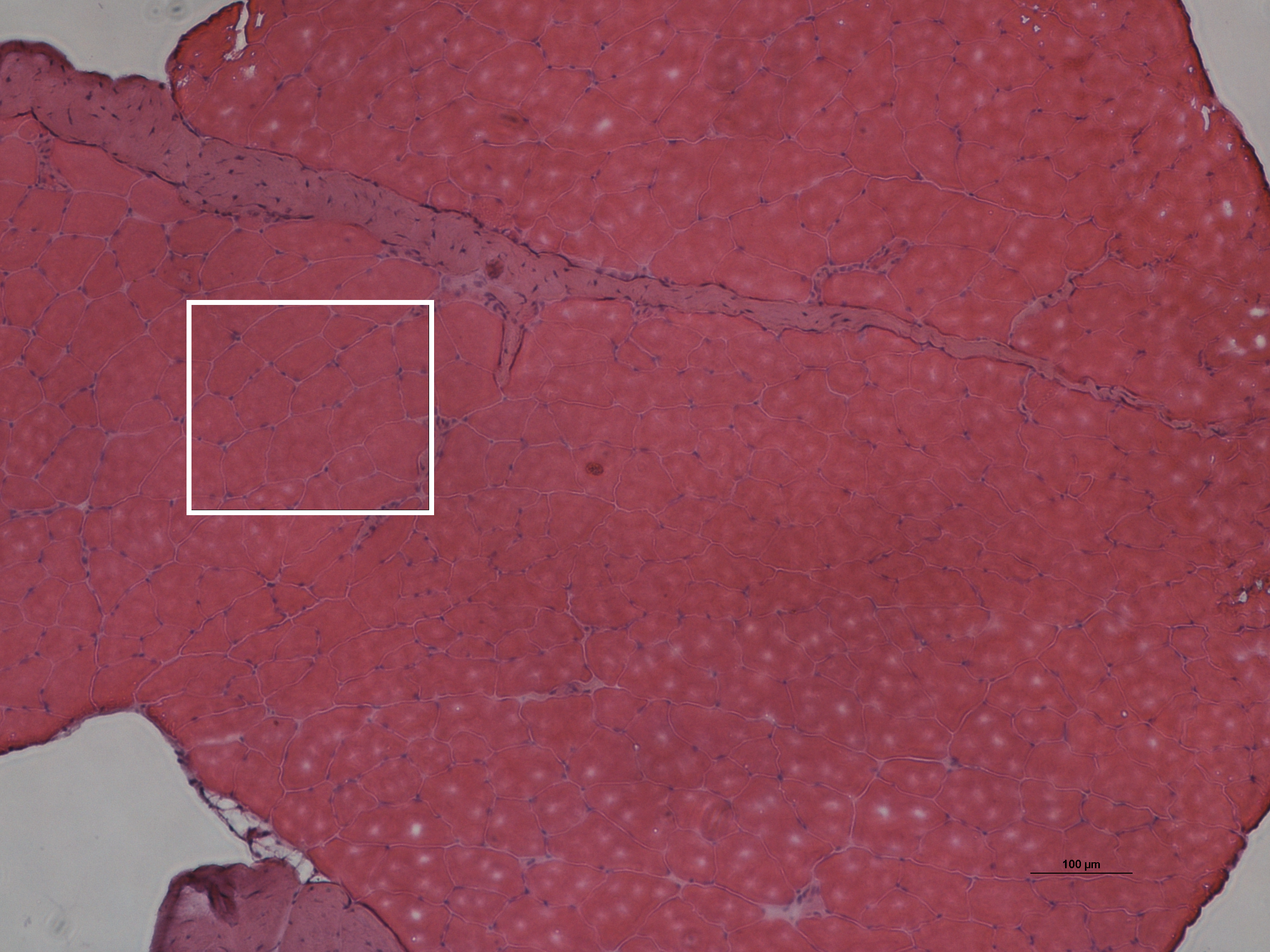

Supplement: Supplementary file 5 — Source data Fig. 2 [file 44319_2024_197_MOESM5_ESM.zip › Figure 2/2F/HE staining 0 and 5 days/Ern1 fl fl 0 day_Representative image with box.tiff]

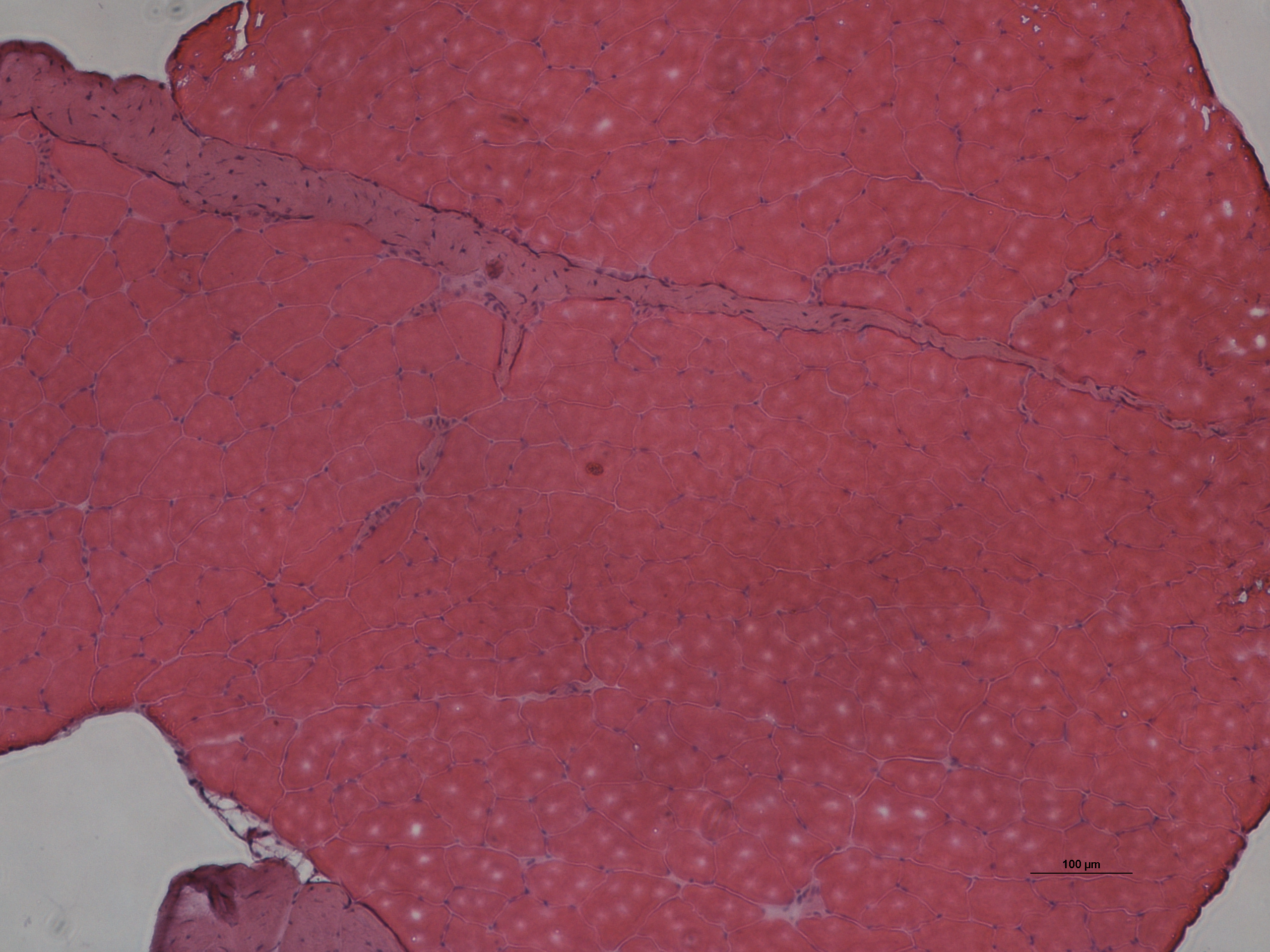

Supplement: Supplementary file 5 — Source data Fig. 2 [file 44319_2024_197_MOESM5_ESM.zip › Figure 2/2F/HE staining 0 and 5 days/Ern1 fl fl 0 day_Representative image.tif]

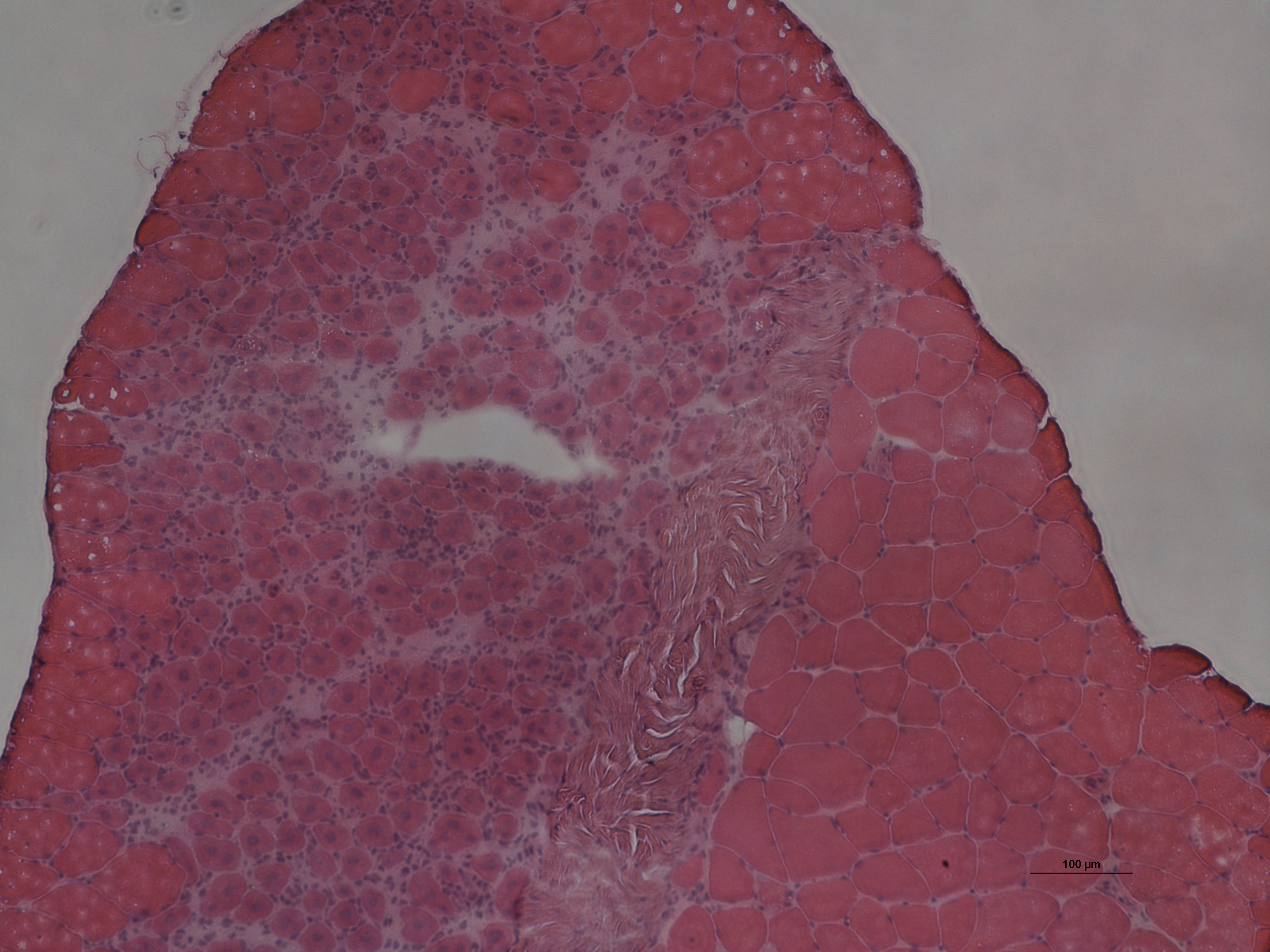

Supplement: Supplementary file 5 — Source data Fig. 2 [file 44319_2024_197_MOESM5_ESM.zip › Figure 2/2F/HE staining 0 and 5 days/Ern1 fl fl 5 days_1.tif]

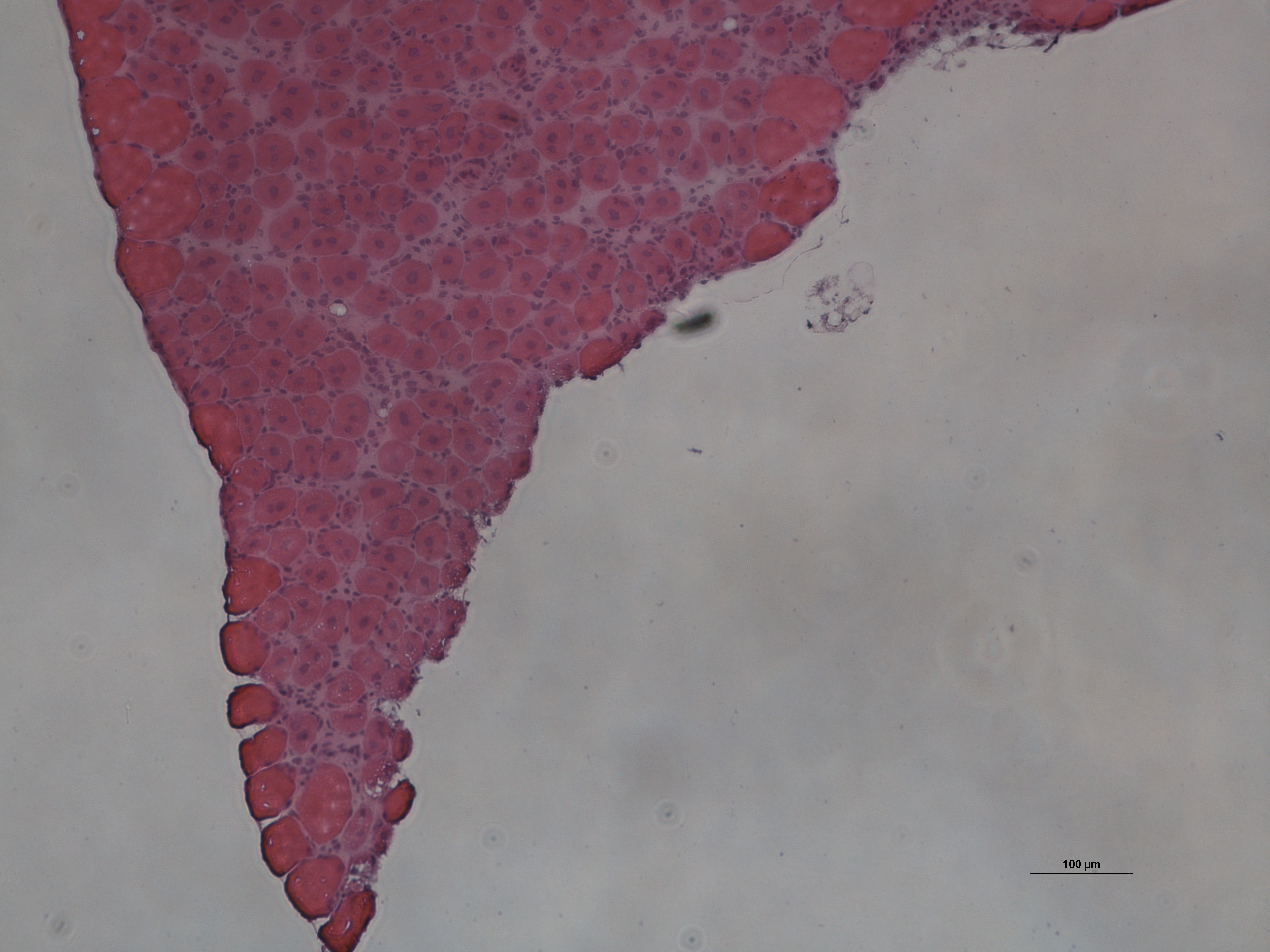

Supplement: Supplementary file 5 — Source data Fig. 2 [file 44319_2024_197_MOESM5_ESM.zip › Figure 2/2F/HE staining 0 and 5 days/Ern1 fl fl 5 days_2.tif]

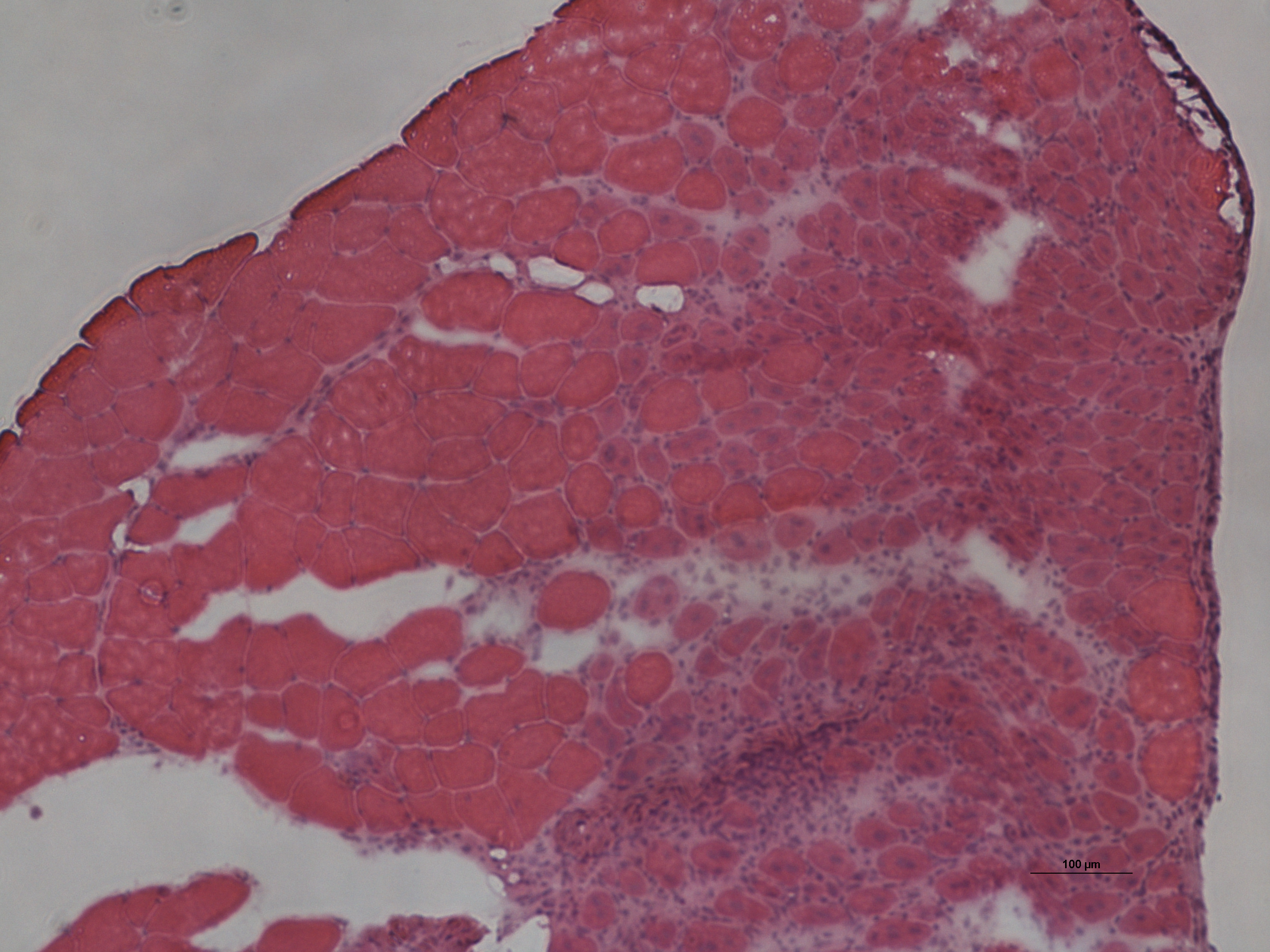

Supplement: Supplementary file 5 — Source data Fig. 2 [file 44319_2024_197_MOESM5_ESM.zip › Figure 2/2F/HE staining 0 and 5 days/Ern1 fl fl 5 days_3.tif]

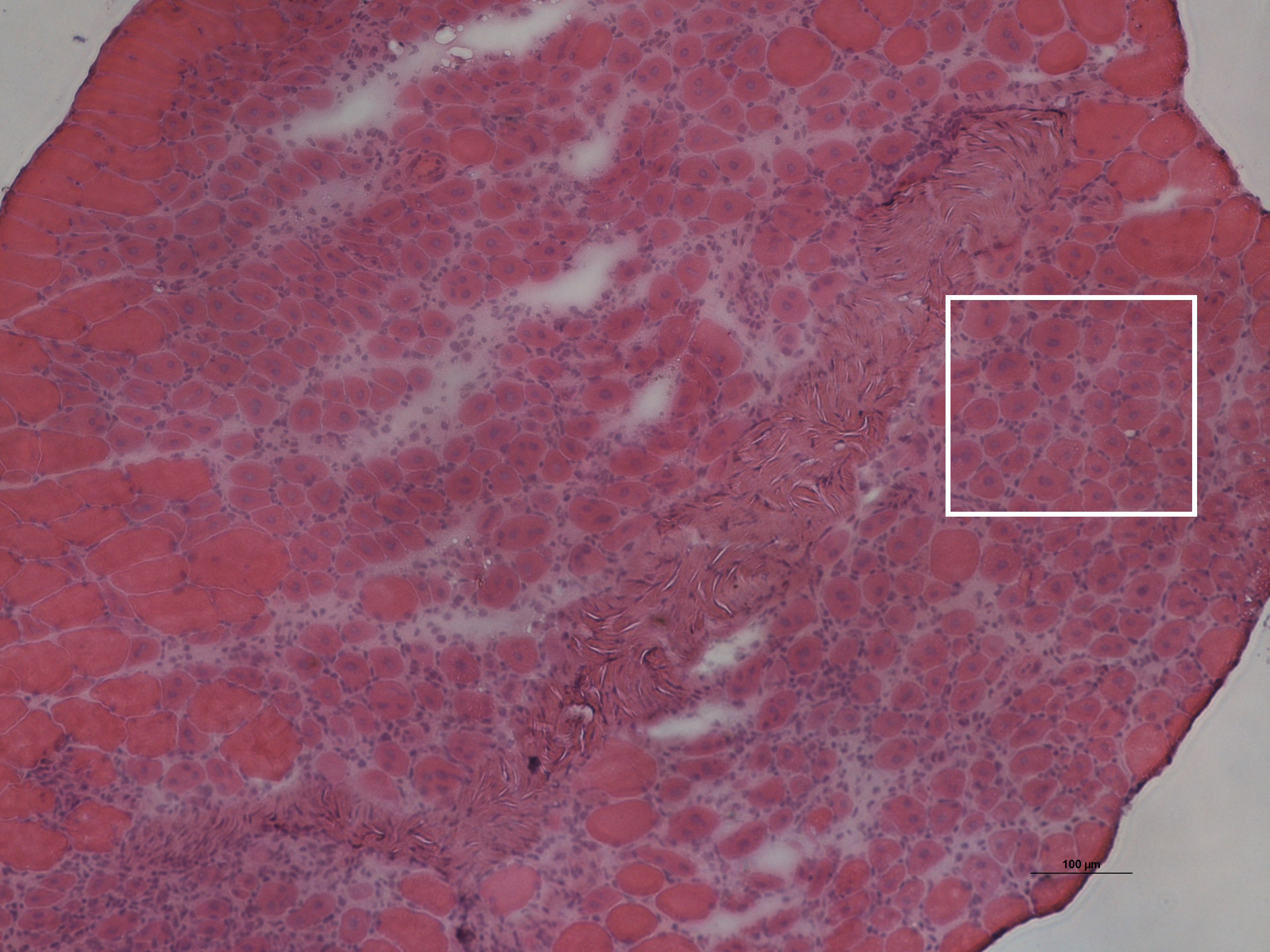

Supplement: Supplementary file 5 — Source data Fig. 2 [file 44319_2024_197_MOESM5_ESM.zip › Figure 2/2F/HE staining 0 and 5 days/Ern1 fl fl 5 days_Representative image with box.tiff]

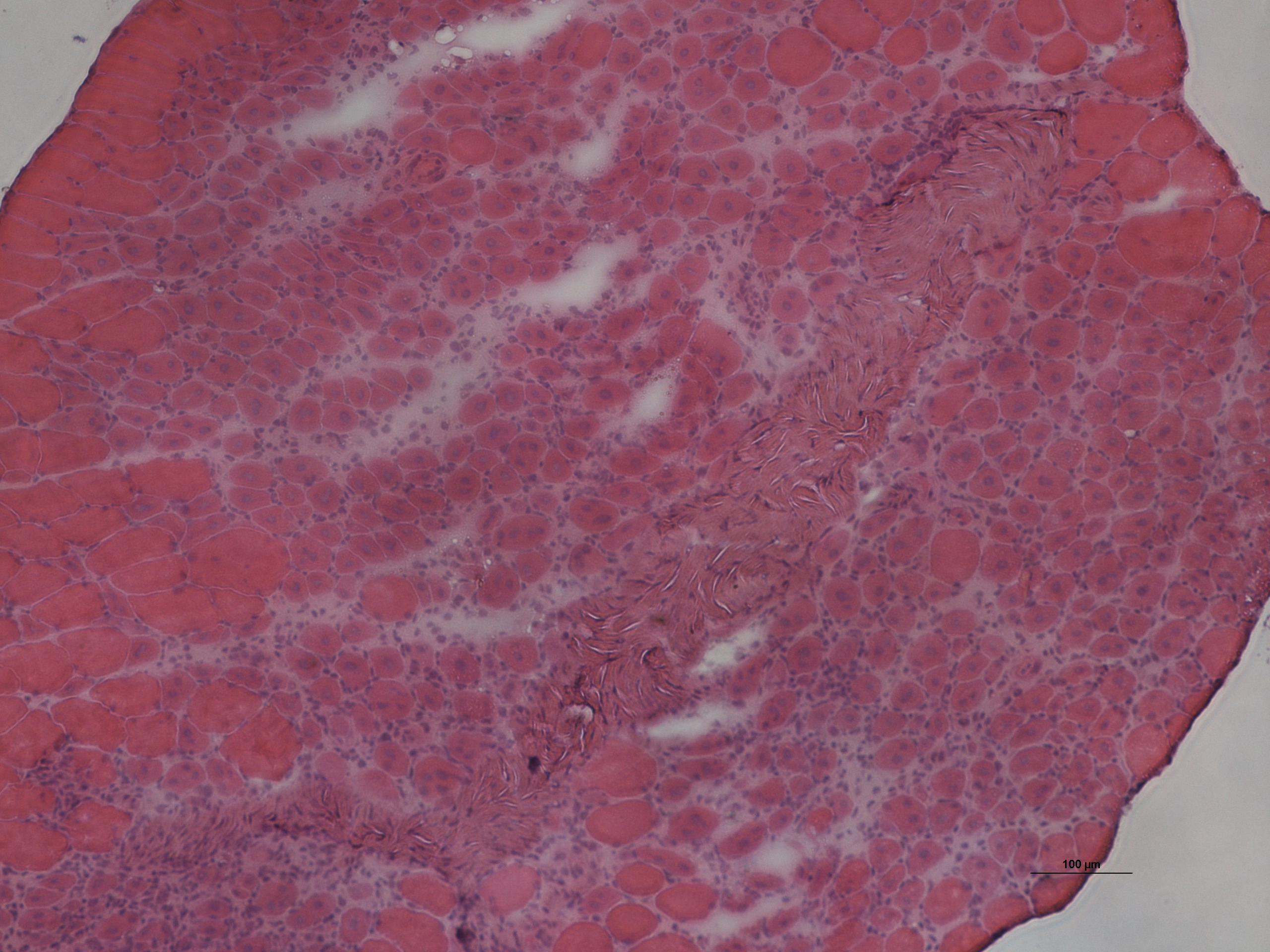

Supplement: Supplementary file 5 — Source data Fig. 2 [file 44319_2024_197_MOESM5_ESM.zip › Figure 2/2F/HE staining 0 and 5 days/Ern1 fl fl 5 days_Representative image.tif]

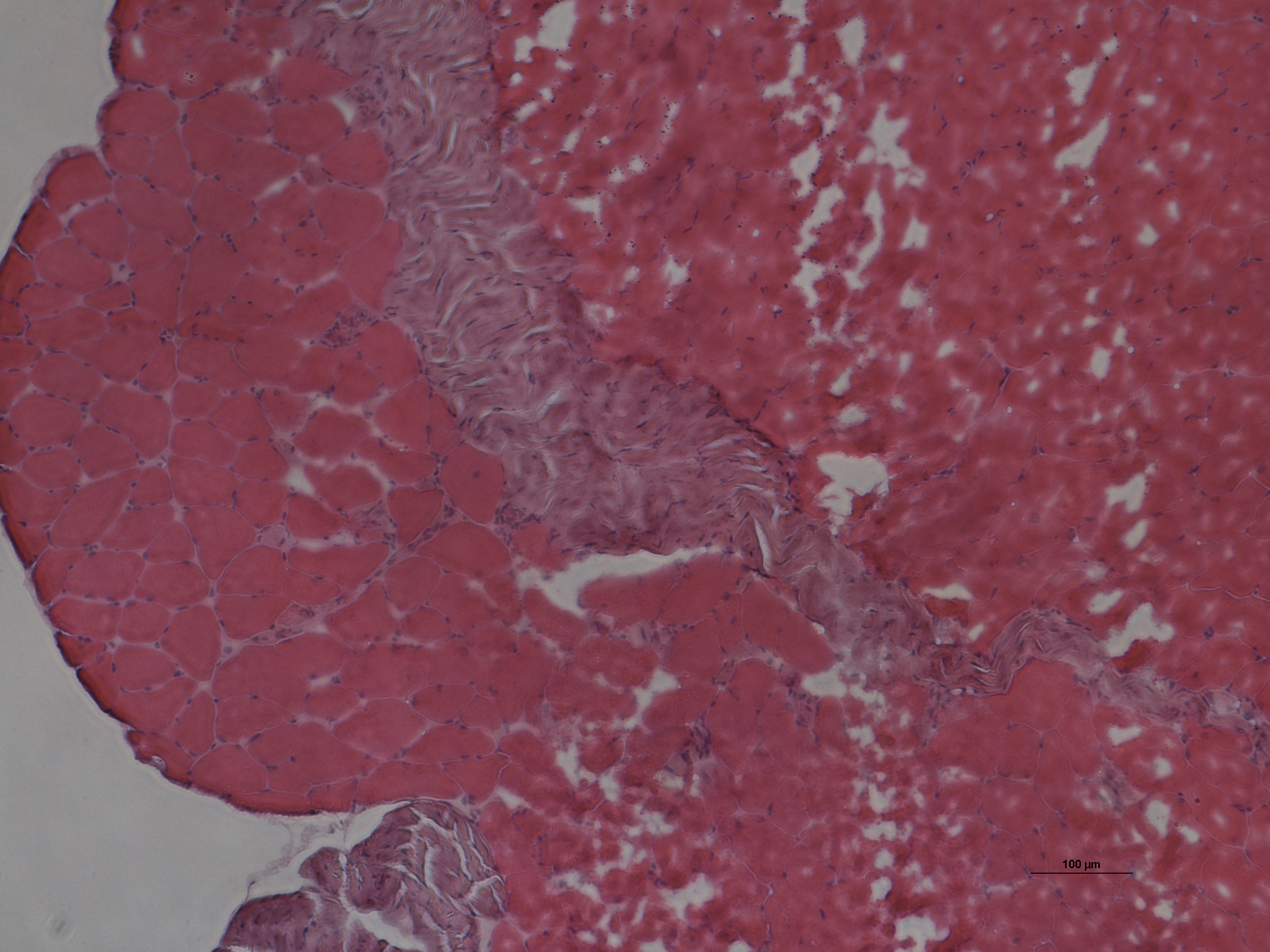

Supplement: Supplementary file 5 — Source data Fig. 2 [file 44319_2024_197_MOESM5_ESM.zip › Figure 2/2F/HE staining 0 and 5 days/Ern1 scKO 0 day_1.tif]

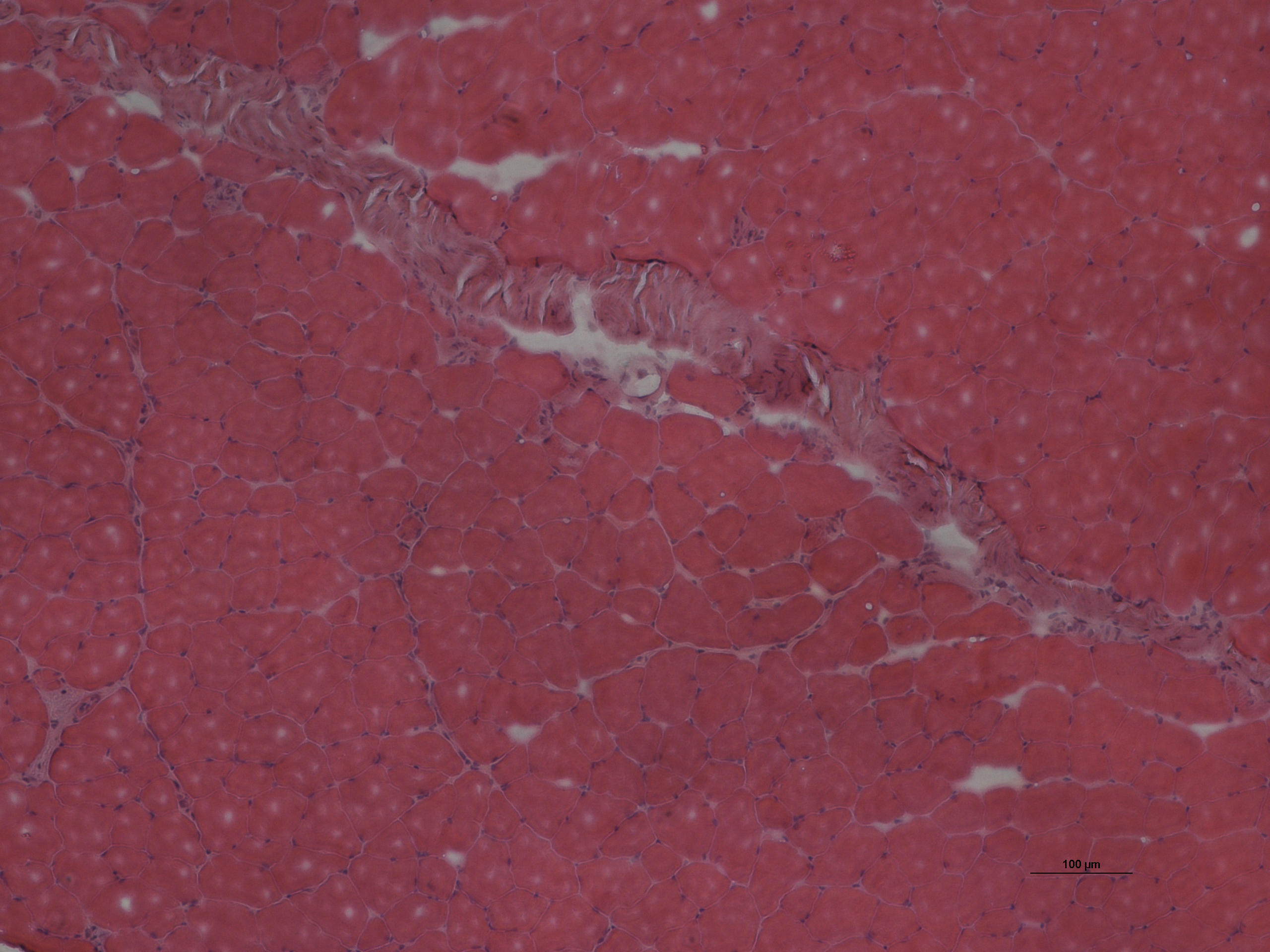

Supplement: Supplementary file 5 — Source data Fig. 2 [file 44319_2024_197_MOESM5_ESM.zip › Figure 2/2F/HE staining 0 and 5 days/Ern1 scKO 0 day_2.tif]

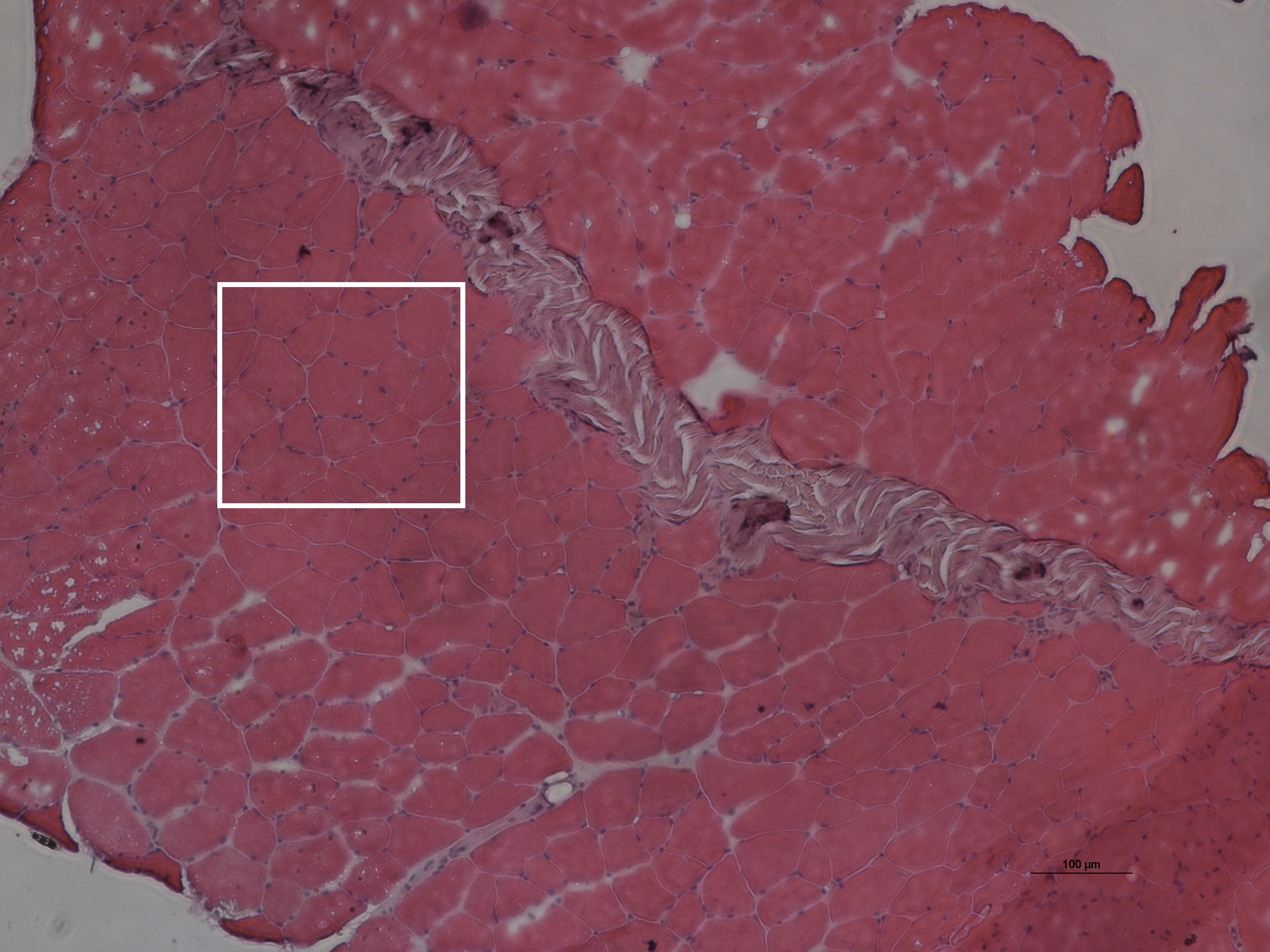

Supplement: Supplementary file 5 — Source data Fig. 2 [file 44319_2024_197_MOESM5_ESM.zip › Figure 2/2F/HE staining 0 and 5 days/Ern1 scKO 0 day_Representative image with box.tiff]

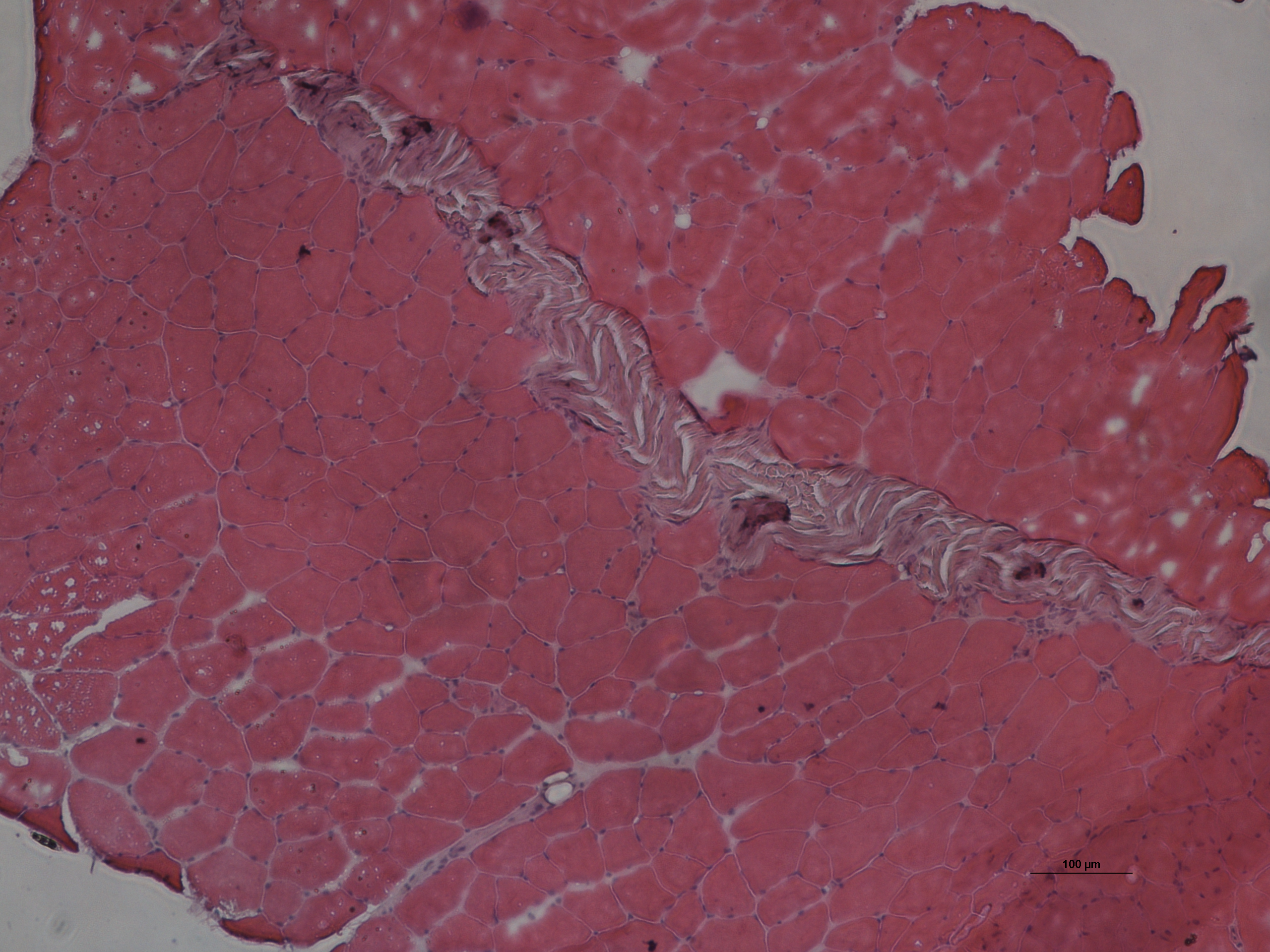

Supplement: Supplementary file 5 — Source data Fig. 2 [file 44319_2024_197_MOESM5_ESM.zip › Figure 2/2F/HE staining 0 and 5 days/Ern1 scKO 0 day_Representative image.tif]

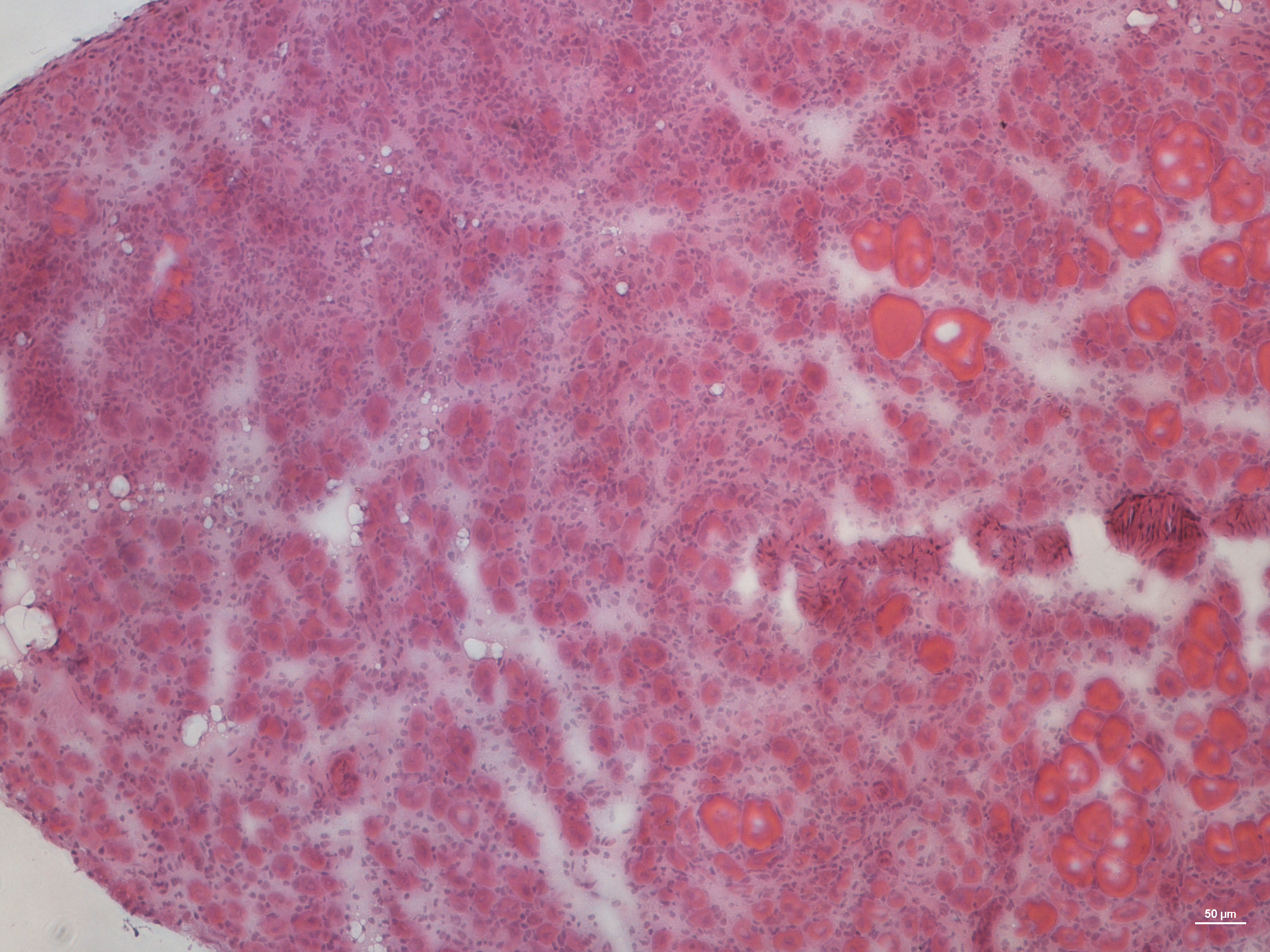

Supplement: Supplementary file 5 — Source data Fig. 2 [file 44319_2024_197_MOESM5_ESM.zip › Figure 2/2F/HE staining 0 and 5 days/Ern1 scKO 5 days_1.tif]

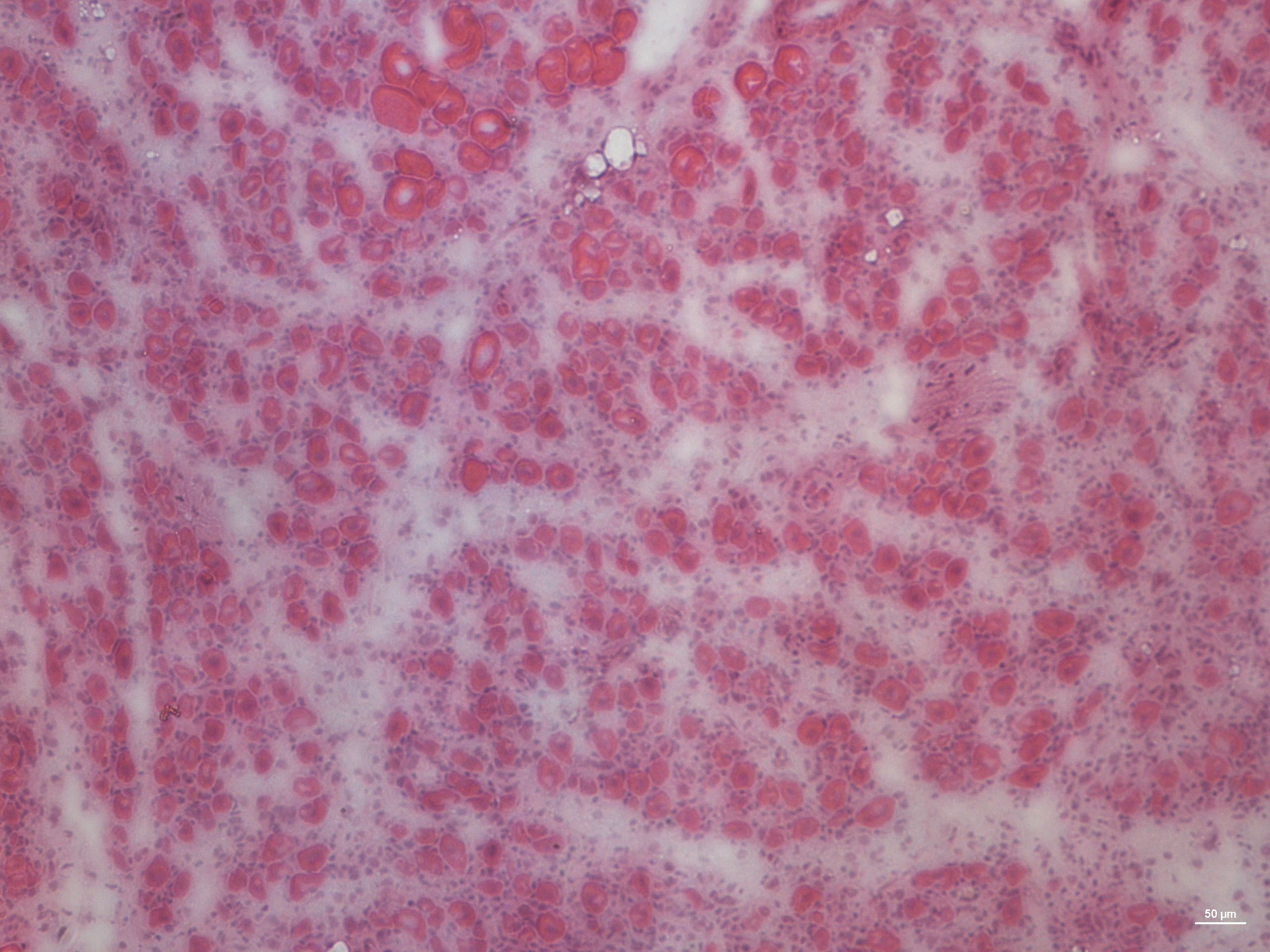

Supplement: Supplementary file 5 — Source data Fig. 2 [file 44319_2024_197_MOESM5_ESM.zip › Figure 2/2F/HE staining 0 and 5 days/Ern1 scKO 5 days_2.tif]

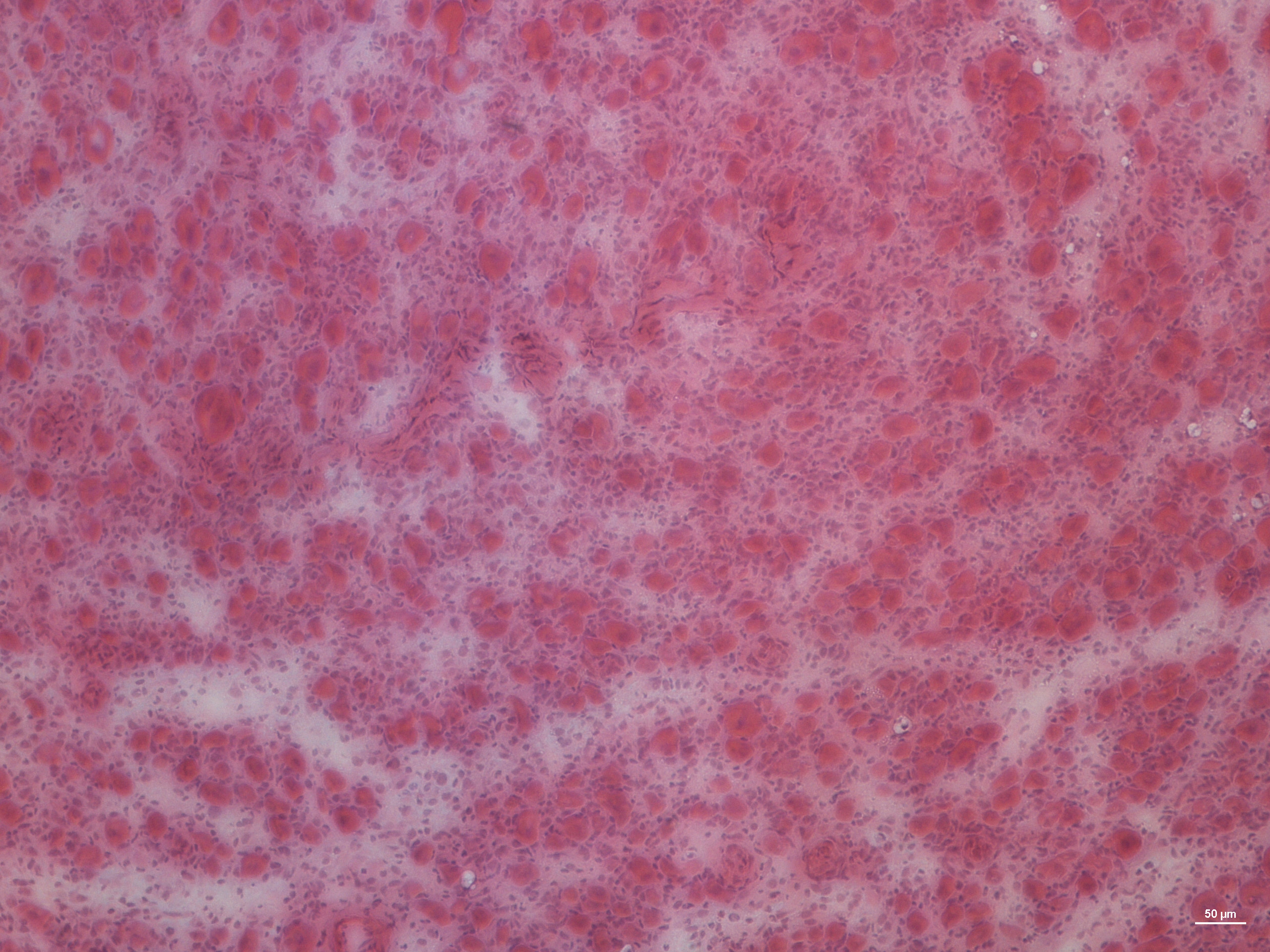

Supplement: Supplementary file 5 — Source data Fig. 2 [file 44319_2024_197_MOESM5_ESM.zip › Figure 2/2F/HE staining 0 and 5 days/Ern1 scKO 5 days_3.tif]

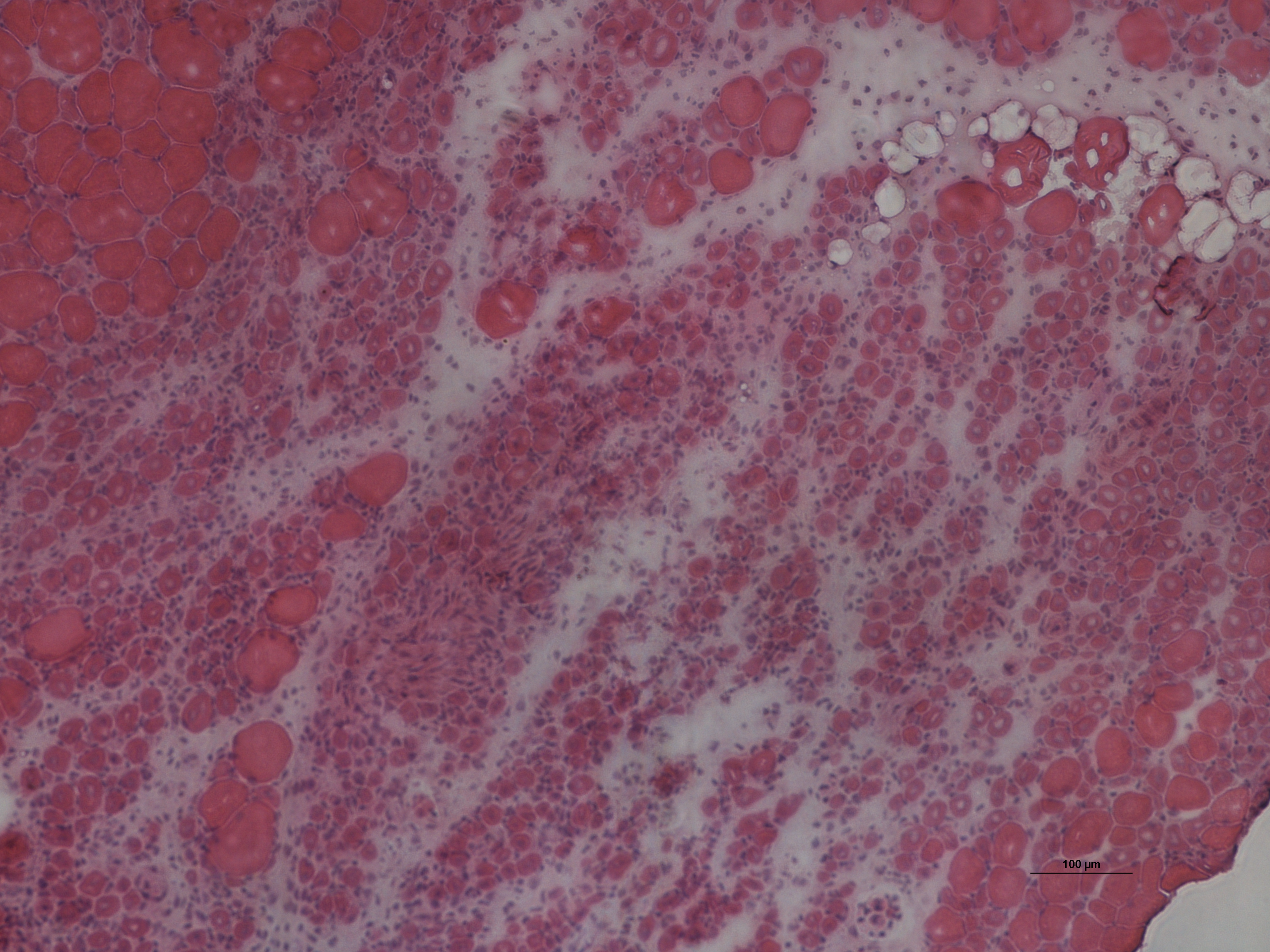

Supplement: Supplementary file 5 — Source data Fig. 2 [file 44319_2024_197_MOESM5_ESM.zip › Figure 2/2F/HE staining 0 and 5 days/Ern1 scKO 5 days_4.tif]

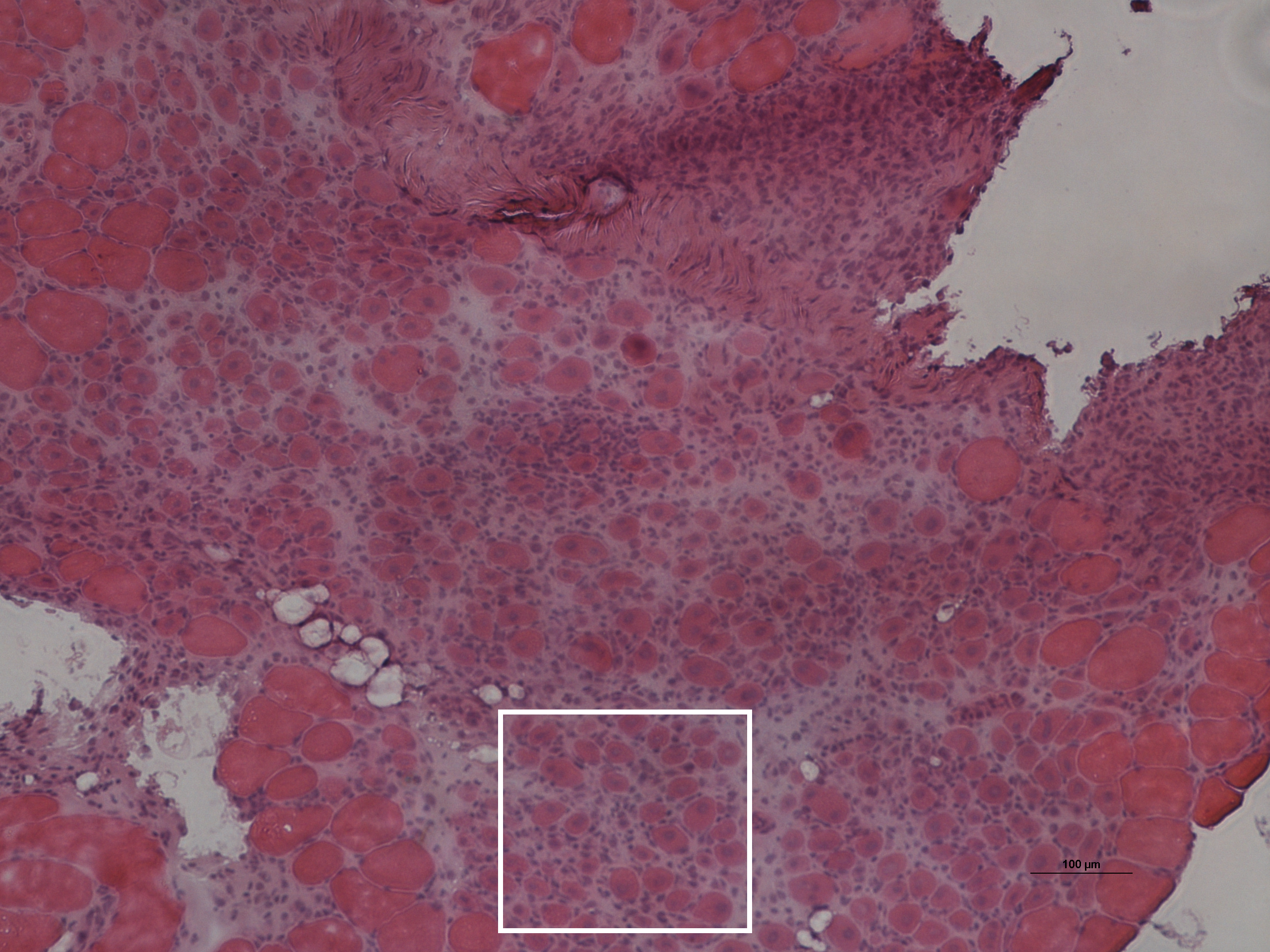

Supplement: Supplementary file 5 — Source data Fig. 2 [file 44319_2024_197_MOESM5_ESM.zip › Figure 2/2F/HE staining 0 and 5 days/Ern1 scKO 5 days_Representative image with box.tiff]

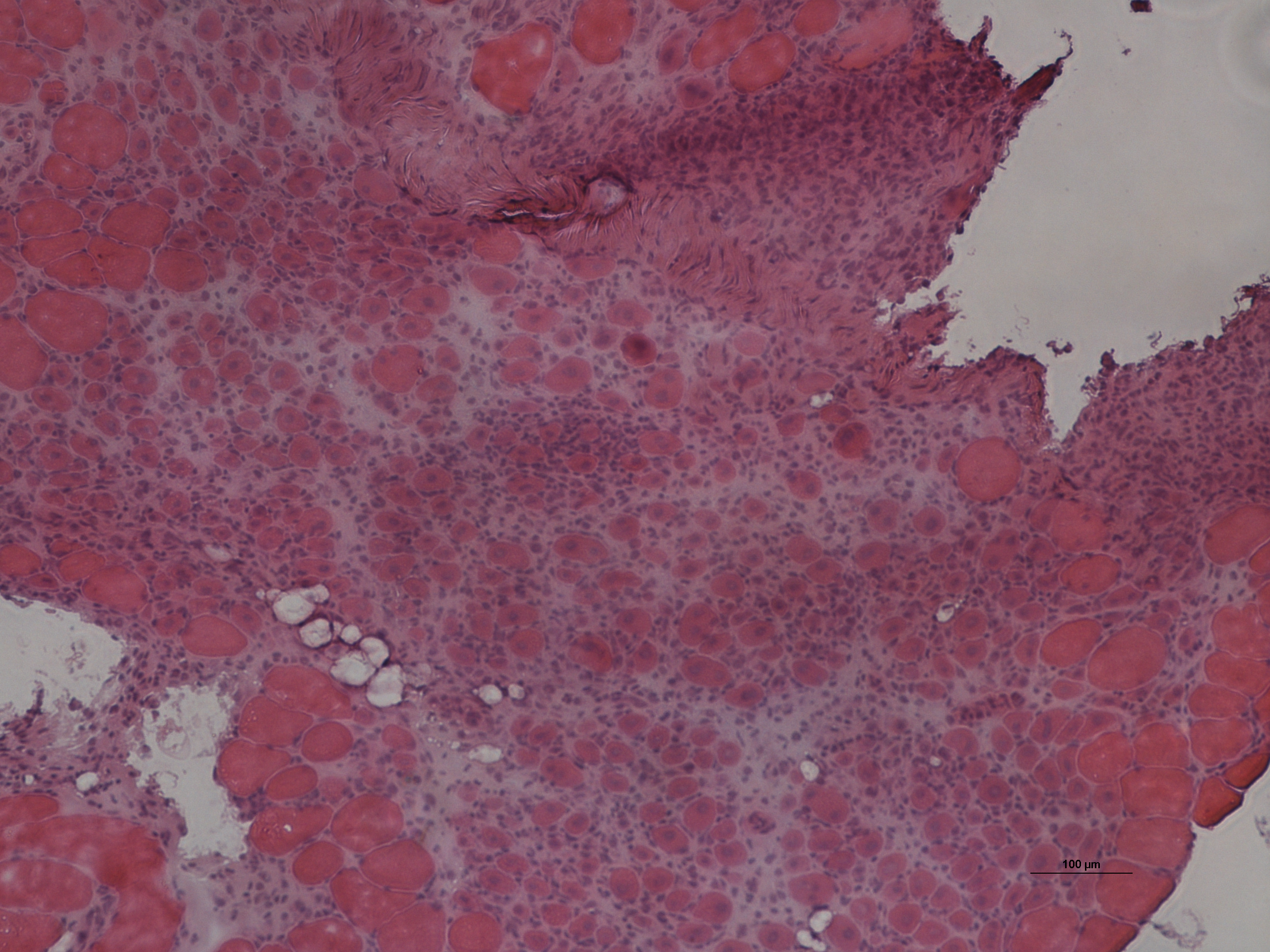

Supplement: Supplementary file 5 — Source data Fig. 2 [file 44319_2024_197_MOESM5_ESM.zip › Figure 2/2F/HE staining 0 and 5 days/Ern1 scKO 5 days_Representative image.tif]

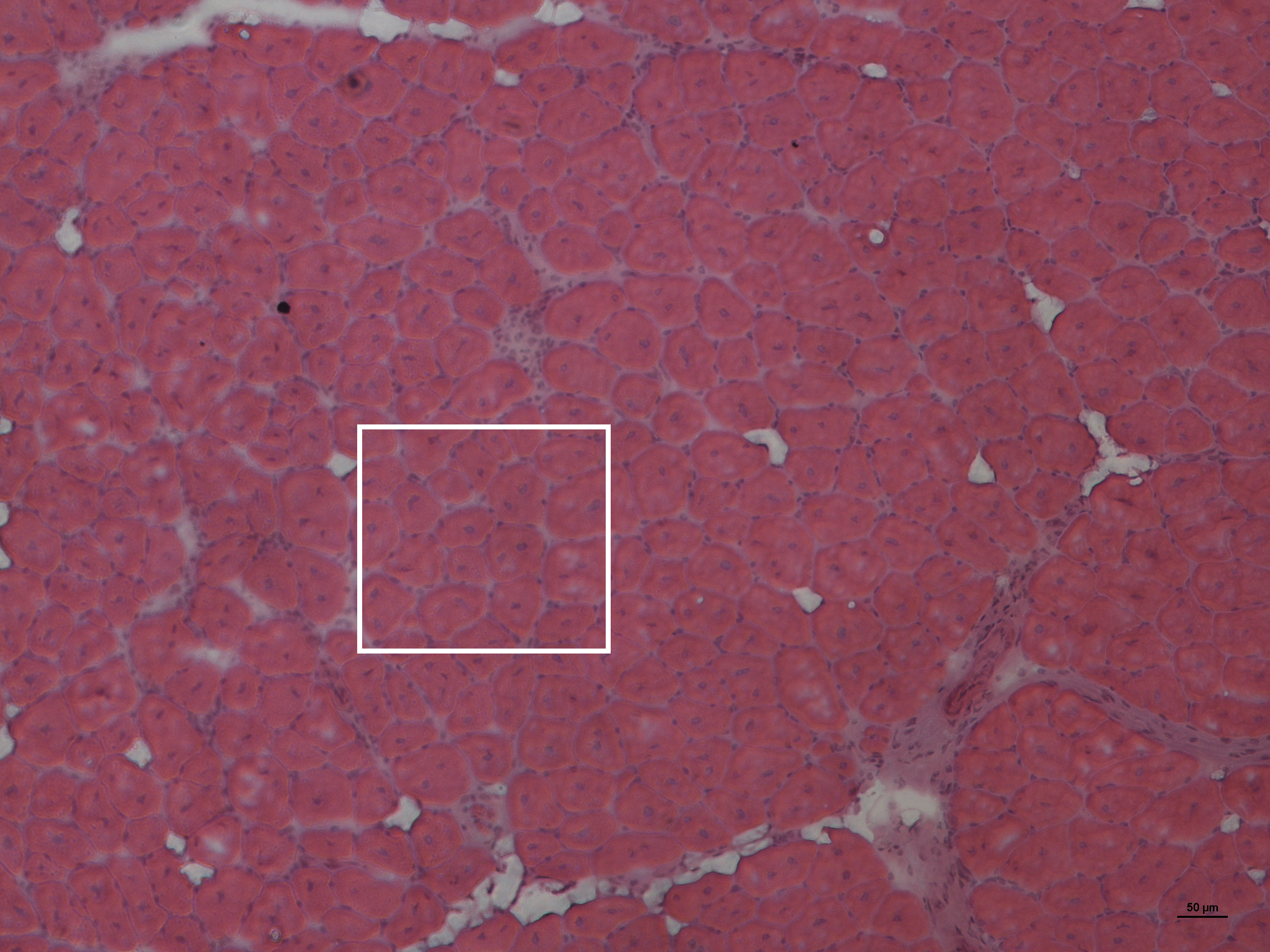

Supplement: Supplementary file 5 — Source data Fig. 2 [file 44319_2024_197_MOESM5_ESM.zip › Figure 2/2F/HE staining 14 days/Ern1 ff 14d-Injured Representative image with box.tiff]

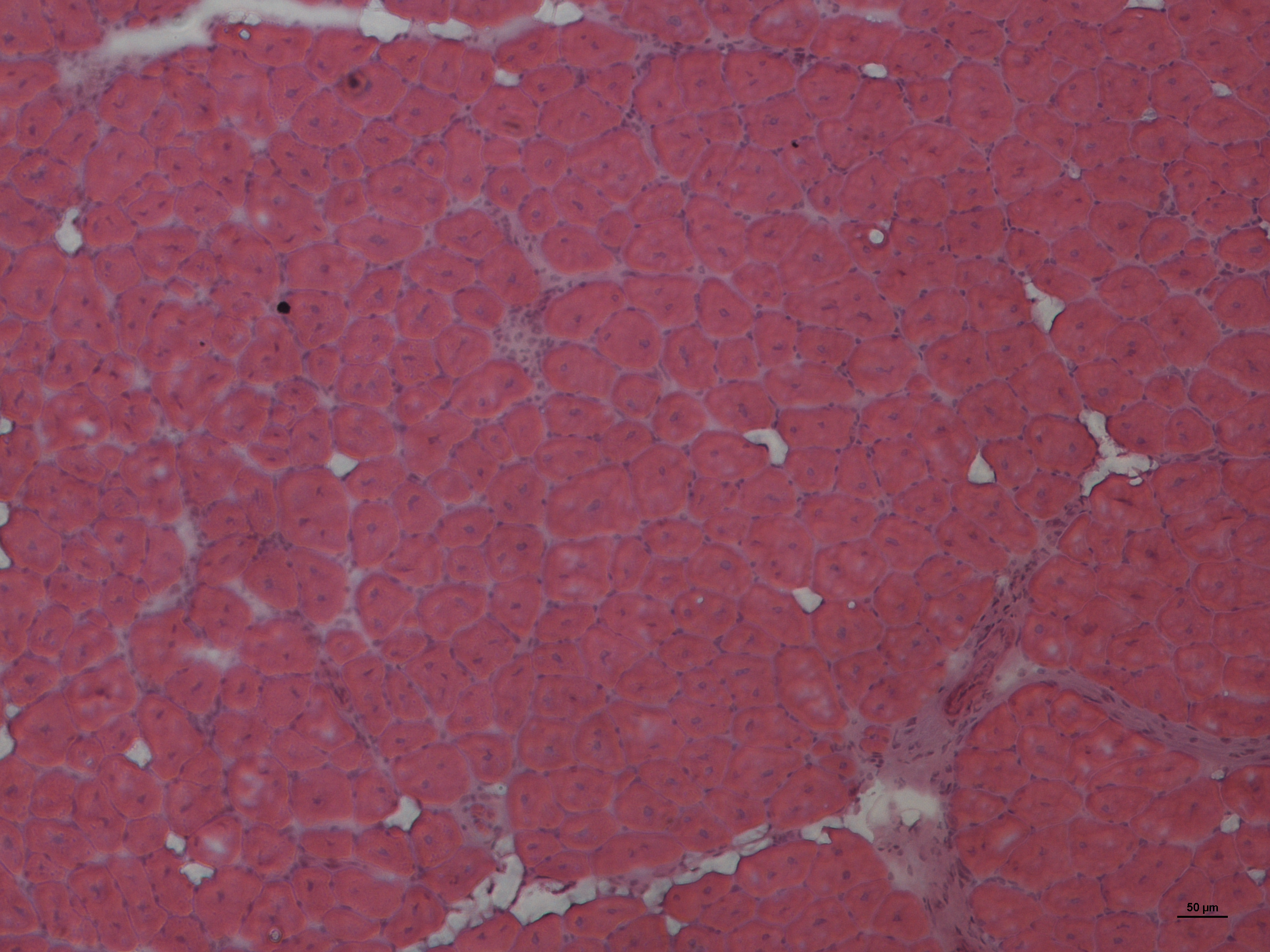

Supplement: Supplementary file 5 — Source data Fig. 2 [file 44319_2024_197_MOESM5_ESM.zip › Figure 2/2F/HE staining 14 days/Ern1 ff 14d-Injured Representative image.tif]

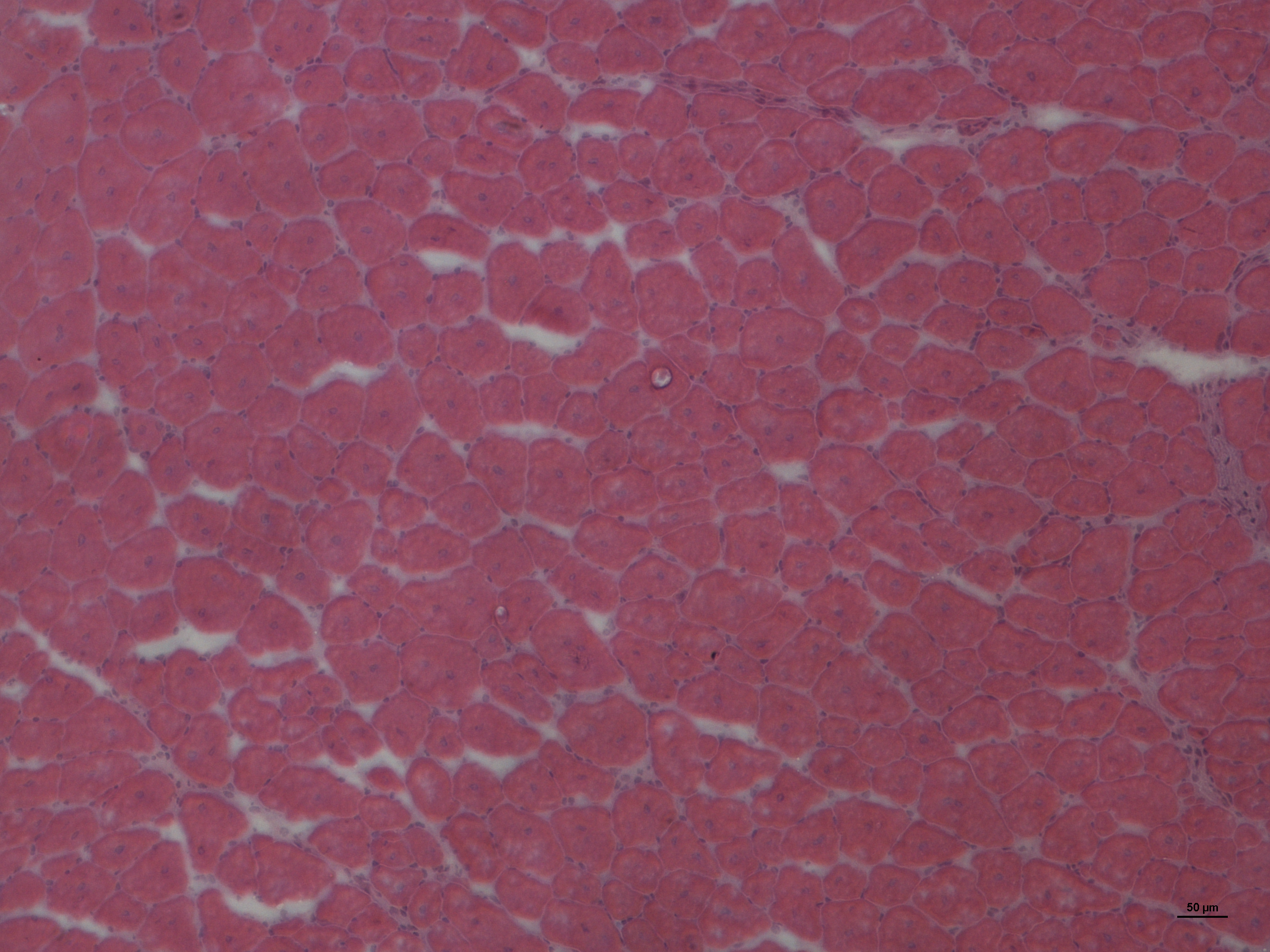

Supplement: Supplementary file 5 — Source data Fig. 2 [file 44319_2024_197_MOESM5_ESM.zip › Figure 2/2F/HE staining 14 days/Ern1 ff 14d-Injured-1.tif]

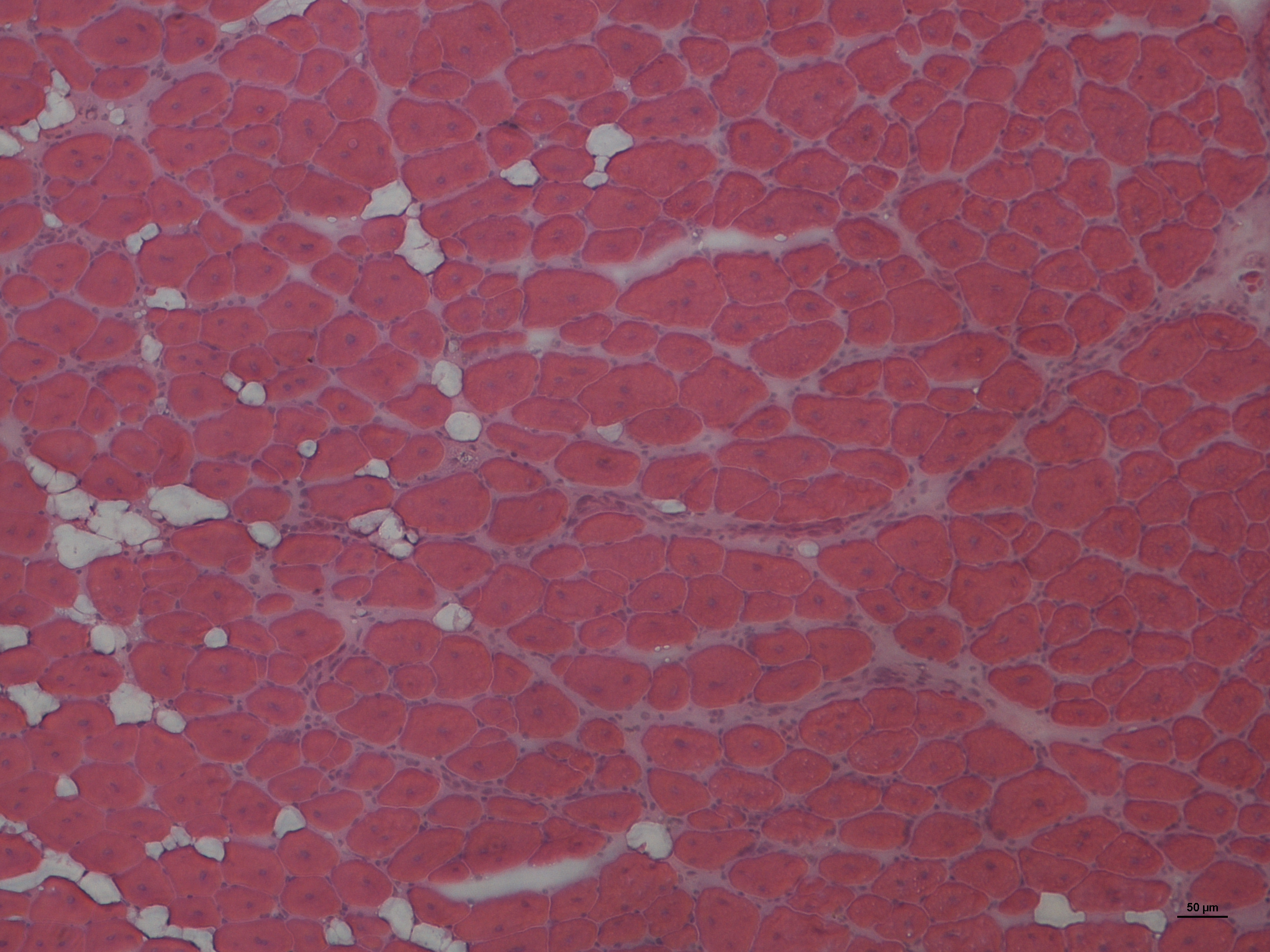

Supplement: Supplementary file 5 — Source data Fig. 2 [file 44319_2024_197_MOESM5_ESM.zip › Figure 2/2F/HE staining 14 days/Ern1 ff 14d-Injured-3.tif]

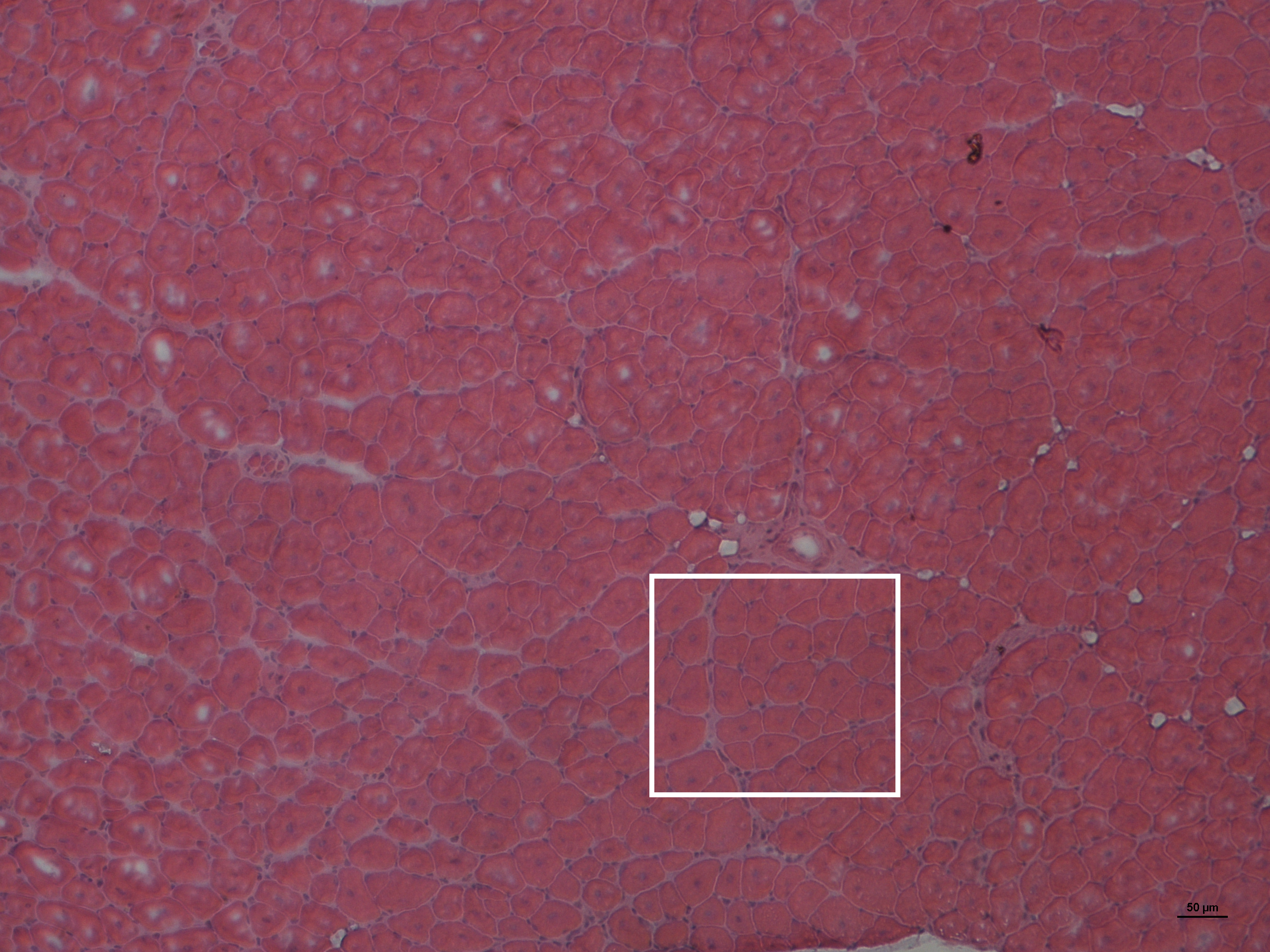

Supplement: Supplementary file 5 — Source data Fig. 2 [file 44319_2024_197_MOESM5_ESM.zip › Figure 2/2F/HE staining 14 days/Ern1scKO 14d-Injured Representative image with box.tiff]

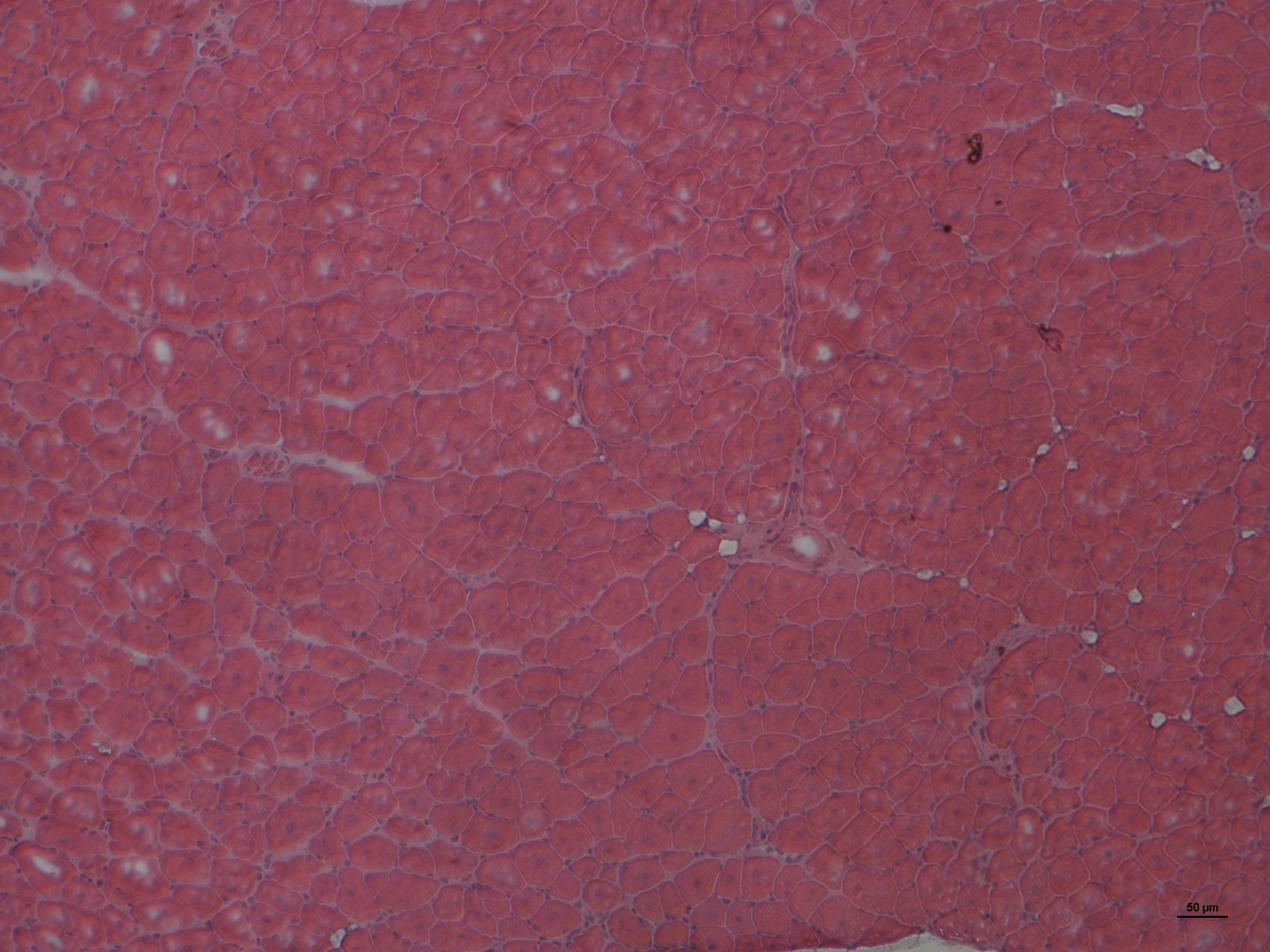

Supplement: Supplementary file 5 — Source data Fig. 2 [file 44319_2024_197_MOESM5_ESM.zip › Figure 2/2F/HE staining 14 days/Ern1scKO 14d-Injured Representative image.tif]

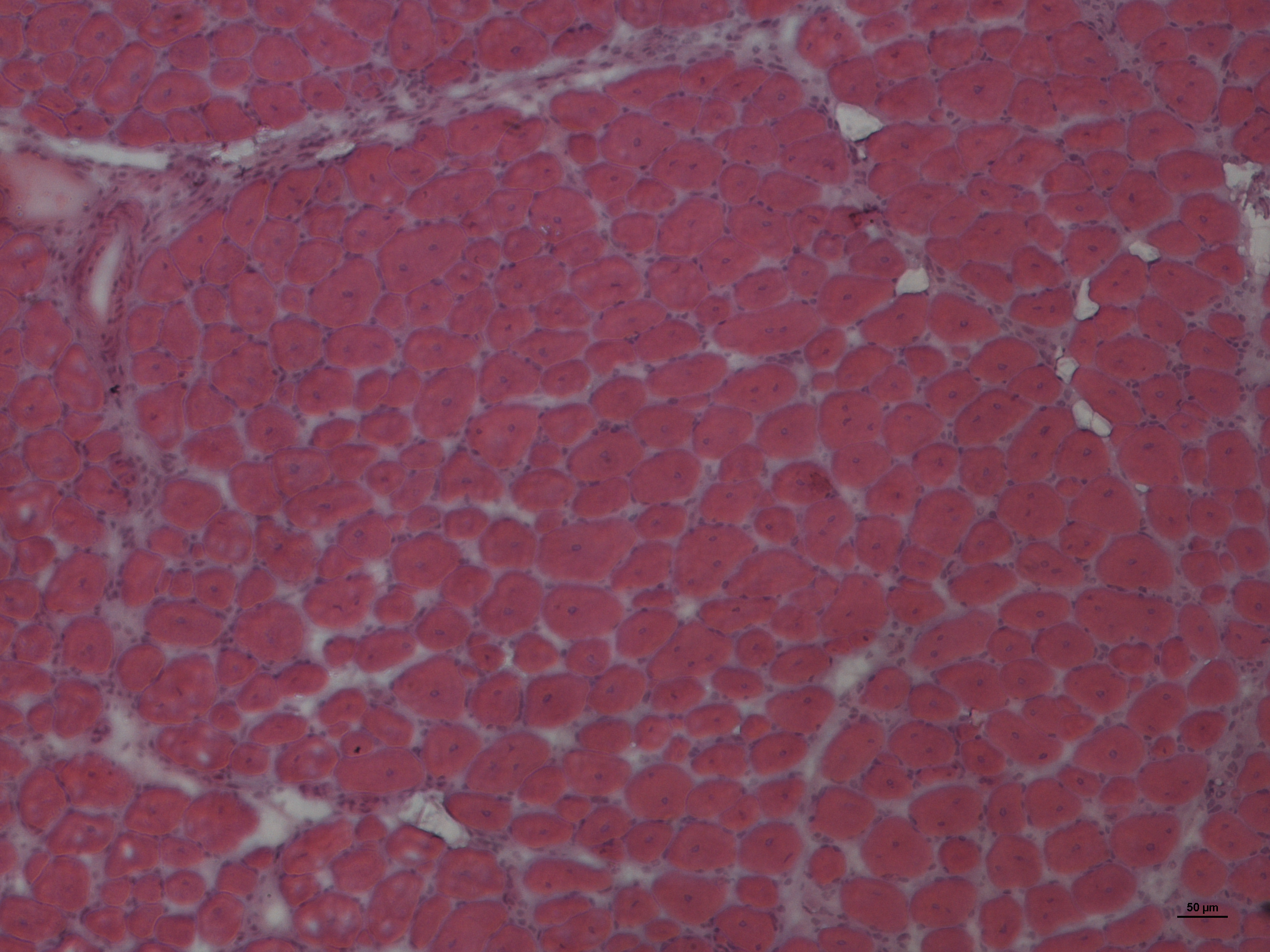

Supplement: Supplementary file 5 — Source data Fig. 2 [file 44319_2024_197_MOESM5_ESM.zip › Figure 2/2F/HE staining 14 days/Ern1scKO 14d-Injured-1.tif]

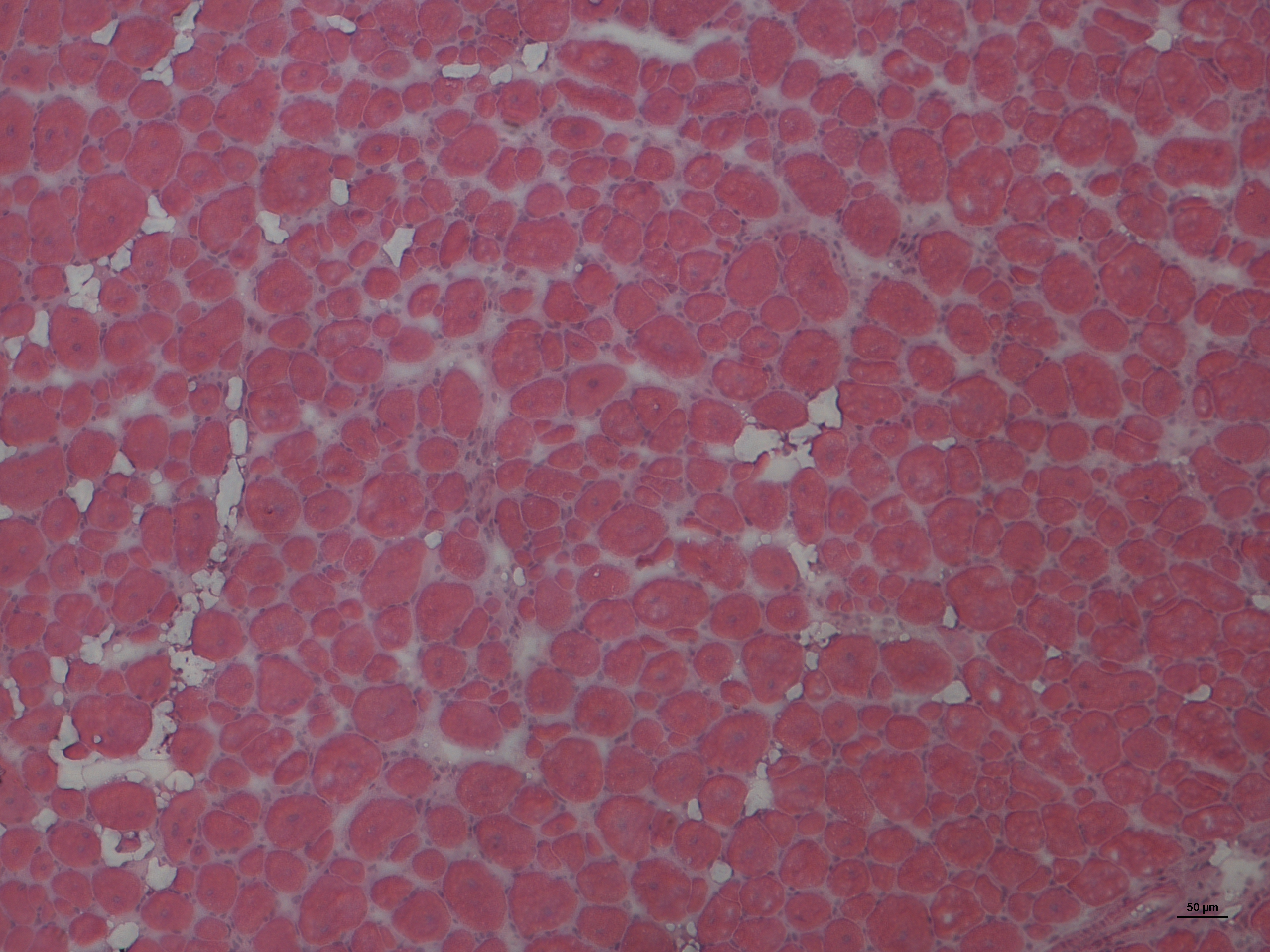

Supplement: Supplementary file 5 — Source data Fig. 2 [file 44319_2024_197_MOESM5_ESM.zip › Figure 2/2F/HE staining 14 days/Ern1scKO 14d-Injured-2.tif]

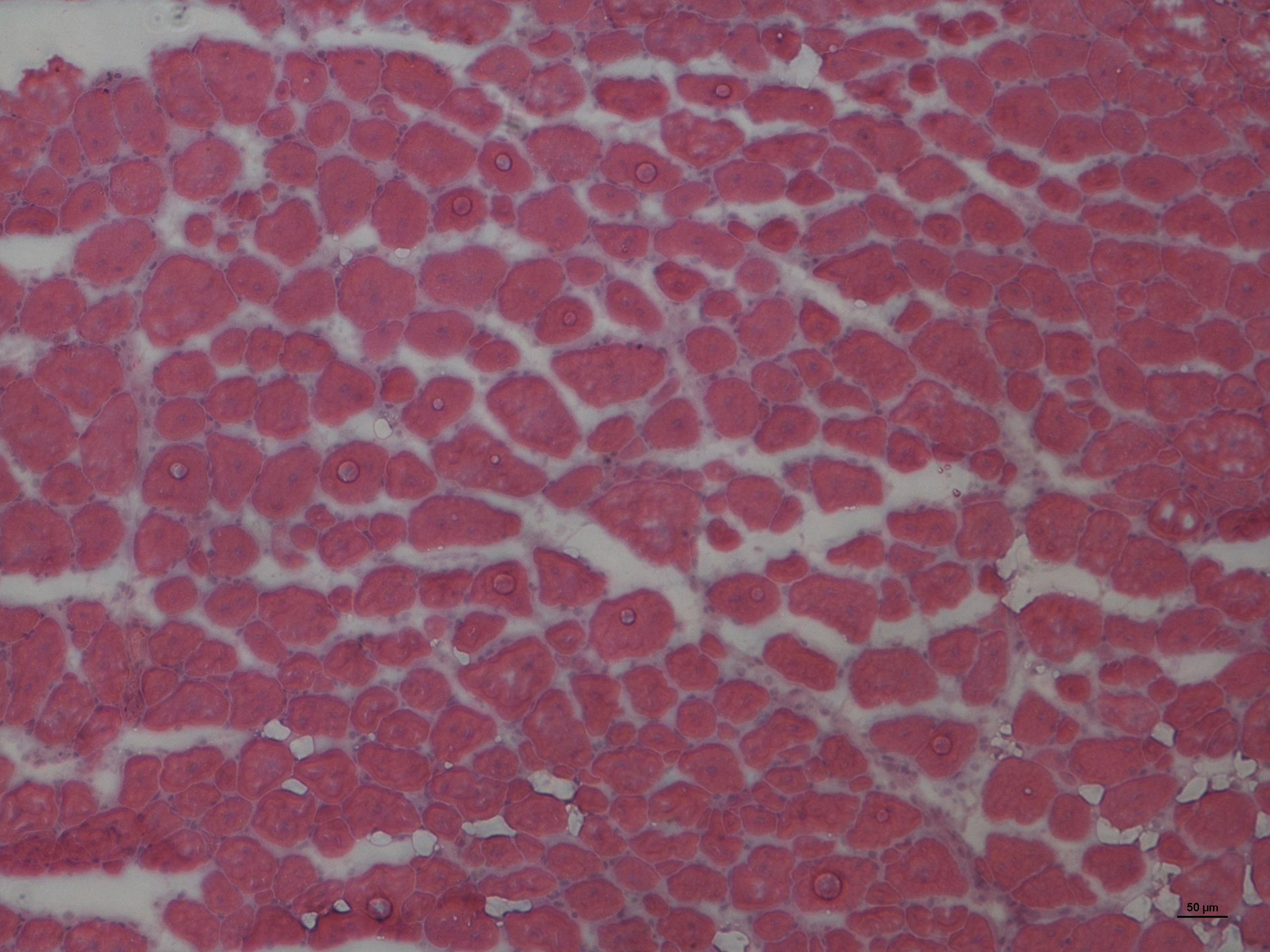

Supplement: Supplementary file 5 — Source data Fig. 2 [file 44319_2024_197_MOESM5_ESM.zip › Figure 2/2F/HE staining 14 days/Ern1scKO 14d-Injured-3.tif]

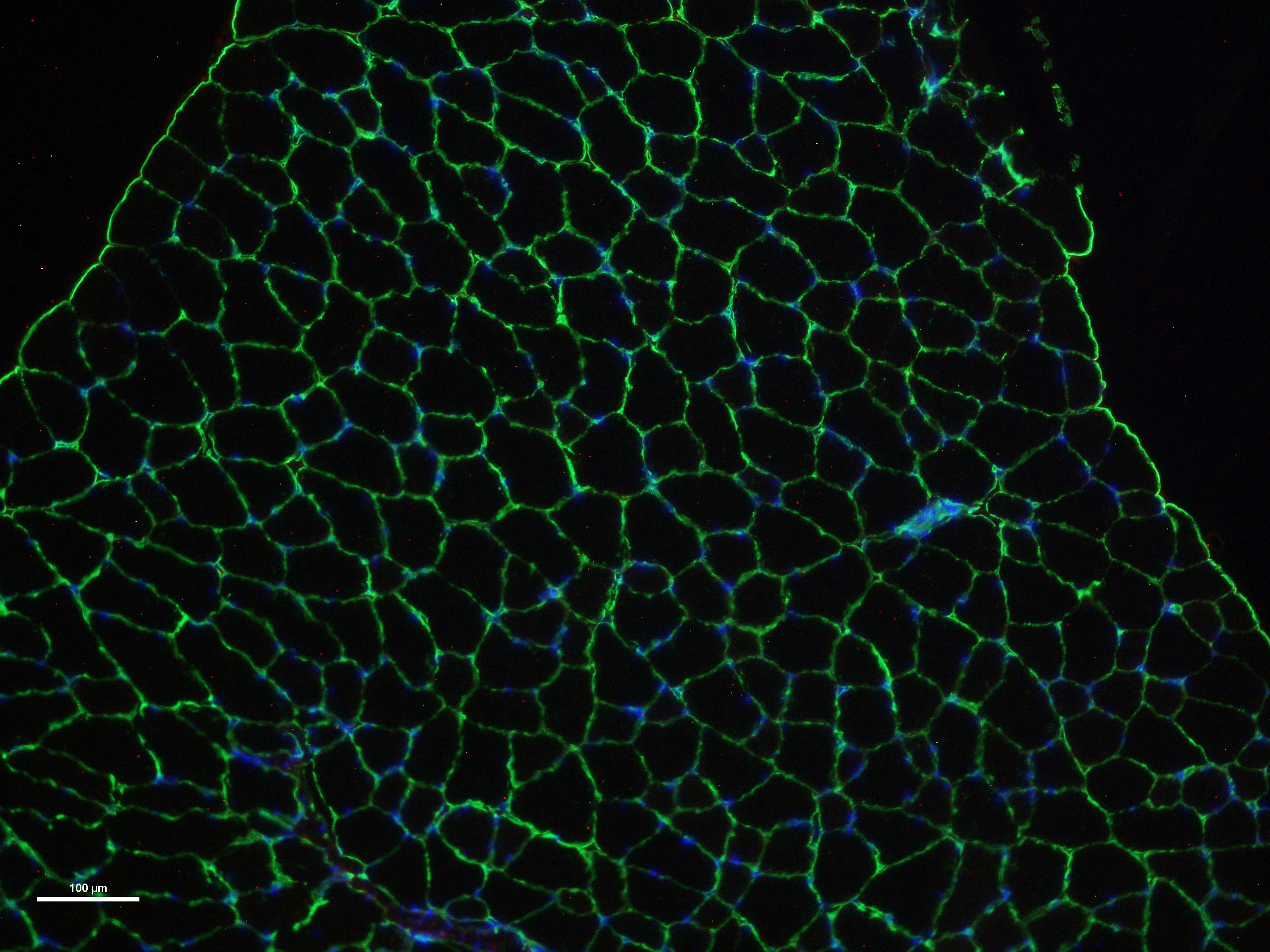

Supplement: Supplementary file 6 — Source data Fig. 3 [file 44319_2024_197_MOESM6_ESM.zip › Figure 3/3A/eMyHC-Laminin staining images/Ern1 fl fl Uninjured_1.tif]

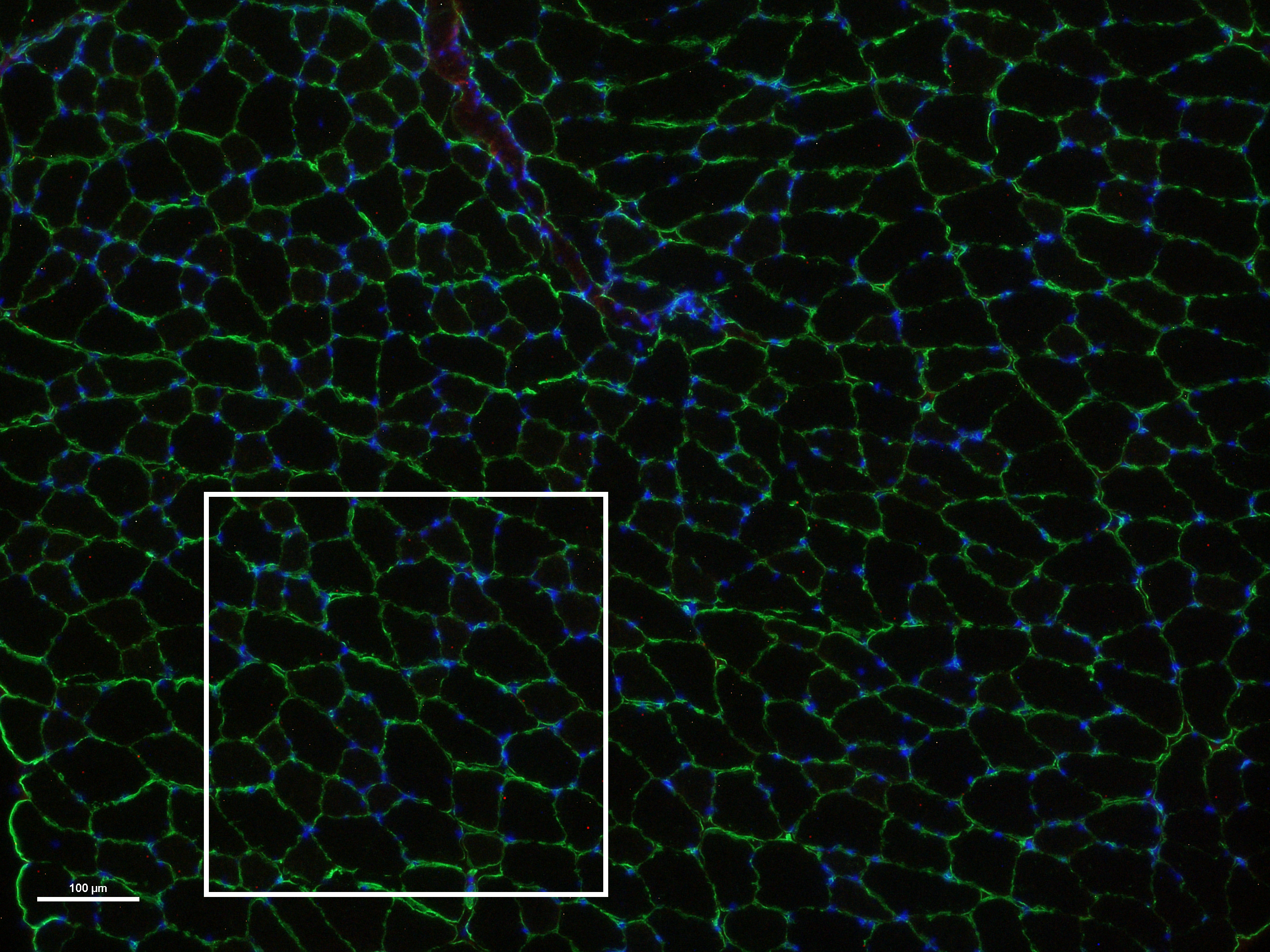

Supplement: Supplementary file 6 — Source data Fig. 3 [file 44319_2024_197_MOESM6_ESM.zip › Figure 3/3A/eMyHC-Laminin staining images/Ern1 fl fl Uninjured_Representative image with box.tiff]

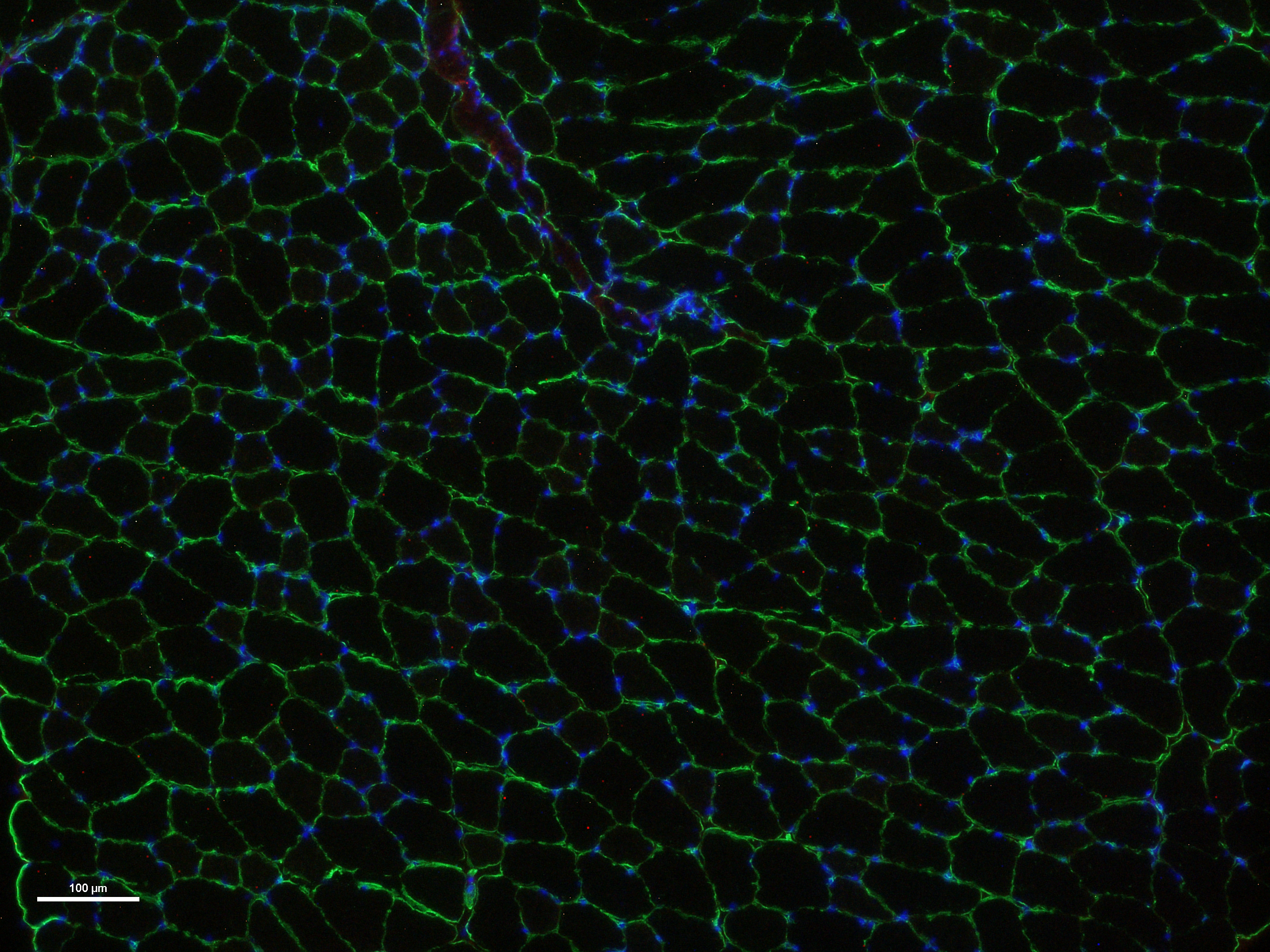

Supplement: Supplementary file 6 — Source data Fig. 3 [file 44319_2024_197_MOESM6_ESM.zip › Figure 3/3A/eMyHC-Laminin staining images/Ern1 fl fl Uninjured_Representative image.tif]

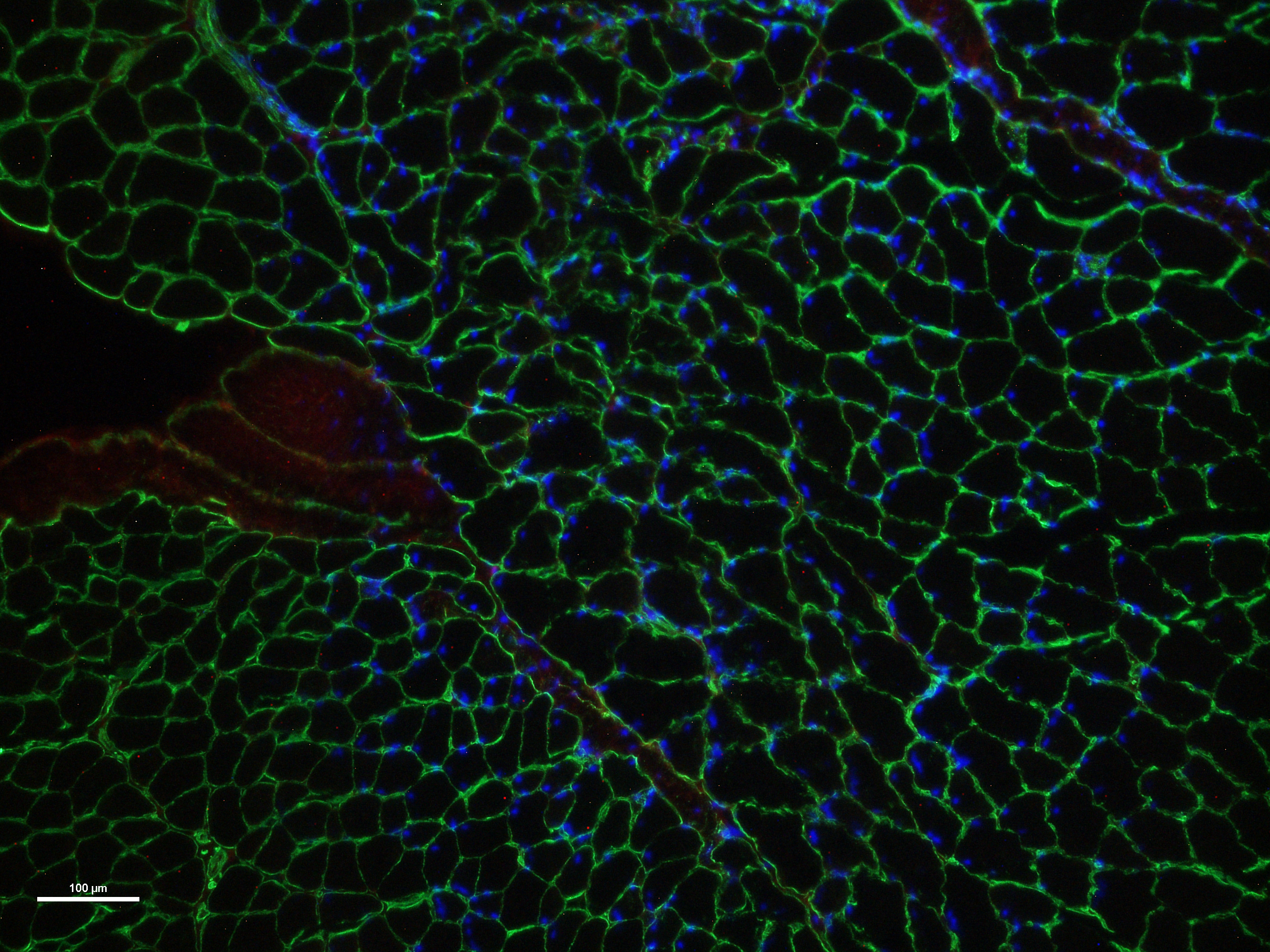

Supplement: Supplementary file 6 — Source data Fig. 3 [file 44319_2024_197_MOESM6_ESM.zip › Figure 3/3A/eMyHC-Laminin staining images/Ern1 scKO Uninjured_1.tif]

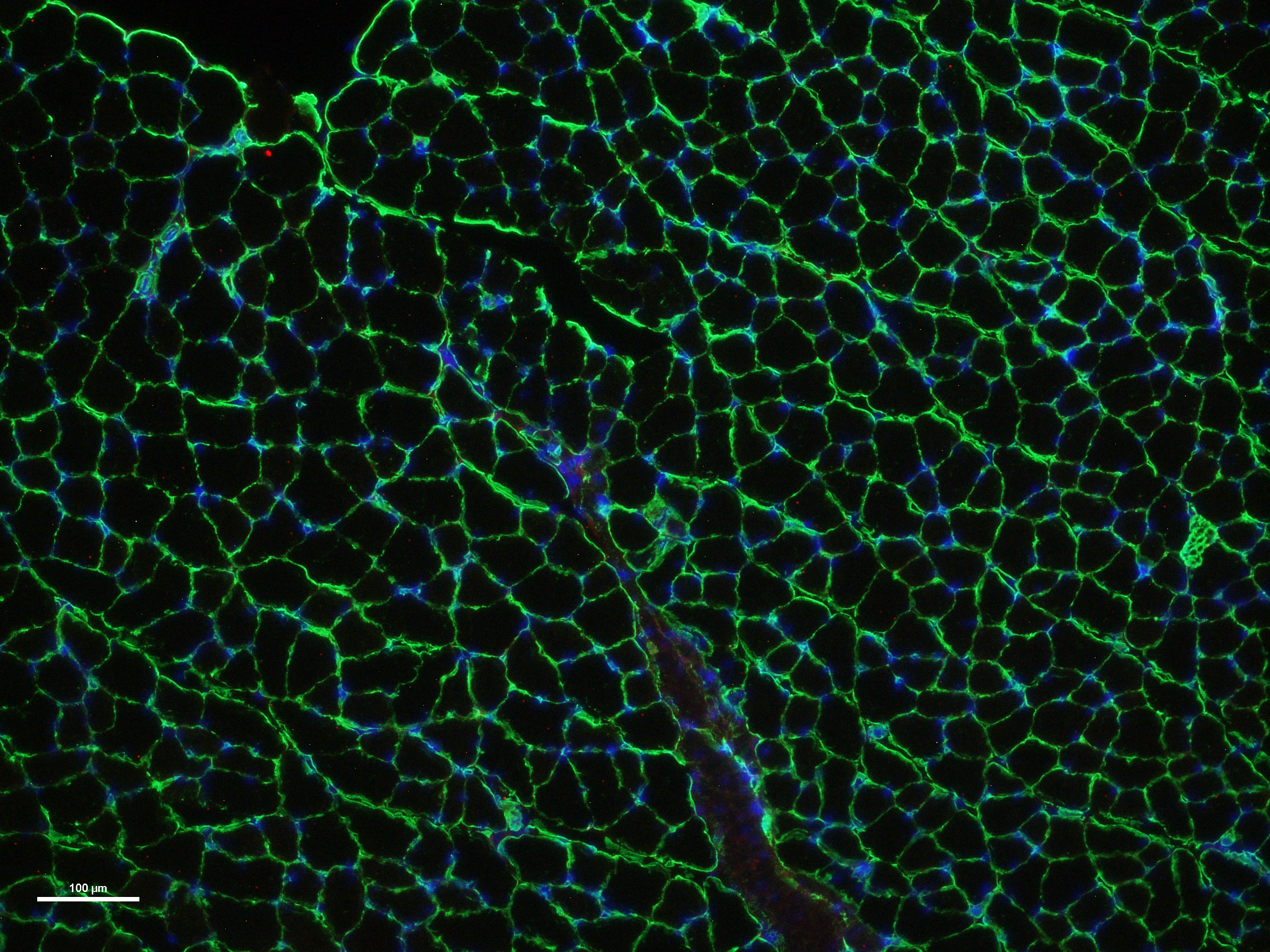

Supplement: Supplementary file 6 — Source data Fig. 3 [file 44319_2024_197_MOESM6_ESM.zip › Figure 3/3A/eMyHC-Laminin staining images/Ern1 scKO Uninjured_2.tif]

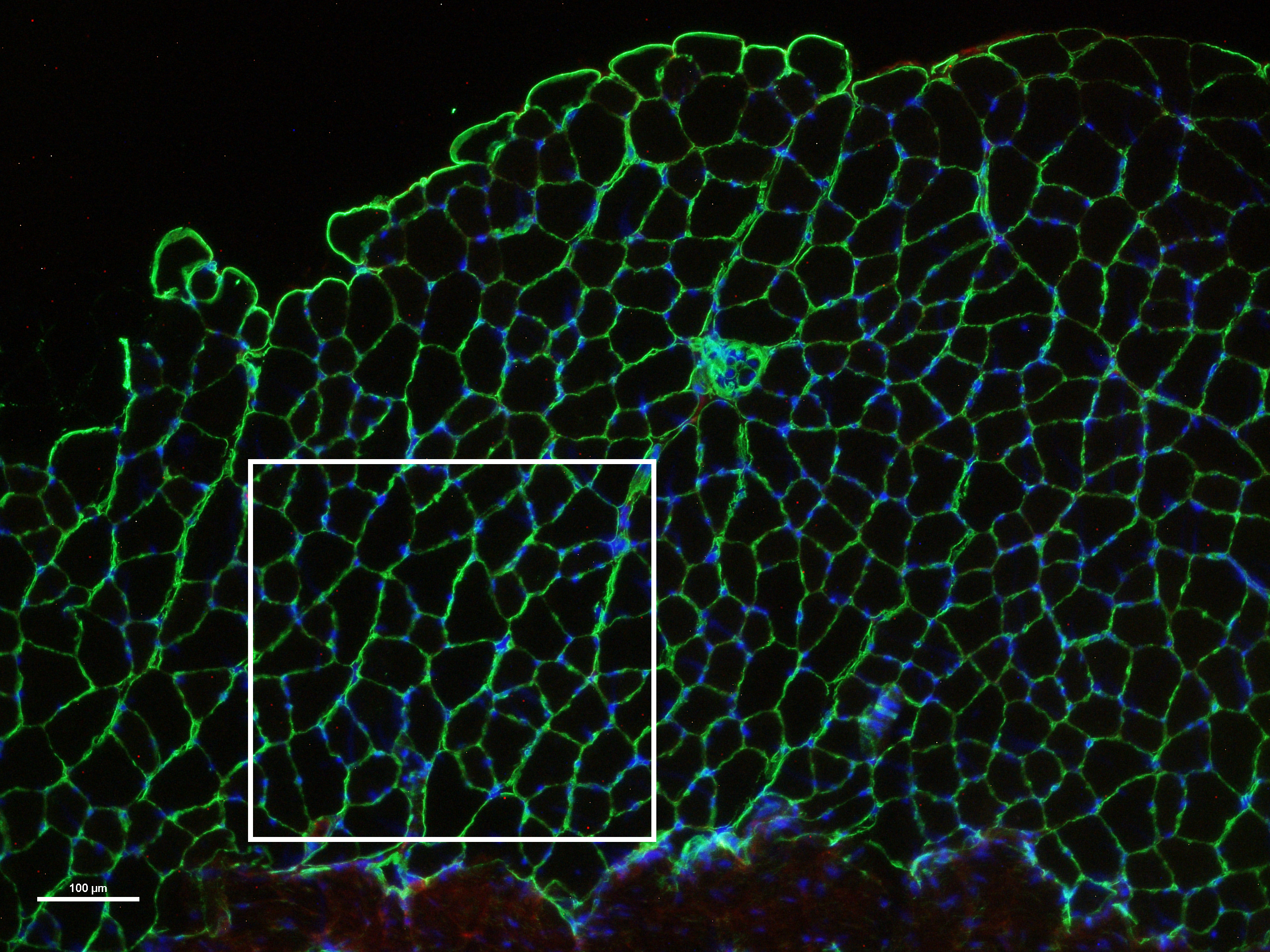

Supplement: Supplementary file 6 — Source data Fig. 3 [file 44319_2024_197_MOESM6_ESM.zip › Figure 3/3A/eMyHC-Laminin staining images/Ern1 scKO Uninjured_Representative image with box.tiff]

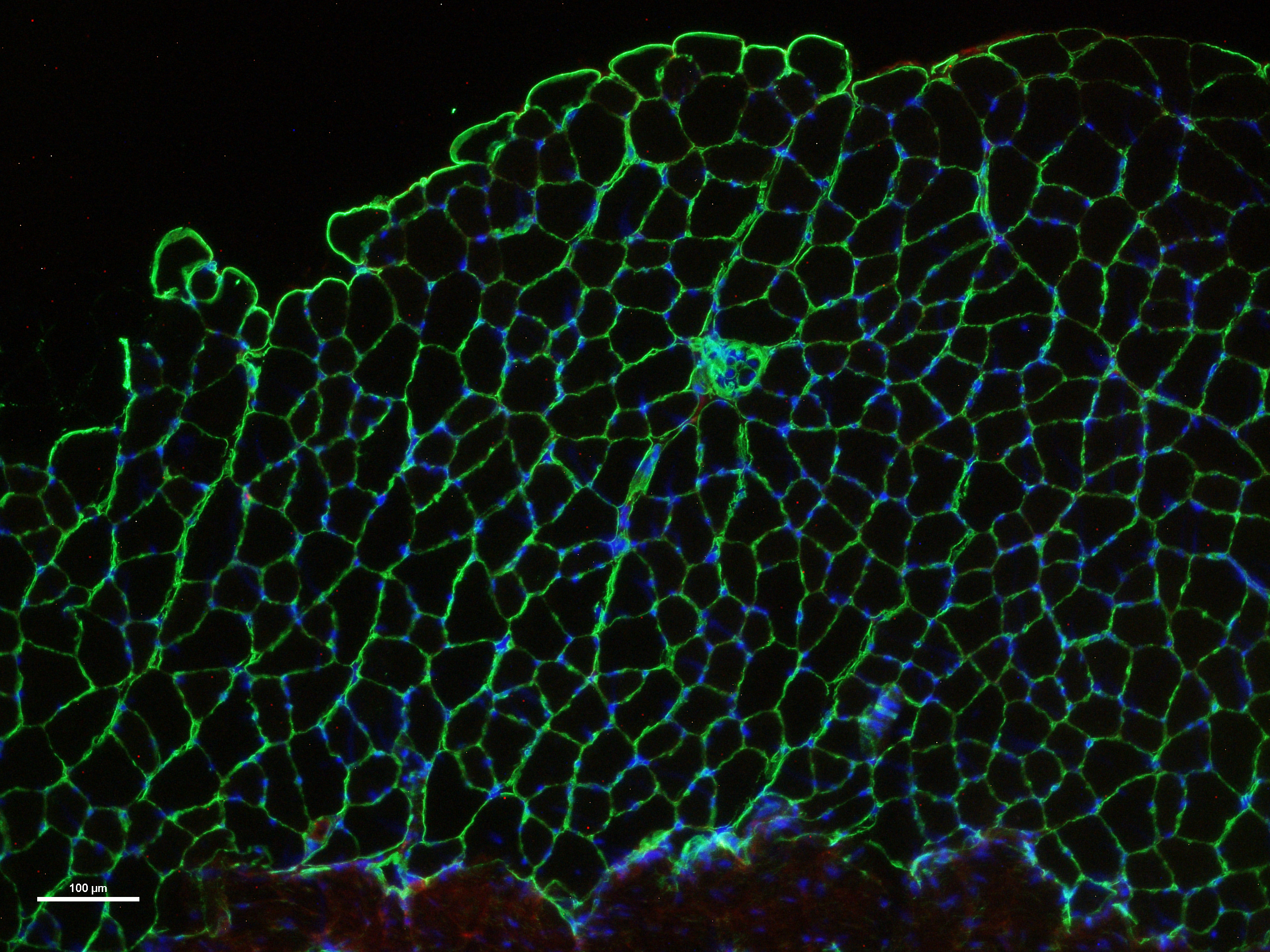

Supplement: Supplementary file 6 — Source data Fig. 3 [file 44319_2024_197_MOESM6_ESM.zip › Figure 3/3A/eMyHC-Laminin staining images/Ern1 scKO Uninjured_Representative image.tif]

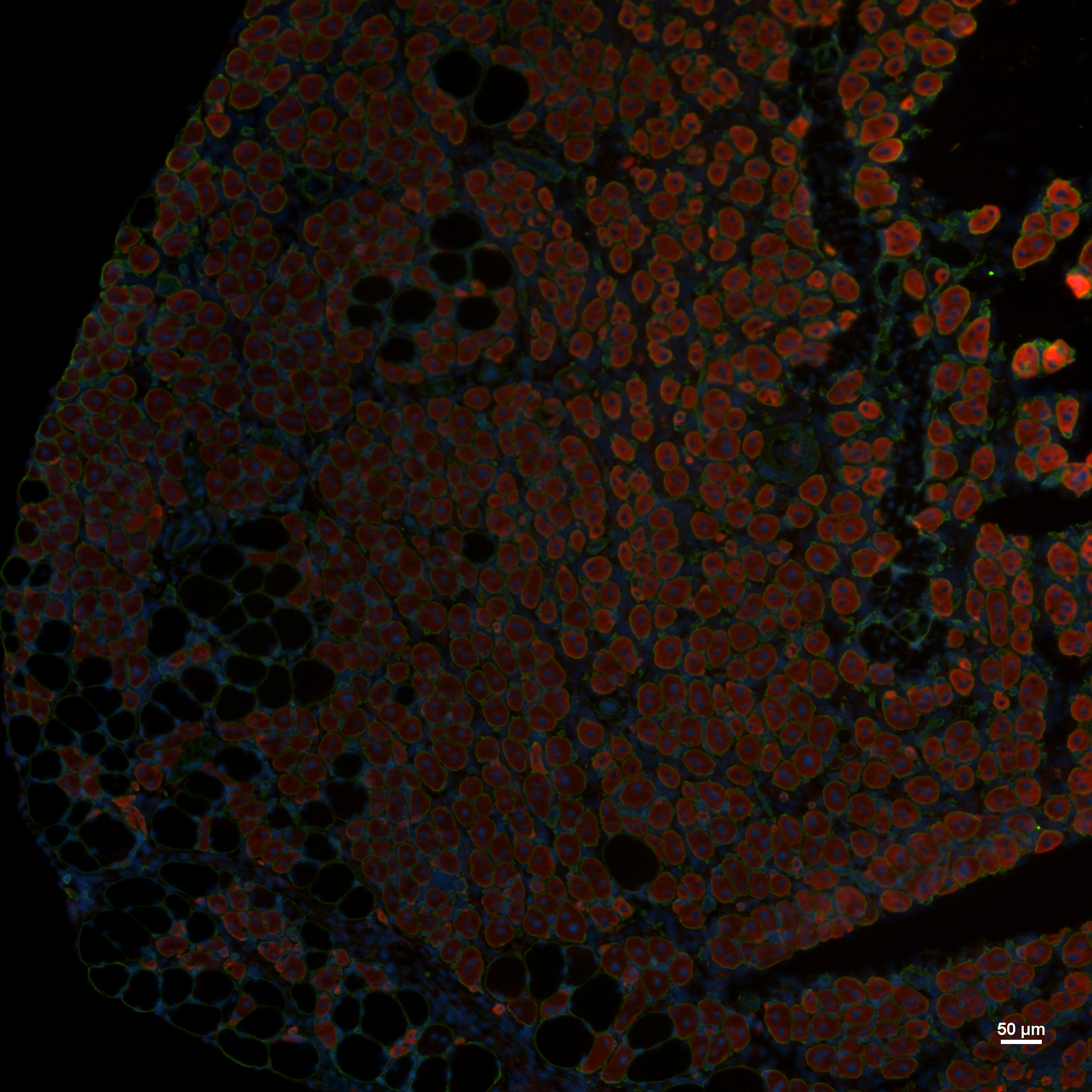

Supplement: Supplementary file 6 — Source data Fig. 3 [file 44319_2024_197_MOESM6_ESM.zip › Figure 3/3A/eMyHC-Laminin staining images/Ern1flfl-Injured-Representative image with box.tif]

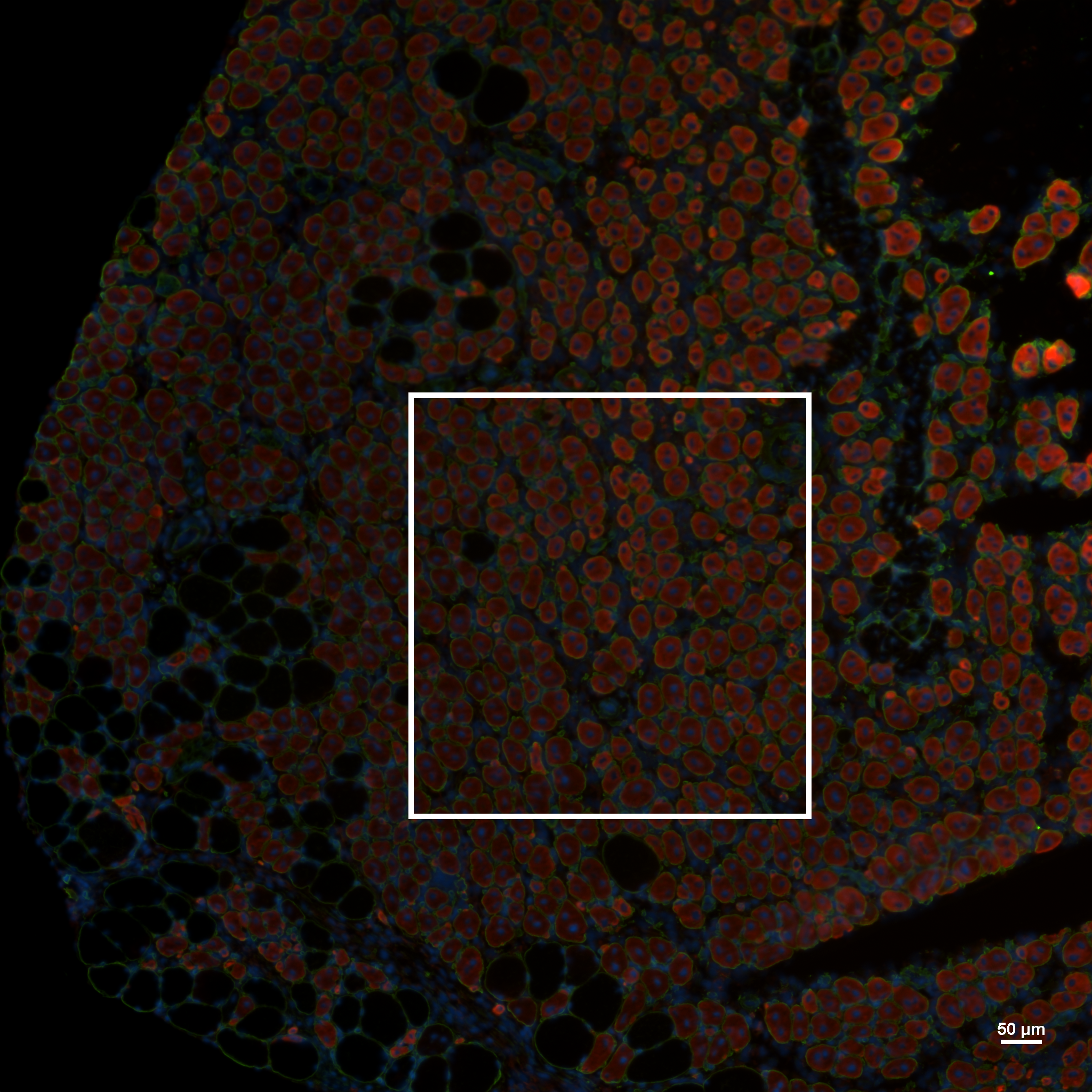

Supplement: Supplementary file 6 — Source data Fig. 3 [file 44319_2024_197_MOESM6_ESM.zip › Figure 3/3A/eMyHC-Laminin staining images/Ern1flfl-Injured-Representative image with box.tiff]

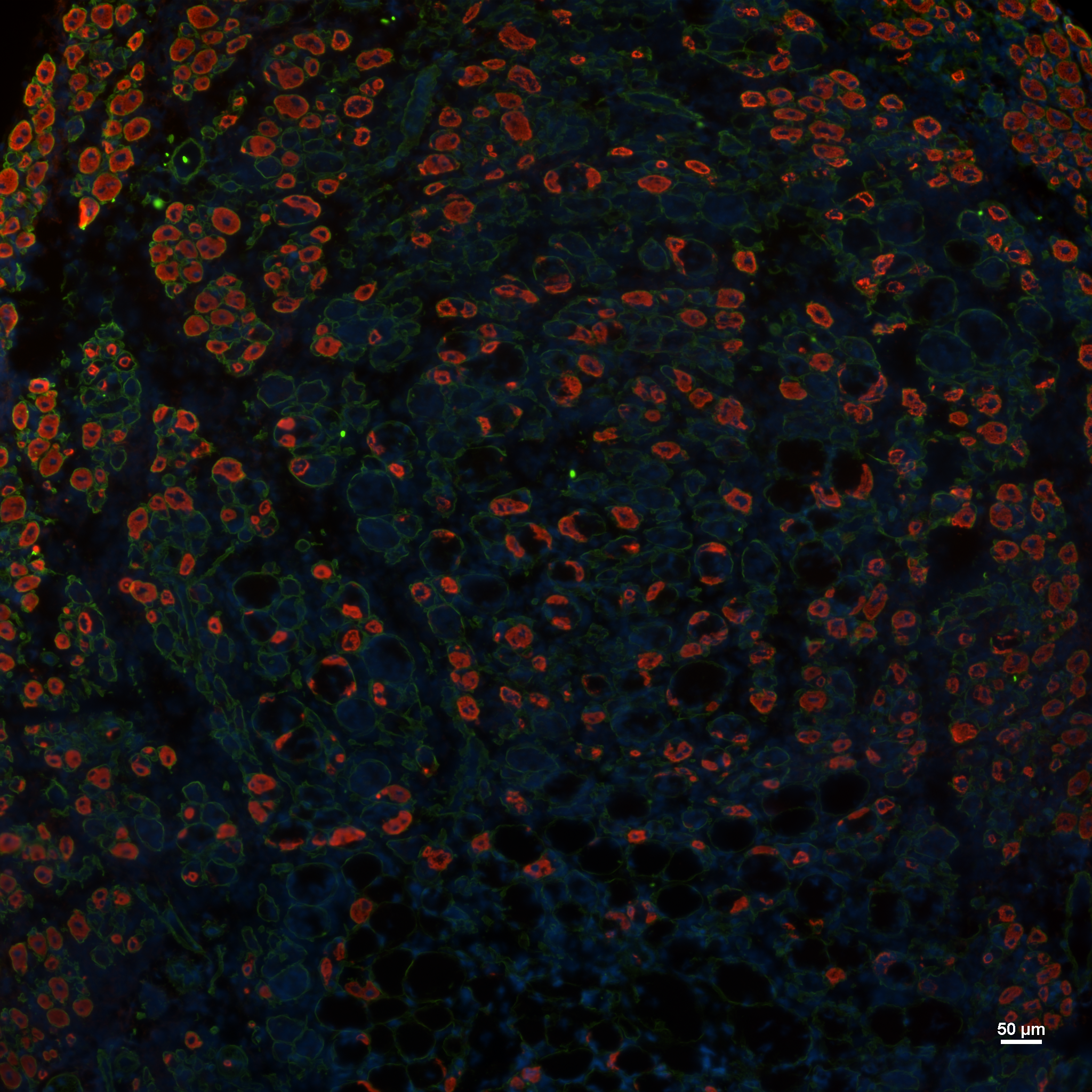

Supplement: Supplementary file 6 — Source data Fig. 3 [file 44319_2024_197_MOESM6_ESM.zip › Figure 3/3A/eMyHC-Laminin staining images/Ern1scko-Injured-2.tif]

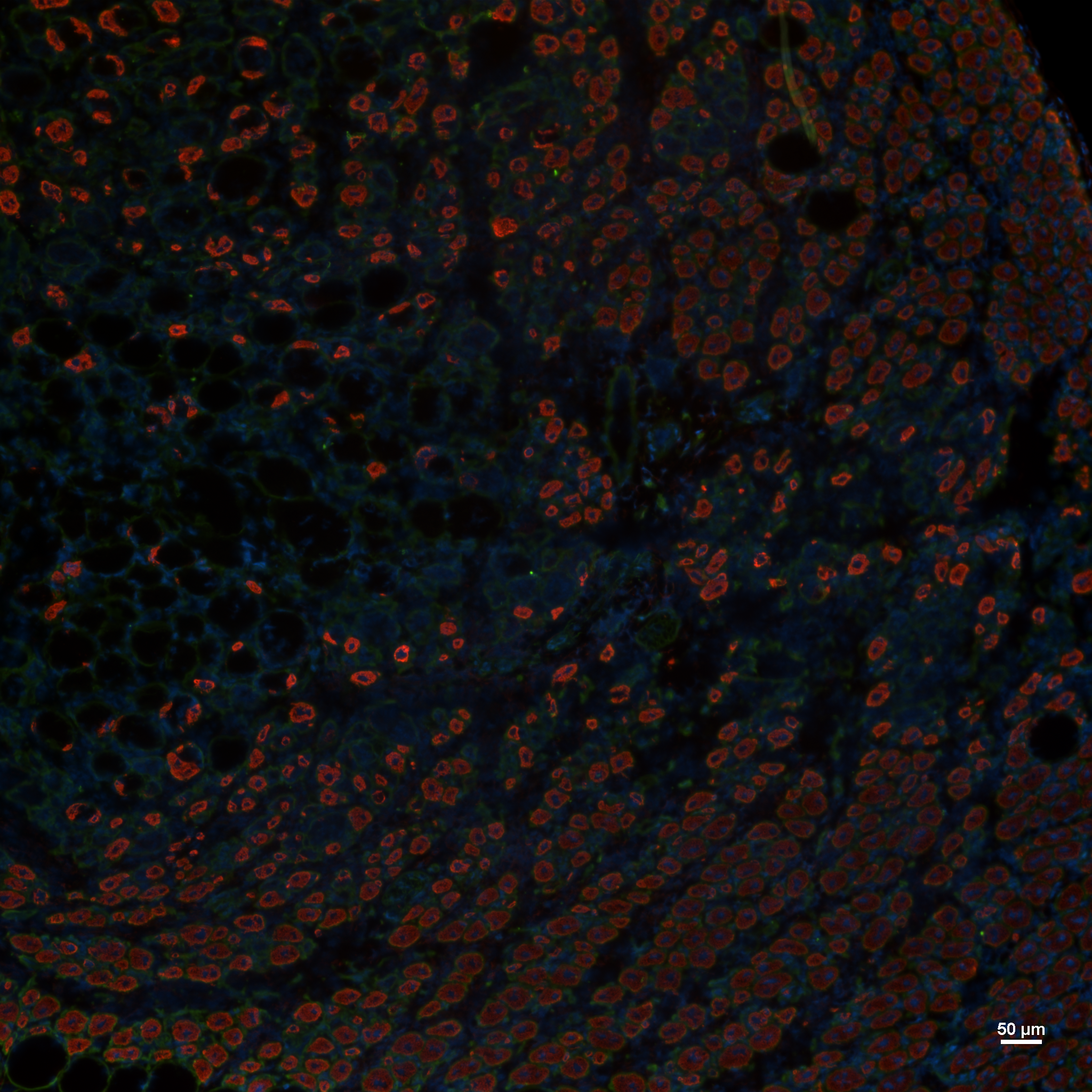

Supplement: Supplementary file 6 — Source data Fig. 3 [file 44319_2024_197_MOESM6_ESM.zip › Figure 3/3A/eMyHC-Laminin staining images/Ern1scko-Injured-Representative image with box.tif]

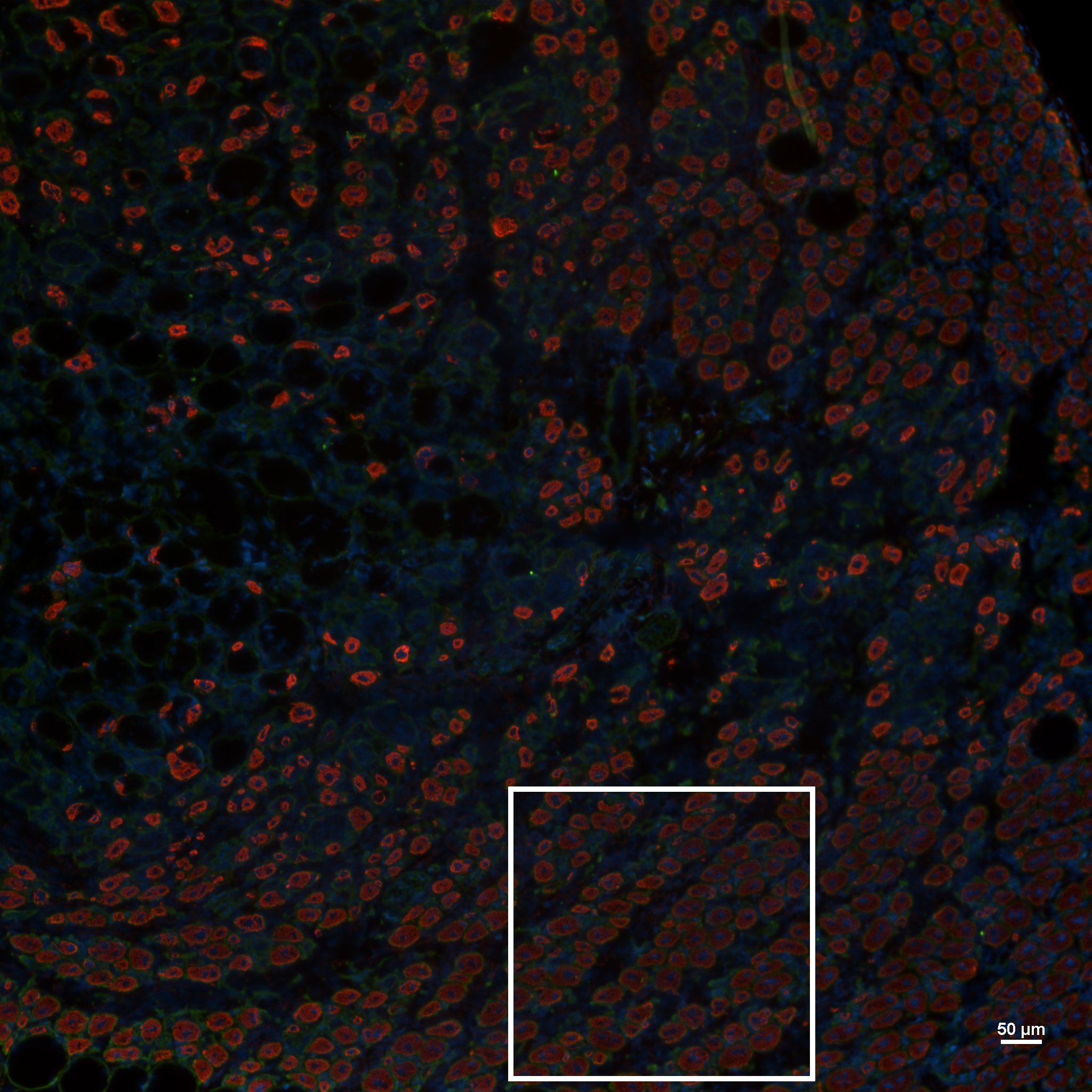

Supplement: Supplementary file 6 — Source data Fig. 3 [file 44319_2024_197_MOESM6_ESM.zip › Figure 3/3A/eMyHC-Laminin staining images/Ern1scko-Injured-Representative image with box.tiff]

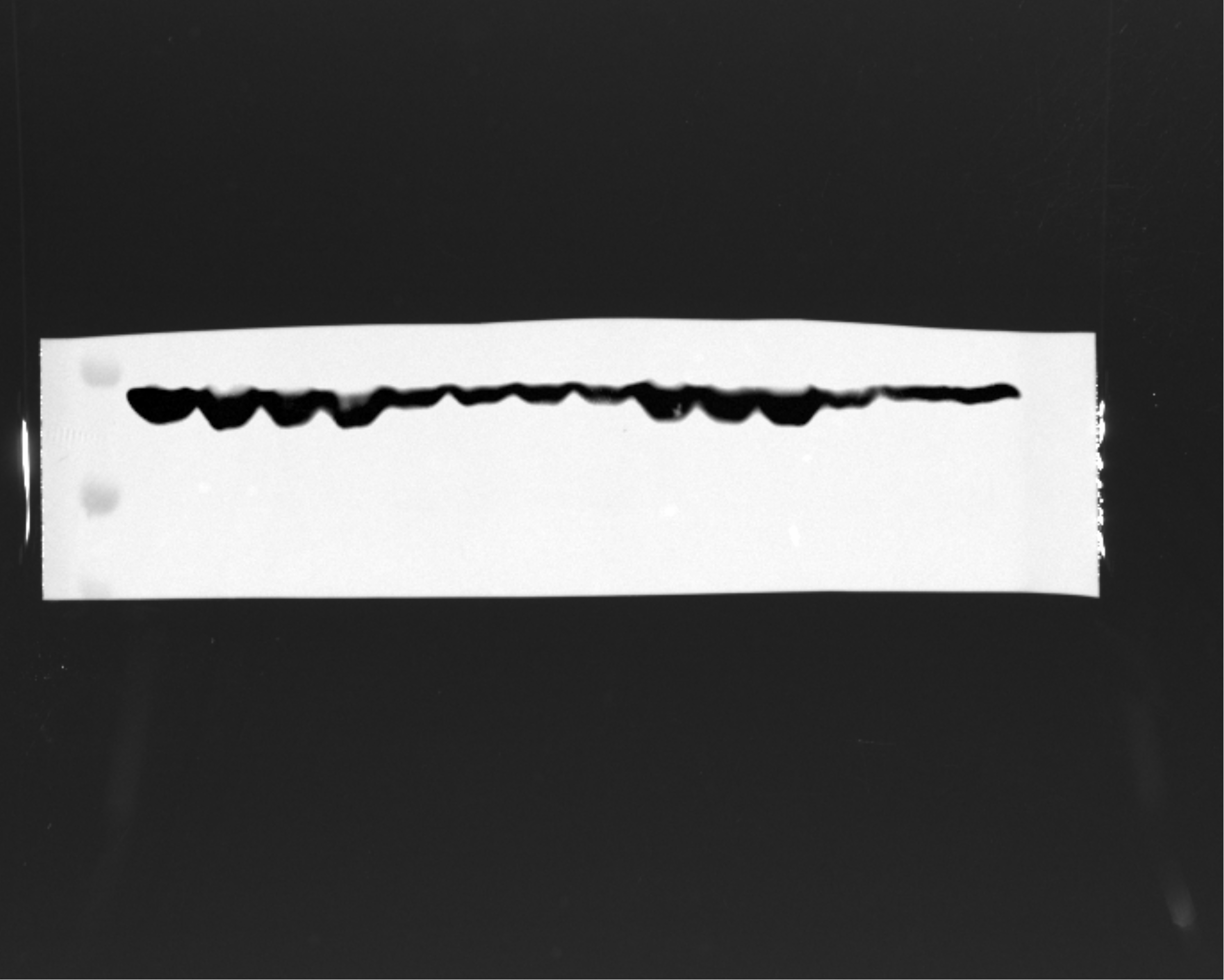

Supplement: Supplementary file 6 — Source data Fig. 3 [file 44319_2024_197_MOESM6_ESM.zip › Figure 3/3E-F/GAPDH.tif]

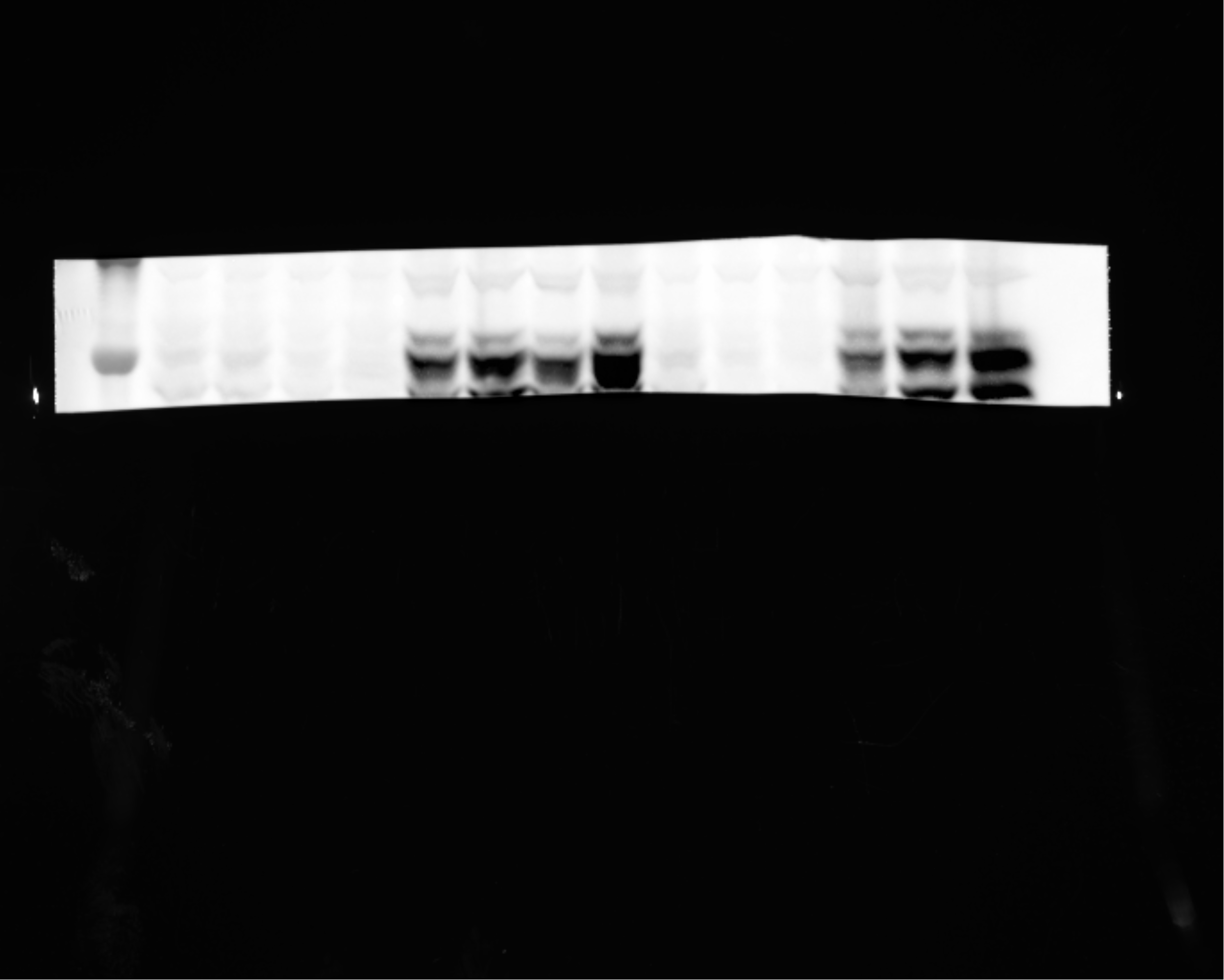

Supplement: Supplementary file 6 — Source data Fig. 3 [file 44319_2024_197_MOESM6_ESM.zip › Figure 3/3E-F/MyoD.tif]

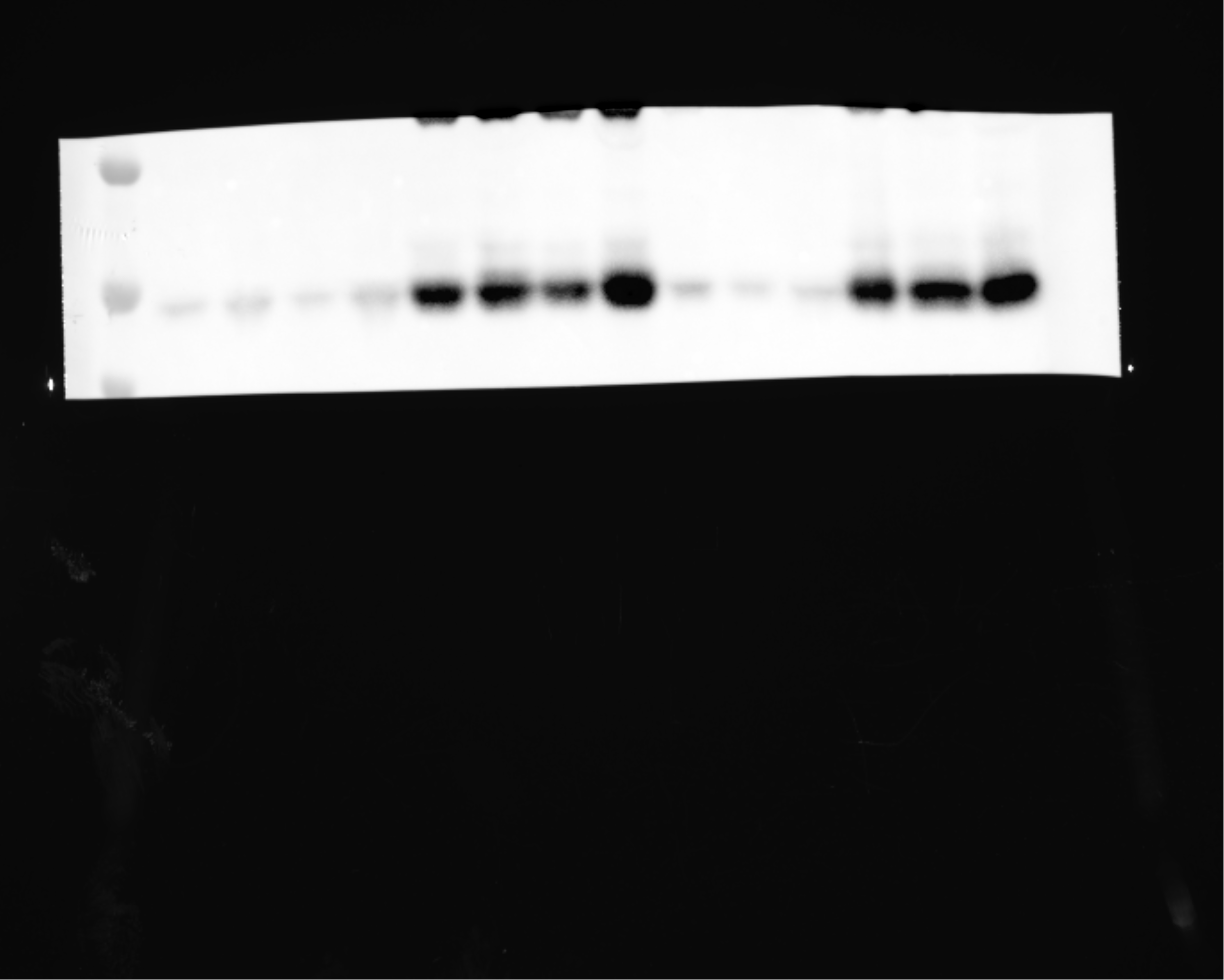

Supplement: Supplementary file 6 — Source data Fig. 3 [file 44319_2024_197_MOESM6_ESM.zip › Figure 3/3E-F/Myogenin.tif]

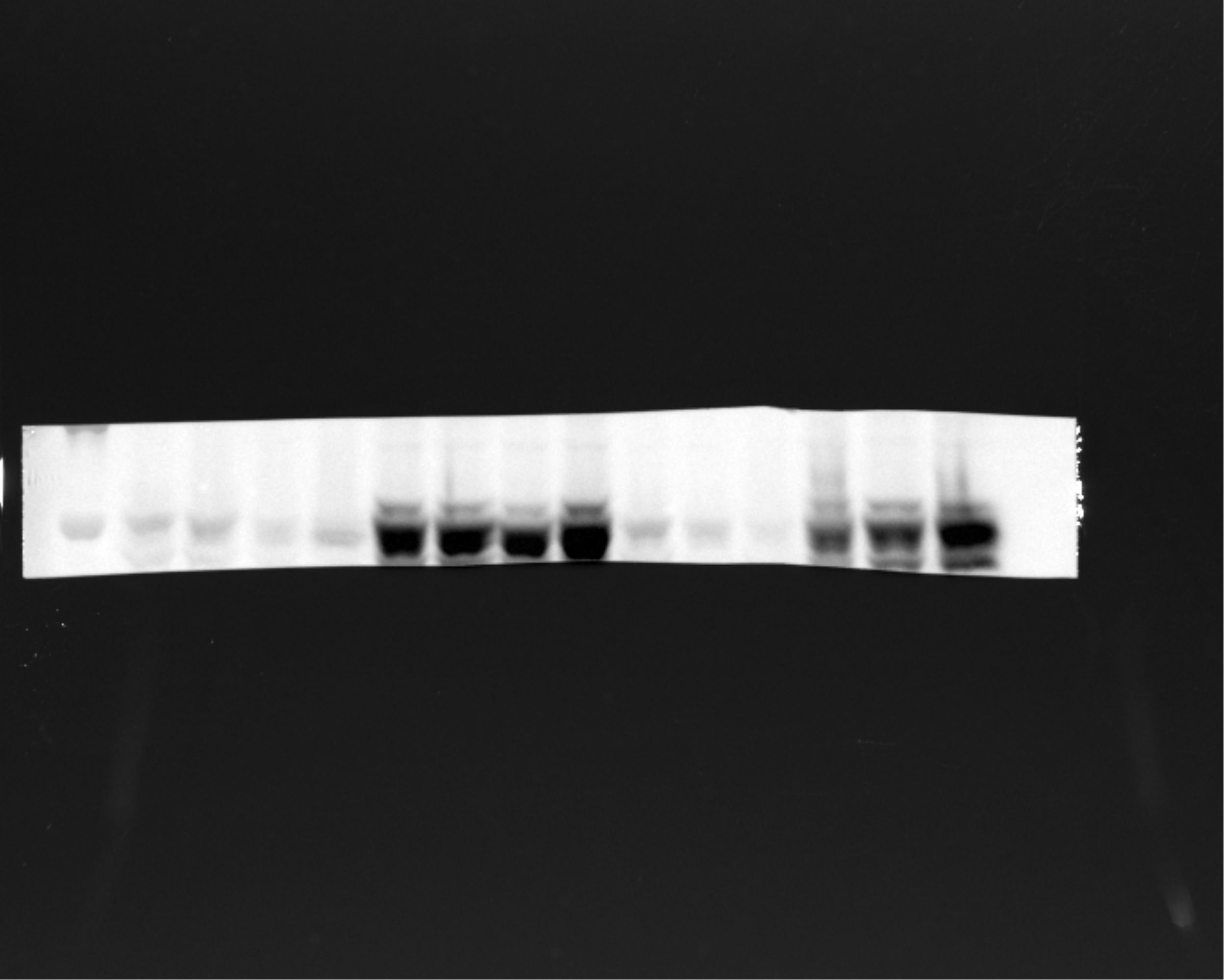

Supplement: Supplementary file 6 — Source data Fig. 3 [file 44319_2024_197_MOESM6_ESM.zip › Figure 3/3E-F/Pax7.tif]

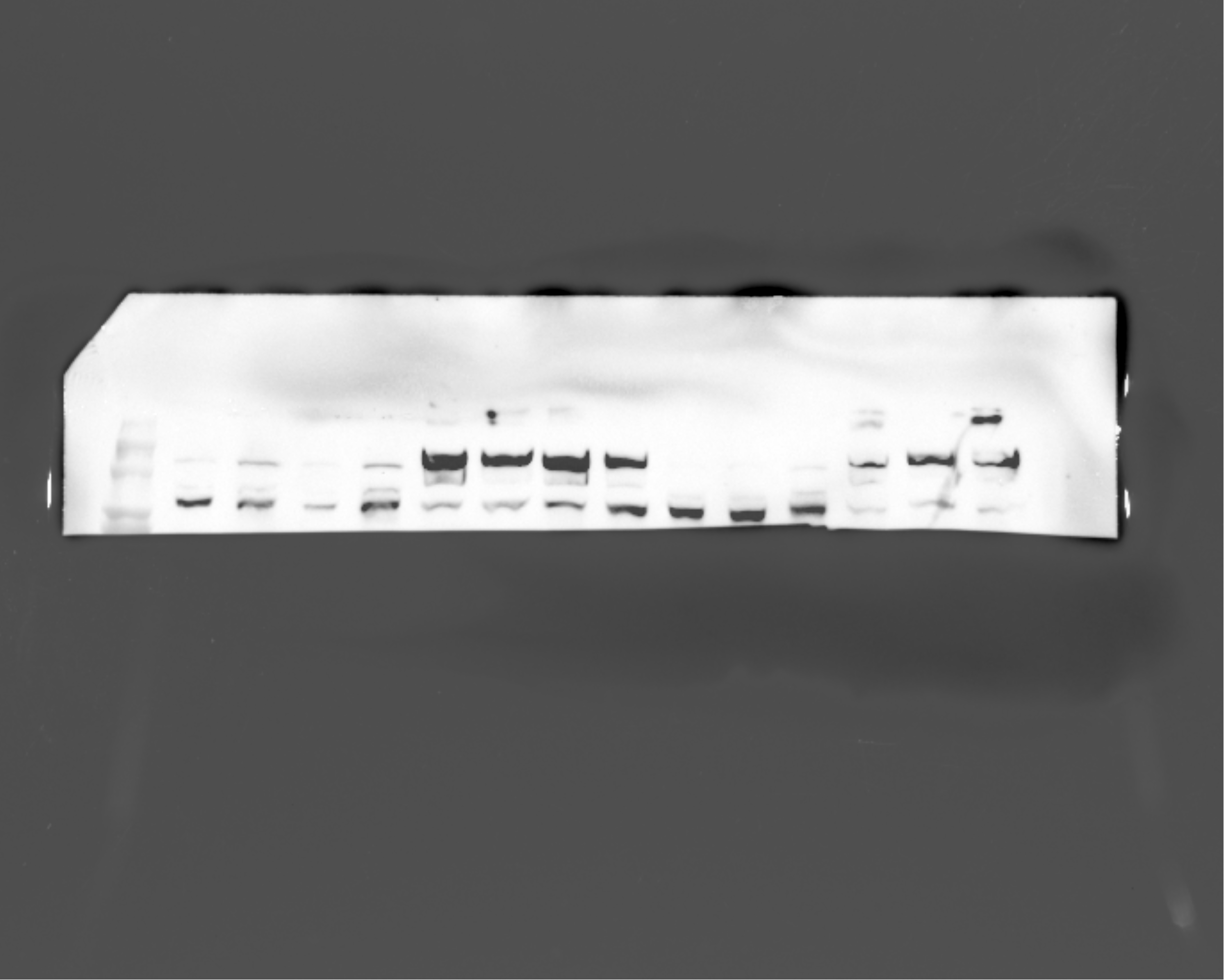

Supplement: Supplementary file 6 — Source data Fig. 3 [file 44319_2024_197_MOESM6_ESM.zip › Figure 3/3E-F/Total IRE1a.tif]

## Slide 1
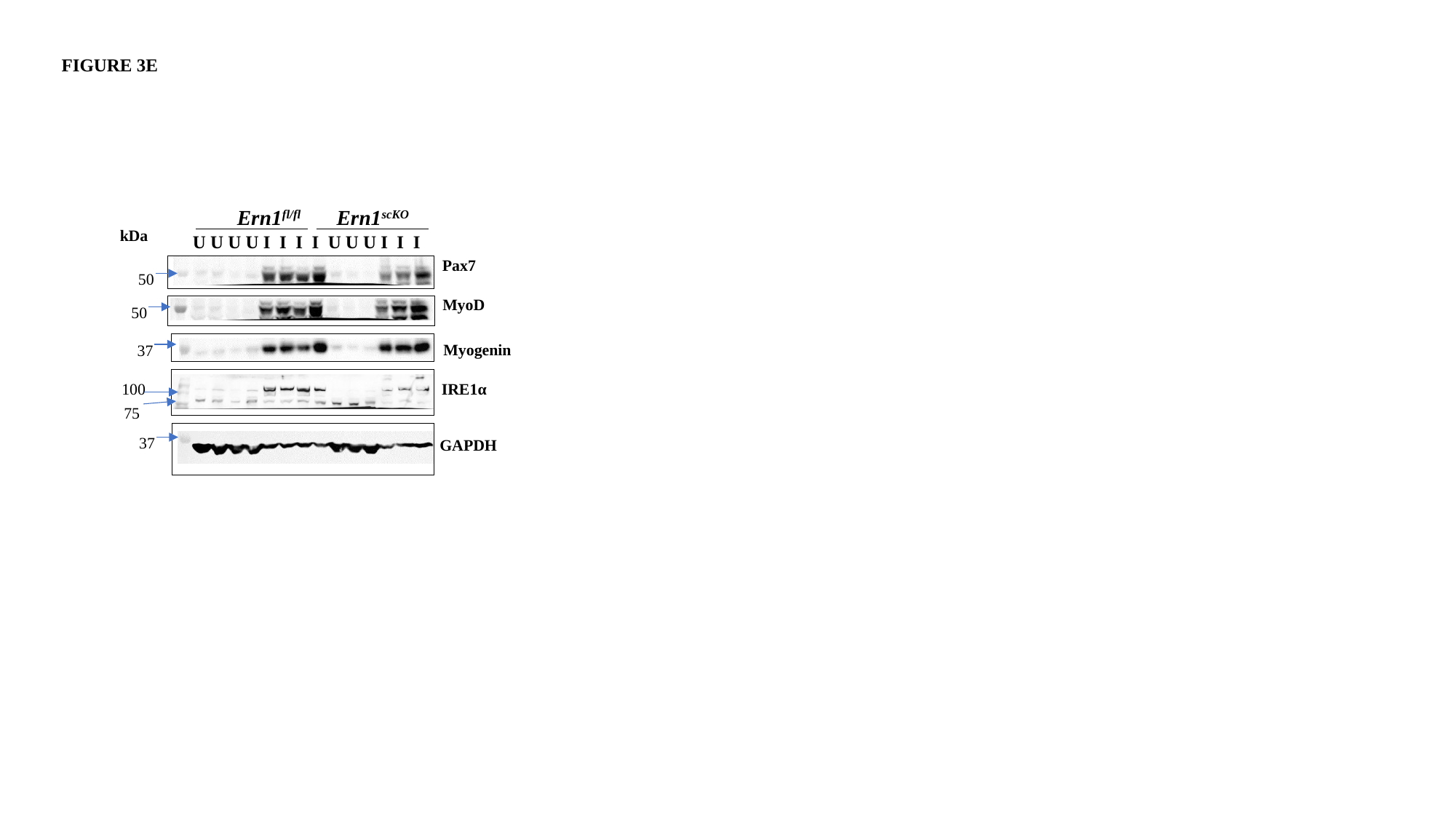

FIGURE 3E
Ern1fl/fl
Ern1scKO
kDa
U U U U I I I I U U U I I I
Pax7
50
MyoD
50
Myogenin
37
IRE1α
100
75
37
GAPDH

Supplement: Supplementary file 6 — Source data Fig. 3 [file 44319_2024_197_MOESM6_ESM.zip › Figure 3/3E-F/Western blot with annotation.pptx]

## Slide 1
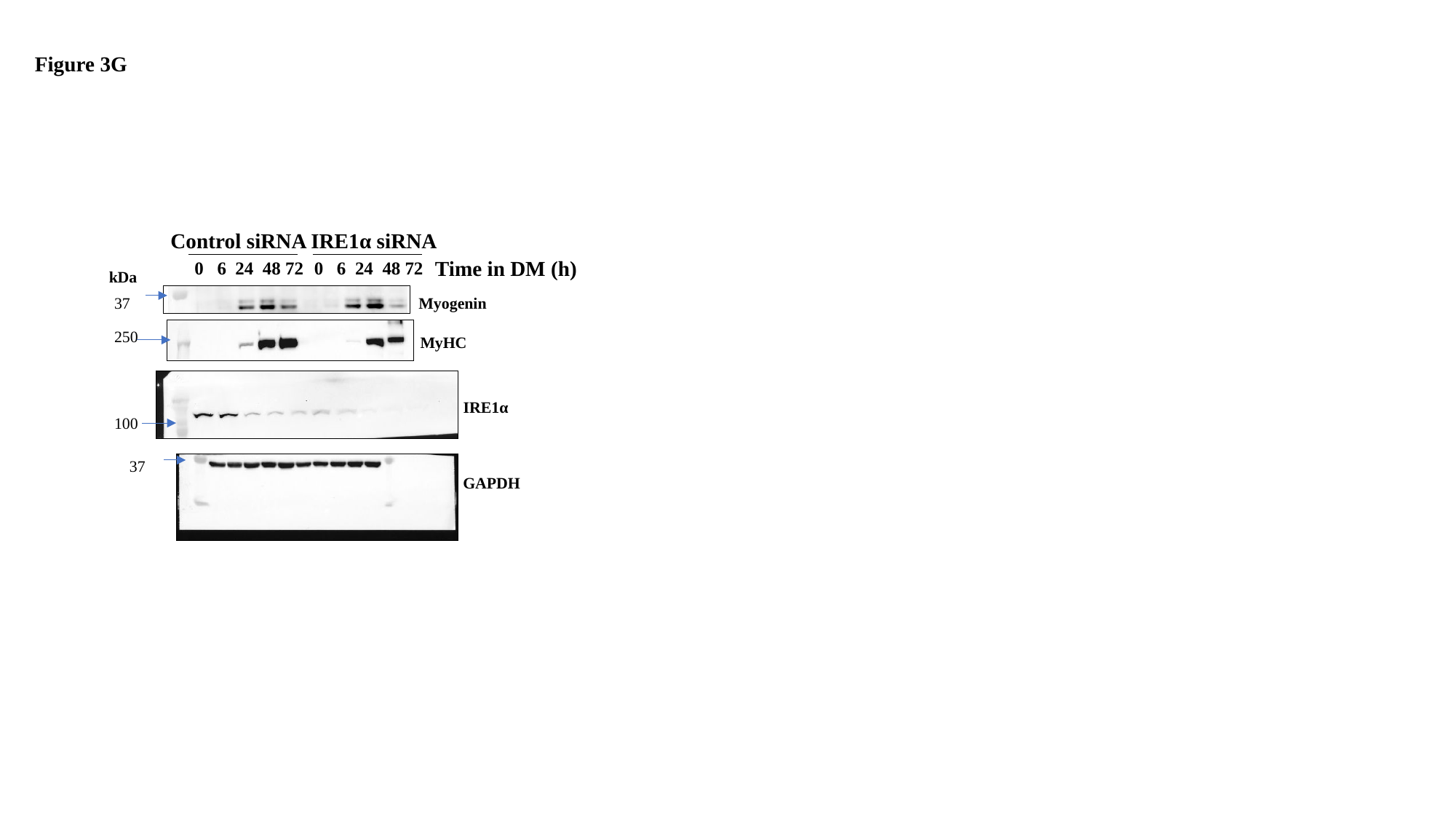

Figure 3G
Control siRNA
IRE1α siRNA
Time in DM (h)
 0 6 24 48 72
 0 6 24 48 72
kDa
37
Myogenin
250
MyHC
IRE1α
100
37
GAPDH

Supplement: Supplementary file 6 — Source data Fig. 3 [file 44319_2024_197_MOESM6_ESM.zip › Figure 3/3G/Western Blot with annotation.pptx]

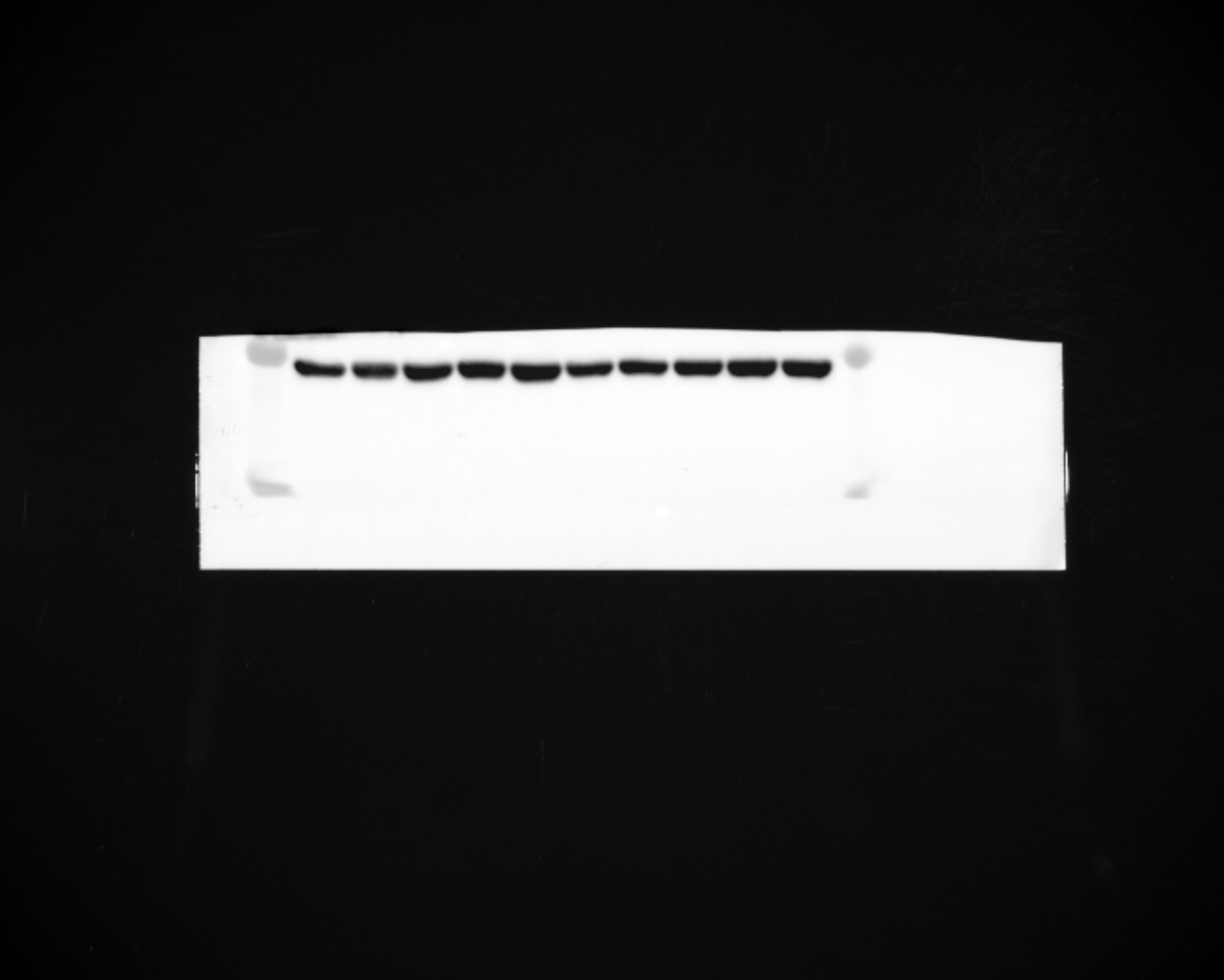

Supplement: Supplementary file 6 — Source data Fig. 3 [file 44319_2024_197_MOESM6_ESM.zip › Figure 3/3G/Western-GAPDH.tif]

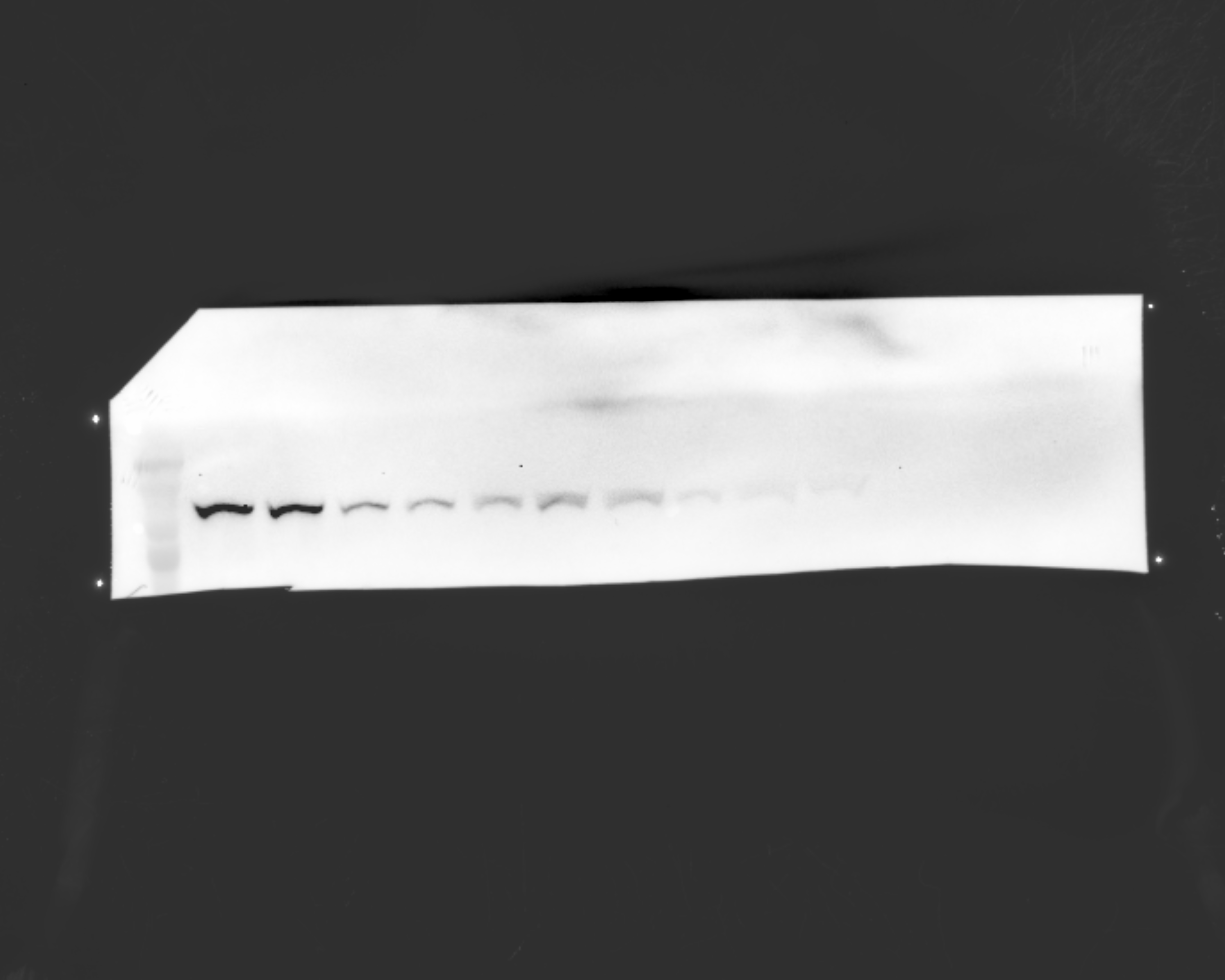

Supplement: Supplementary file 6 — Source data Fig. 3 [file 44319_2024_197_MOESM6_ESM.zip › Figure 3/3G/Western-IRE1a.tif]

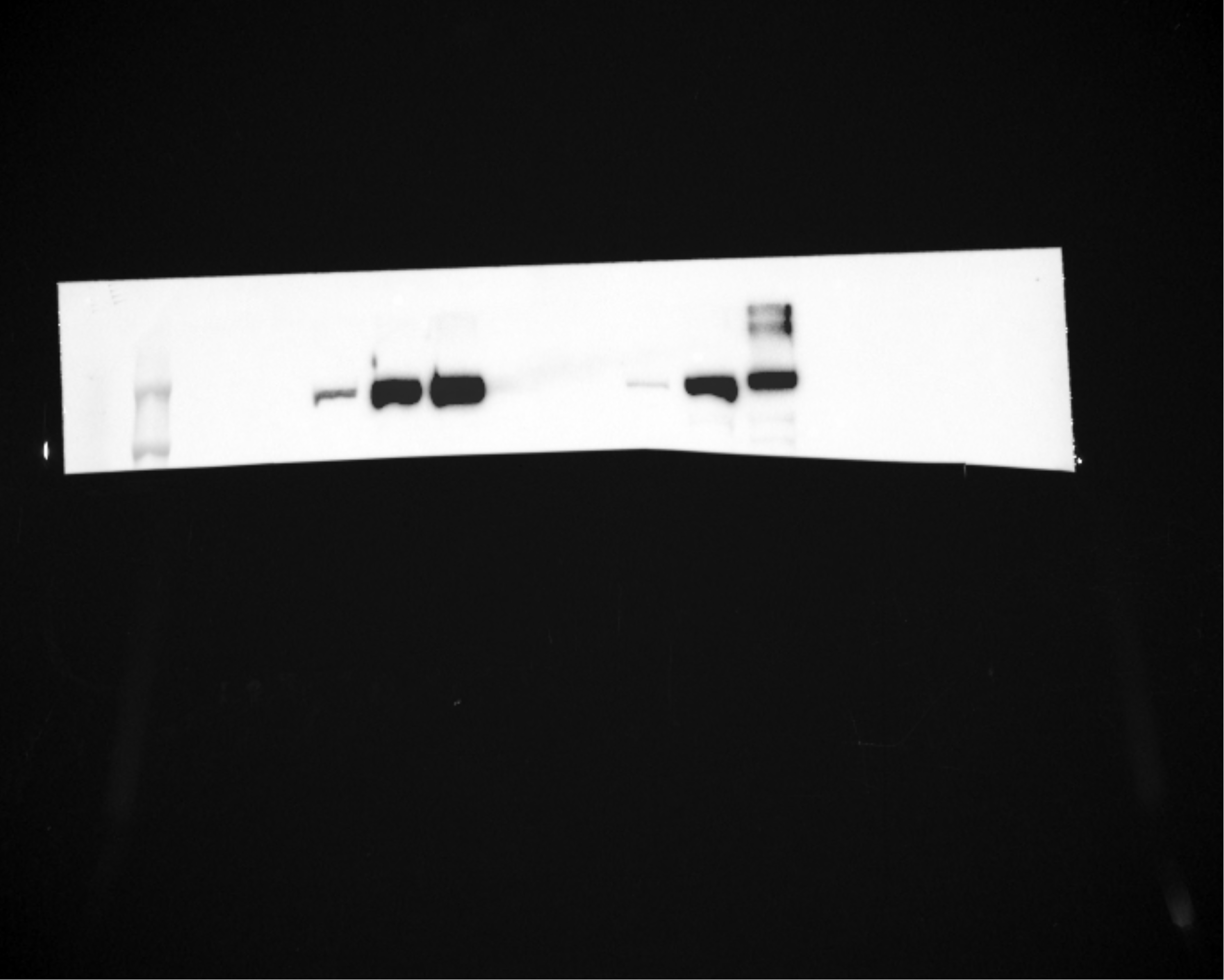

Supplement: Supplementary file 6 — Source data Fig. 3 [file 44319_2024_197_MOESM6_ESM.zip › Figure 3/3G/Western-MyHC.tif]

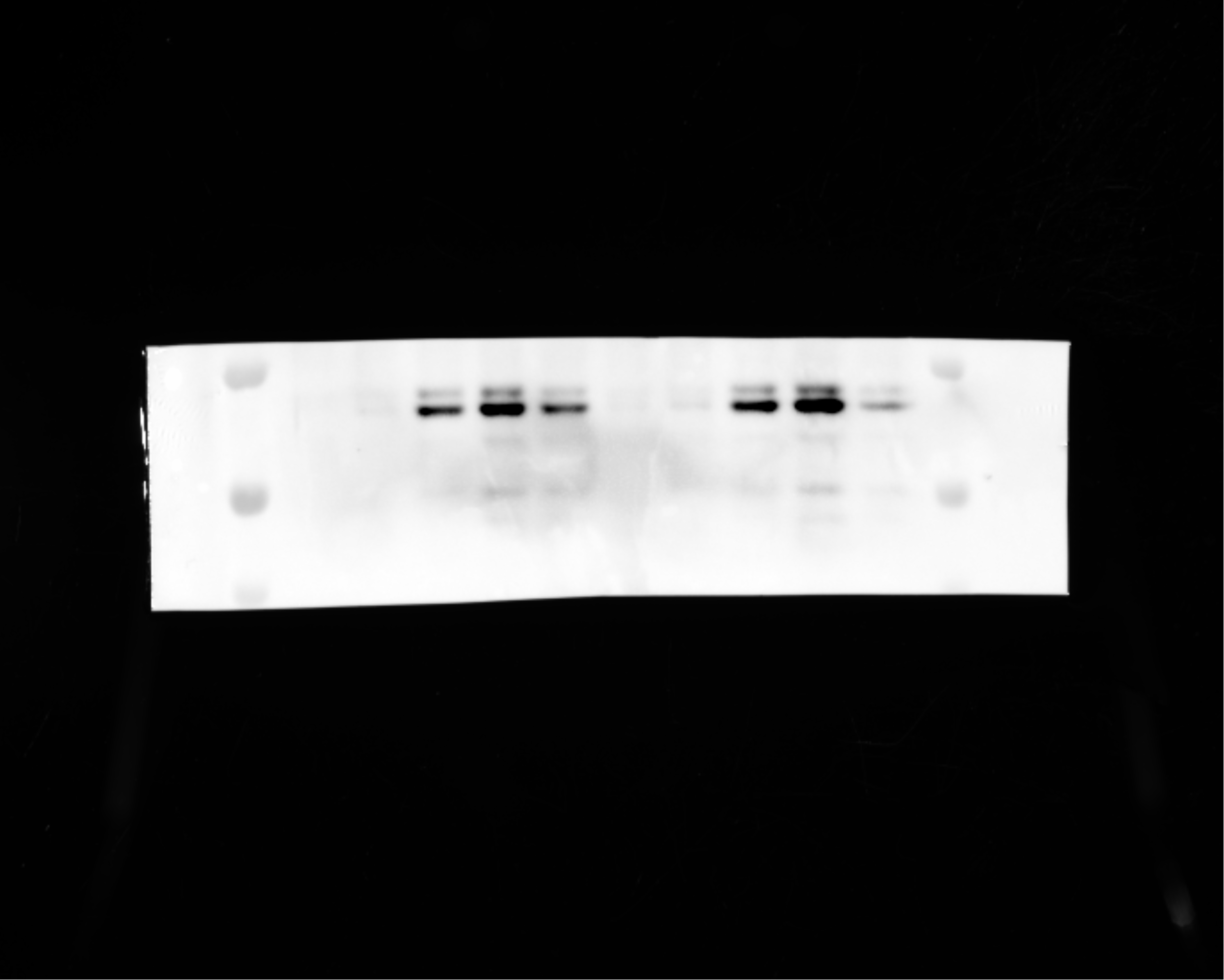

Supplement: Supplementary file 6 — Source data Fig. 3 [file 44319_2024_197_MOESM6_ESM.zip › Figure 3/3G/Western-Myogenin.tif]

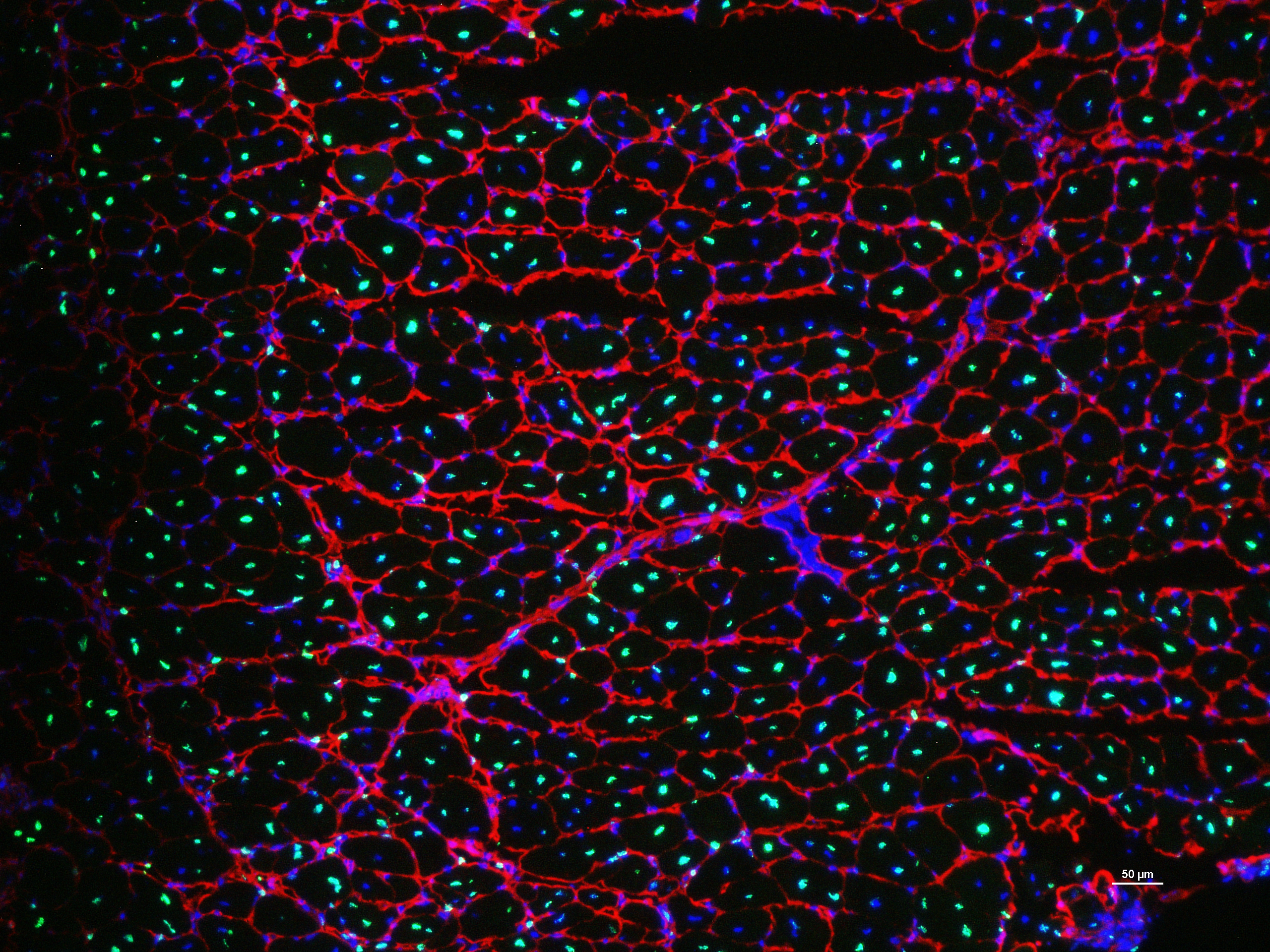

Supplement: Supplementary file 7 — Source data Fig. 4 [file 44319_2024_197_MOESM7_ESM.zip › Figure 4/4A-C/4A/EdU-Laminin staining images/Ern1 flfl -2.tif]

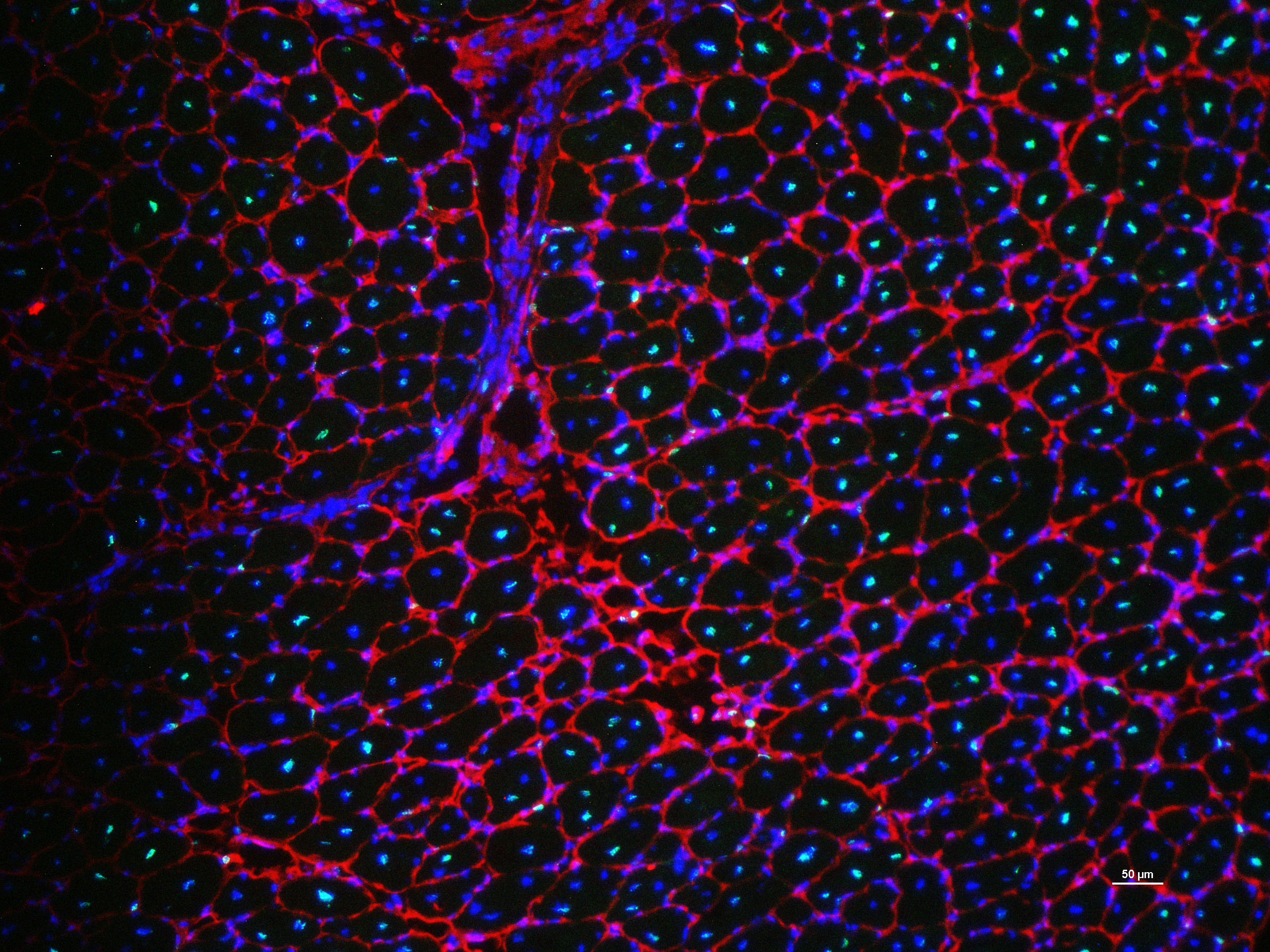

Supplement: Supplementary file 7 — Source data Fig. 4 [file 44319_2024_197_MOESM7_ESM.zip › Figure 4/4A-C/4A/EdU-Laminin staining images/Ern1 flfl -3.tif]

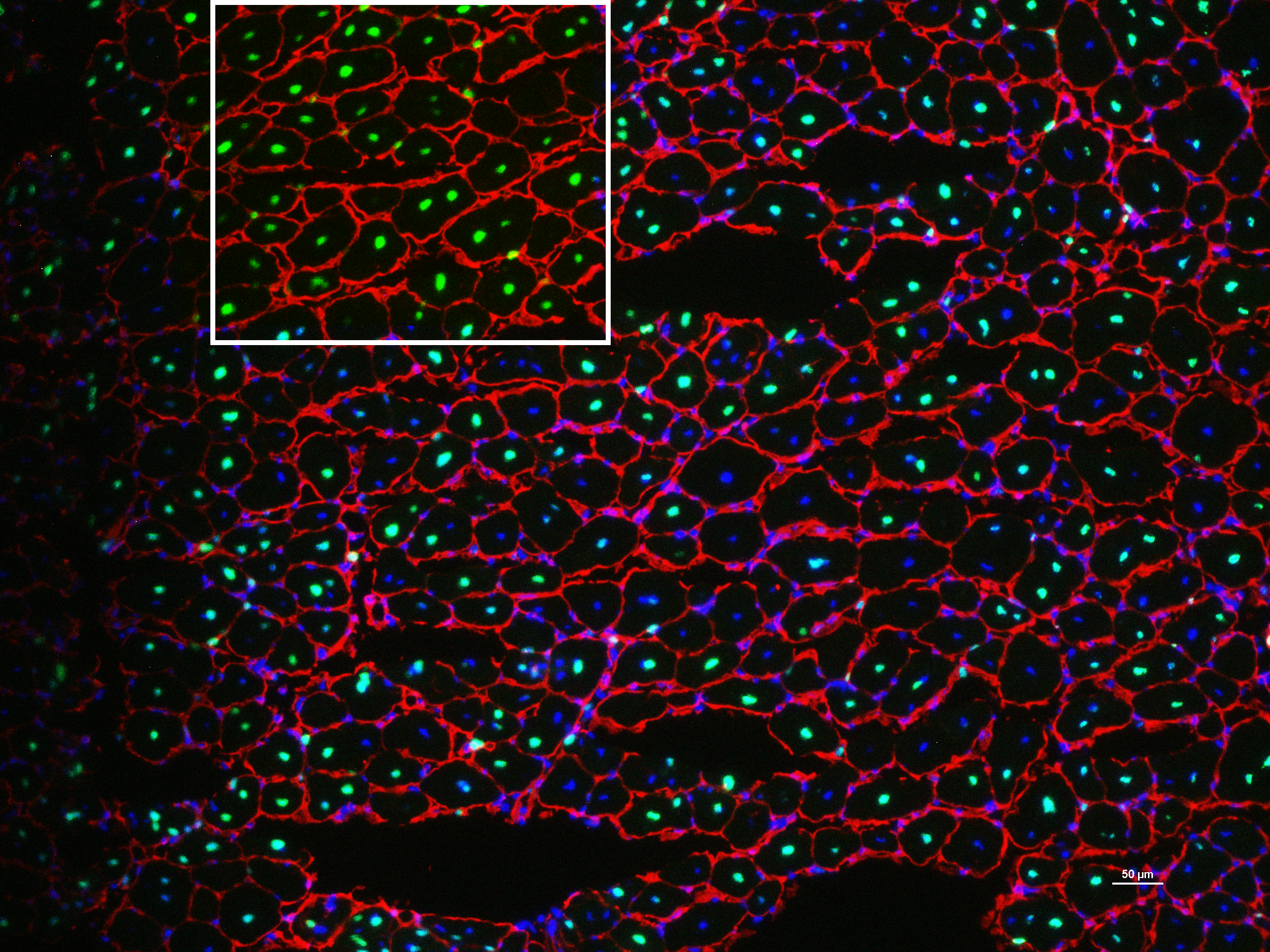

Supplement: Supplementary file 7 — Source data Fig. 4 [file 44319_2024_197_MOESM7_ESM.zip › Figure 4/4A-C/4A/EdU-Laminin staining images/Ern1 flfl Representative image with box.tiff]

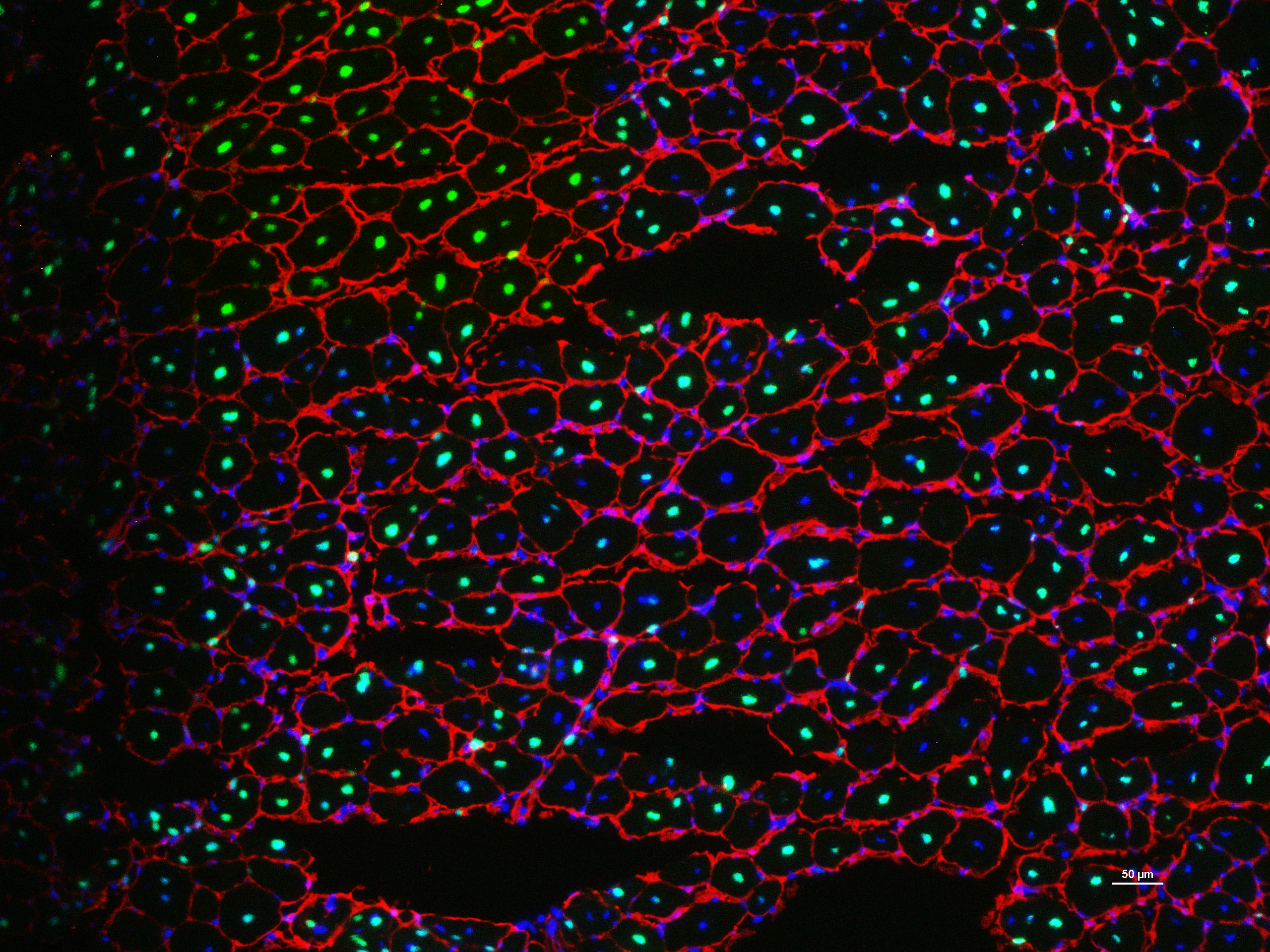

Supplement: Supplementary file 7 — Source data Fig. 4 [file 44319_2024_197_MOESM7_ESM.zip › Figure 4/4A-C/4A/EdU-Laminin staining images/Ern1 flfl Representative image.tif]

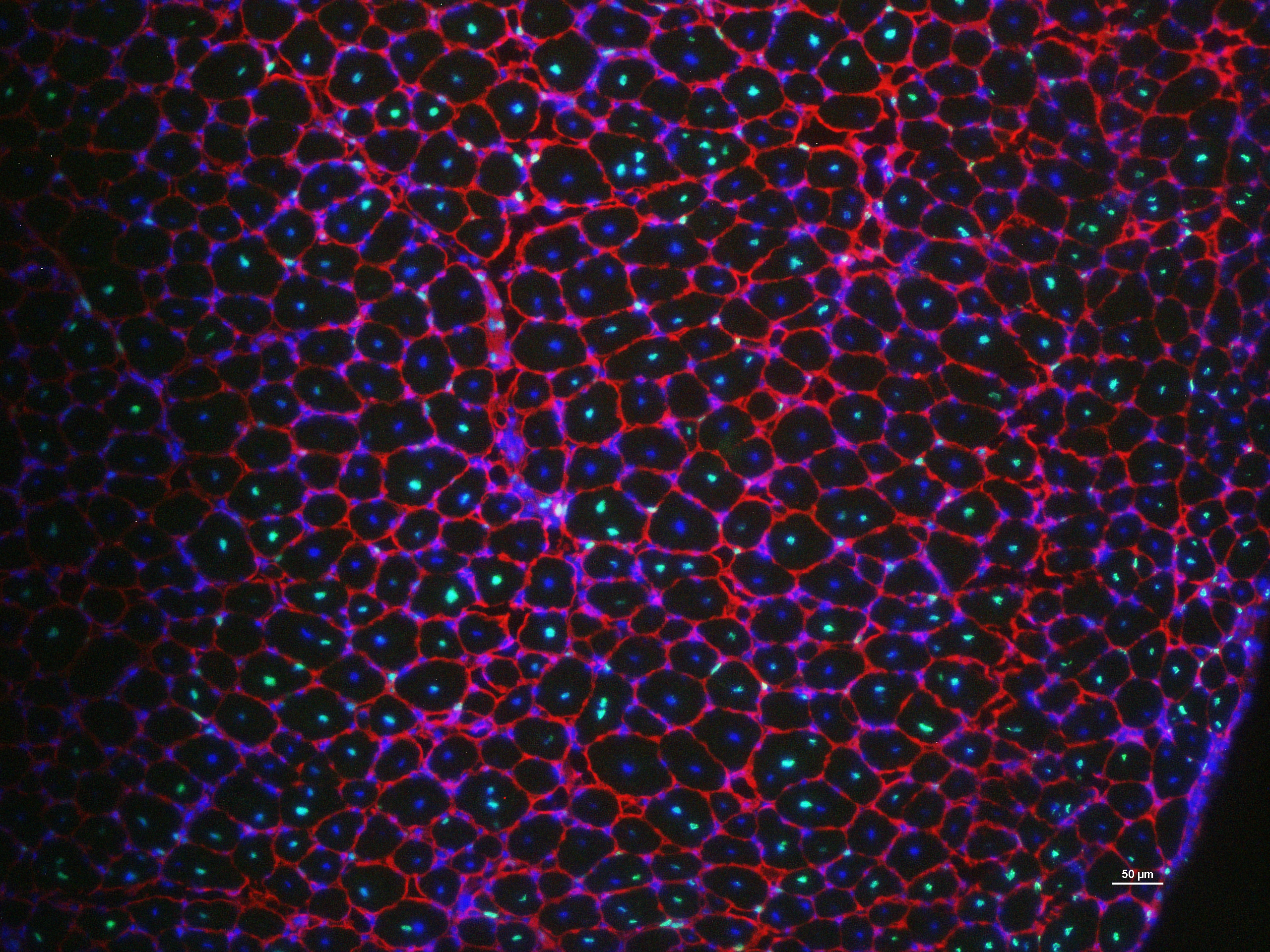

Supplement: Supplementary file 7 — Source data Fig. 4 [file 44319_2024_197_MOESM7_ESM.zip › Figure 4/4A-C/4A/EdU-Laminin staining images/Ern1 scko -2.tif]

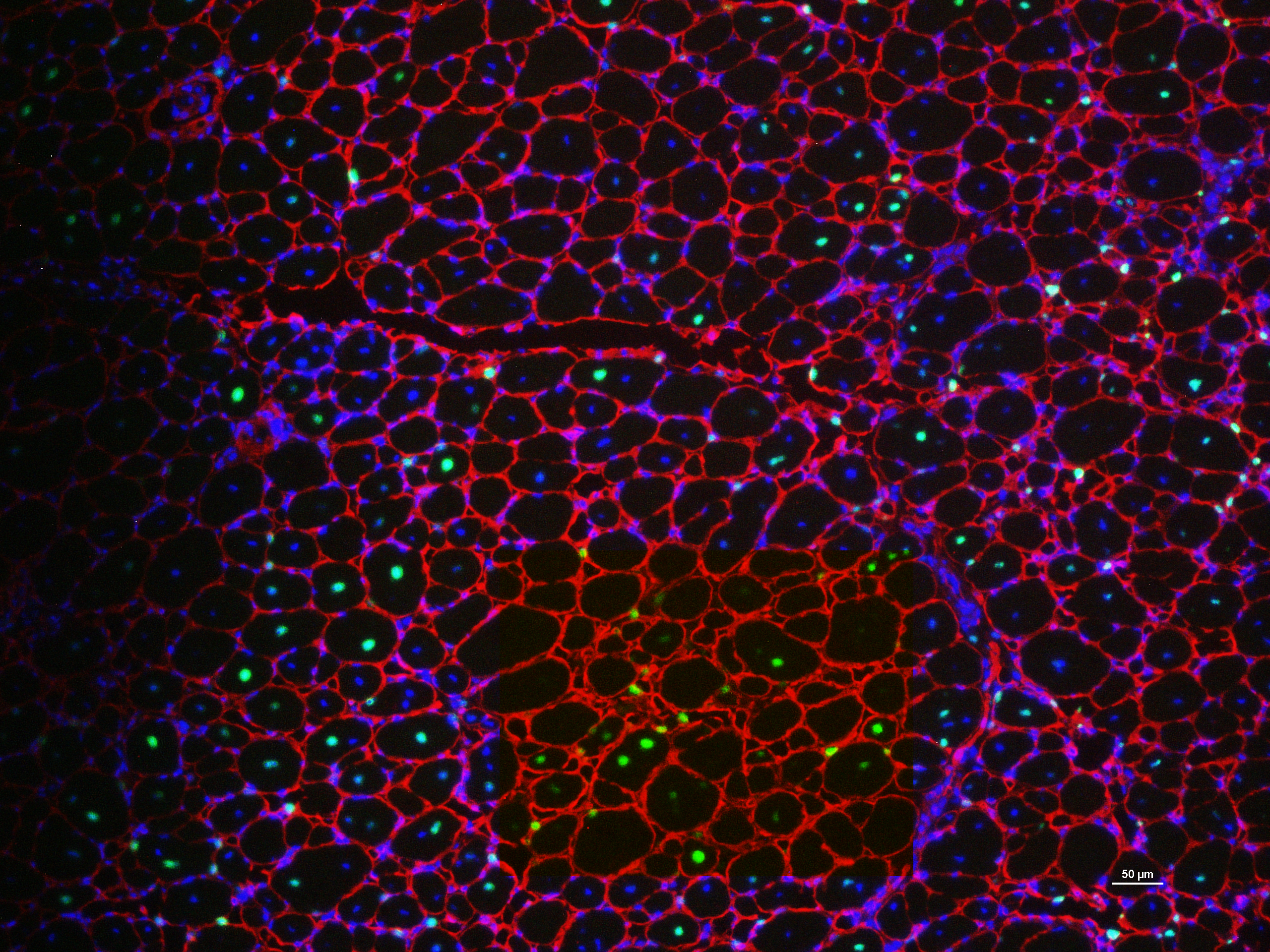

Supplement: Supplementary file 7 — Source data Fig. 4 [file 44319_2024_197_MOESM7_ESM.zip › Figure 4/4A-C/4A/EdU-Laminin staining images/Ern1 scko -3.tif]

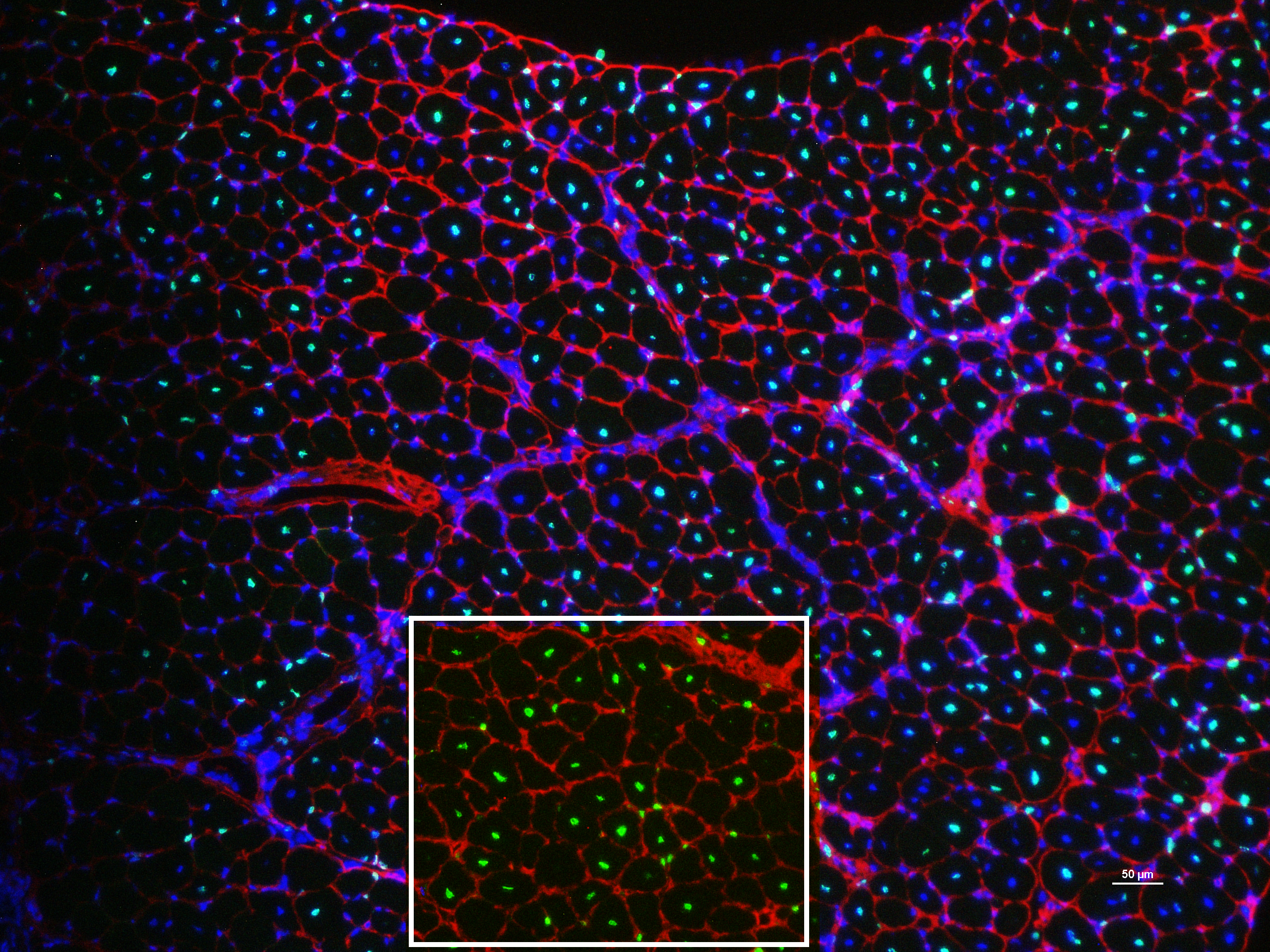

Supplement: Supplementary file 7 — Source data Fig. 4 [file 44319_2024_197_MOESM7_ESM.zip › Figure 4/4A-C/4A/EdU-Laminin staining images/Ern1 scko Representative image with box.tiff]

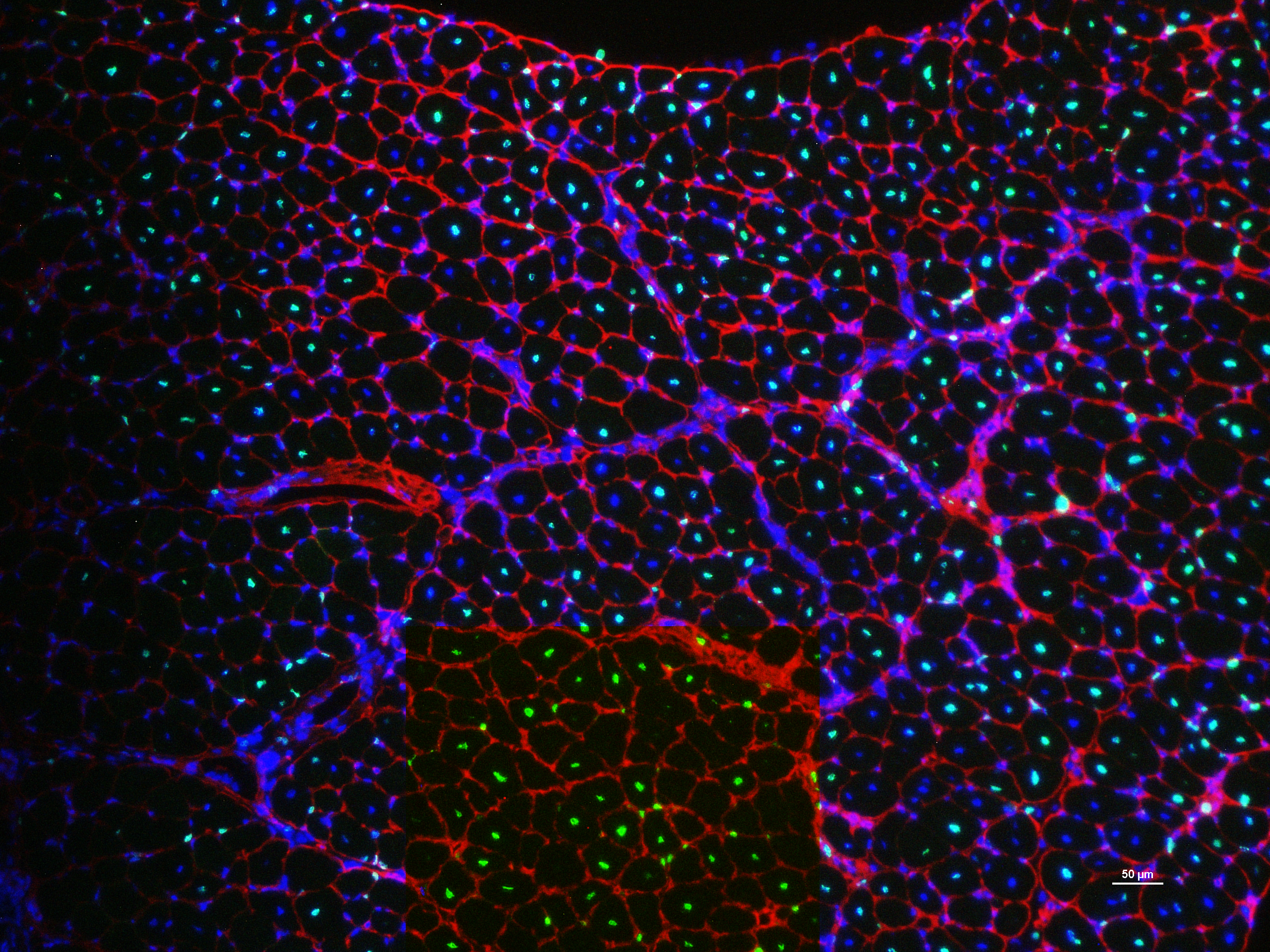

Supplement: Supplementary file 7 — Source data Fig. 4 [file 44319_2024_197_MOESM7_ESM.zip › Figure 4/4A-C/4A/EdU-Laminin staining images/Ern1 scko Representative image.tif]

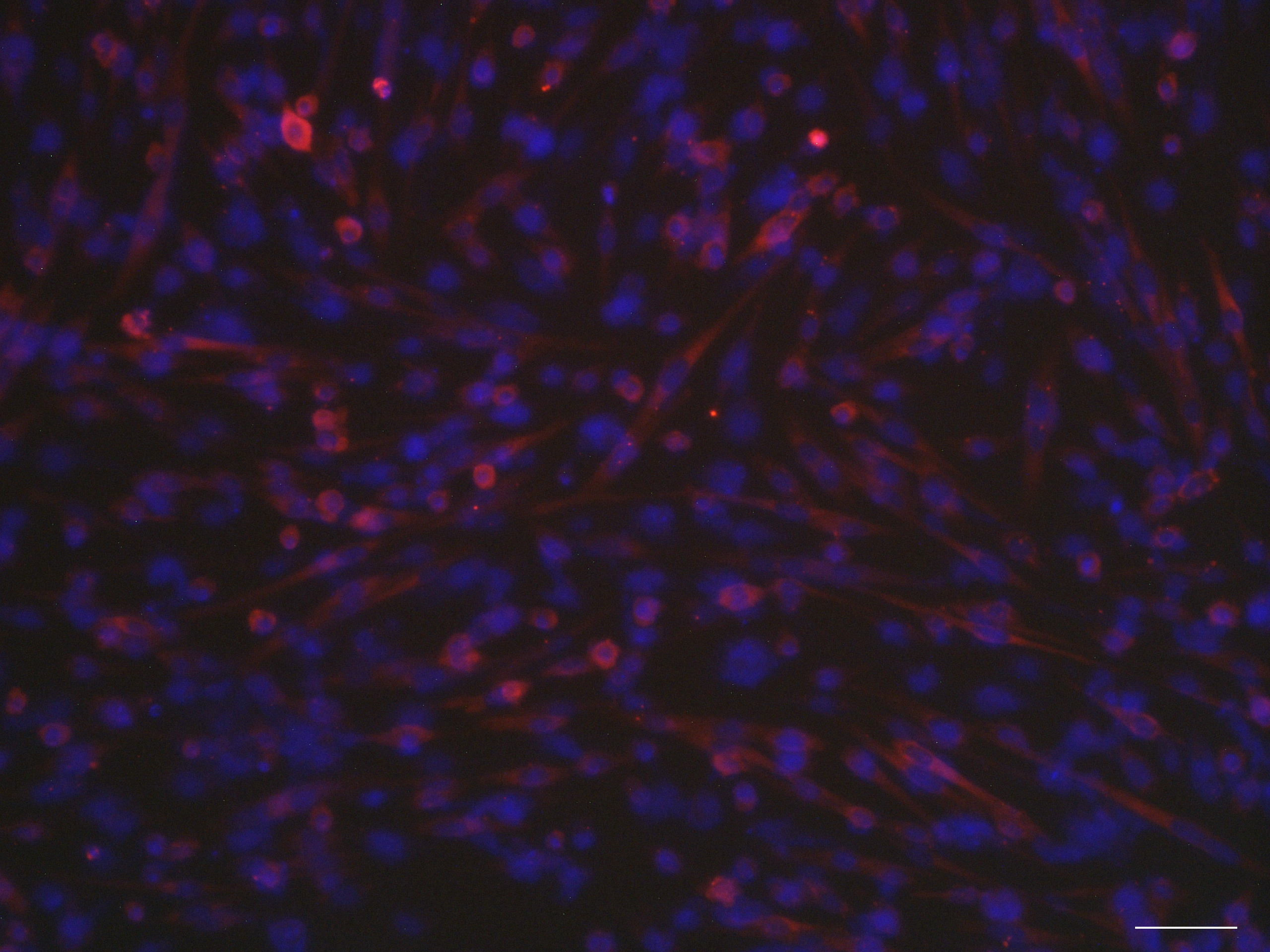

Supplement: Supplementary file 7 — Source data Fig. 4 [file 44319_2024_197_MOESM7_ESM.zip › Figure 4/4D-I/4D/IRE1 siRNA-MyHC images/24 h Con siRNA replicate 2.tif]

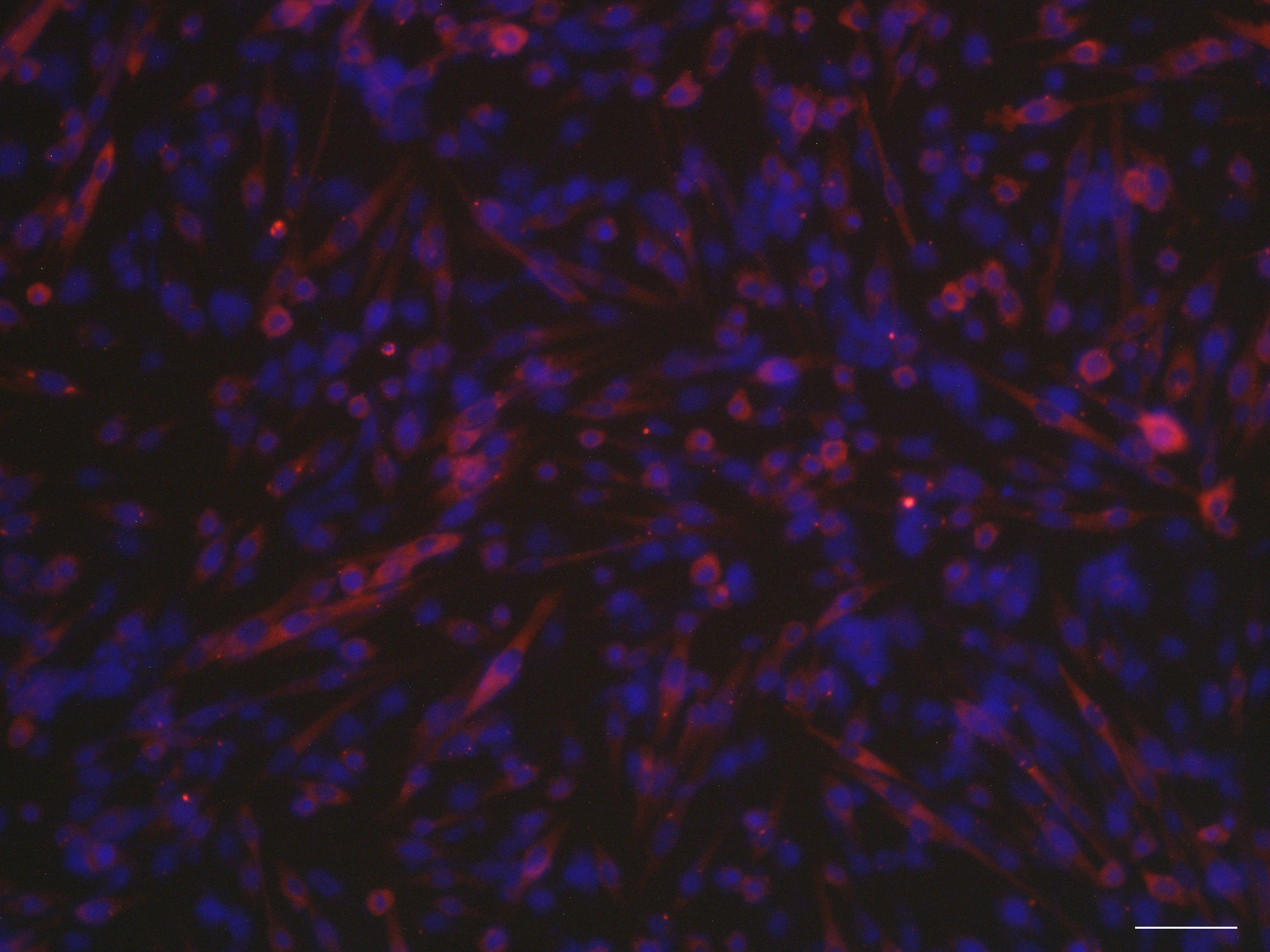

Supplement: Supplementary file 7 — Source data Fig. 4 [file 44319_2024_197_MOESM7_ESM.zip › Figure 4/4D-I/4D/IRE1 siRNA-MyHC images/24 h Con siRNA replicate 3.tif]

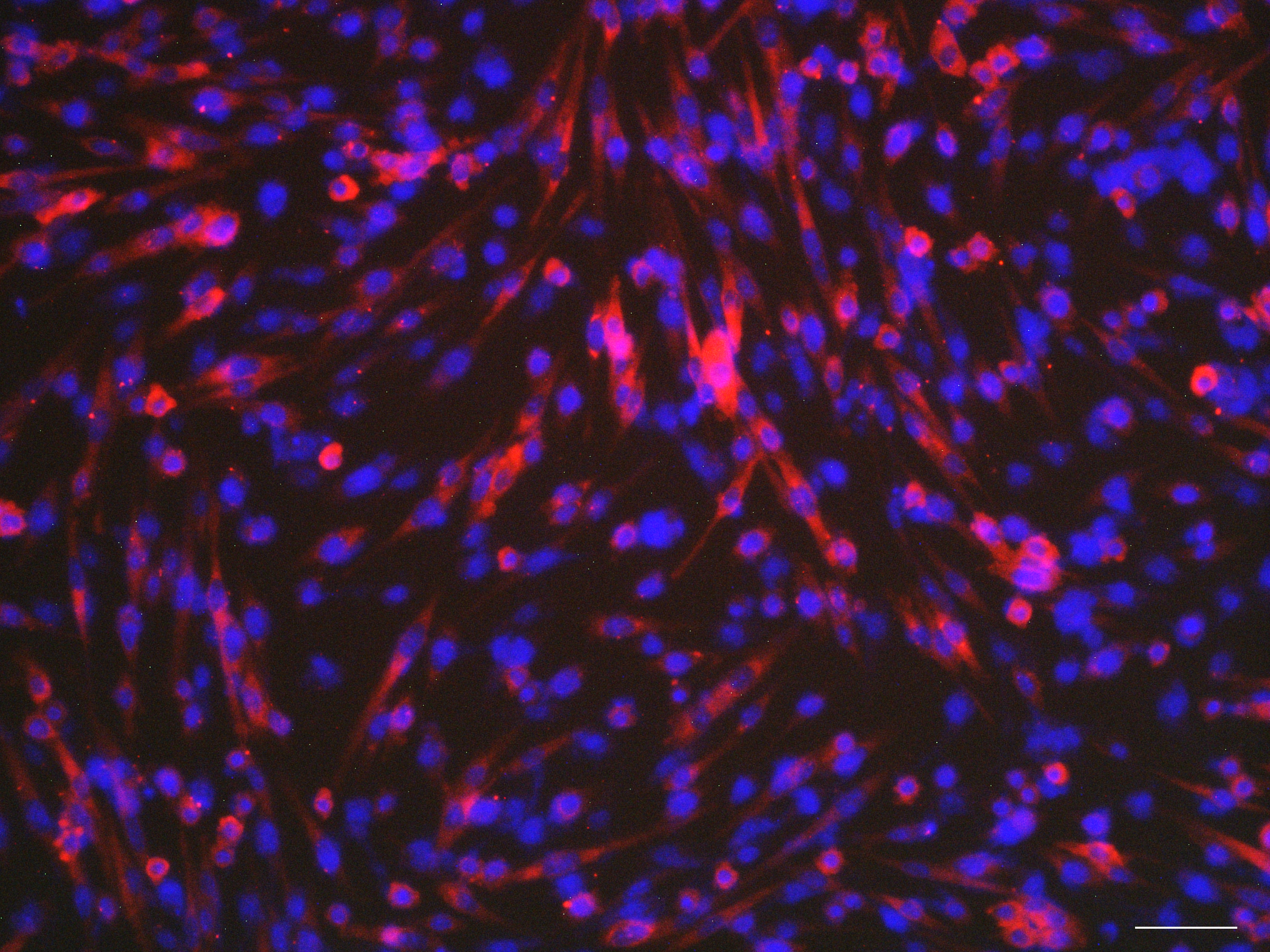

Supplement: Supplementary file 7 — Source data Fig. 4 [file 44319_2024_197_MOESM7_ESM.zip › Figure 4/4D-I/4D/IRE1 siRNA-MyHC images/24 h Con siRNA Representative image.tif]

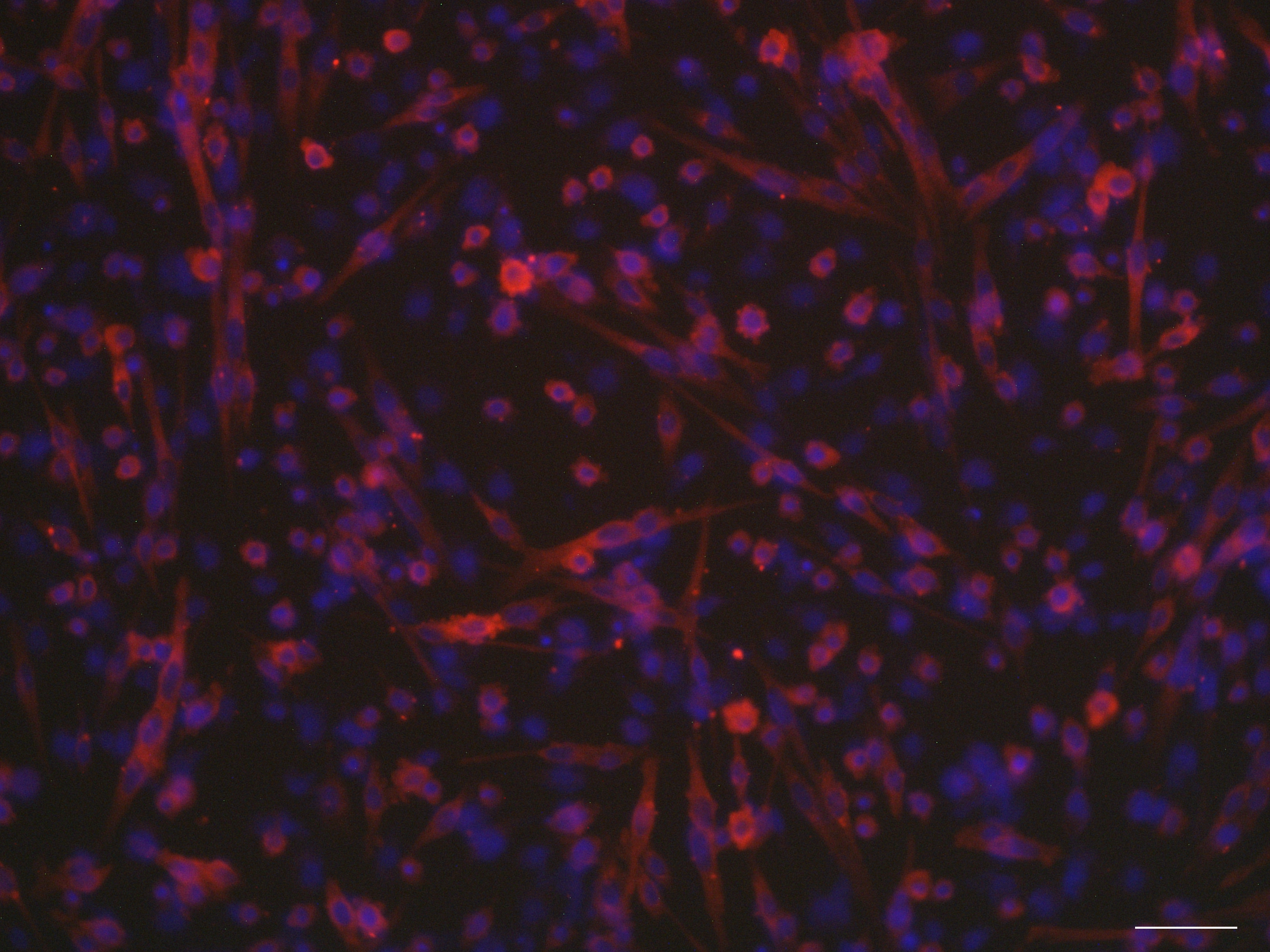

Supplement: Supplementary file 7 — Source data Fig. 4 [file 44319_2024_197_MOESM7_ESM.zip › Figure 4/4D-I/4D/IRE1 siRNA-MyHC images/24 h IRE1a siRNA replicate 2.tif]

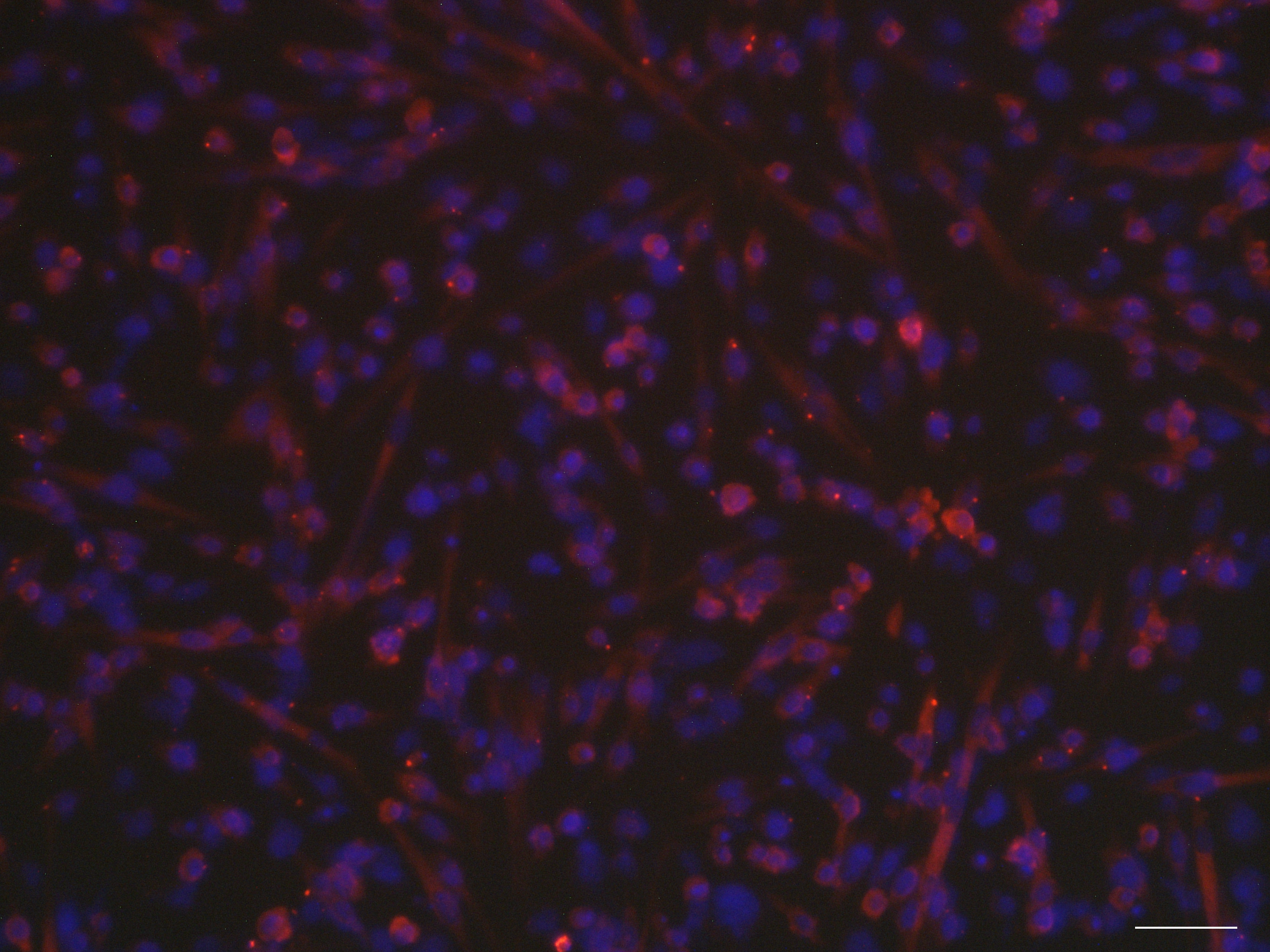

Supplement: Supplementary file 7 — Source data Fig. 4 [file 44319_2024_197_MOESM7_ESM.zip › Figure 4/4D-I/4D/IRE1 siRNA-MyHC images/24 h IRE1a siRNA replicate 3.tif]
