# Supplementary figures and images for: The IRE1α/XBP1 signaling axis drives myoblast fusion in adult skeletal muscle (part 2 of 4)
Source: EMBO Rep. 2024 Jul 9;25(8):3627–50. doi: 10.1038/s44319-024-00197-4 (PMC11316051; doi:10.1038/s44319-024-00197-4)

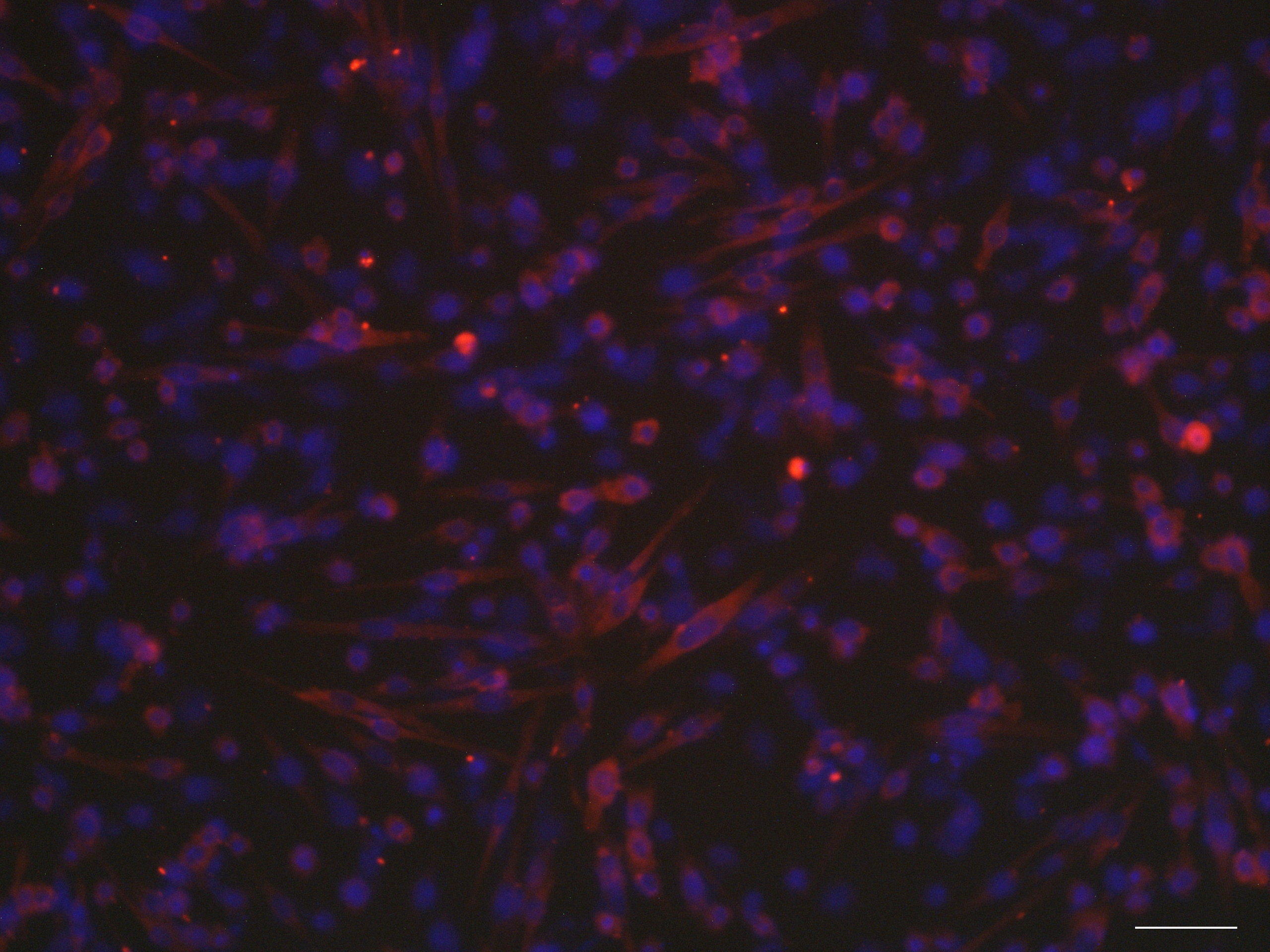

Supplement: Supplementary file 7 — Source data Fig. 4 [file 44319_2024_197_MOESM7_ESM.zip › Figure 4/4D-I/4D/IRE1 siRNA-MyHC images/24 h IRE1a siRNA Representative image.tif]

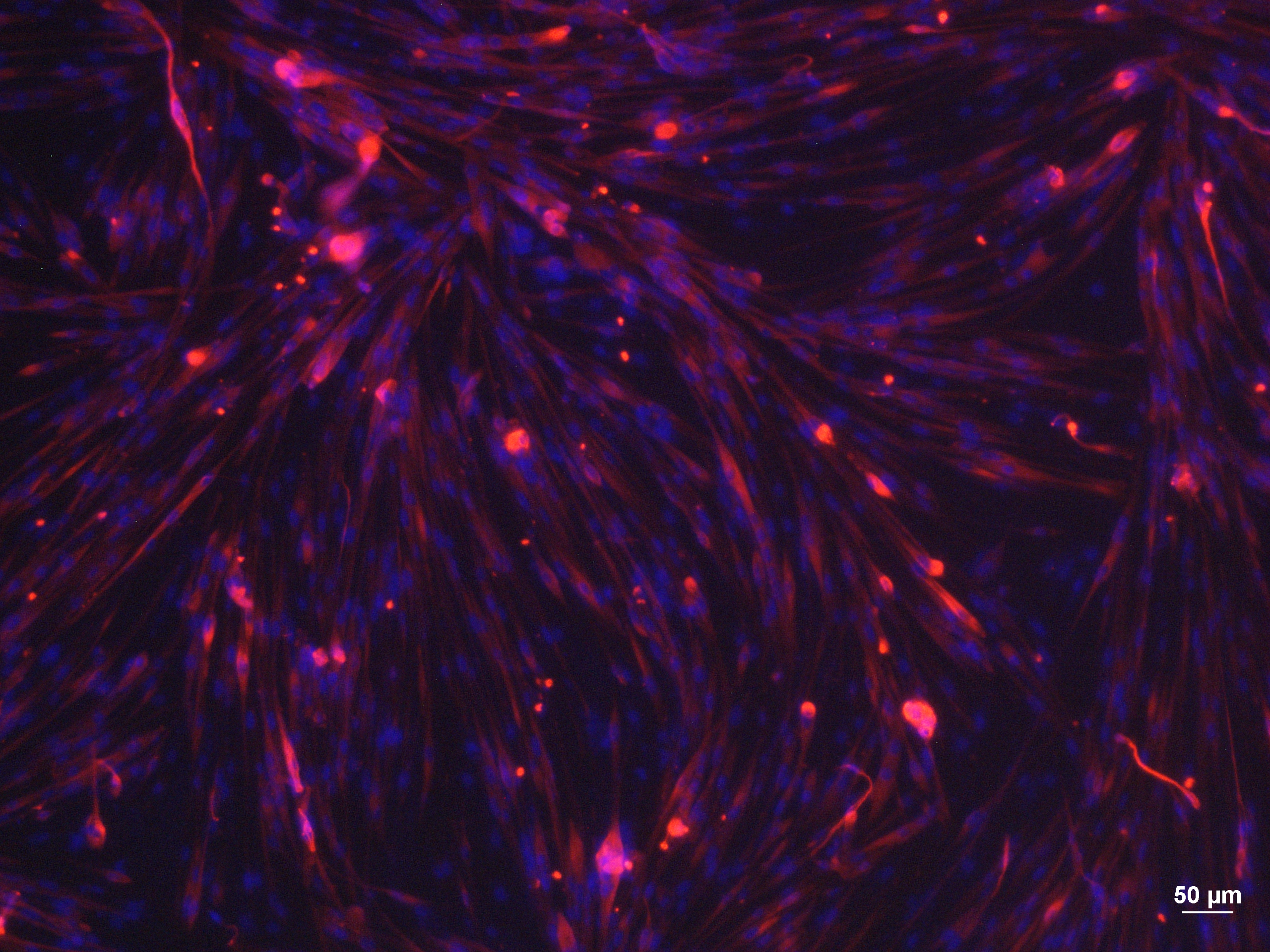

Supplement: Supplementary file 7 — Source data Fig. 4 [file 44319_2024_197_MOESM7_ESM.zip › Figure 4/4D-I/4D/IRE1 siRNA-MyHC images/48 h Con siRNA replicate 2.tif]

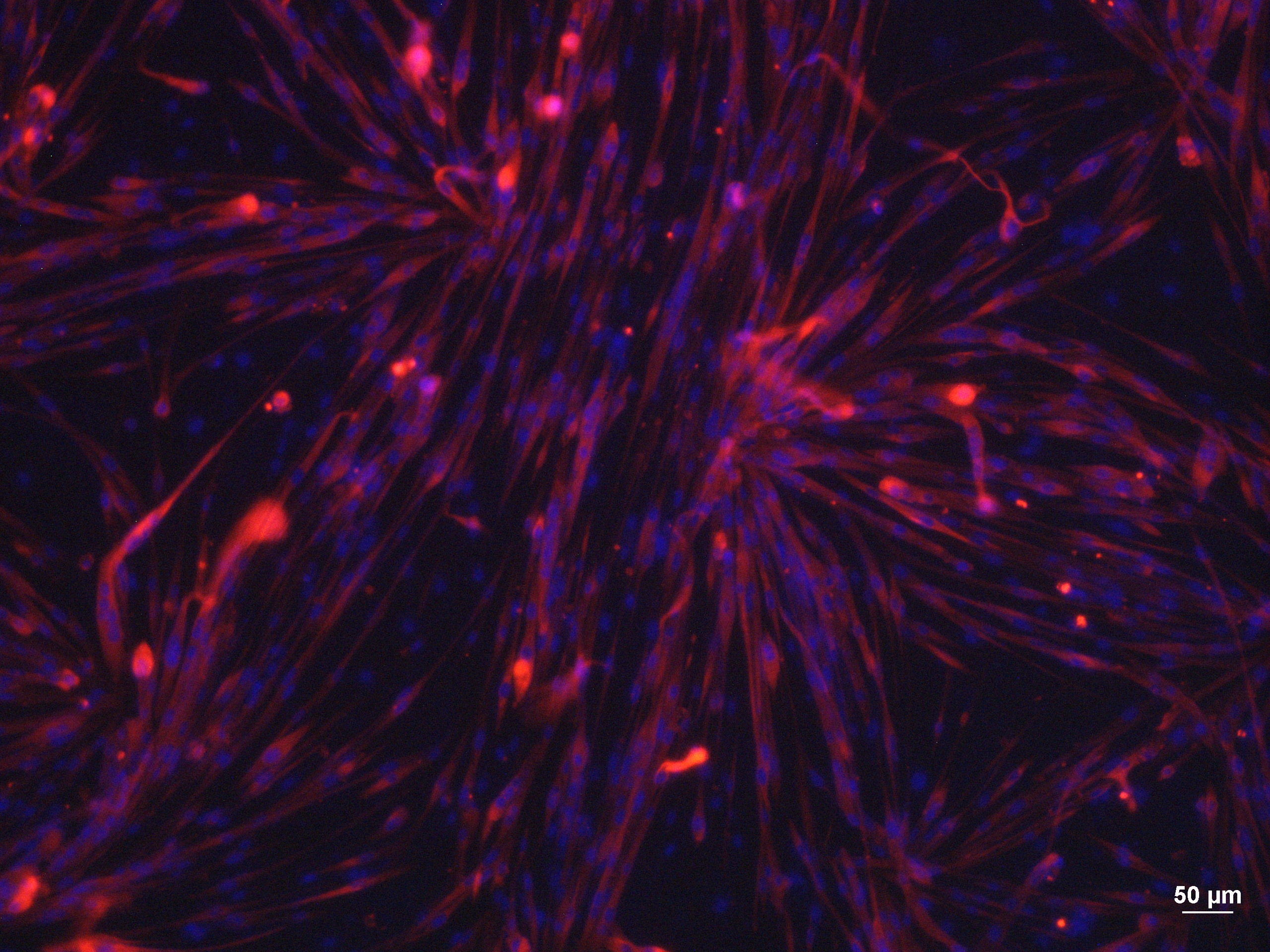

Supplement: Supplementary file 7 — Source data Fig. 4 [file 44319_2024_197_MOESM7_ESM.zip › Figure 4/4D-I/4D/IRE1 siRNA-MyHC images/48 h Con siRNA replicate 3.tif]

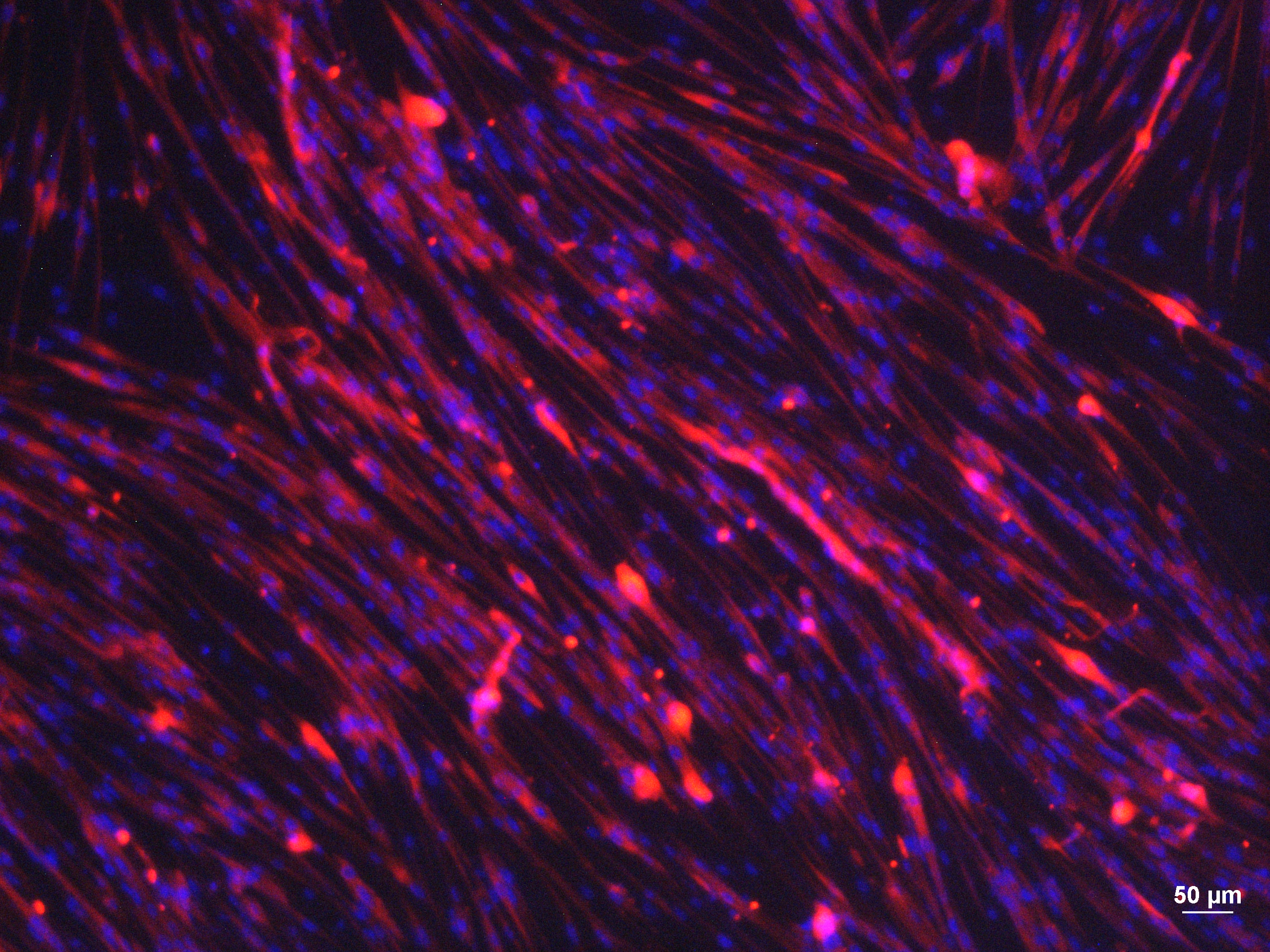

Supplement: Supplementary file 7 — Source data Fig. 4 [file 44319_2024_197_MOESM7_ESM.zip › Figure 4/4D-I/4D/IRE1 siRNA-MyHC images/48 h Con siRNA Representative image.tif]

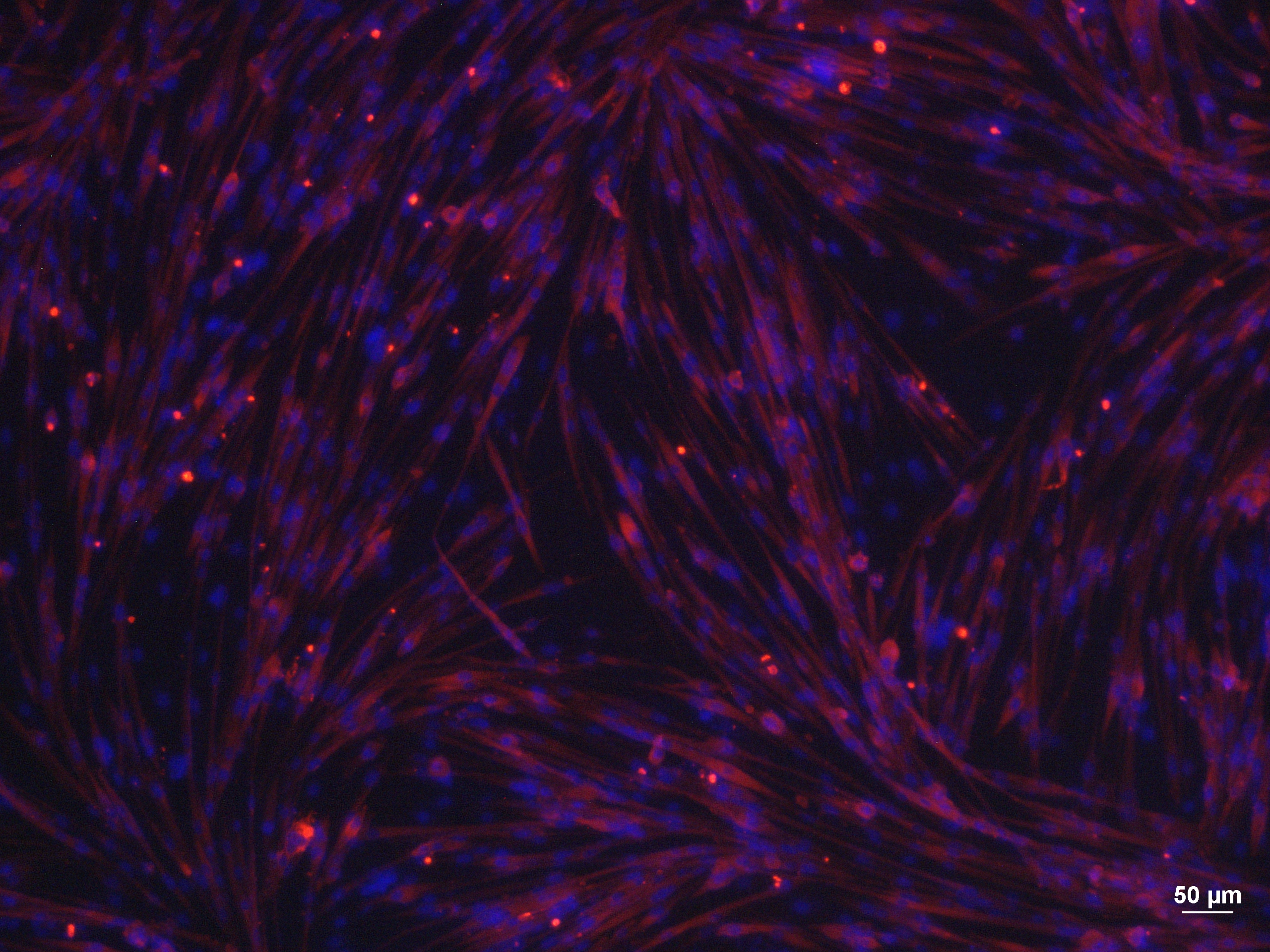

Supplement: Supplementary file 7 — Source data Fig. 4 [file 44319_2024_197_MOESM7_ESM.zip › Figure 4/4D-I/4D/IRE1 siRNA-MyHC images/48 h IRE1a siRNA replicate 2.tif]

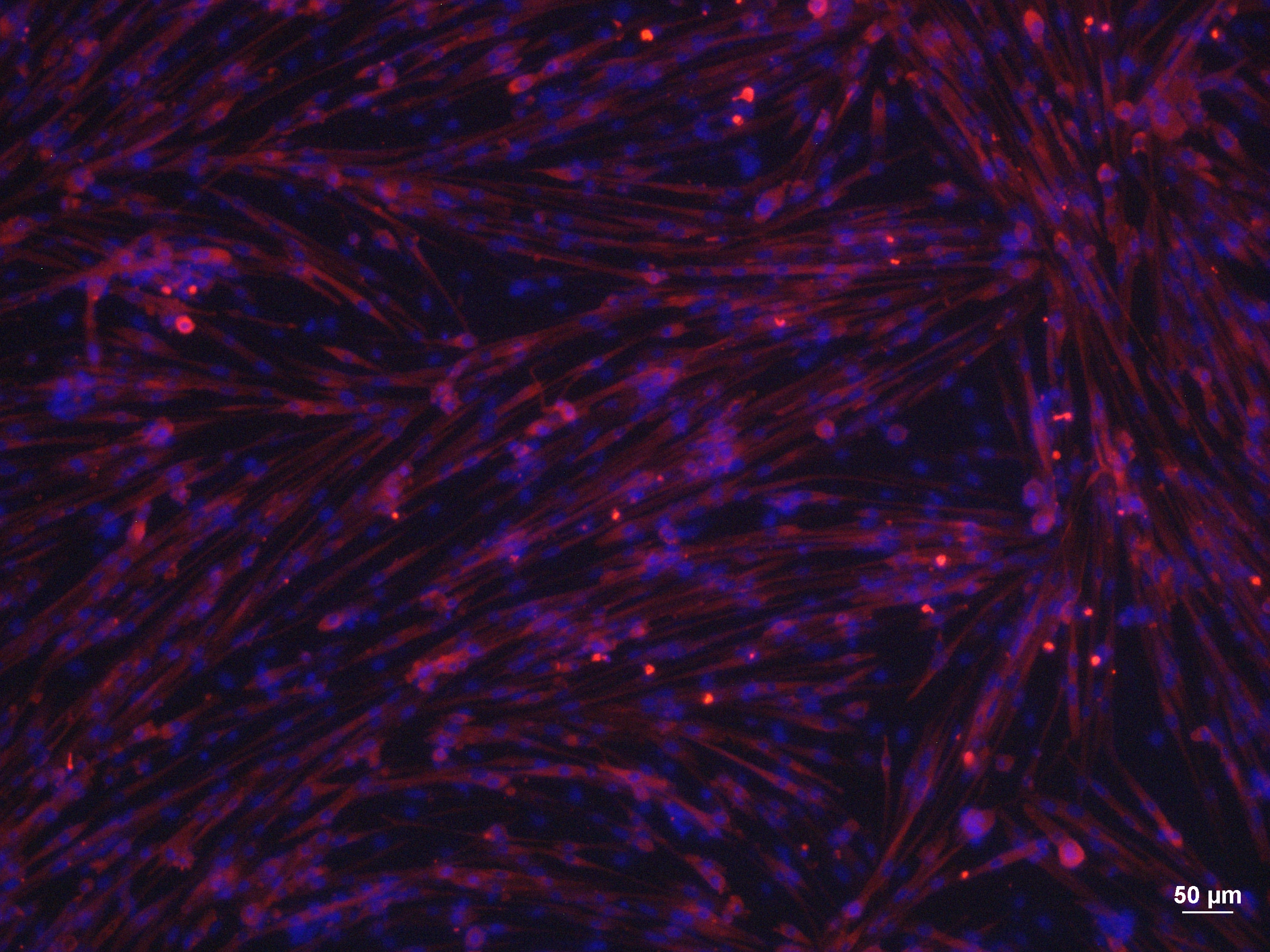

Supplement: Supplementary file 7 — Source data Fig. 4 [file 44319_2024_197_MOESM7_ESM.zip › Figure 4/4D-I/4D/IRE1 siRNA-MyHC images/48 h IRE1a siRNA replicate 3.tif]

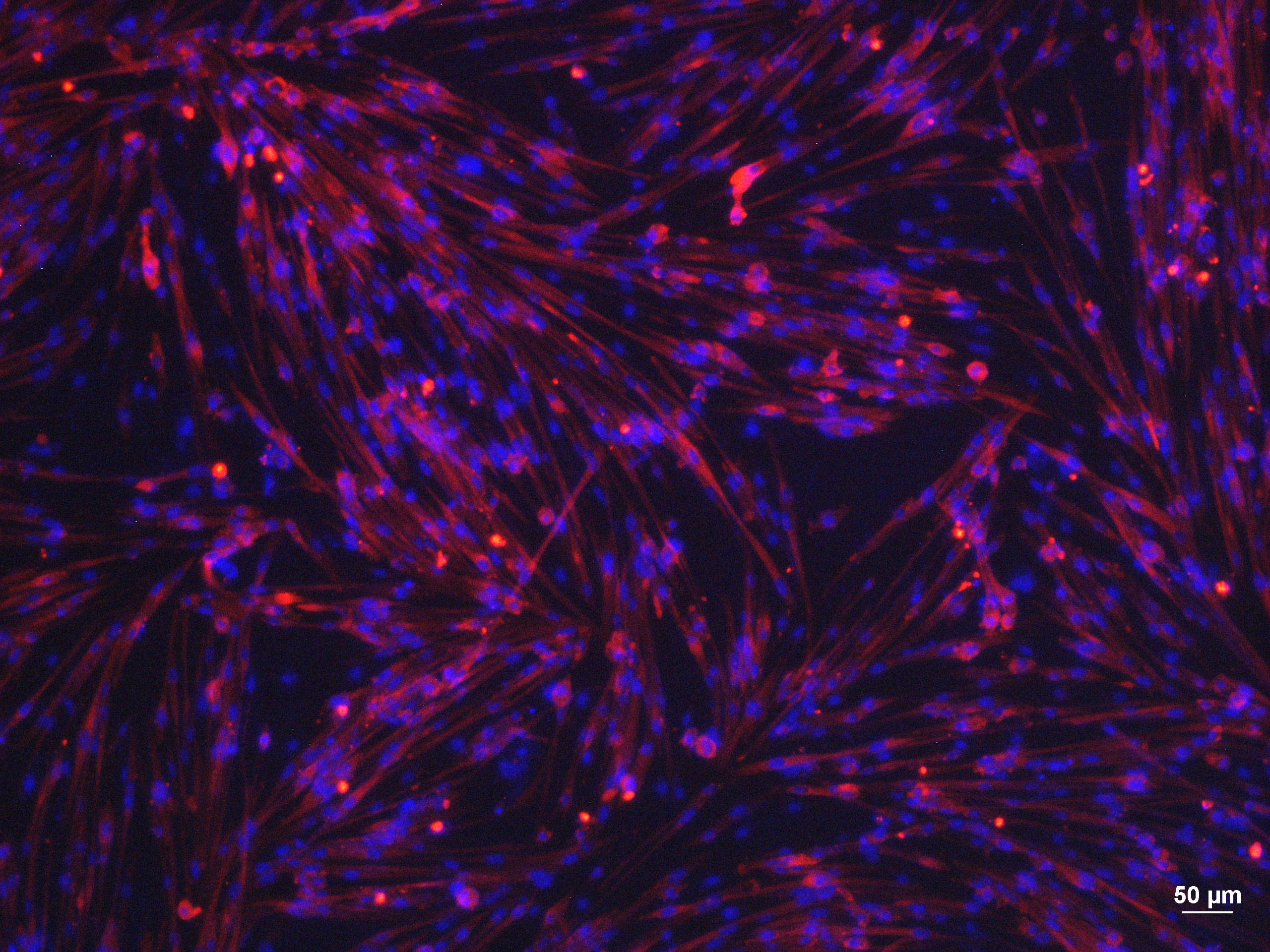

Supplement: Supplementary file 7 — Source data Fig. 4 [file 44319_2024_197_MOESM7_ESM.zip › Figure 4/4D-I/4D/IRE1 siRNA-MyHC images/48 h IRE1a siRNA Representative image.tif]

## Slide 1
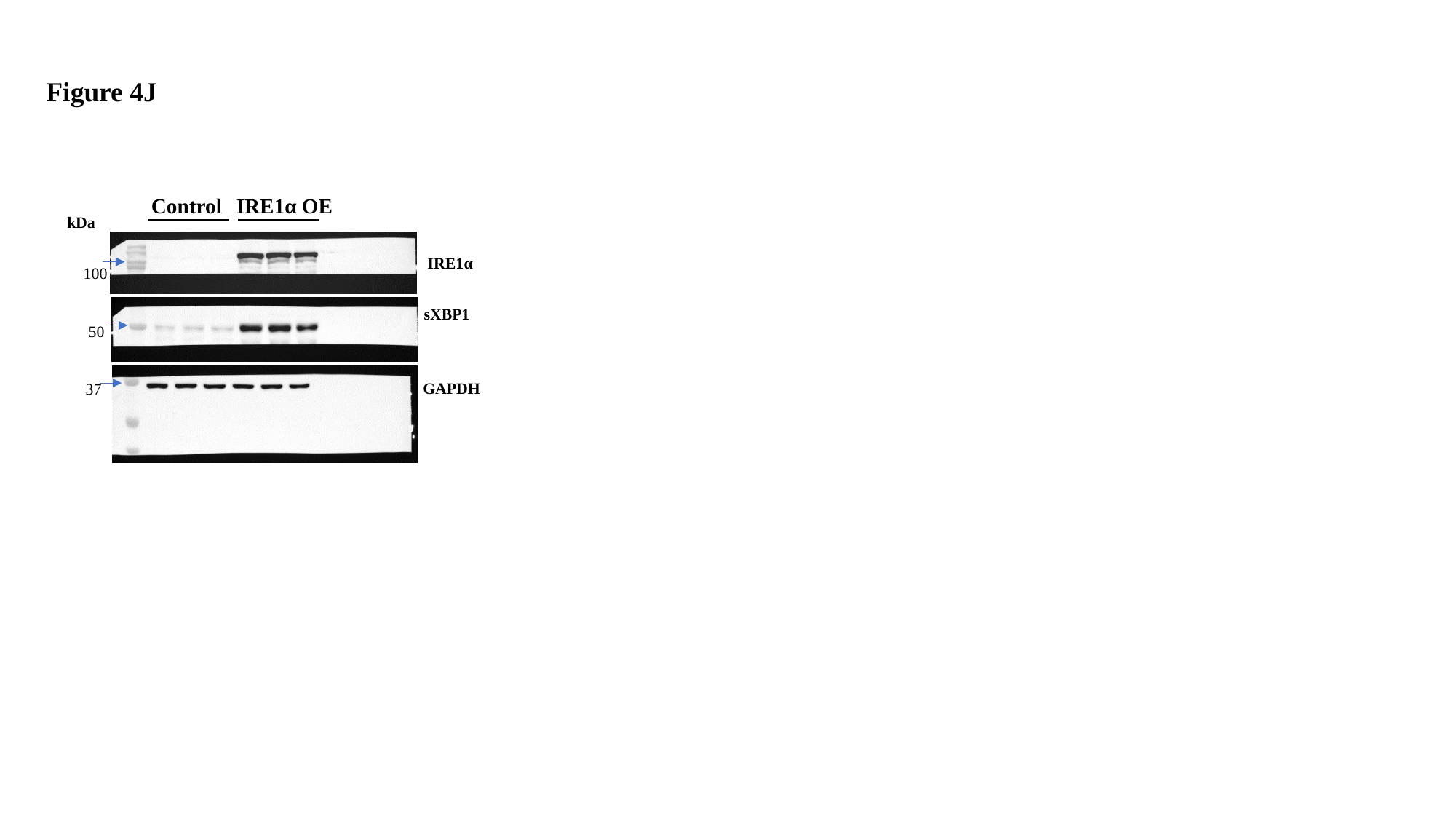

Figure 4J
Control
IRE1α OE
kDa
IRE1α
100
sXBP1
50
GAPDH
37

Supplement: Supplementary file 7 — Source data Fig. 4 [file 44319_2024_197_MOESM7_ESM.zip › Figure 4/4J-M/4J/Western blot with annotation.pptx]

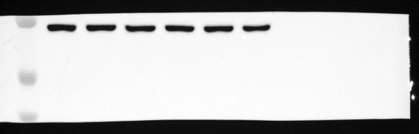

Supplement: Supplementary file 7 — Source data Fig. 4 [file 44319_2024_197_MOESM7_ESM.zip › Figure 4/4J-M/4J/Western-GAPDH.tif]

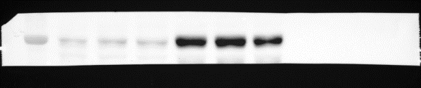

Supplement: Supplementary file 7 — Source data Fig. 4 [file 44319_2024_197_MOESM7_ESM.zip › Figure 4/4J-M/4J/Western-sXBP1.tif]

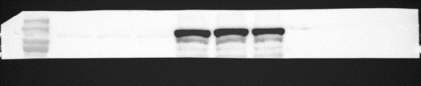

Supplement: Supplementary file 7 — Source data Fig. 4 [file 44319_2024_197_MOESM7_ESM.zip › Figure 4/4J-M/4J/Western-Total IRE1a.tif]

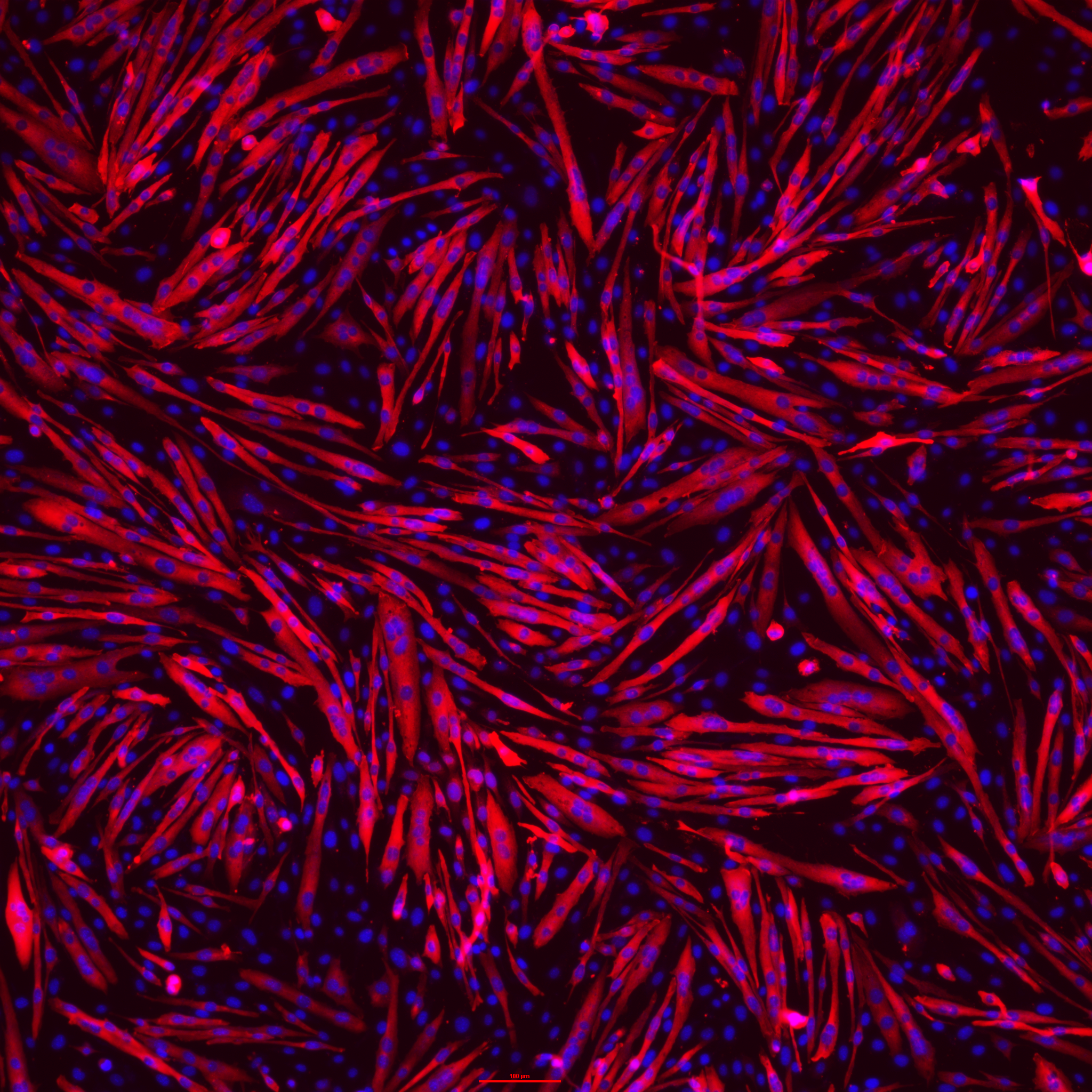

Supplement: Supplementary file 7 — Source data Fig. 4 [file 44319_2024_197_MOESM7_ESM.zip › Figure 4/4J-M/4K/IRE1 OE-MyHC images/24 h Control replicate 2.tif]

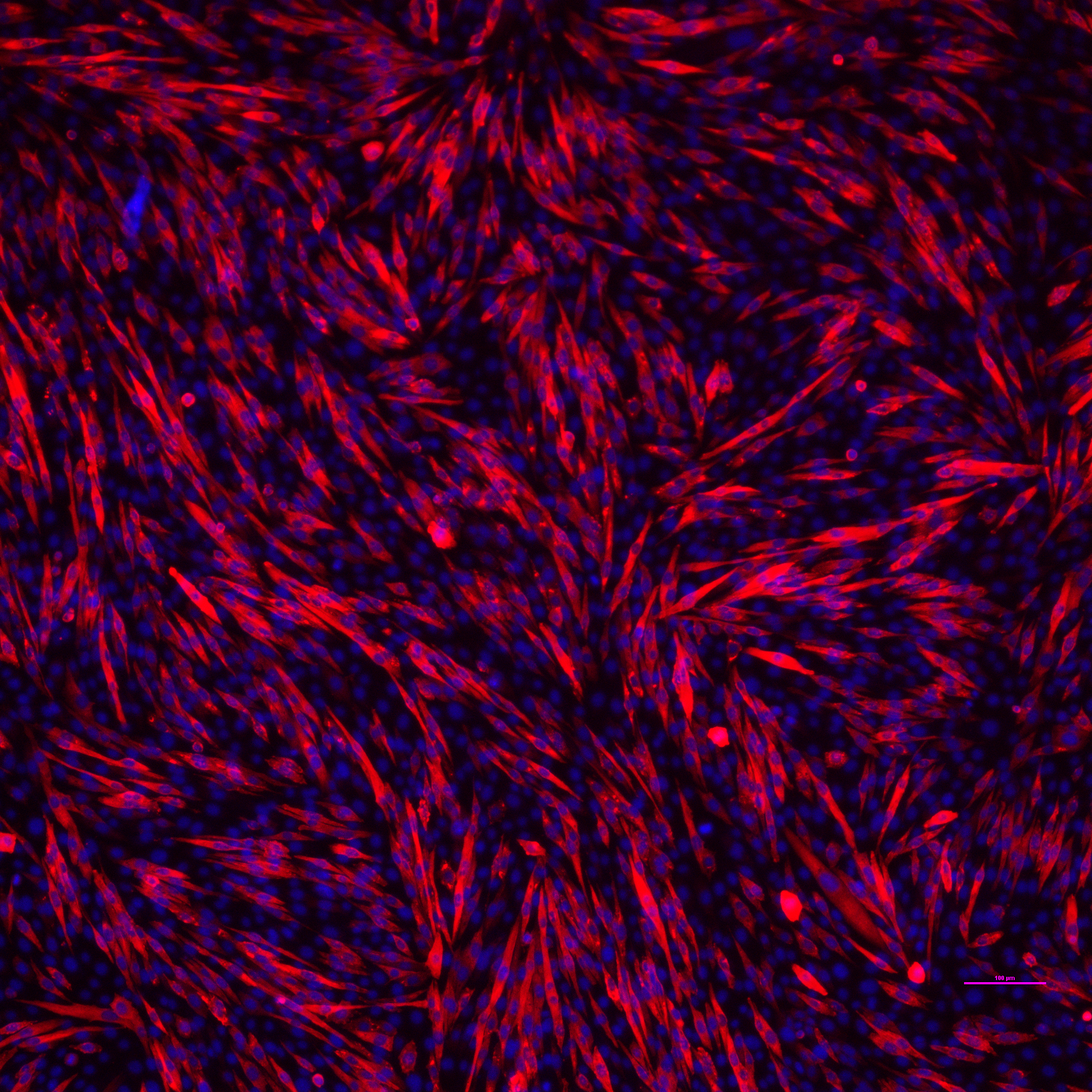

Supplement: Supplementary file 7 — Source data Fig. 4 [file 44319_2024_197_MOESM7_ESM.zip › Figure 4/4J-M/4K/IRE1 OE-MyHC images/24 h Control replicate 3.tif]

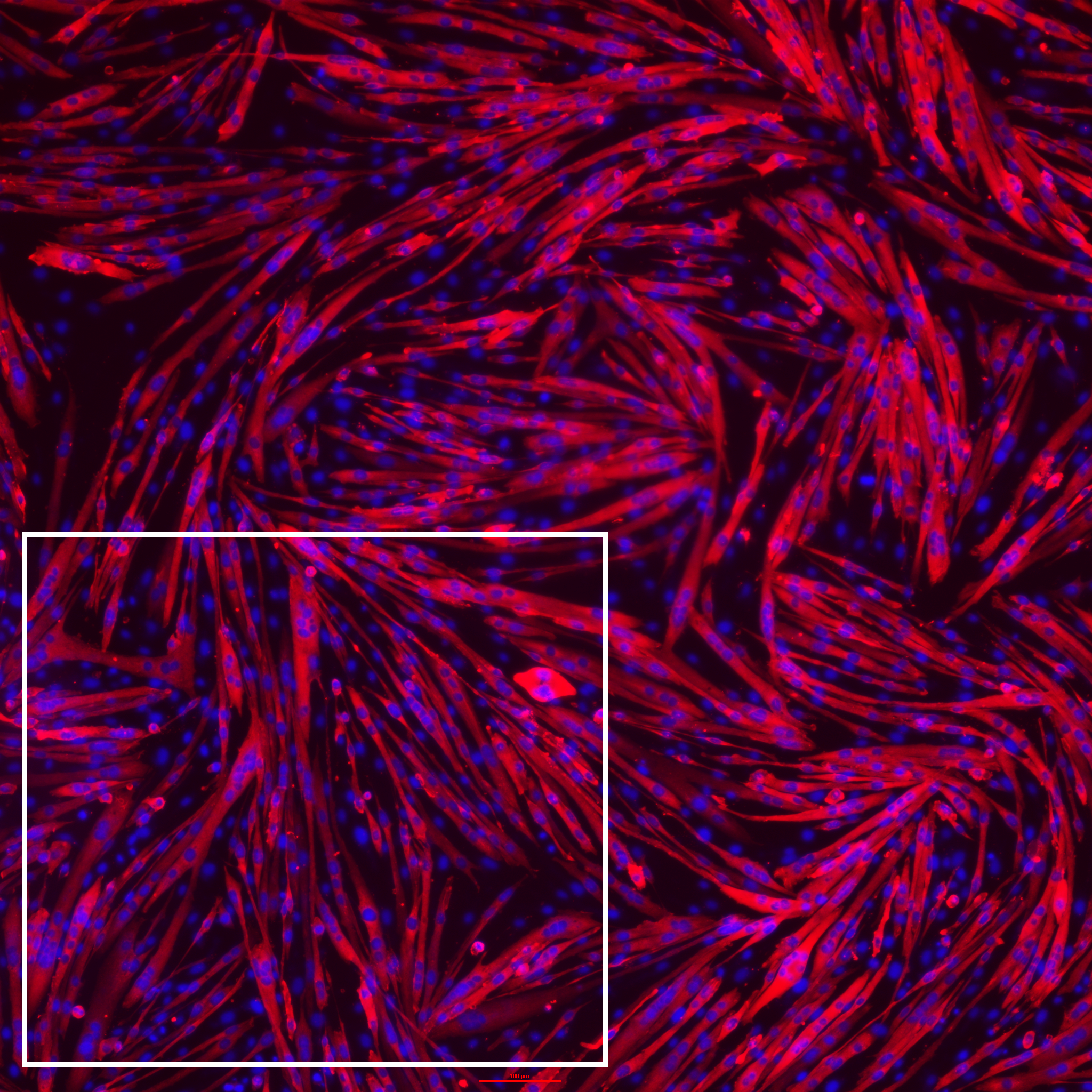

Supplement: Supplementary file 7 — Source data Fig. 4 [file 44319_2024_197_MOESM7_ESM.zip › Figure 4/4J-M/4K/IRE1 OE-MyHC images/24 h Control Representative image with box.tif]

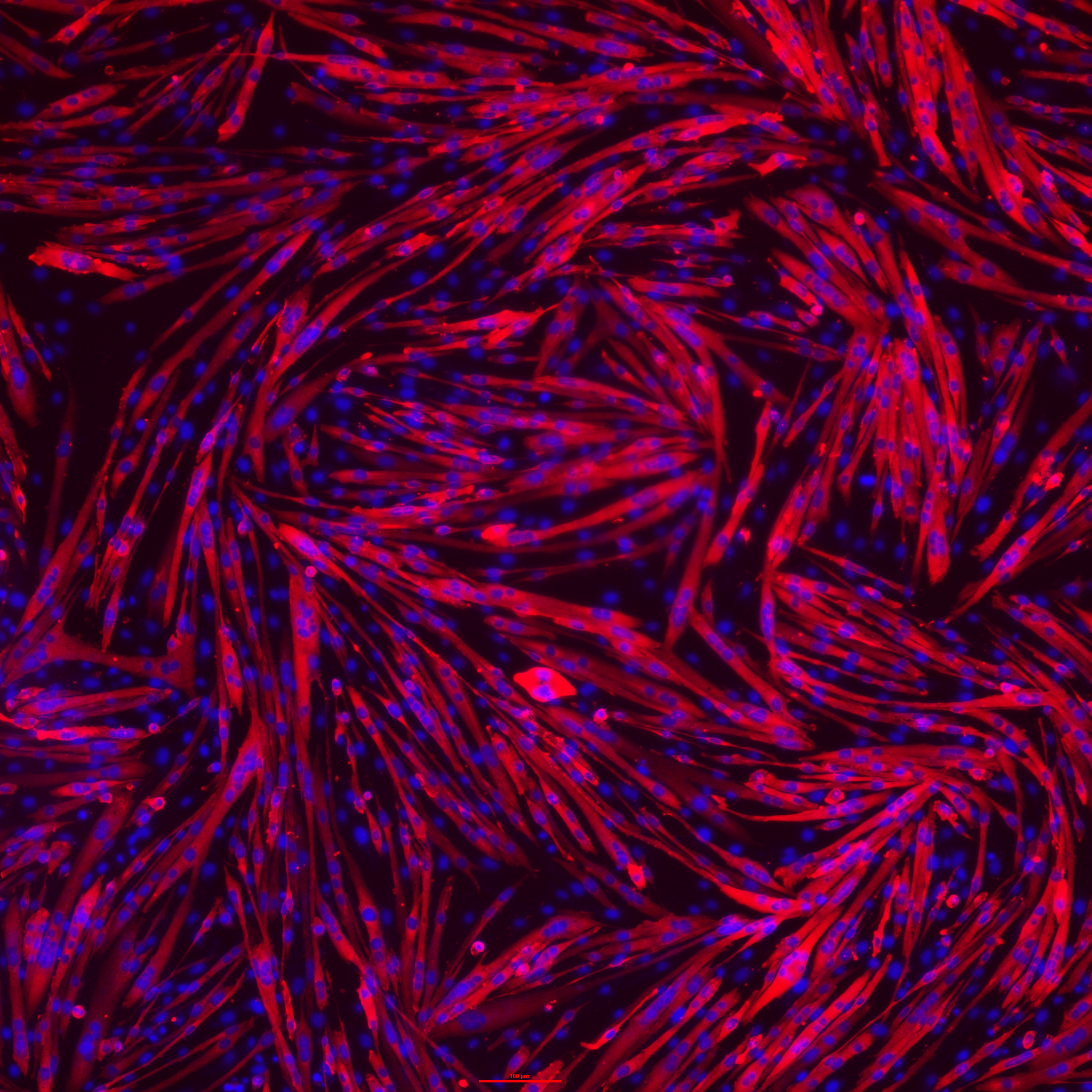

Supplement: Supplementary file 7 — Source data Fig. 4 [file 44319_2024_197_MOESM7_ESM.zip › Figure 4/4J-M/4K/IRE1 OE-MyHC images/24 h Control Representative image.tif]

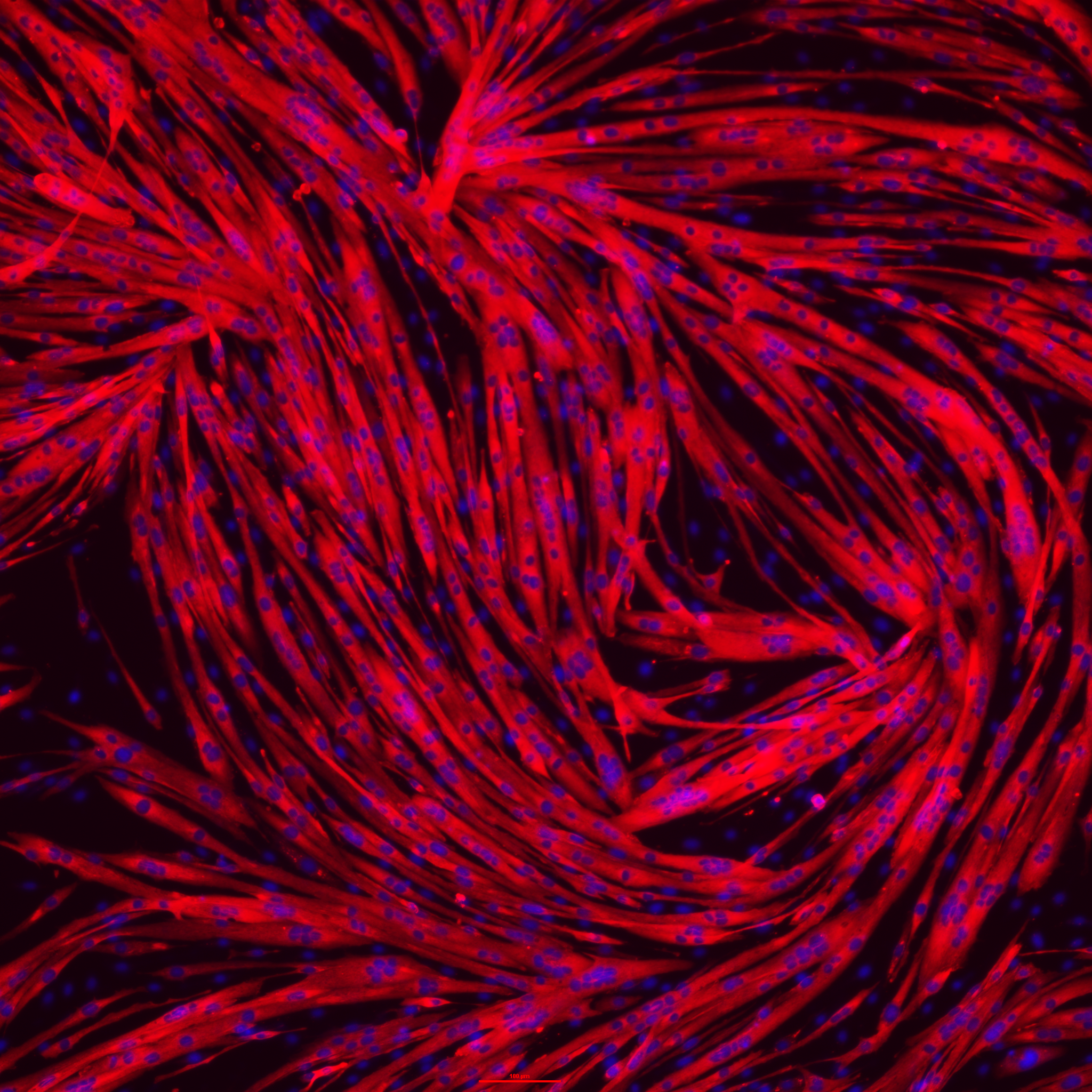

Supplement: Supplementary file 7 — Source data Fig. 4 [file 44319_2024_197_MOESM7_ESM.zip › Figure 4/4J-M/4K/IRE1 OE-MyHC images/24 h IRE1a OE -replicate 2.tif]

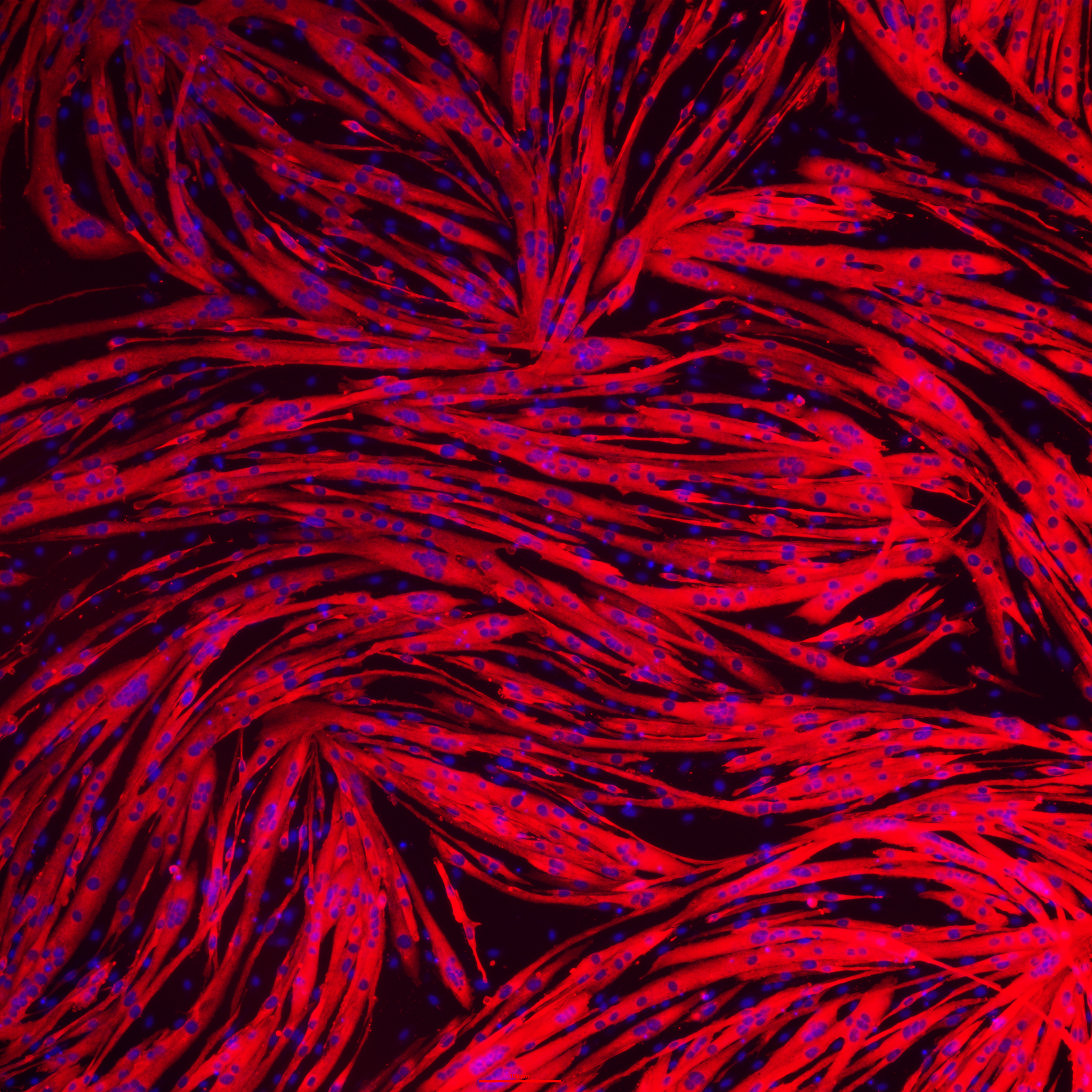

Supplement: Supplementary file 7 — Source data Fig. 4 [file 44319_2024_197_MOESM7_ESM.zip › Figure 4/4J-M/4K/IRE1 OE-MyHC images/24 h IRE1a OE -replicate 3.tif]

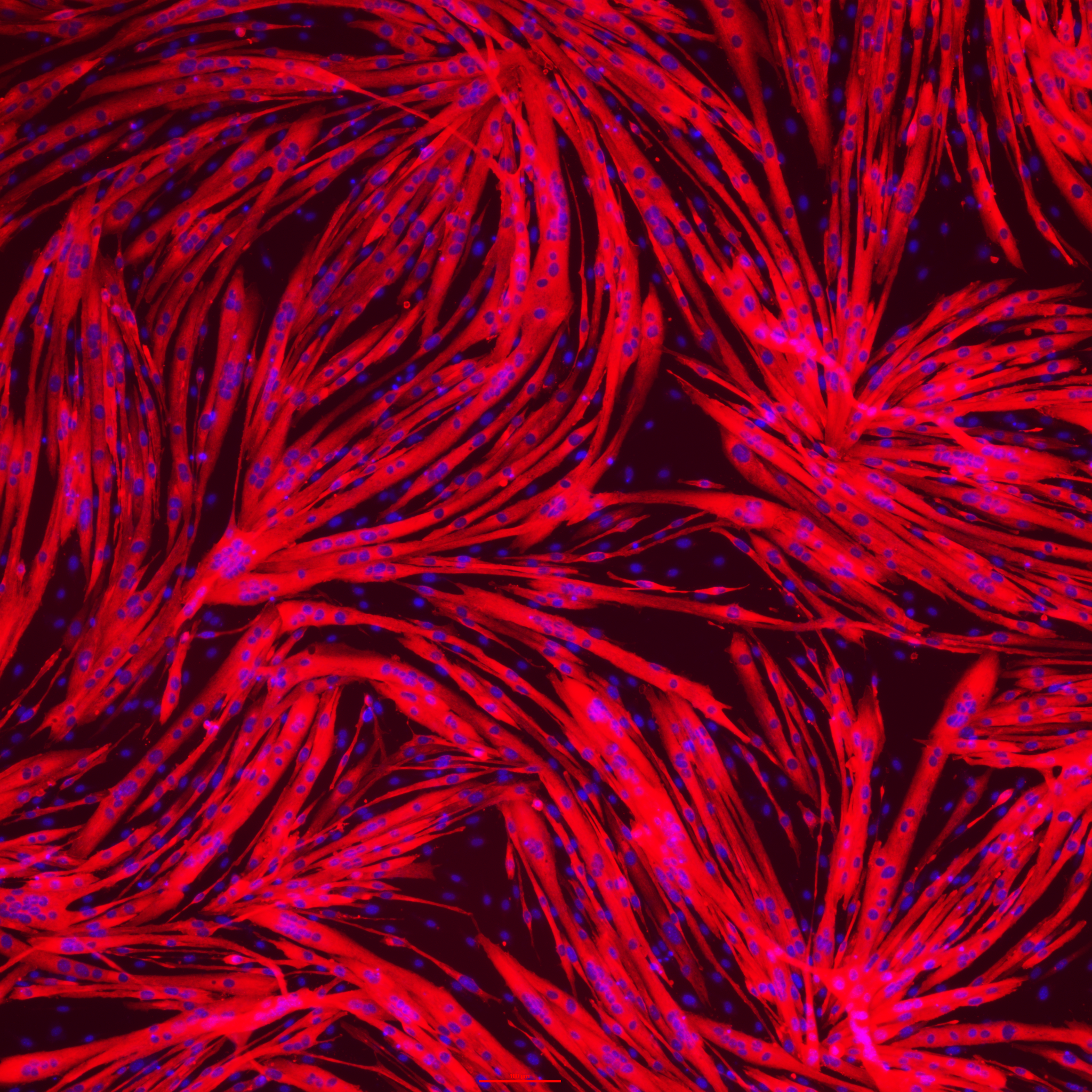

Supplement: Supplementary file 7 — Source data Fig. 4 [file 44319_2024_197_MOESM7_ESM.zip › Figure 4/4J-M/4K/IRE1 OE-MyHC images/24 h IRE1a OE -Representative image.tif]

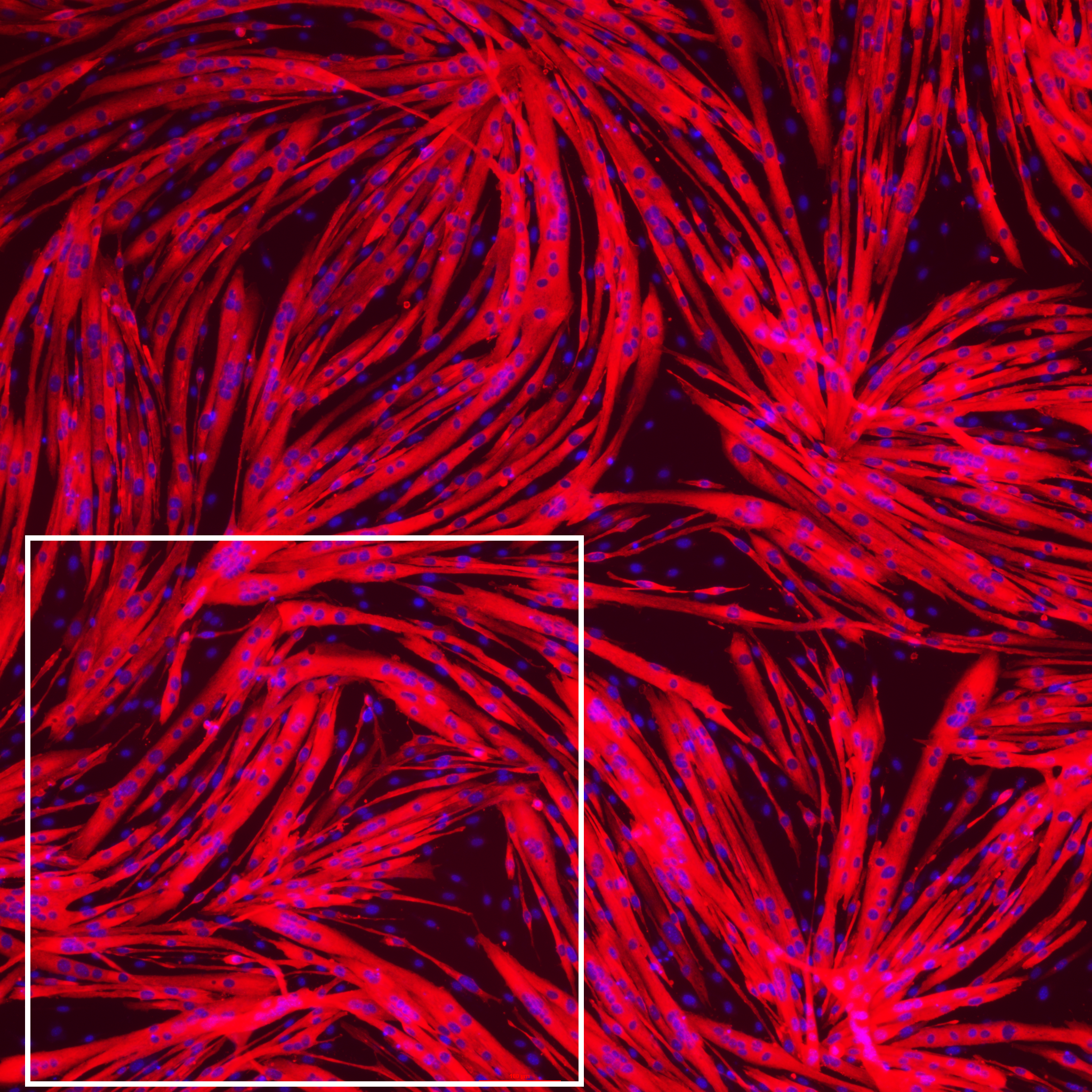

Supplement: Supplementary file 7 — Source data Fig. 4 [file 44319_2024_197_MOESM7_ESM.zip › Figure 4/4J-M/4K/IRE1 OE-MyHC images/24 h IRE1a OE Repsentative image with box.tif]

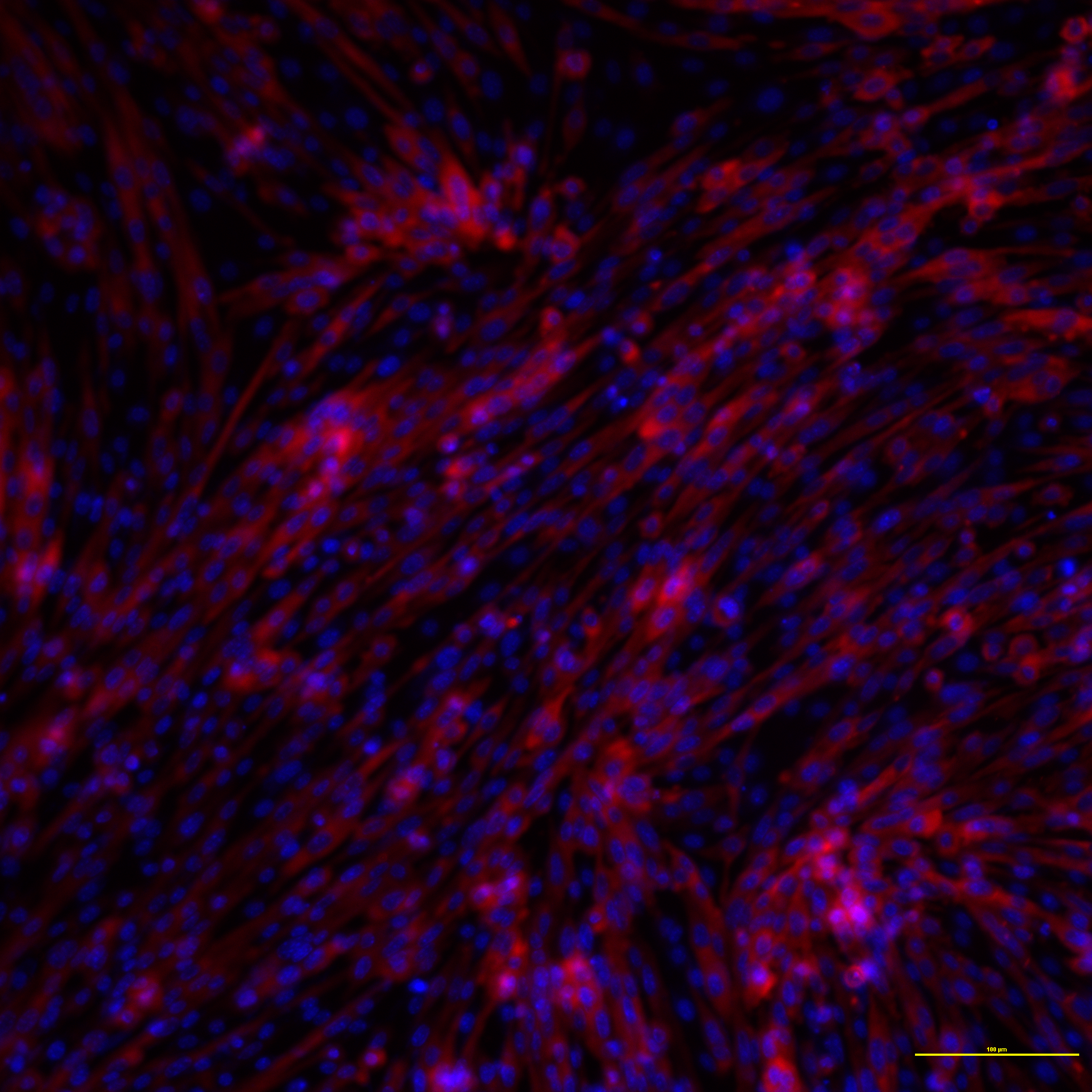

Supplement: Supplementary file 9 — Source data Fig. 6 [file 44319_2024_197_MOESM9_ESM.zip › Figure 6/6A-F/6A/XBP1 siRNA-MyHC images/24 h Control siRNA replicate 2.tif]

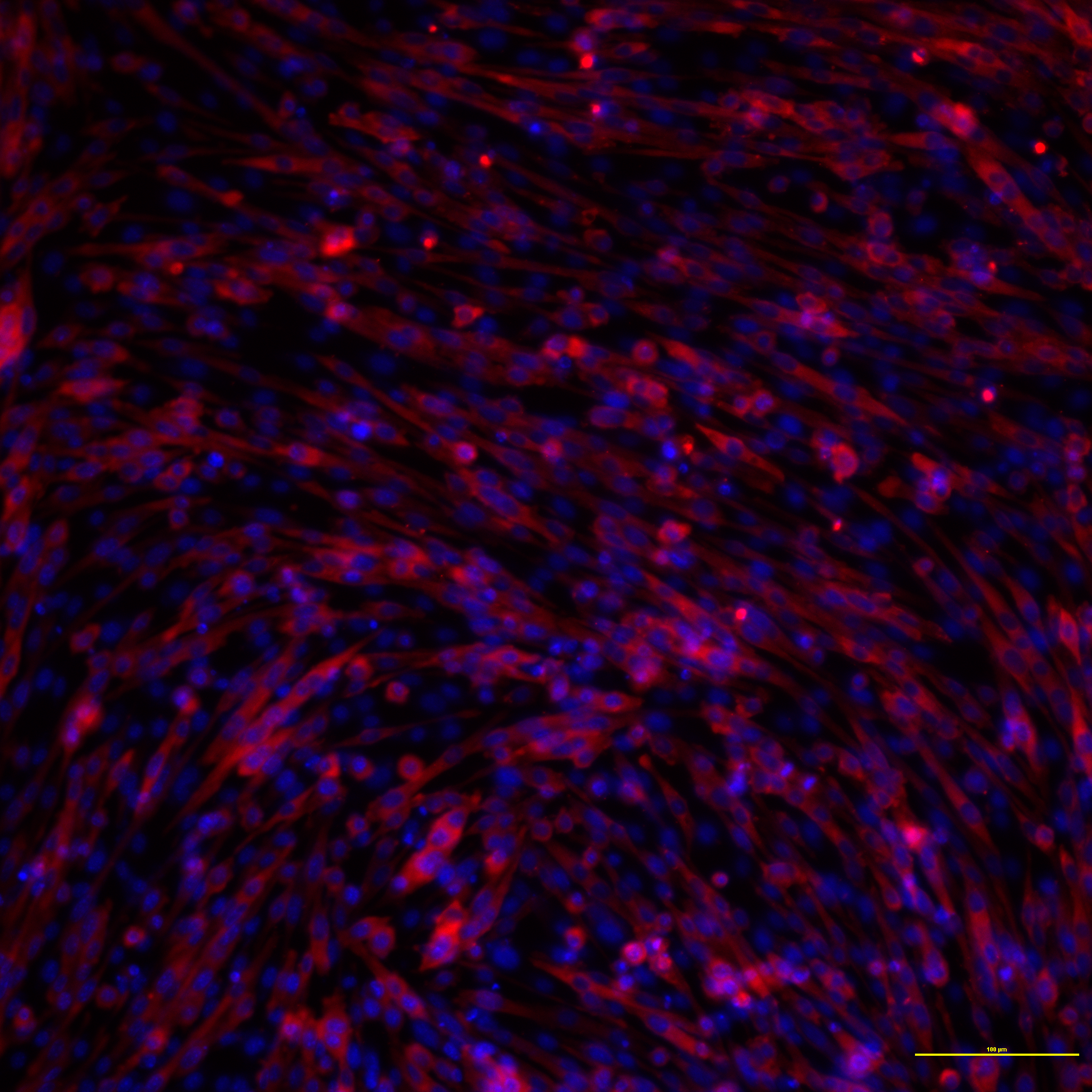

Supplement: Supplementary file 9 — Source data Fig. 6 [file 44319_2024_197_MOESM9_ESM.zip › Figure 6/6A-F/6A/XBP1 siRNA-MyHC images/24 h Control siRNA replicate 3.tif]

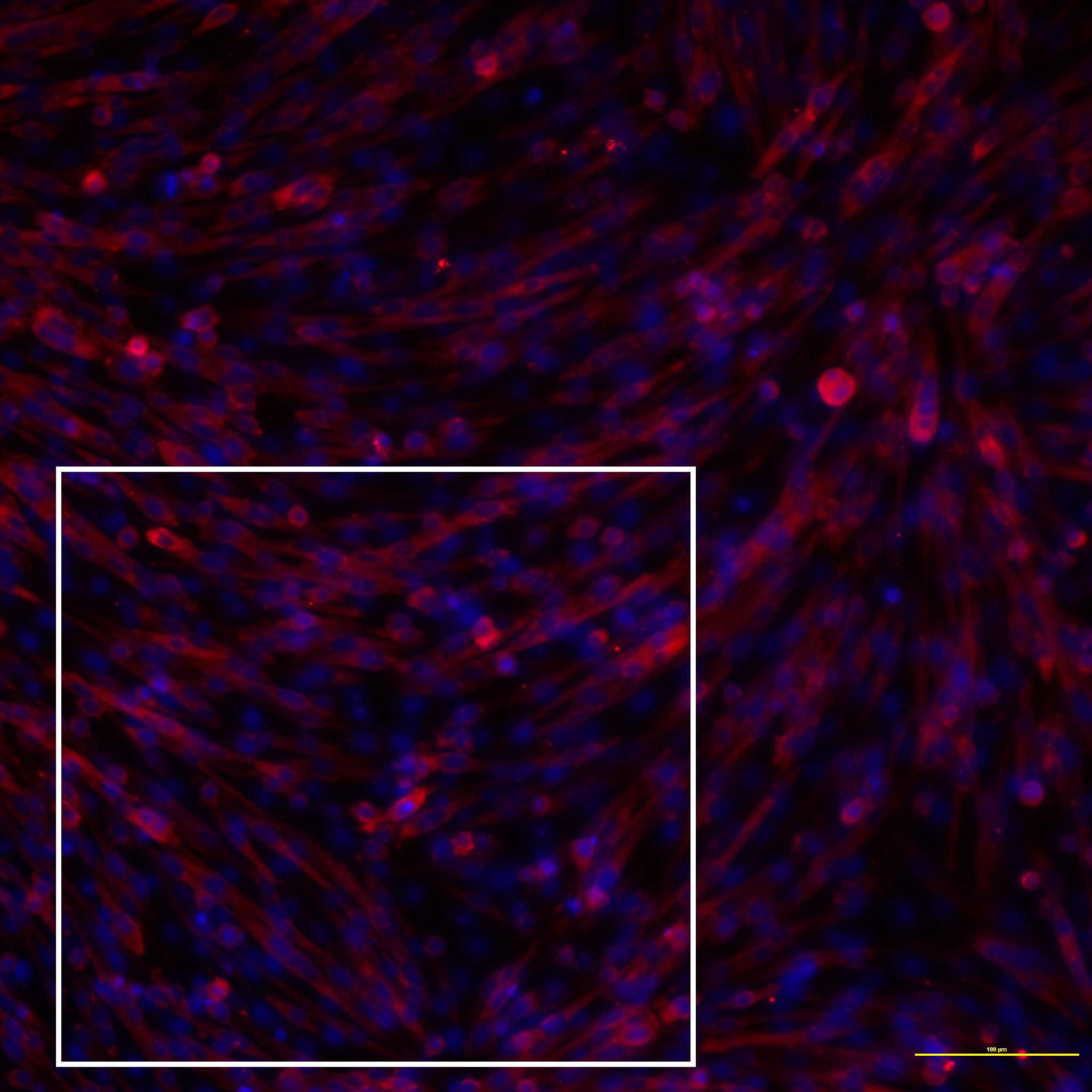

Supplement: Supplementary file 9 — Source data Fig. 6 [file 44319_2024_197_MOESM9_ESM.zip › Figure 6/6A-F/6A/XBP1 siRNA-MyHC images/24 h Control siRNA Representative image with box.tif]

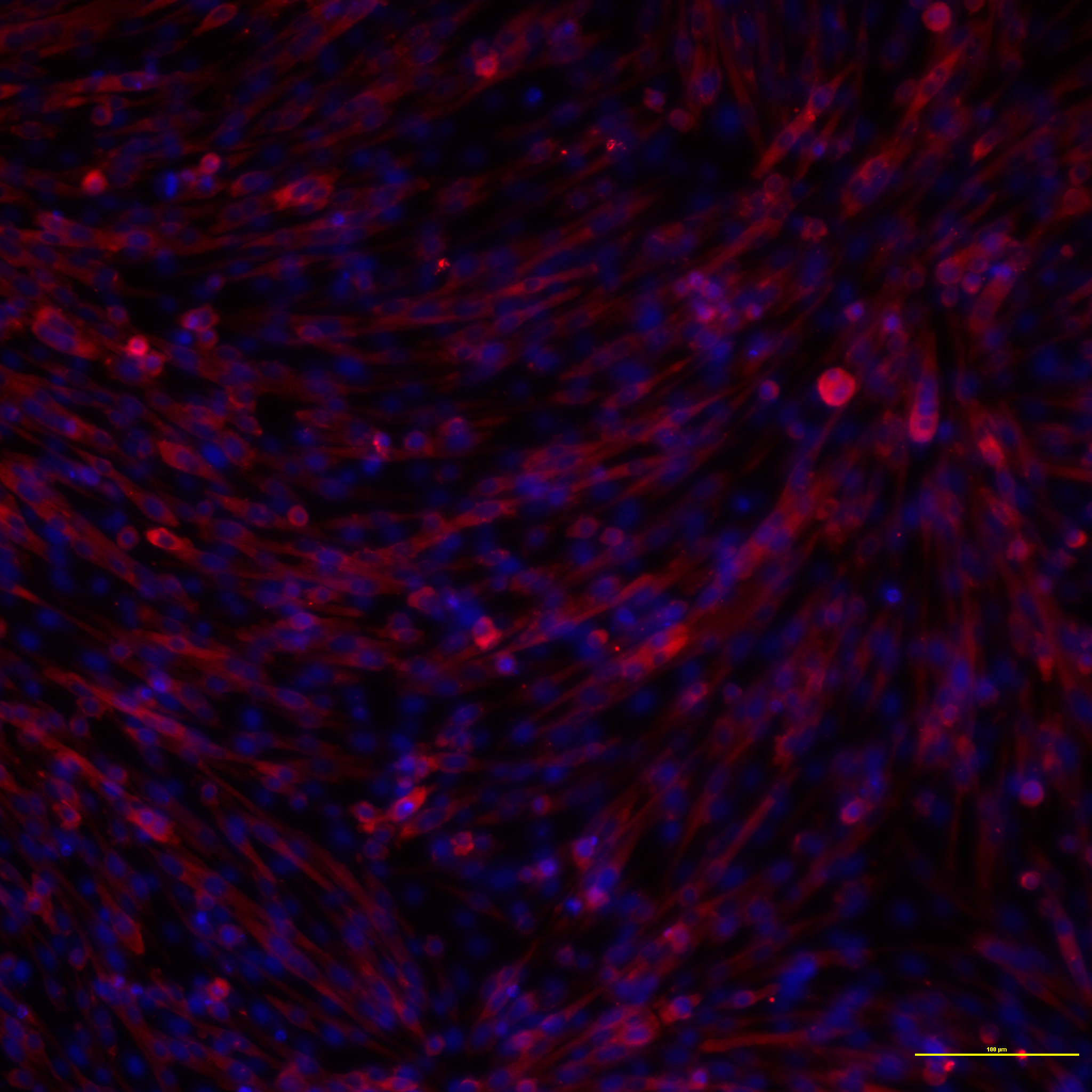

Supplement: Supplementary file 9 — Source data Fig. 6 [file 44319_2024_197_MOESM9_ESM.zip › Figure 6/6A-F/6A/XBP1 siRNA-MyHC images/24 h Control siRNA Representative image.tif]

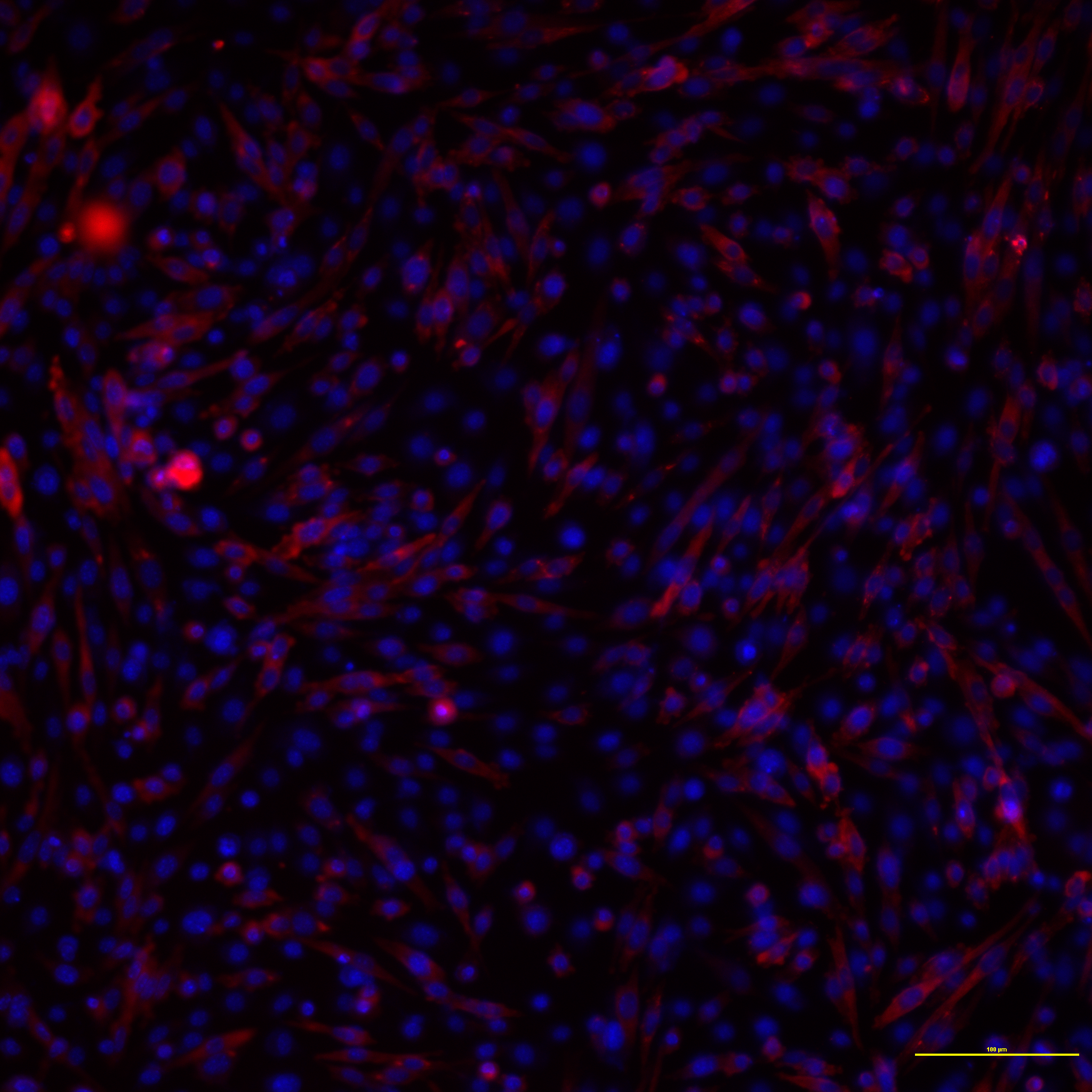

Supplement: Supplementary file 9 — Source data Fig. 6 [file 44319_2024_197_MOESM9_ESM.zip › Figure 6/6A-F/6A/XBP1 siRNA-MyHC images/24 h XBP1 siRNA replicate 2.tif]

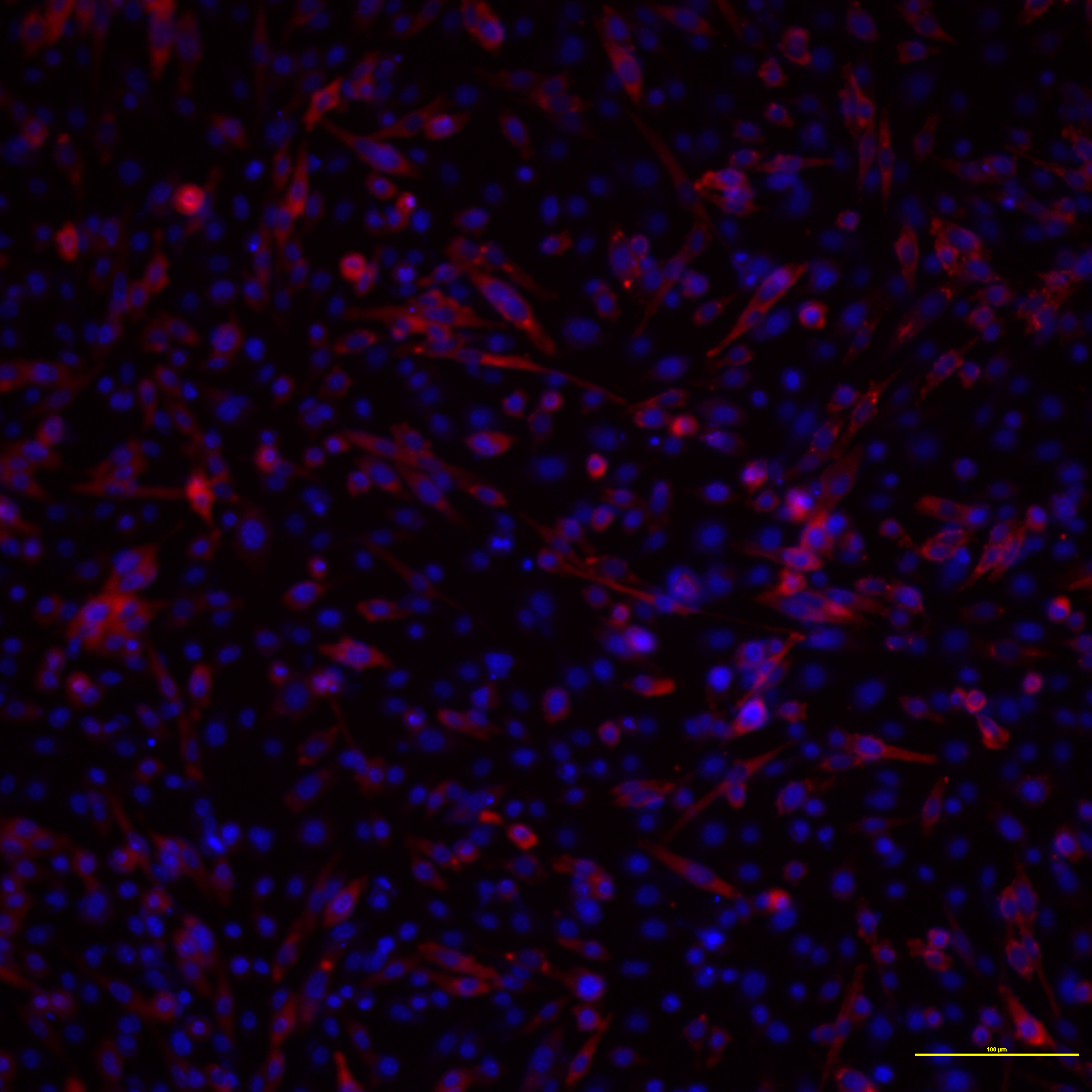

Supplement: Supplementary file 9 — Source data Fig. 6 [file 44319_2024_197_MOESM9_ESM.zip › Figure 6/6A-F/6A/XBP1 siRNA-MyHC images/24 h XBP1 siRNA replicate 3.tif]

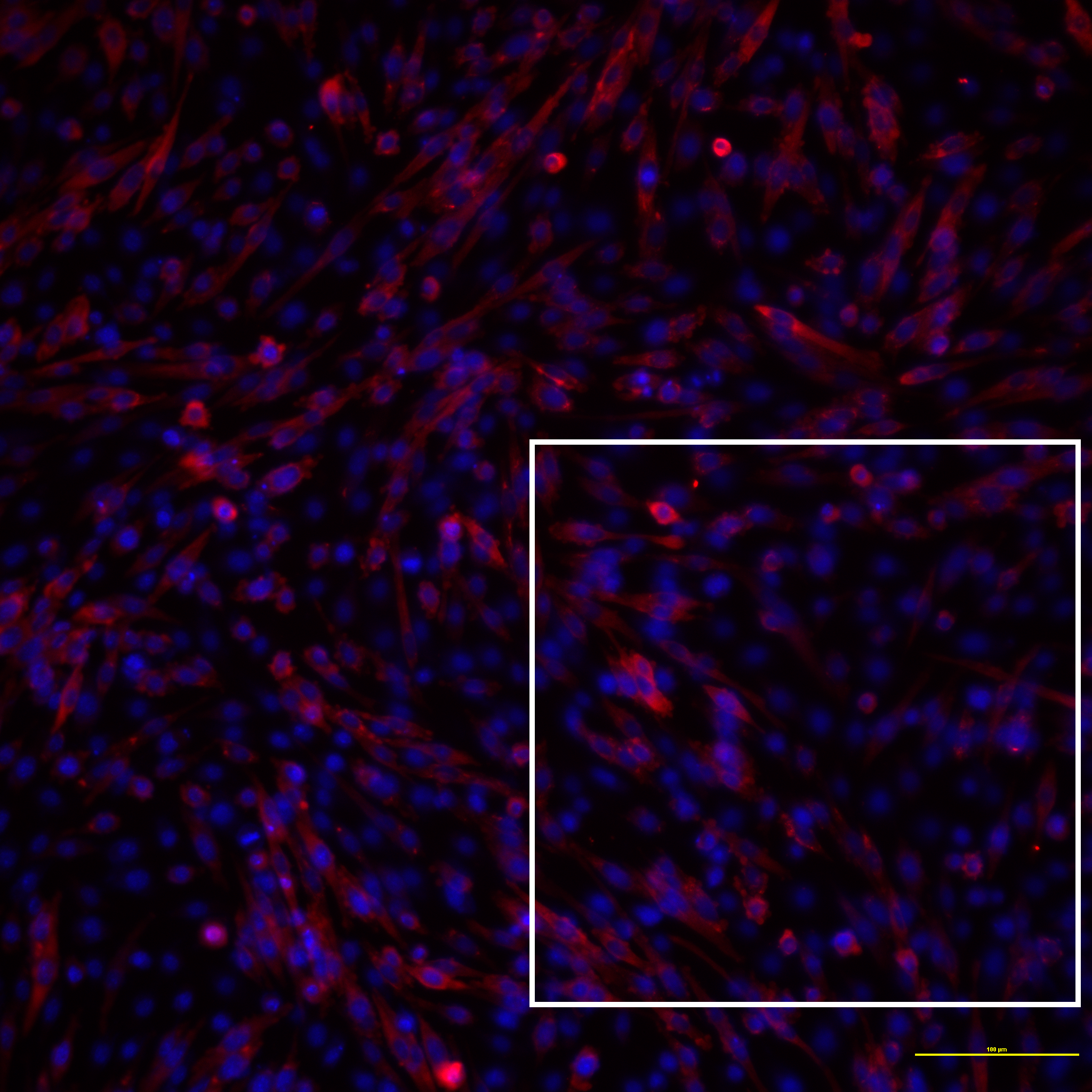

Supplement: Supplementary file 9 — Source data Fig. 6 [file 44319_2024_197_MOESM9_ESM.zip › Figure 6/6A-F/6A/XBP1 siRNA-MyHC images/24 h XBP1 siRNA Representative image with box.tif]

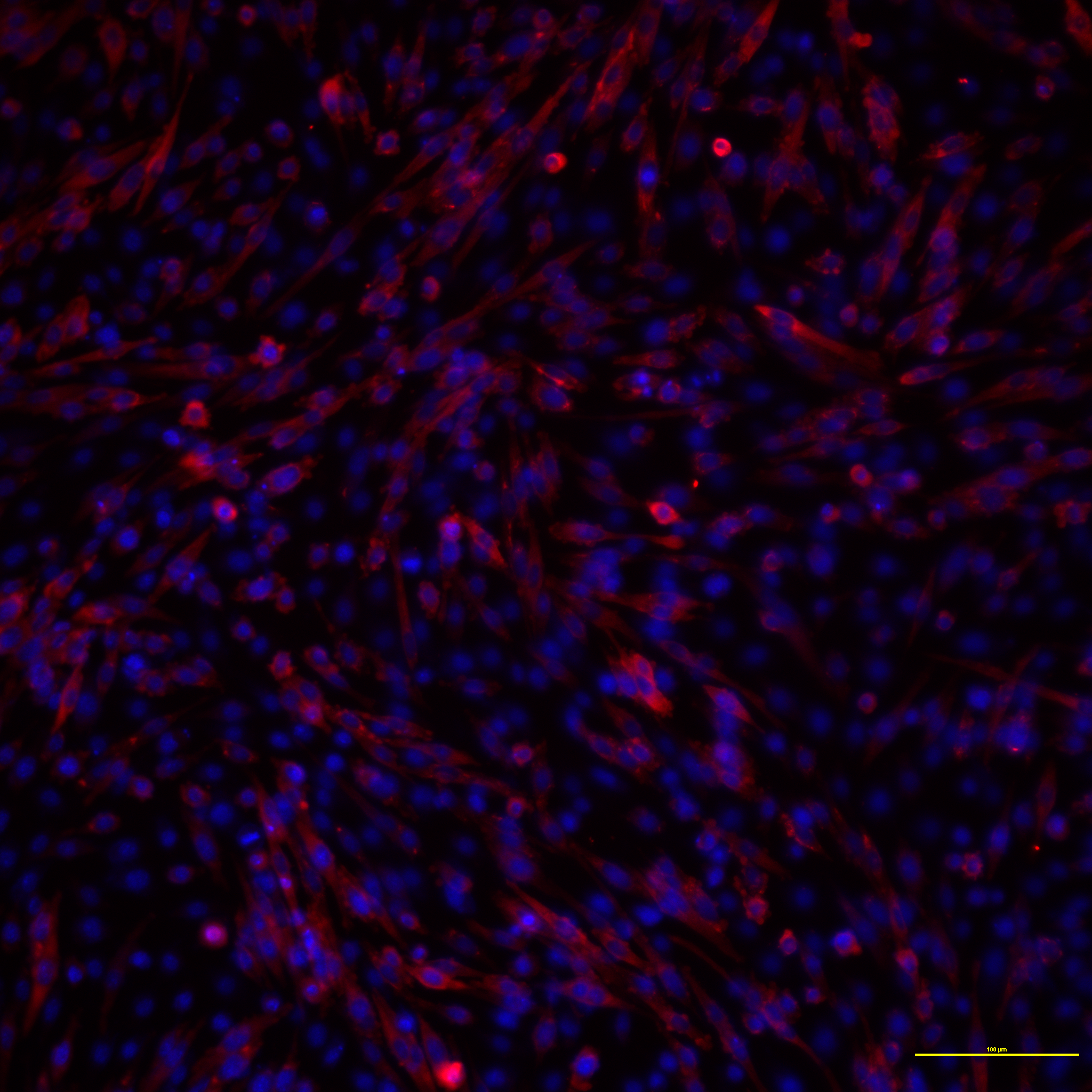

Supplement: Supplementary file 9 — Source data Fig. 6 [file 44319_2024_197_MOESM9_ESM.zip › Figure 6/6A-F/6A/XBP1 siRNA-MyHC images/24 h XBP1 siRNA Representative image.tif]

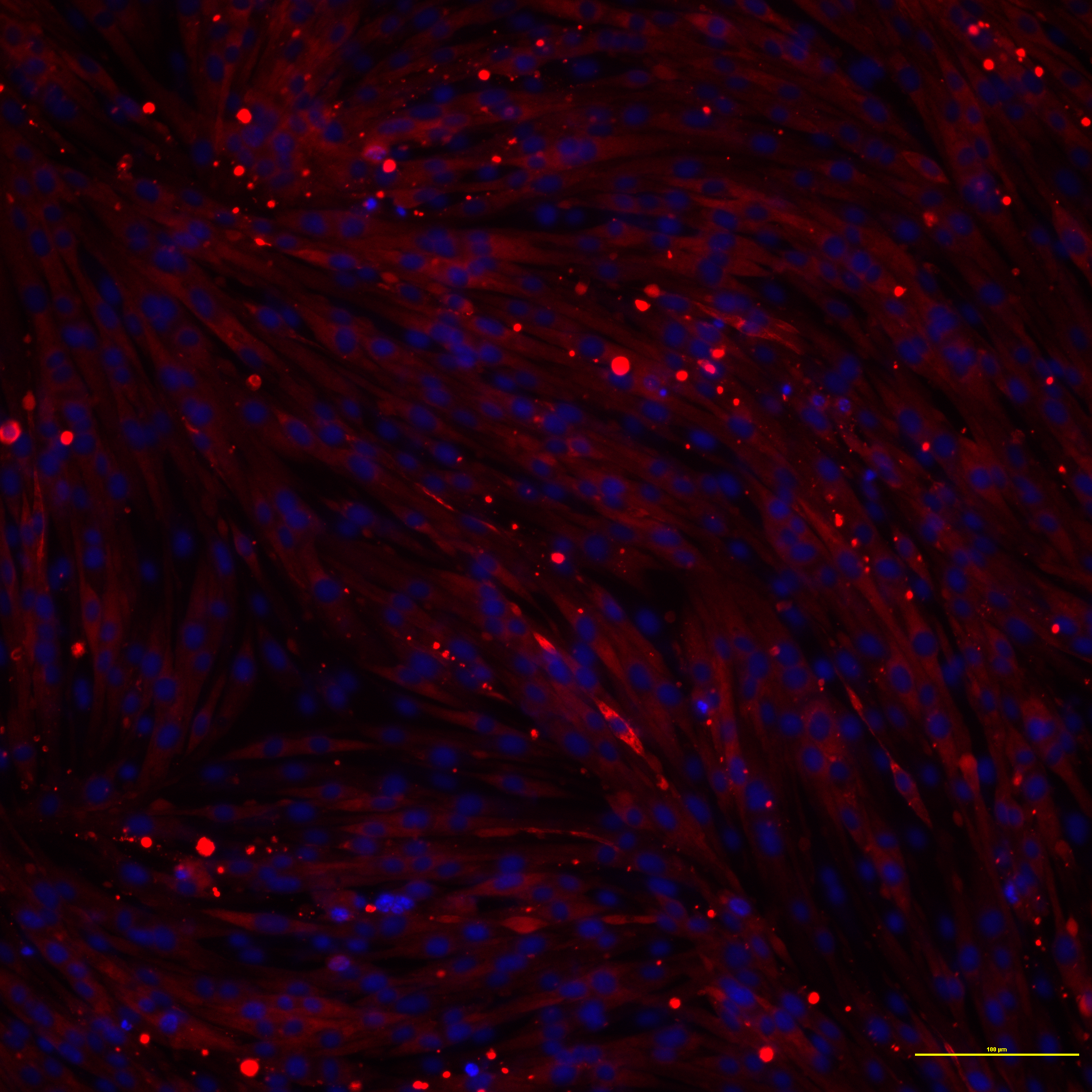

Supplement: Supplementary file 9 — Source data Fig. 6 [file 44319_2024_197_MOESM9_ESM.zip › Figure 6/6A-F/6A/XBP1 siRNA-MyHC images/48 h Control siRNA replicate 2.tif]

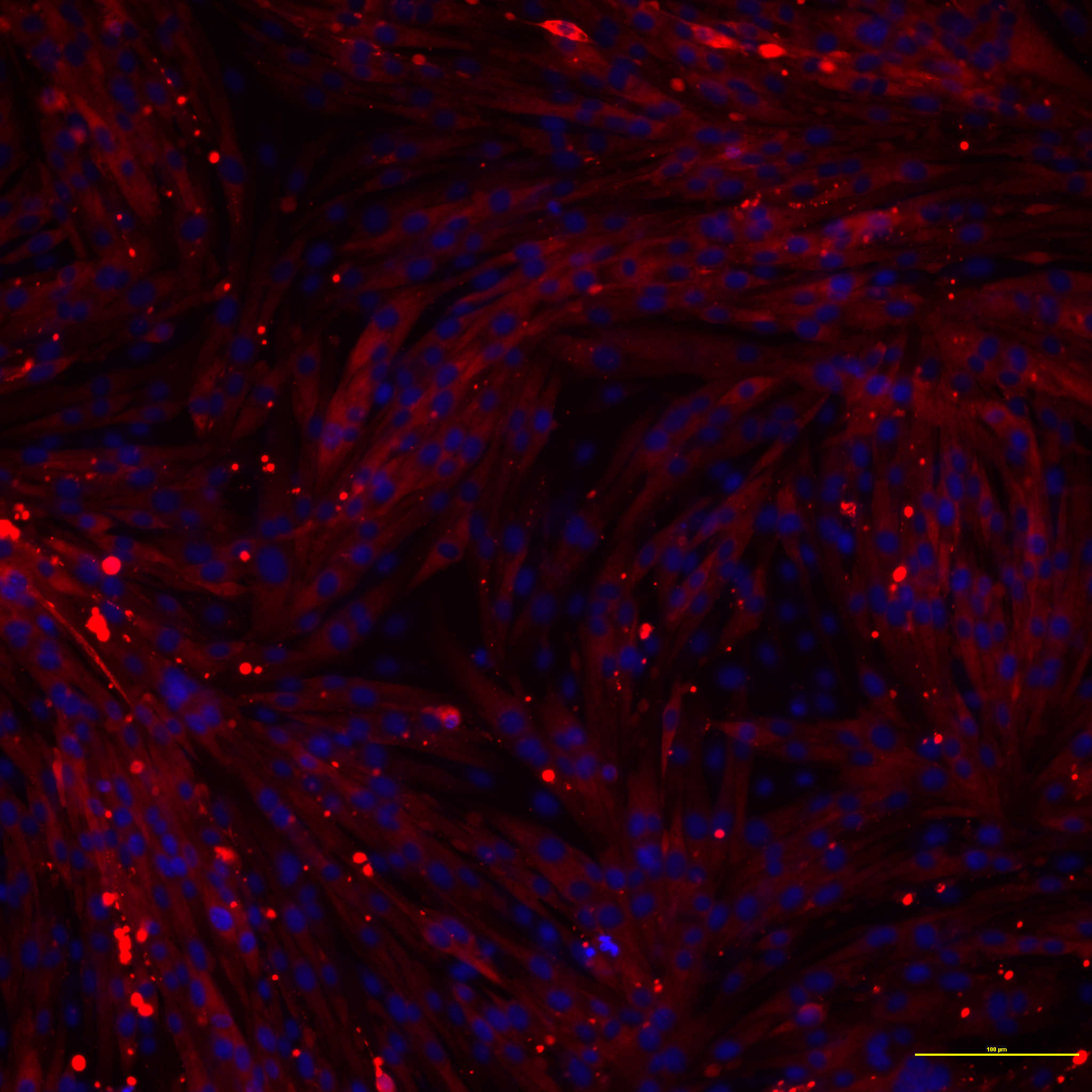

Supplement: Supplementary file 9 — Source data Fig. 6 [file 44319_2024_197_MOESM9_ESM.zip › Figure 6/6A-F/6A/XBP1 siRNA-MyHC images/48 h Control siRNA replicate 3.tif]

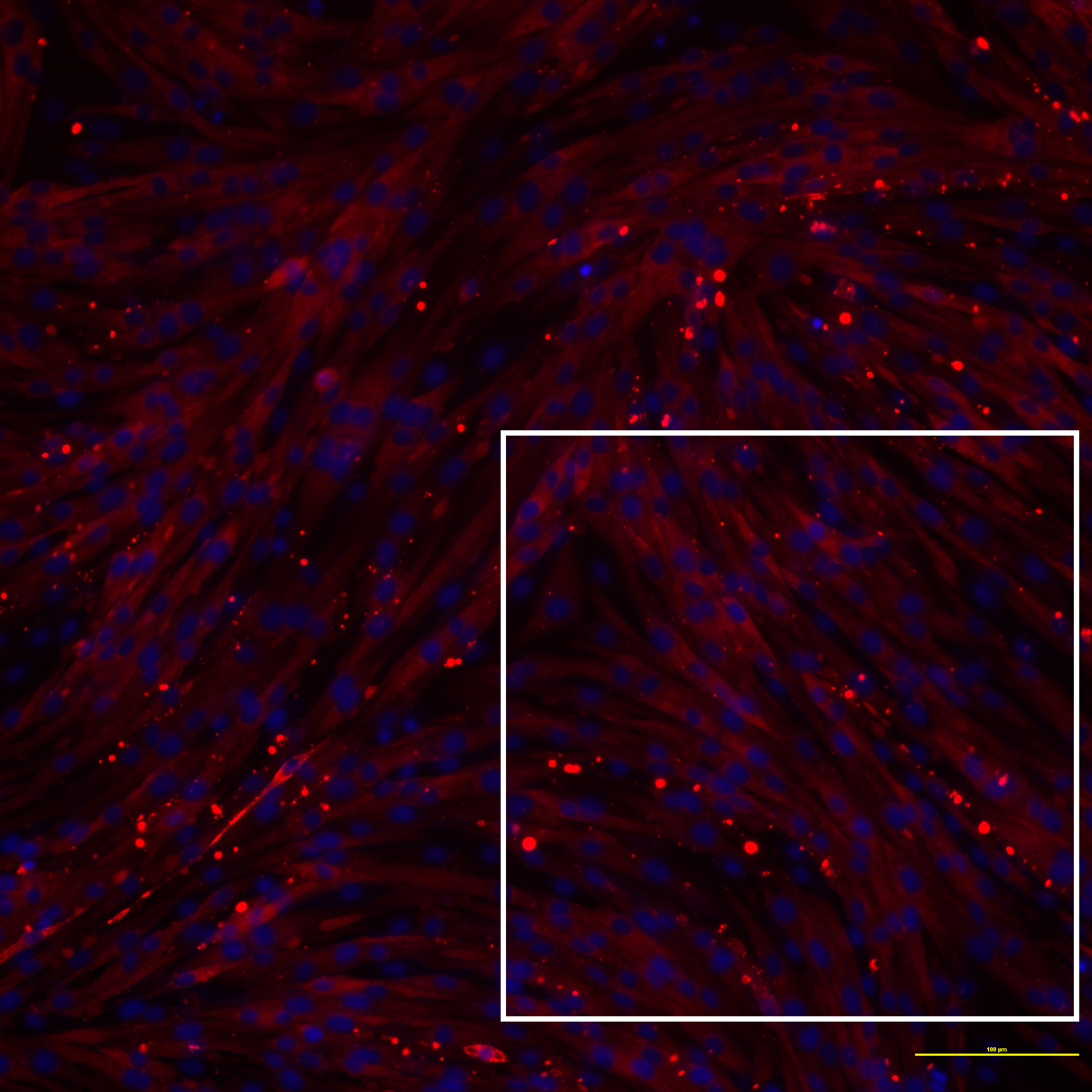

Supplement: Supplementary file 9 — Source data Fig. 6 [file 44319_2024_197_MOESM9_ESM.zip › Figure 6/6A-F/6A/XBP1 siRNA-MyHC images/48 h Control siRNA Representative image with box.tif]

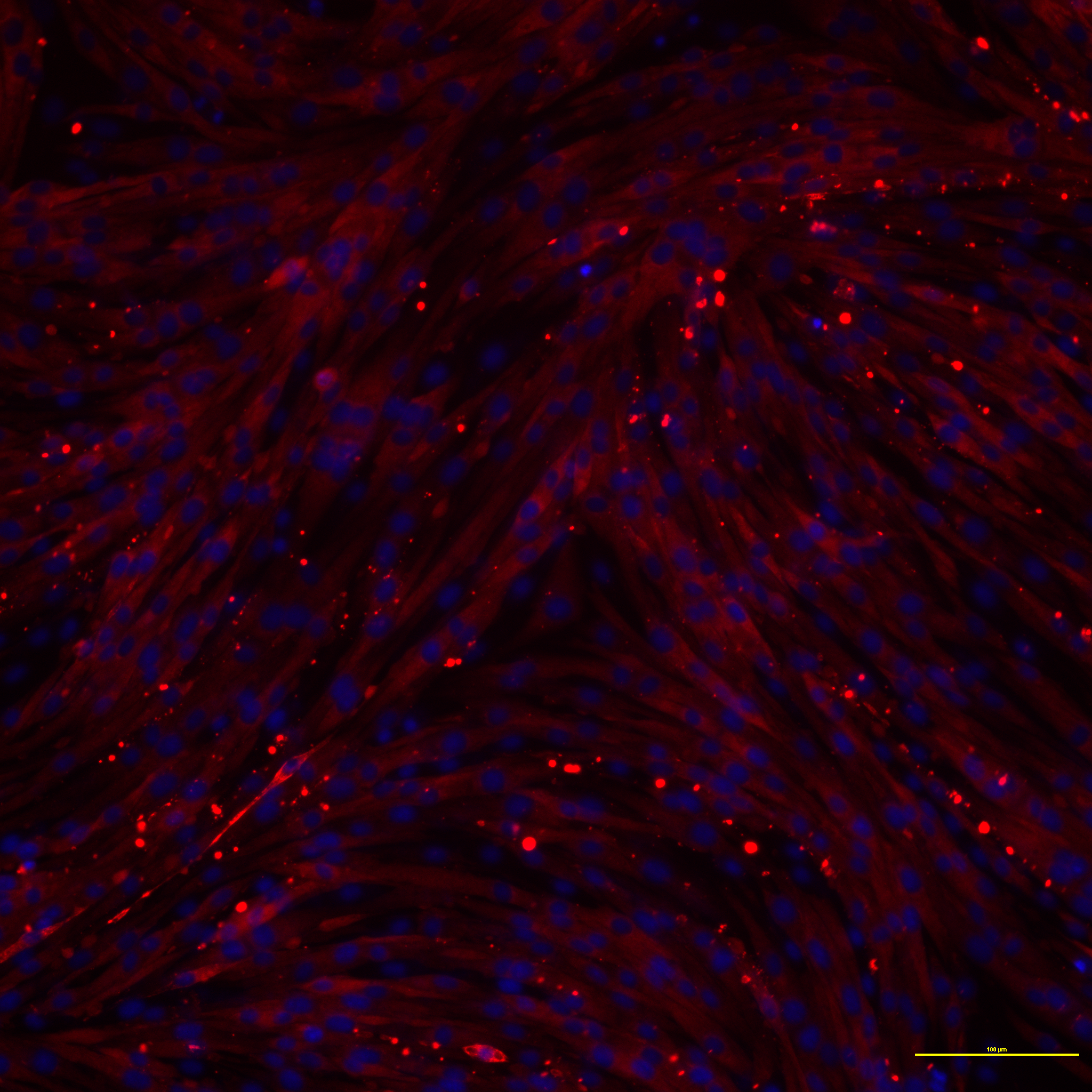

Supplement: Supplementary file 9 — Source data Fig. 6 [file 44319_2024_197_MOESM9_ESM.zip › Figure 6/6A-F/6A/XBP1 siRNA-MyHC images/48 h Control siRNA Representative image.tif]

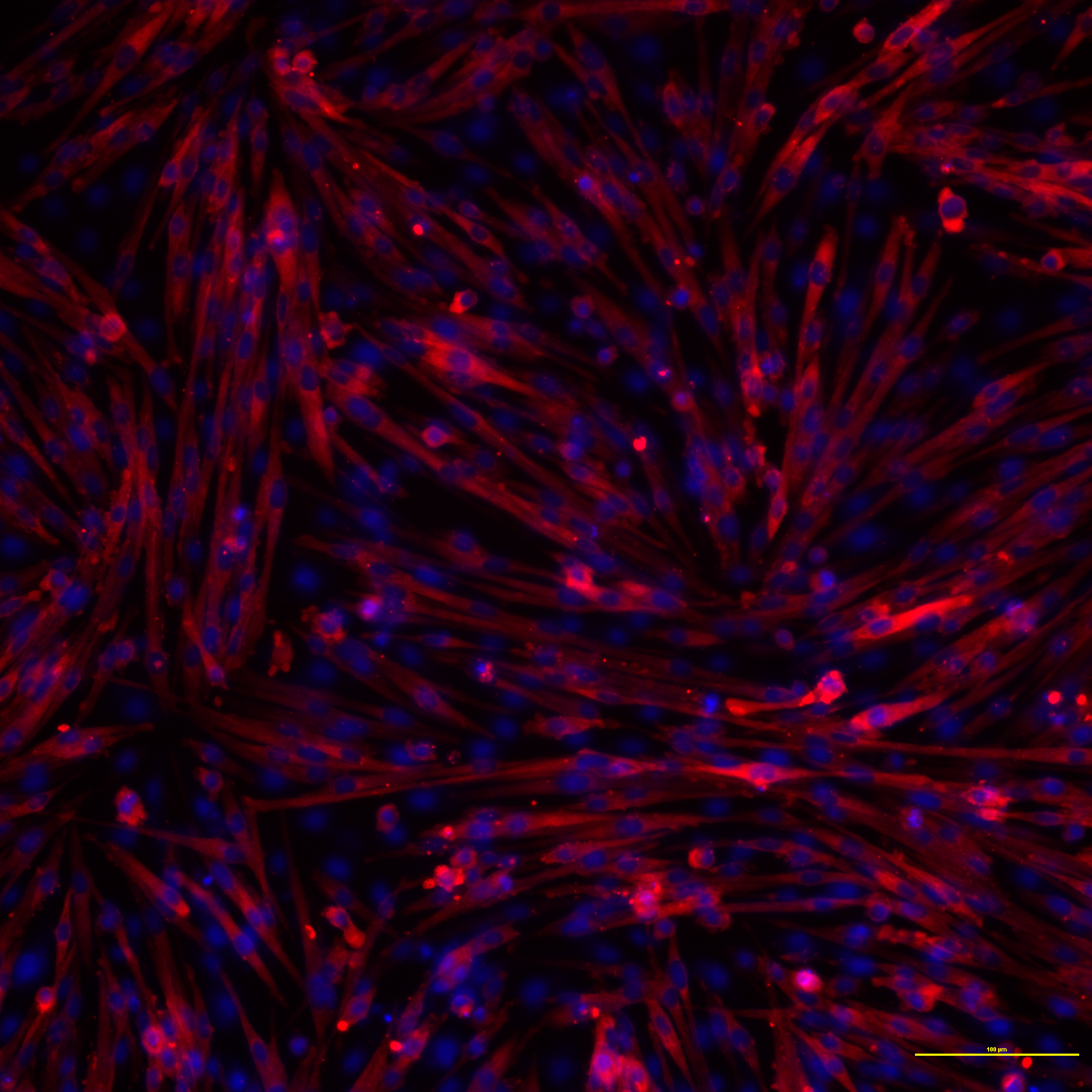

Supplement: Supplementary file 9 — Source data Fig. 6 [file 44319_2024_197_MOESM9_ESM.zip › Figure 6/6A-F/6A/XBP1 siRNA-MyHC images/48 h XBP1 siRNA replicate 2.tif]

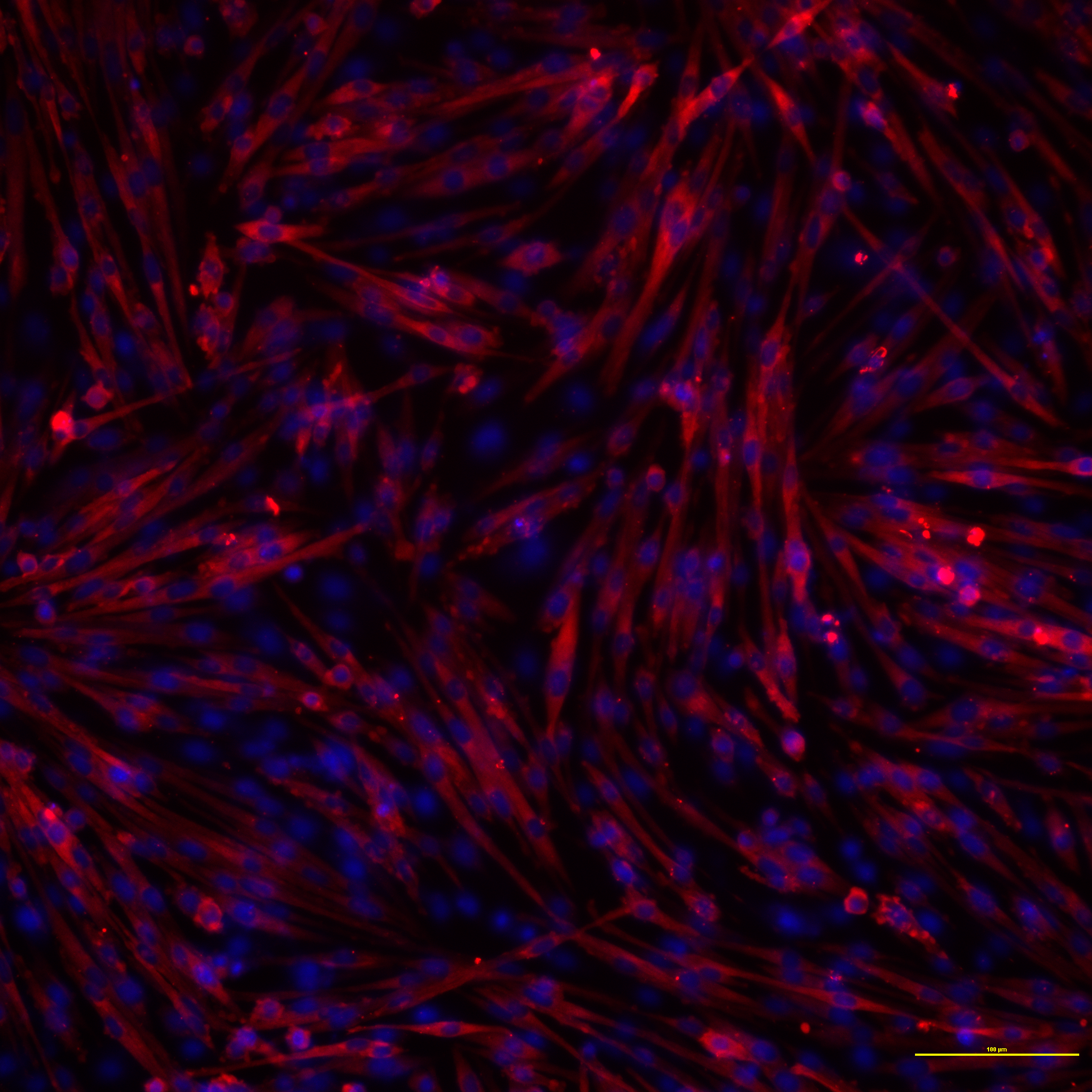

Supplement: Supplementary file 9 — Source data Fig. 6 [file 44319_2024_197_MOESM9_ESM.zip › Figure 6/6A-F/6A/XBP1 siRNA-MyHC images/48 h XBP1 siRNA replicate 3.tif]

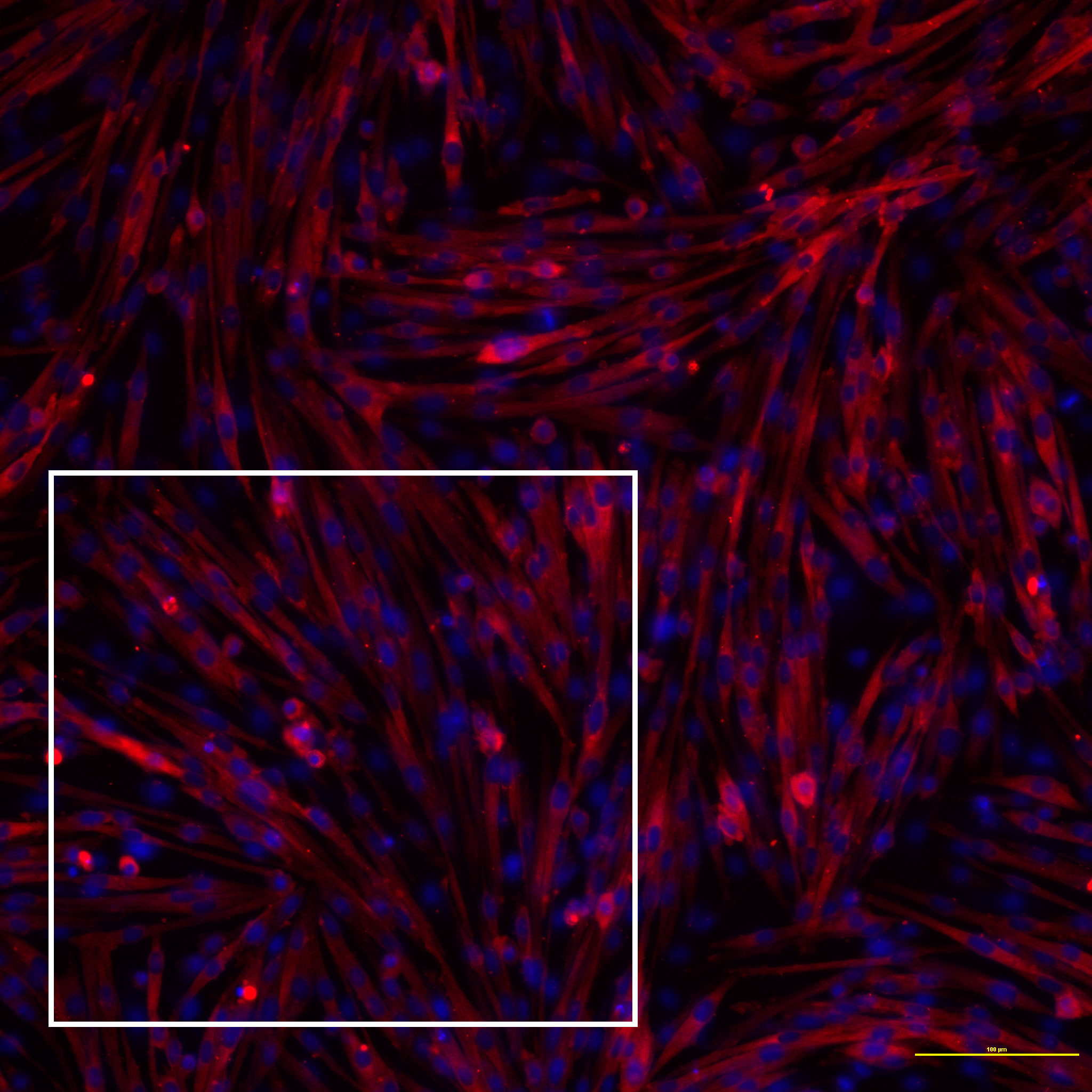

Supplement: Supplementary file 9 — Source data Fig. 6 [file 44319_2024_197_MOESM9_ESM.zip › Figure 6/6A-F/6A/XBP1 siRNA-MyHC images/48 h XBP1 siRNA Representative image with box.tif]

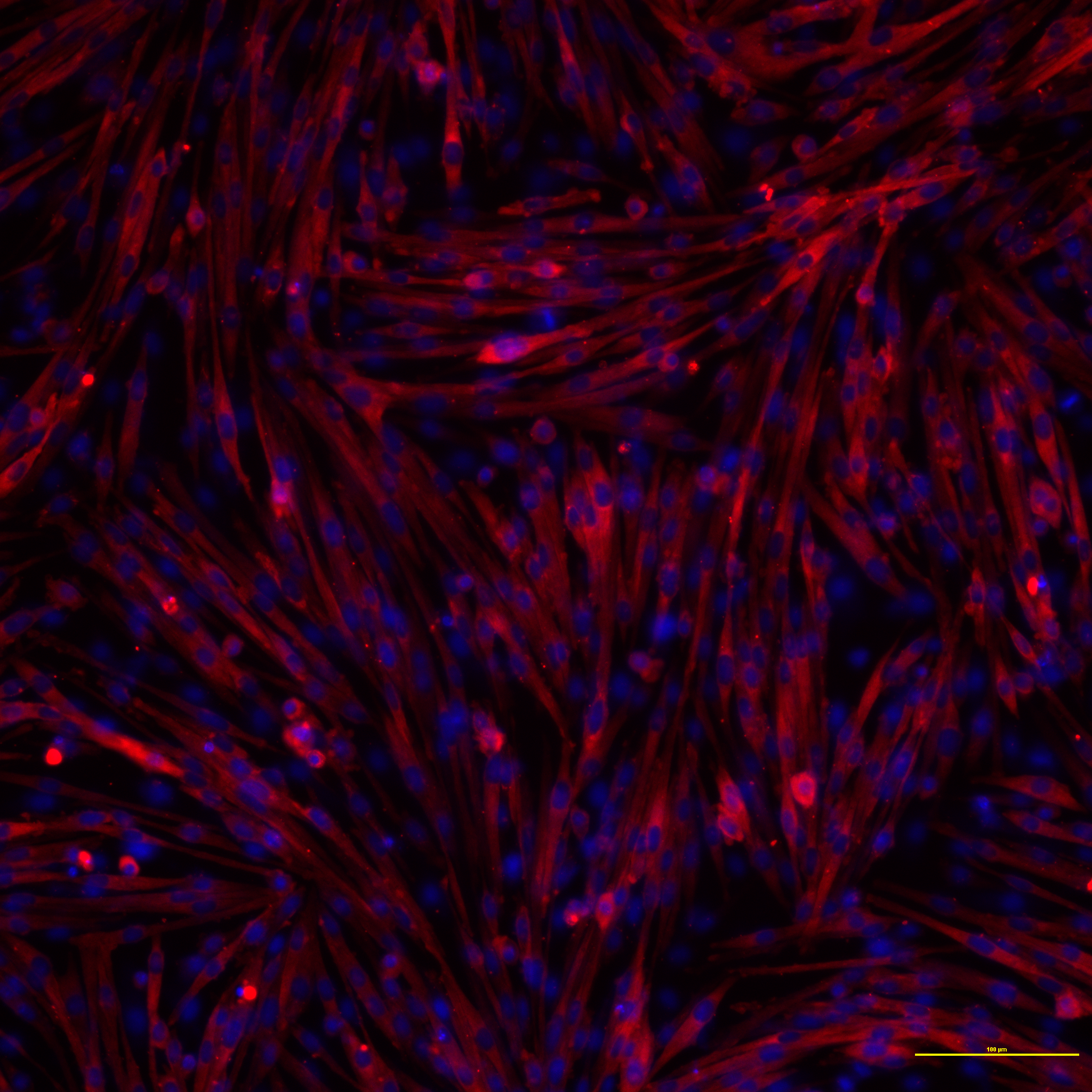

Supplement: Supplementary file 9 — Source data Fig. 6 [file 44319_2024_197_MOESM9_ESM.zip › Figure 6/6A-F/6A/XBP1 siRNA-MyHC images/48 h XBP1 siRNA Representative image.tif]

## Slide 1
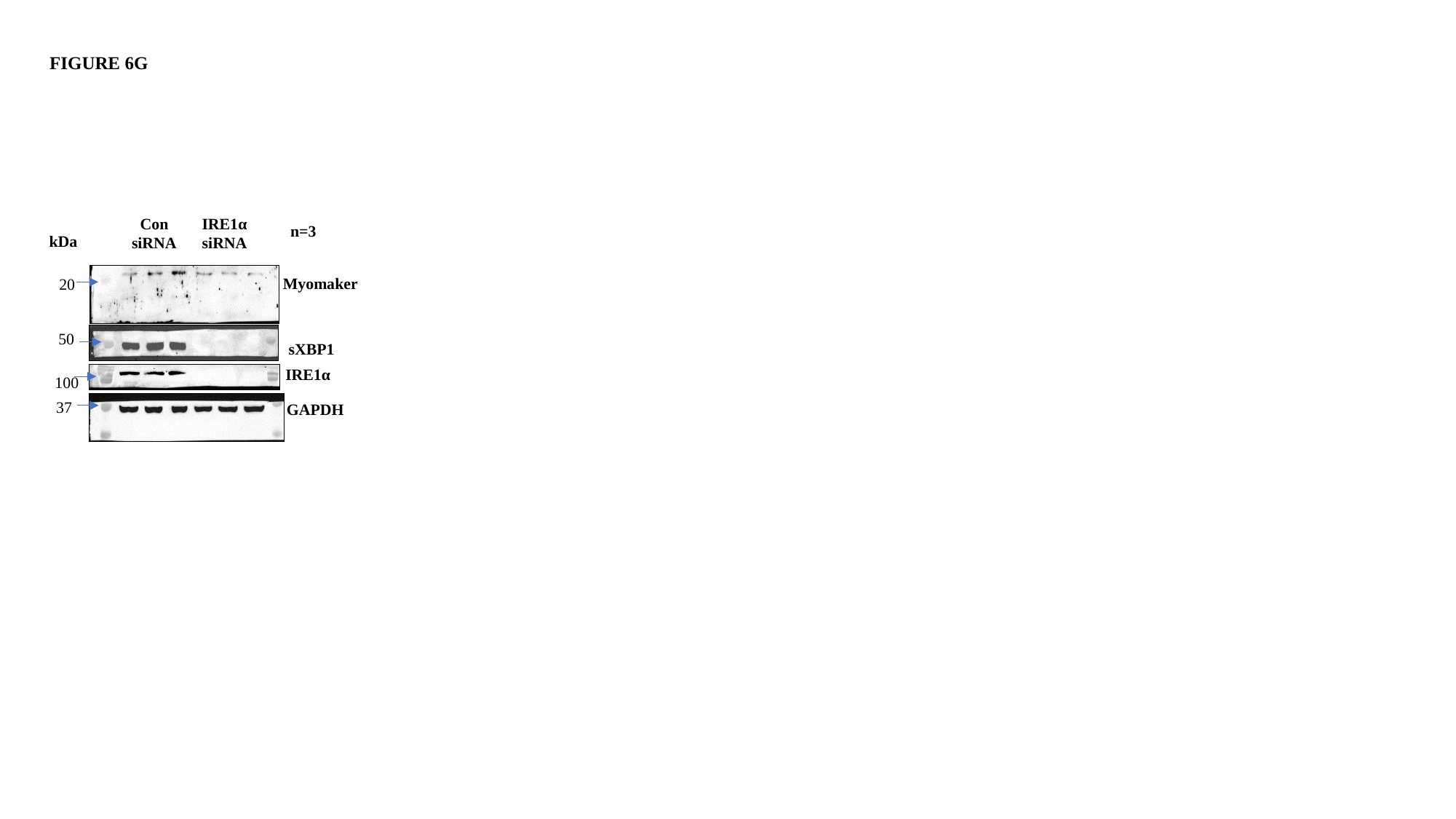

FIGURE 6G
Con siRNA
IRE1α siRNA
n=3
kDa
Myomaker
20
50
sXBP1
IRE1α
100
37
GAPDH

Supplement: Supplementary file 9 — Source data Fig. 6 [file 44319_2024_197_MOESM9_ESM.zip › Figure 6/6G/Western blot with annotation.pptx]

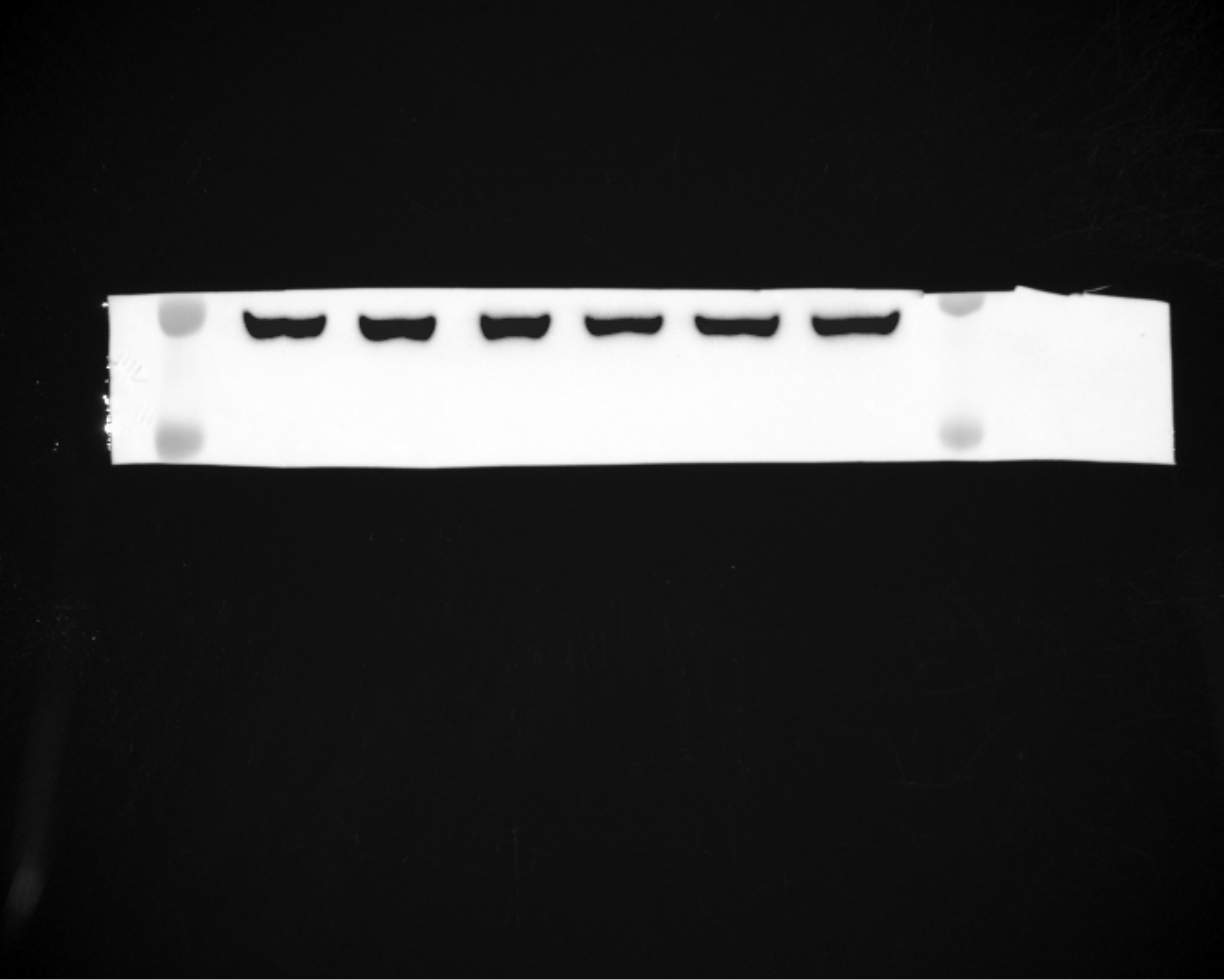

Supplement: Supplementary file 9 — Source data Fig. 6 [file 44319_2024_197_MOESM9_ESM.zip › Figure 6/6G/Western-GAPDH.tif]

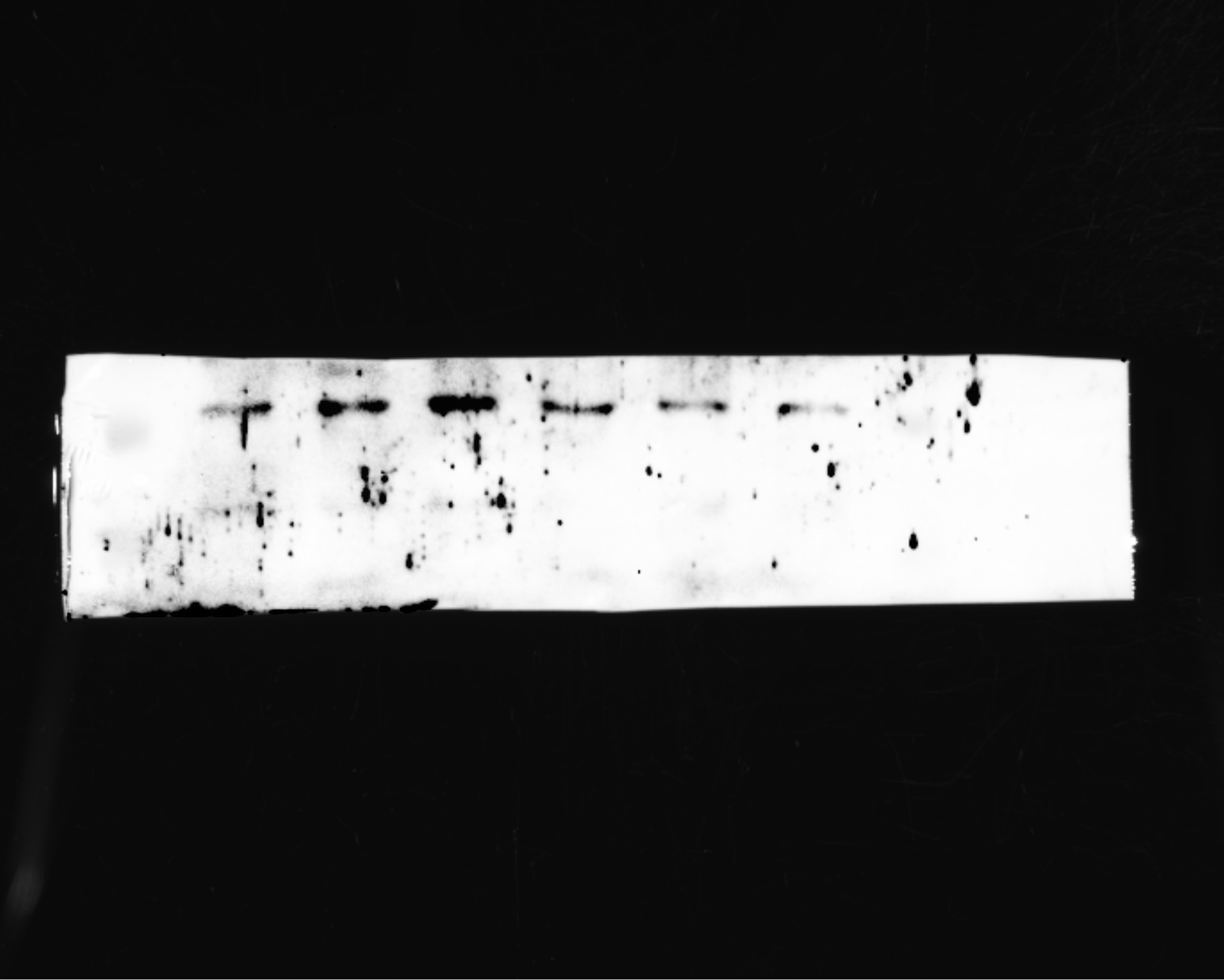

Supplement: Supplementary file 9 — Source data Fig. 6 [file 44319_2024_197_MOESM9_ESM.zip › Figure 6/6G/Western-Myomaker.tif]

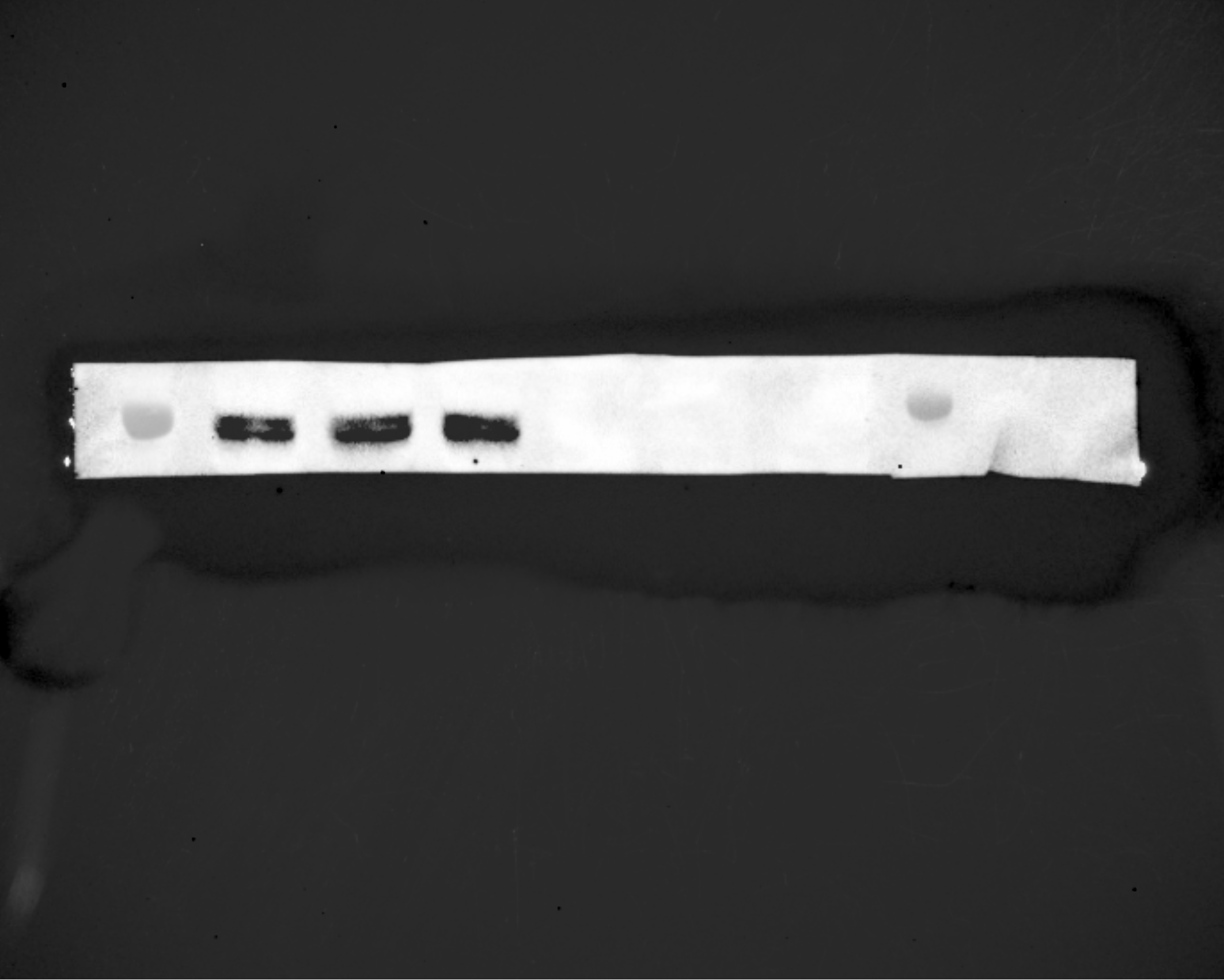

Supplement: Supplementary file 9 — Source data Fig. 6 [file 44319_2024_197_MOESM9_ESM.zip › Figure 6/6G/Western-sXBP1.tif]

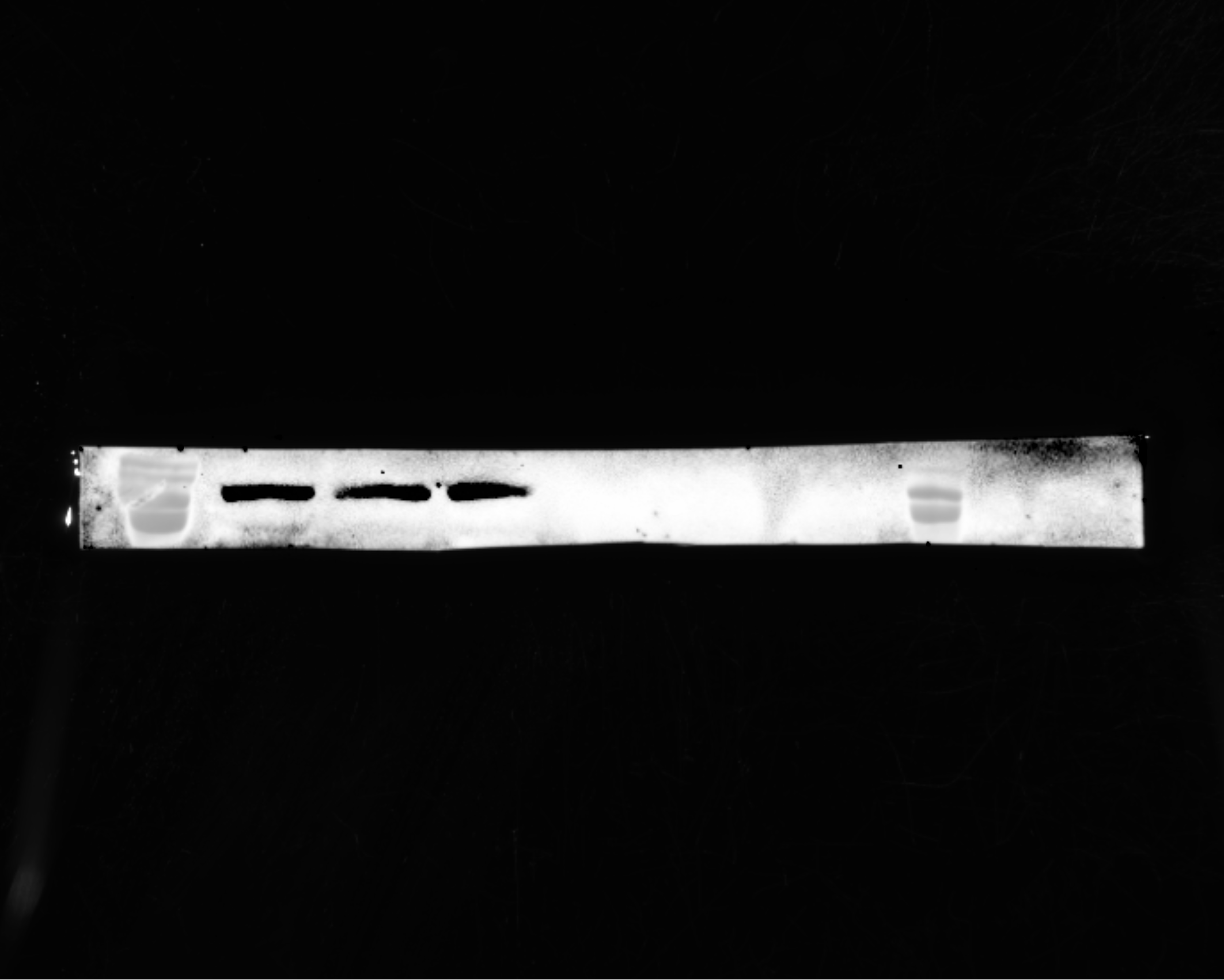

Supplement: Supplementary file 9 — Source data Fig. 6 [file 44319_2024_197_MOESM9_ESM.zip › Figure 6/6G/Western-Total IRE1a.tif]

## Slide 1
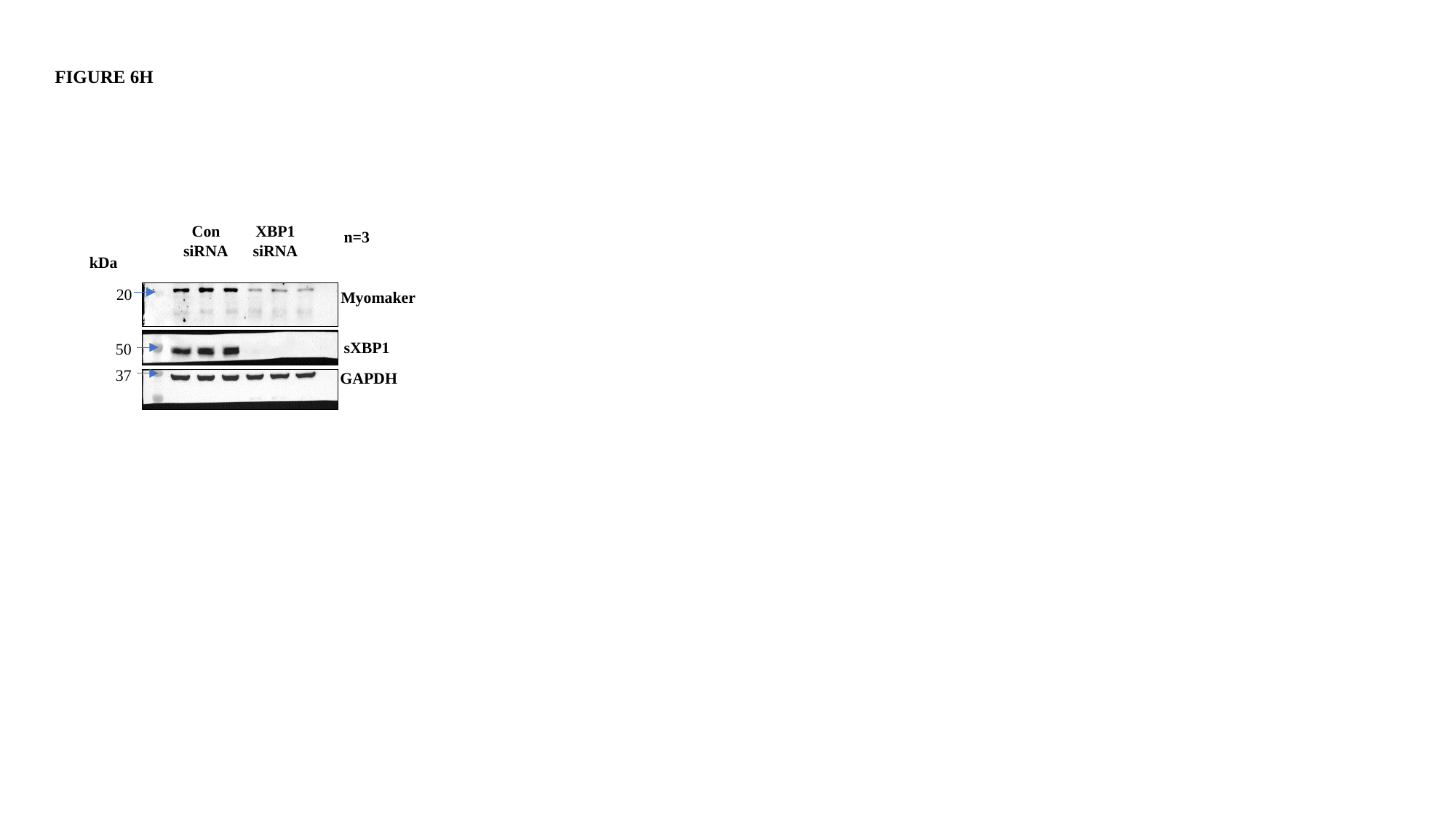

FIGURE 6H
Con
siRNA
XBP1
siRNA
n=3
kDa
20
Myomaker
sXBP1
50
37
GAPDH

Supplement: Supplementary file 9 — Source data Fig. 6 [file 44319_2024_197_MOESM9_ESM.zip › Figure 6/6H/Western blot with annotation.pptx]

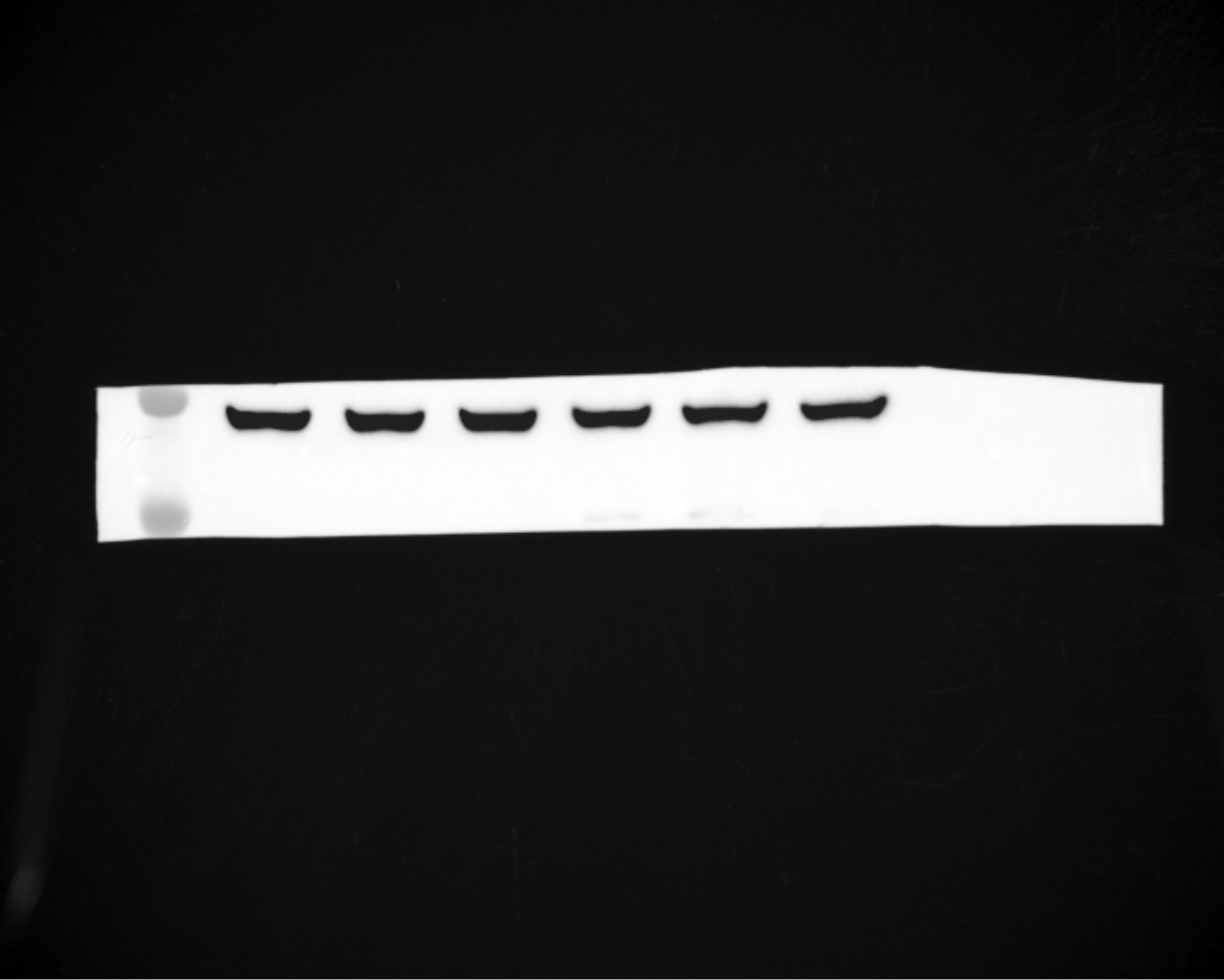

Supplement: Supplementary file 9 — Source data Fig. 6 [file 44319_2024_197_MOESM9_ESM.zip › Figure 6/6H/Western-GAPDH.tif]

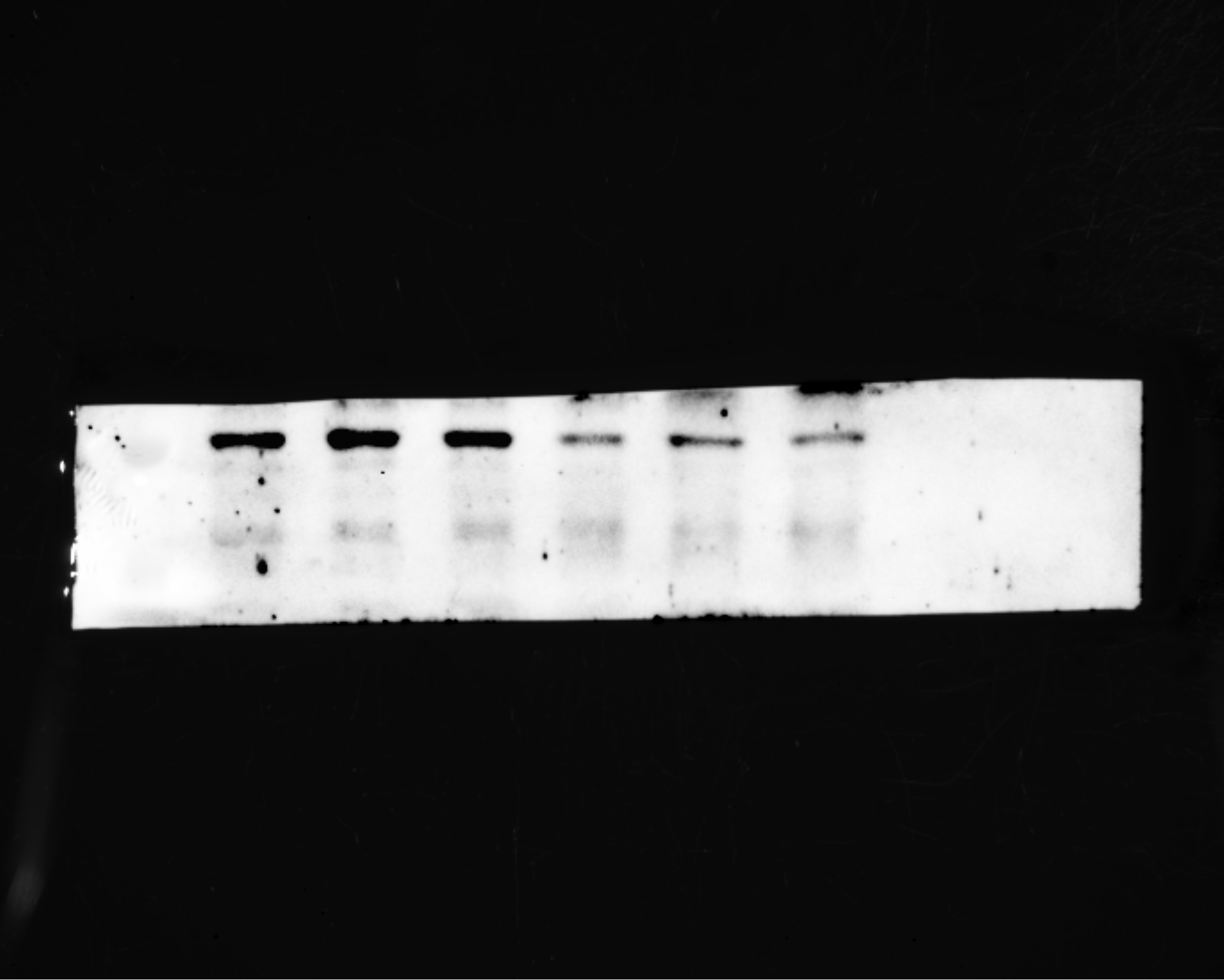

Supplement: Supplementary file 9 — Source data Fig. 6 [file 44319_2024_197_MOESM9_ESM.zip › Figure 6/6H/Western-Myomaker.tif]

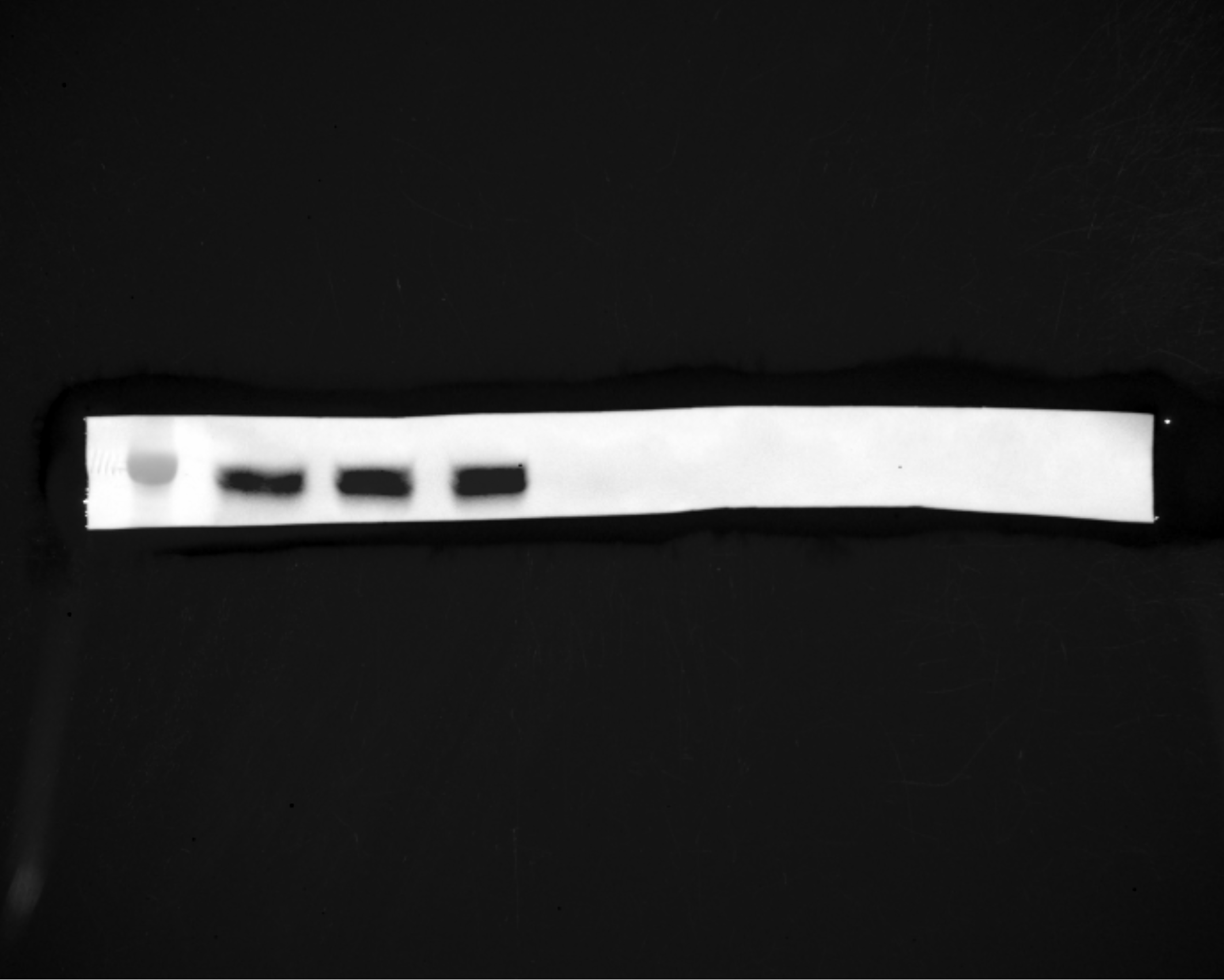

Supplement: Supplementary file 9 — Source data Fig. 6 [file 44319_2024_197_MOESM9_ESM.zip › Figure 6/6H/Western-sXBP1.tif]

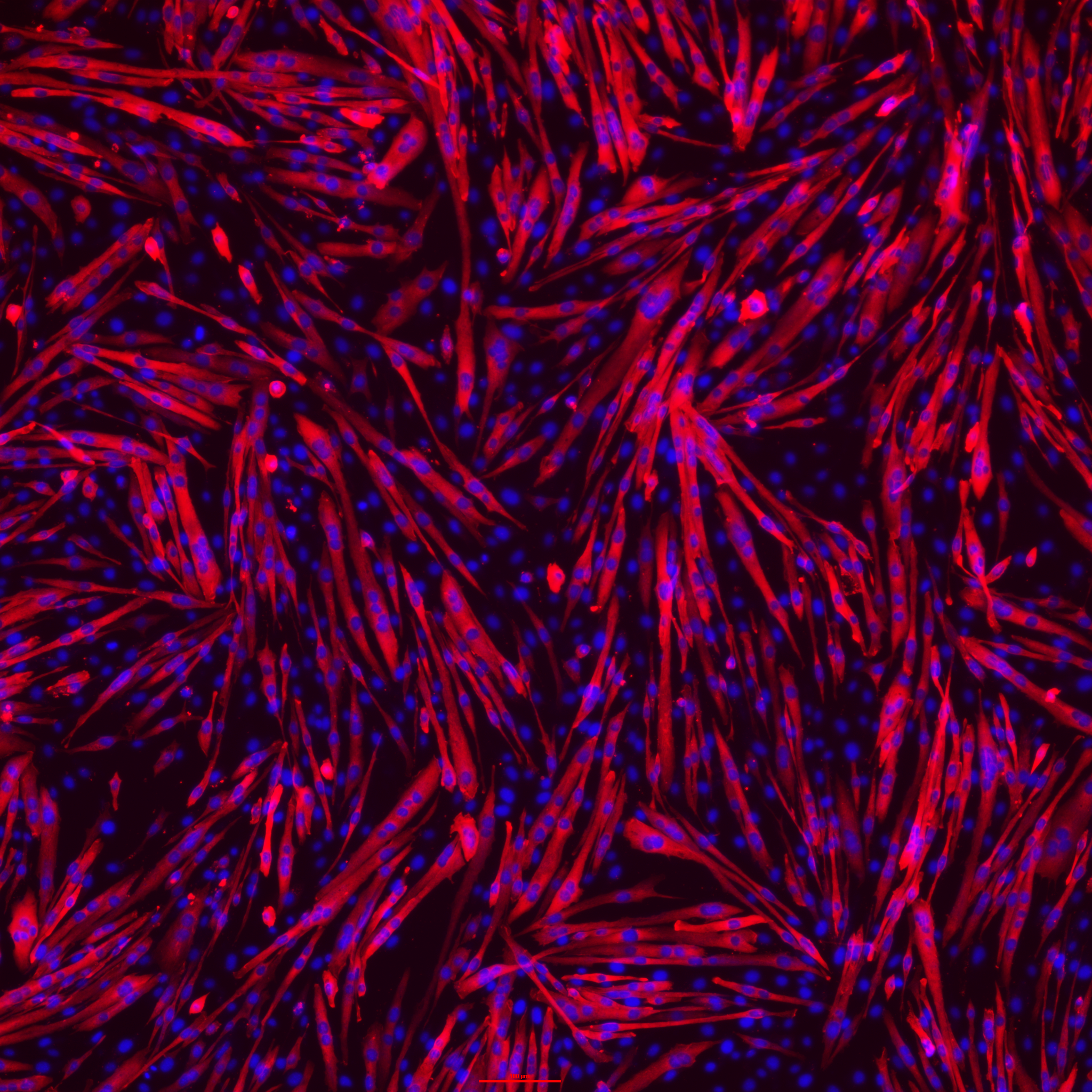

Supplement: Supplementary file 9 — Source data Fig. 6 [file 44319_2024_197_MOESM9_ESM.zip › Figure 6/6I-L/6I/sXBP1 OE-MyHC staining images/24 h Control replicate 2.tif]

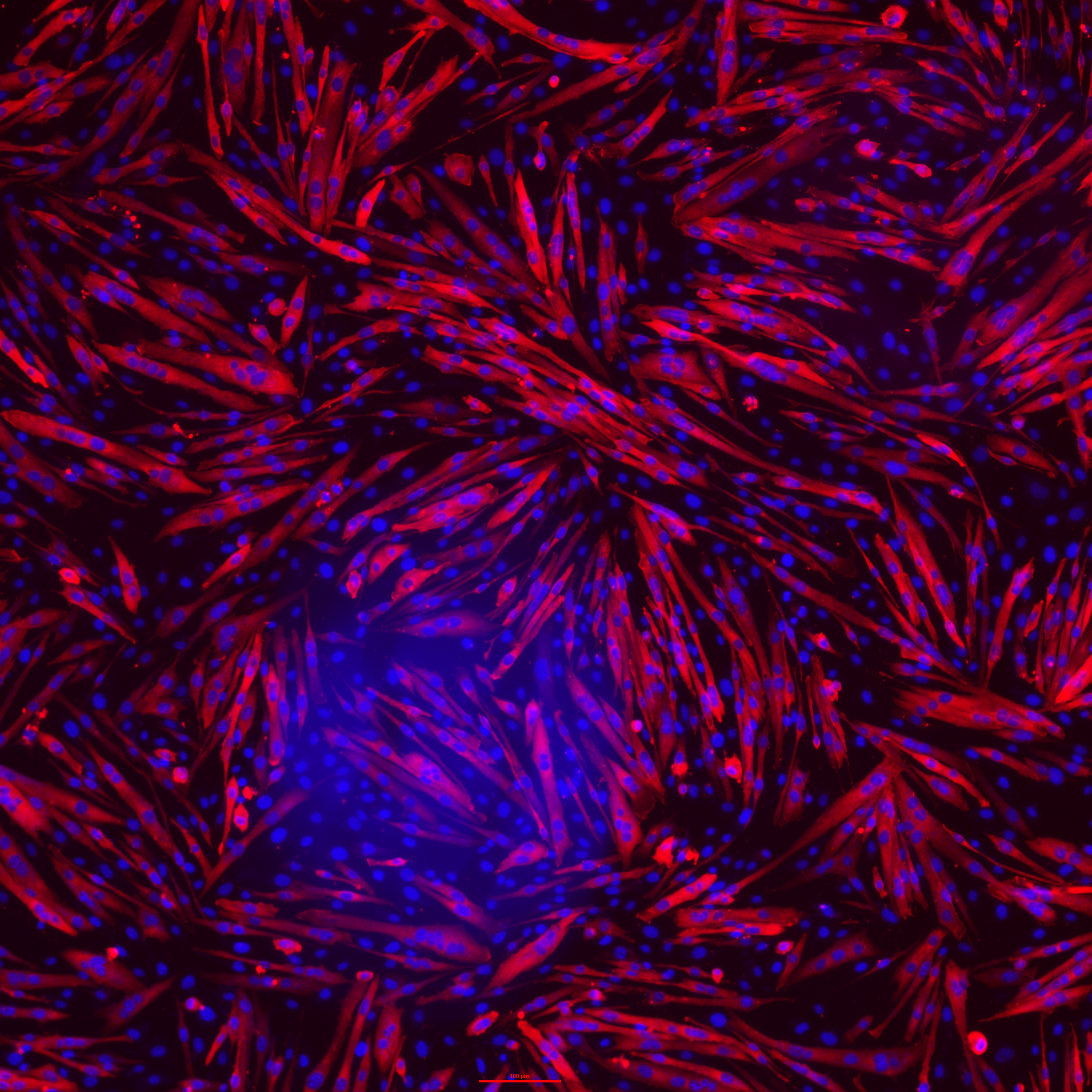

Supplement: Supplementary file 9 — Source data Fig. 6 [file 44319_2024_197_MOESM9_ESM.zip › Figure 6/6I-L/6I/sXBP1 OE-MyHC staining images/24 h Control replicate 3.tif]

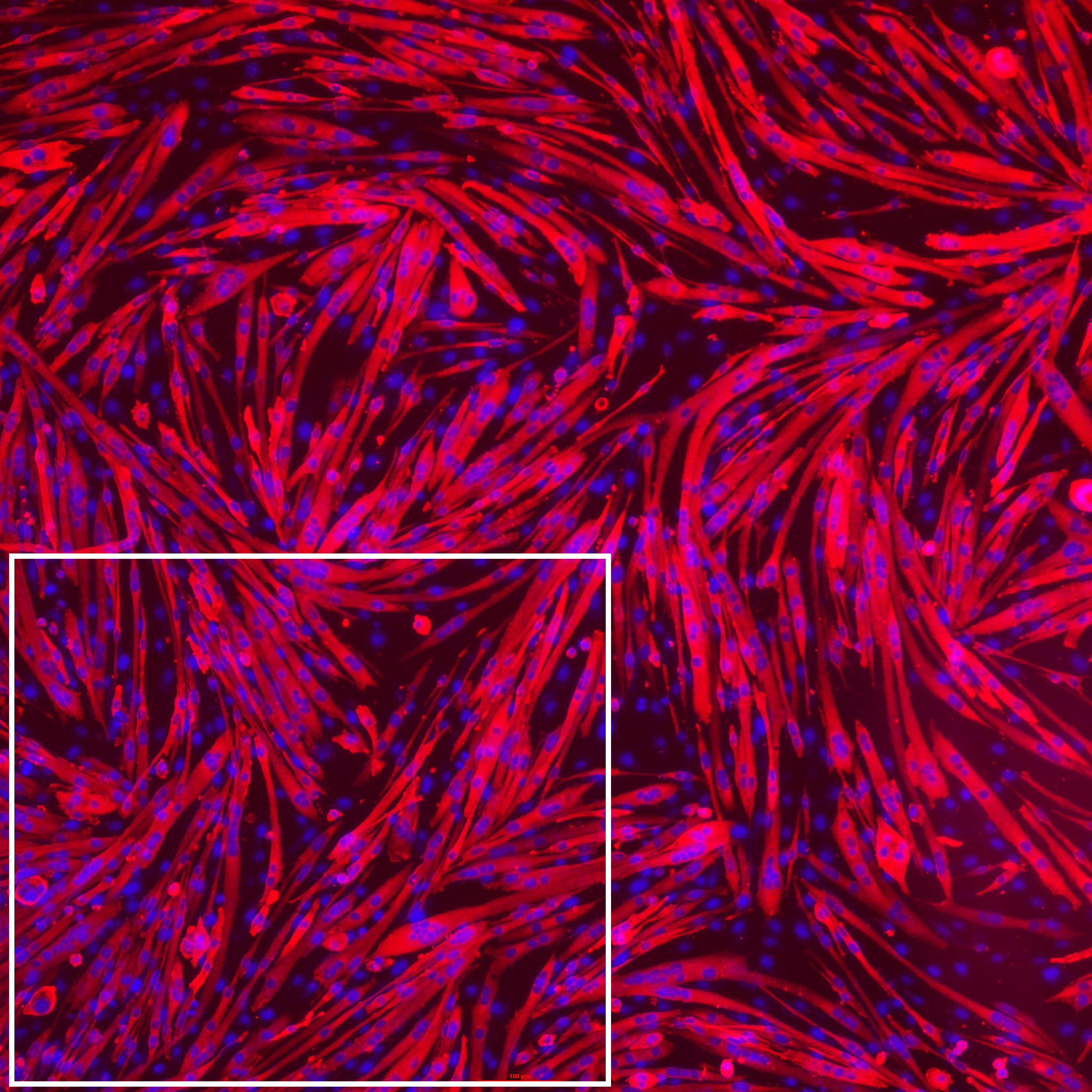

Supplement: Supplementary file 9 — Source data Fig. 6 [file 44319_2024_197_MOESM9_ESM.zip › Figure 6/6I-L/6I/sXBP1 OE-MyHC staining images/24 h Control Representative image with box.tif]

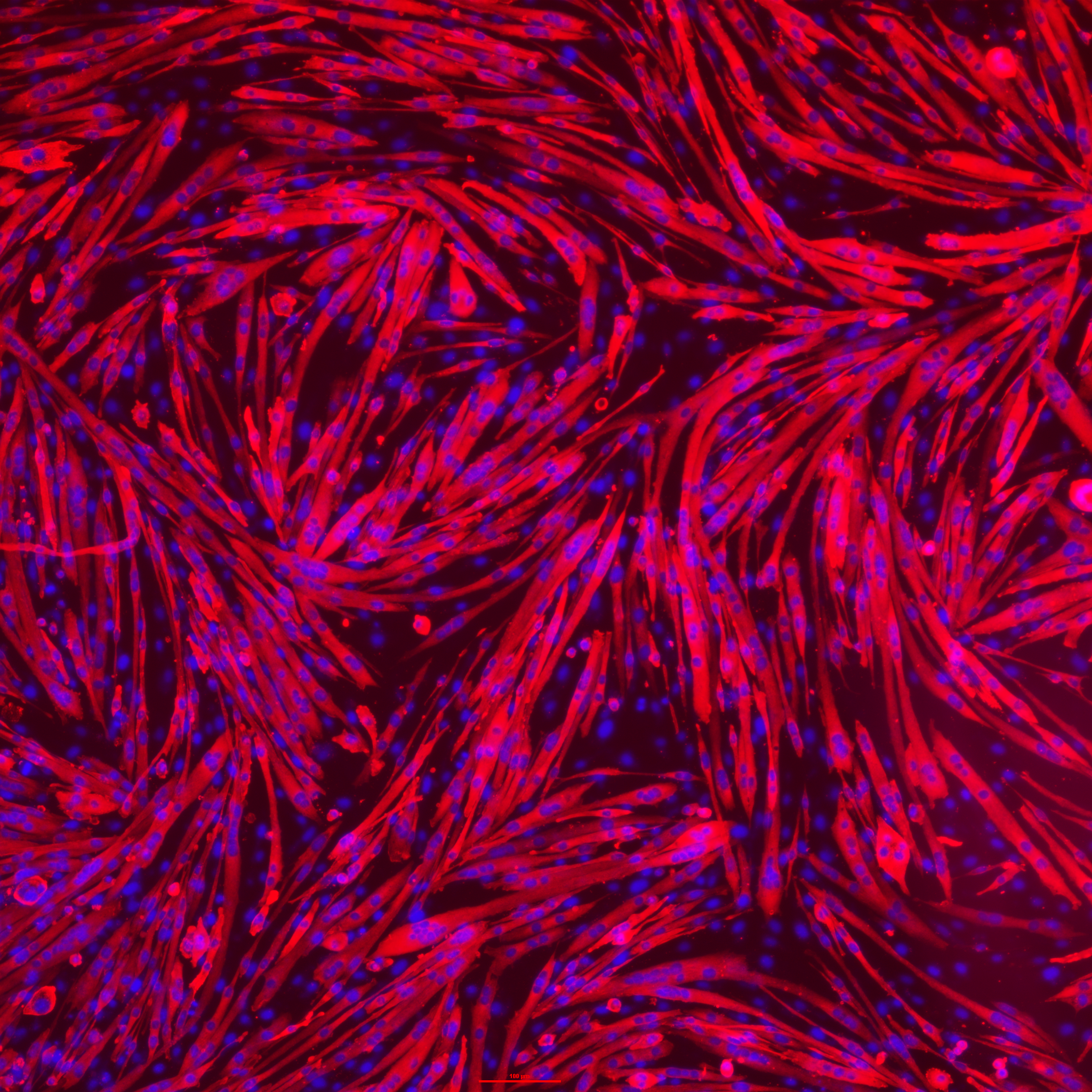

Supplement: Supplementary file 9 — Source data Fig. 6 [file 44319_2024_197_MOESM9_ESM.zip › Figure 6/6I-L/6I/sXBP1 OE-MyHC staining images/24 h Control Representative image.tif]

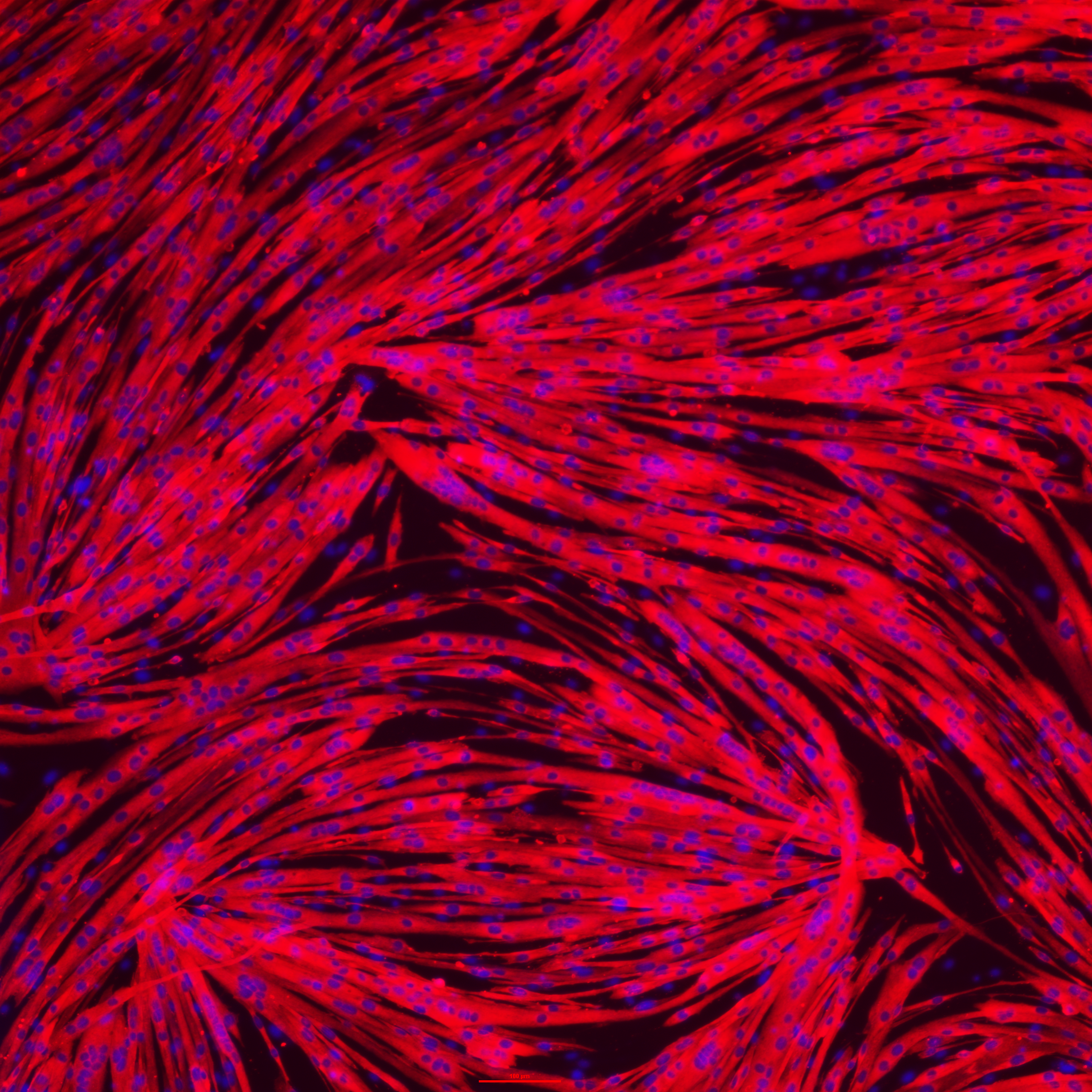

Supplement: Supplementary file 9 — Source data Fig. 6 [file 44319_2024_197_MOESM9_ESM.zip › Figure 6/6I-L/6I/sXBP1 OE-MyHC staining images/24 h sXBP1 OE replicate 2.tif]

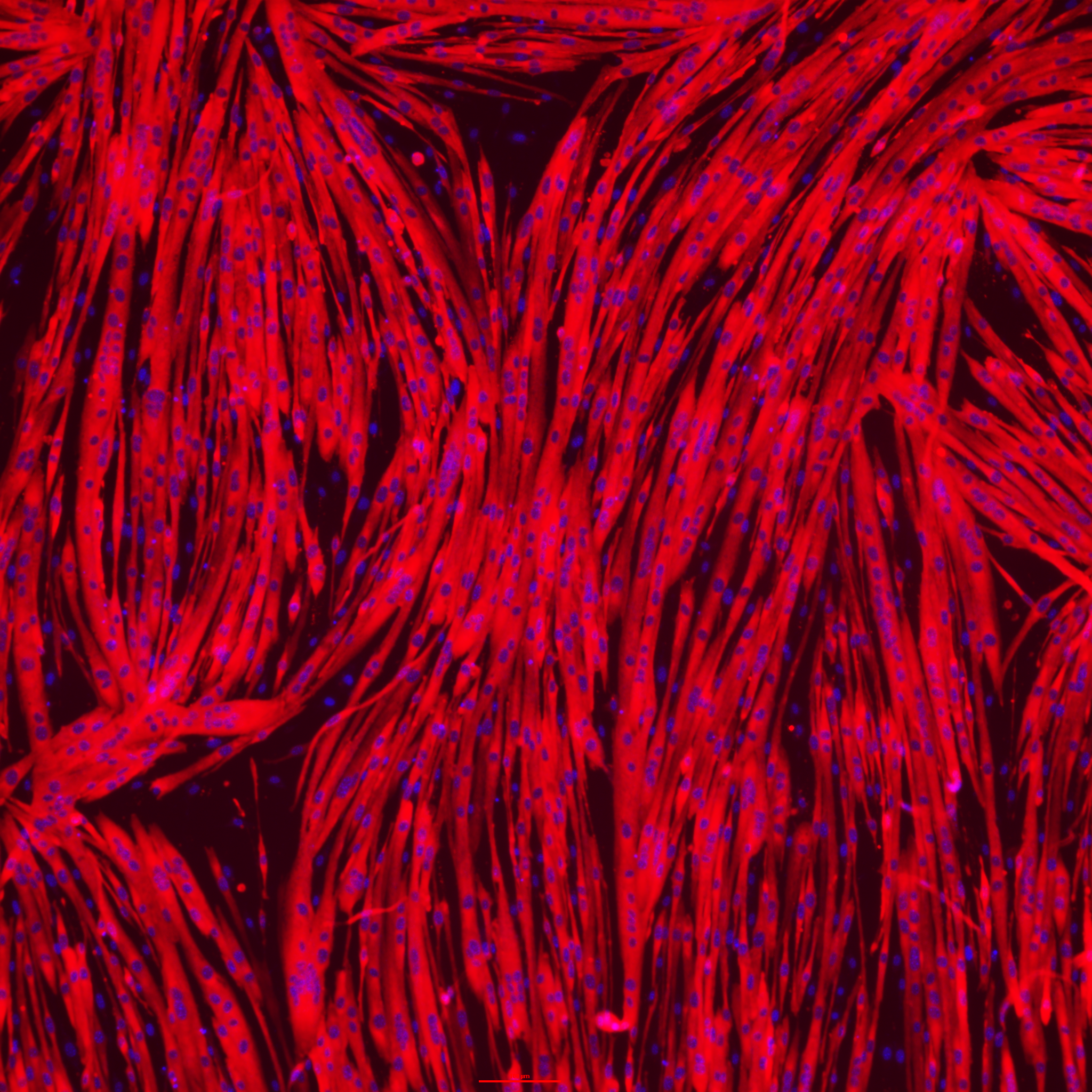

Supplement: Supplementary file 9 — Source data Fig. 6 [file 44319_2024_197_MOESM9_ESM.zip › Figure 6/6I-L/6I/sXBP1 OE-MyHC staining images/24 h sXBP1 OE replicate 3.tif]

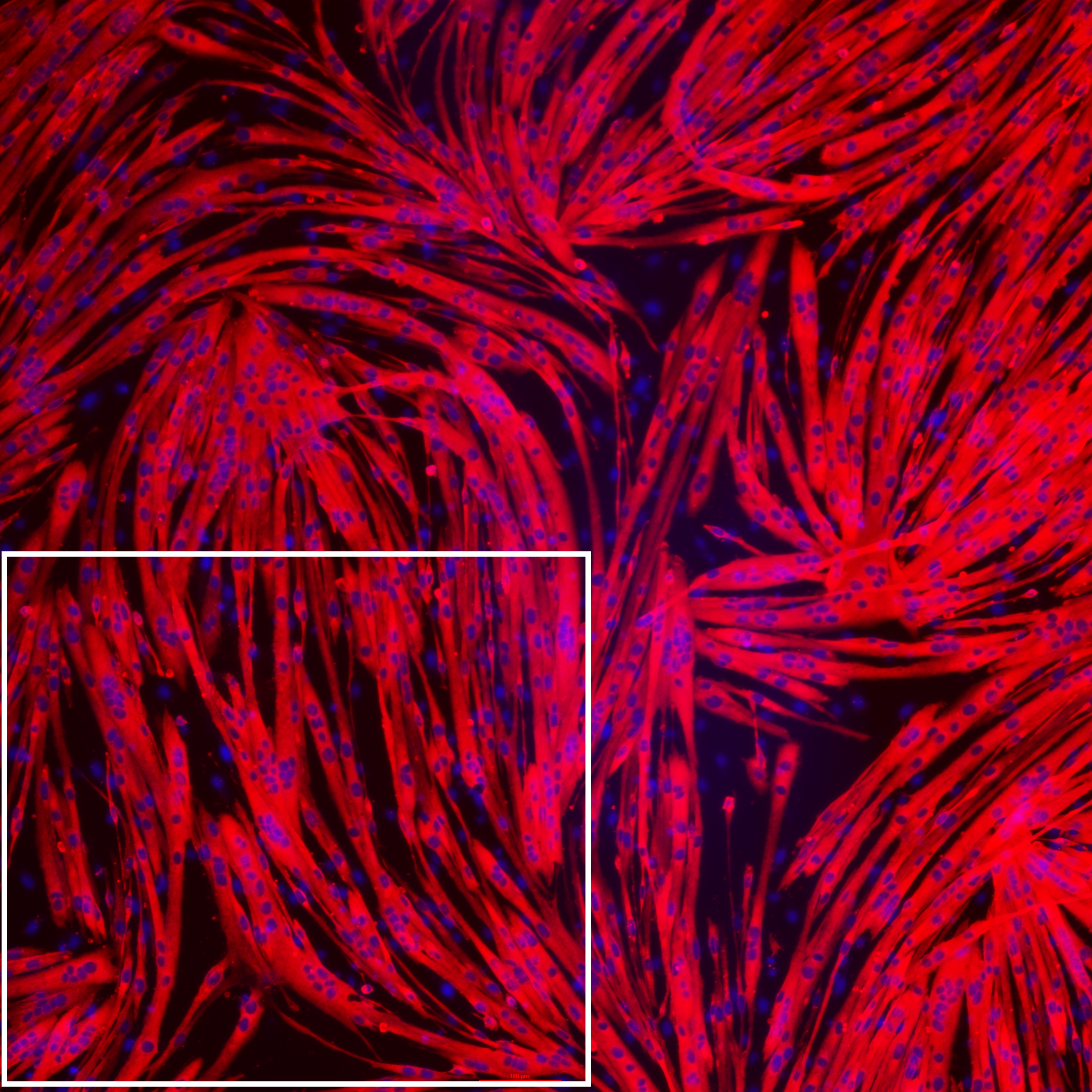

Supplement: Supplementary file 9 — Source data Fig. 6 [file 44319_2024_197_MOESM9_ESM.zip › Figure 6/6I-L/6I/sXBP1 OE-MyHC staining images/24 h sXBP1 OE Representative image with box.tif]

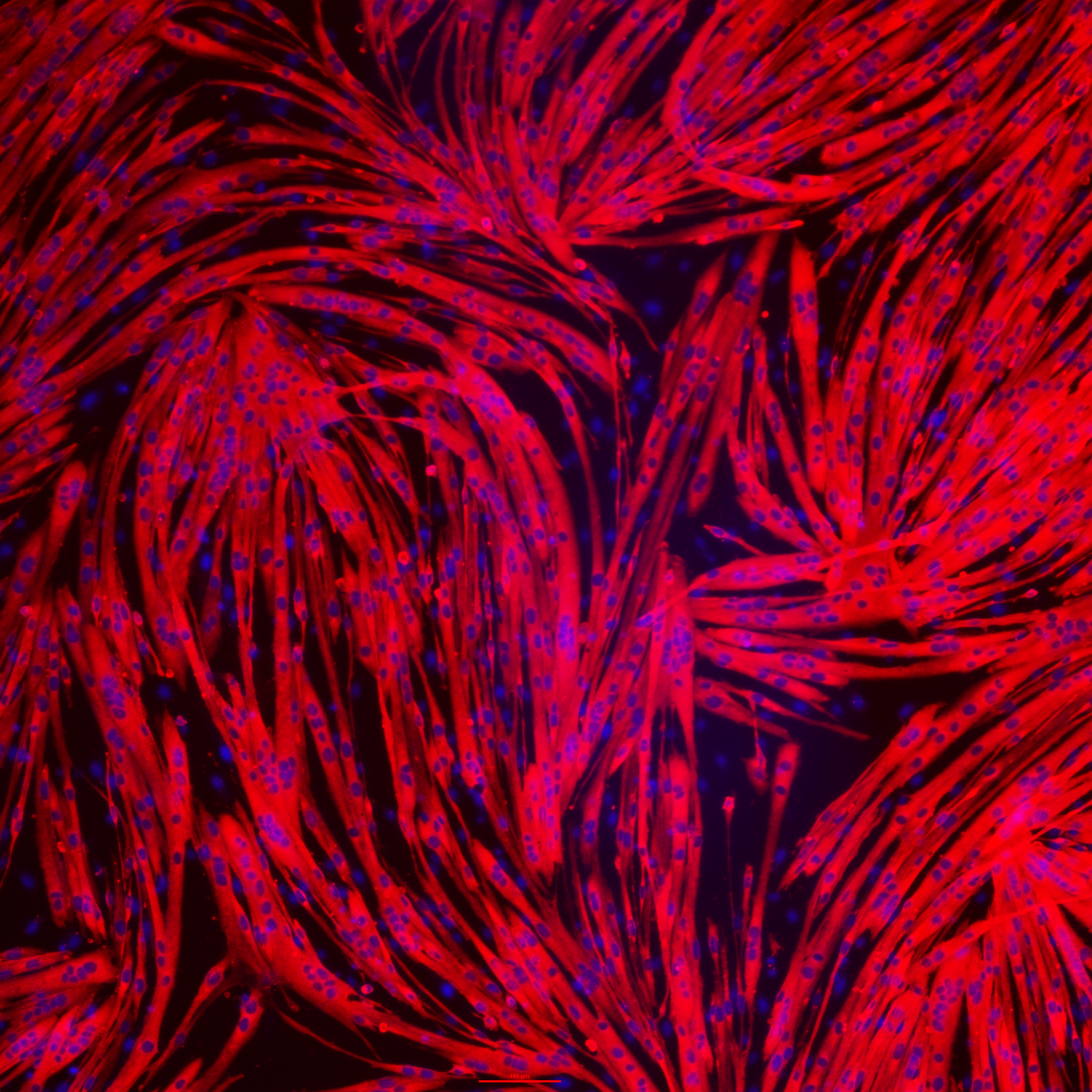

Supplement: Supplementary file 9 — Source data Fig. 6 [file 44319_2024_197_MOESM9_ESM.zip › Figure 6/6I-L/6I/sXBP1 OE-MyHC staining images/24 h sXBP1 OE Representative image.tif]

## Slide 1
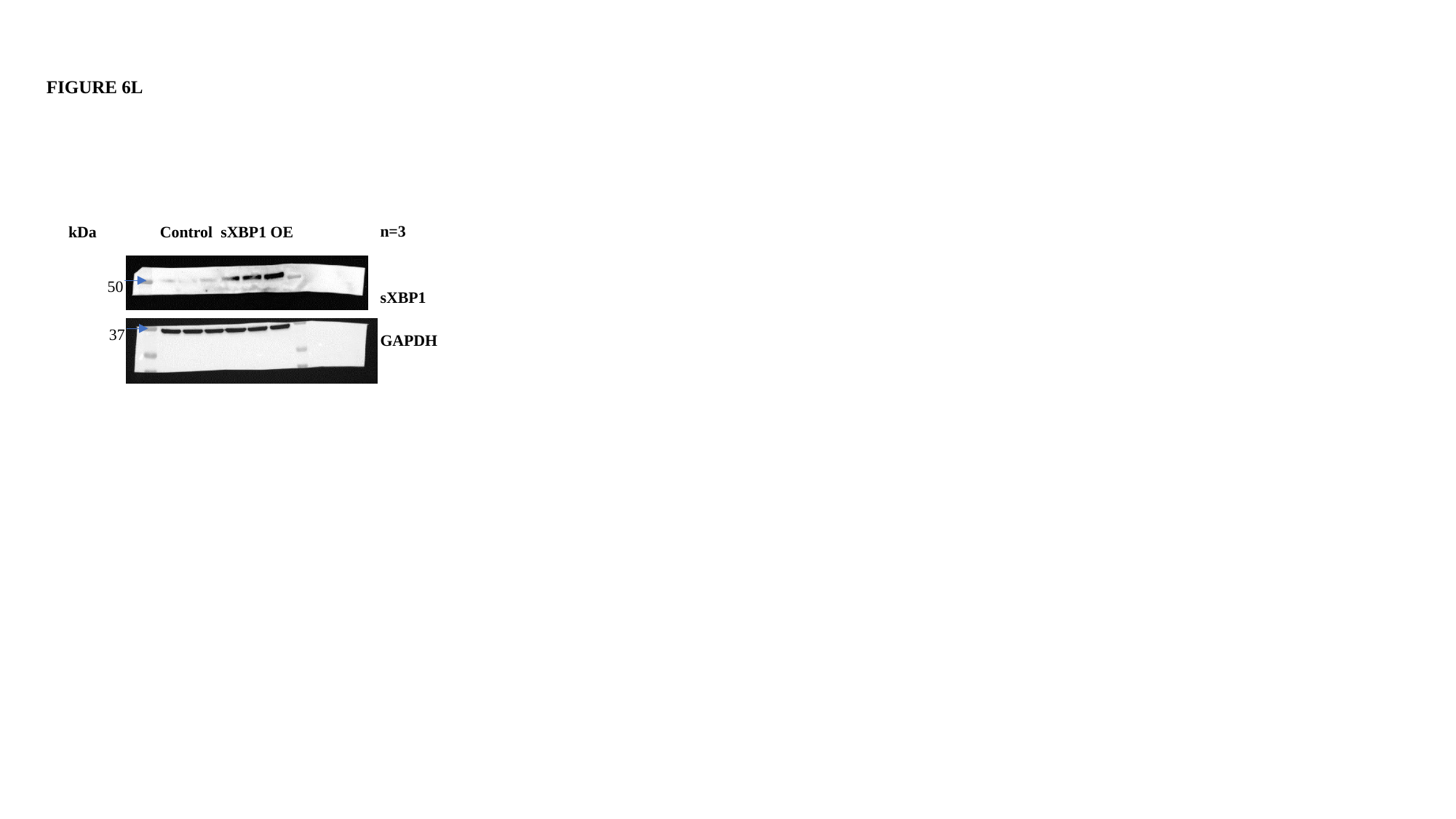

FIGURE 6L
n=3
kDa
Control
sXBP1 OE
50
sXBP1
37
GAPDH

Supplement: Supplementary file 9 — Source data Fig. 6 [file 44319_2024_197_MOESM9_ESM.zip › Figure 6/6I-L/6L/Western blot with annotation.pptx]

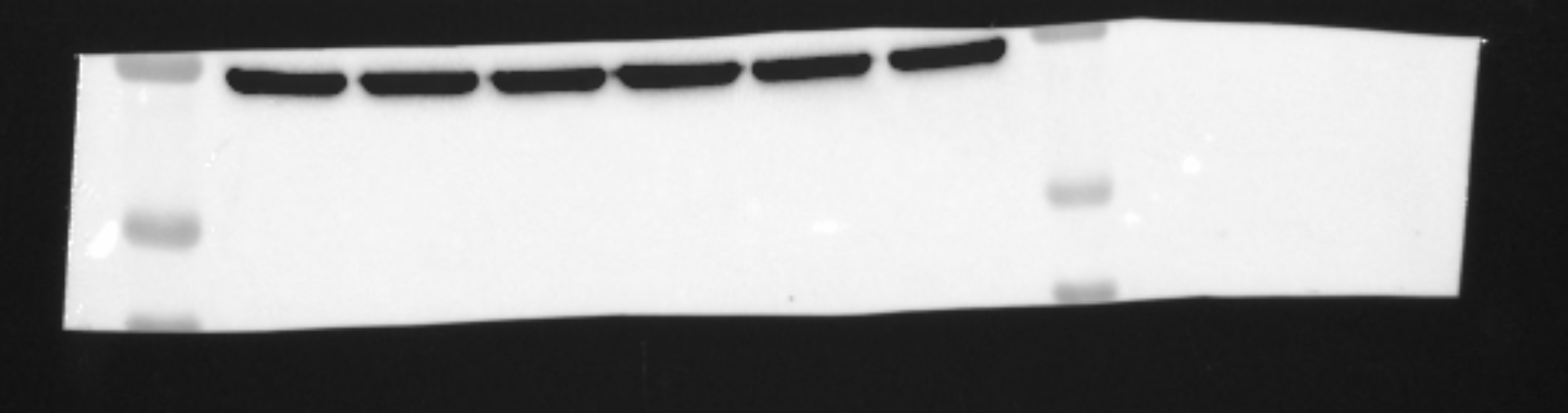

Supplement: Supplementary file 9 — Source data Fig. 6 [file 44319_2024_197_MOESM9_ESM.zip › Figure 6/6I-L/6L/Western-GAPDH.tif]

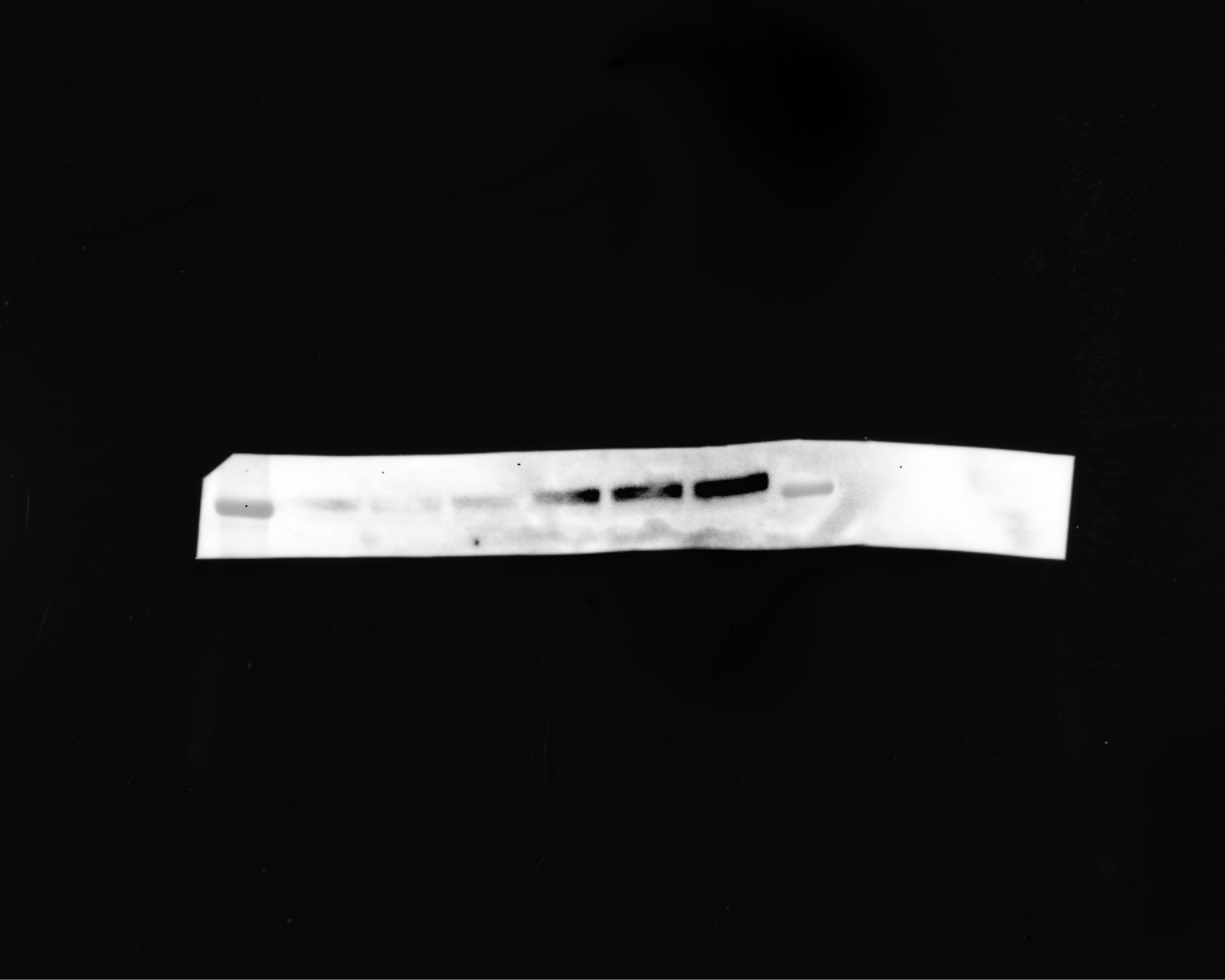

Supplement: Supplementary file 9 — Source data Fig. 6 [file 44319_2024_197_MOESM9_ESM.zip › Figure 6/6I-L/6L/Western-sXBP1.tif]

## Slide 1
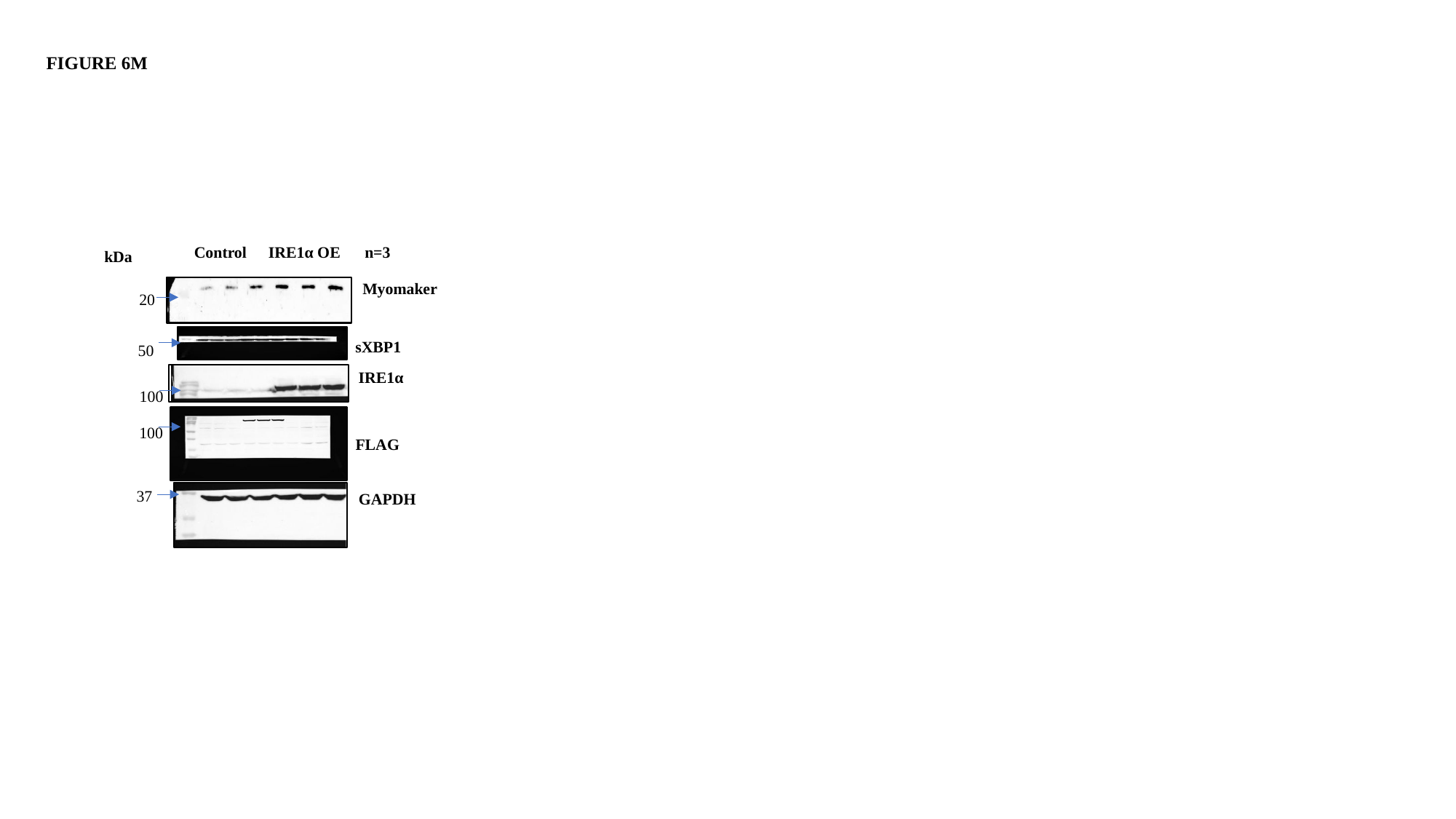

FIGURE 6M
Control
IRE1α OE
n=3
kDa
Myomaker
20
sXBP1
50
IRE1α
100
100
FLAG
37
GAPDH

Supplement: Supplementary file 9 — Source data Fig. 6 [file 44319_2024_197_MOESM9_ESM.zip › Figure 6/6M/Western blot with annotation.pptx]

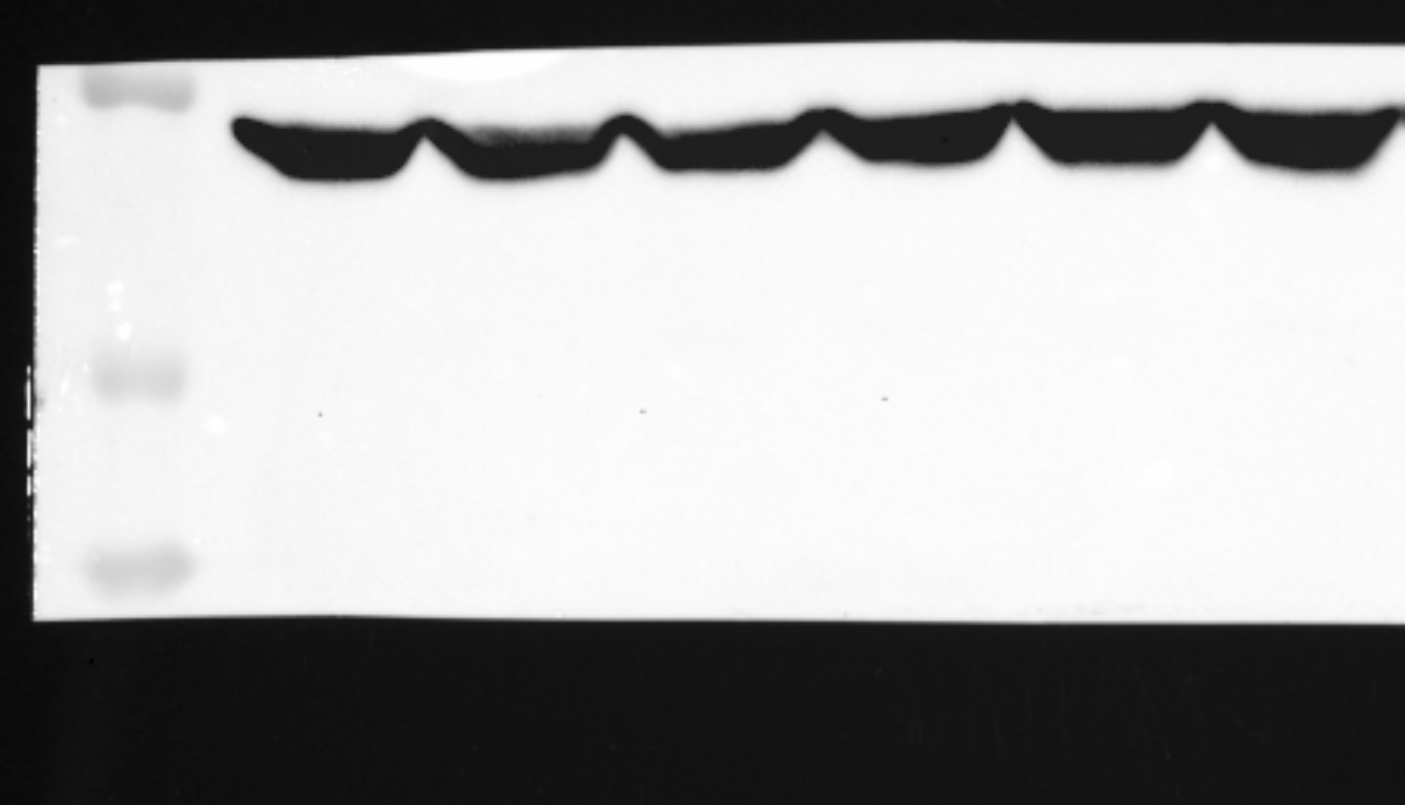

Supplement: Supplementary file 9 — Source data Fig. 6 [file 44319_2024_197_MOESM9_ESM.zip › Figure 6/6M/Western-GAPDH.tif]

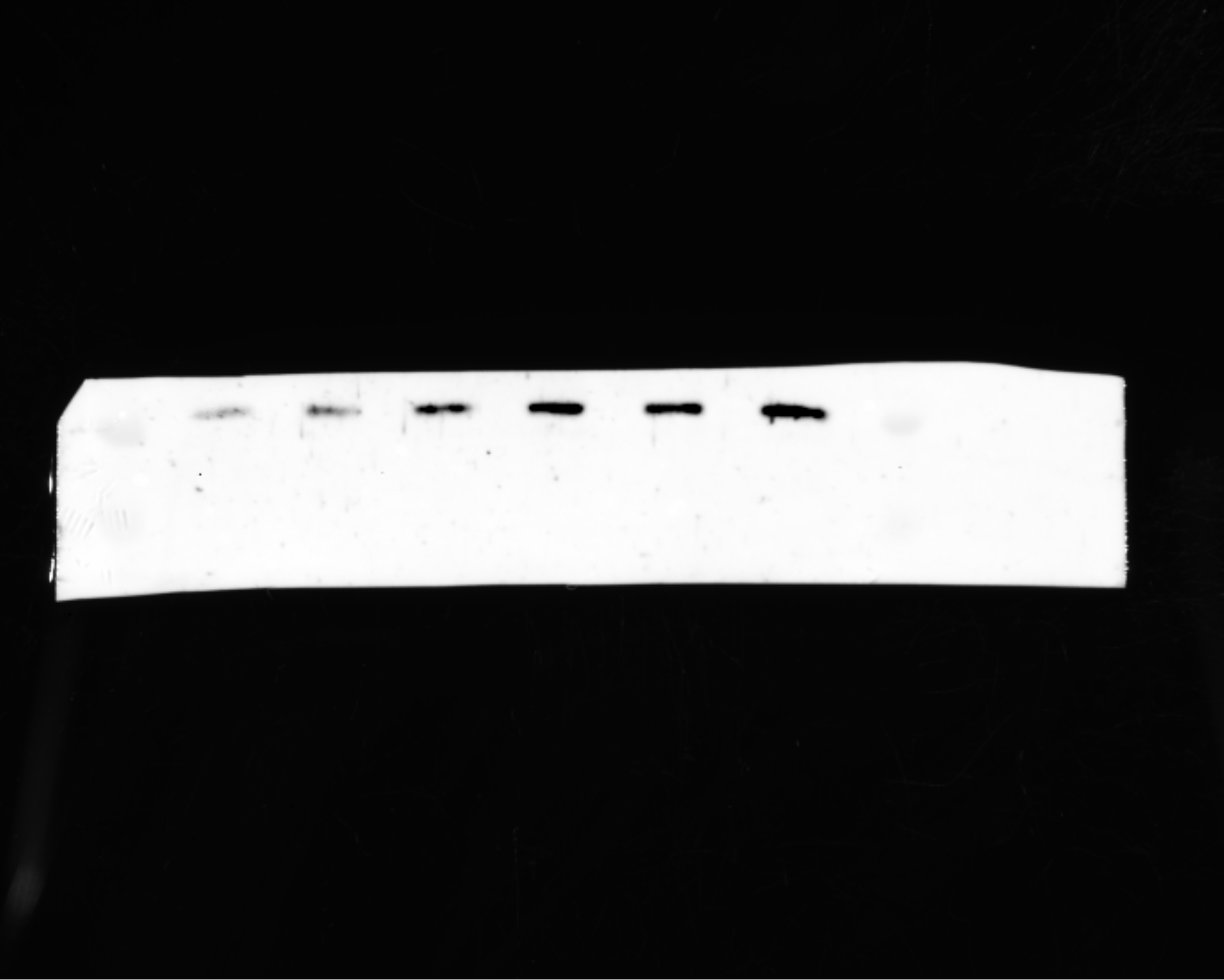

Supplement: Supplementary file 9 — Source data Fig. 6 [file 44319_2024_197_MOESM9_ESM.zip › Figure 6/6M/Western-Myomaker.tif]

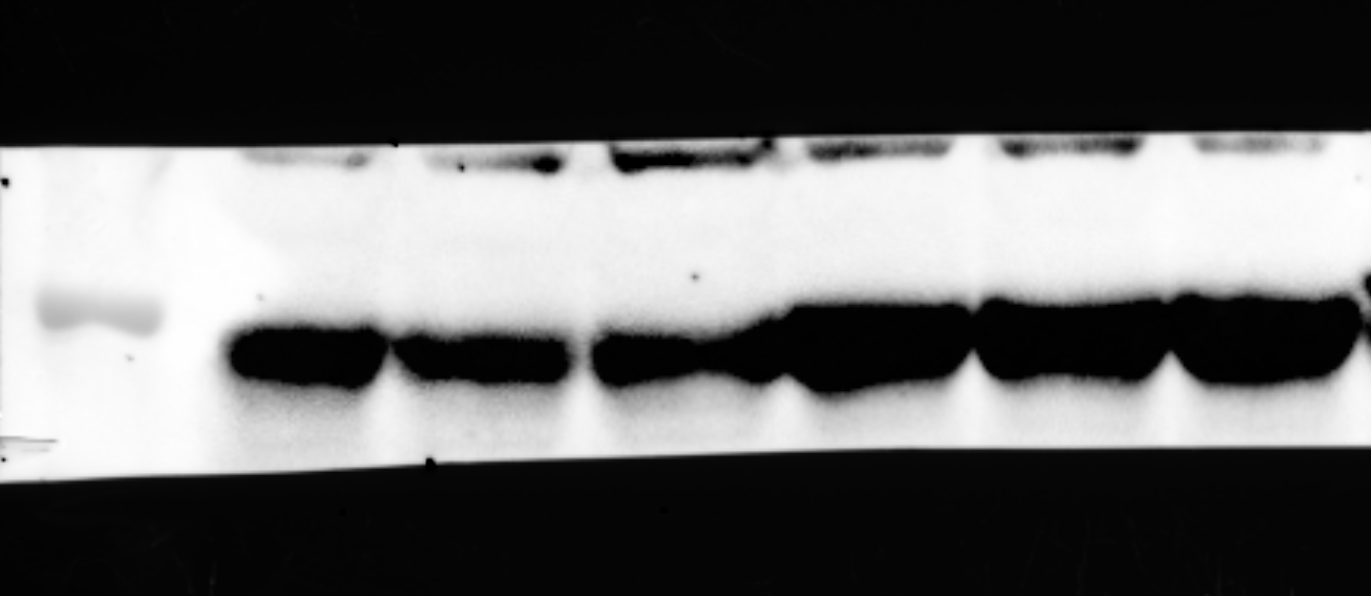

Supplement: Supplementary file 9 — Source data Fig. 6 [file 44319_2024_197_MOESM9_ESM.zip › Figure 6/6M/Western-sXBP1.tif]

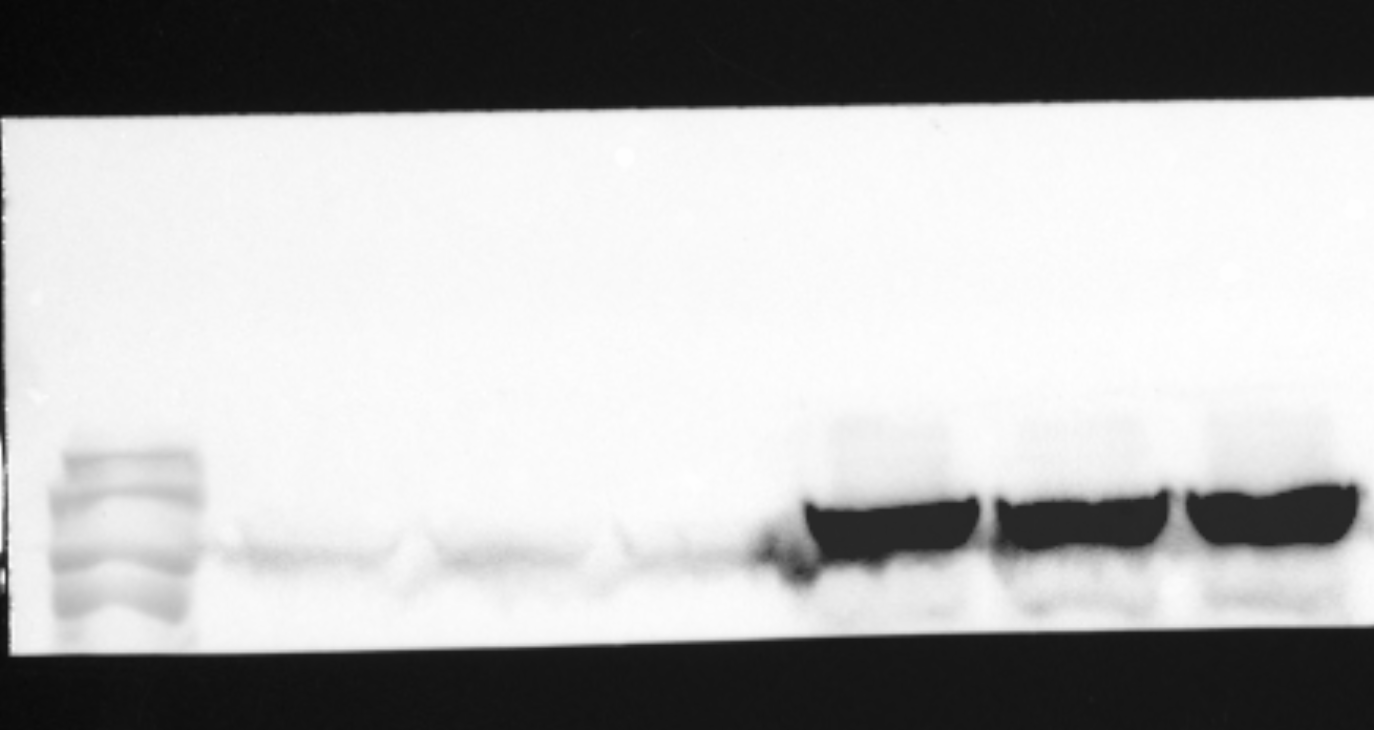

Supplement: Supplementary file 9 — Source data Fig. 6 [file 44319_2024_197_MOESM9_ESM.zip › Figure 6/6M/Western-Total IRE1a.tif]

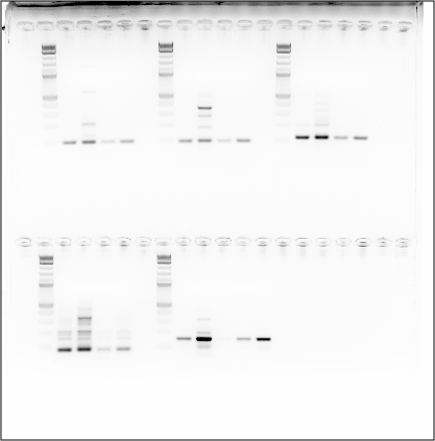

Supplement: Supplementary file 10 — Source data Fig. 7 [file 44319_2024_197_MOESM10_ESM.zip › Figure 7/7C/ChIP PCR Agarose gel image.tif]

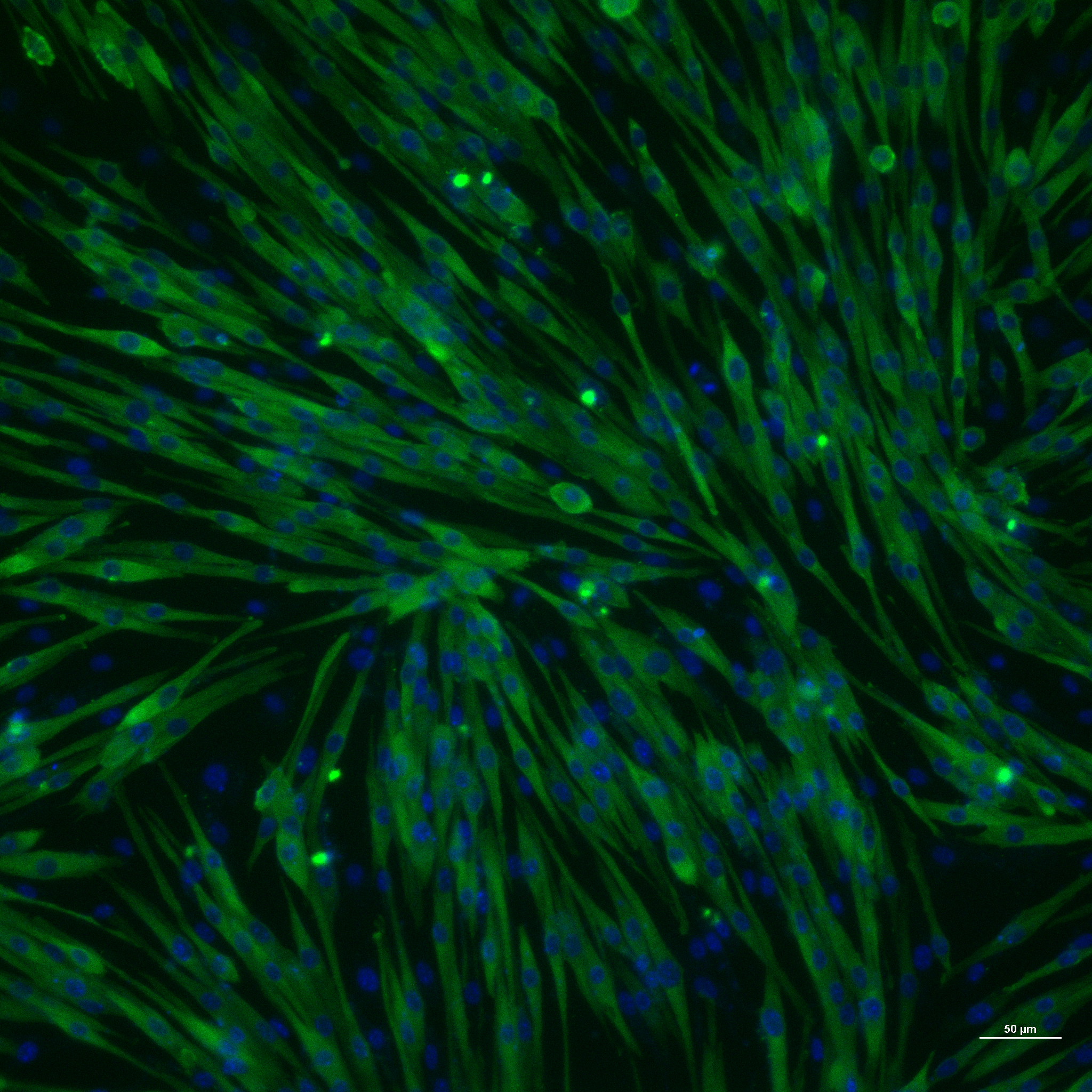

Supplement: Supplementary file 10 — Source data Fig. 7 [file 44319_2024_197_MOESM10_ESM.zip › Figure 7/7F/IRE1 shRNA-Mymk OE_MyHC images/IRE1a shRNA-Mymk cDNA replicate 2.tif]

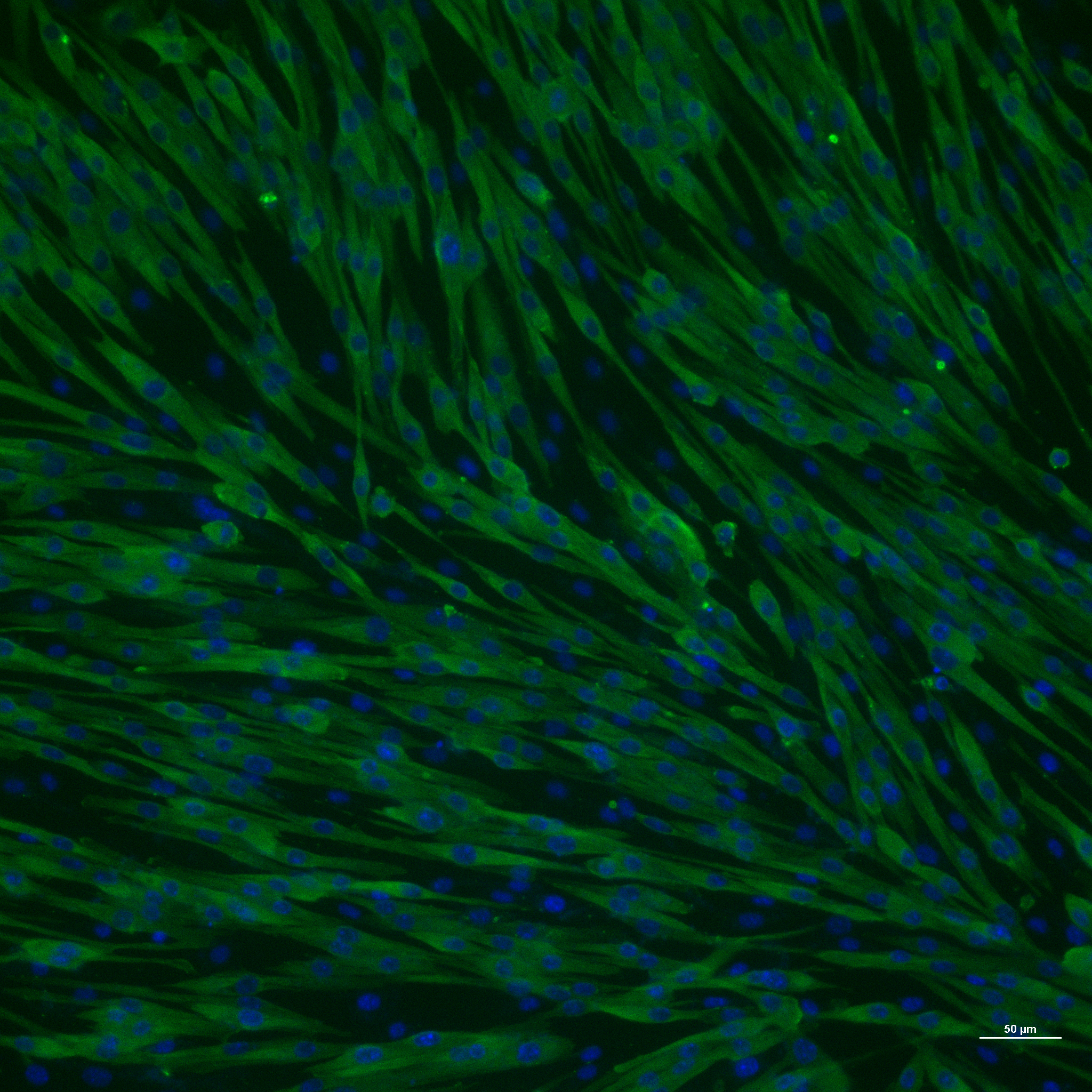

Supplement: Supplementary file 10 — Source data Fig. 7 [file 44319_2024_197_MOESM10_ESM.zip › Figure 7/7F/IRE1 shRNA-Mymk OE_MyHC images/IRE1a shRNA-Mymk cDNA replicate 3.tif]

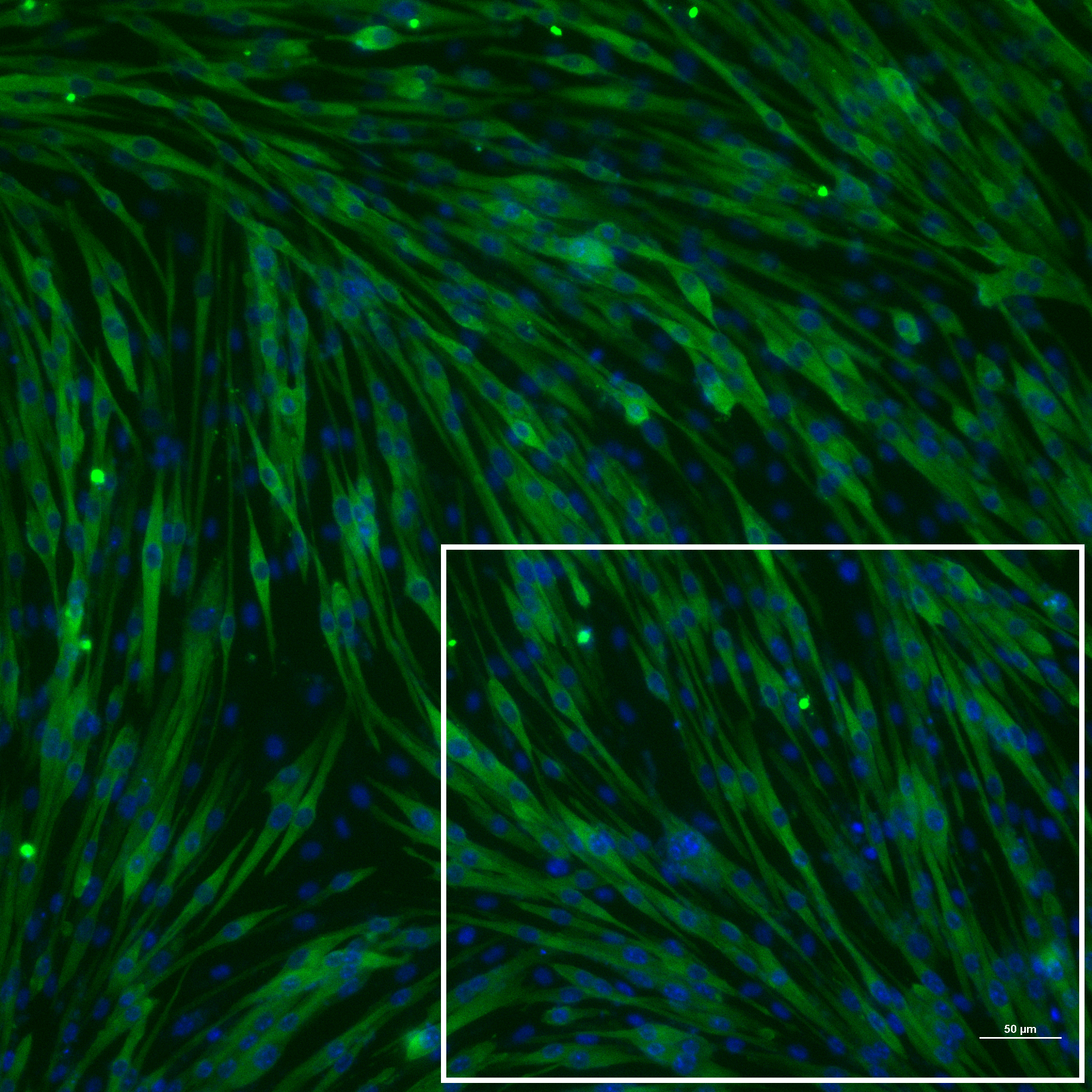

Supplement: Supplementary file 10 — Source data Fig. 7 [file 44319_2024_197_MOESM10_ESM.zip › Figure 7/7F/IRE1 shRNA-Mymk OE_MyHC images/IRE1a shRNA-Mymk cDNA Representative image with box.tiff]

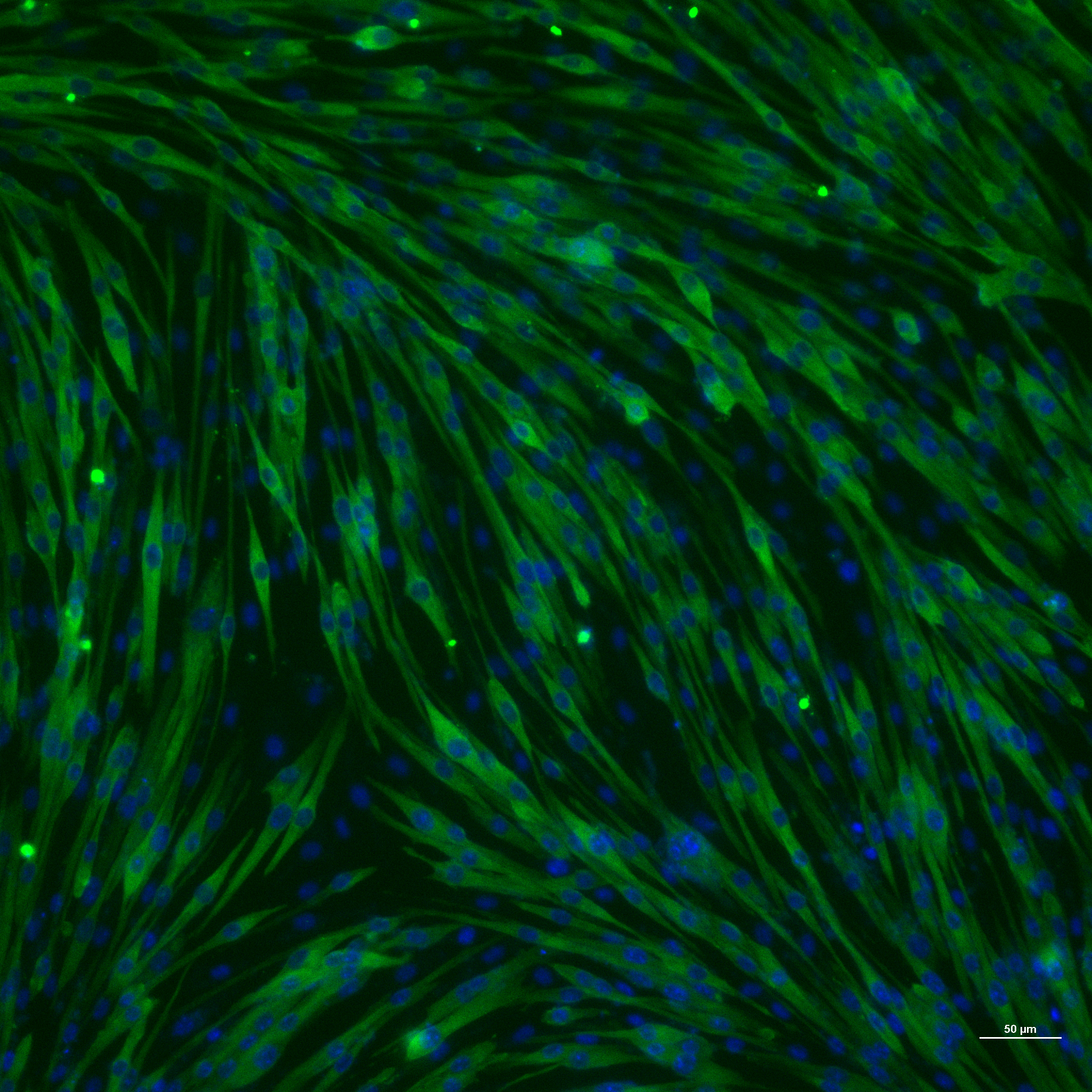

Supplement: Supplementary file 10 — Source data Fig. 7 [file 44319_2024_197_MOESM10_ESM.zip › Figure 7/7F/IRE1 shRNA-Mymk OE_MyHC images/IRE1a shRNA-Mymk cDNA Representative image.tif]

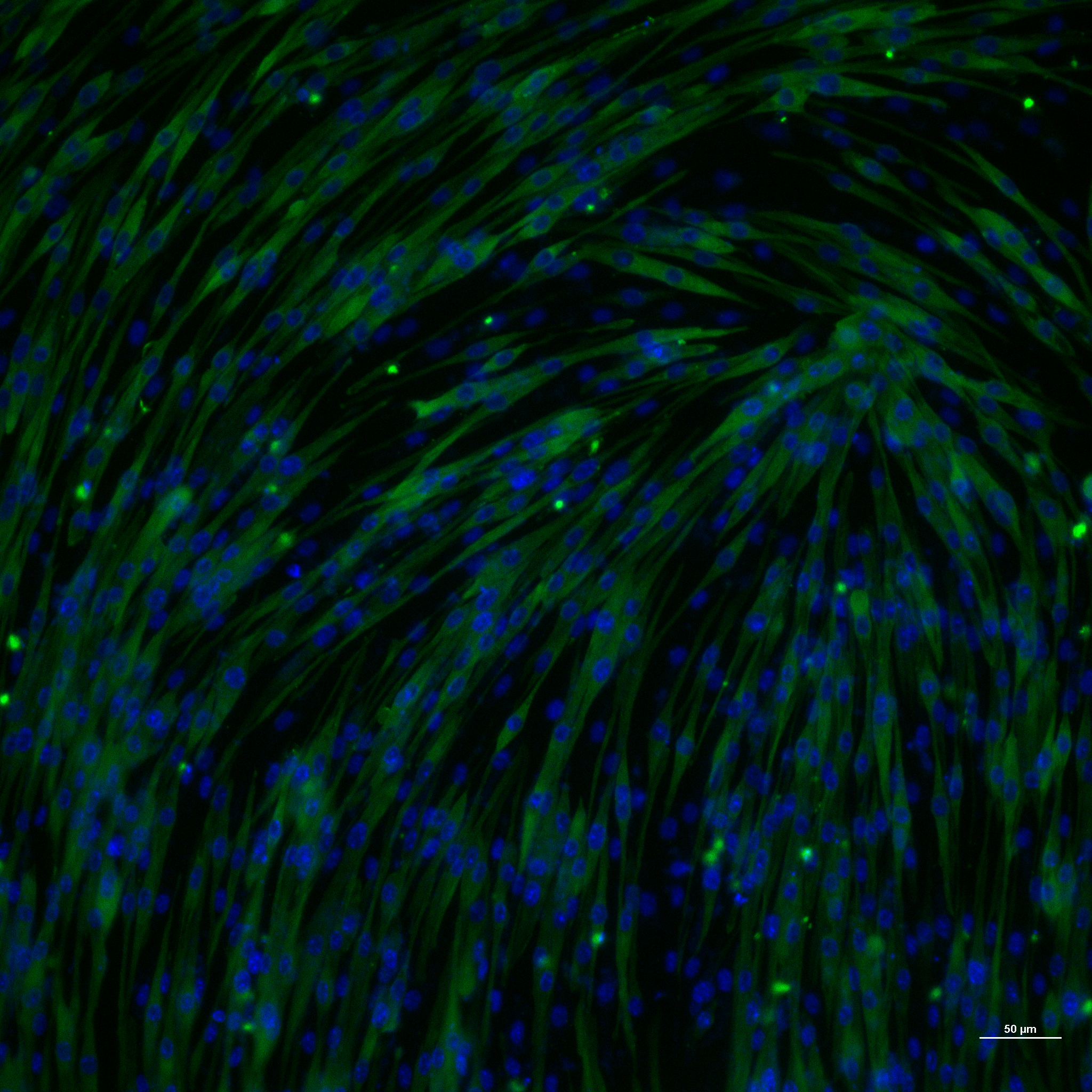

Supplement: Supplementary file 10 — Source data Fig. 7 [file 44319_2024_197_MOESM10_ESM.zip › Figure 7/7F/IRE1 shRNA-Mymk OE_MyHC images/IRE1a shRNA-vector alone replicate 2.tif]

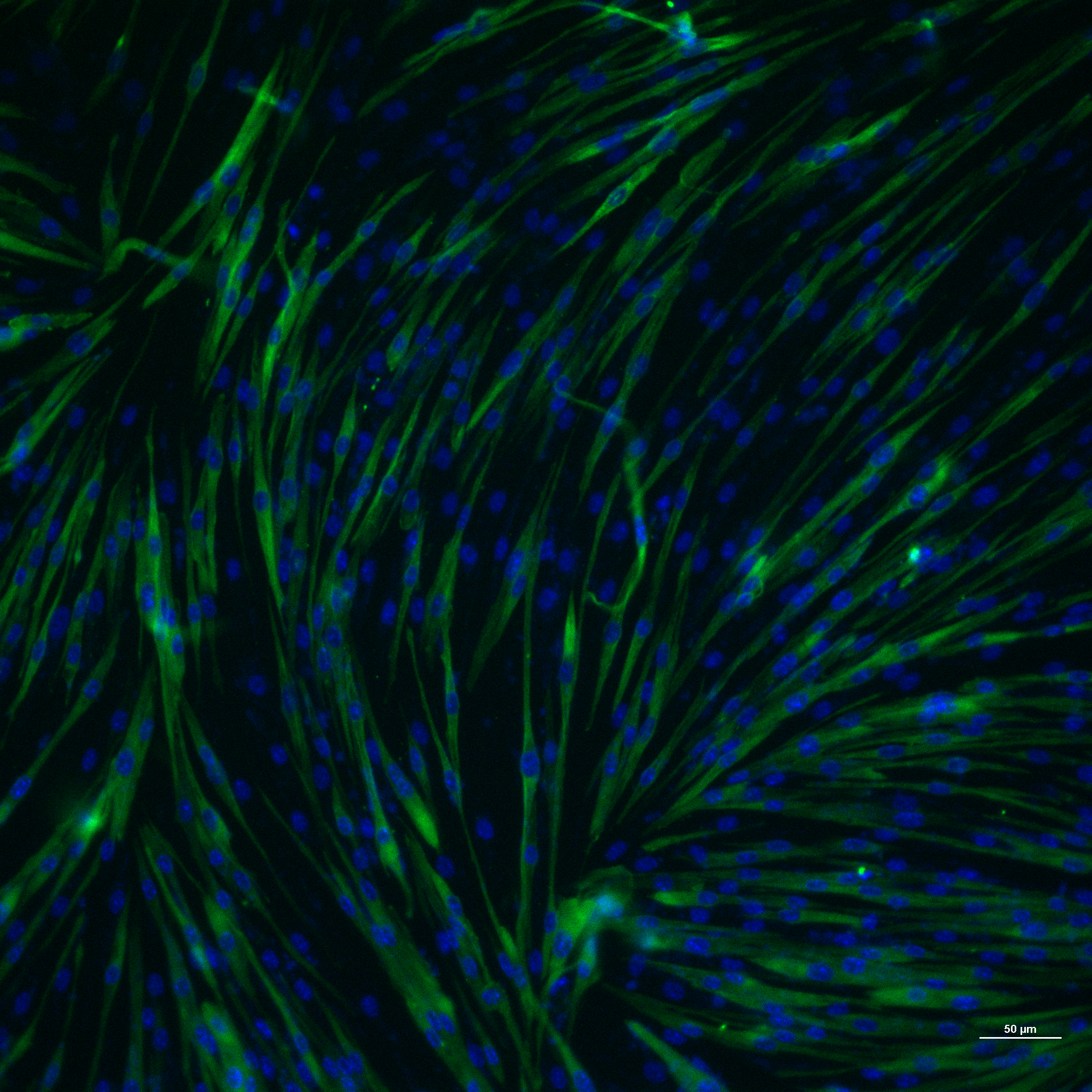

Supplement: Supplementary file 10 — Source data Fig. 7 [file 44319_2024_197_MOESM10_ESM.zip › Figure 7/7F/IRE1 shRNA-Mymk OE_MyHC images/IRE1a shRNA-vector alone replicate 3.tif]

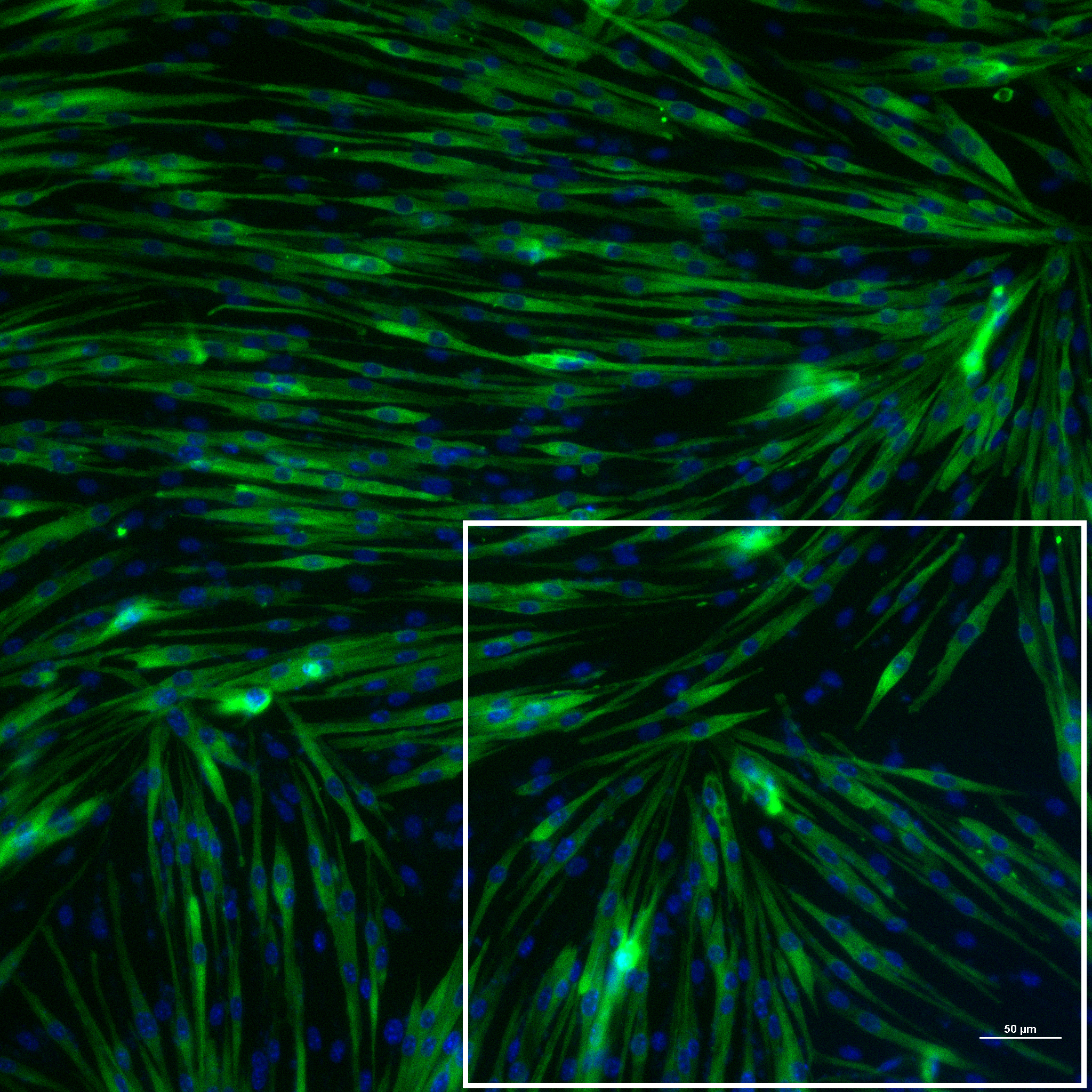

Supplement: Supplementary file 10 — Source data Fig. 7 [file 44319_2024_197_MOESM10_ESM.zip › Figure 7/7F/IRE1 shRNA-Mymk OE_MyHC images/IRE1a shRNA-vector alone Representative image with box.tiff]

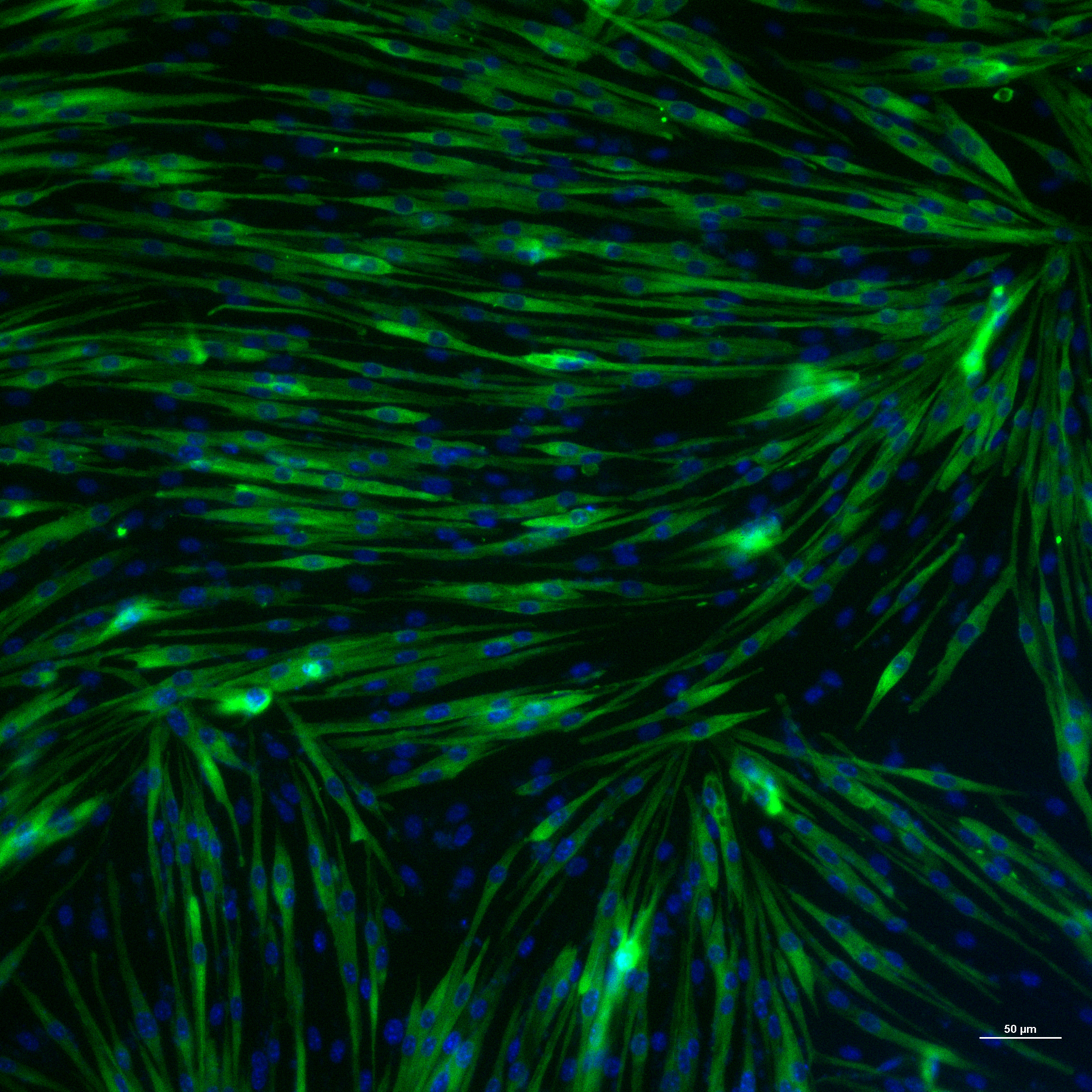

Supplement: Supplementary file 10 — Source data Fig. 7 [file 44319_2024_197_MOESM10_ESM.zip › Figure 7/7F/IRE1 shRNA-Mymk OE_MyHC images/IRE1a shRNA-vector alone Representative image.tif]

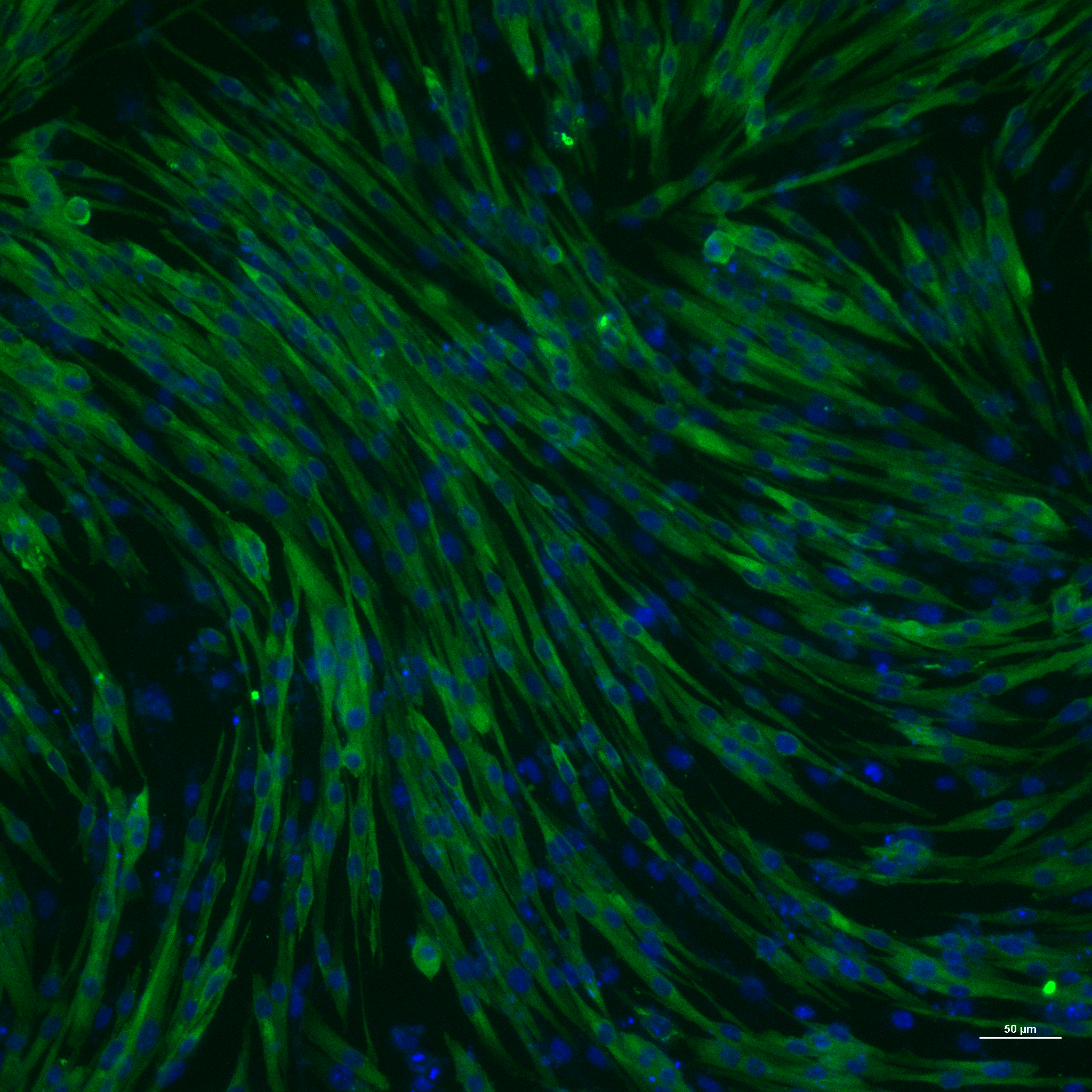

Supplement: Supplementary file 10 — Source data Fig. 7 [file 44319_2024_197_MOESM10_ESM.zip › Figure 7/7F/IRE1 shRNA-Mymk OE_MyHC images/Scrambled shRNA-Mymk cDNA replicate 2.tif]

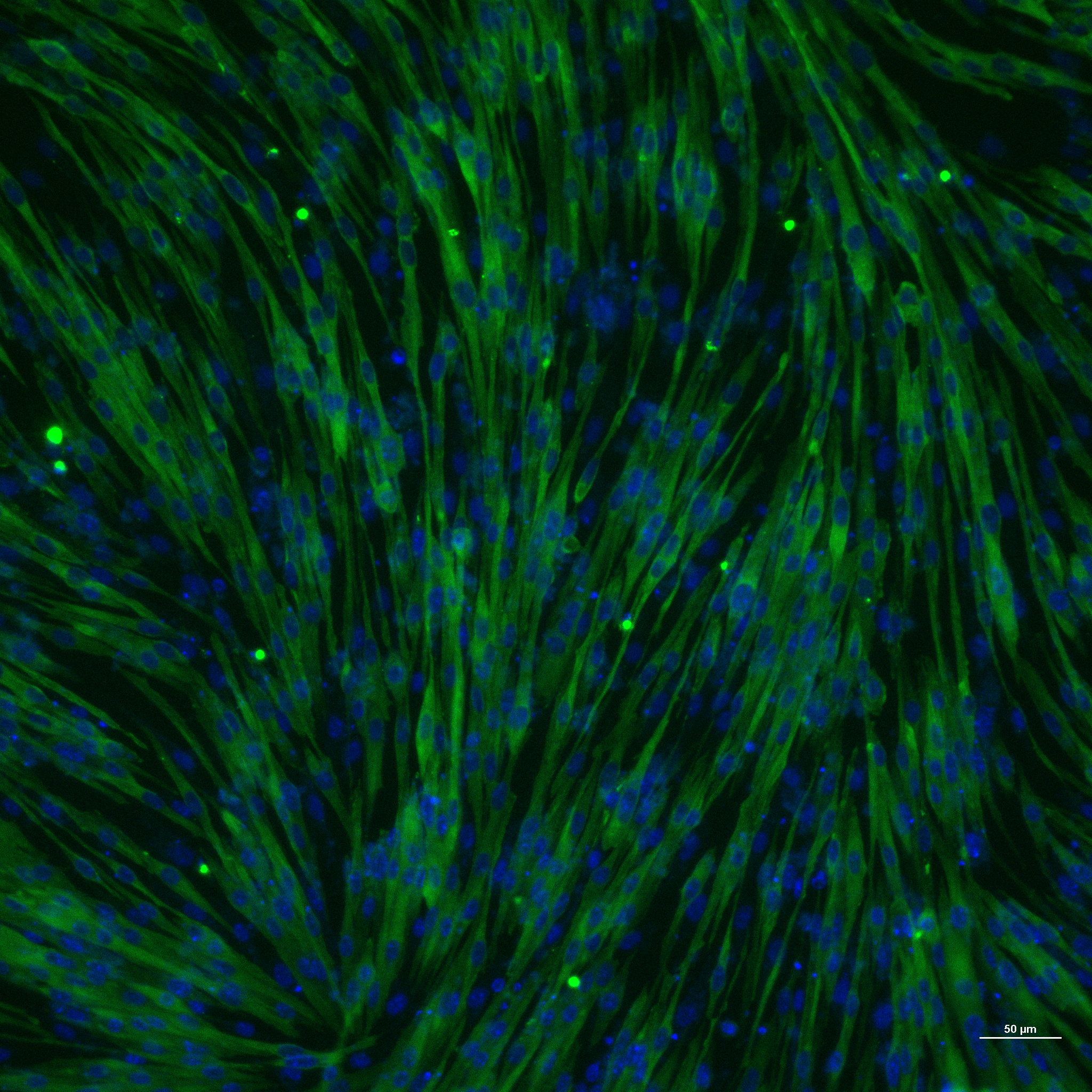

Supplement: Supplementary file 10 — Source data Fig. 7 [file 44319_2024_197_MOESM10_ESM.zip › Figure 7/7F/IRE1 shRNA-Mymk OE_MyHC images/Scrambled shRNA-Mymk cDNA replicate 3.tif]

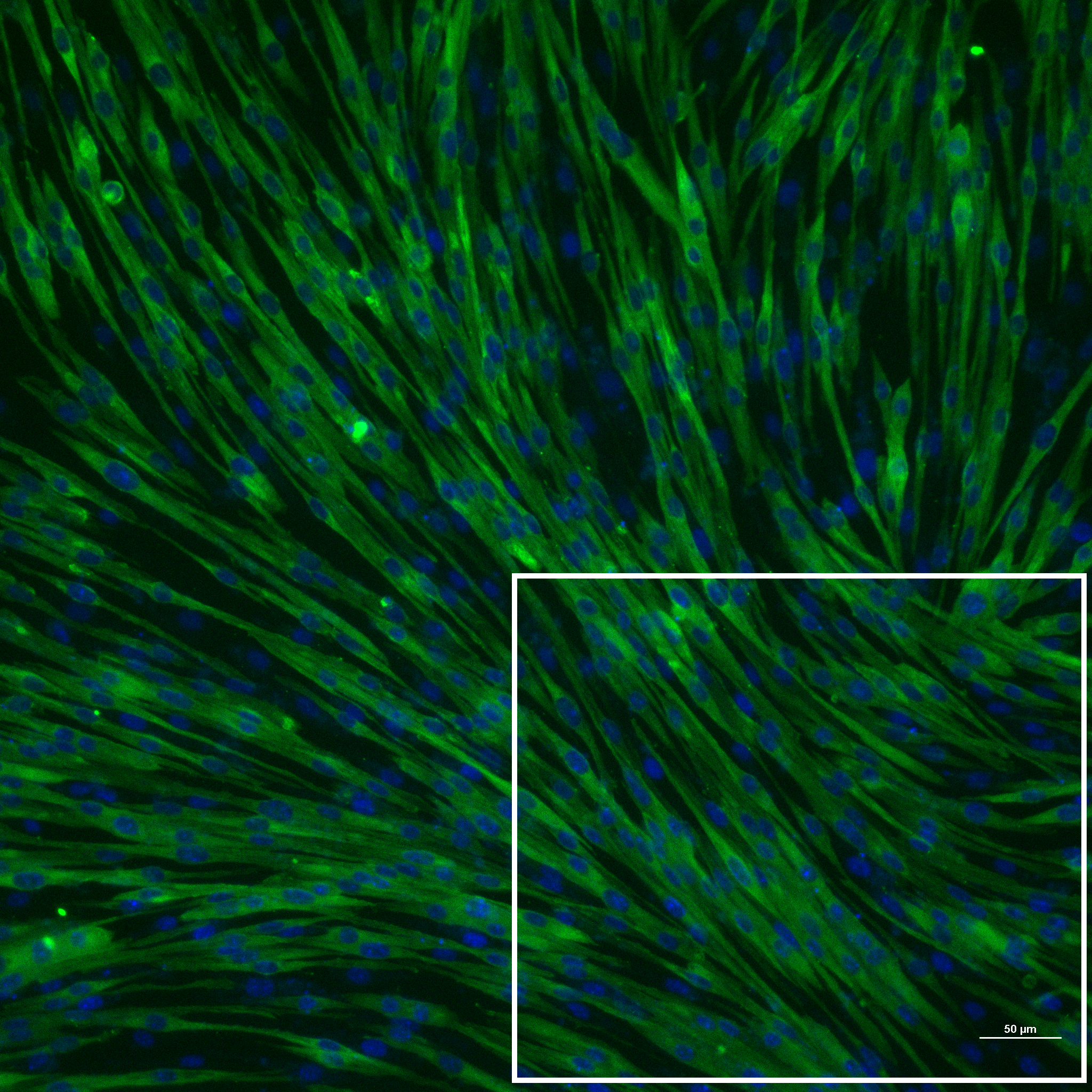

Supplement: Supplementary file 10 — Source data Fig. 7 [file 44319_2024_197_MOESM10_ESM.zip › Figure 7/7F/IRE1 shRNA-Mymk OE_MyHC images/Scrambled shRNA-Mymk cDNA Representative image with box.tiff]

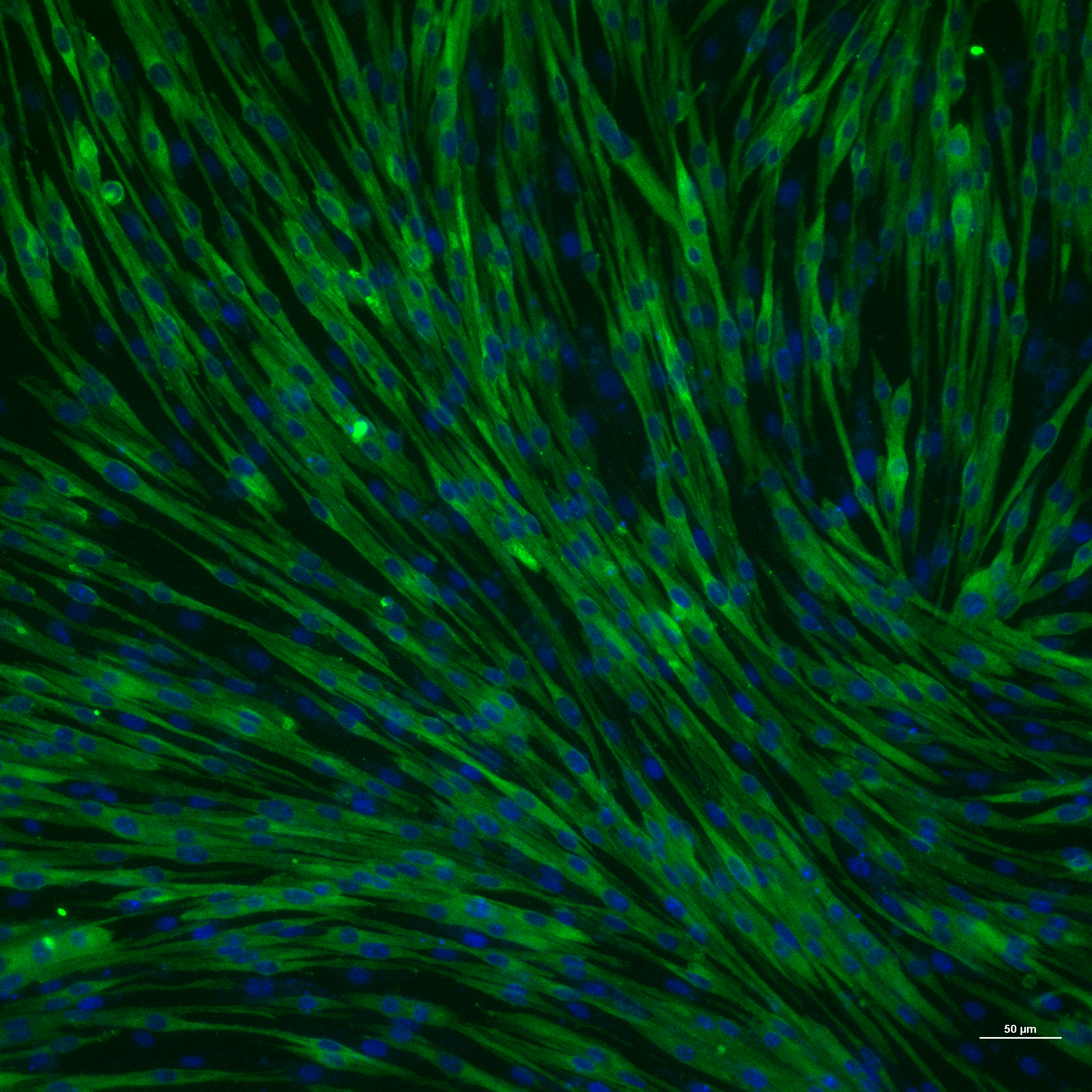

Supplement: Supplementary file 10 — Source data Fig. 7 [file 44319_2024_197_MOESM10_ESM.zip › Figure 7/7F/IRE1 shRNA-Mymk OE_MyHC images/Scrambled shRNA-Mymk cDNA Representative image.tif]

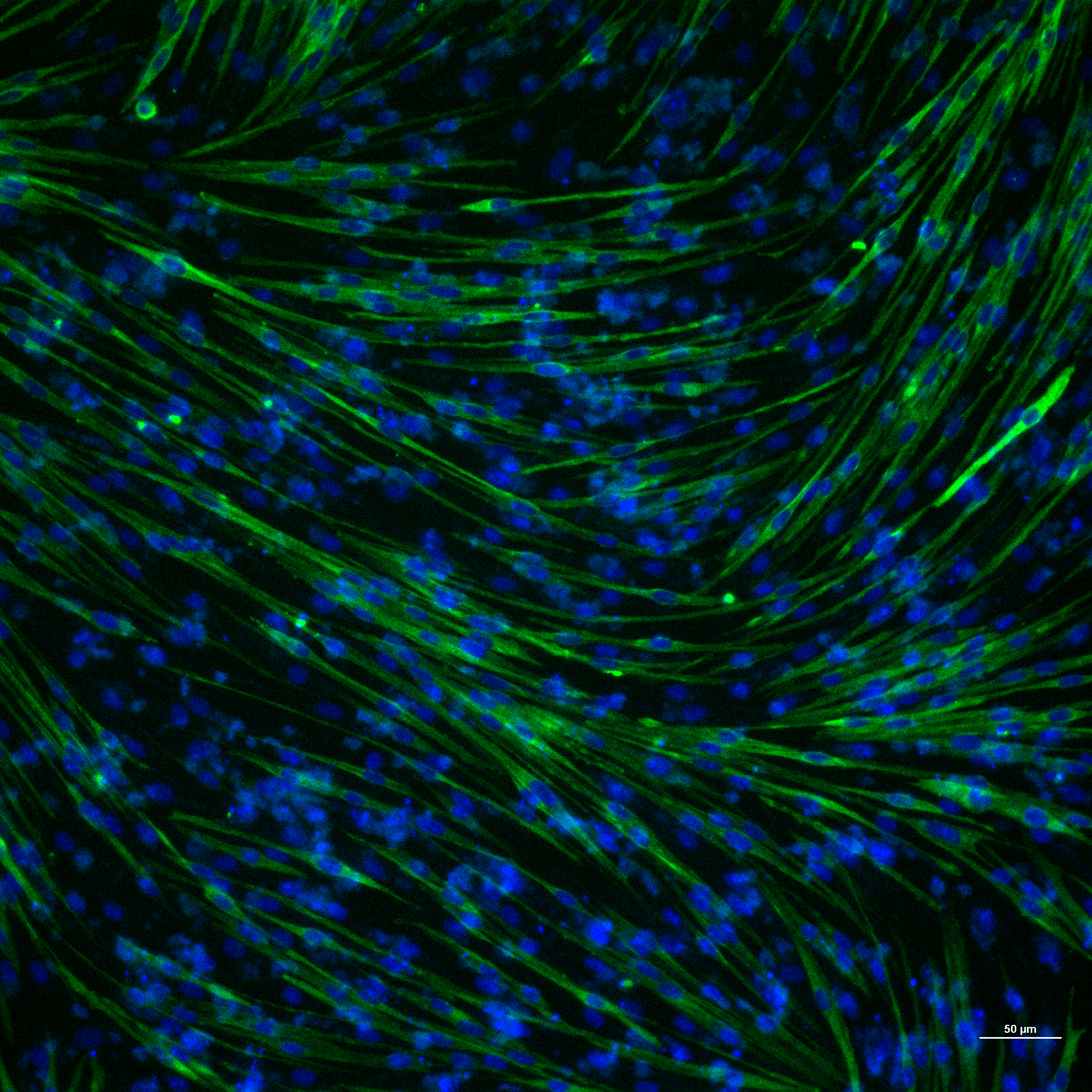

Supplement: Supplementary file 10 — Source data Fig. 7 [file 44319_2024_197_MOESM10_ESM.zip › Figure 7/7F/IRE1 shRNA-Mymk OE_MyHC images/Scrambled shRNA-vector alone replicate 2.tif]

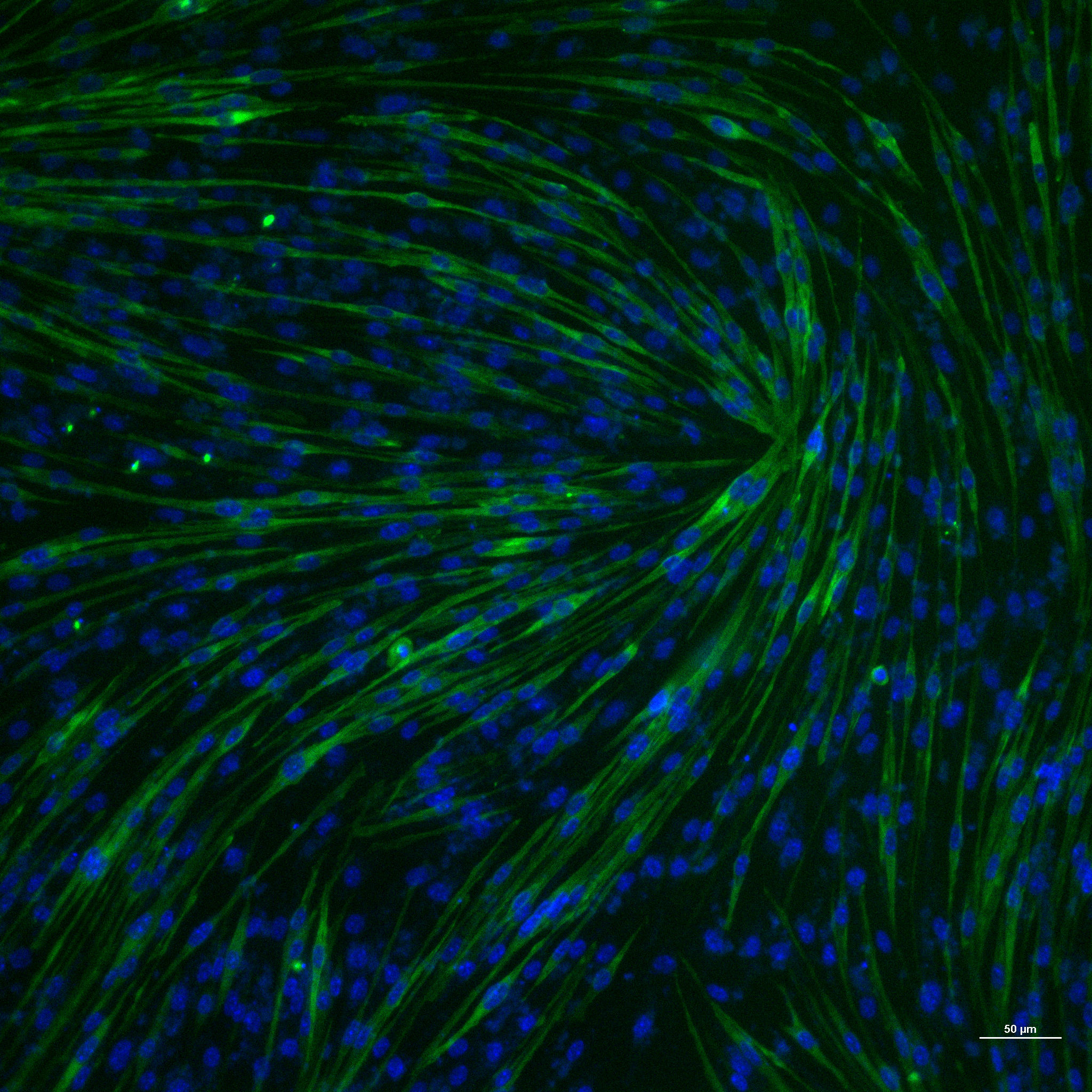

Supplement: Supplementary file 10 — Source data Fig. 7 [file 44319_2024_197_MOESM10_ESM.zip › Figure 7/7F/IRE1 shRNA-Mymk OE_MyHC images/Scrambled shRNA-vector alone replicate 3.tif]

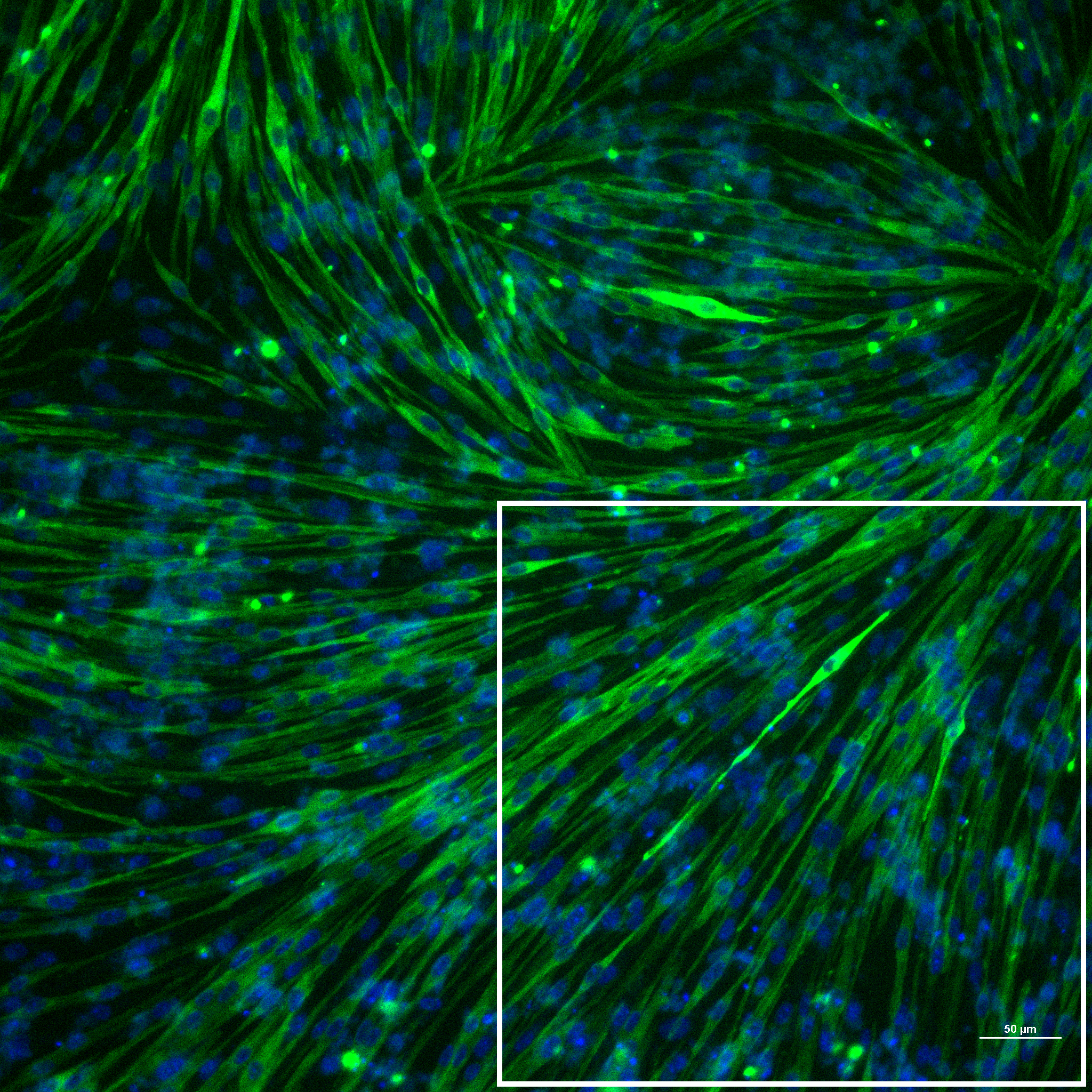

Supplement: Supplementary file 10 — Source data Fig. 7 [file 44319_2024_197_MOESM10_ESM.zip › Figure 7/7F/IRE1 shRNA-Mymk OE_MyHC images/Scrambled shRNA-vector alone Representative image with box.tiff]

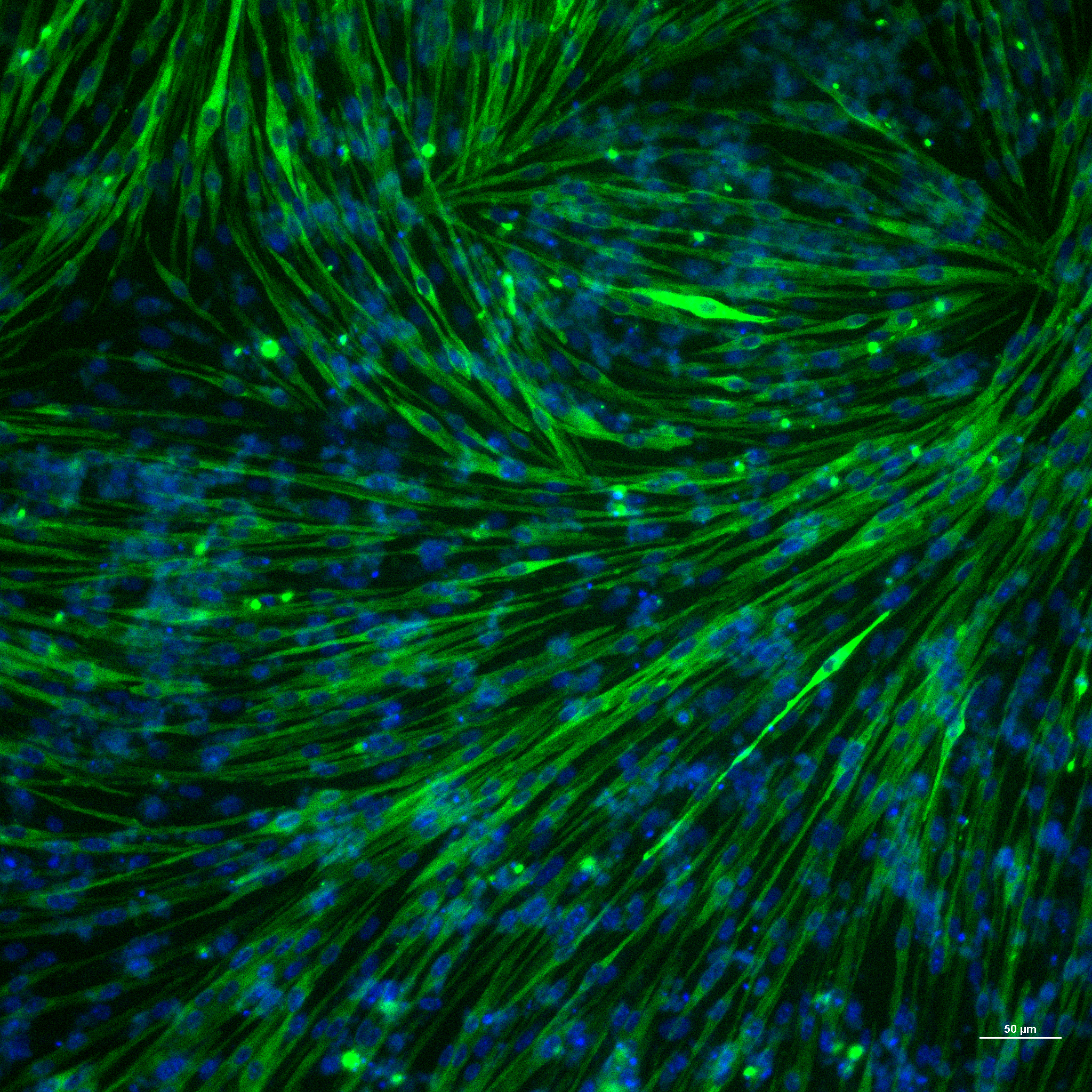

Supplement: Supplementary file 10 — Source data Fig. 7 [file 44319_2024_197_MOESM10_ESM.zip › Figure 7/7F/IRE1 shRNA-Mymk OE_MyHC images/Scrambled shRNA-vector alone Representative image.tif]

## Slide 1
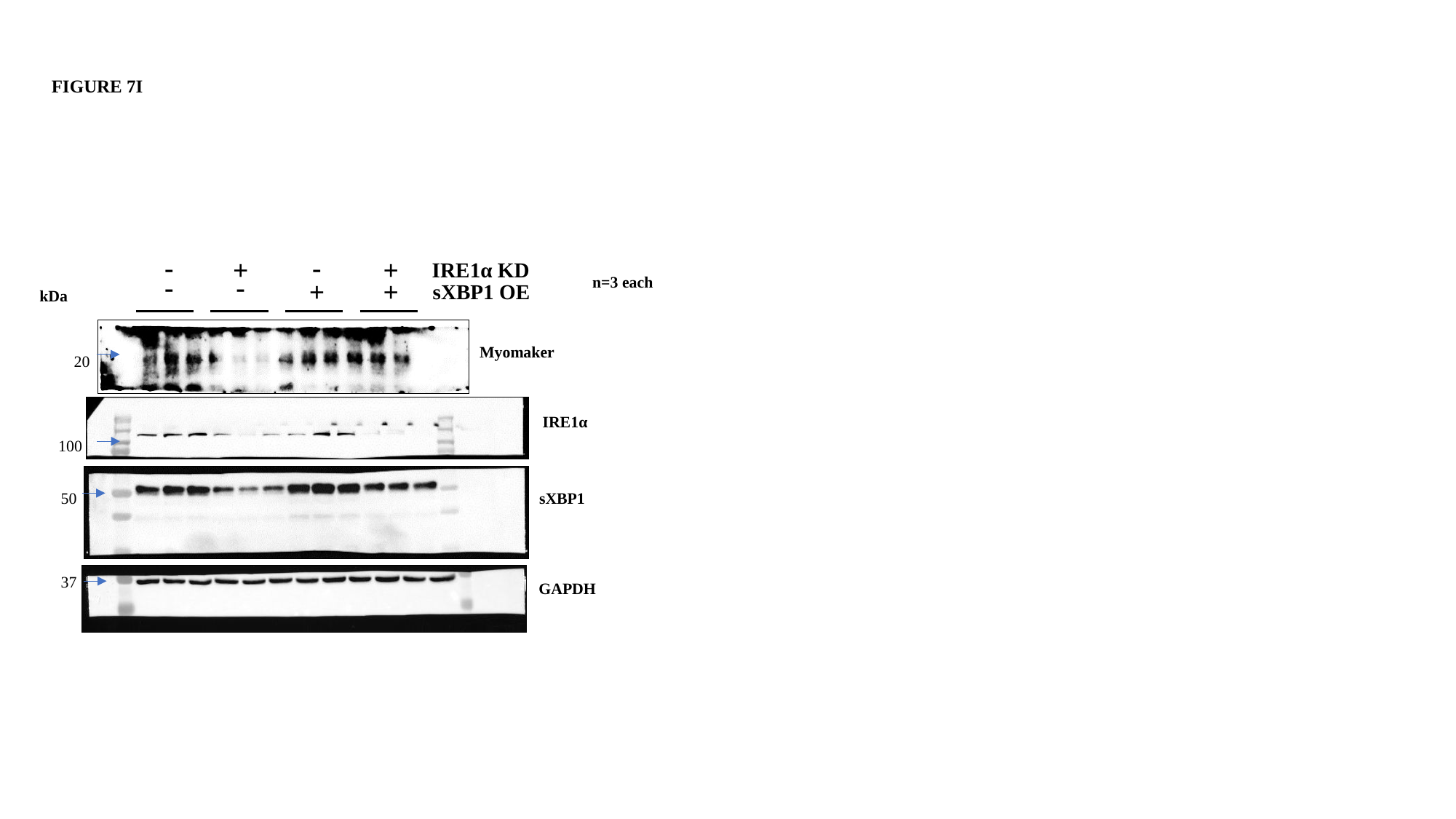

FIGURE 7I
-
-
+
+
IRE1α KD
-
-
n=3 each
+
+
sXBP1 OE
kDa
Myomaker
20
IRE1α
100
sXBP1
50
37
GAPDH

Supplement: Supplementary file 10 — Source data Fig. 7 [file 44319_2024_197_MOESM10_ESM.zip › Figure 7/7I-J/7I/Western blot with annotation.pptx]

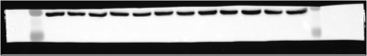

Supplement: Supplementary file 10 — Source data Fig. 7 [file 44319_2024_197_MOESM10_ESM.zip › Figure 7/7I-J/7I/Western-GAPDH.tif]

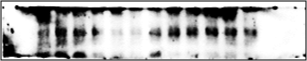

Supplement: Supplementary file 10 — Source data Fig. 7 [file 44319_2024_197_MOESM10_ESM.zip › Figure 7/7I-J/7I/Western-Myomaker.tif]

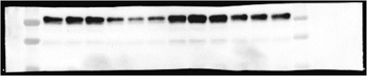

Supplement: Supplementary file 10 — Source data Fig. 7 [file 44319_2024_197_MOESM10_ESM.zip › Figure 7/7I-J/7I/Western-sXBP1.tif]

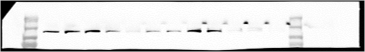

Supplement: Supplementary file 10 — Source data Fig. 7 [file 44319_2024_197_MOESM10_ESM.zip › Figure 7/7I-J/7I/Western-Total IRE1a.tif]

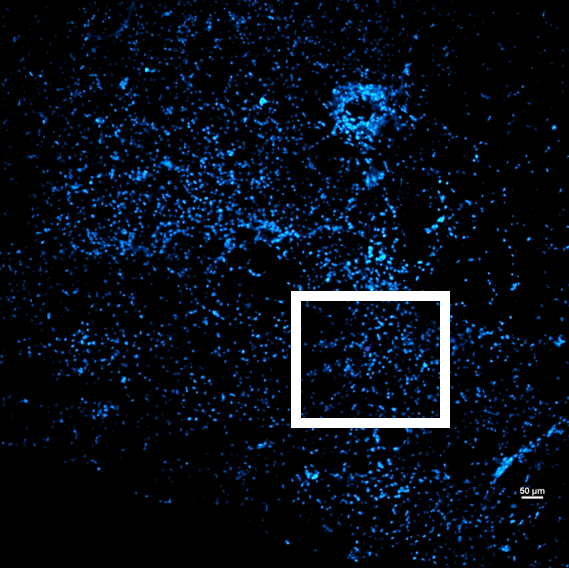

Supplement: Supplementary file 12 — Figure EV1 Source Data [file 44319_2024_197_MOESM12_ESM.zip › Figure EV1/EV1A/Ern1 flfl-DAPI-Representative image with box.tiff]

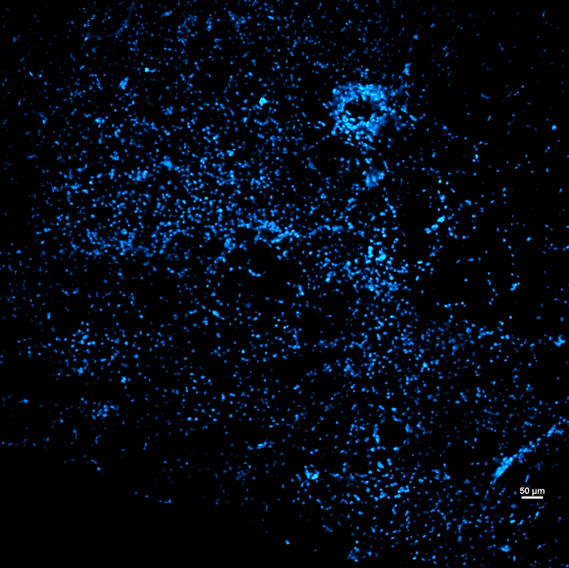

Supplement: Supplementary file 12 — Figure EV1 Source Data [file 44319_2024_197_MOESM12_ESM.zip › Figure EV1/EV1A/Ern1 flfl-DAPI-Representative image.tif]

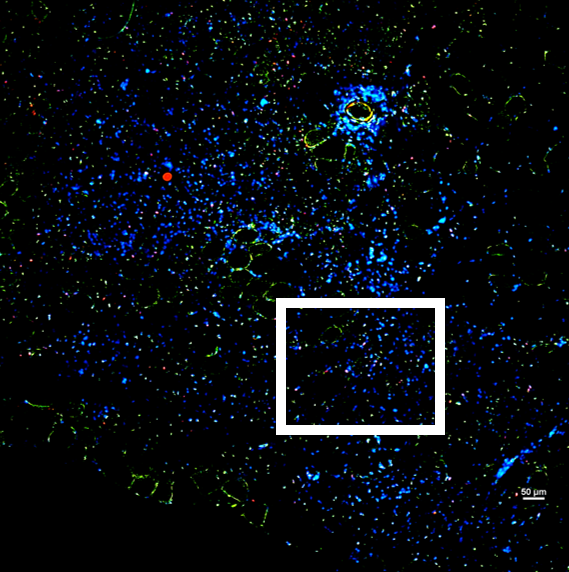

Supplement: Supplementary file 12 — Figure EV1 Source Data [file 44319_2024_197_MOESM12_ESM.zip › Figure EV1/EV1A/Ern1 flfl-Merged-Representative image with box.tiff]

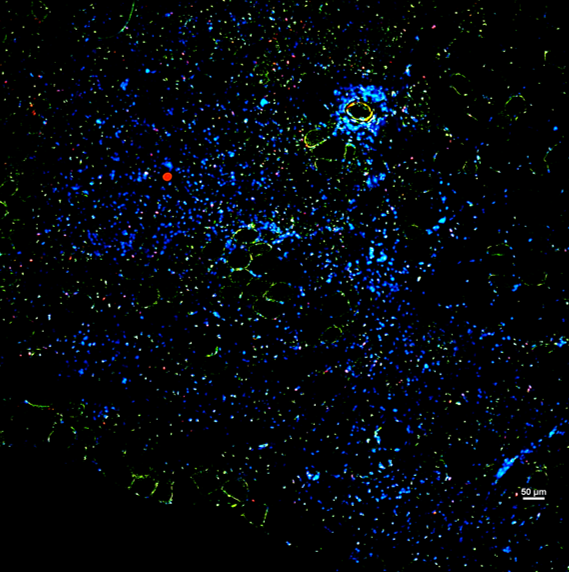

Supplement: Supplementary file 12 — Figure EV1 Source Data [file 44319_2024_197_MOESM12_ESM.zip › Figure EV1/EV1A/Ern1 flfl-Merged-Representative image.tif]

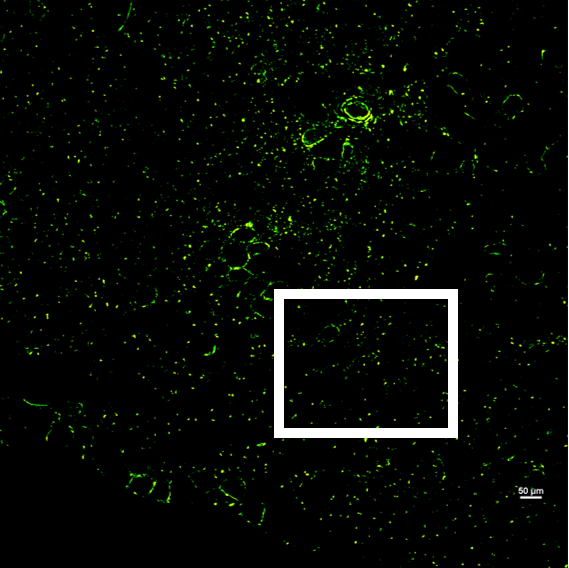

Supplement: Supplementary file 12 — Figure EV1 Source Data [file 44319_2024_197_MOESM12_ESM.zip › Figure EV1/EV1A/Ern1 flfl-p-IRE1a-Representative image with box.tiff]

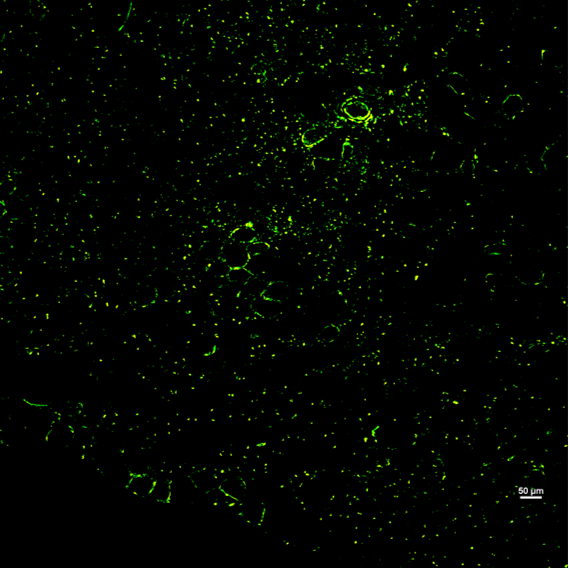

Supplement: Supplementary file 12 — Figure EV1 Source Data [file 44319_2024_197_MOESM12_ESM.zip › Figure EV1/EV1A/Ern1 flfl-p-IRE1a-Representative image.tif]

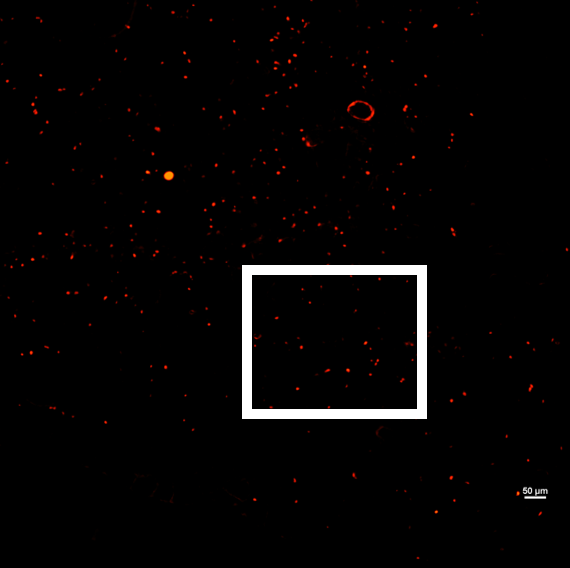

Supplement: Supplementary file 12 — Figure EV1 Source Data [file 44319_2024_197_MOESM12_ESM.zip › Figure EV1/EV1A/Ern1 flfl-Pax7-Representative image with box.tiff]

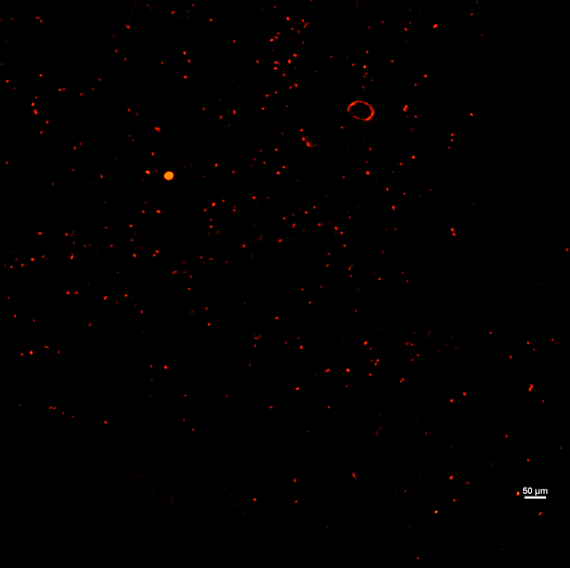

Supplement: Supplementary file 12 — Figure EV1 Source Data [file 44319_2024_197_MOESM12_ESM.zip › Figure EV1/EV1A/Ern1 flfl-Pax7-Representative image.tif]

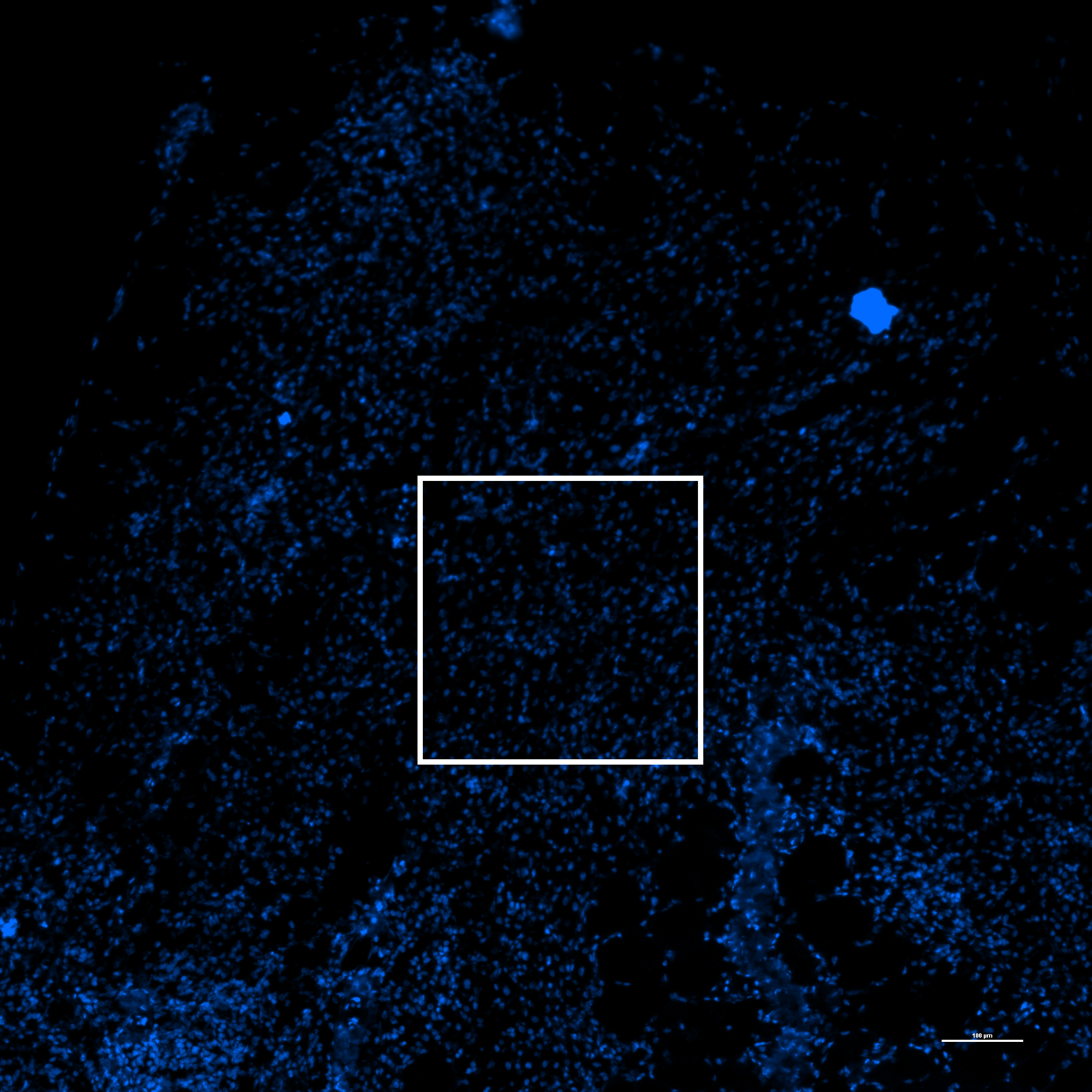

Supplement: Supplementary file 12 — Figure EV1 Source Data [file 44319_2024_197_MOESM12_ESM.zip › Figure EV1/EV1A/Ern1 scko-DAPI-Representative image with box.tiff]

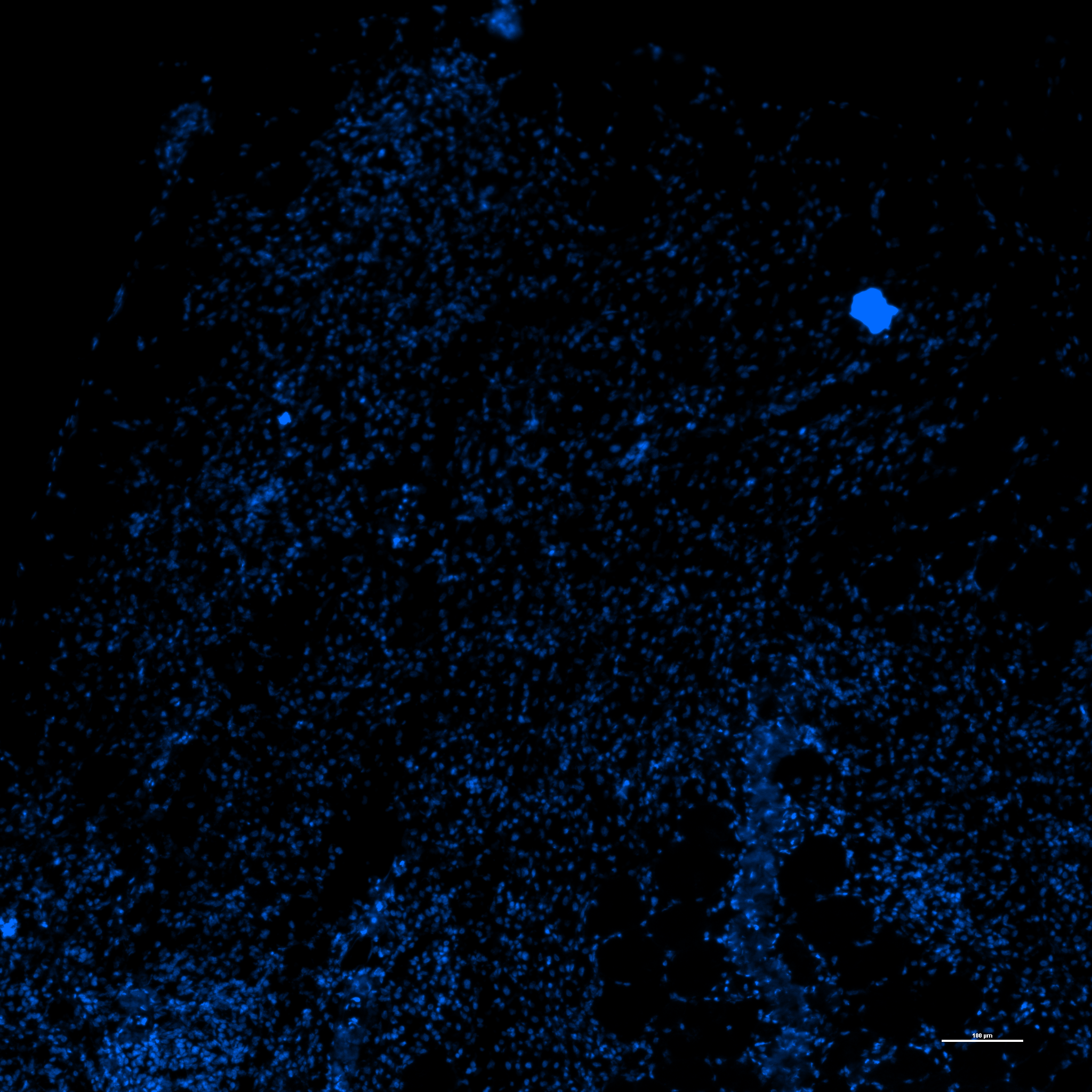

Supplement: Supplementary file 12 — Figure EV1 Source Data [file 44319_2024_197_MOESM12_ESM.zip › Figure EV1/EV1A/Ern1 scko-DAPI-Representative image.tif]

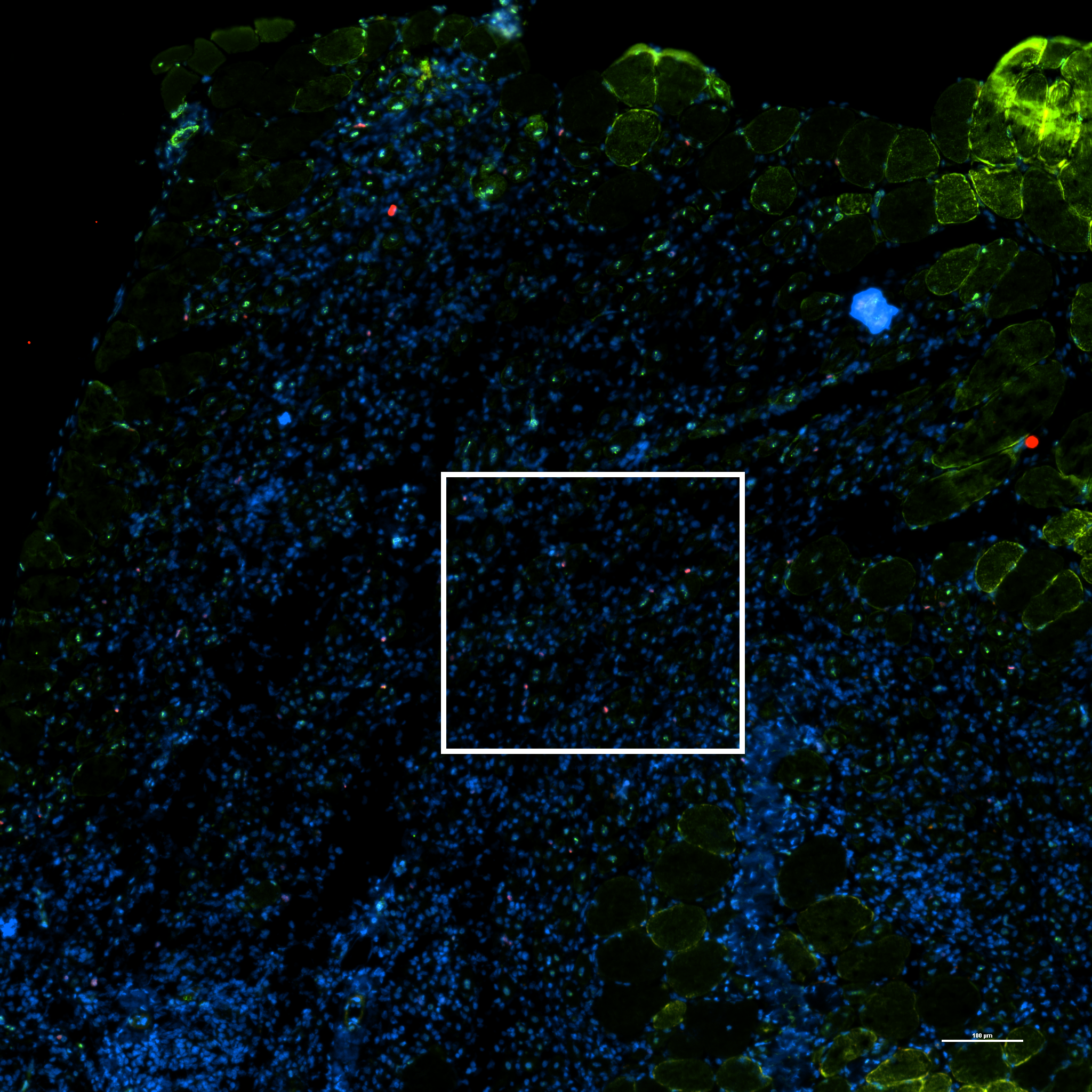

Supplement: Supplementary file 12 — Figure EV1 Source Data [file 44319_2024_197_MOESM12_ESM.zip › Figure EV1/EV1A/Ern1 scko-Merged-Representative image with box.tiff]

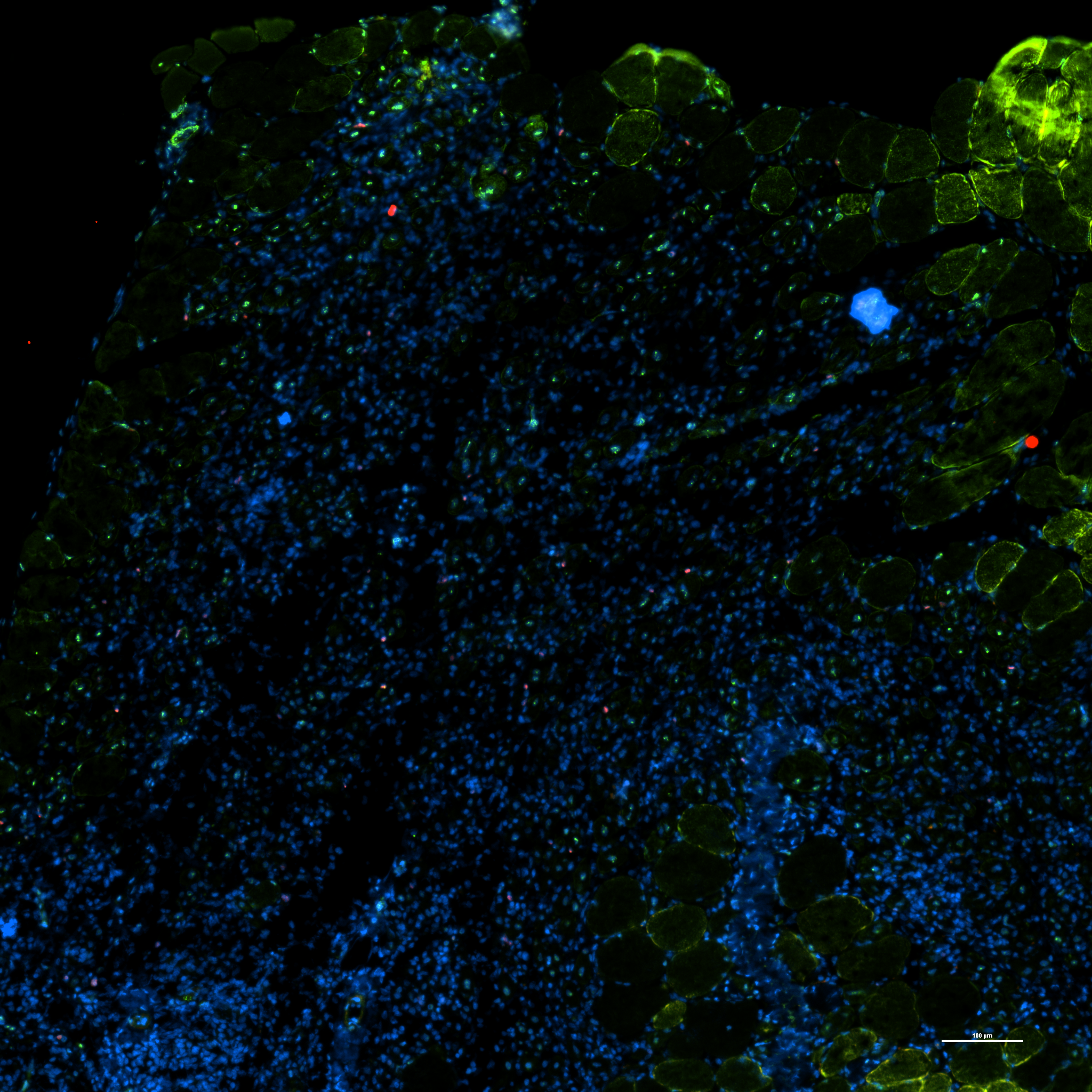

Supplement: Supplementary file 12 — Figure EV1 Source Data [file 44319_2024_197_MOESM12_ESM.zip › Figure EV1/EV1A/Ern1 scko-Merged-Representative image.tif]

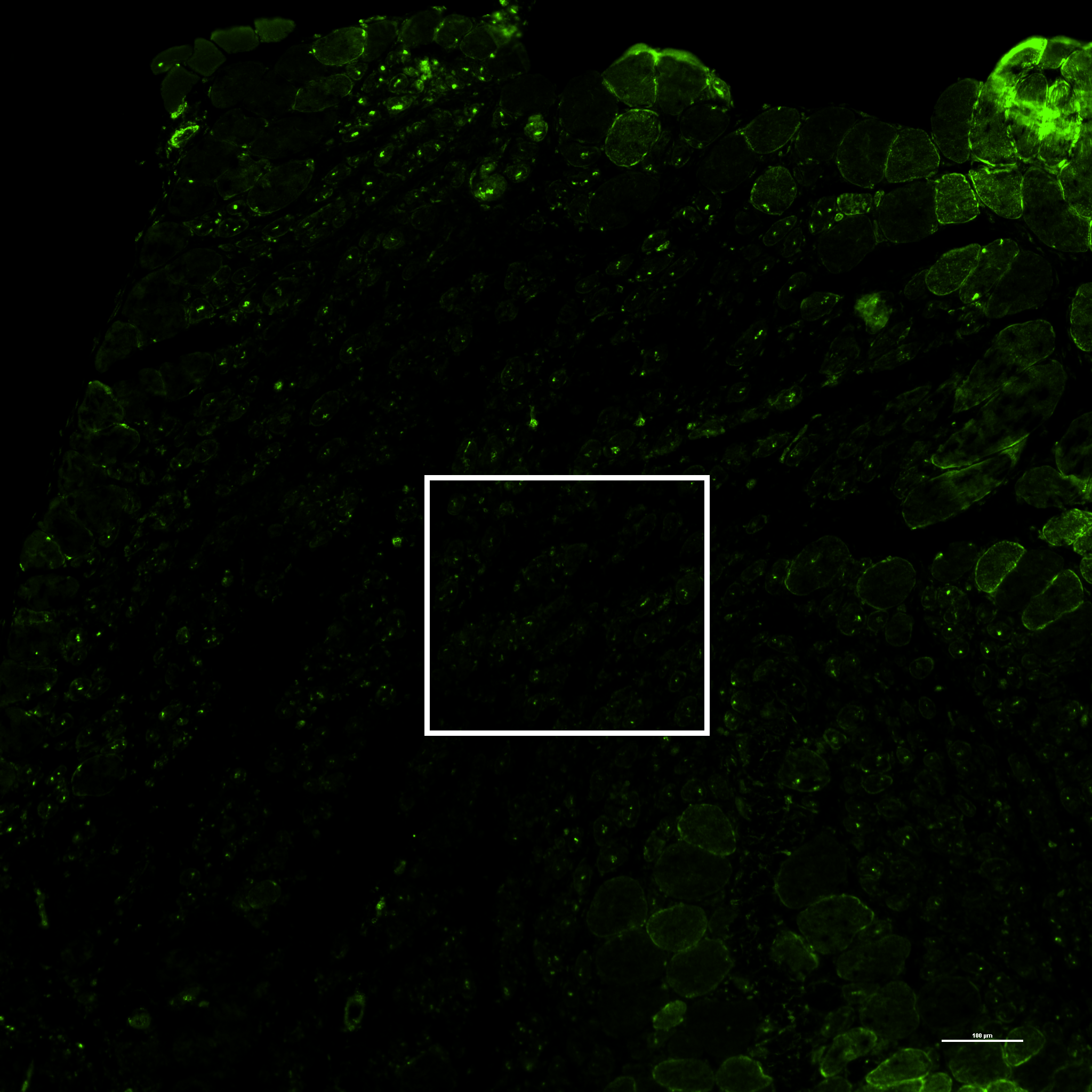

Supplement: Supplementary file 12 — Figure EV1 Source Data [file 44319_2024_197_MOESM12_ESM.zip › Figure EV1/EV1A/Ern1 scko-p-IRE1a-Representative image with box.tiff]

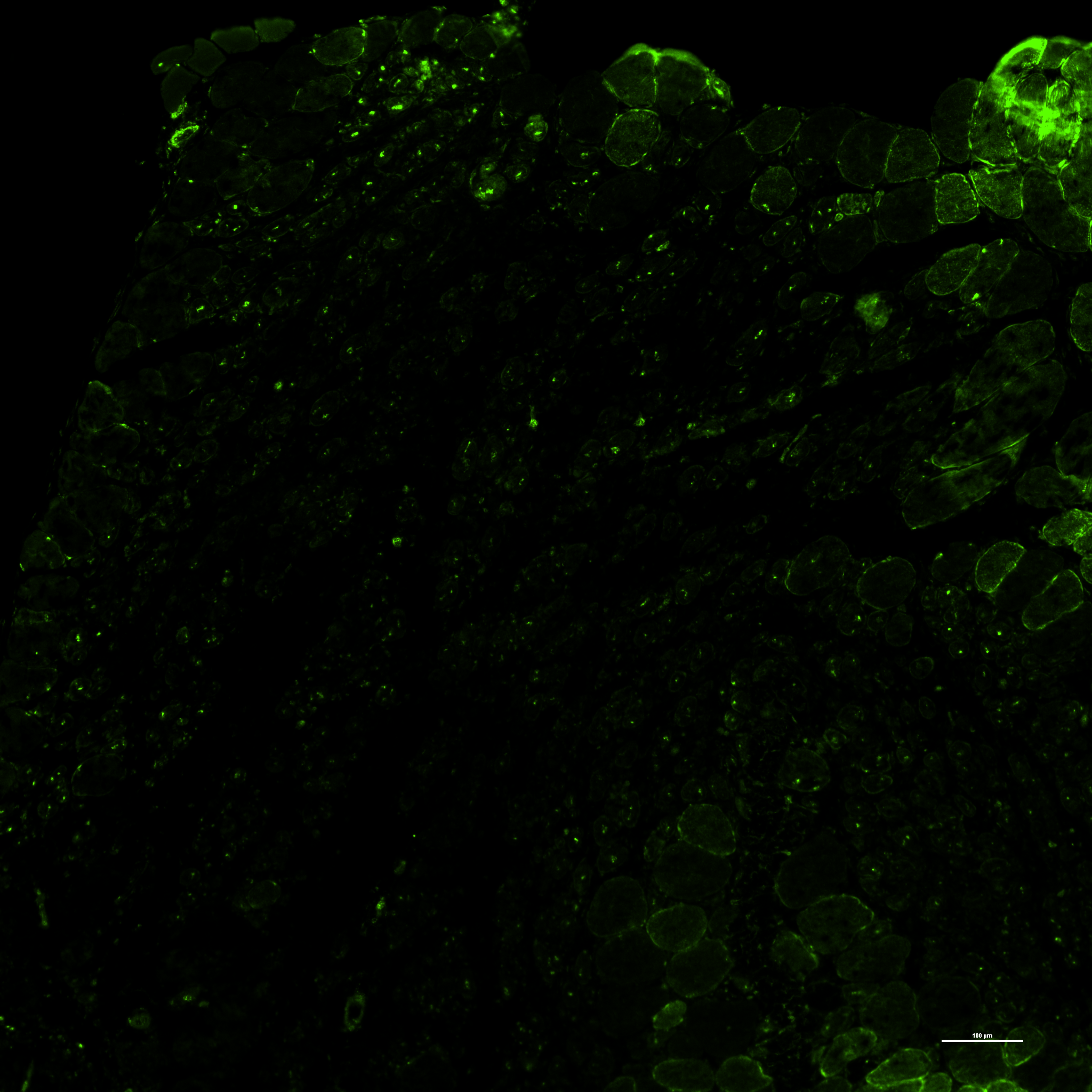

Supplement: Supplementary file 12 — Figure EV1 Source Data [file 44319_2024_197_MOESM12_ESM.zip › Figure EV1/EV1A/Ern1 scko-p-IRE1a-Representative image.tif]

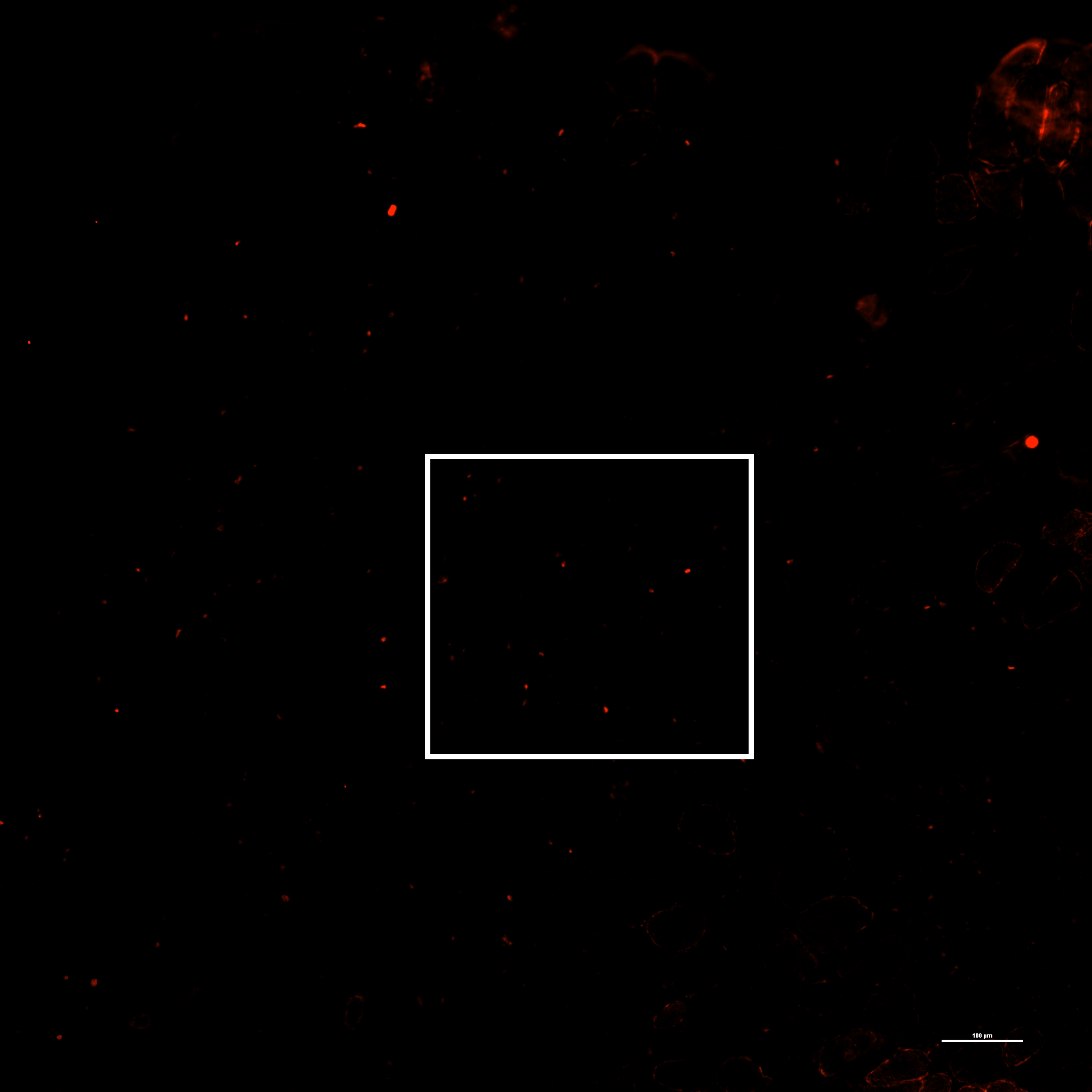

Supplement: Supplementary file 12 — Figure EV1 Source Data [file 44319_2024_197_MOESM12_ESM.zip › Figure EV1/EV1A/Ern1 scko-Pax7-Representative image with box.tiff]

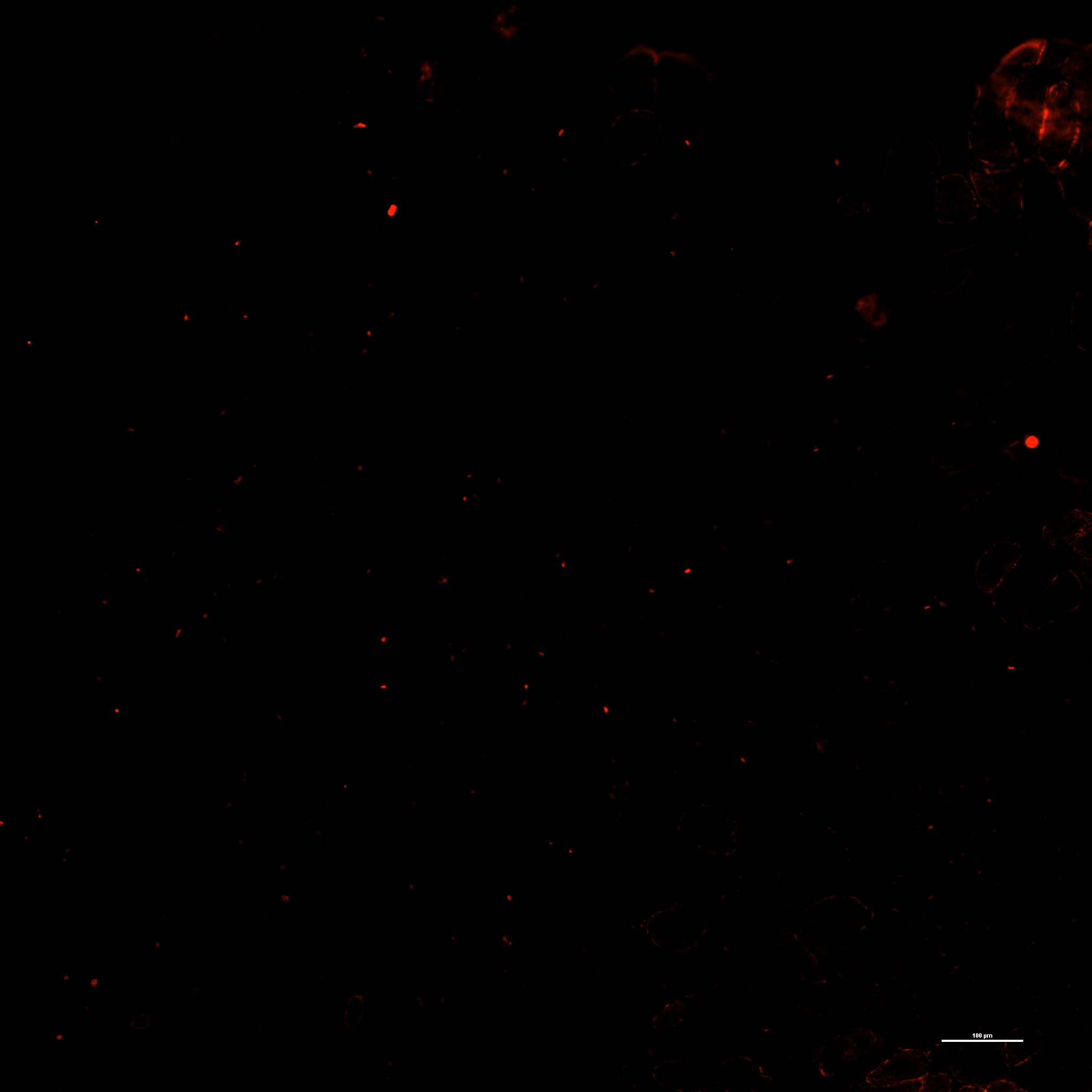

Supplement: Supplementary file 12 — Figure EV1 Source Data [file 44319_2024_197_MOESM12_ESM.zip › Figure EV1/EV1A/Ern1 scko-Pax7-Representative image.tif]

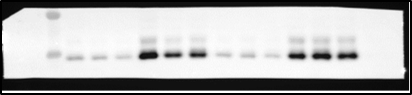

Supplement: Supplementary file 12 — Figure EV1 Source Data [file 44319_2024_197_MOESM12_ESM.zip › Figure EV1/EV1B/CHOP - Western.tif]

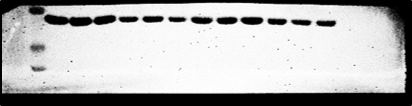

Supplement: Supplementary file 12 — Figure EV1 Source Data [file 44319_2024_197_MOESM12_ESM.zip › Figure EV1/EV1B/GAPDH - Western.tif]
