# Supplementary material for: The IRE1α/XBP1 signaling axis drives myoblast fusion in adult skeletal muscle
Source: EMBO Rep. 2024 Jul 9;25(8):3627–50. doi: 10.1038/s44319-024-00197-4 (PMC11316051; doi:10.1038/s44319-024-00197-4)
Supplement: Supplementary file 10 — Source data Fig. 7 [file 44319_2024_197_MOESM10_ESM.zip › Figure 7/7C/ChIP-PCR agarose gel with annotation.pptx]

## Slide 1
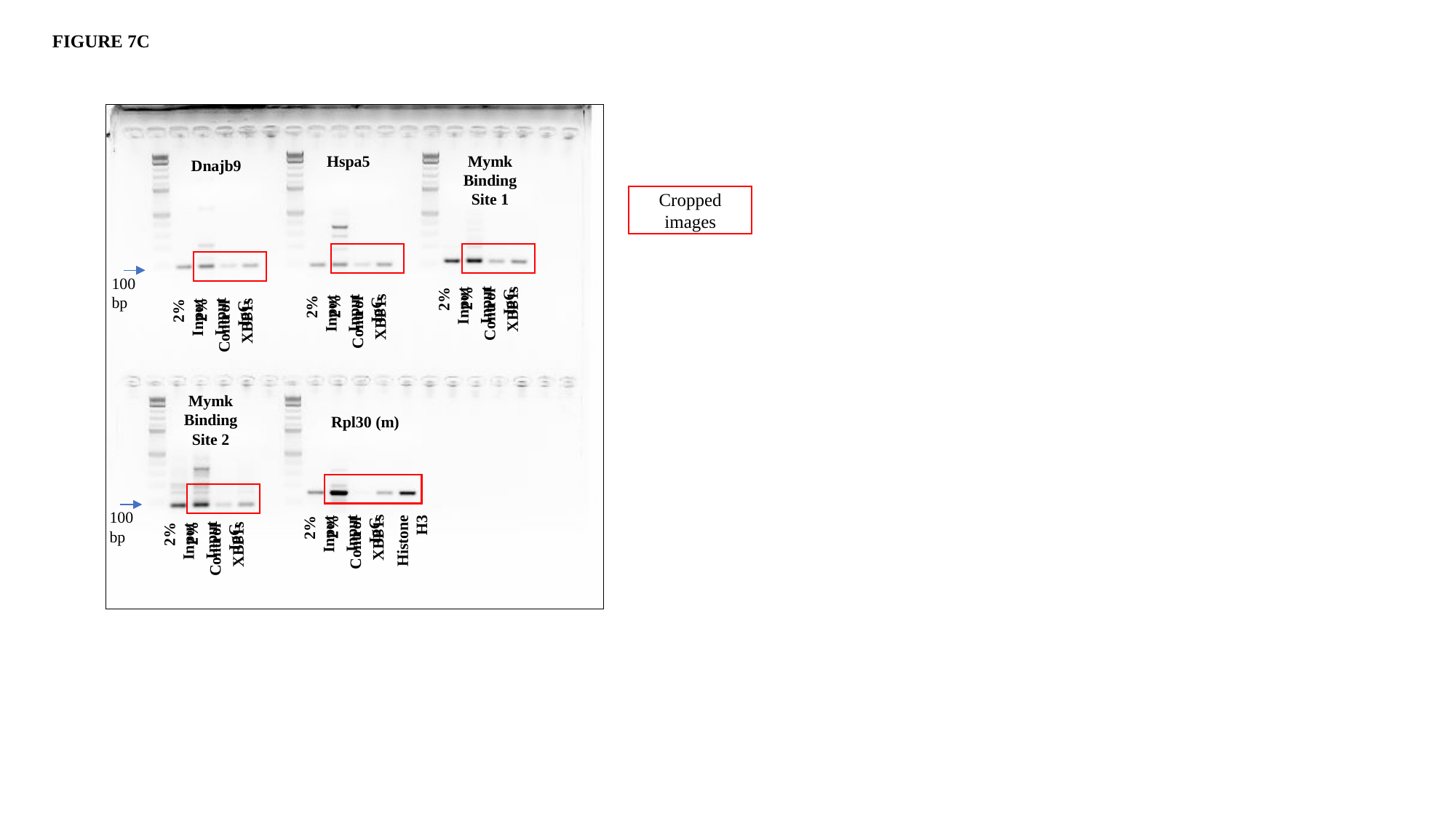

FIGURE 7C
Hspa5
Mymk Binding Site 1
Dnajb9
Cropped images
100 bp
2% Input
XBP1s
2% Input
Control IgG
2% Input
XBP1s
2% Input
Control IgG
2% Input
XBP1s
2% Input
Control IgG
Mymk Binding Site 2
Rpl30 (m)
100 bp
2% Input
XBP1s
Histone H3
2% Input
Control IgG
2% Input
XBP1s
2% Input
Control IgG
