# Supplementary material for: The IRE1α/XBP1 signaling axis drives myoblast fusion in adult skeletal muscle
Source: EMBO Rep. 2024 Jul 9;25(8):3627–50. doi: 10.1038/s44319-024-00197-4 (PMC11316051; doi:10.1038/s44319-024-00197-4)
Supplement: Supplementary file 12 — Figure EV1 Source Data [file 44319_2024_197_MOESM12_ESM.zip › Figure EV1/EV1B/Western blot with annotation.pptx]

## Slide 1
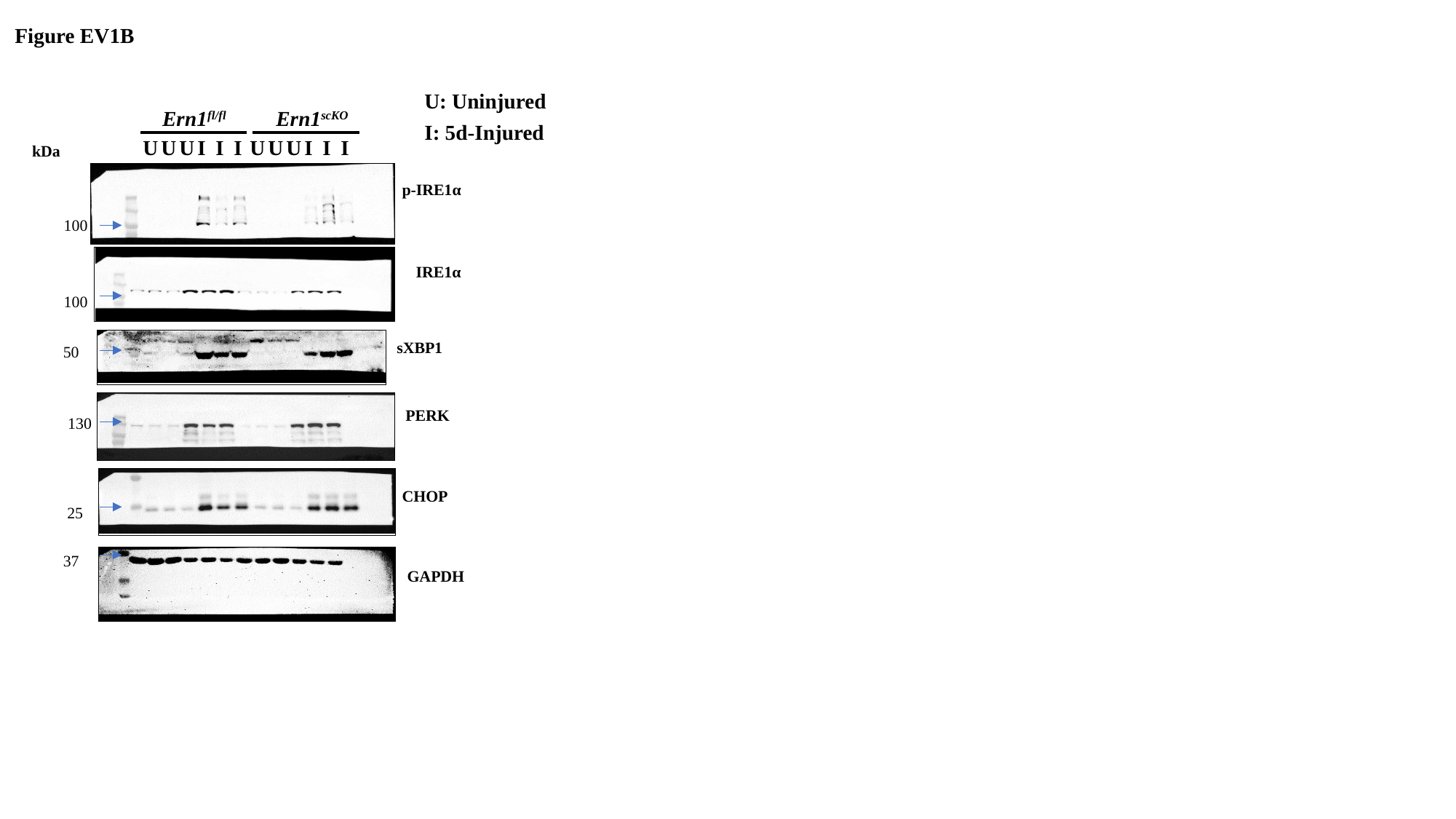

Figure EV1B
U: Uninjured
Ern1fl/fl
Ern1scKO
I: 5d-Injured
U
U
U
I
I
I
U
U
U
I
I
I
kDa
p-IRE1α
100
IRE1α
100
sXBP1
50
PERK
130
CHOP
25
37
GAPDH
