# Supplementary figures and images for: The IRE1α/XBP1 signaling axis drives myoblast fusion in adult skeletal muscle (part 3 of 4)
Source: EMBO Rep. 2024 Jul 9;25(8):3627–50. doi: 10.1038/s44319-024-00197-4 (PMC11316051; doi:10.1038/s44319-024-00197-4)

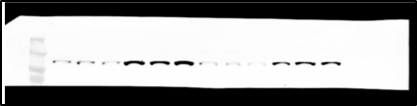

Supplement: Supplementary file 12 — Figure EV1 Source Data [file 44319_2024_197_MOESM12_ESM.zip › Figure EV1/EV1B/IRE1a - Western.tif]

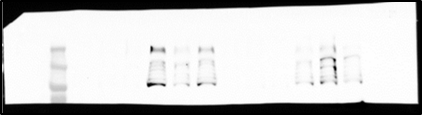

Supplement: Supplementary file 12 — Figure EV1 Source Data [file 44319_2024_197_MOESM12_ESM.zip › Figure EV1/EV1B/p-IRE1a - Western.tif]

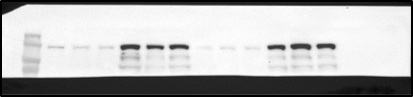

Supplement: Supplementary file 12 — Figure EV1 Source Data [file 44319_2024_197_MOESM12_ESM.zip › Figure EV1/EV1B/PERK - Western.tif]

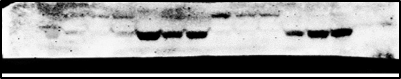

Supplement: Supplementary file 12 — Figure EV1 Source Data [file 44319_2024_197_MOESM12_ESM.zip › Figure EV1/EV1B/sXBP1 - Western.tif]

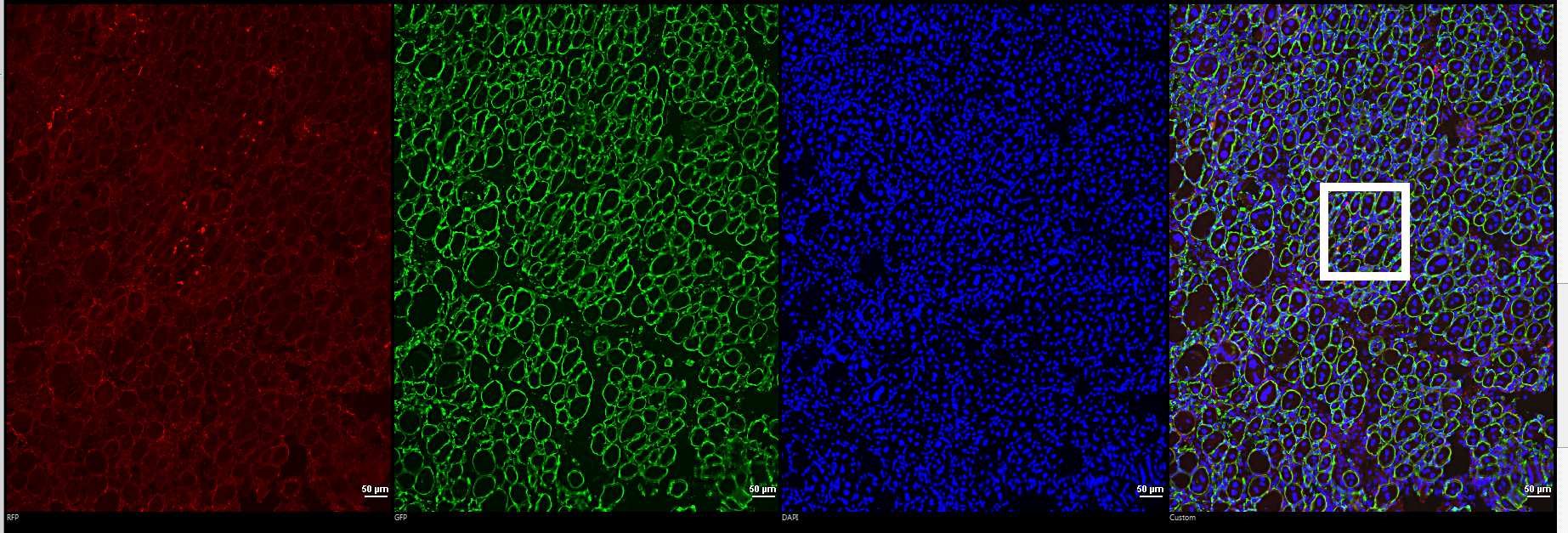

Supplement: Supplementary file 13 — Figure EV2 Source Data [file 44319_2024_197_MOESM13_ESM.zip › Figure EV2/EV2A-B/EV2A-Pax7 staining/Ern1 fl fl Representative image with box.tiff]

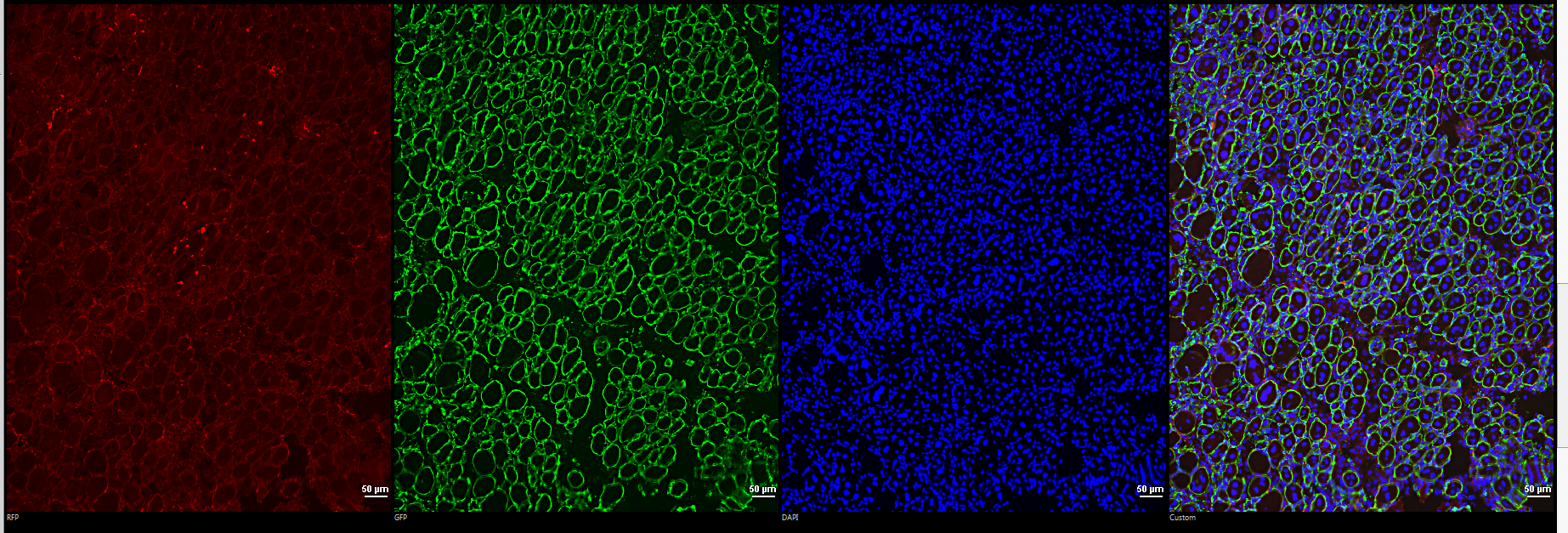

Supplement: Supplementary file 13 — Figure EV2 Source Data [file 44319_2024_197_MOESM13_ESM.zip › Figure EV2/EV2A-B/EV2A-Pax7 staining/Ern1 fl fl Representative image.tif]

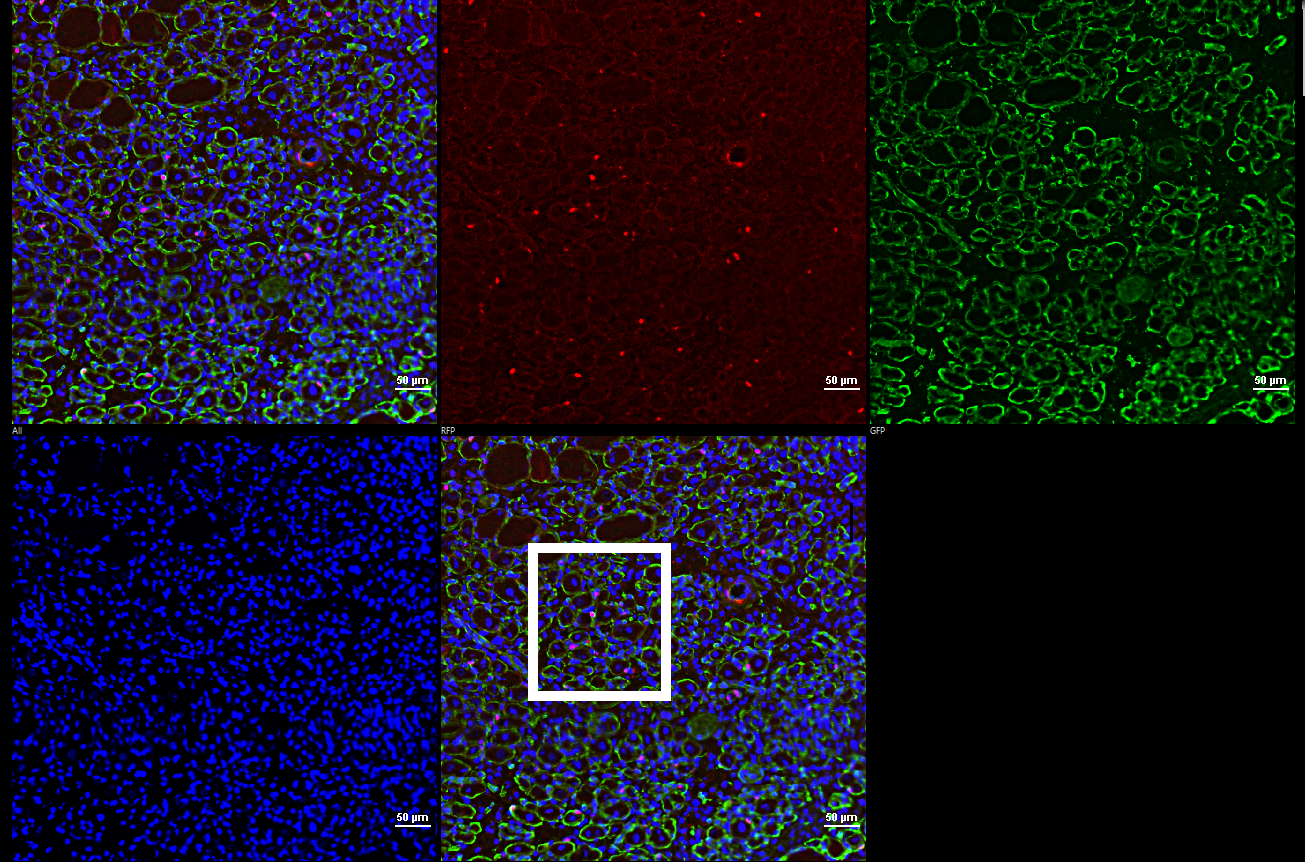

Supplement: Supplementary file 13 — Figure EV2 Source Data [file 44319_2024_197_MOESM13_ESM.zip › Figure EV2/EV2A-B/EV2A-Pax7 staining/Ern1 scKO Representative image with box.tiff]

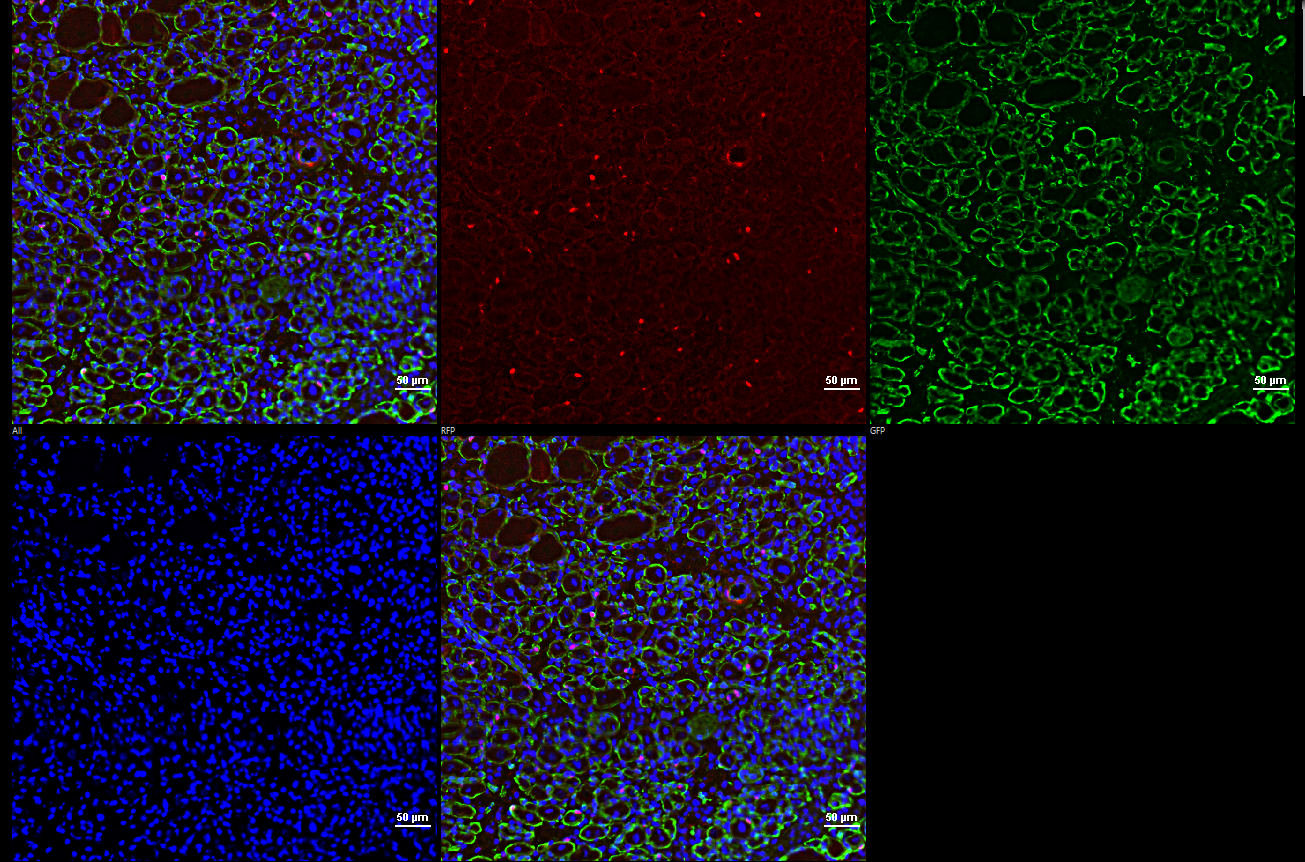

Supplement: Supplementary file 13 — Figure EV2 Source Data [file 44319_2024_197_MOESM13_ESM.zip › Figure EV2/EV2A-B/EV2A-Pax7 staining/Ern1 scKO Representative image.tif]

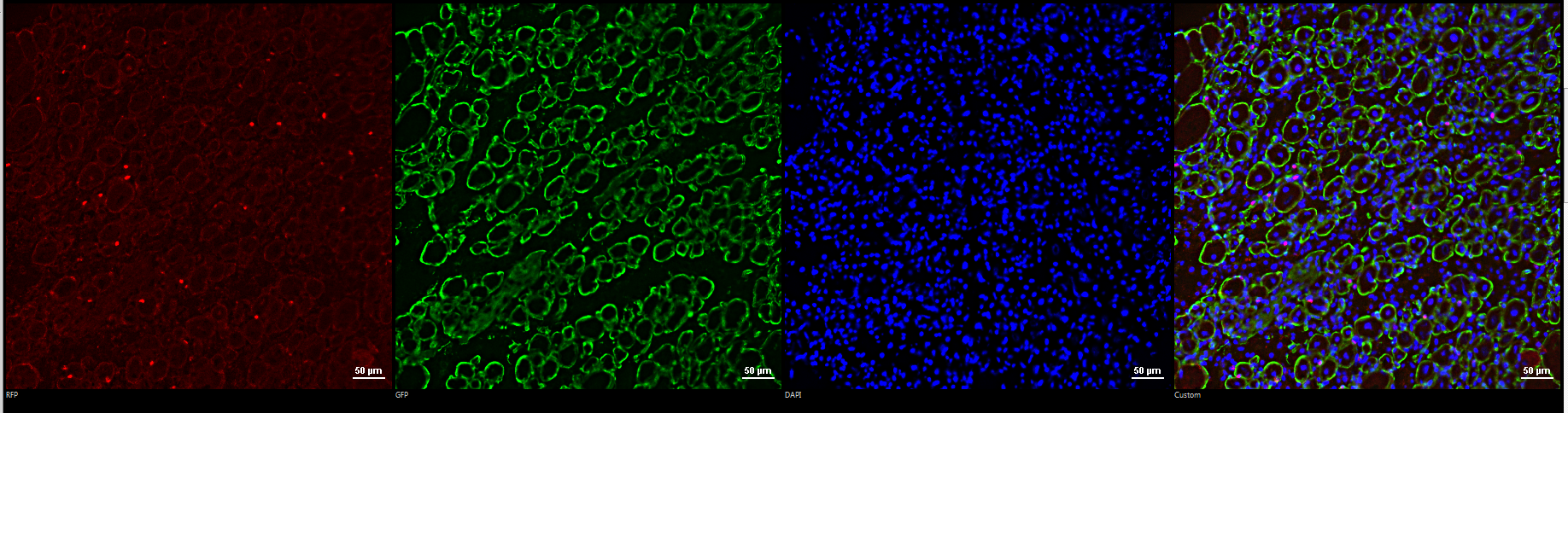

Supplement: Supplementary file 13 — Figure EV2 Source Data [file 44319_2024_197_MOESM13_ESM.zip › Figure EV2/EV2A-B/EV2A-Pax7 staining/Pax7 staining-Ern1 scKO.tif]

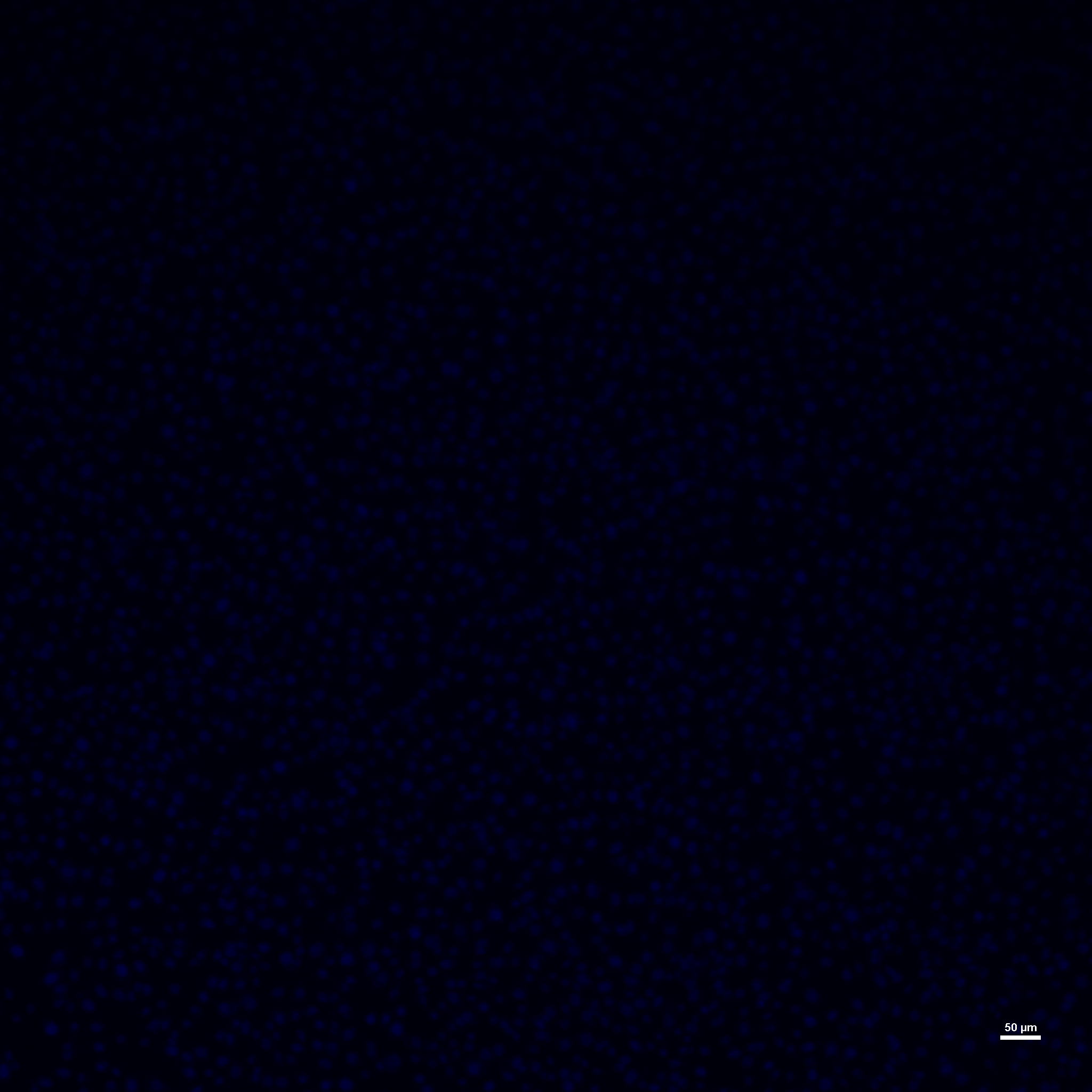

Supplement: Supplementary file 13 — Figure EV2 Source Data [file 44319_2024_197_MOESM13_ESM.zip › Figure EV2/EV2C-F/Pax7-MyoD-DAPI staining/Control siRNA-DAPI-Representative image.tif]

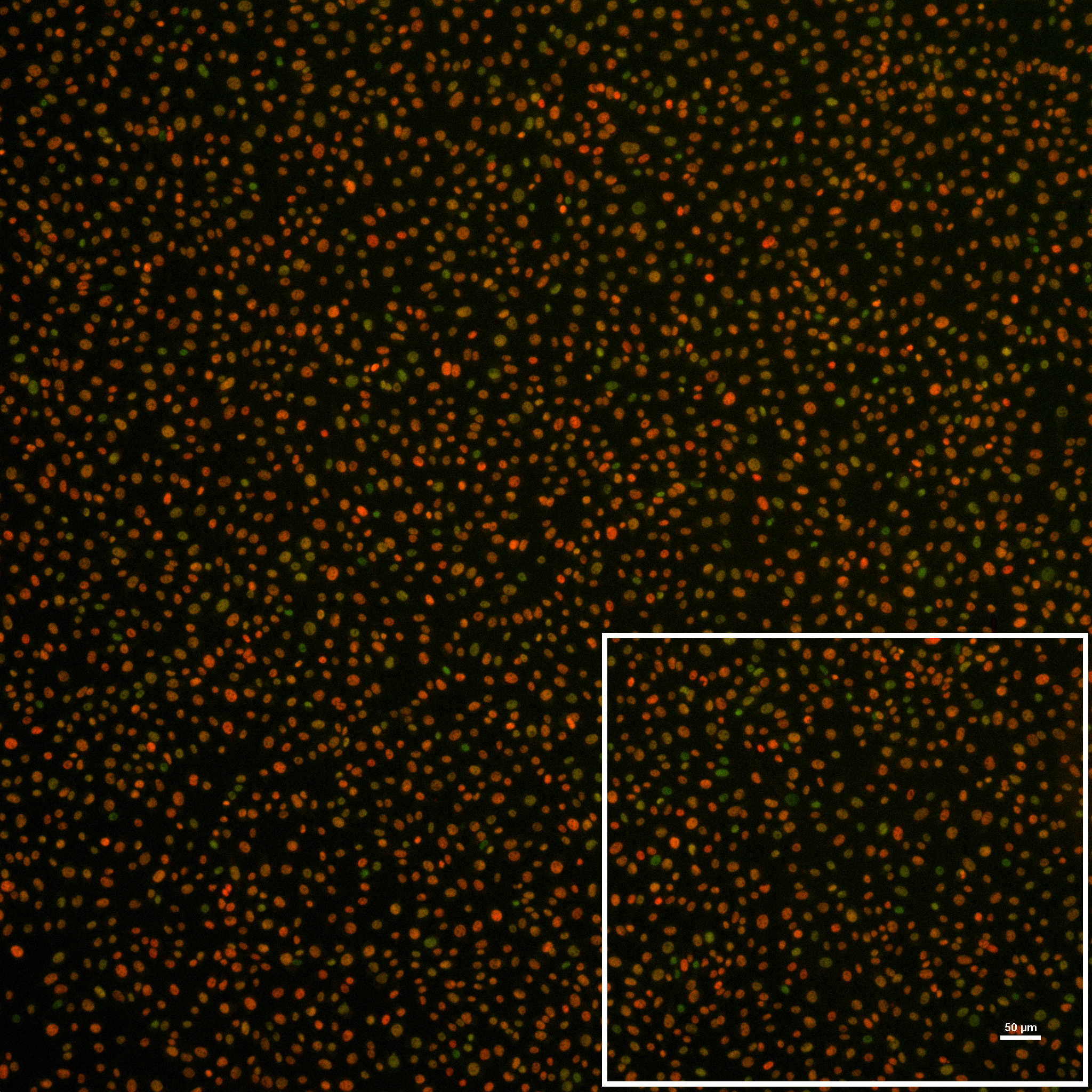

Supplement: Supplementary file 13 — Figure EV2 Source Data [file 44319_2024_197_MOESM13_ESM.zip › Figure EV2/EV2C-F/Pax7-MyoD-DAPI staining/Control siRNA-Merged-Representative image with box.tiff]

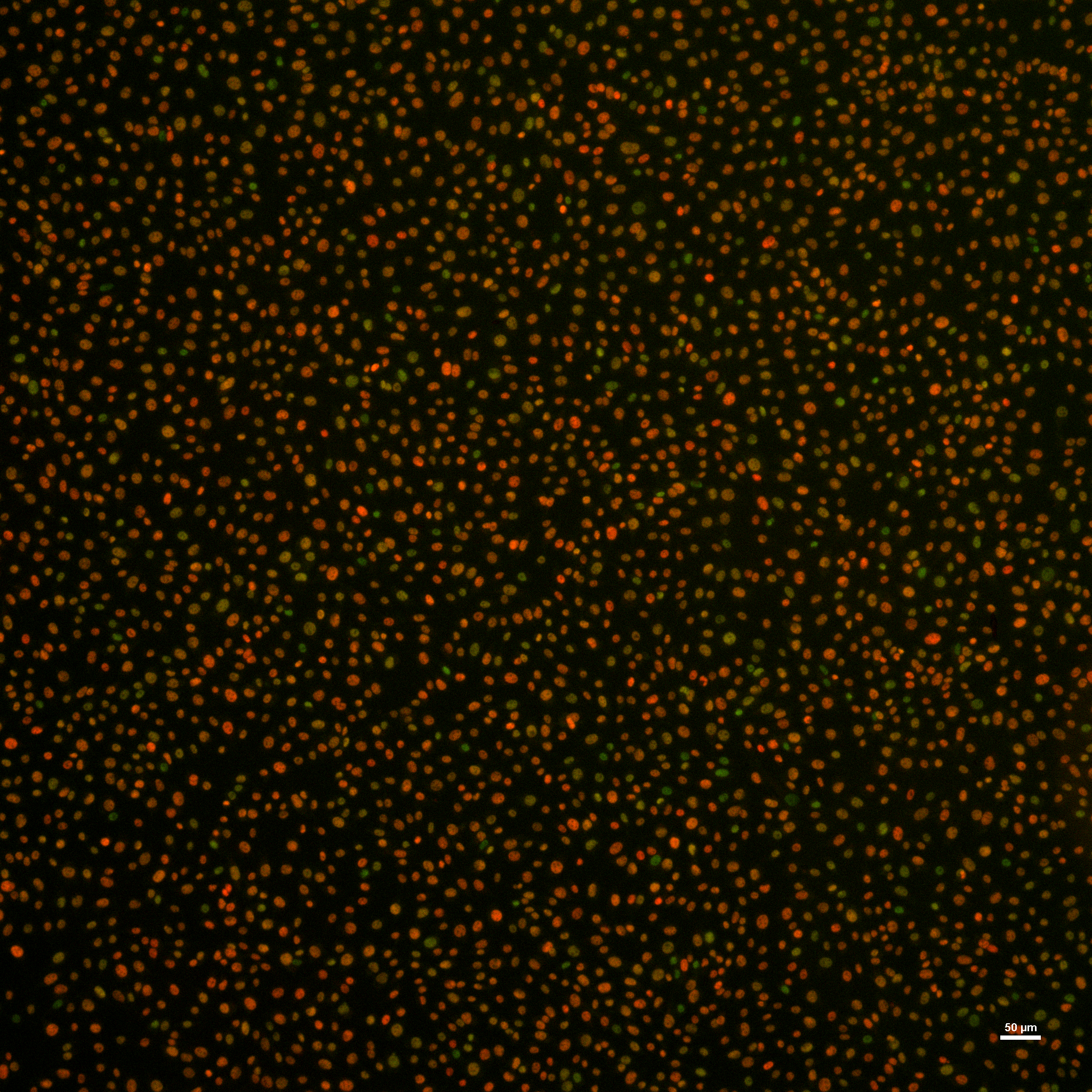

Supplement: Supplementary file 13 — Figure EV2 Source Data [file 44319_2024_197_MOESM13_ESM.zip › Figure EV2/EV2C-F/Pax7-MyoD-DAPI staining/Control siRNA-Merged-Representative image.tif]

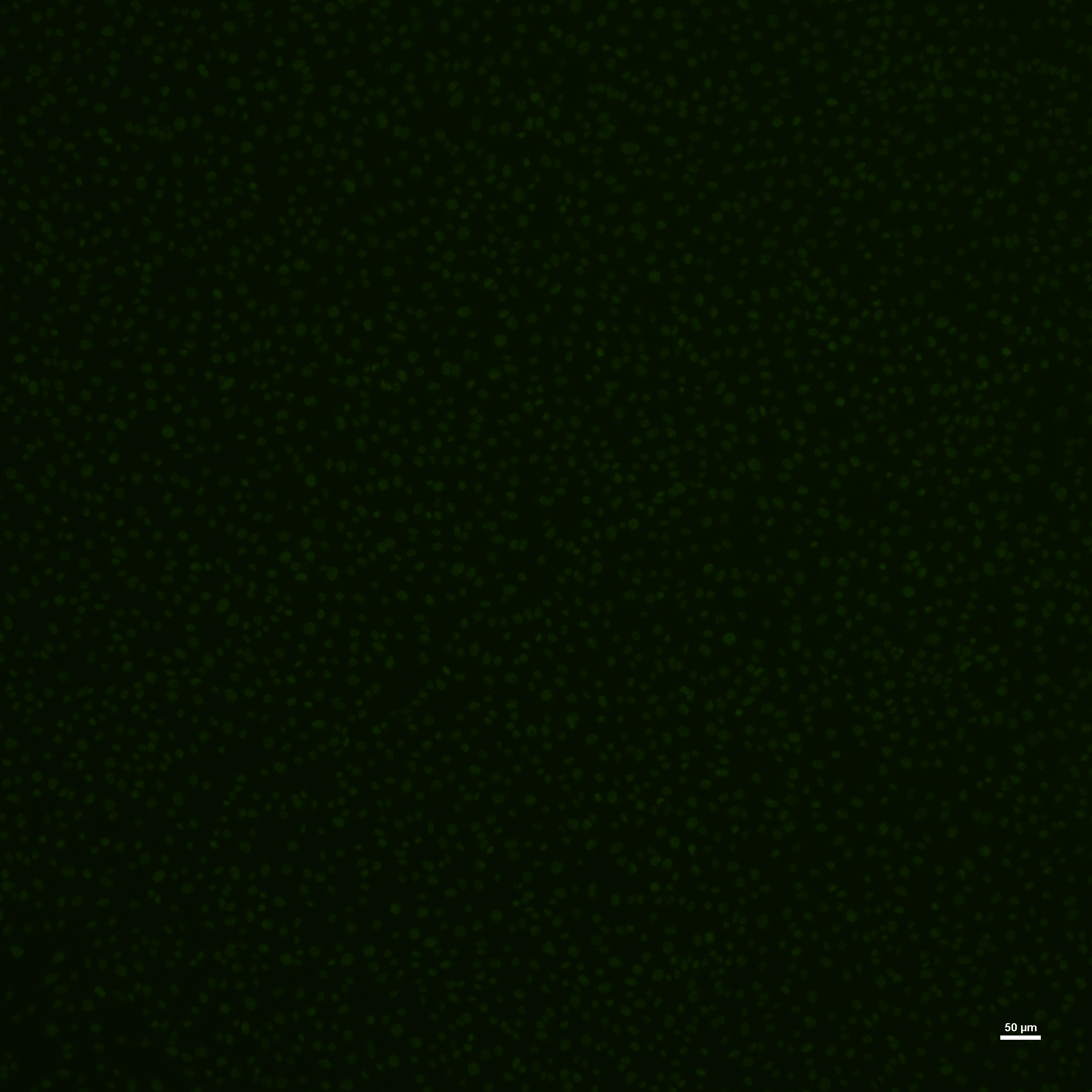

Supplement: Supplementary file 13 — Figure EV2 Source Data [file 44319_2024_197_MOESM13_ESM.zip › Figure EV2/EV2C-F/Pax7-MyoD-DAPI staining/Control siRNA-MyoD-Representative image.tif]

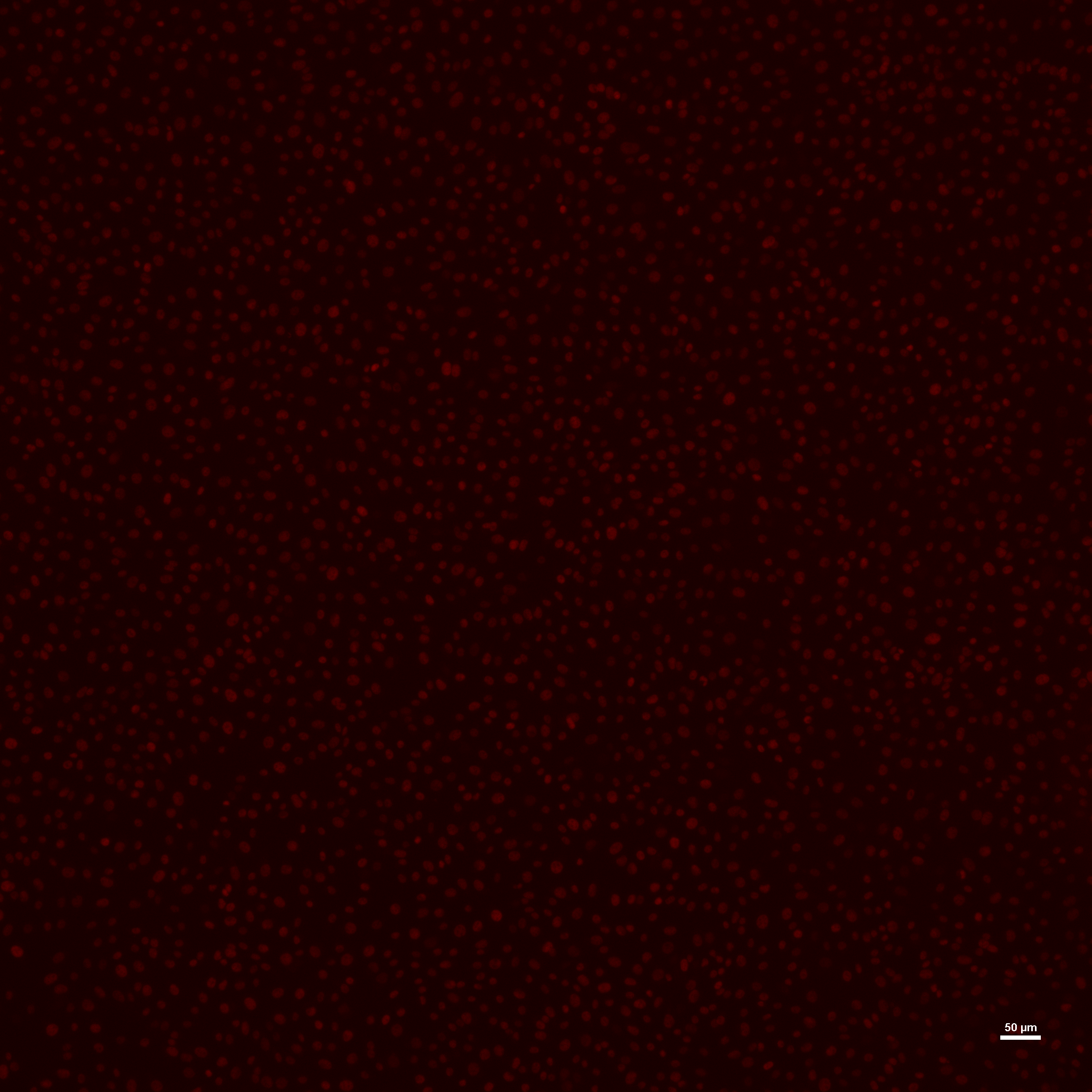

Supplement: Supplementary file 13 — Figure EV2 Source Data [file 44319_2024_197_MOESM13_ESM.zip › Figure EV2/EV2C-F/Pax7-MyoD-DAPI staining/Control siRNA-Pax7-Representative image.tif]

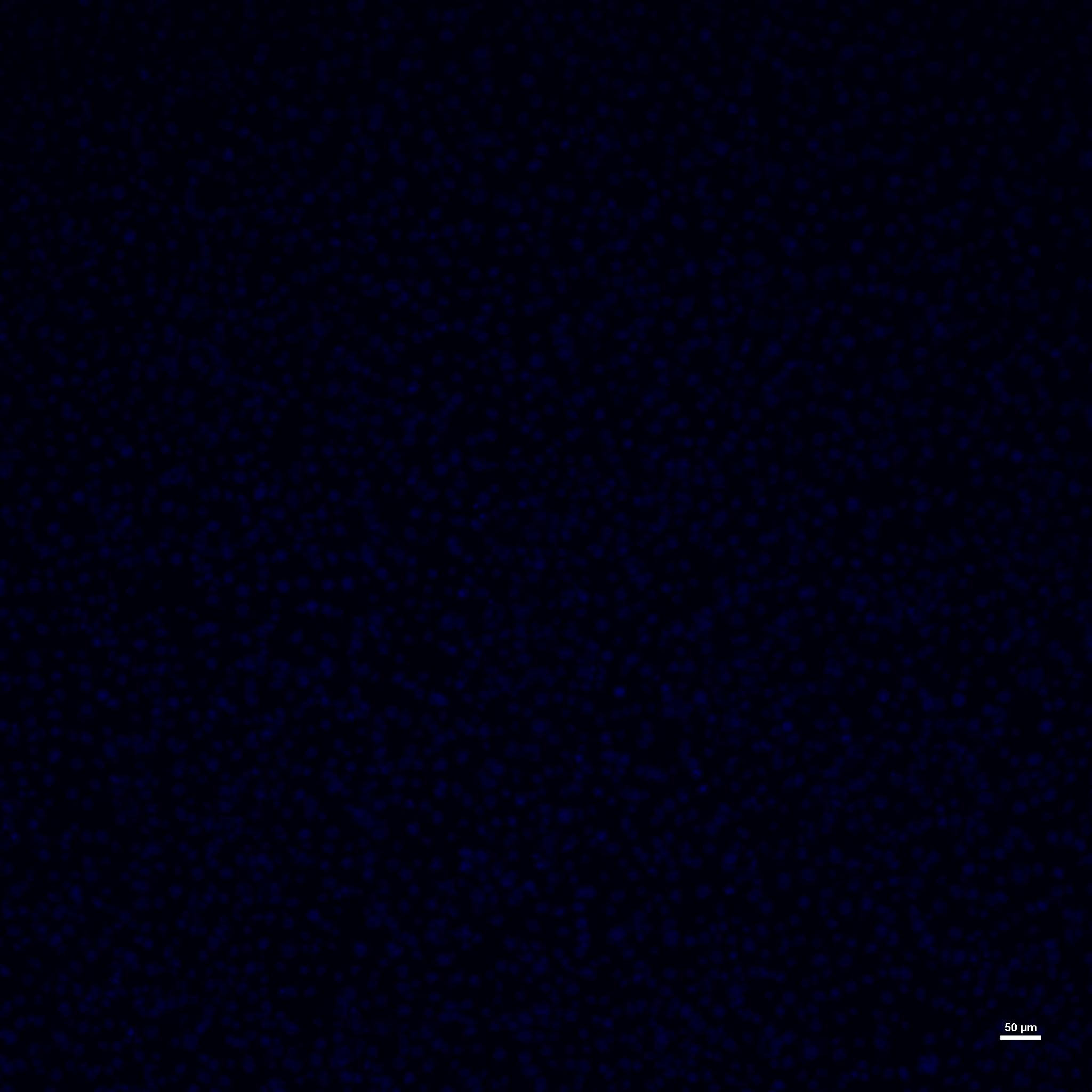

Supplement: Supplementary file 13 — Figure EV2 Source Data [file 44319_2024_197_MOESM13_ESM.zip › Figure EV2/EV2C-F/Pax7-MyoD-DAPI staining/IRE1a siRNA-DAPI-Representative image.tif]

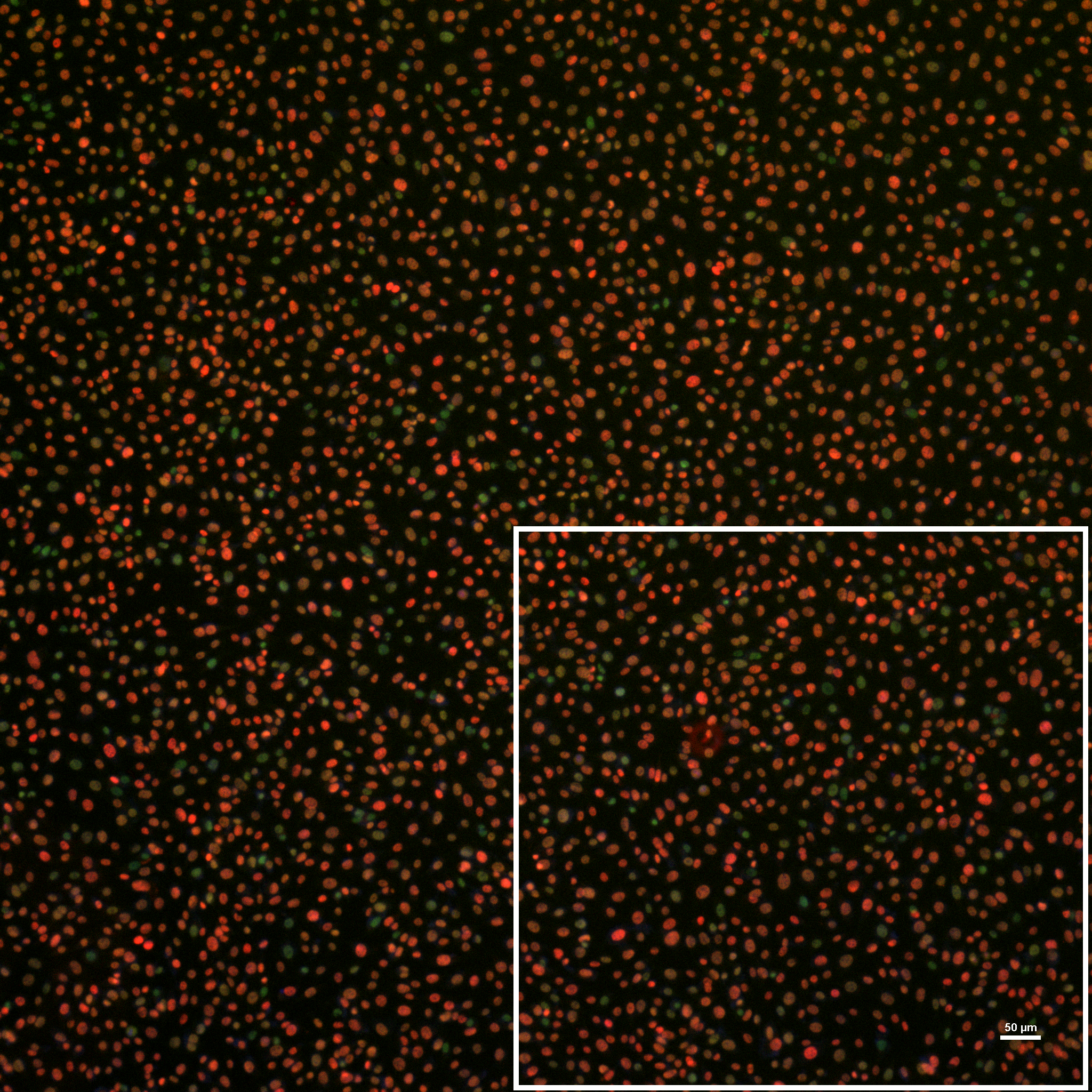

Supplement: Supplementary file 13 — Figure EV2 Source Data [file 44319_2024_197_MOESM13_ESM.zip › Figure EV2/EV2C-F/Pax7-MyoD-DAPI staining/IRE1a siRNA-Merged-Representative image with box.tiff]

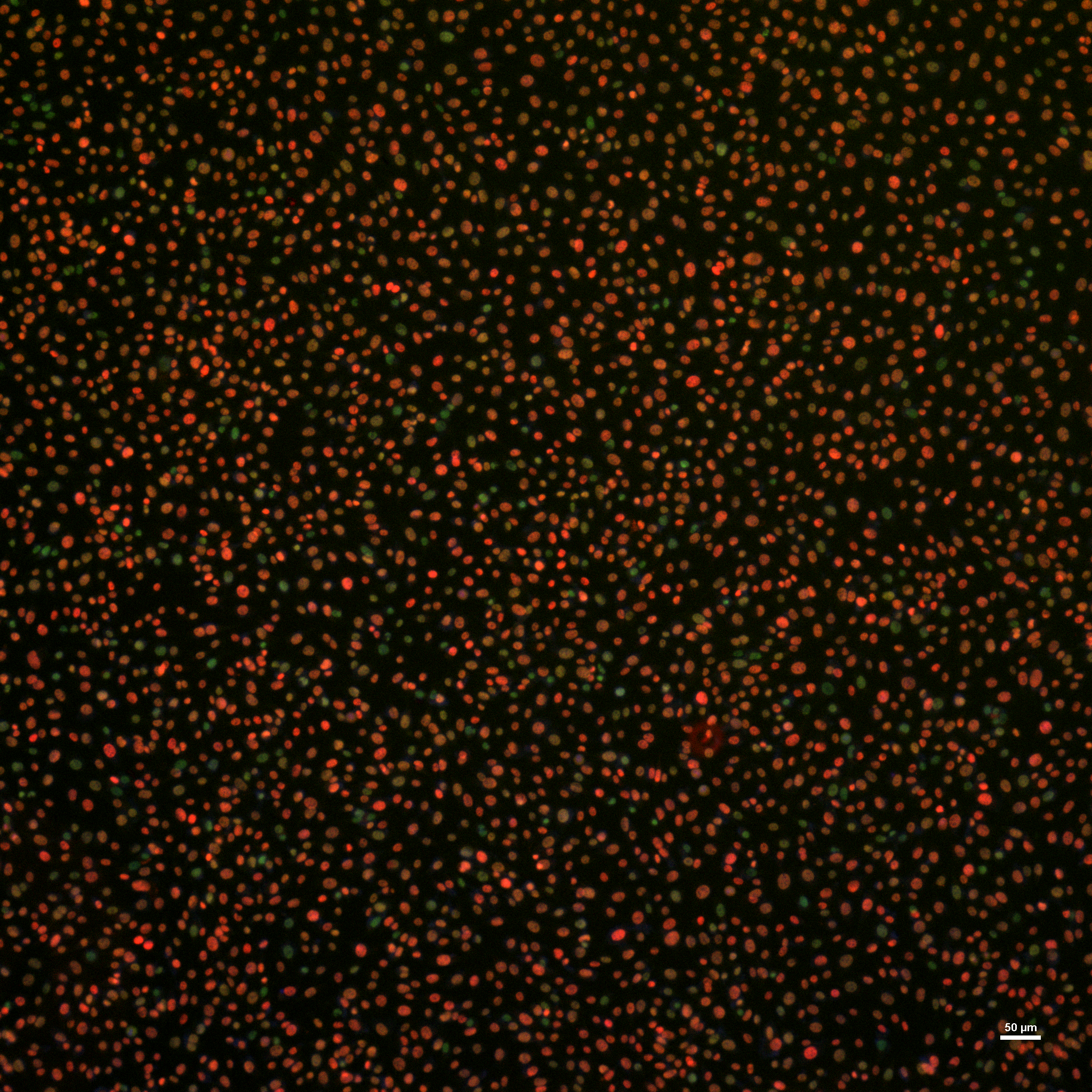

Supplement: Supplementary file 13 — Figure EV2 Source Data [file 44319_2024_197_MOESM13_ESM.zip › Figure EV2/EV2C-F/Pax7-MyoD-DAPI staining/IRE1a siRNA-Merged-Representative image.tif]

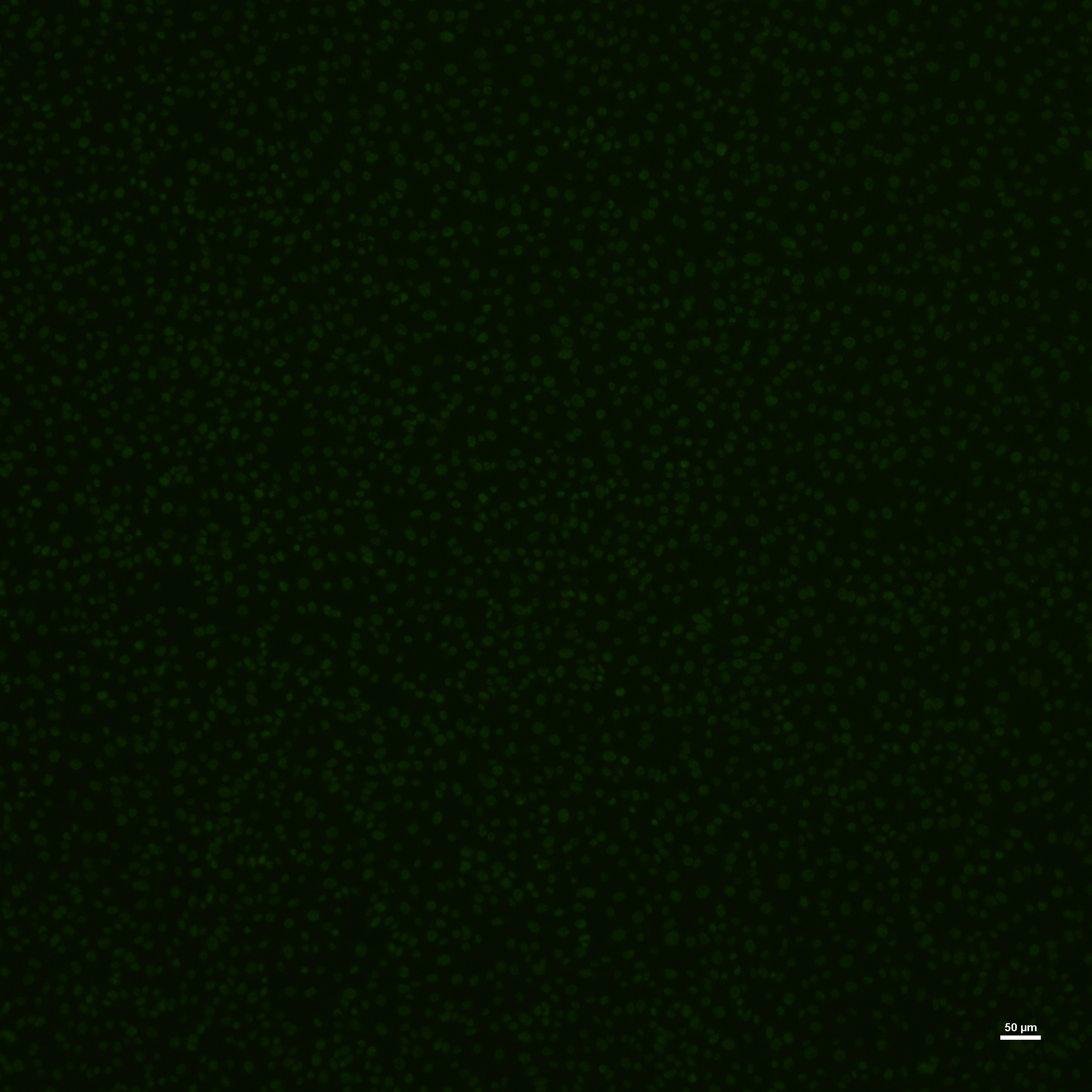

Supplement: Supplementary file 13 — Figure EV2 Source Data [file 44319_2024_197_MOESM13_ESM.zip › Figure EV2/EV2C-F/Pax7-MyoD-DAPI staining/IRE1a siRNA-MyoD-Representative image.tif]

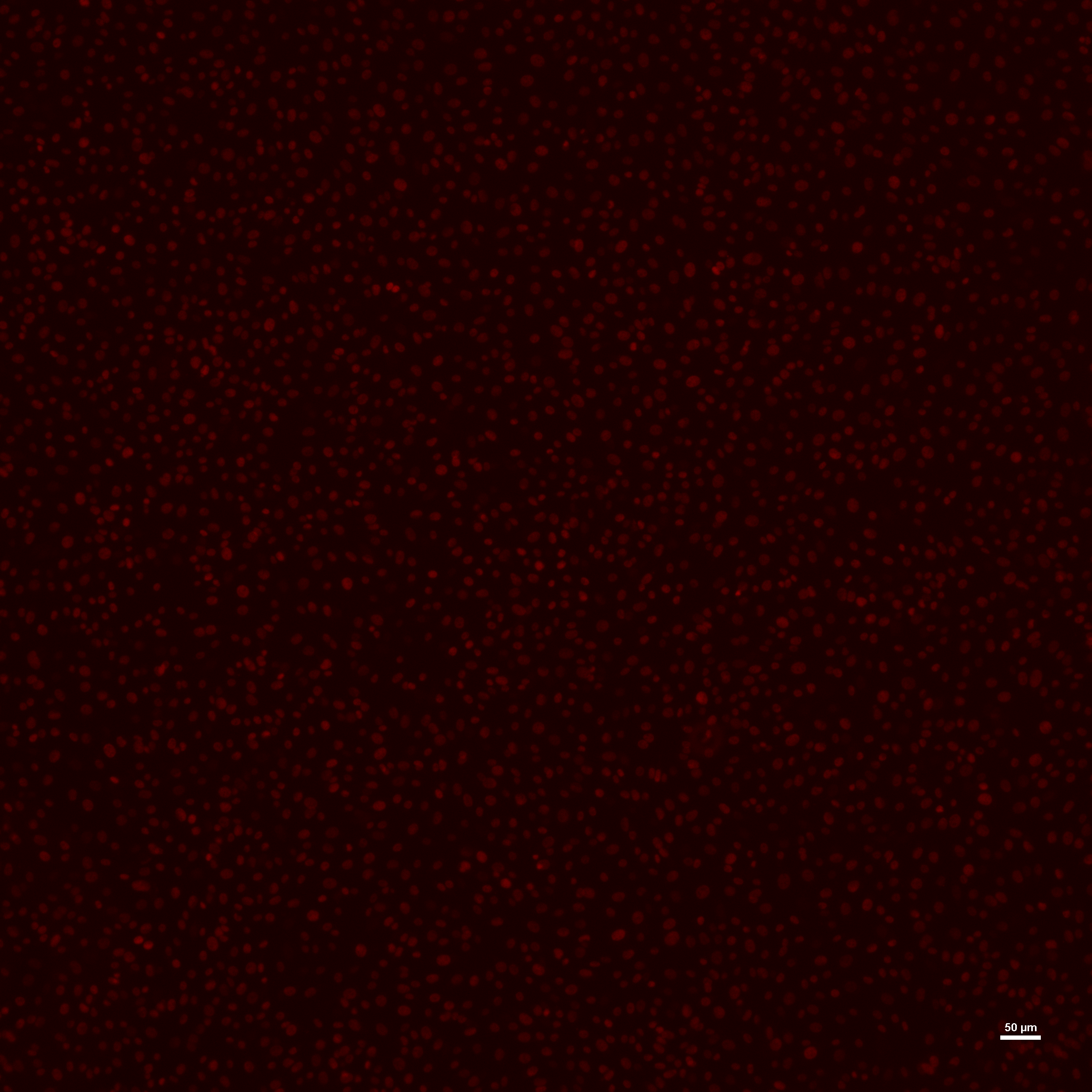

Supplement: Supplementary file 13 — Figure EV2 Source Data [file 44319_2024_197_MOESM13_ESM.zip › Figure EV2/EV2C-F/Pax7-MyoD-DAPI staining/IRE1a siRNA-Pax7-Representative image.tif]

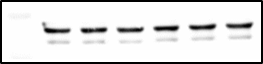

Supplement: Supplementary file 13 — Figure EV2 Source Data [file 44319_2024_197_MOESM13_ESM.zip › Figure EV2/EV2G/GAPDH - Western.tif]

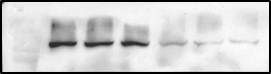

Supplement: Supplementary file 13 — Figure EV2 Source Data [file 44319_2024_197_MOESM13_ESM.zip › Figure EV2/EV2G/IRE1a - Western.tif]

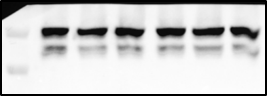

Supplement: Supplementary file 13 — Figure EV2 Source Data [file 44319_2024_197_MOESM13_ESM.zip › Figure EV2/EV2G/JNK - Western.tif]

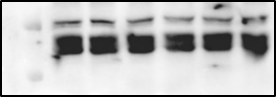

Supplement: Supplementary file 13 — Figure EV2 Source Data [file 44319_2024_197_MOESM13_ESM.zip › Figure EV2/EV2G/MyoD - Western.tif]

## Slide 1
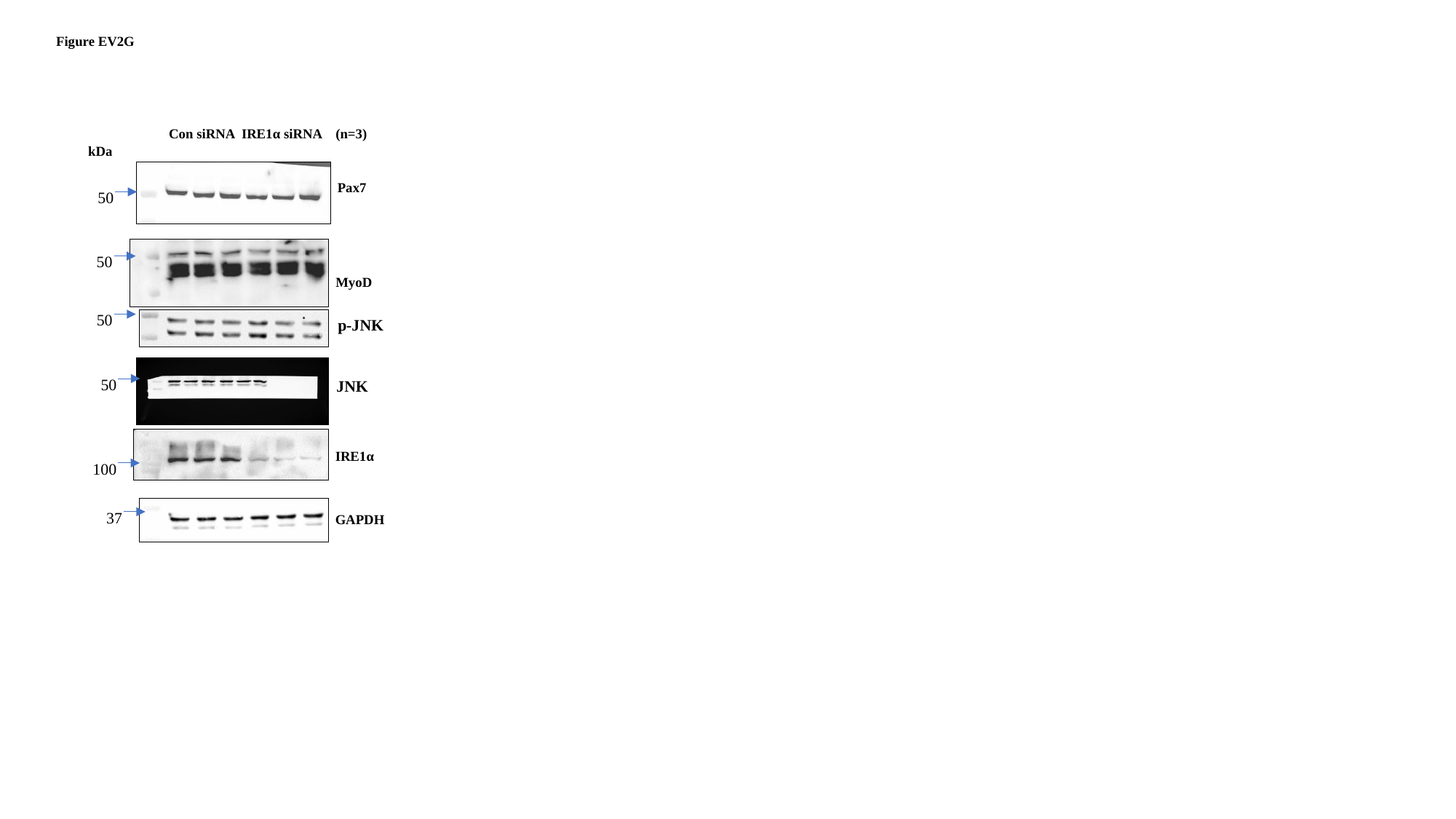

Figure EV2G
Con siRNA
IRE1α siRNA (n=3)
kDa
Pax7
50
50
MyoD
50
p-JNK
50
JNK
IRE1α
100
37
GAPDH

Supplement: Supplementary file 13 — Figure EV2 Source Data [file 44319_2024_197_MOESM13_ESM.zip › Figure EV2/EV2G/New Microsoft PowerPoint Presentation.pptx]

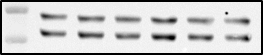

Supplement: Supplementary file 13 — Figure EV2 Source Data [file 44319_2024_197_MOESM13_ESM.zip › Figure EV2/EV2G/p-JNK - Western.tif]

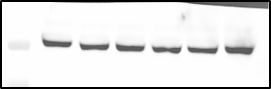

Supplement: Supplementary file 13 — Figure EV2 Source Data [file 44319_2024_197_MOESM13_ESM.zip › Figure EV2/EV2G/Pax7 - Western.tif]

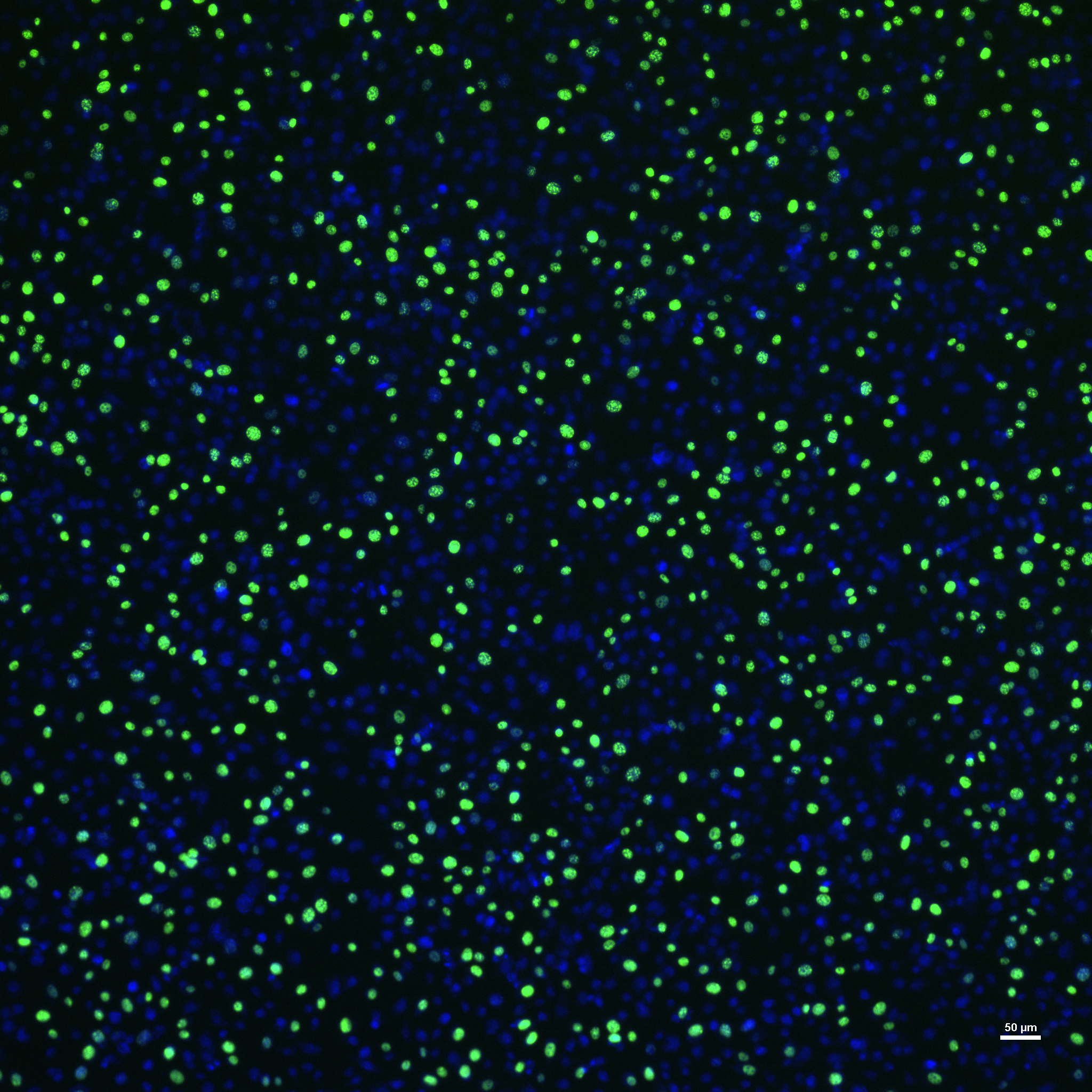

Supplement: Supplementary file 13 — Figure EV2 Source Data [file 44319_2024_197_MOESM13_ESM.zip › Figure EV2/EV2H-I/EdU Staining images/Control siRNA Representative image.tif]

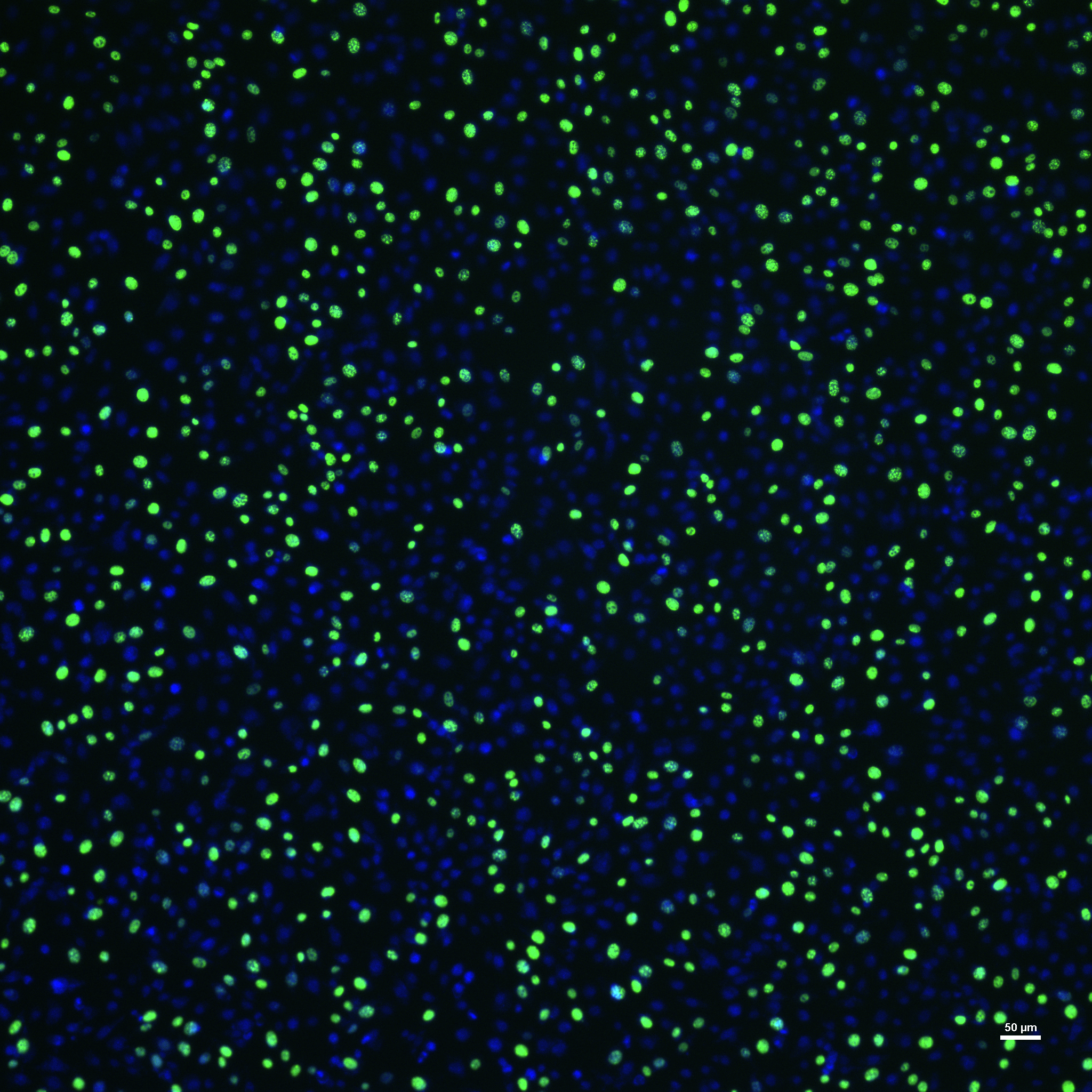

Supplement: Supplementary file 13 — Figure EV2 Source Data [file 44319_2024_197_MOESM13_ESM.zip › Figure EV2/EV2H-I/EdU Staining images/Control siRNA-2.tif]

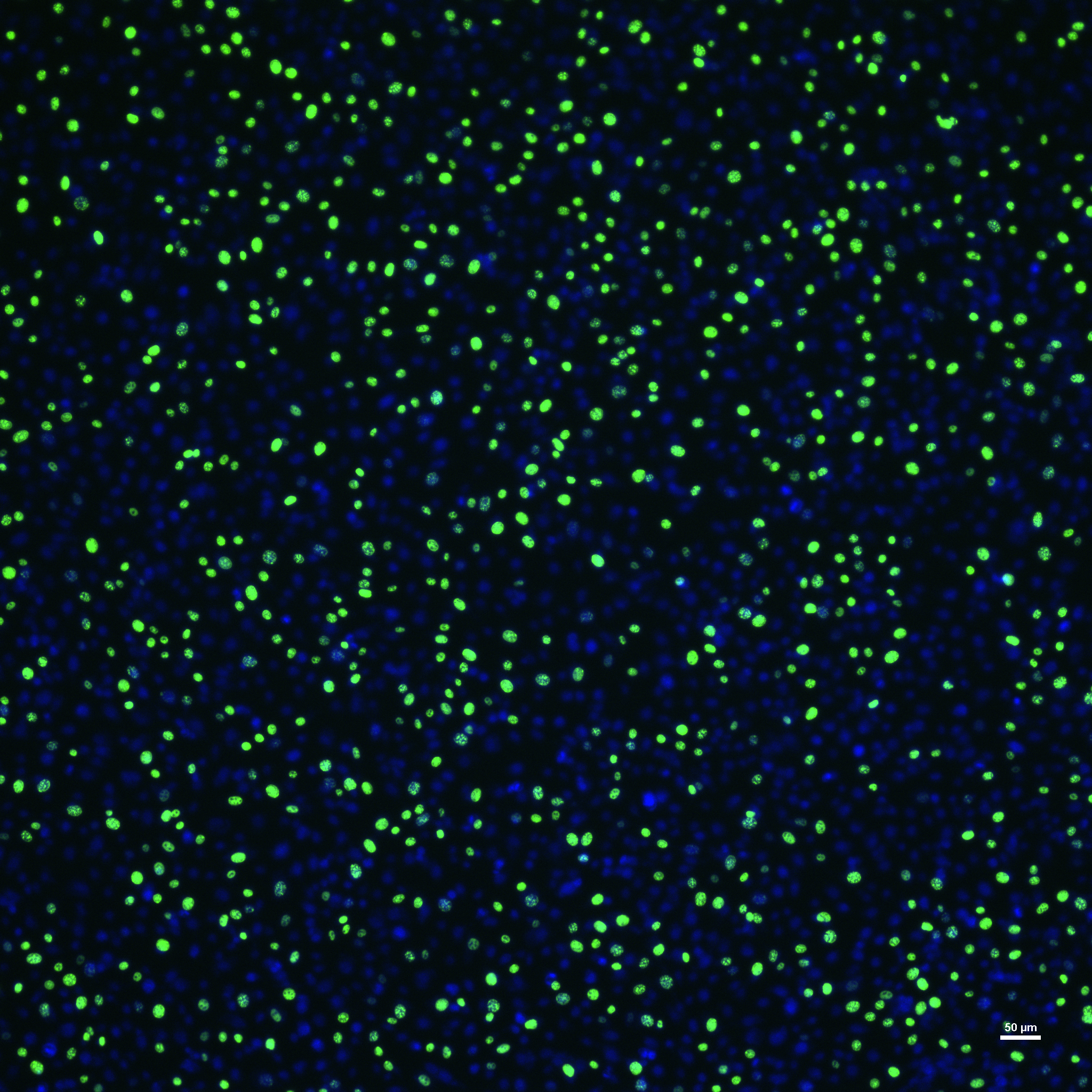

Supplement: Supplementary file 13 — Figure EV2 Source Data [file 44319_2024_197_MOESM13_ESM.zip › Figure EV2/EV2H-I/EdU Staining images/Control siRNA-3.tif]

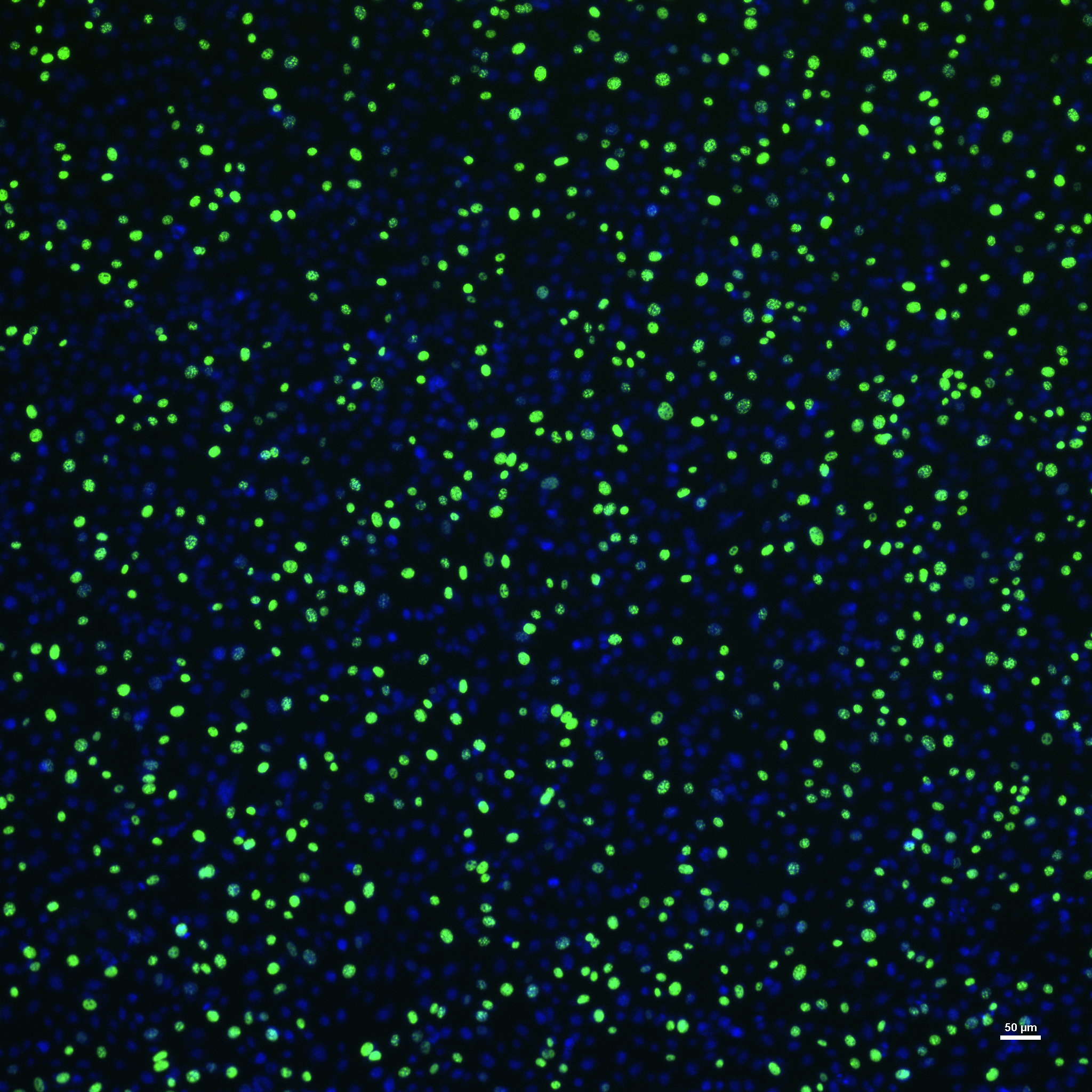

Supplement: Supplementary file 13 — Figure EV2 Source Data [file 44319_2024_197_MOESM13_ESM.zip › Figure EV2/EV2H-I/EdU Staining images/Control siRNA-4.tif]

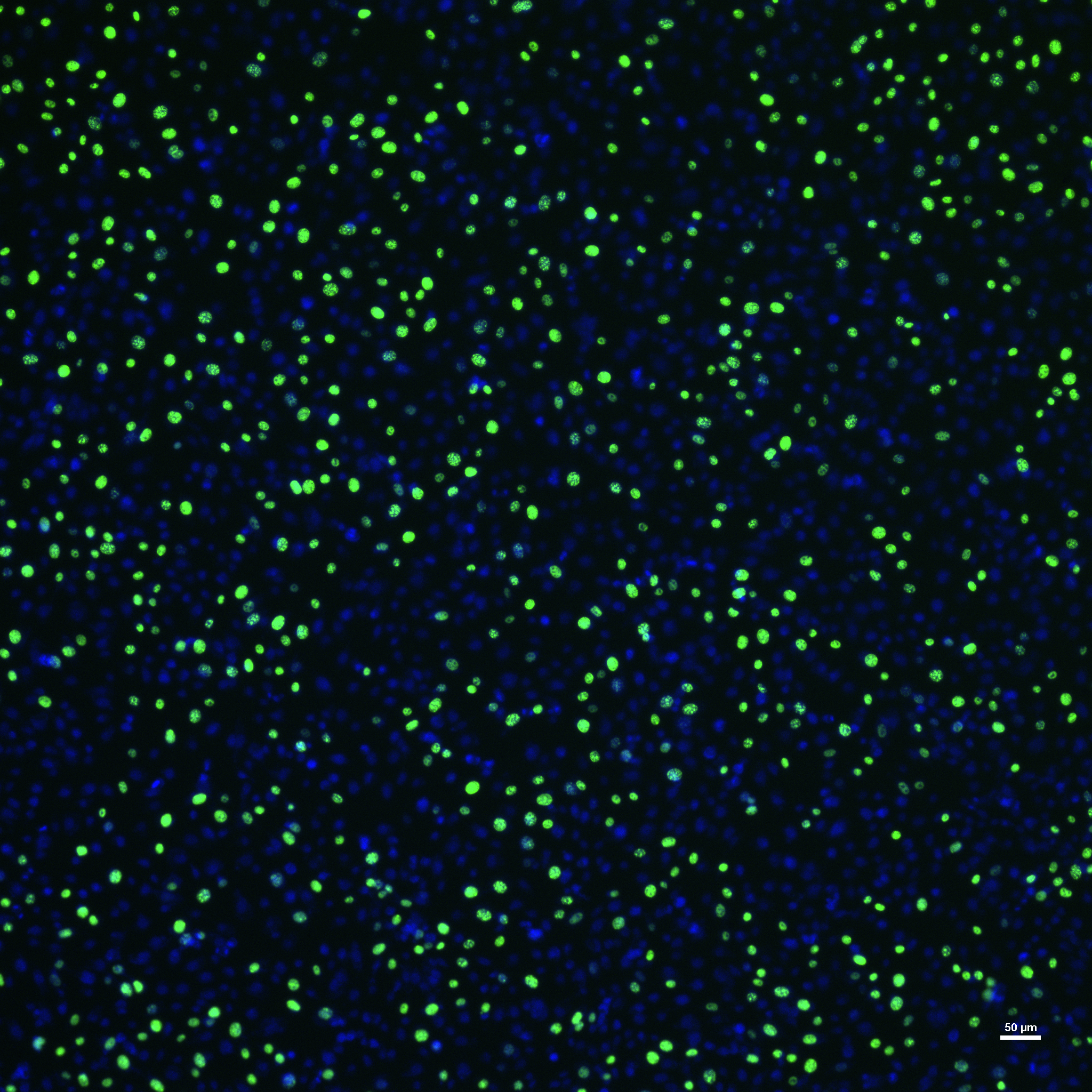

Supplement: Supplementary file 13 — Figure EV2 Source Data [file 44319_2024_197_MOESM13_ESM.zip › Figure EV2/EV2H-I/EdU Staining images/Control siRNA-5.tif]

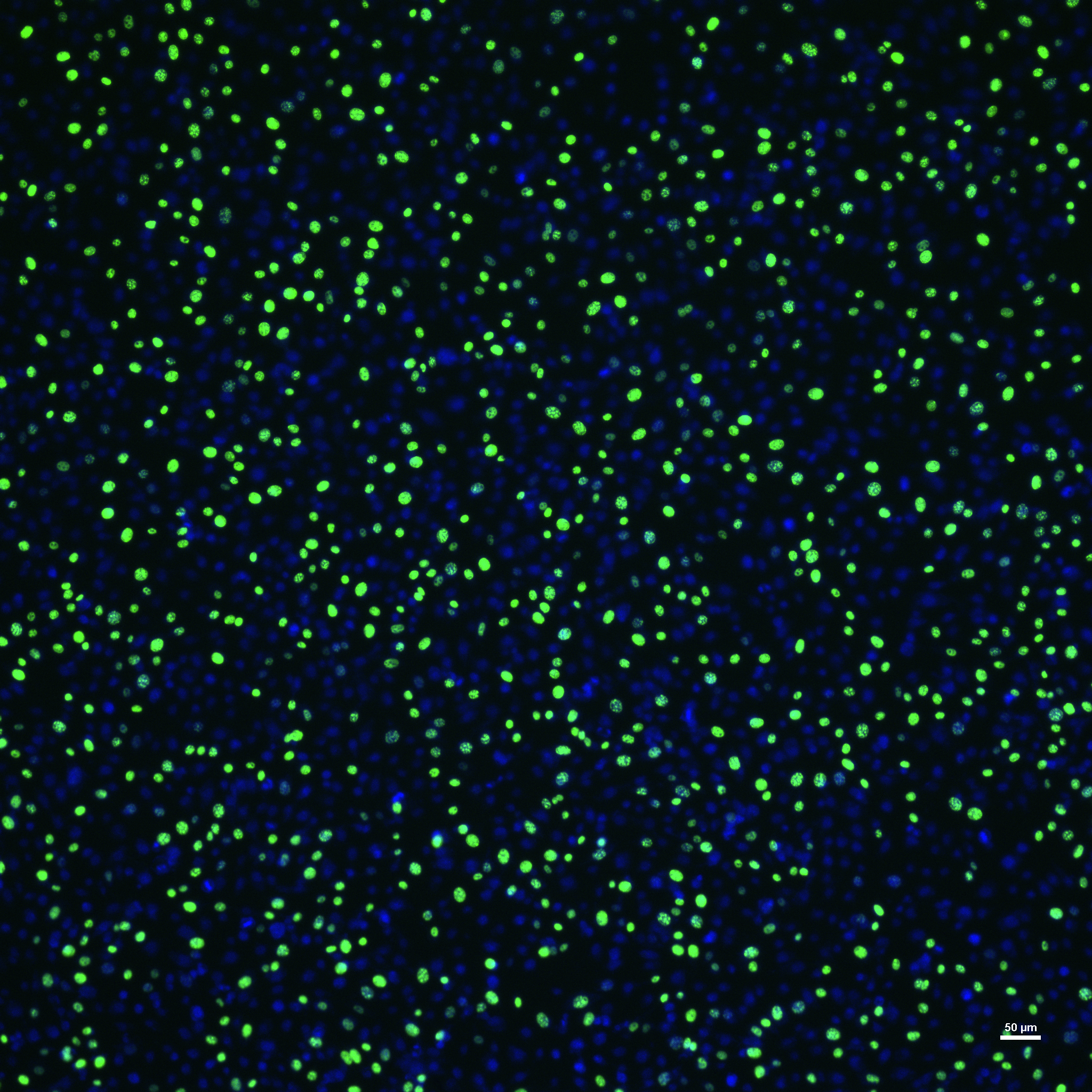

Supplement: Supplementary file 13 — Figure EV2 Source Data [file 44319_2024_197_MOESM13_ESM.zip › Figure EV2/EV2H-I/EdU Staining images/Control siRNA-6.tif]

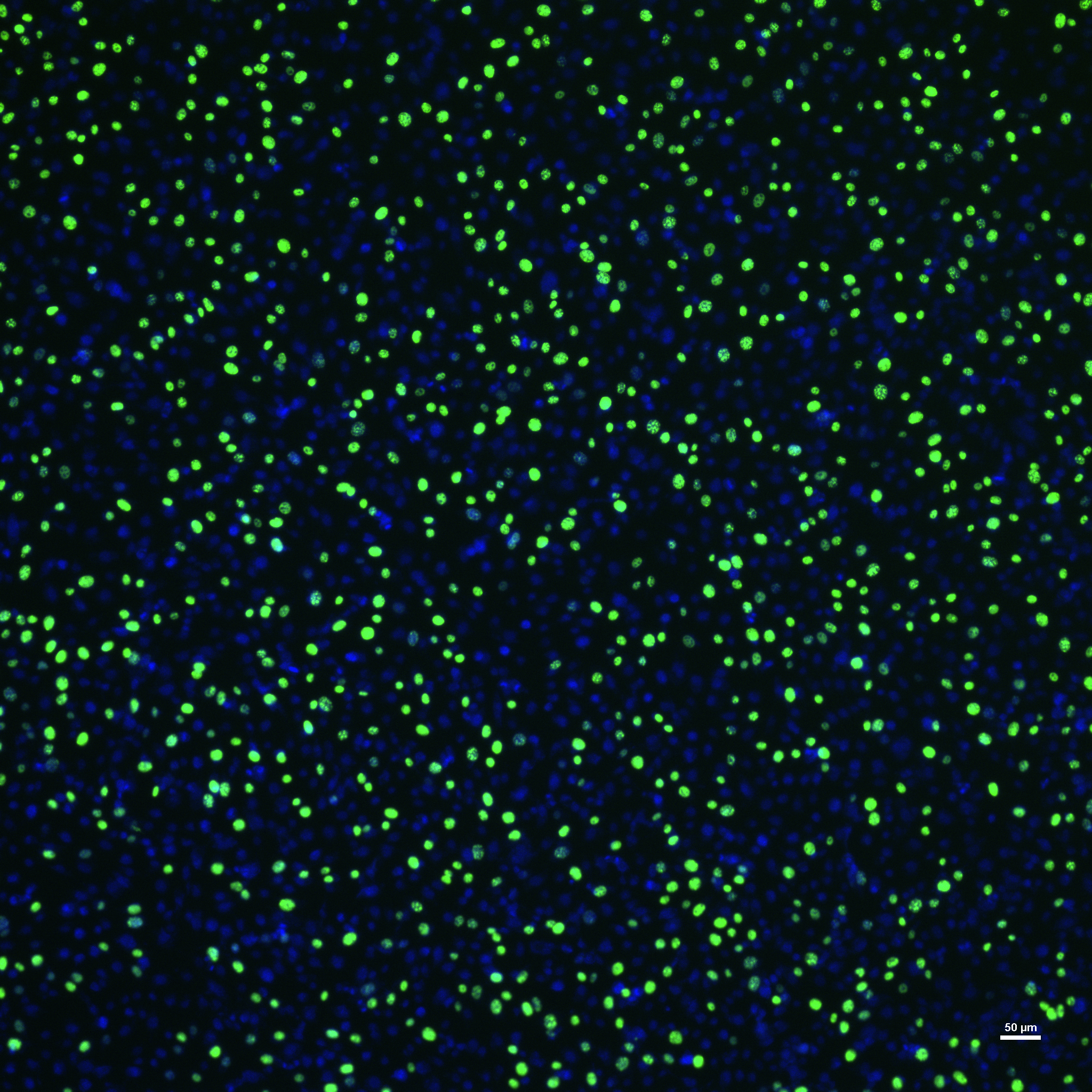

Supplement: Supplementary file 13 — Figure EV2 Source Data [file 44319_2024_197_MOESM13_ESM.zip › Figure EV2/EV2H-I/EdU Staining images/IRE1a siRNA Representative image.tif]

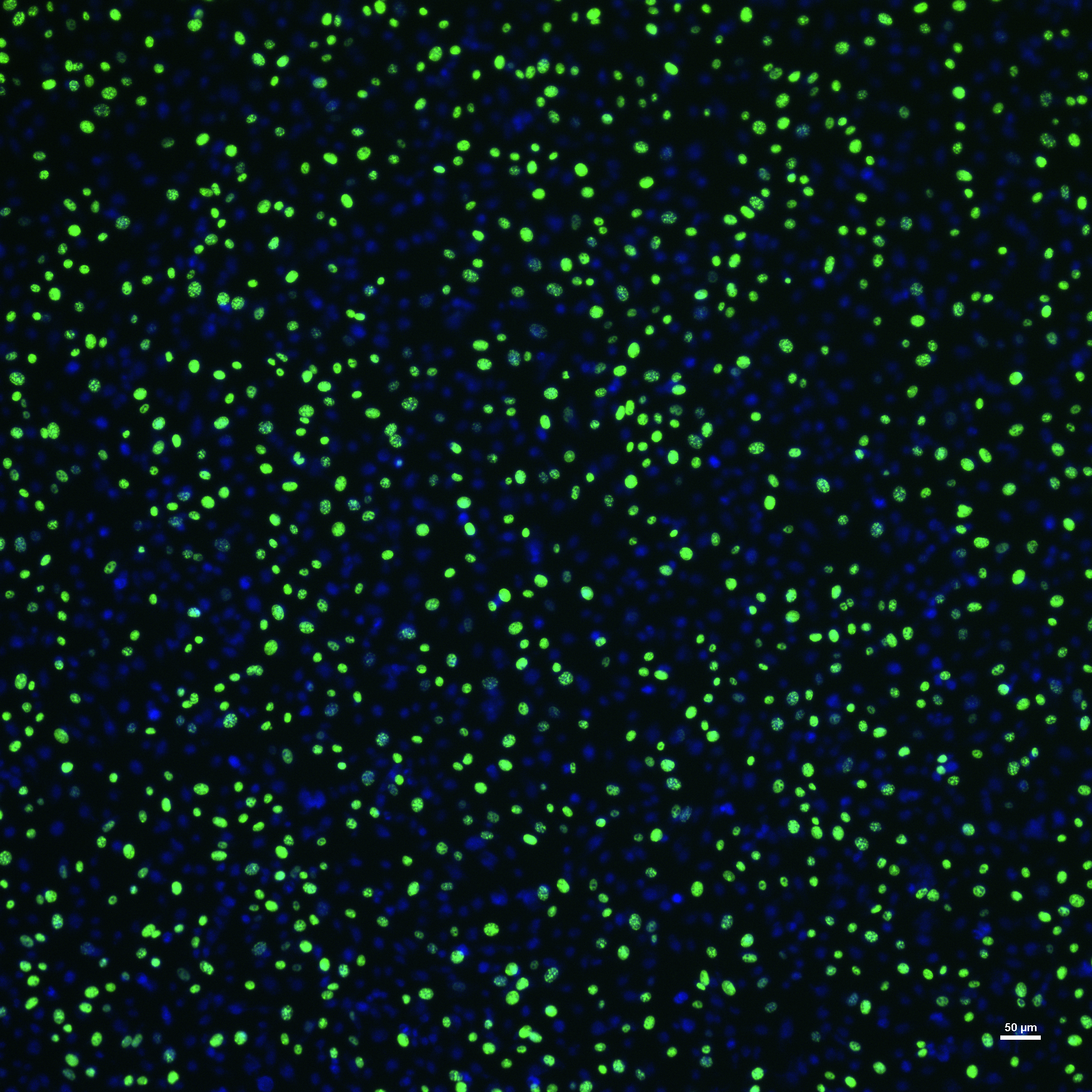

Supplement: Supplementary file 13 — Figure EV2 Source Data [file 44319_2024_197_MOESM13_ESM.zip › Figure EV2/EV2H-I/EdU Staining images/IRE1a siRNA-2.tif]

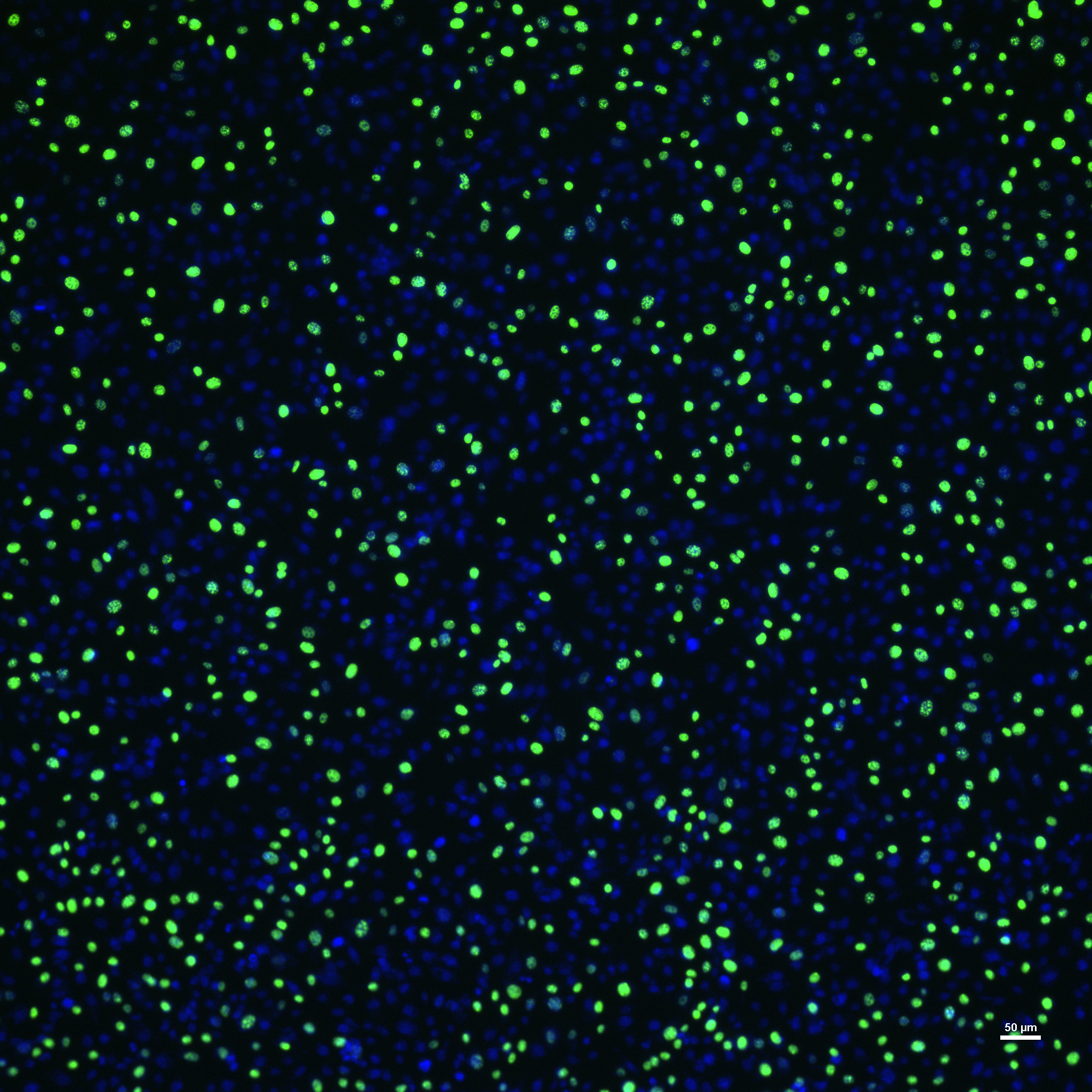

Supplement: Supplementary file 13 — Figure EV2 Source Data [file 44319_2024_197_MOESM13_ESM.zip › Figure EV2/EV2H-I/EdU Staining images/IRE1a siRNA-3.tif]

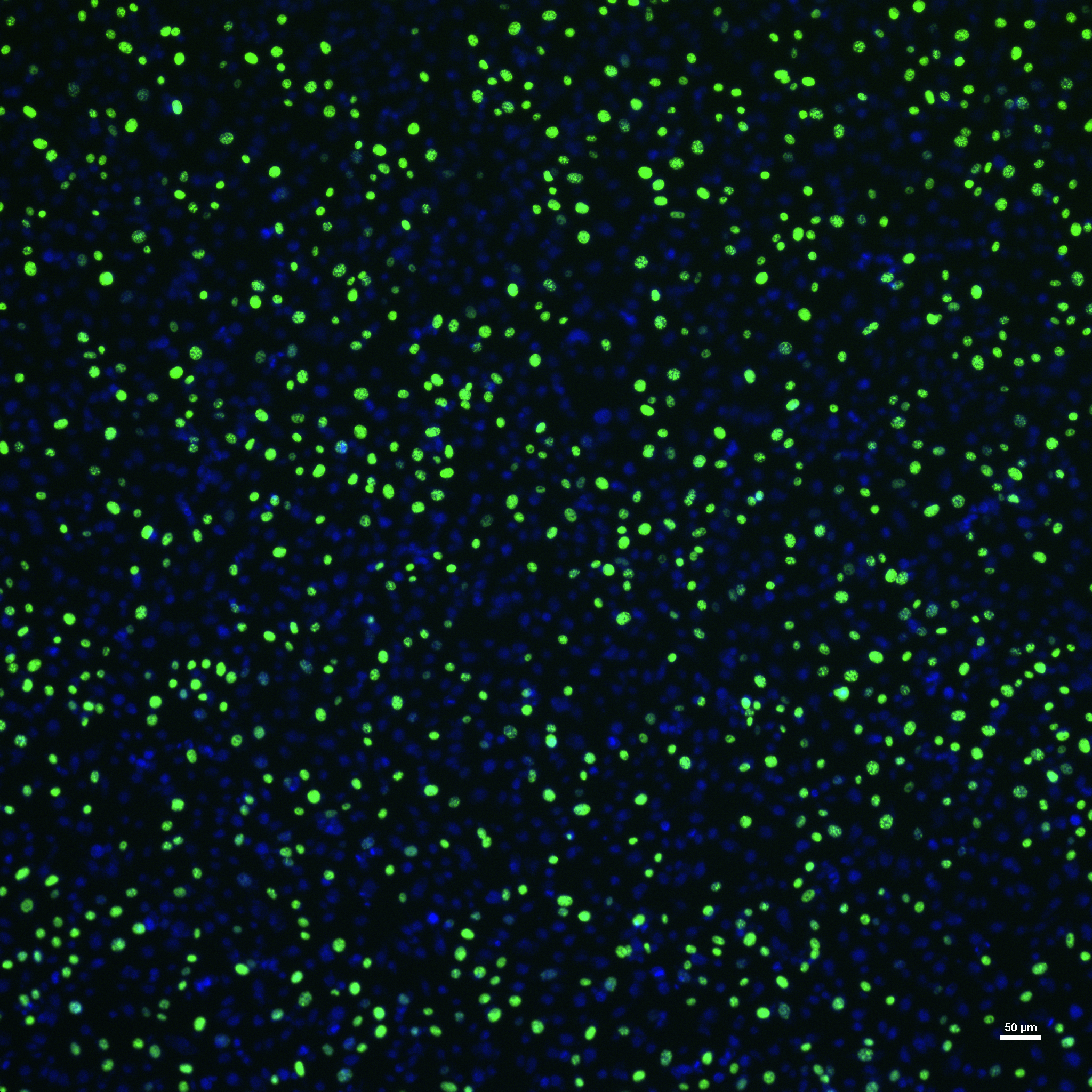

Supplement: Supplementary file 13 — Figure EV2 Source Data [file 44319_2024_197_MOESM13_ESM.zip › Figure EV2/EV2H-I/EdU Staining images/IRE1a siRNA-4.tif]

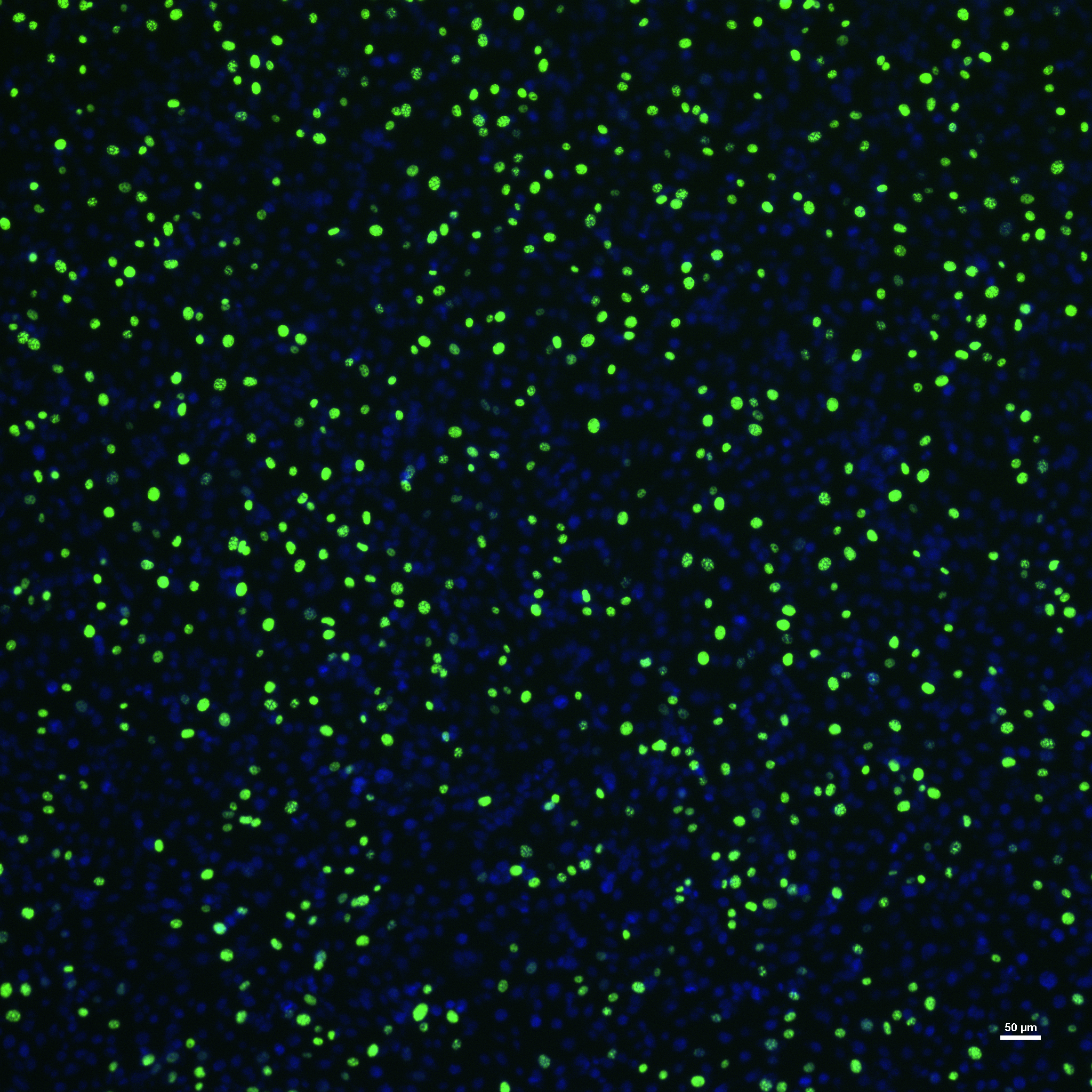

Supplement: Supplementary file 13 — Figure EV2 Source Data [file 44319_2024_197_MOESM13_ESM.zip › Figure EV2/EV2H-I/EdU Staining images/IRE1a siRNA-5.tif]

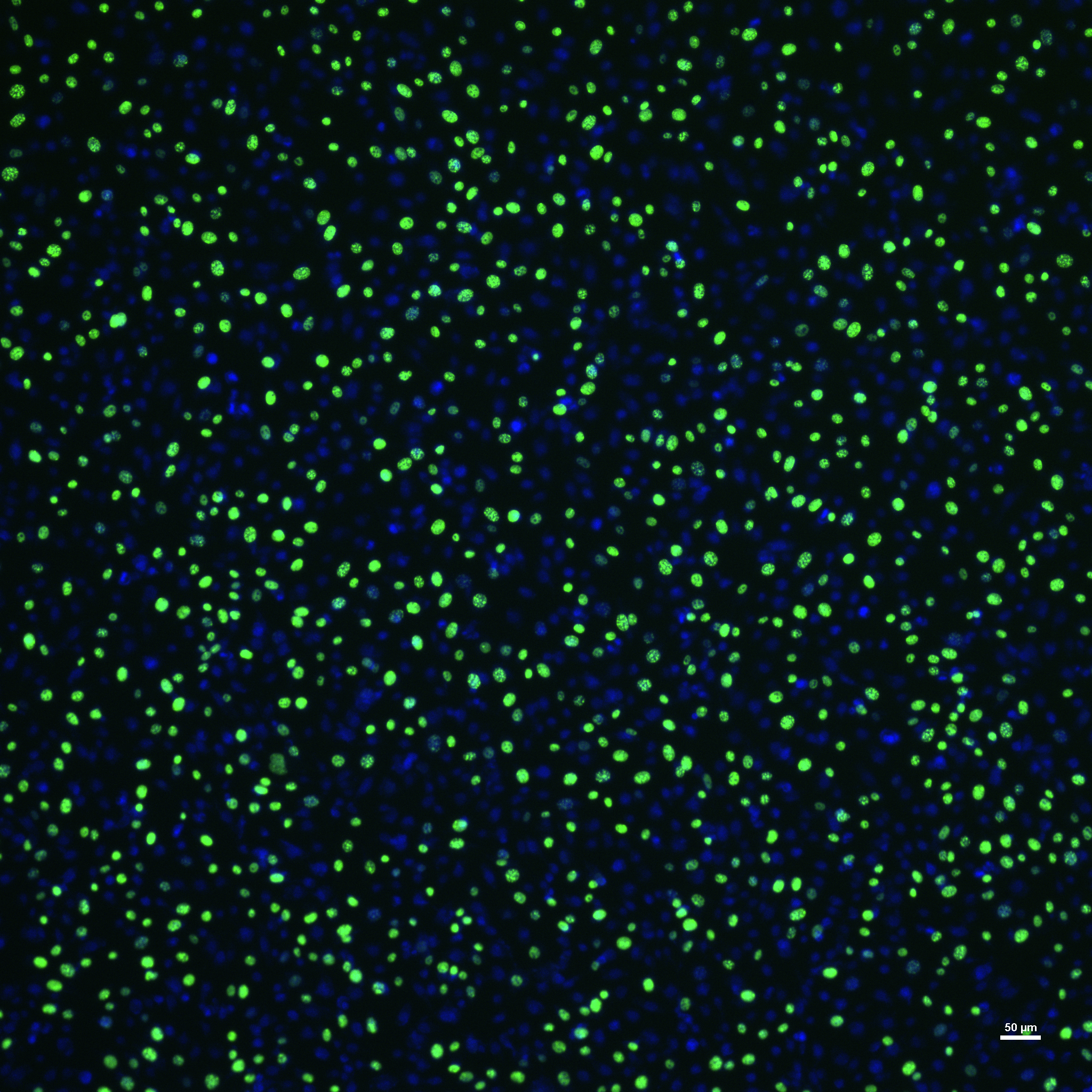

Supplement: Supplementary file 13 — Figure EV2 Source Data [file 44319_2024_197_MOESM13_ESM.zip › Figure EV2/EV2H-I/EdU Staining images/IRE1a siRNA-6.tif]

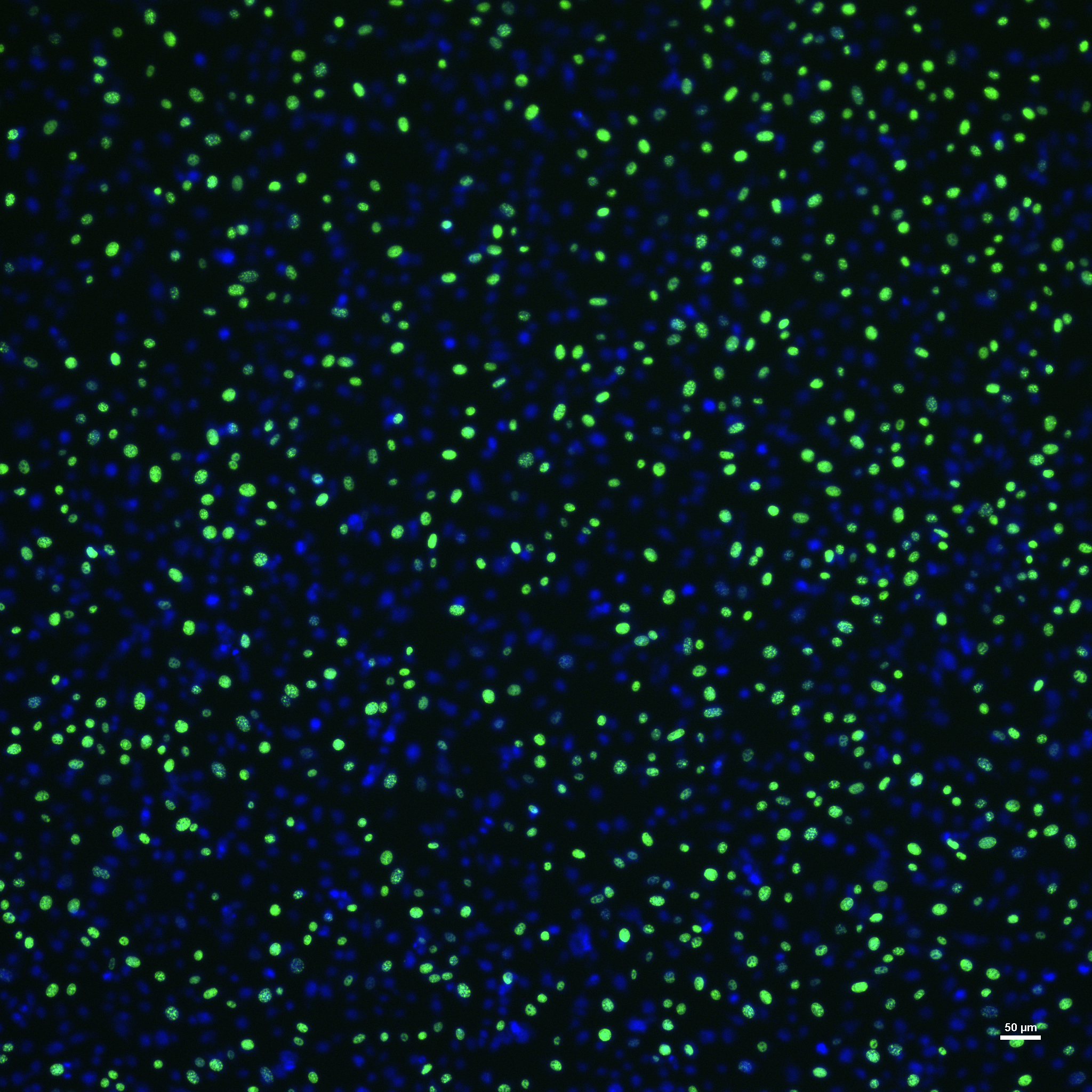

Supplement: Supplementary file 13 — Figure EV2 Source Data [file 44319_2024_197_MOESM13_ESM.zip › Figure EV2/EV2H-I/EdU Staining images/IRE1a siRNA-7.tif]

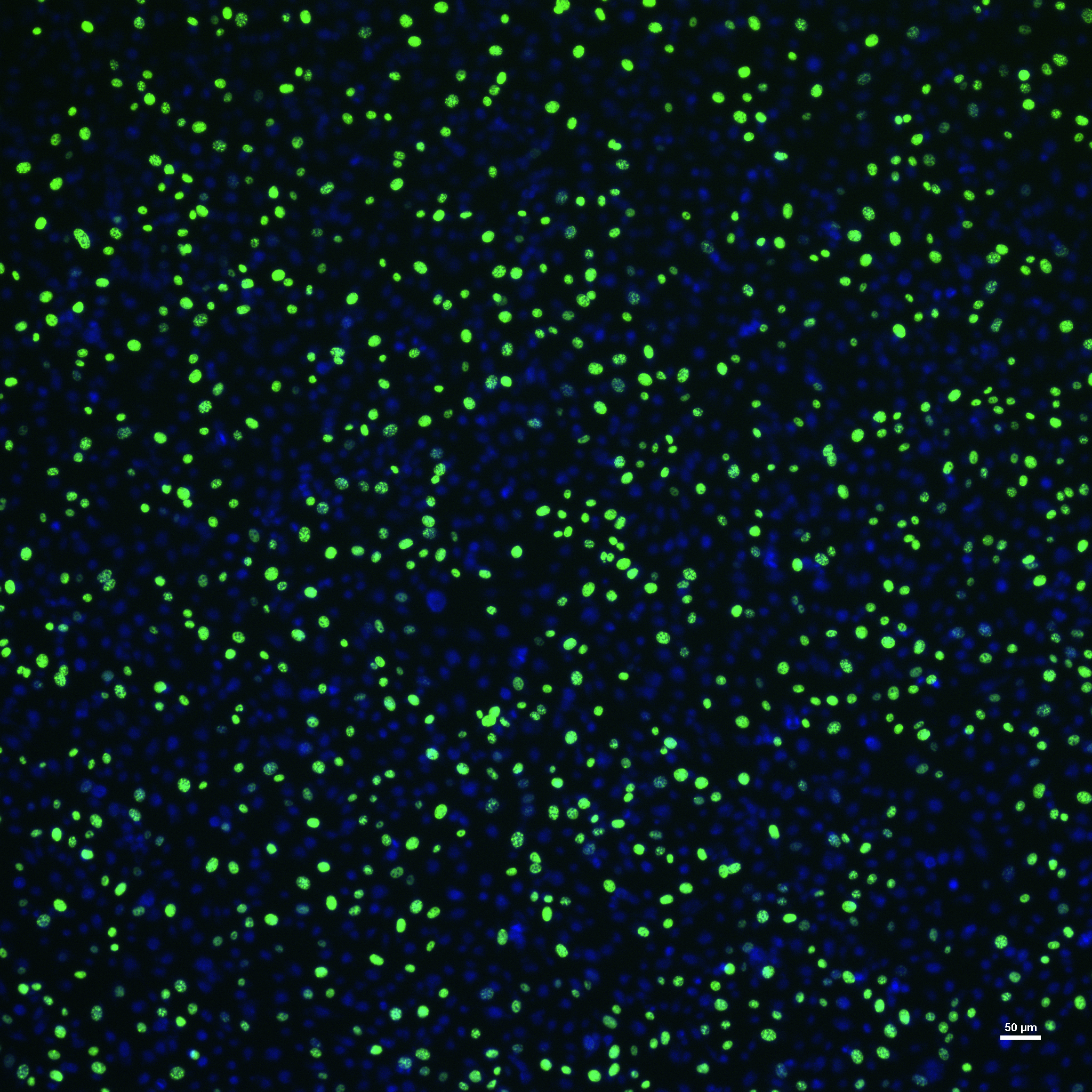

Supplement: Supplementary file 13 — Figure EV2 Source Data [file 44319_2024_197_MOESM13_ESM.zip › Figure EV2/EV2H-I/EdU Staining images/IRE1a siRNA-8.tif]

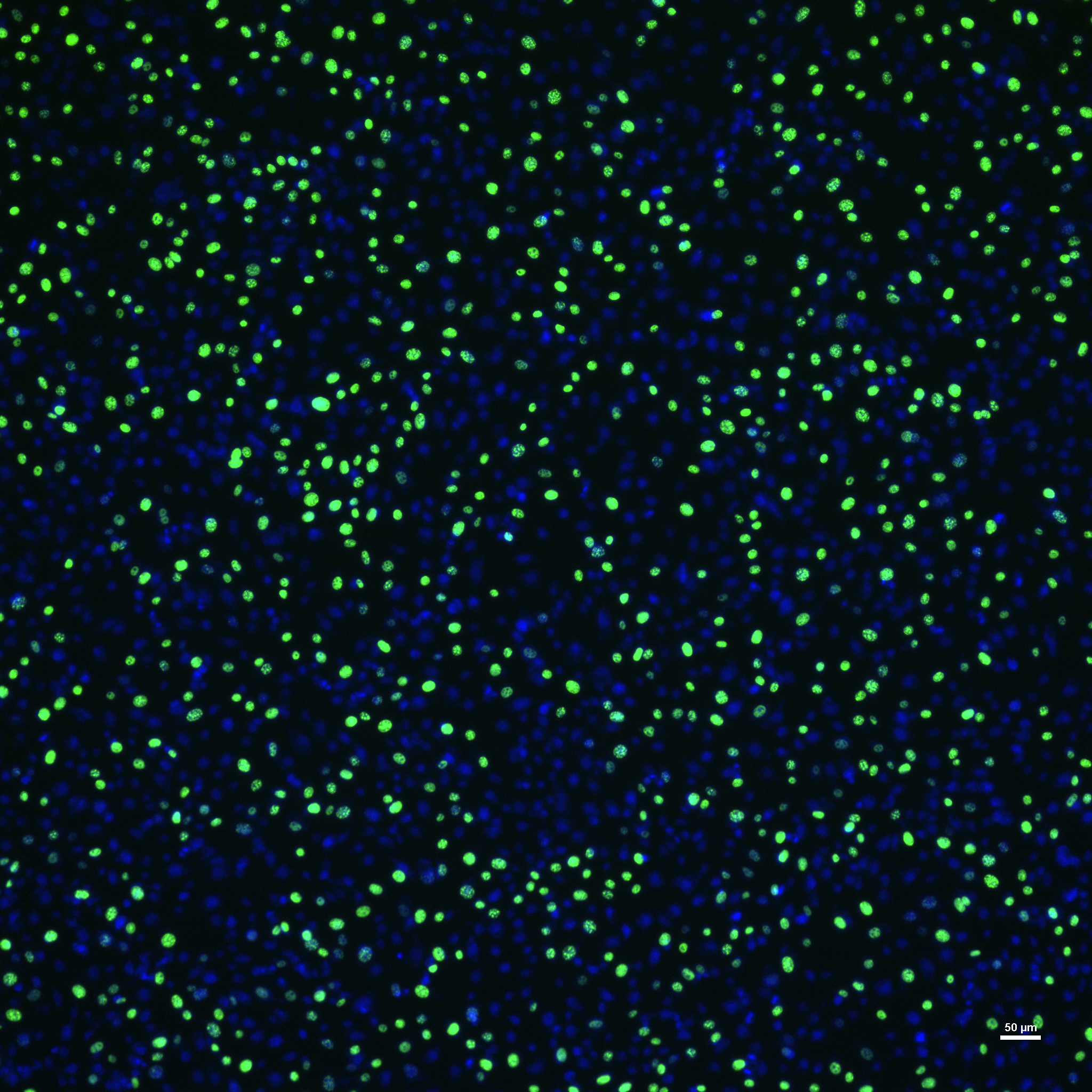

Supplement: Supplementary file 13 — Figure EV2 Source Data [file 44319_2024_197_MOESM13_ESM.zip › Figure EV2/EV2H-I/EdU Staining images/IRE1a siRNA-9.tif]

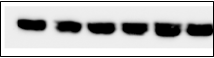

Supplement: Supplementary file 14 — Figure EV3 Source Data [file 44319_2024_197_MOESM14_ESM.zip › Figure EV3/EV3A/GAPDH - Western.tif]

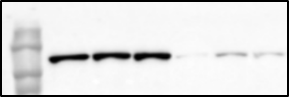

Supplement: Supplementary file 14 — Figure EV3 Source Data [file 44319_2024_197_MOESM14_ESM.zip › Figure EV3/EV3A/IRE1a - Western.tif]

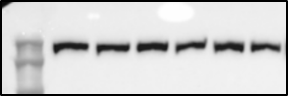

Supplement: Supplementary file 14 — Figure EV3 Source Data [file 44319_2024_197_MOESM14_ESM.zip › Figure EV3/EV3A/MyHC - Western.tif]

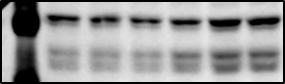

Supplement: Supplementary file 14 — Figure EV3 Source Data [file 44319_2024_197_MOESM14_ESM.zip › Figure EV3/EV3A/MyoD - Western.tif]

## Slide 1
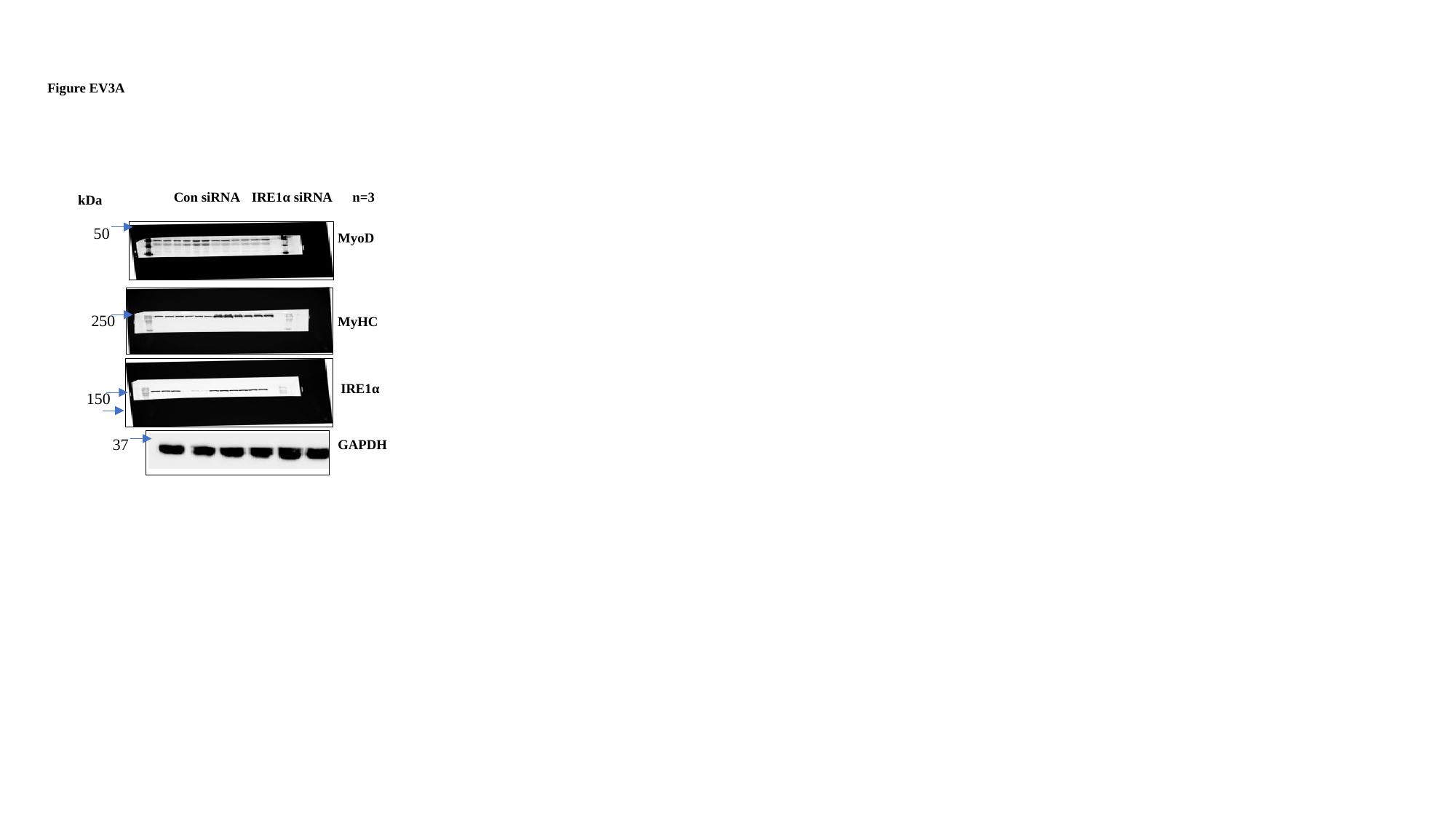

Figure EV3A
Con siRNA
IRE1α siRNA n=3
kDa
50
MyoD
250
MyHC
IRE1α
150
37
GAPDH

Supplement: Supplementary file 14 — Figure EV3 Source Data [file 44319_2024_197_MOESM14_ESM.zip › Figure EV3/EV3A/Western blot with annotation.pptx]

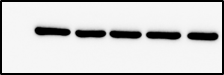

Supplement: Supplementary file 14 — Figure EV3 Source Data [file 44319_2024_197_MOESM14_ESM.zip › Figure EV3/EV3B/GAPDH - Western.tif]

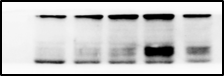

Supplement: Supplementary file 14 — Figure EV3 Source Data [file 44319_2024_197_MOESM14_ESM.zip › Figure EV3/EV3B/sXBP1 - Western.tif]

## Slide 1
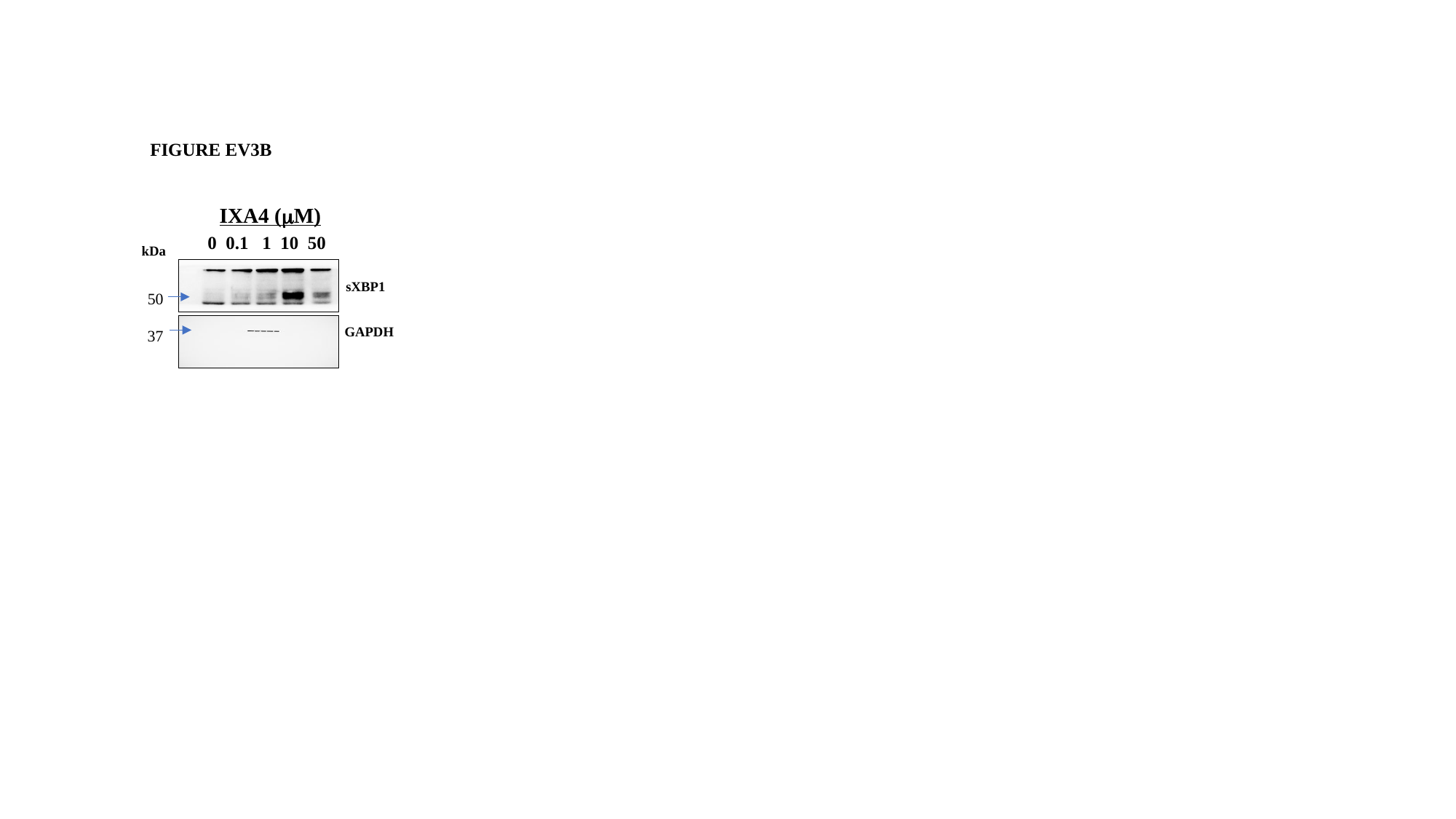

FIGURE EV3B
IXA4 (μM)
0 0.1 1 10 50
kDa
sXBP1
50
GAPDH
37

Supplement: Supplementary file 14 — Figure EV3 Source Data [file 44319_2024_197_MOESM14_ESM.zip › Figure EV3/EV3B/Western blot with Annotation.pptx]

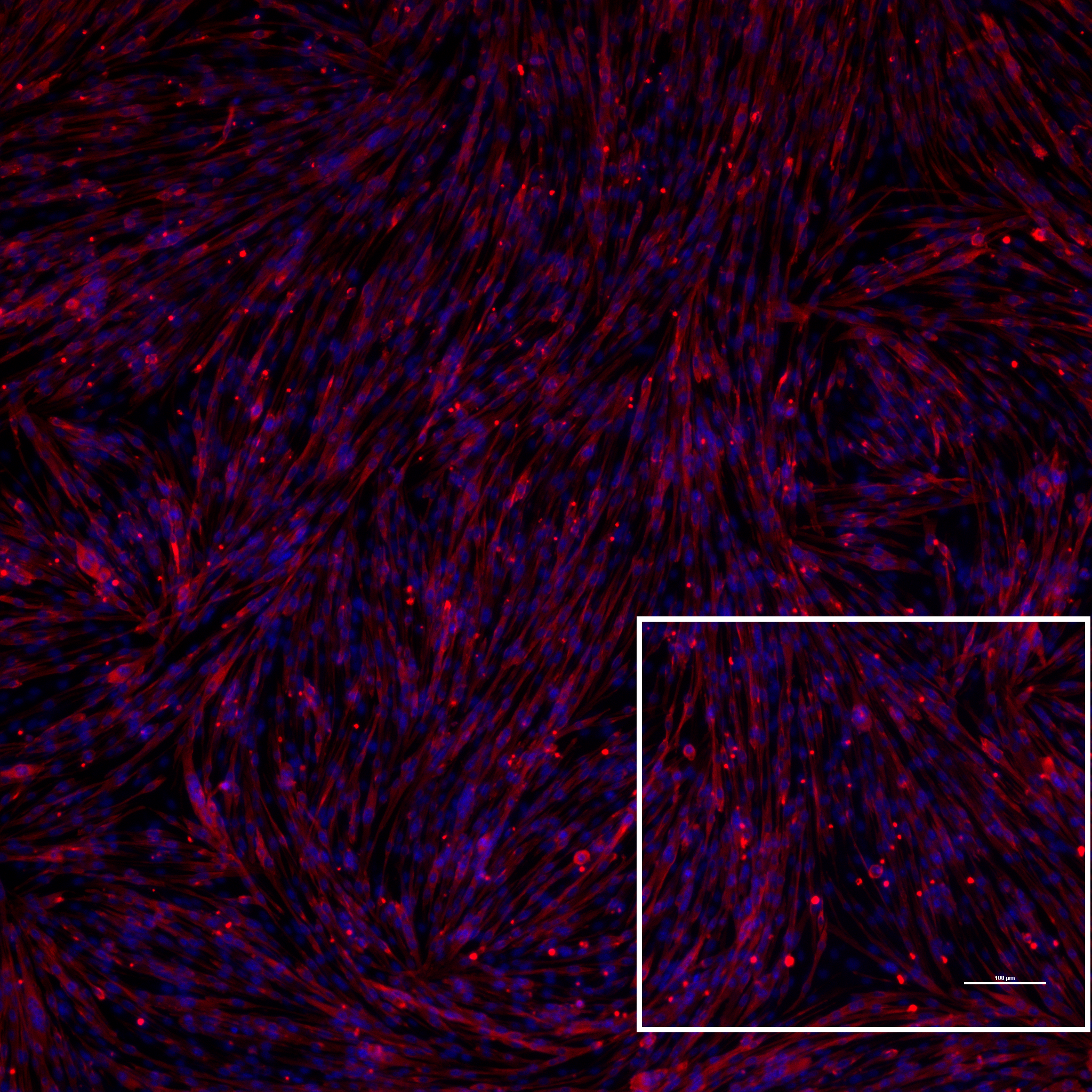

Supplement: Supplementary file 14 — Figure EV3 Source Data [file 44319_2024_197_MOESM14_ESM.zip › Figure EV3/EV3C-E/IXA4 treatment-MyHC staining images/48 h Control Representative image with box.tiff]

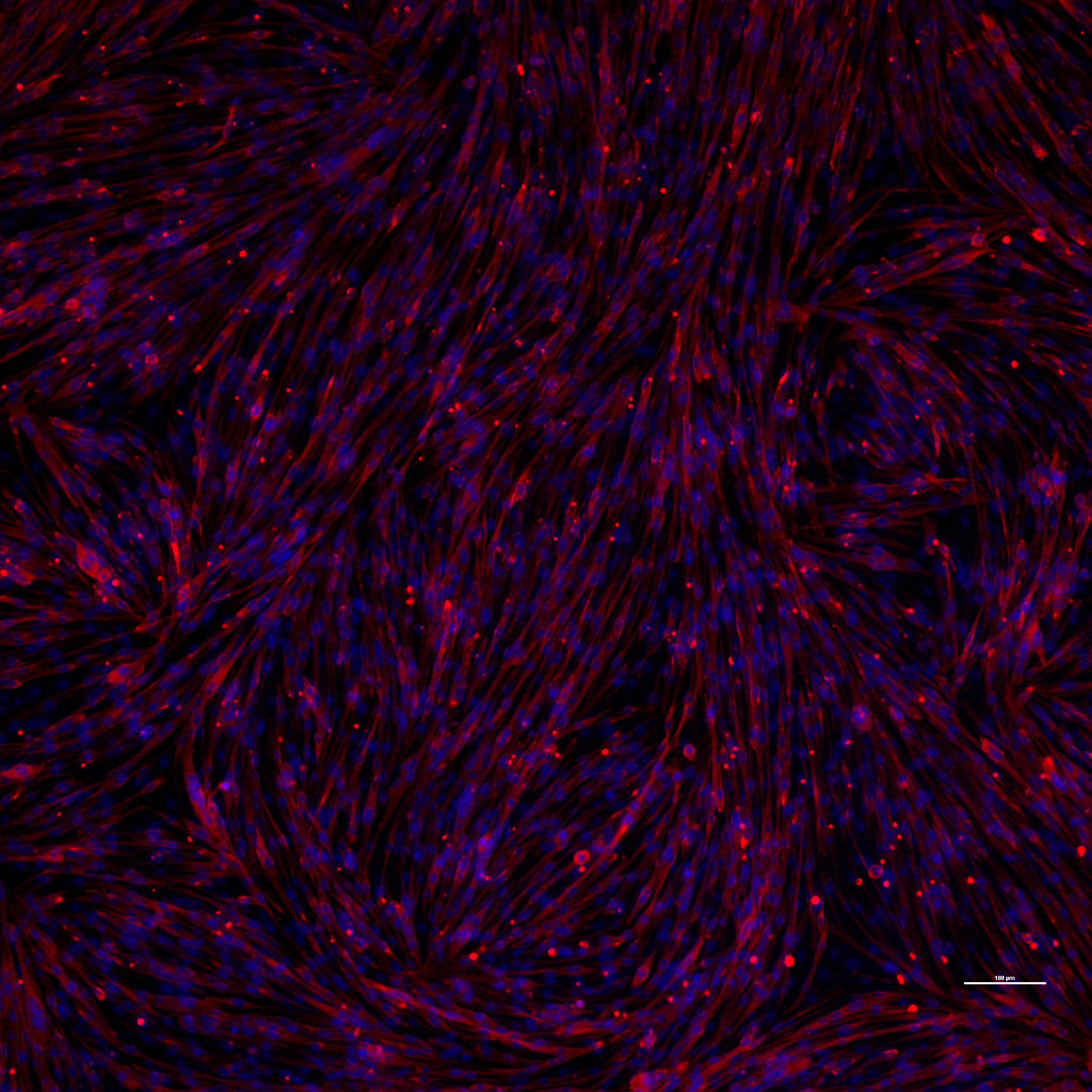

Supplement: Supplementary file 14 — Figure EV3 Source Data [file 44319_2024_197_MOESM14_ESM.zip › Figure EV3/EV3C-E/IXA4 treatment-MyHC staining images/48 h Control Representative image.tif]

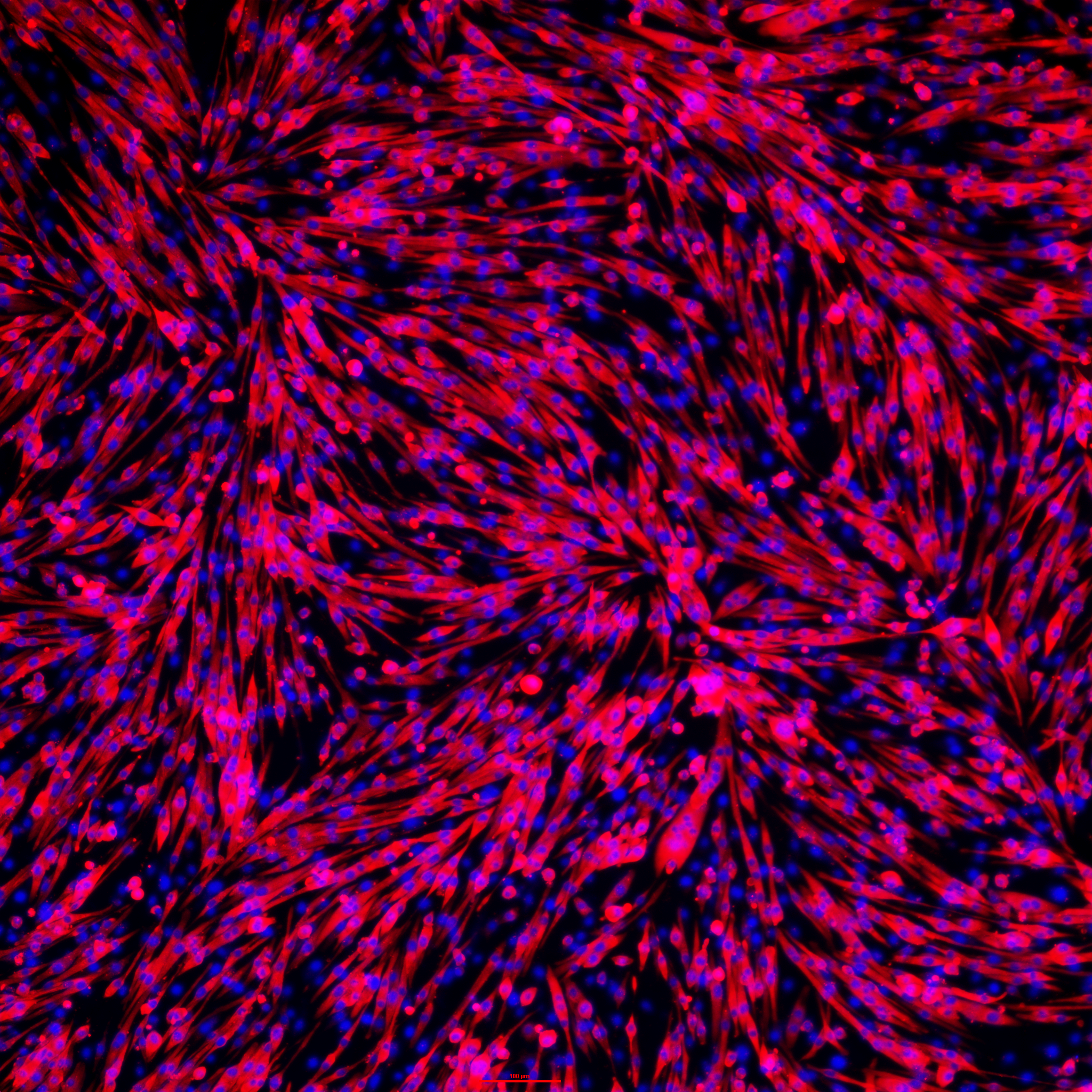

Supplement: Supplementary file 14 — Figure EV3 Source Data [file 44319_2024_197_MOESM14_ESM.zip › Figure EV3/EV3C-E/IXA4 treatment-MyHC staining images/48 h Control-2.tif]

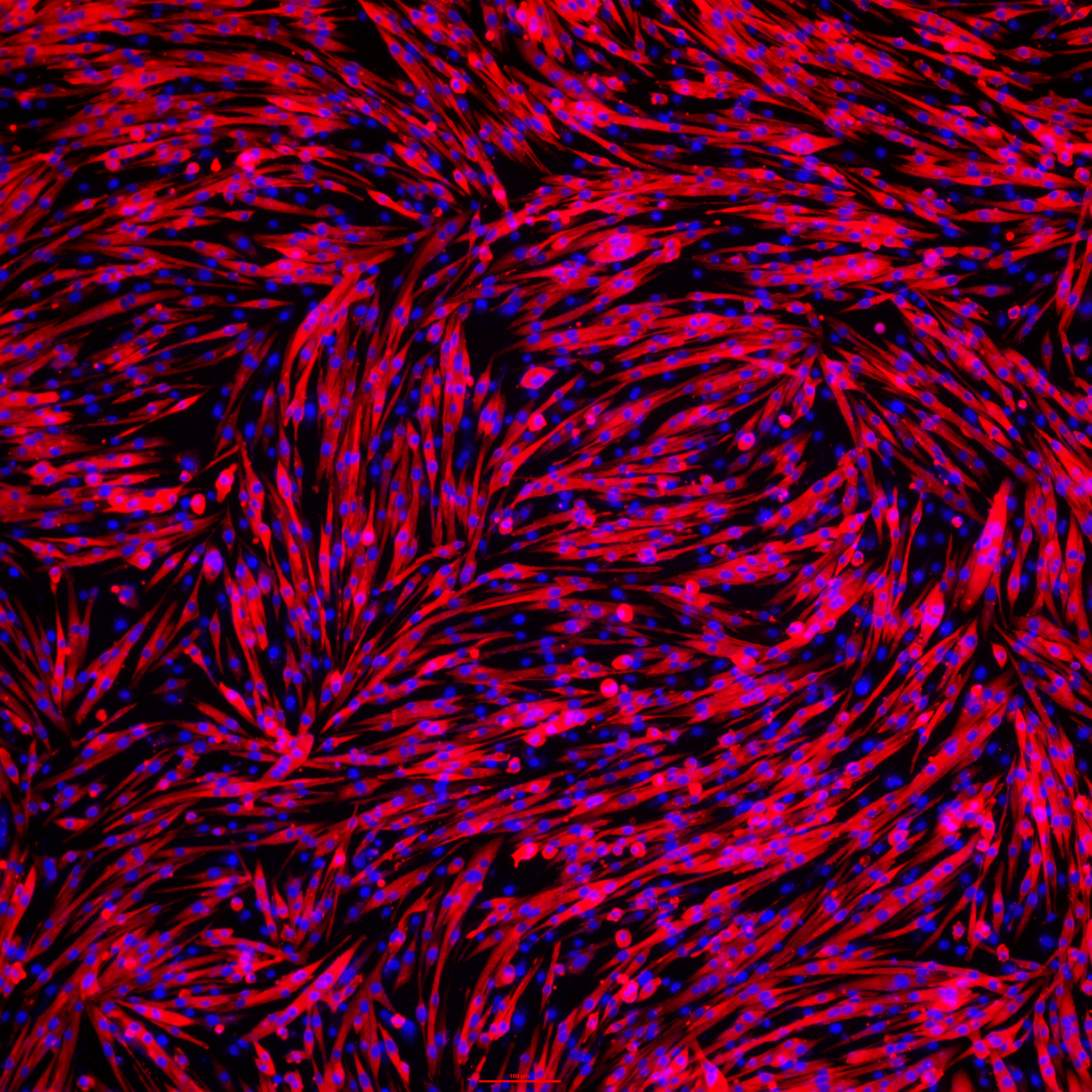

Supplement: Supplementary file 14 — Figure EV3 Source Data [file 44319_2024_197_MOESM14_ESM.zip › Figure EV3/EV3C-E/IXA4 treatment-MyHC staining images/48 h Control-3.tif]

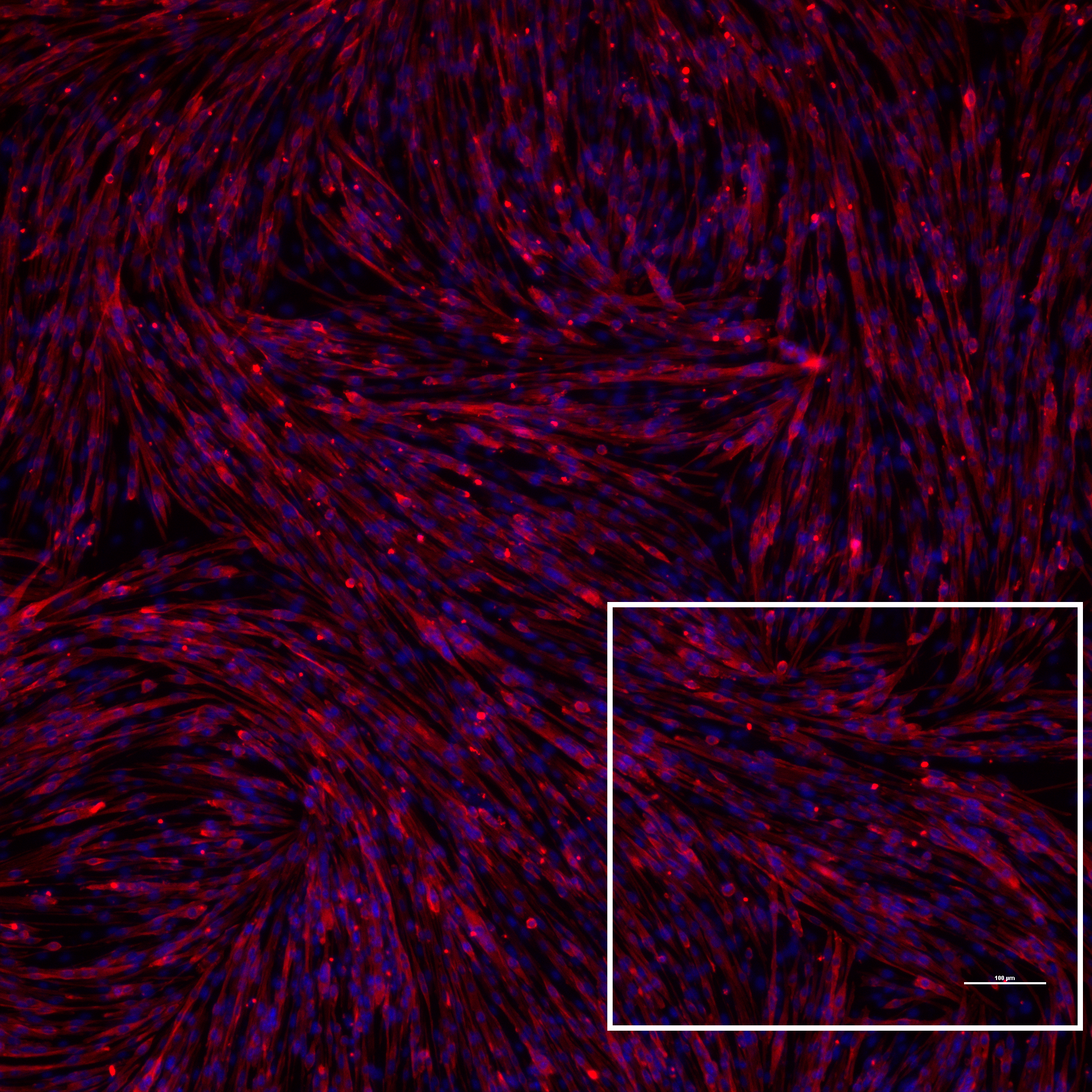

Supplement: Supplementary file 14 — Figure EV3 Source Data [file 44319_2024_197_MOESM14_ESM.zip › Figure EV3/EV3C-E/IXA4 treatment-MyHC staining images/48 h IXA4 Representative image with box.tiff]

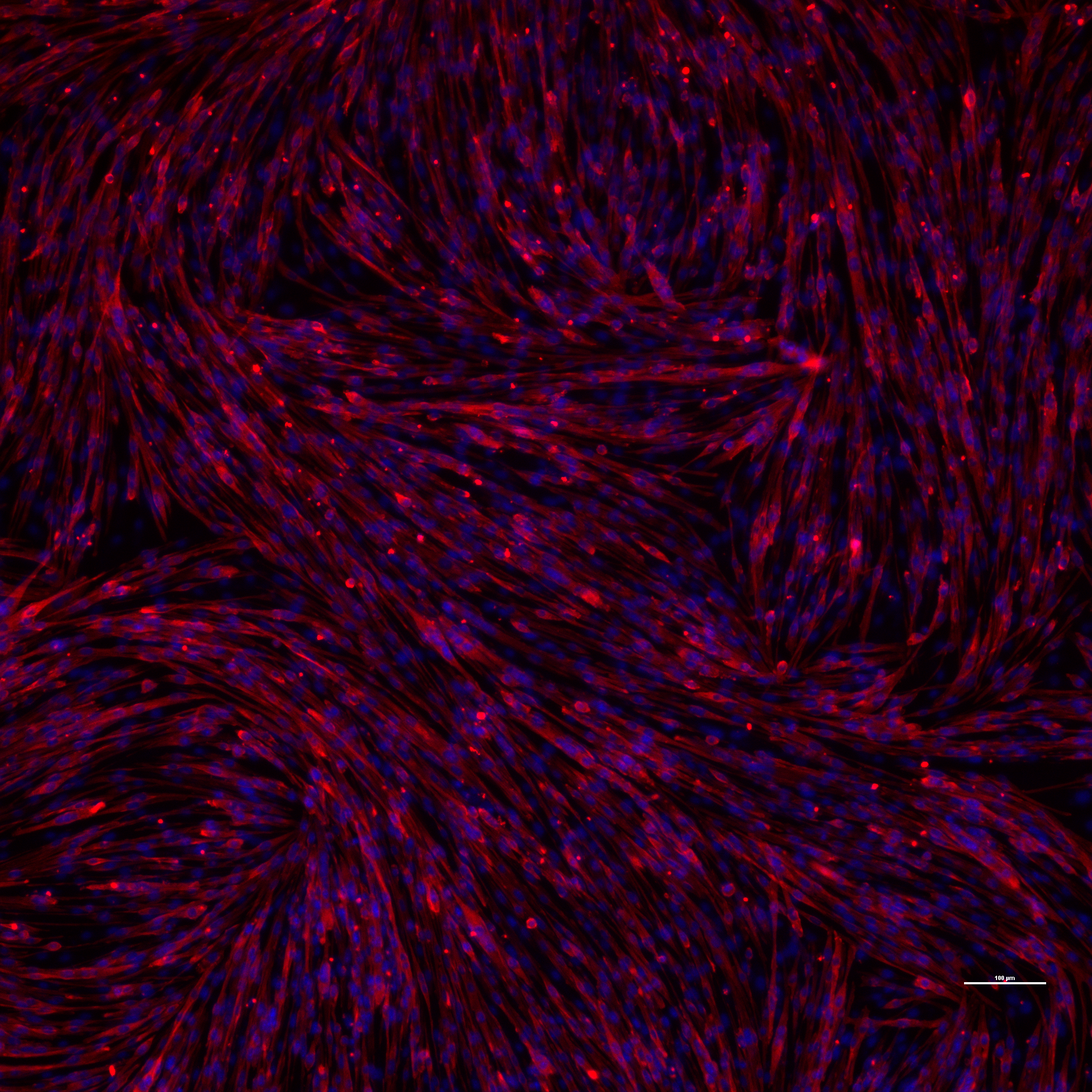

Supplement: Supplementary file 14 — Figure EV3 Source Data [file 44319_2024_197_MOESM14_ESM.zip › Figure EV3/EV3C-E/IXA4 treatment-MyHC staining images/48 h IXA4 Representative image.tif]

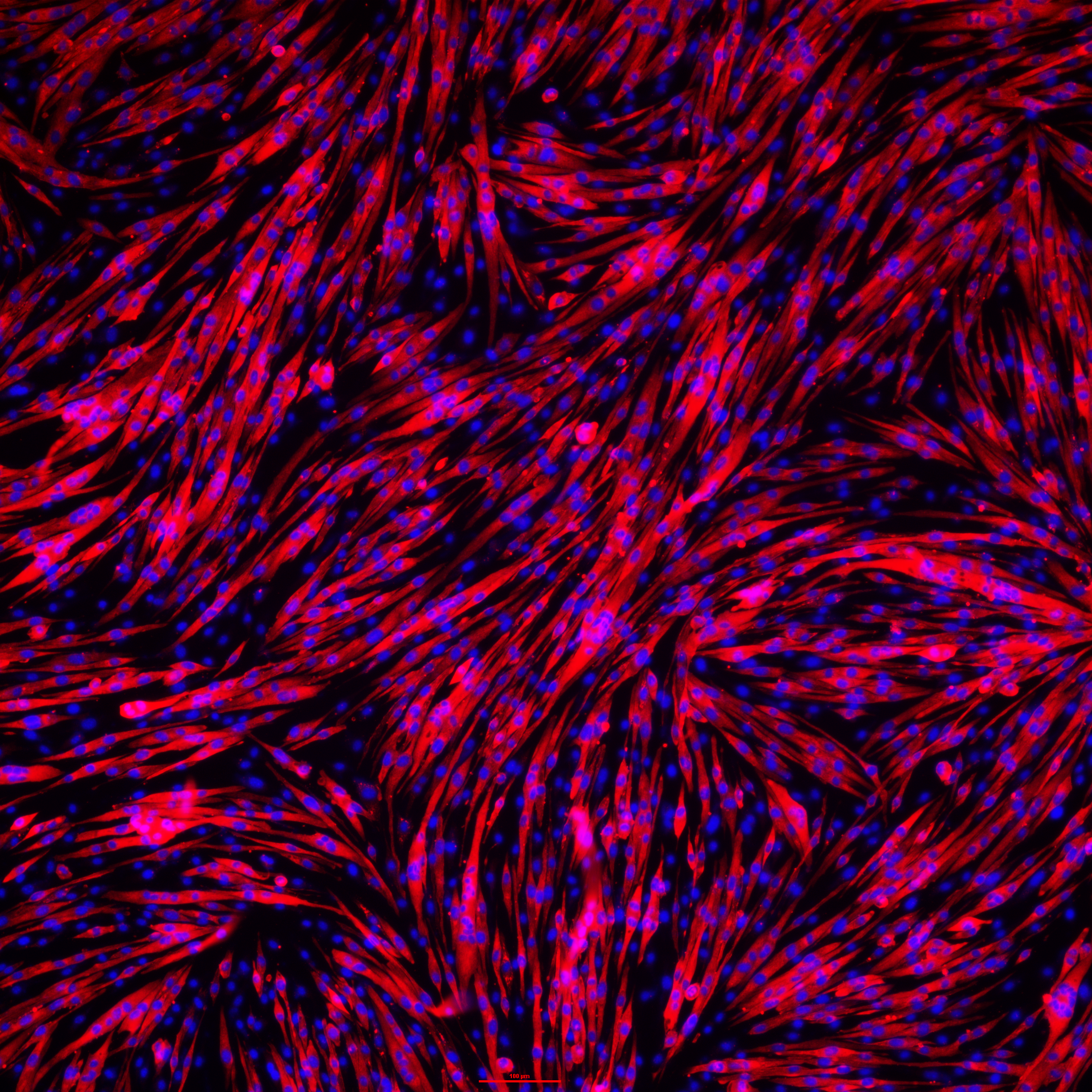

Supplement: Supplementary file 14 — Figure EV3 Source Data [file 44319_2024_197_MOESM14_ESM.zip › Figure EV3/EV3C-E/IXA4 treatment-MyHC staining images/48 h IXA4-2.tif]

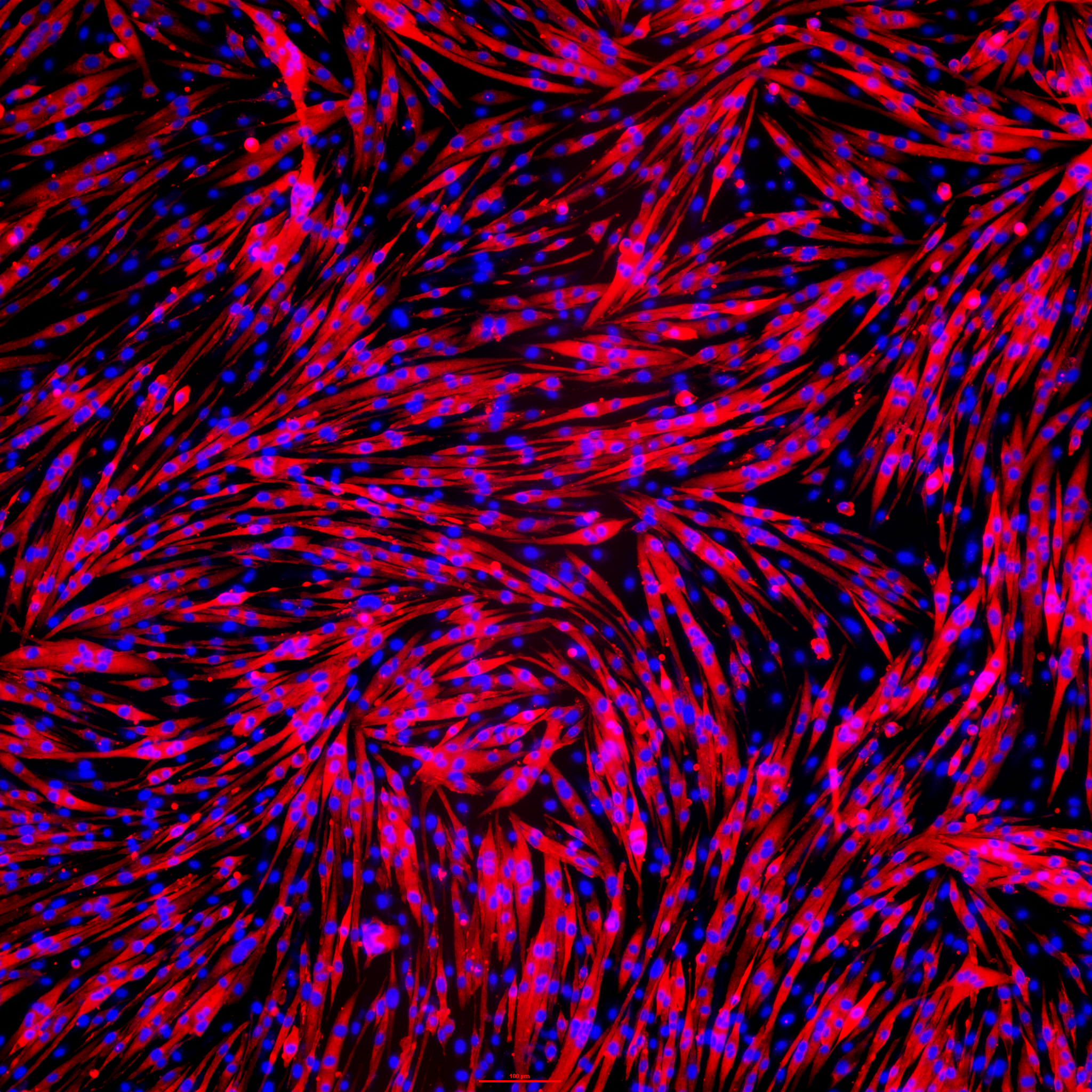

Supplement: Supplementary file 14 — Figure EV3 Source Data [file 44319_2024_197_MOESM14_ESM.zip › Figure EV3/EV3C-E/IXA4 treatment-MyHC staining images/48 h IXA4-3.tif]

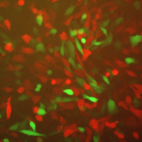

Supplement: Supplementary file 14 — Figure EV3 Source Data [file 44319_2024_197_MOESM14_ESM.zip › Figure EV3/EV3F/Cell mixing experiment- images/0 h GFP control-mCherry control Representative image.tif]

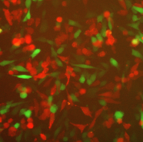

Supplement: Supplementary file 14 — Figure EV3 Source Data [file 44319_2024_197_MOESM14_ESM.zip › Figure EV3/EV3F/Cell mixing experiment- images/0 h GFP control-mCherry IRE1a KD Representative image.tif]

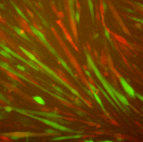

Supplement: Supplementary file 14 — Figure EV3 Source Data [file 44319_2024_197_MOESM14_ESM.zip › Figure EV3/EV3F/Cell mixing experiment- images/48 h GFP control-mCherry control Representative image.tif]

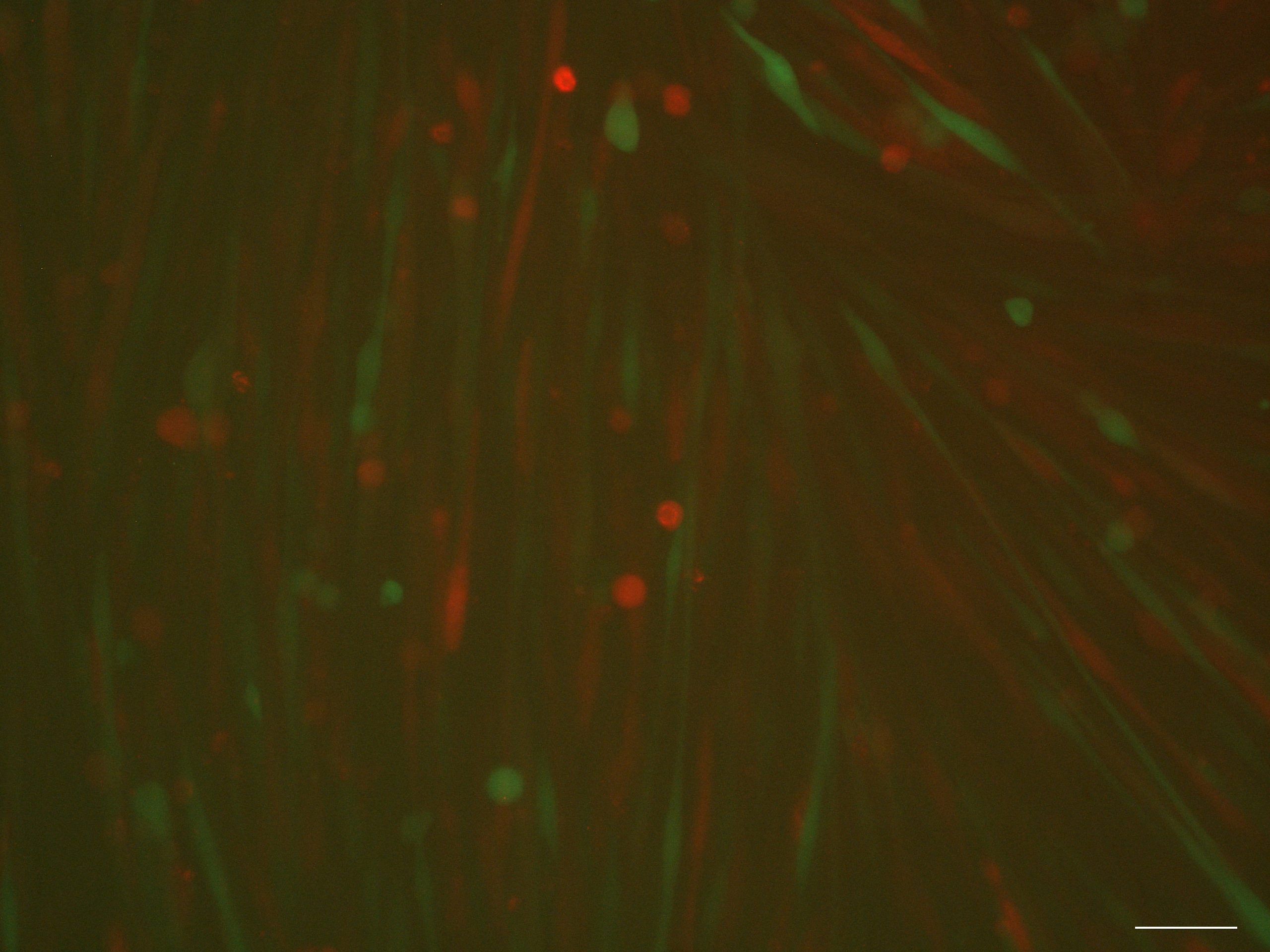

Supplement: Supplementary file 14 — Figure EV3 Source Data [file 44319_2024_197_MOESM14_ESM.zip › Figure EV3/EV3F/Cell mixing experiment- images/48 h GFP control-mCherry control-2.tif]

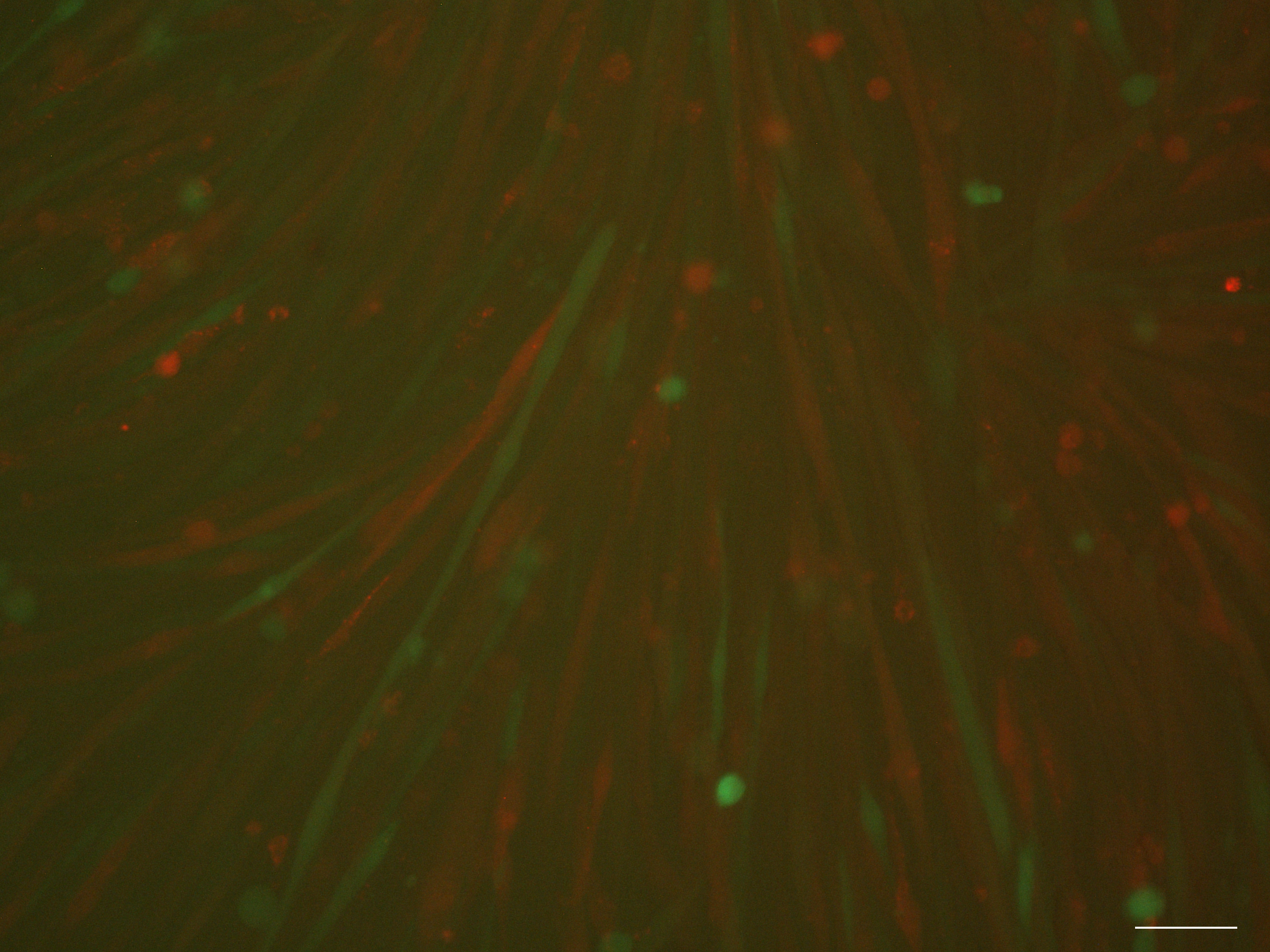

Supplement: Supplementary file 14 — Figure EV3 Source Data [file 44319_2024_197_MOESM14_ESM.zip › Figure EV3/EV3F/Cell mixing experiment- images/48 h GFP control-mCherry control-3.tif]

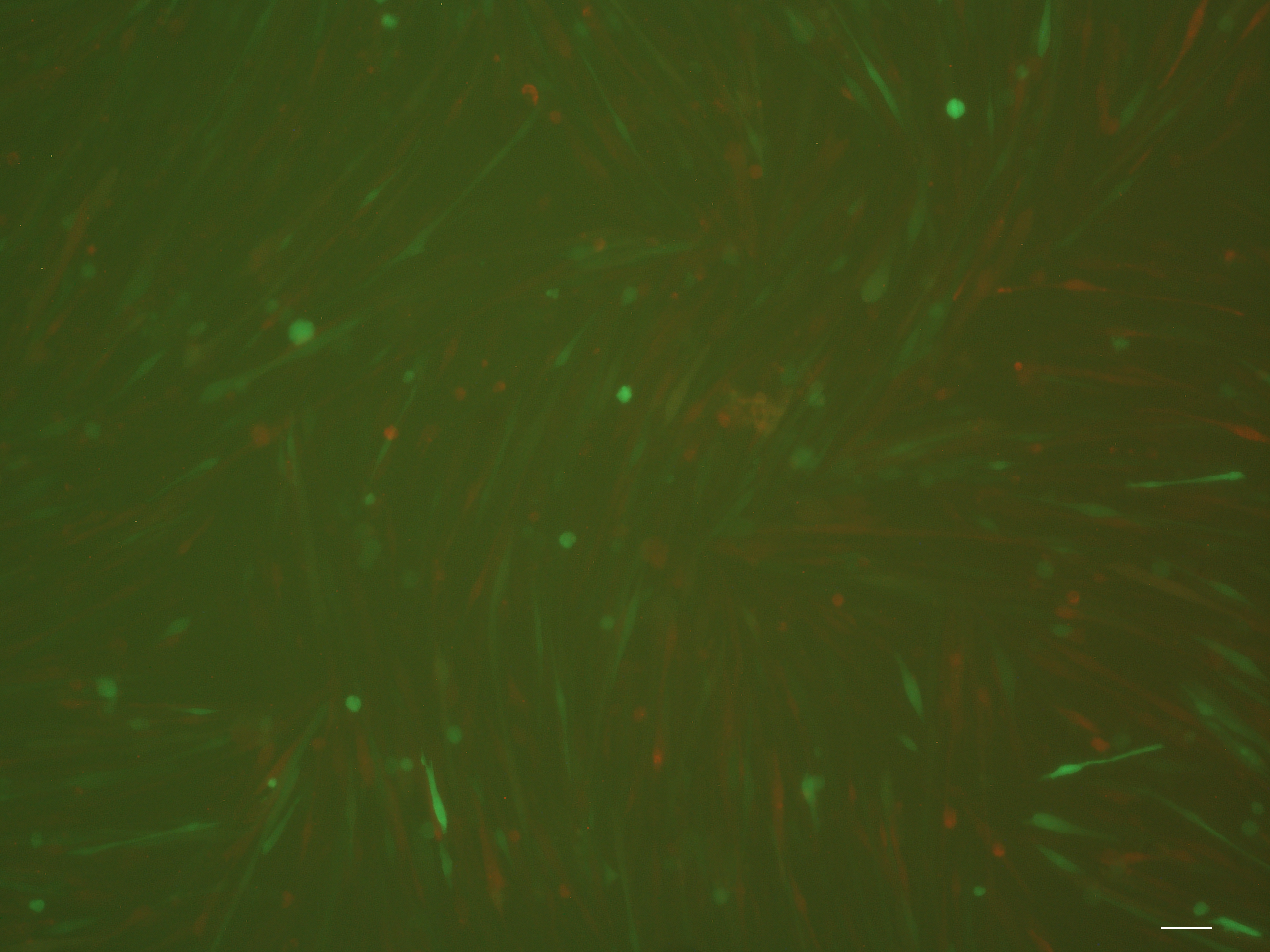

Supplement: Supplementary file 14 — Figure EV3 Source Data [file 44319_2024_197_MOESM14_ESM.zip › Figure EV3/EV3F/Cell mixing experiment- images/48 h GFP control-mCherry control-4.tif]

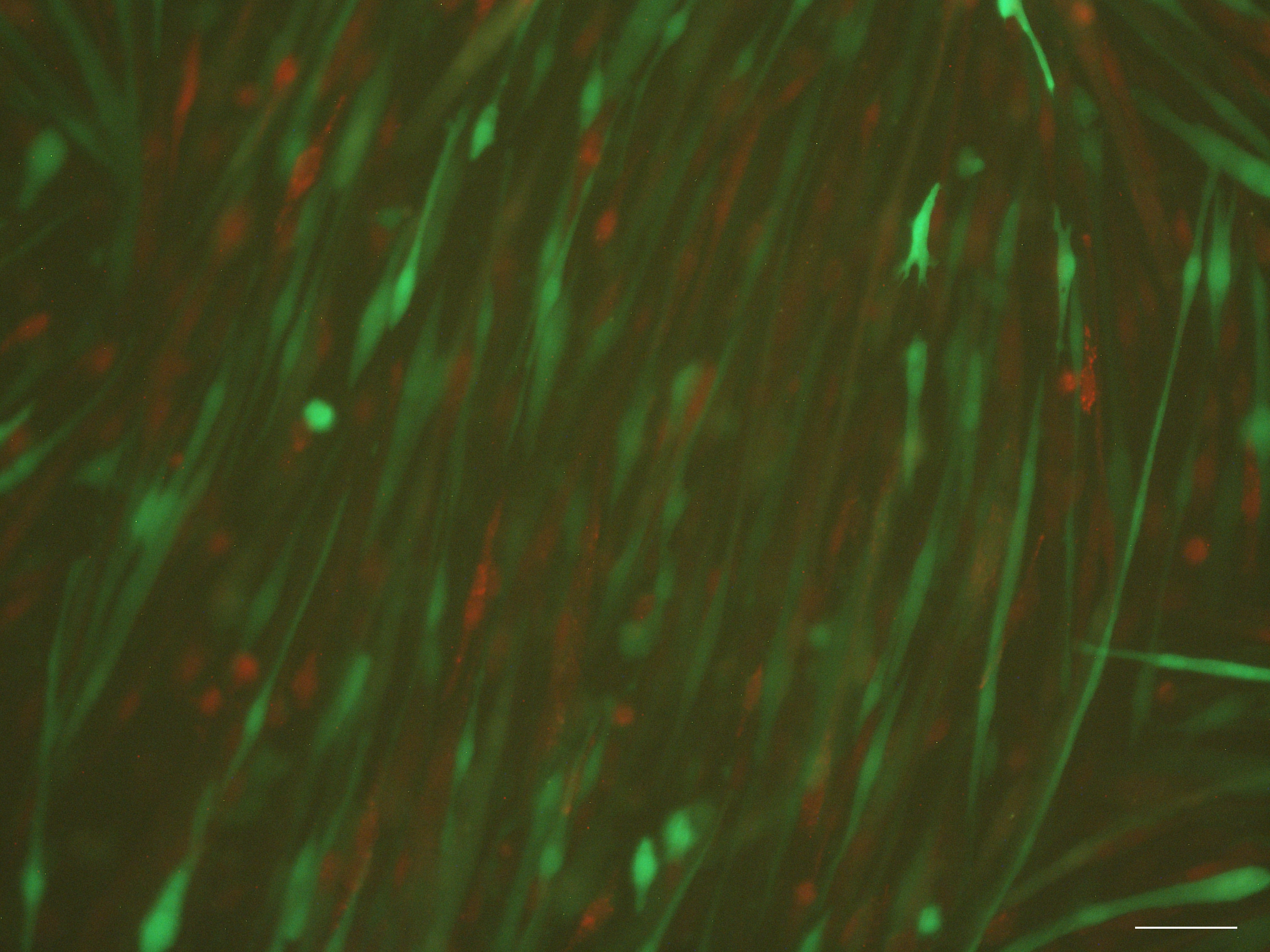

Supplement: Supplementary file 14 — Figure EV3 Source Data [file 44319_2024_197_MOESM14_ESM.zip › Figure EV3/EV3F/Cell mixing experiment- images/48 h GFP control-mCherry control-5.tif]

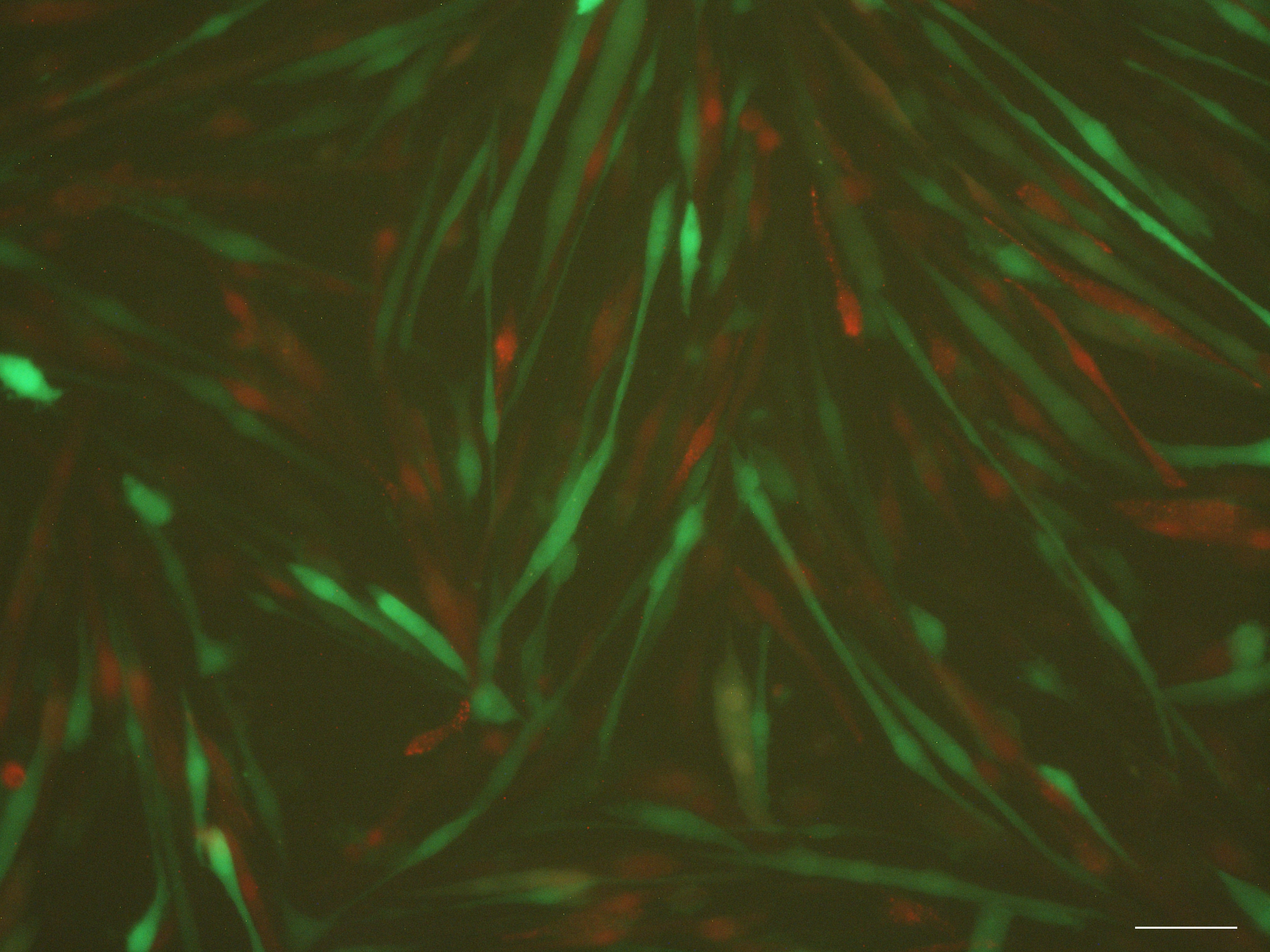

Supplement: Supplementary file 14 — Figure EV3 Source Data [file 44319_2024_197_MOESM14_ESM.zip › Figure EV3/EV3F/Cell mixing experiment- images/48 h GFP control-mCherry control-6.tif]

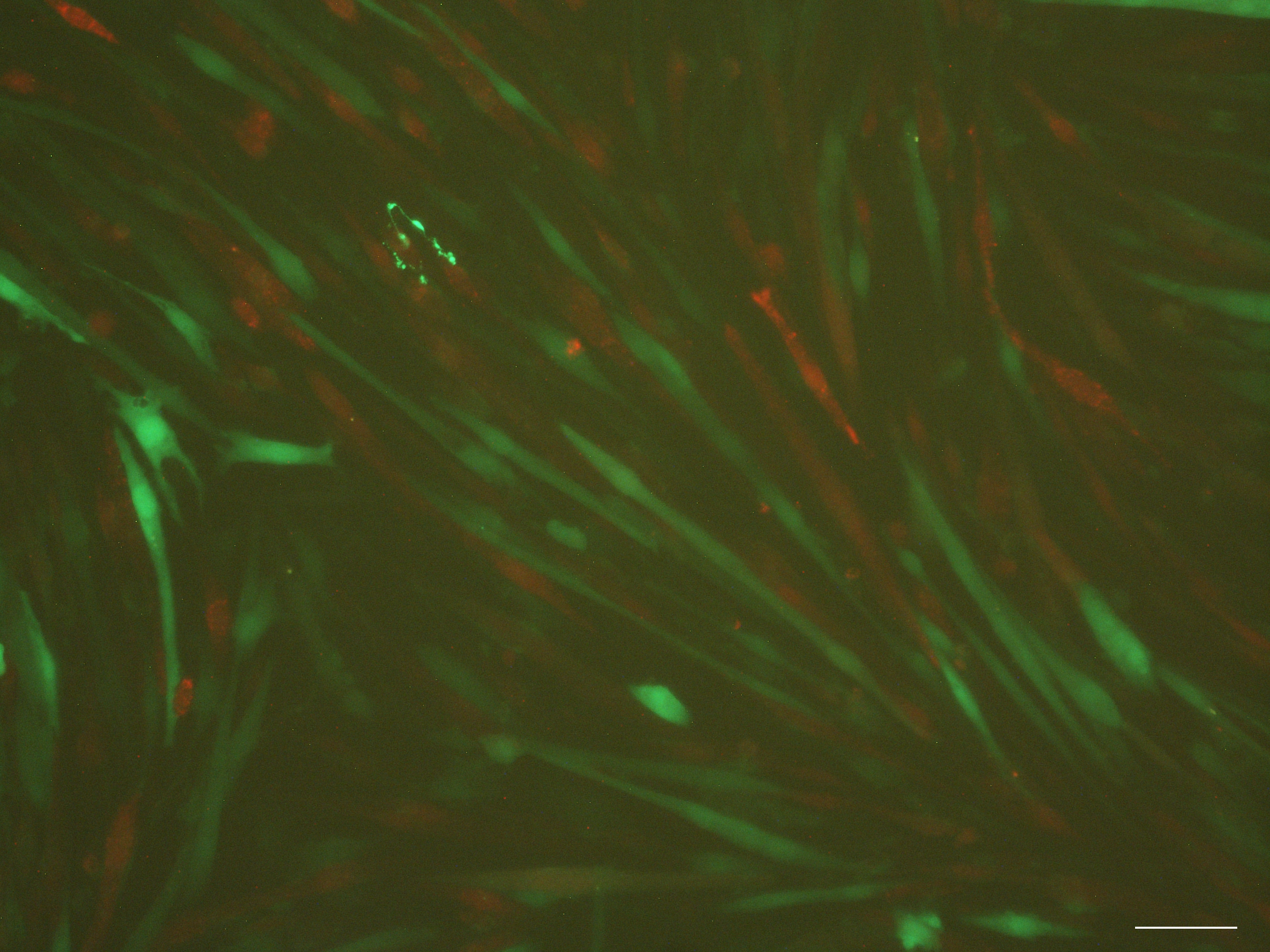

Supplement: Supplementary file 14 — Figure EV3 Source Data [file 44319_2024_197_MOESM14_ESM.zip › Figure EV3/EV3F/Cell mixing experiment- images/48 h GFP control-mCherry control-7.tif]

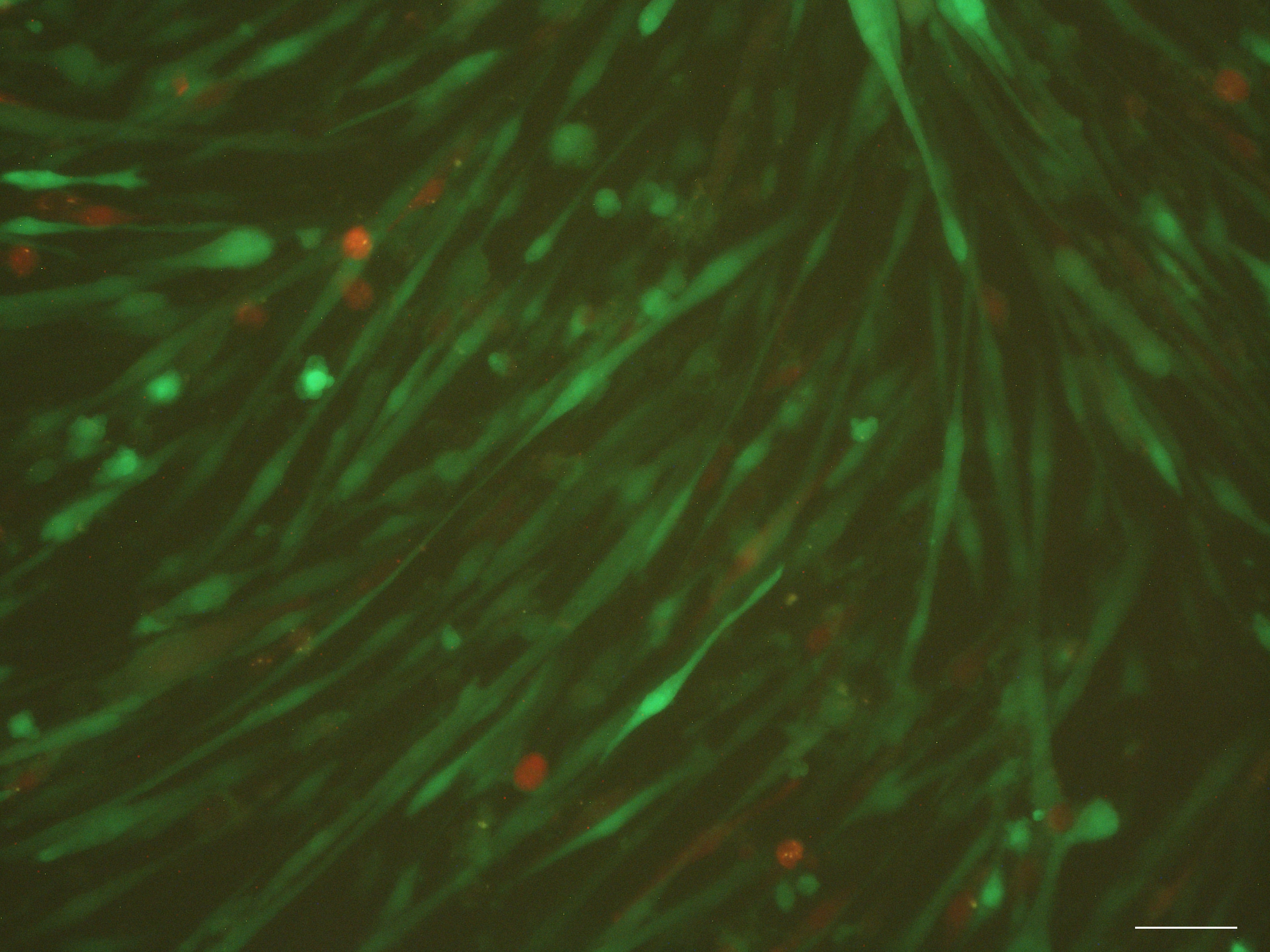

Supplement: Supplementary file 14 — Figure EV3 Source Data [file 44319_2024_197_MOESM14_ESM.zip › Figure EV3/EV3F/Cell mixing experiment- images/48 h GFP control-mCherry IRE1a KD Representative image.tif]

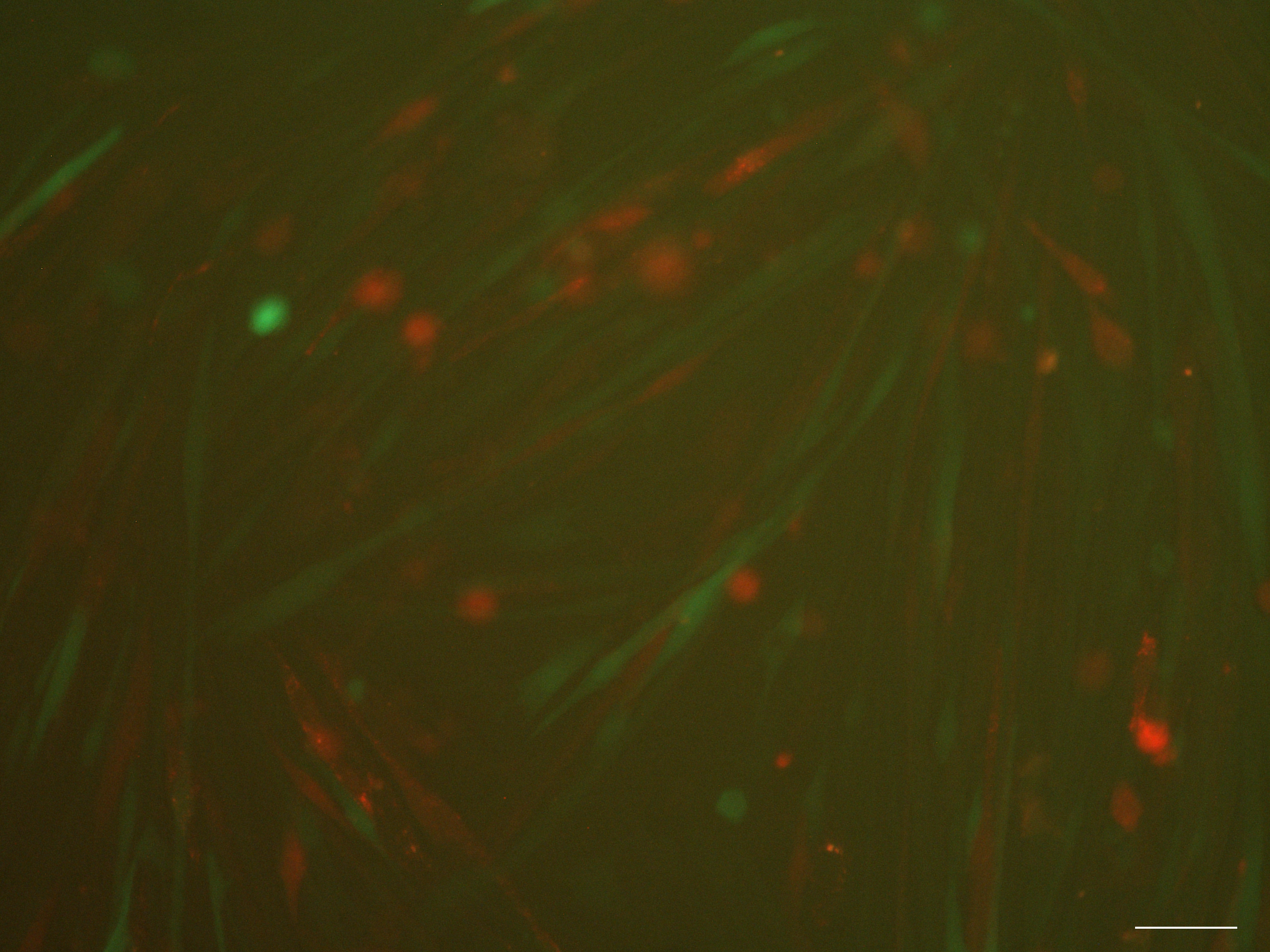

Supplement: Supplementary file 14 — Figure EV3 Source Data [file 44319_2024_197_MOESM14_ESM.zip › Figure EV3/EV3F/Cell mixing experiment- images/48 h GFP control-mCherry IRE1a KD-2.tif]

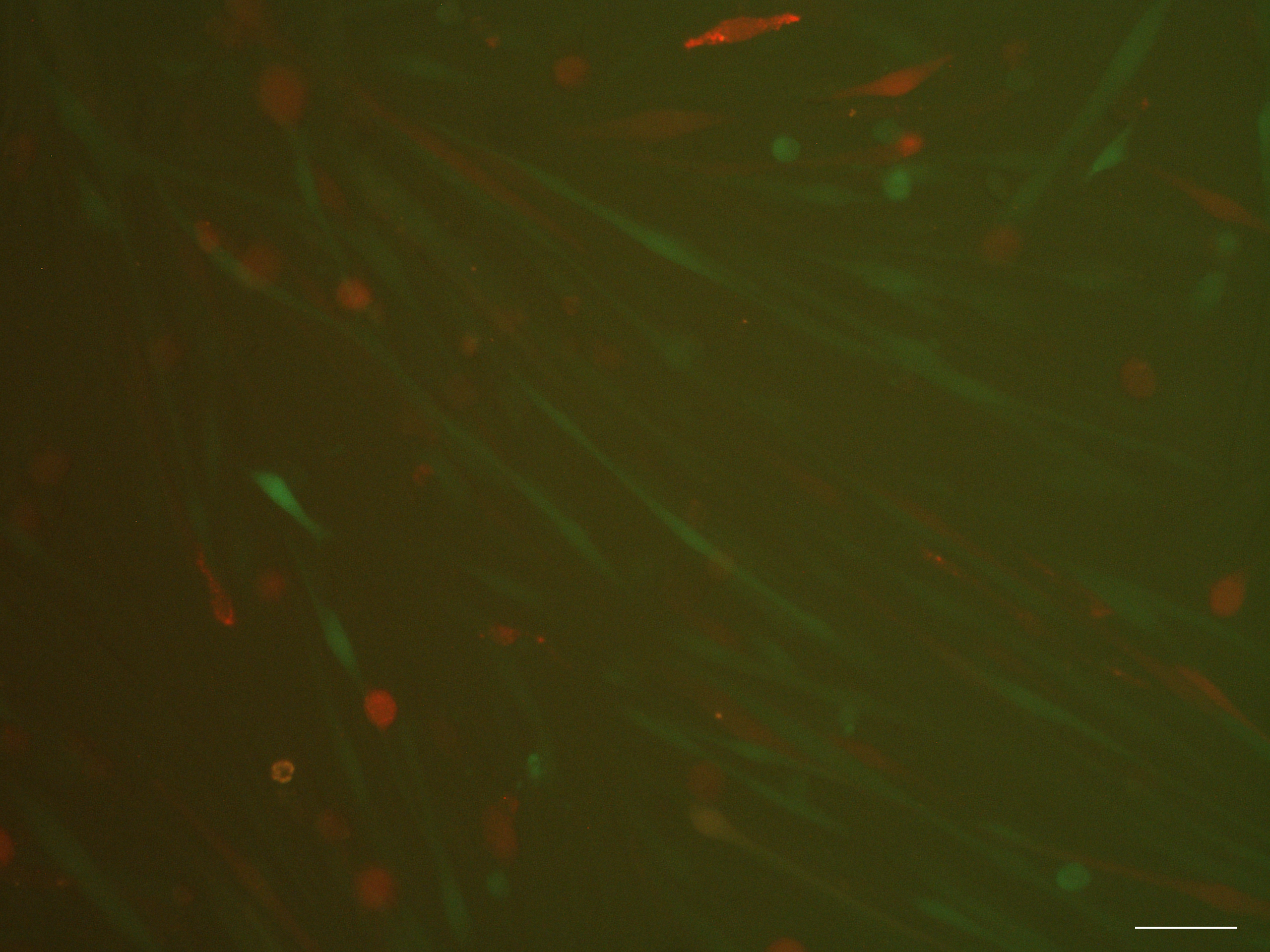

Supplement: Supplementary file 14 — Figure EV3 Source Data [file 44319_2024_197_MOESM14_ESM.zip › Figure EV3/EV3F/Cell mixing experiment- images/48 h GFP control-mCherry IRE1a KD-3.tif]

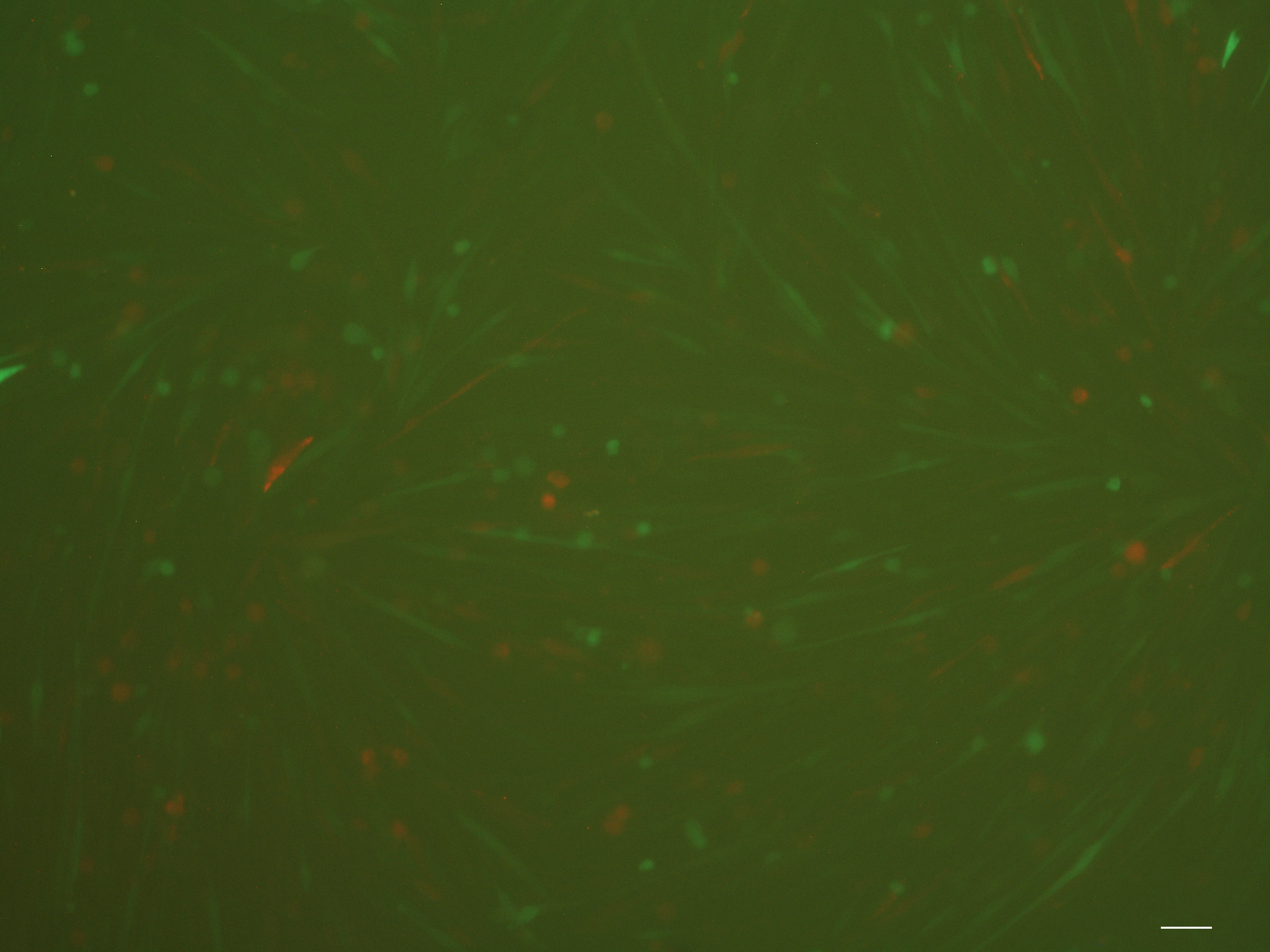

Supplement: Supplementary file 14 — Figure EV3 Source Data [file 44319_2024_197_MOESM14_ESM.zip › Figure EV3/EV3F/Cell mixing experiment- images/48 h GFP control-mCherry IRE1a KD-4.tif]

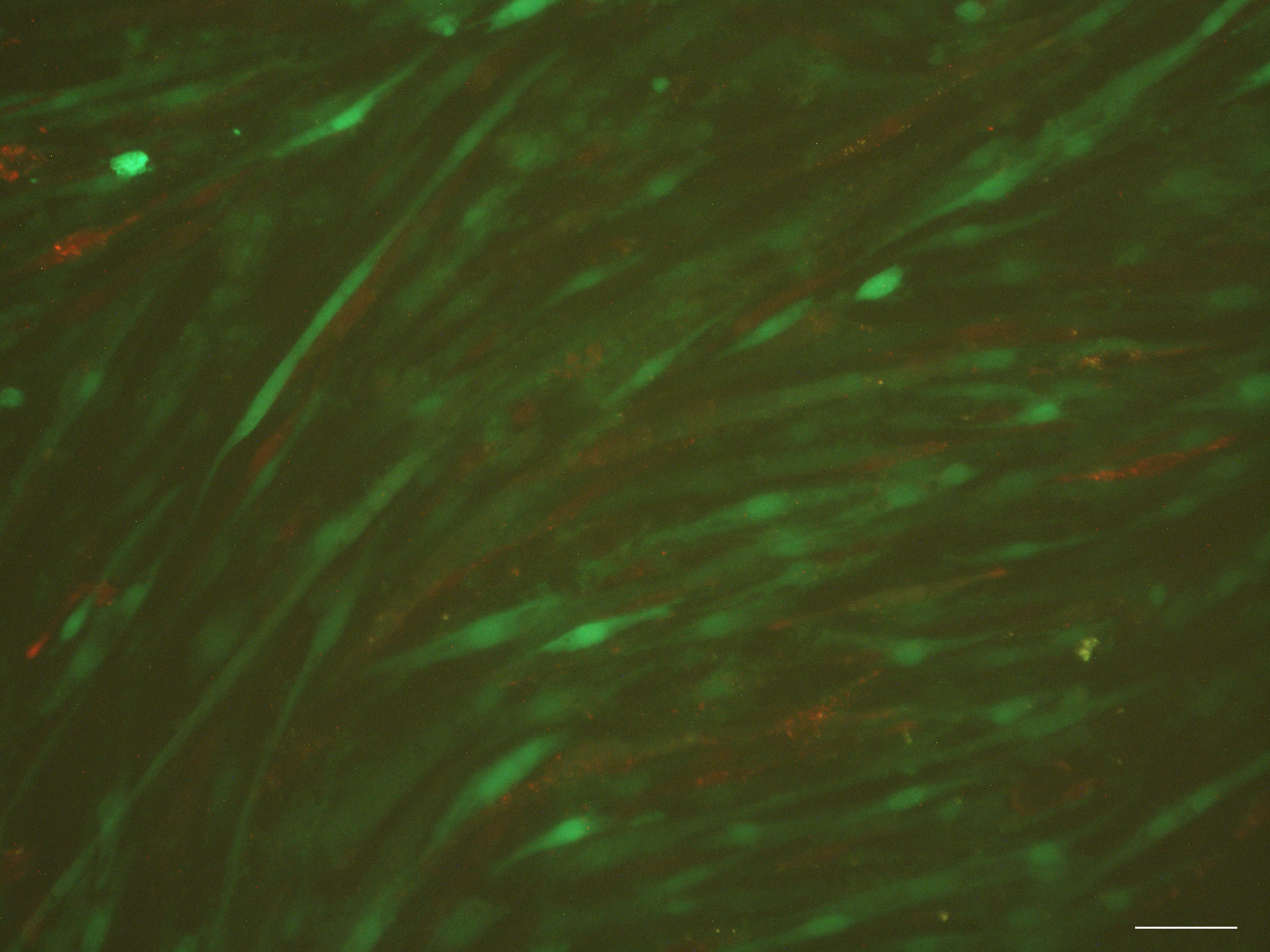

Supplement: Supplementary file 14 — Figure EV3 Source Data [file 44319_2024_197_MOESM14_ESM.zip › Figure EV3/EV3F/Cell mixing experiment- images/48 h GFP control-mCherry IRE1a KD-5.tif]

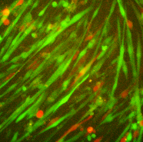

Supplement: Supplementary file 14 — Figure EV3 Source Data [file 44319_2024_197_MOESM14_ESM.zip › Figure EV3/EV3F/Cell mixing experiment- images/gfp + mcherry IRE1-KD 48h.tif]

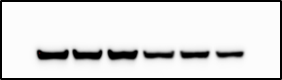

Supplement: Supplementary file 14 — Figure EV3 Source Data [file 44319_2024_197_MOESM14_ESM.zip › Figure EV3/EV3G/GAPDH - Western.tif]

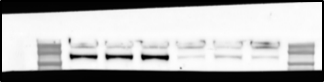

Supplement: Supplementary file 14 — Figure EV3 Source Data [file 44319_2024_197_MOESM14_ESM.zip › Figure EV3/EV3G/IRE1a - Western.tif]

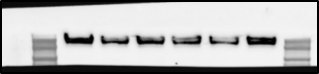

Supplement: Supplementary file 14 — Figure EV3 Source Data [file 44319_2024_197_MOESM14_ESM.zip › Figure EV3/EV3G/MyHC - Western.tif]

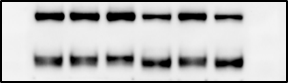

Supplement: Supplementary file 14 — Figure EV3 Source Data [file 44319_2024_197_MOESM14_ESM.zip › Figure EV3/EV3G/p-100-p52 - Western.tif]

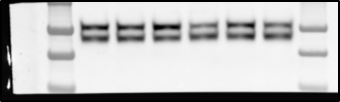

Supplement: Supplementary file 14 — Figure EV3 Source Data [file 44319_2024_197_MOESM14_ESM.zip › Figure EV3/EV3G/p-GSK-3B - Western.tif]

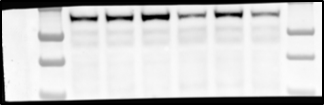

Supplement: Supplementary file 14 — Figure EV3 Source Data [file 44319_2024_197_MOESM14_ESM.zip › Figure EV3/EV3G/p-p65 - Western.tif]

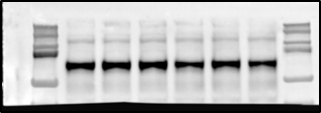

Supplement: Supplementary file 14 — Figure EV3 Source Data [file 44319_2024_197_MOESM14_ESM.zip › Figure EV3/EV3G/p65 - Western.tif]

## Slide 1
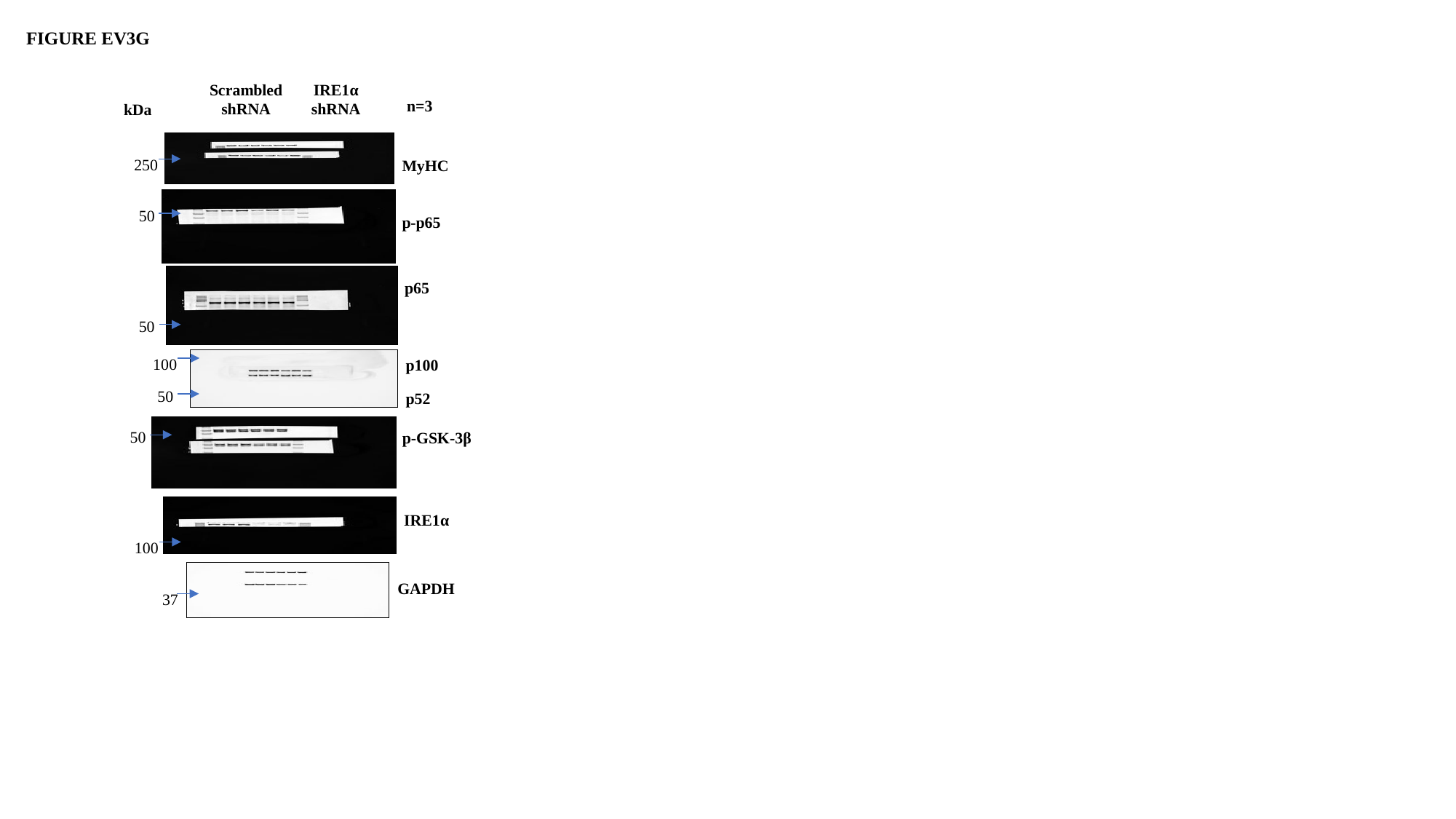

FIGURE EV3G
Scrambled
shRNA
IRE1α
shRNA
n=3
kDa
250
MyHC
50
p-p65
p65
50
100
p100
50
p52
50
p-GSK-3β
IRE1α
100
GAPDH
37

Supplement: Supplementary file 14 — Figure EV3 Source Data [file 44319_2024_197_MOESM14_ESM.zip › Figure EV3/EV3G/Western blot with annotation.pptx]

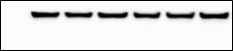

Supplement: Supplementary file 16 — Figure EV5 Source Data [file 44319_2024_197_MOESM16_ESM.zip › Figure EV5/EV5B/GAPDH- Western.tif]

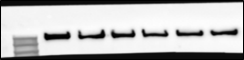

Supplement: Supplementary file 16 — Figure EV5 Source Data [file 44319_2024_197_MOESM16_ESM.zip › Figure EV5/EV5B/MyHC- Western.tif]

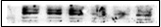

Supplement: Supplementary file 16 — Figure EV5 Source Data [file 44319_2024_197_MOESM16_ESM.zip › Figure EV5/EV5B/sXBP1- Western.tif]

## Slide 1
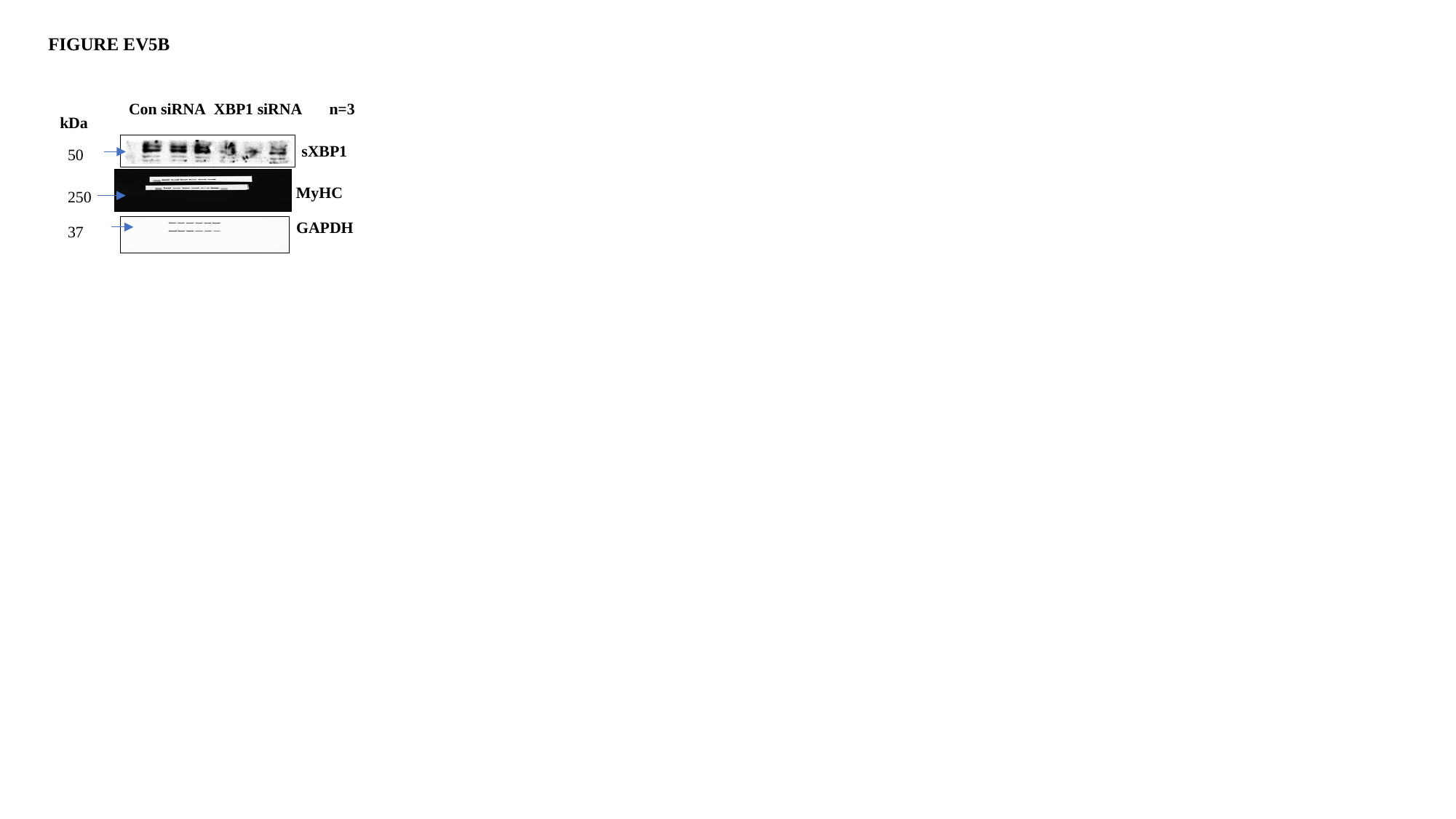

FIGURE EV5B
Con siRNA
XBP1 siRNA n=3
kDa
sXBP1
50
MyHC
250
GAPDH
37

Supplement: Supplementary file 16 — Figure EV5 Source Data [file 44319_2024_197_MOESM16_ESM.zip › Figure EV5/EV5B/Western blot with annotation.pptx]

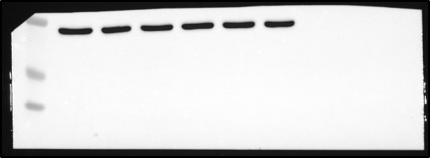

Supplement: Supplementary file 16 — Figure EV5 Source Data [file 44319_2024_197_MOESM16_ESM.zip › Figure EV5/EV5C/GAPDH- Western.tif]

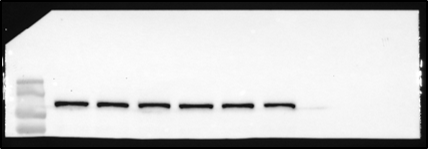

Supplement: Supplementary file 16 — Figure EV5 Source Data [file 44319_2024_197_MOESM16_ESM.zip › Figure EV5/EV5C/IRE1a- Western.tif]

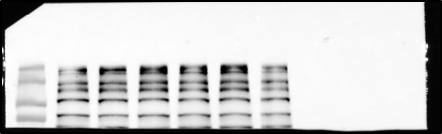

Supplement: Supplementary file 16 — Figure EV5 Source Data [file 44319_2024_197_MOESM16_ESM.zip › Figure EV5/EV5C/pIRE1a- Western.tif]

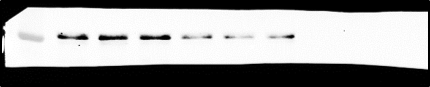

Supplement: Supplementary file 16 — Figure EV5 Source Data [file 44319_2024_197_MOESM16_ESM.zip › Figure EV5/EV5C/sXBP1- Western.tif]

## Slide 1
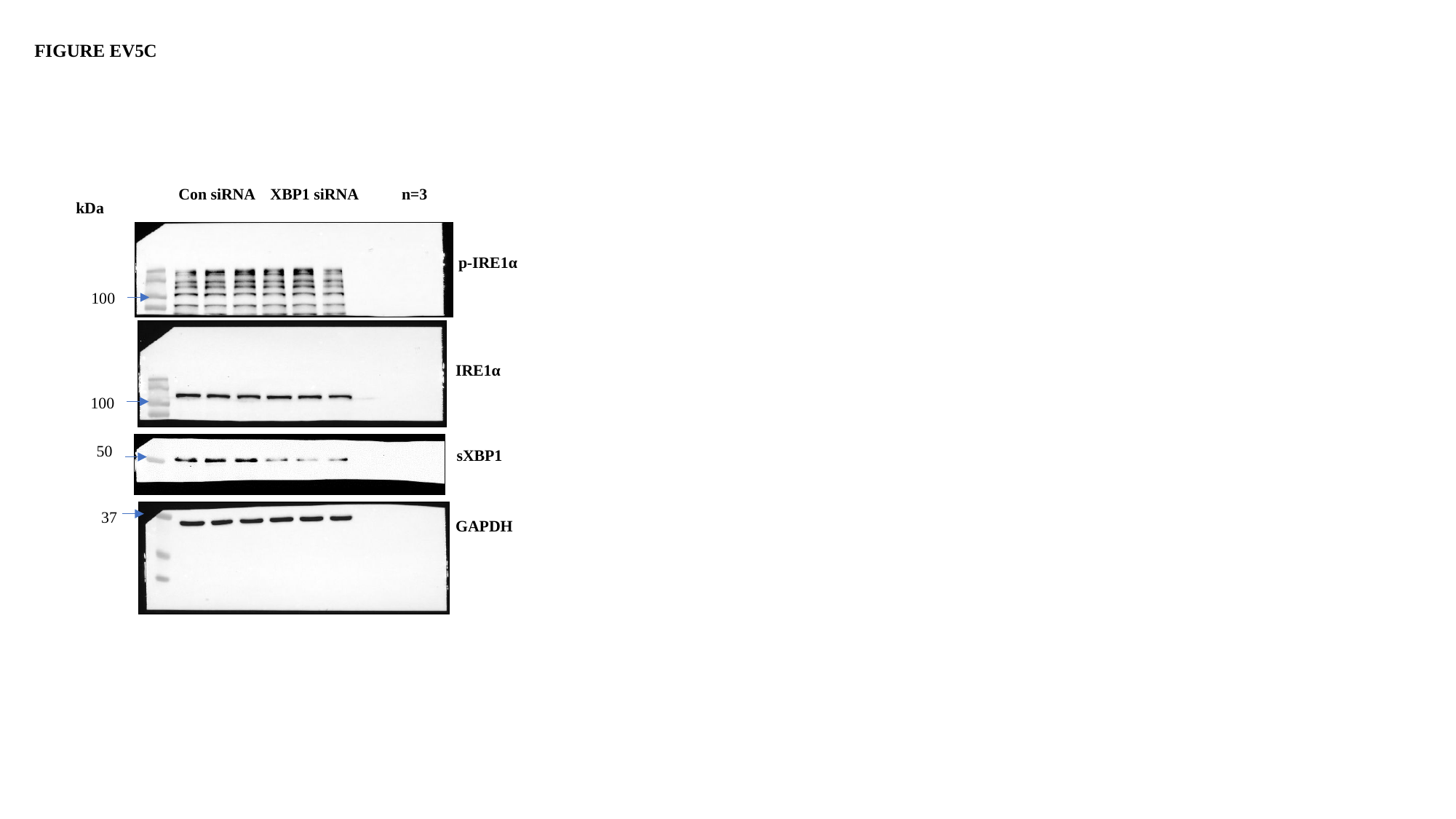

FIGURE EV5C
Con siRNA
XBP1 siRNA n=3
kDa
p-IRE1α
100
IRE1α
100
50
sXBP1
37
GAPDH

Supplement: Supplementary file 16 — Figure EV5 Source Data [file 44319_2024_197_MOESM16_ESM.zip › Figure EV5/EV5C/Western blot with annotation.pptx]

## Slide 1
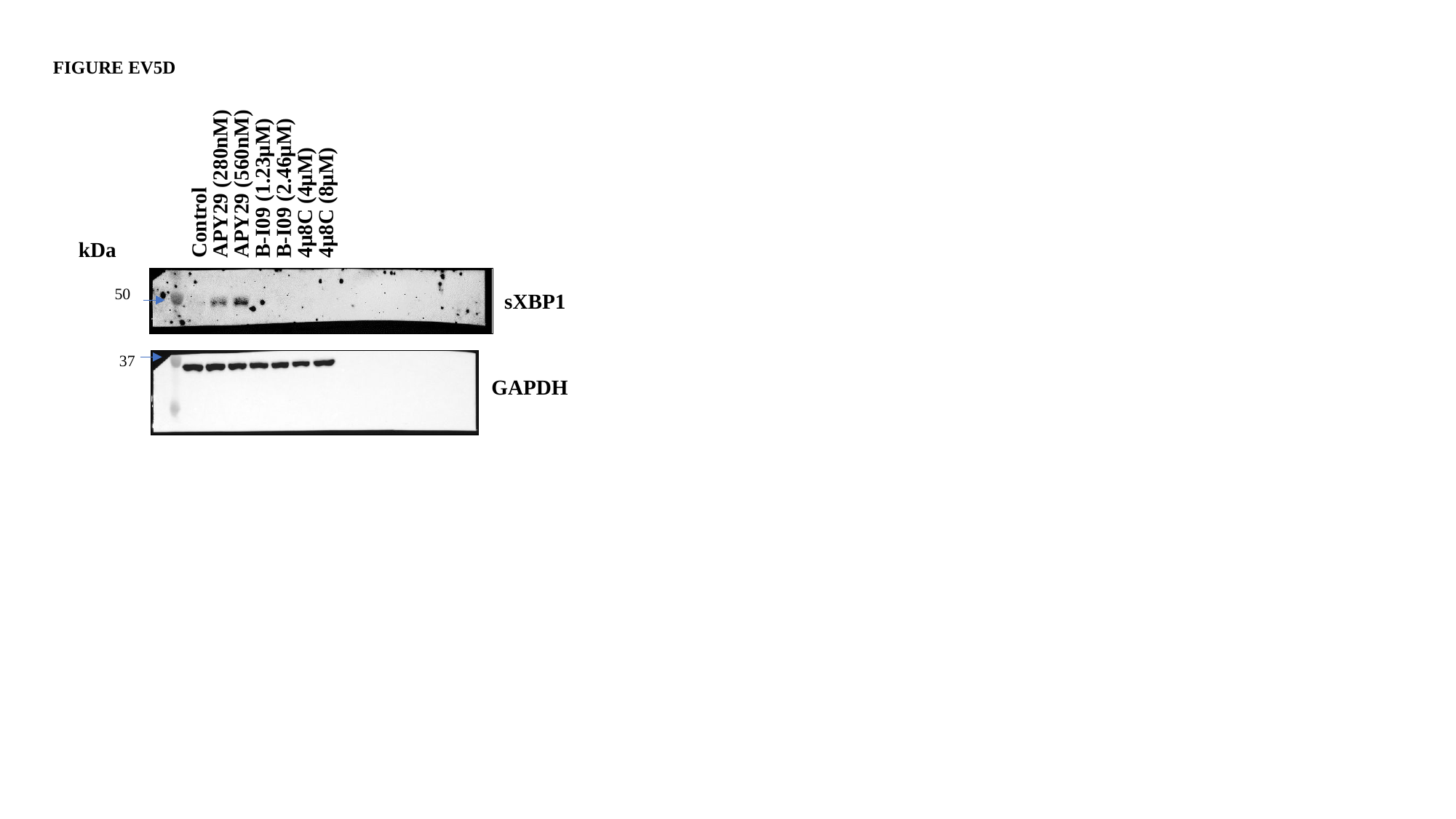

FIGURE EV5D
APY29 (280nM)
APY29 (560nM)
B-I09 (1.23μM)
B-I09 (2.46μM)
4μ8C (4μM)
4μ8C (8μM)
Control
kDa
50
sXBP1
37
GAPDH

Supplement: Supplementary file 16 — Figure EV5 Source Data [file 44319_2024_197_MOESM16_ESM.zip › Figure EV5/EV5D-G/EV5D/Western blot with annotation.pptx]

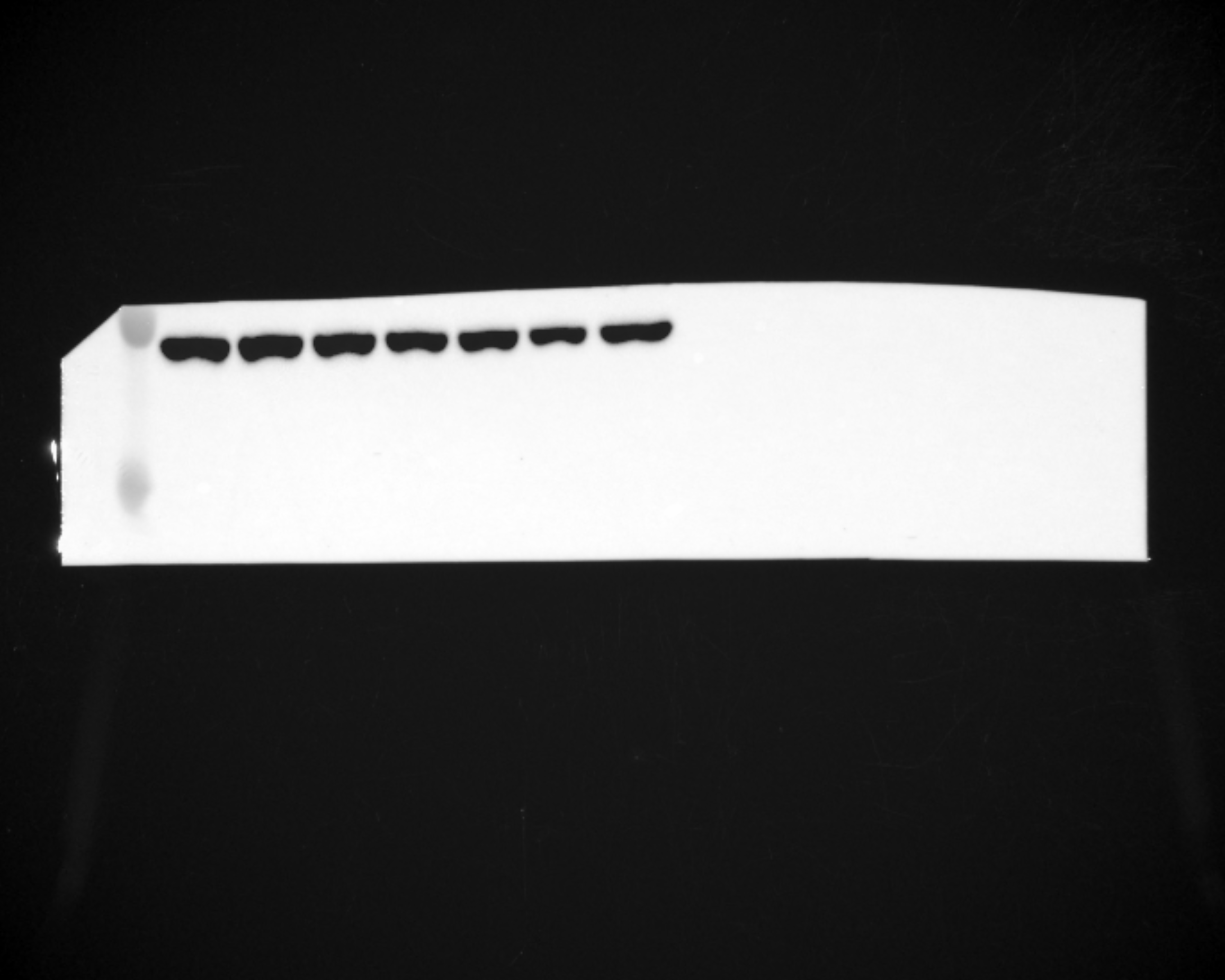

Supplement: Supplementary file 16 — Figure EV5 Source Data [file 44319_2024_197_MOESM16_ESM.zip › Figure EV5/EV5D-G/EV5D/Western-GAPDH.tif]

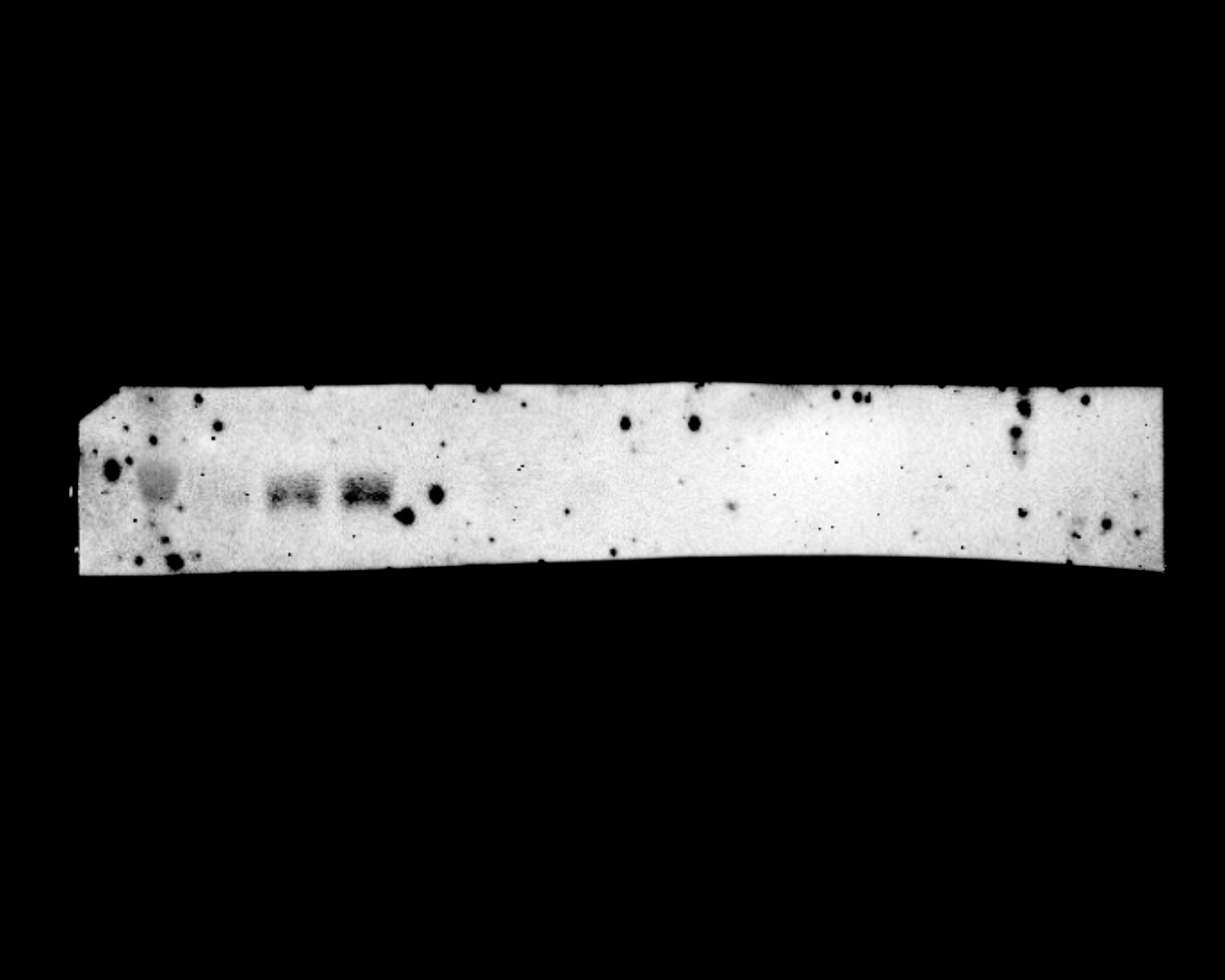

Supplement: Supplementary file 16 — Figure EV5 Source Data [file 44319_2024_197_MOESM16_ESM.zip › Figure EV5/EV5D-G/EV5D/Western-sXBP1.tif]

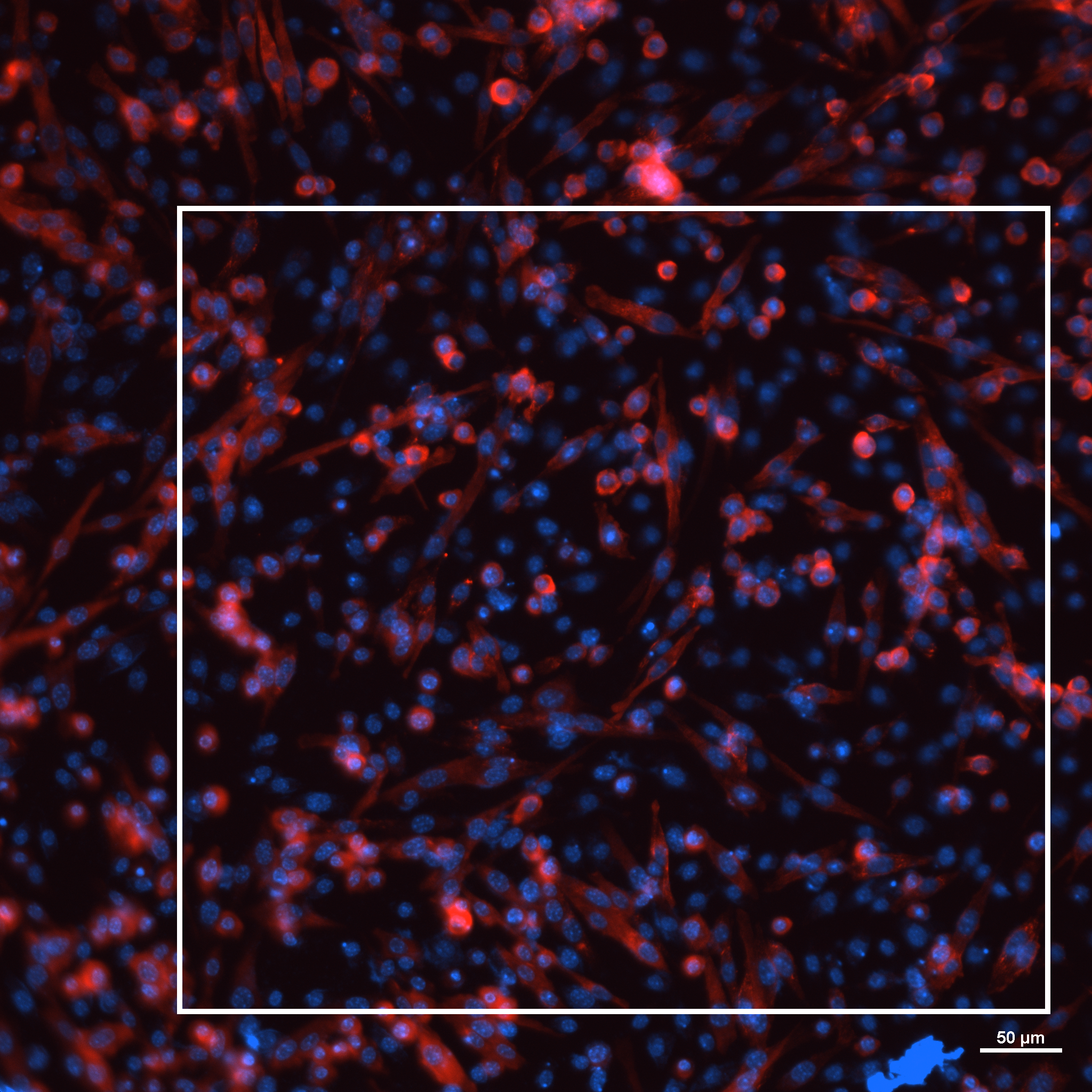

Supplement: Supplementary file 16 — Figure EV5 Source Data [file 44319_2024_197_MOESM16_ESM.zip › Figure EV5/EV5D-G/EV5E/IRE1 inhibitors-MyHC staining images/4u8C Representative image with box.tiff]

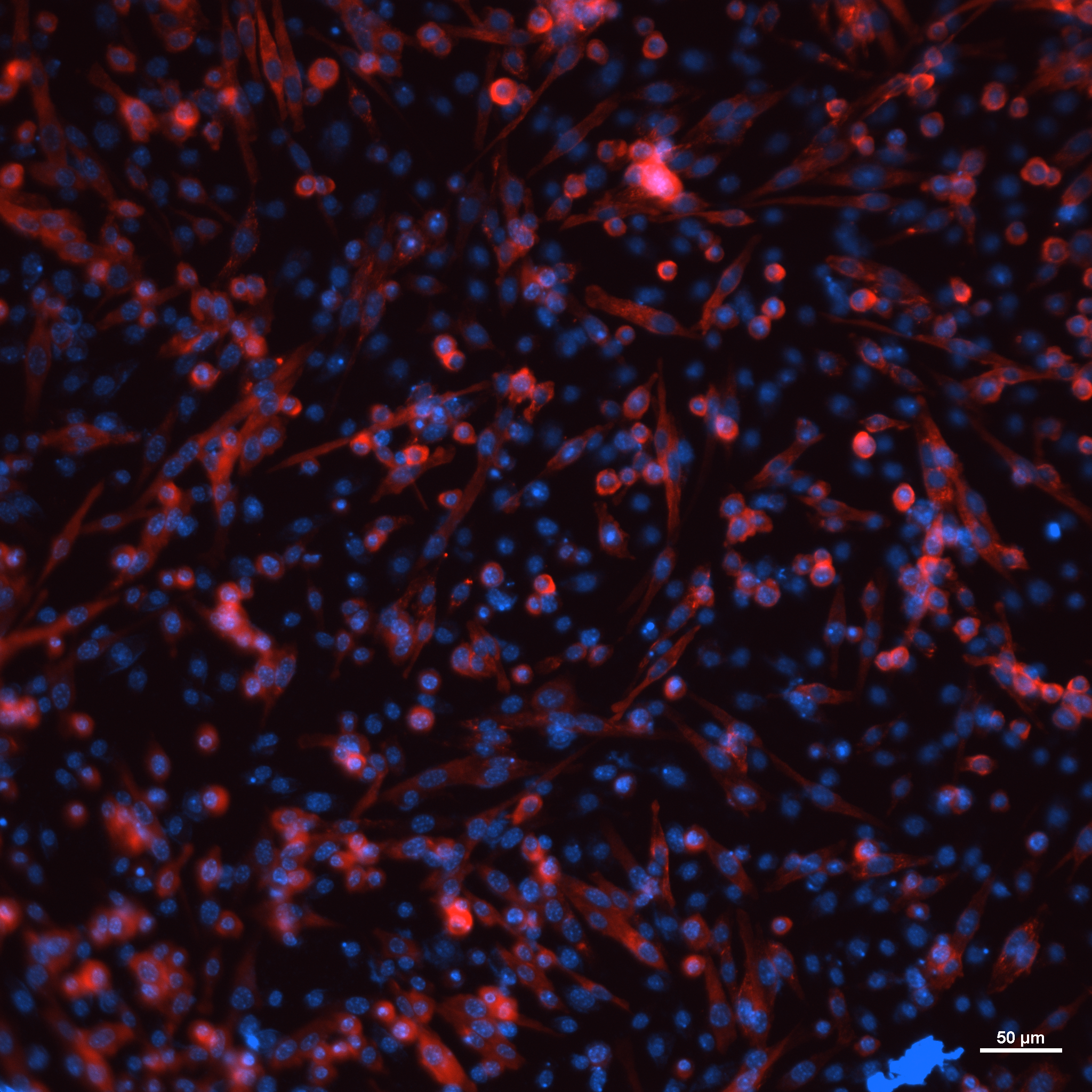

Supplement: Supplementary file 16 — Figure EV5 Source Data [file 44319_2024_197_MOESM16_ESM.zip › Figure EV5/EV5D-G/EV5E/IRE1 inhibitors-MyHC staining images/4u8C Representative image.tif]

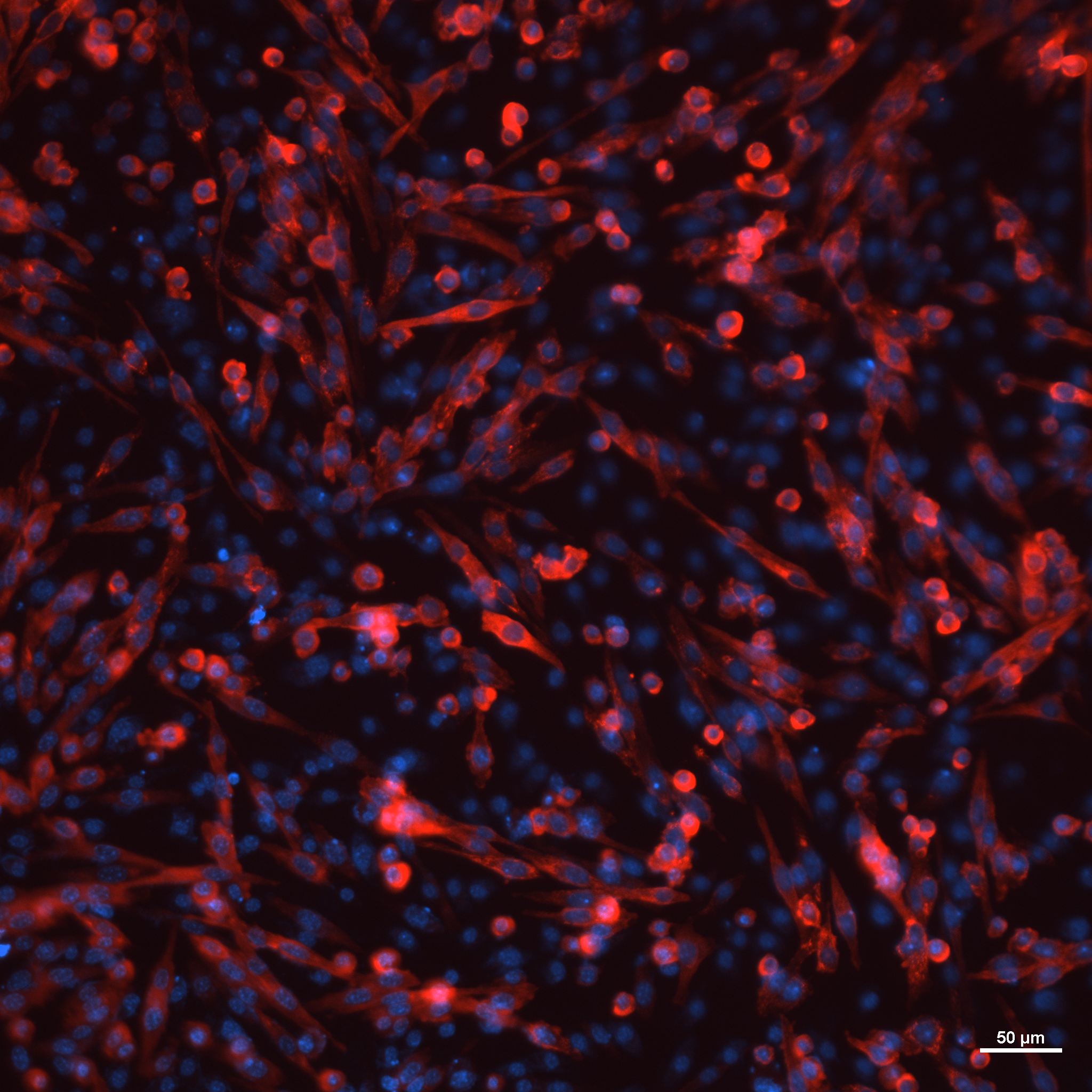

Supplement: Supplementary file 16 — Figure EV5 Source Data [file 44319_2024_197_MOESM16_ESM.zip › Figure EV5/EV5D-G/EV5E/IRE1 inhibitors-MyHC staining images/4u8c-2.tif]

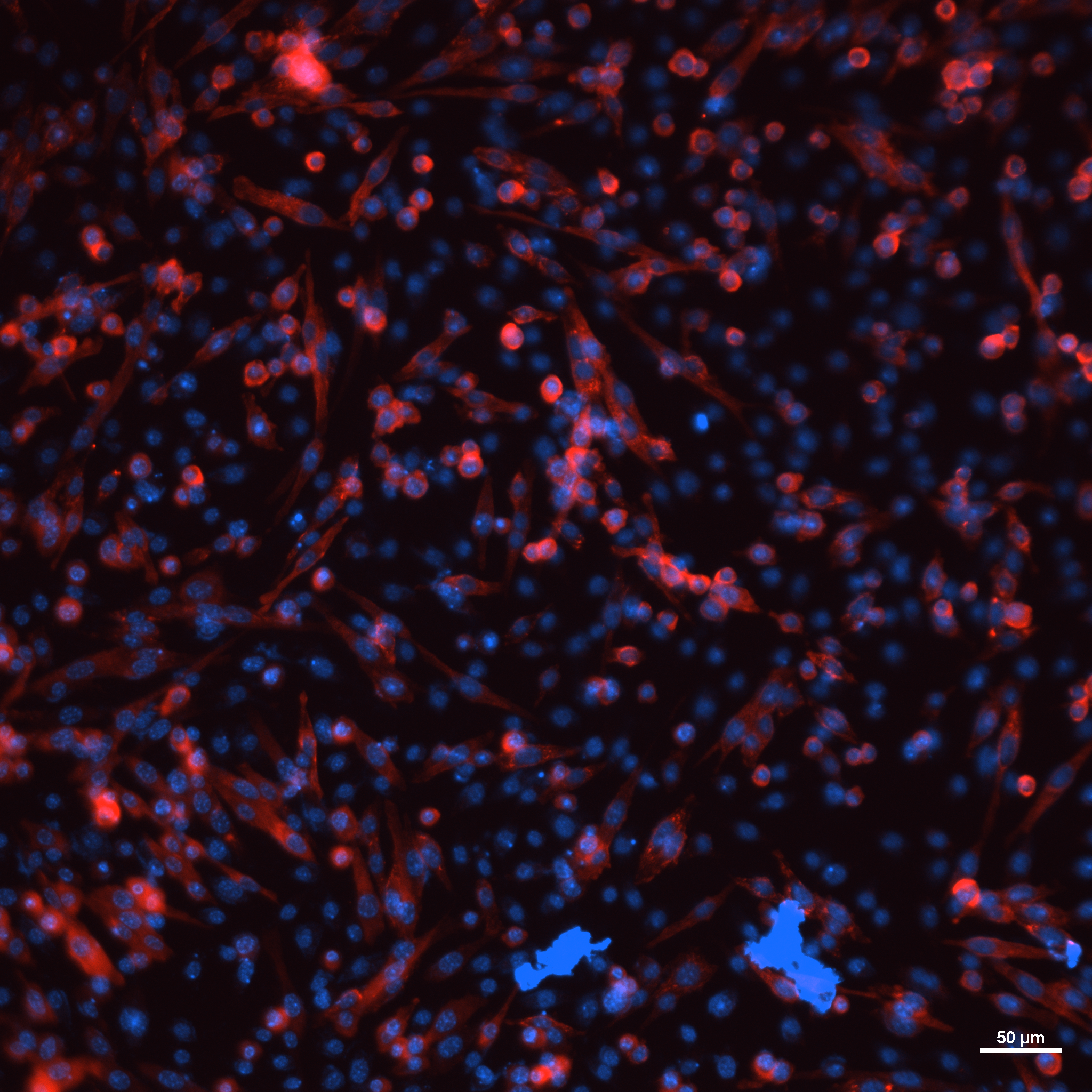

Supplement: Supplementary file 16 — Figure EV5 Source Data [file 44319_2024_197_MOESM16_ESM.zip › Figure EV5/EV5D-G/EV5E/IRE1 inhibitors-MyHC staining images/4u8c-3.tif]

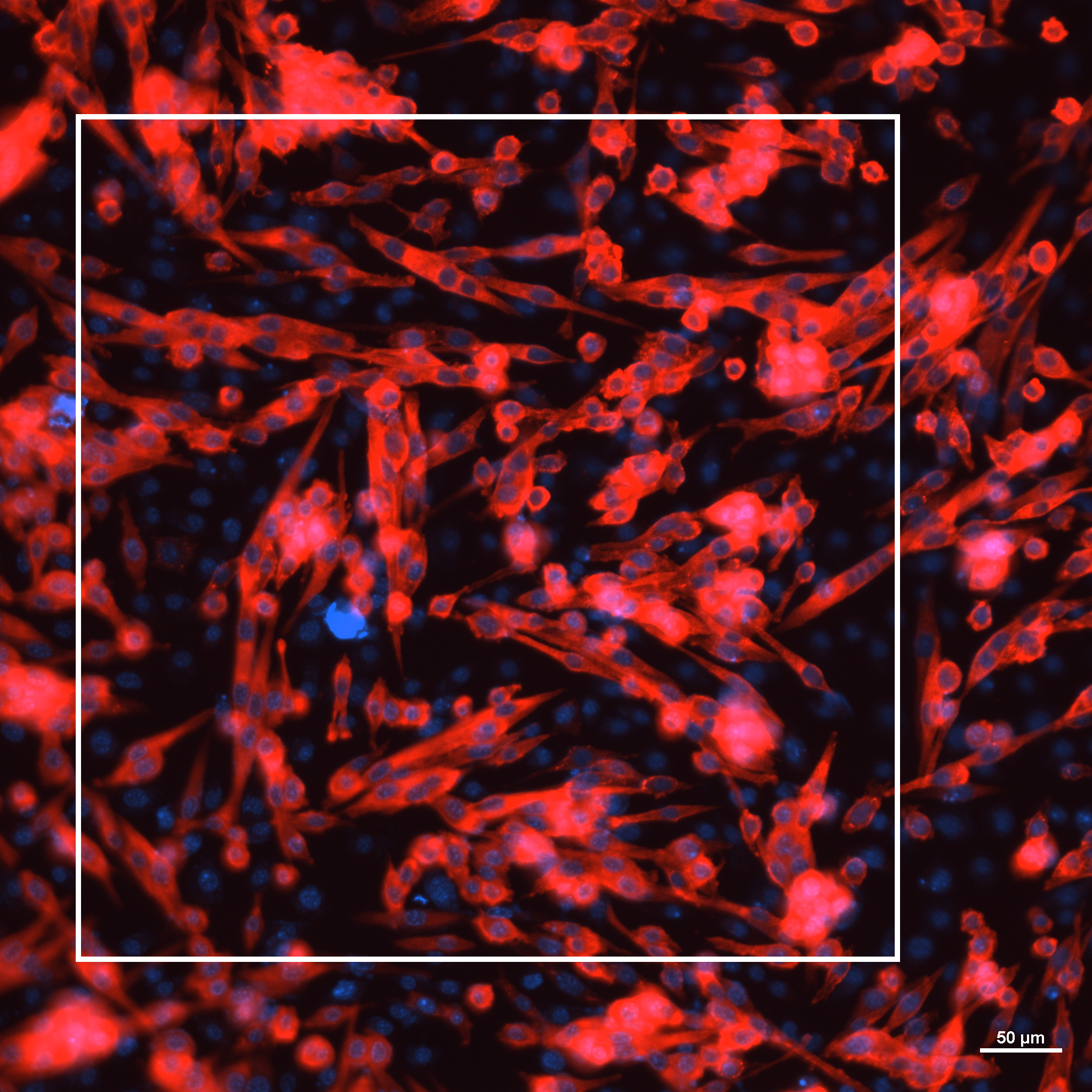

Supplement: Supplementary file 16 — Figure EV5 Source Data [file 44319_2024_197_MOESM16_ESM.zip › Figure EV5/EV5D-G/EV5E/IRE1 inhibitors-MyHC staining images/APY29 Representative image with box.tiff]

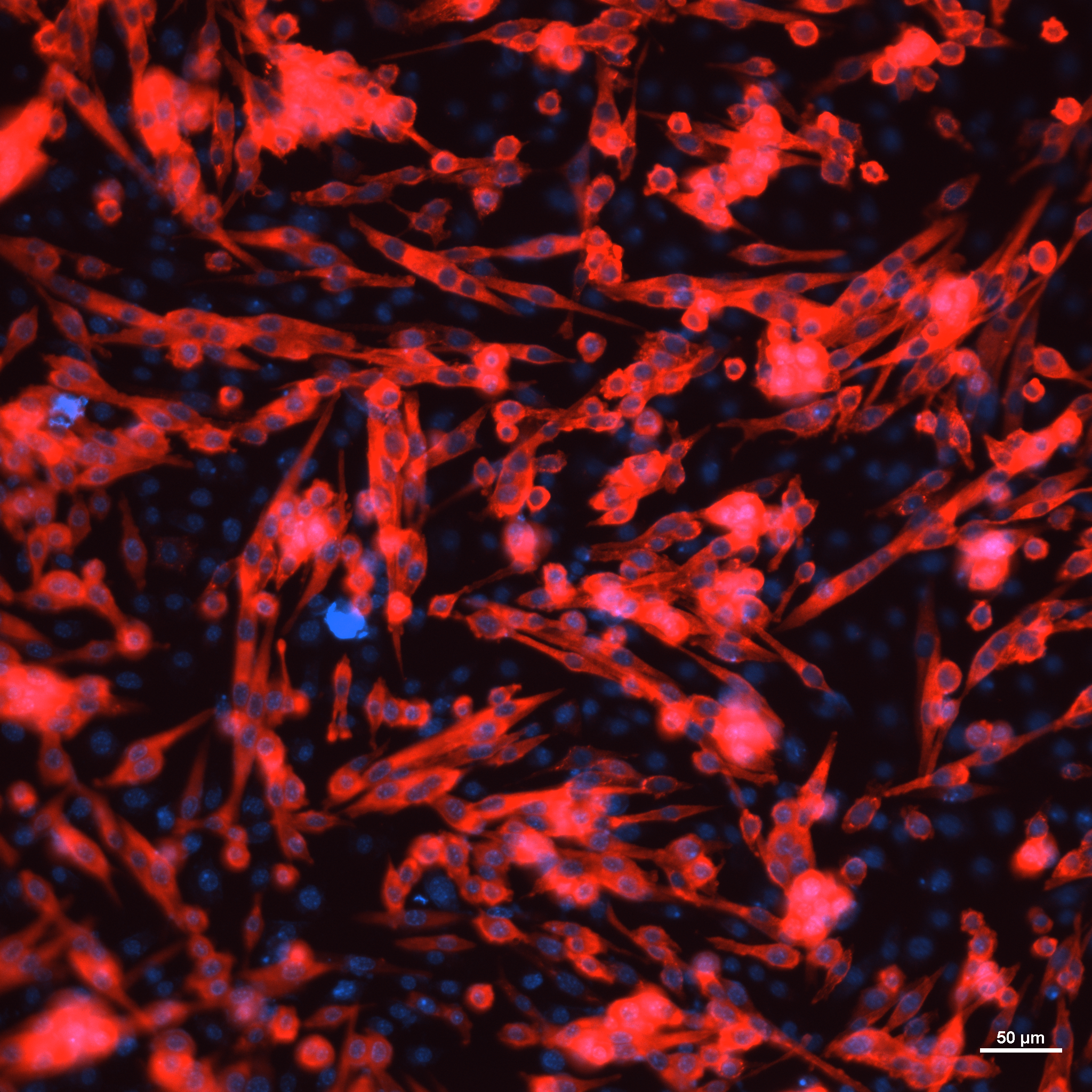

Supplement: Supplementary file 16 — Figure EV5 Source Data [file 44319_2024_197_MOESM16_ESM.zip › Figure EV5/EV5D-G/EV5E/IRE1 inhibitors-MyHC staining images/APY29 Representative image.tif]

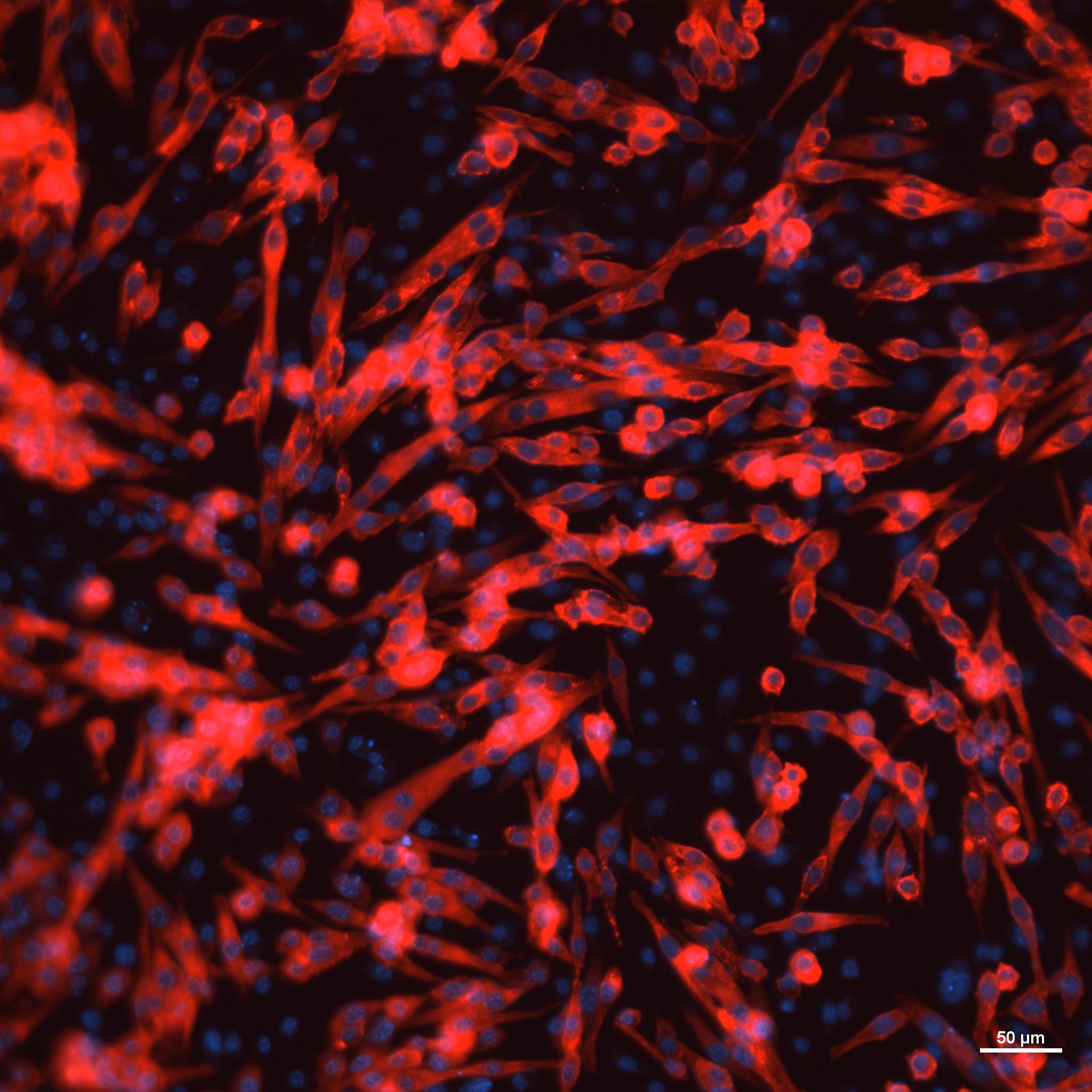

Supplement: Supplementary file 16 — Figure EV5 Source Data [file 44319_2024_197_MOESM16_ESM.zip › Figure EV5/EV5D-G/EV5E/IRE1 inhibitors-MyHC staining images/APY29-2.tif]

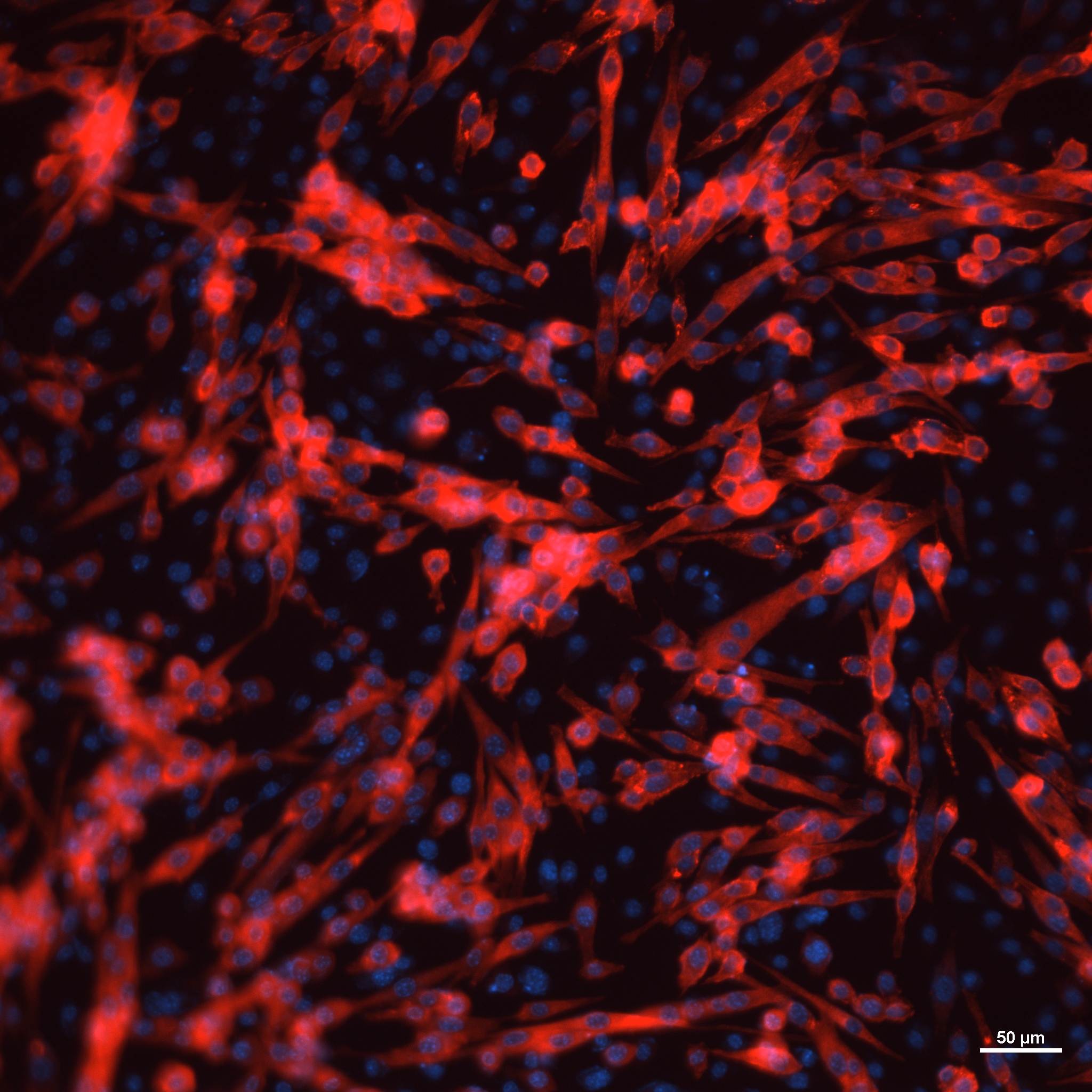

Supplement: Supplementary file 16 — Figure EV5 Source Data [file 44319_2024_197_MOESM16_ESM.zip › Figure EV5/EV5D-G/EV5E/IRE1 inhibitors-MyHC staining images/APY29-3.tif]
