# Supplementary material for: The IRE1α/XBP1 signaling axis drives myoblast fusion in adult skeletal muscle
Source: EMBO Rep. 2024 Jul 9;25(8):3627–50. doi: 10.1038/s44319-024-00197-4 (PMC11316051; doi:10.1038/s44319-024-00197-4)
Supplement: Supplementary file 16 — Figure EV5 Source Data [file 44319_2024_197_MOESM16_ESM.zip › Figure EV5/EV5I/Western blot with annotation.pptx]

## Slide 1
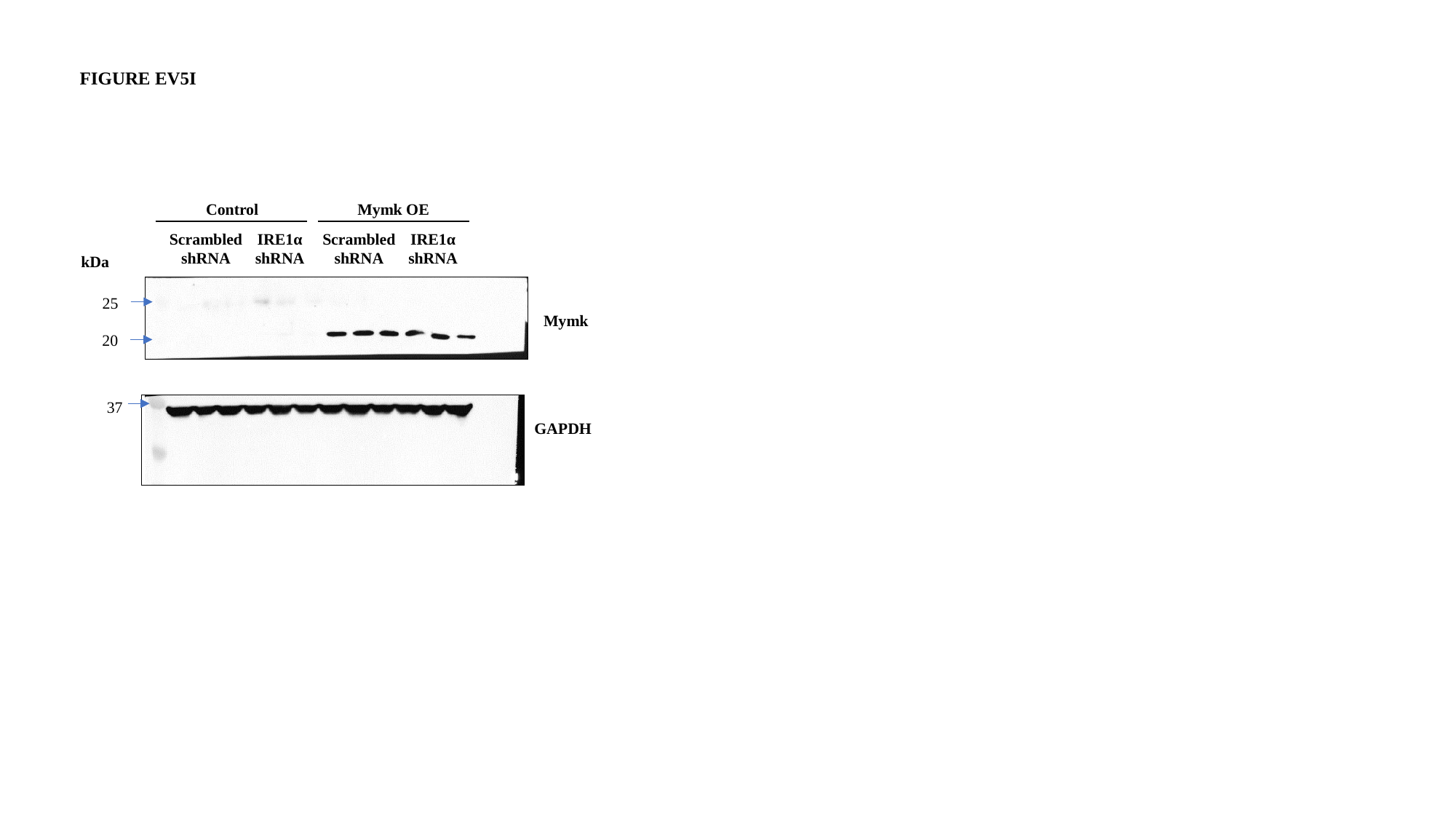

FIGURE EV5I
Control
Mymk OE
Scrambled
shRNA
IRE1α
shRNA
Scrambled
shRNA
IRE1α
shRNA
kDa
25
Mymk
20
37
GAPDH
