# Supplementary figures and images for: The IRE1α/XBP1 signaling axis drives myoblast fusion in adult skeletal muscle (part 4 of 4)
Source: EMBO Rep. 2024 Jul 9;25(8):3627–50. doi: 10.1038/s44319-024-00197-4 (PMC11316051; doi:10.1038/s44319-024-00197-4)

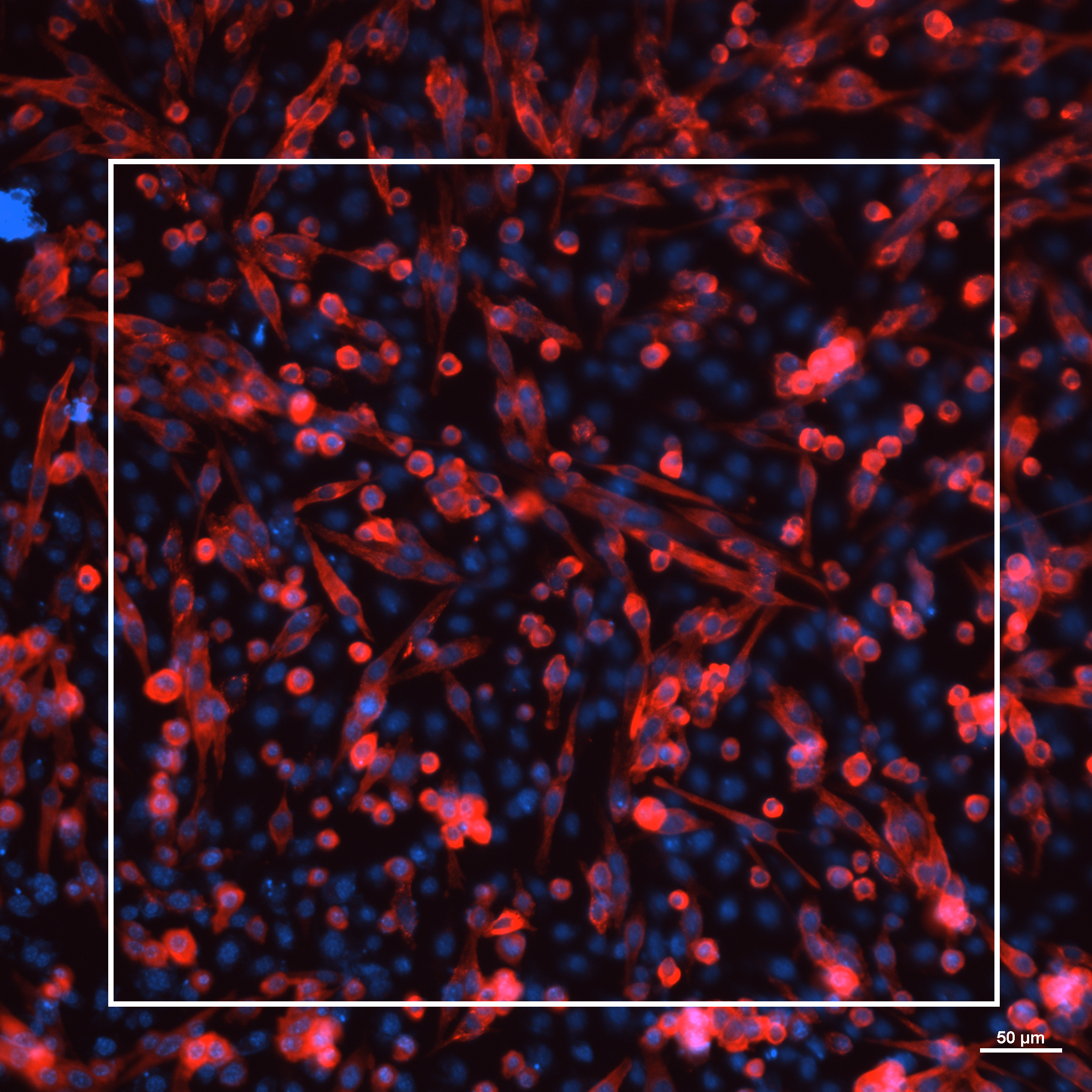

Supplement: Supplementary file 16 — Figure EV5 Source Data [file 44319_2024_197_MOESM16_ESM.zip › Figure EV5/EV5D-G/EV5E/IRE1 inhibitors-MyHC staining images/B-I09 Representative image with box.tiff]

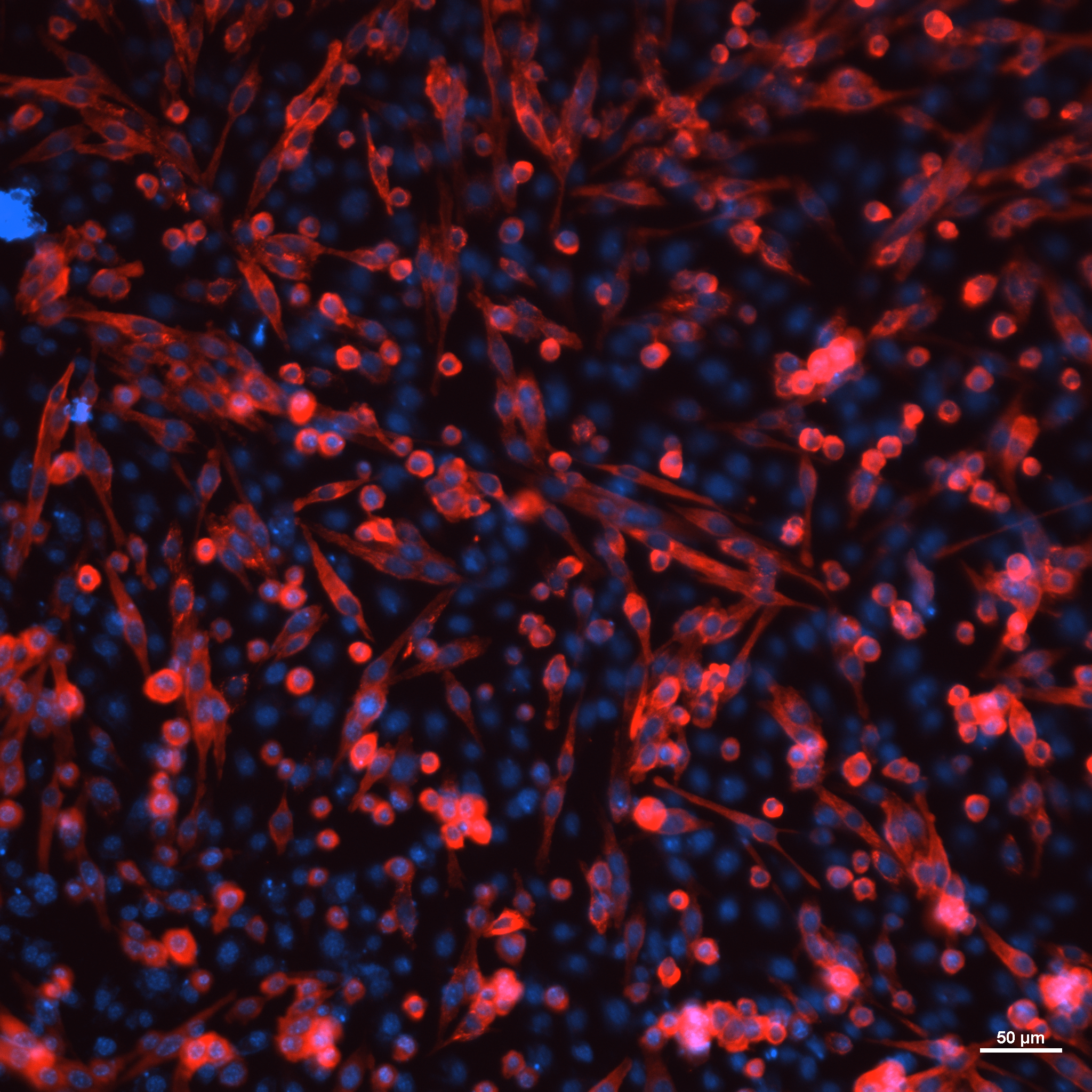

Supplement: Supplementary file 16 — Figure EV5 Source Data [file 44319_2024_197_MOESM16_ESM.zip › Figure EV5/EV5D-G/EV5E/IRE1 inhibitors-MyHC staining images/B-I09 Representative image.tif]

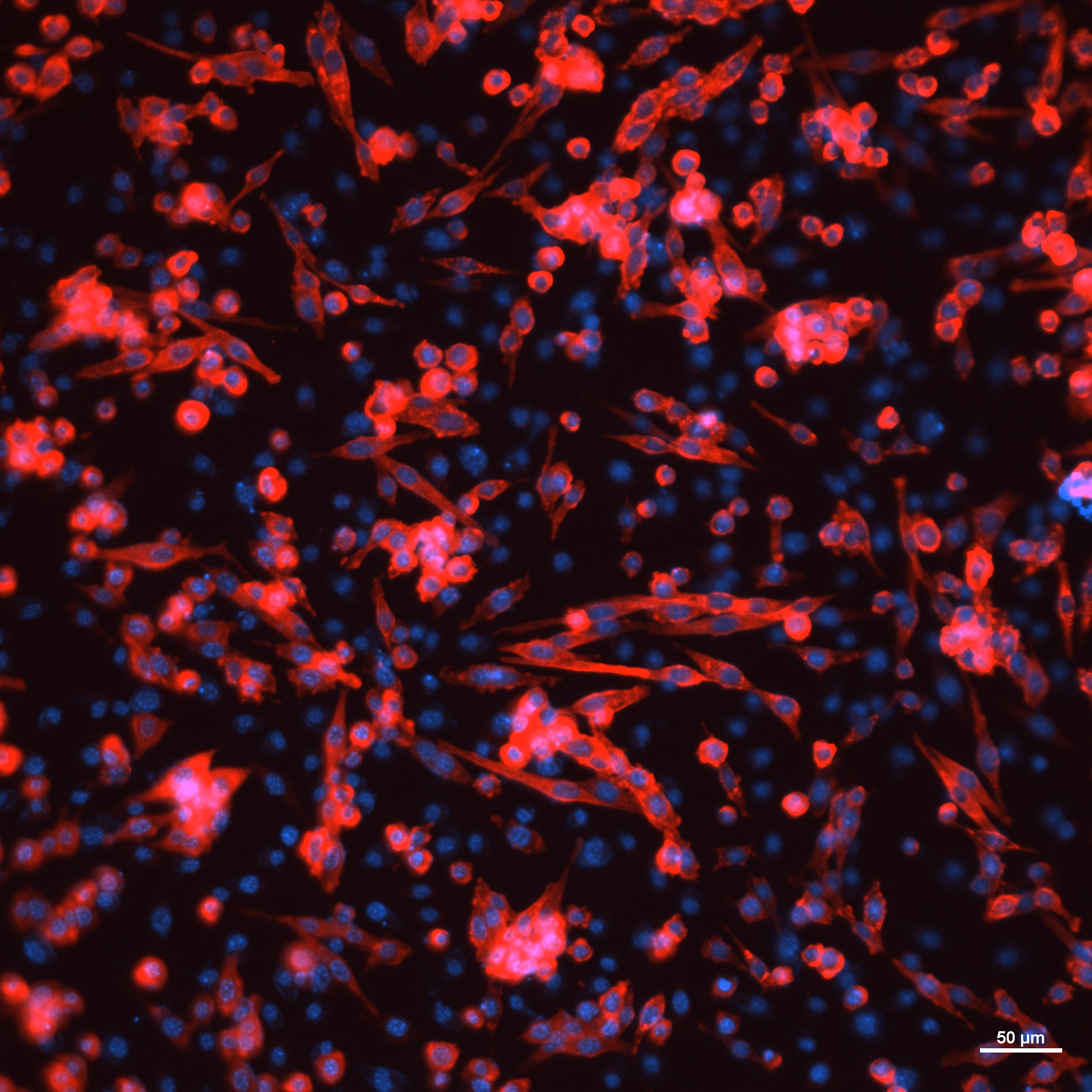

Supplement: Supplementary file 16 — Figure EV5 Source Data [file 44319_2024_197_MOESM16_ESM.zip › Figure EV5/EV5D-G/EV5E/IRE1 inhibitors-MyHC staining images/B-I09-2.tif]

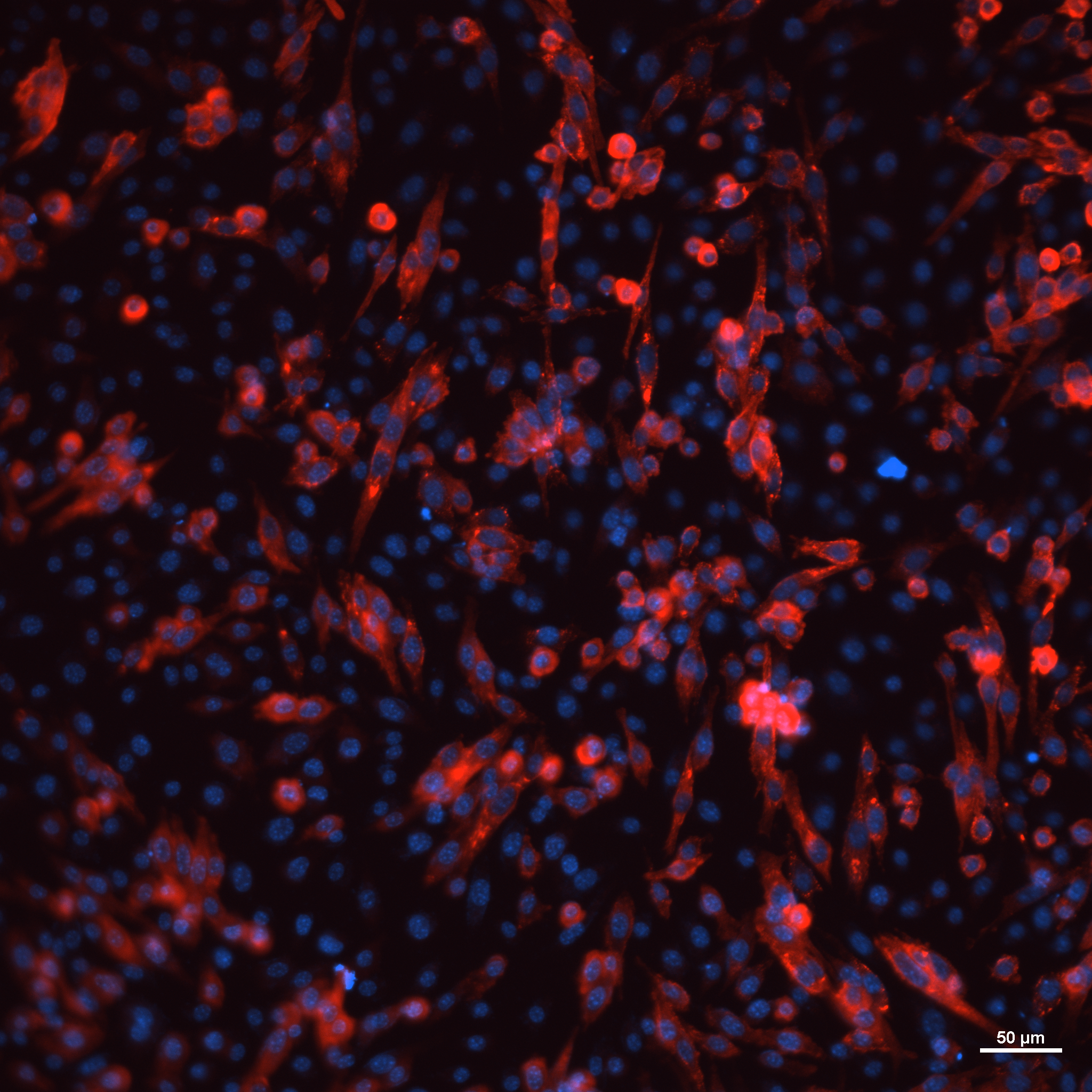

Supplement: Supplementary file 16 — Figure EV5 Source Data [file 44319_2024_197_MOESM16_ESM.zip › Figure EV5/EV5D-G/EV5E/IRE1 inhibitors-MyHC staining images/B-I09-3.tif]

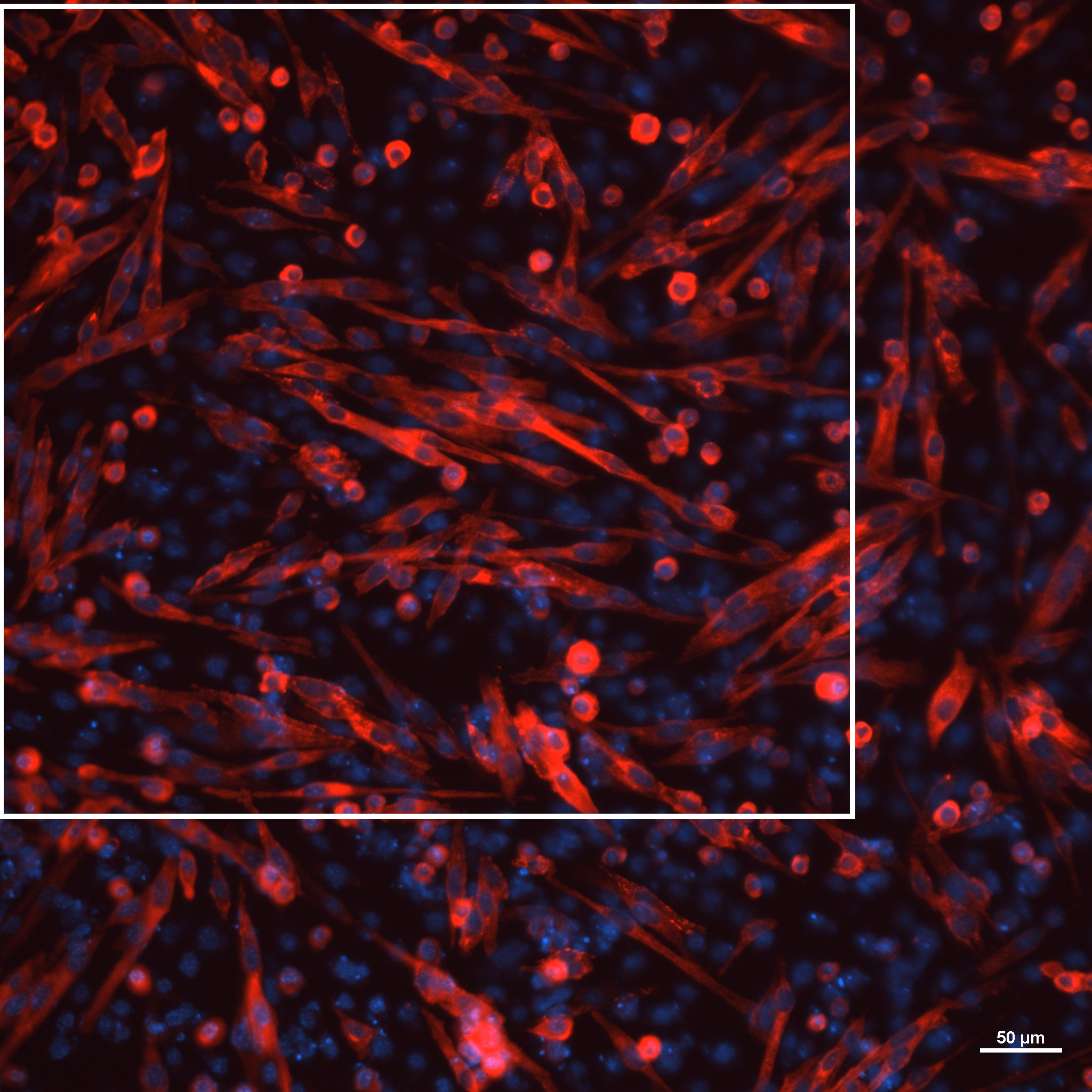

Supplement: Supplementary file 16 — Figure EV5 Source Data [file 44319_2024_197_MOESM16_ESM.zip › Figure EV5/EV5D-G/EV5E/IRE1 inhibitors-MyHC staining images/Control Representative image with box.tiff]

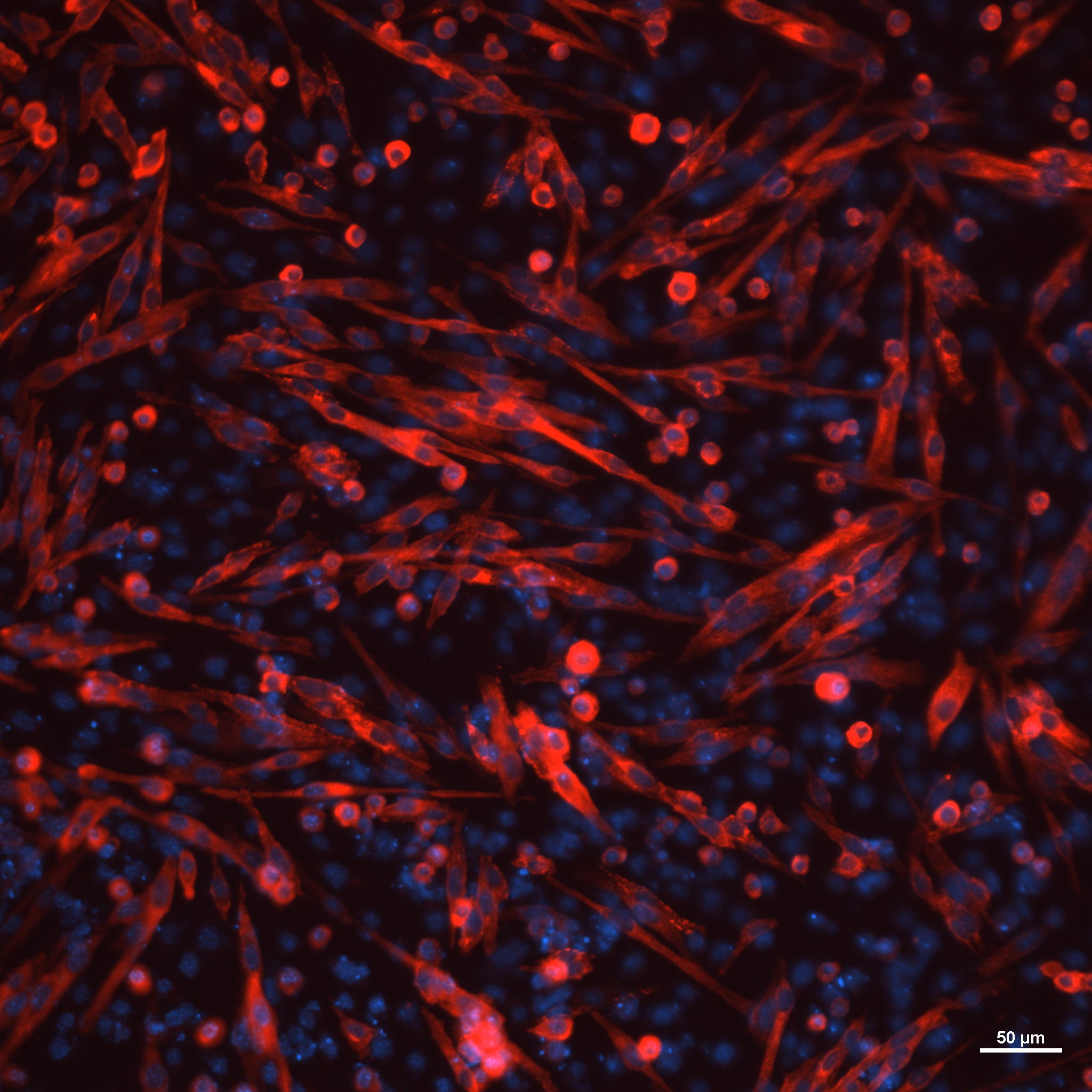

Supplement: Supplementary file 16 — Figure EV5 Source Data [file 44319_2024_197_MOESM16_ESM.zip › Figure EV5/EV5D-G/EV5E/IRE1 inhibitors-MyHC staining images/Control Representative image.tif]

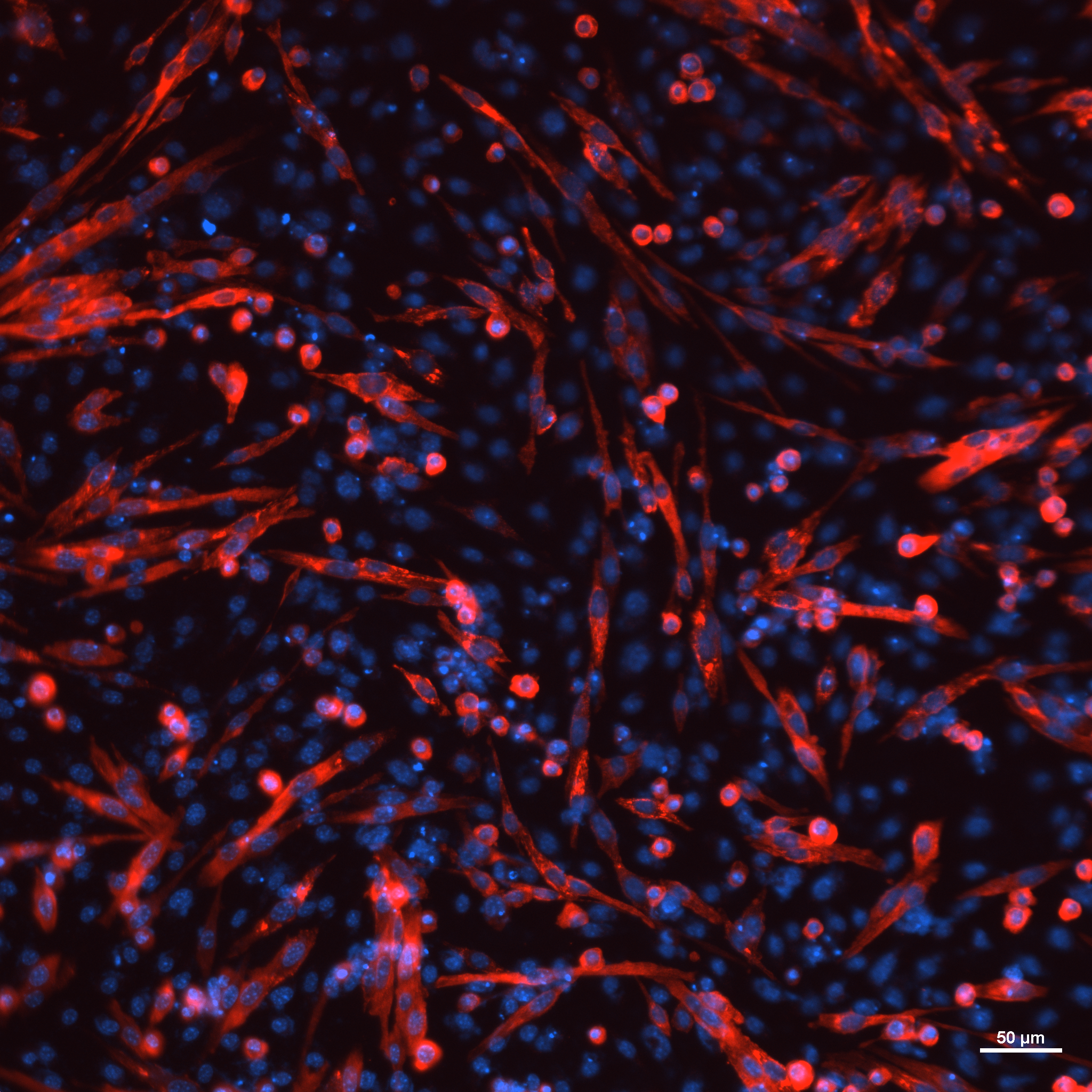

Supplement: Supplementary file 16 — Figure EV5 Source Data [file 44319_2024_197_MOESM16_ESM.zip › Figure EV5/EV5D-G/EV5E/IRE1 inhibitors-MyHC staining images/Control-2.tif]

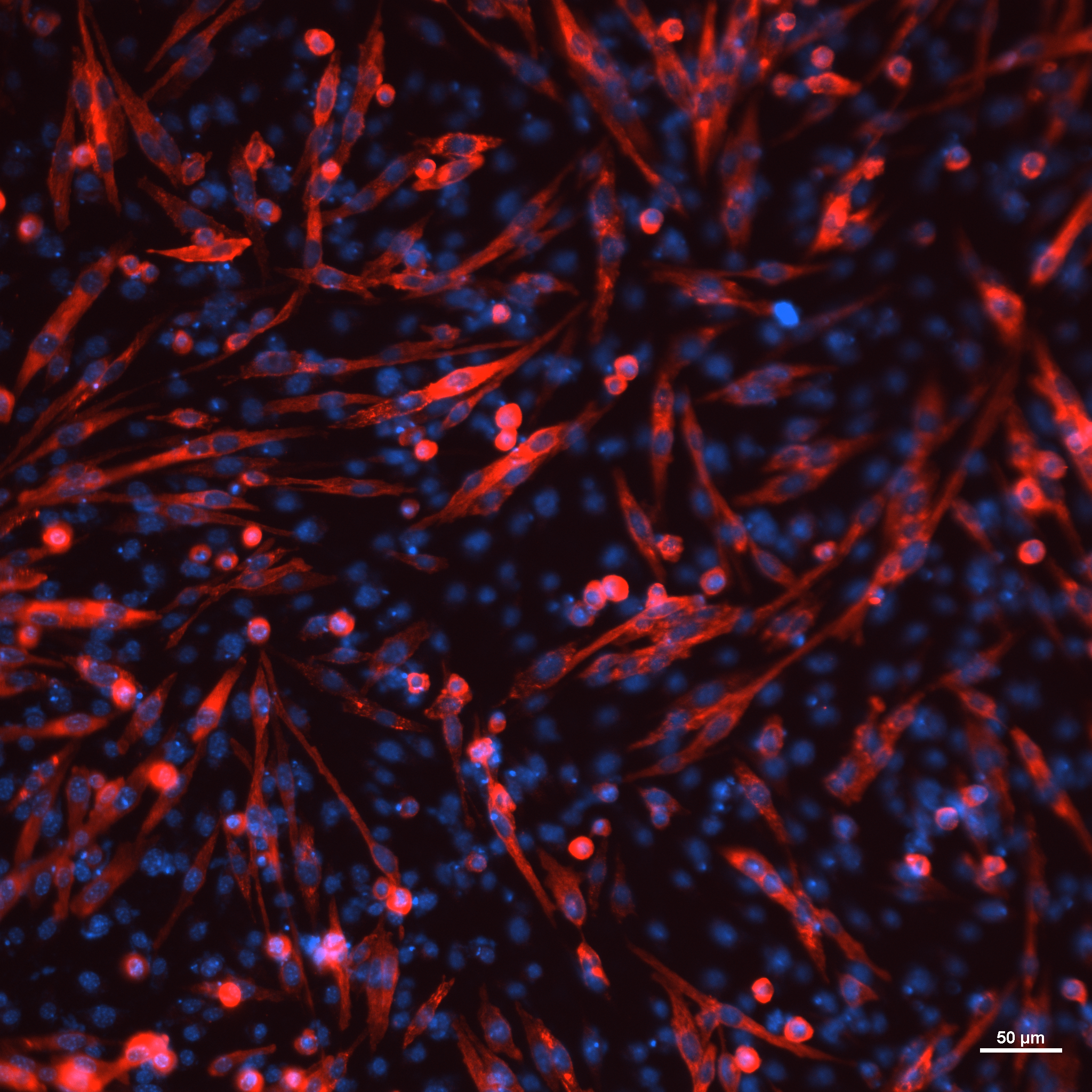

Supplement: Supplementary file 16 — Figure EV5 Source Data [file 44319_2024_197_MOESM16_ESM.zip › Figure EV5/EV5D-G/EV5E/IRE1 inhibitors-MyHC staining images/Control-3.tif]

## Slide 1
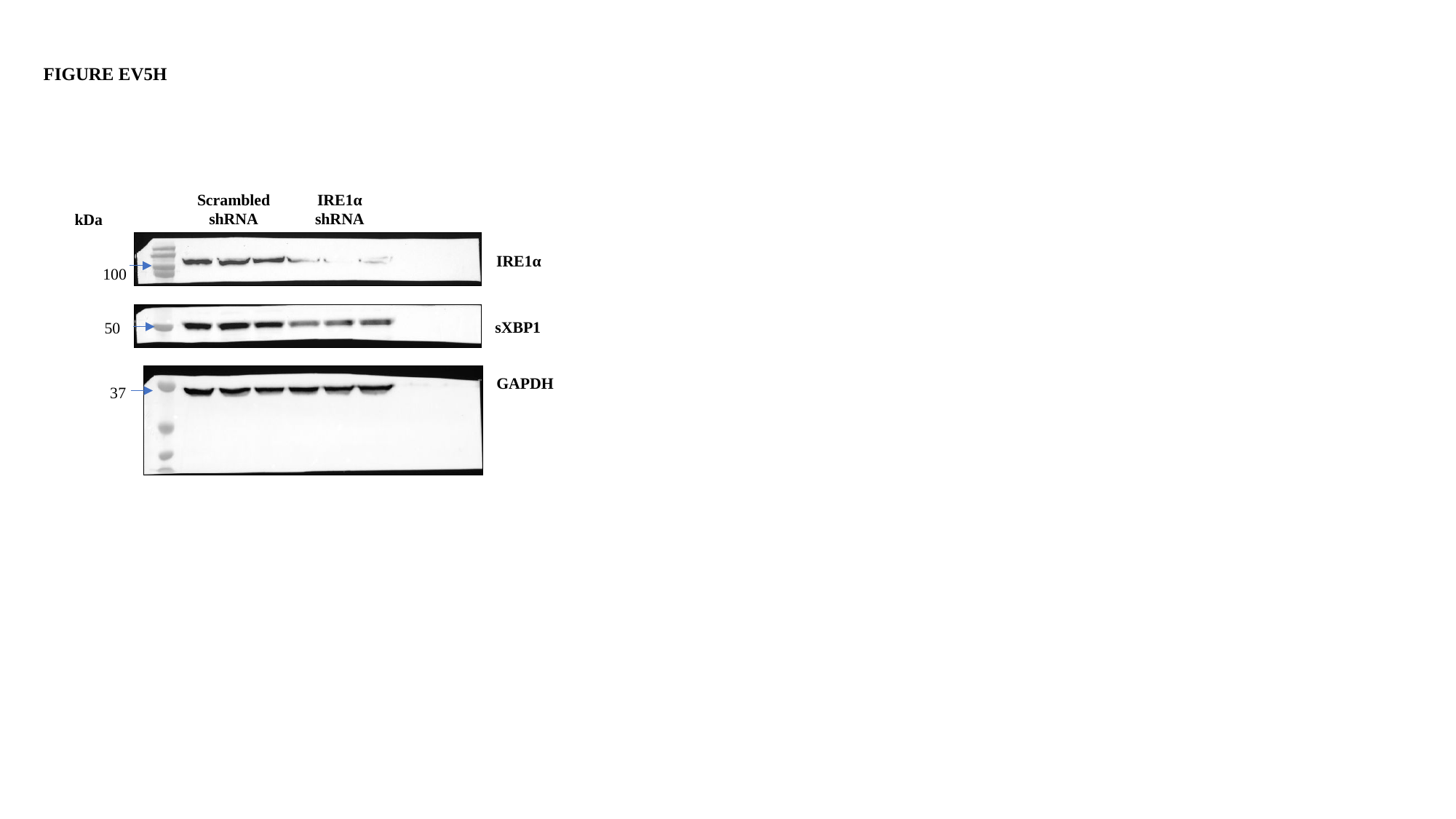

FIGURE EV5H
Scrambled
shRNA
IRE1α
shRNA
kDa
IRE1α
100
sXBP1
50
GAPDH
37

Supplement: Supplementary file 16 — Figure EV5 Source Data [file 44319_2024_197_MOESM16_ESM.zip › Figure EV5/EV5H/Western blot with annotation.pptx]

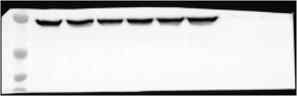

Supplement: Supplementary file 16 — Figure EV5 Source Data [file 44319_2024_197_MOESM16_ESM.zip › Figure EV5/EV5H/Western-GAPDH.png]

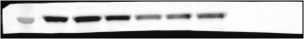

Supplement: Supplementary file 16 — Figure EV5 Source Data [file 44319_2024_197_MOESM16_ESM.zip › Figure EV5/EV5H/Western-sXBP1.png]

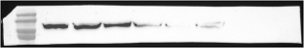

Supplement: Supplementary file 16 — Figure EV5 Source Data [file 44319_2024_197_MOESM16_ESM.zip › Figure EV5/EV5H/Western-Total IRE1a.png]

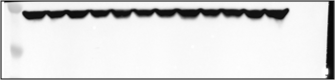

Supplement: Supplementary file 16 — Figure EV5 Source Data [file 44319_2024_197_MOESM16_ESM.zip › Figure EV5/EV5I/Western-GAPDH.tif]

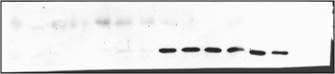

Supplement: Supplementary file 16 — Figure EV5 Source Data [file 44319_2024_197_MOESM16_ESM.zip › Figure EV5/EV5I/Western-Myomaker.tif]
