# Supplementary material for: The IRE1α/XBP1 signaling axis drives myoblast fusion in adult skeletal muscle
Source: EMBO Rep. 2024 Jul 9;25(8):3627–50. doi: 10.1038/s44319-024-00197-4 (PMC11316051; doi:10.1038/s44319-024-00197-4)
Supplement: Supplementary file 17 — Expanded View Figures [file 44319_2024_197_MOESM17_ESM.pdf]

Expanded View Figures

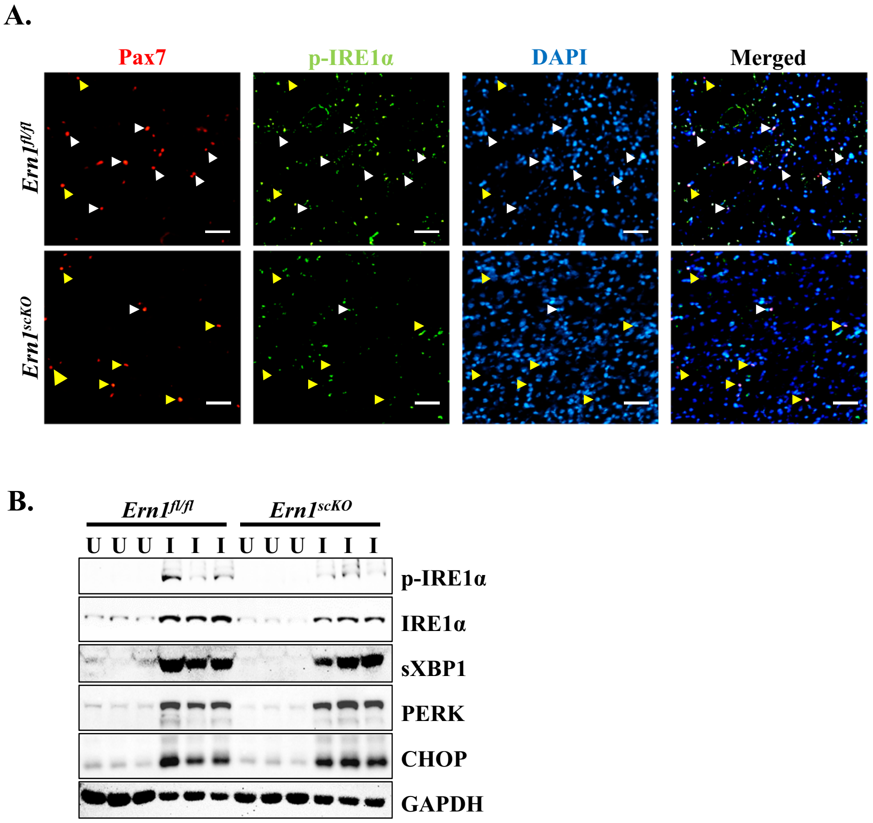

Figure EV1. Deletion of IRE1α in satellite cells of Ern1^{scKO} mice.

(**A**) Representative photomicrographs of 5d-injured TA muscle of *Ern1*$^{fl/fl}$ and *Ern1*^{scKO} mice after immunostaining for Pax7 and p-IRE1α protein and DAPI staining. White arrowheads point to Pax7 and p-IRE1α double-positive cells whereas yellow arrows point to Pax7 positive cells. Scale bar: 50 µm. (**B**) Immunoblots presented here demonstrate the levels of p-IRE1α, total IRE1α, sXBP1, PERK and CHOP protein in uninjured and 5d-injured TA muscle of *Ern1*$^{fl/fl}$ and *Ern1*^{scKO} mice. U uninjured, I injured. Source data are available online for this figure.

 © The Author(s)

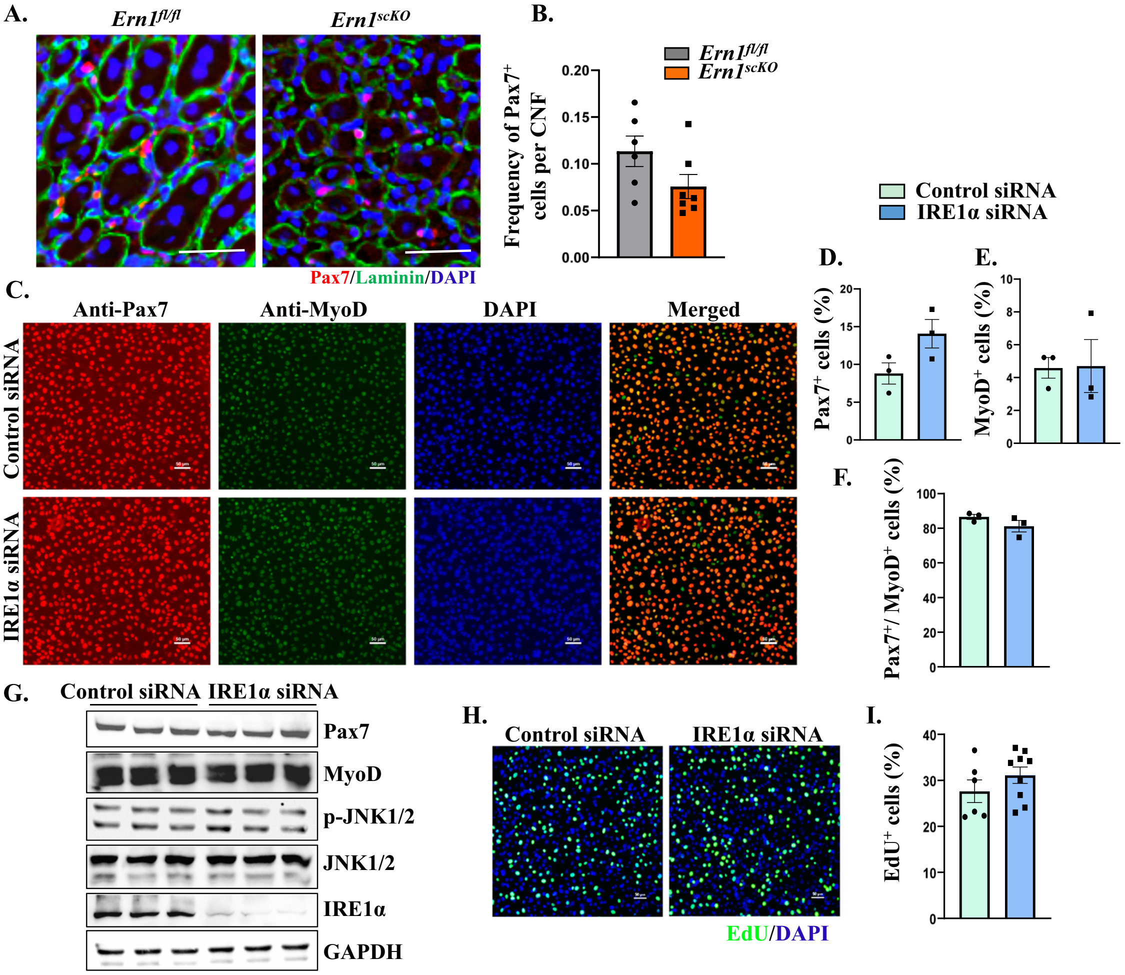

Figure EV2. Targeted ablation of IRE1α does not affect the abundance and proliferation of satellite cells.

(**A**) Representative images of 5d-injured TA muscle of *Ern1*$^{fl/fl}$ and *Ern1*^{scKO} mice after immunostaining for Pax7 and Laminin protein and DAPI staining. Scale bar: 50 µm. (**B**) Quantification of the frequency of Pax7^{+} cells per centrally nucleated myofiber (CNF) in 5d-injured TA muscle of *Ern1*$^{fl/fl}$ and *Ern1*^{scKO} mice. $n = 6$–7 mice per group. Data information: Data were presented as mean ± SEM. No significant difference was observed by unpaired Student *t*-test. (**C**) Representative photomicrographs of primary myoblast cultures transfected with control or IRE1α siRNA for 24 h followed by immunostaining for Pax7 and MyoD protein and DAPI staining. Scale bar: 50 µm. (**D–F**) Quantitative analysis of the proportion of (**D**) Pax7^{+}, (**E**) MyoD^{+}, and (**F**) Pax7^{+}/MyoD^{+} cells in cultures transfected with control or IRE1α siRNA. $n = 3$ (biological replicates) per group. Data information: Data were presented as mean ± SEM. No significant difference was observed by unpaired Student *t*-test. (**G**) Immunoblots presented here show protein levels of Pax7, MyoD, p-JNK1/2, total JNK1/2, IRE1α, and an unrelated protein GAPDH in control and IRE1α siRNA transfected myoblast cultures. (**H**) Representative photomicrographs of EdU^{+}/DAPI^{+} control and IRE1α knockdown cultures. Scale bar: 50 µm. (**I**) Quantification of the proportion of EdU^{+} cells in control and IRE1α knockdown myoblast cultures. $n = 6$–9 (biological replicates) per group. Data information: Data were presented as mean ± SEM. No significant difference was observed by unpaired Student *t*-test. Source data are available online for this figure.

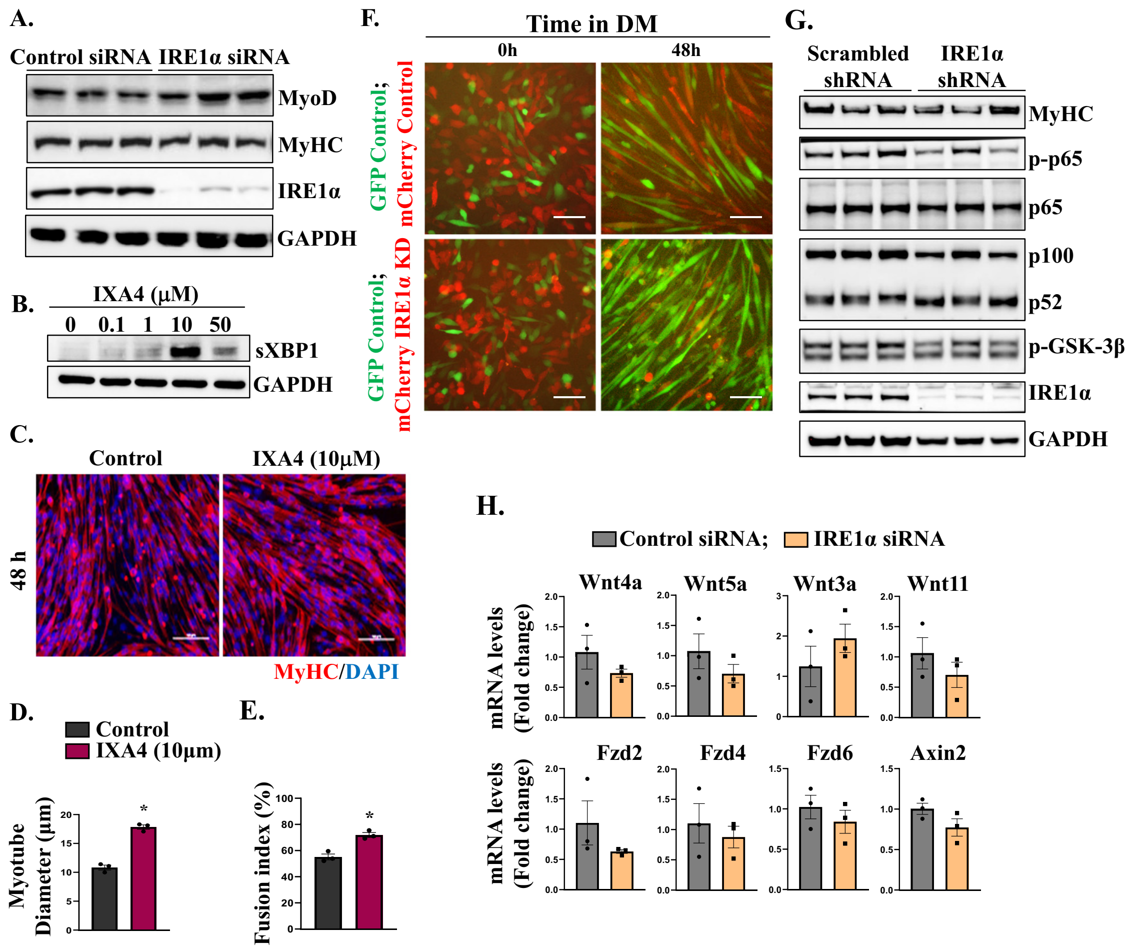

Figure EV3. Knockdown of IRE1α inhibits myoblast fusion without affecting NF-κB and Wnt pathways.

(**A**) Immunoblots presented here show levels of MyoD, MyHC, IRE1α, and GAPDH protein in myoblast cultures after 24 h of transfection with control siRNA or IRE1α siRNA. (**B**) The immunoblot presented here shows the levels of sXBP1 and GAPDH protein in primary myoblasts after 24 h of treatment with indicated concentrations (0, 0.1, 1, 10, 50 μM) of IXA4 compound. (**C**) Representative photomicrographs of cultures treated with vehicle alone (Control) or 10 μM IXA4 for 24 h followed by incubation in DM for 48 h and staining for MyHC. Scale bar: 50 μm. (**D**, **E**) Quantification of (**D**) average myotube diameter and (**E**) fusion index in control and IXA4-treated cultures at 48 h of incubation with DM. $n = 3$ (biological replicates) per group. Data information: Data were presented as mean ± SEM. *$p \leq 0.05$, values significantly different from control cultures analyzed by unpaired Student *t*-test. (**F**) Representative photomicrographs of co-cultured myogenic cells transduced with lentiviral particles expressing GFP protein (GFP Control), scrambled shRNA (Control), or IRE1α shRNA (IRE1 KD) along with mCherry protein at 0 and 48 h of addition of DM. Scale bar: 50 μm. (**G**) Immunoblots showing protein levels of MyHC, p-p65, p65, p100/p52, p-GSK-3β, and IRE1α in scrambled or IRE1 shRNA-expressing cultures at 24 h of differentiation. (**H**) Relative mRNA levels of *Wnt4a*, *Wnt5a*, *Wnt3a*, *Wnt11*, *Fzd2*, *Fzd4*, *Fzd6*, and *Axin2* in primary myoblasts transfected with control or IRE1α siRNA for 24 h. $n = 3$ (biological replicates) per group. Data information: Data were presented as mean ± SEM. No significance was observed by unpaired Student *t*-test. Source data are available online for this figure.

© The Author(s)

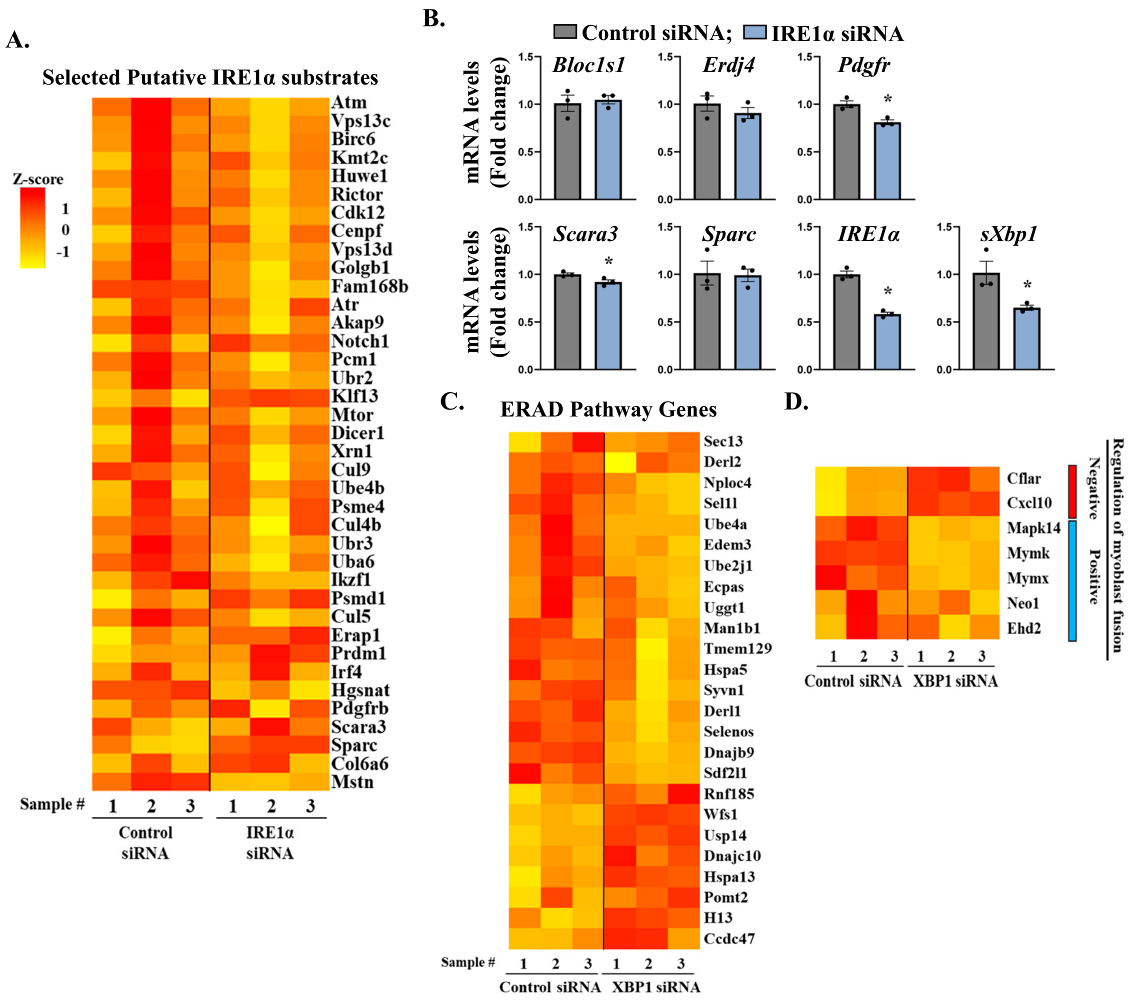

Figure EV4. Gene expression analysis of IRE1α or XBP1 knockdown cultures.

(**A**) Heatmap representing relative mRNA levels of selected putative RIDD substrates in IRE1α knockdown myoblast cultures at 24 h of addition of DM. (**B**) Relative mRNA levels of Bloc1s1, Erdj4, Pdgfr, Scara3, Sparc, IRE1α, and sXBP1 in control and IRE1α siRNA transfected myoblast cultures at 48 h of addition of DM. $n = 3$ (biological replicates) per group. Data information: Data were presented as mean ± SEM. $*p \leq 0.05$, values significantly different from cultures transfected with control siRNA analyzed by unpaired Student *t*-test. (**C**) Heatmap showing mRNA levels of molecules associated with ERAD pathway in XBP1 knockdown cultures compared to control cultures at 24 h of incubation in DM. (**D**) Heatmap showing mRNA levels of regulators of myoblast fusion in XBP1 knockdown cultures compared to control cultures at 24 h of incubation in DM. Source data are available online for this figure.

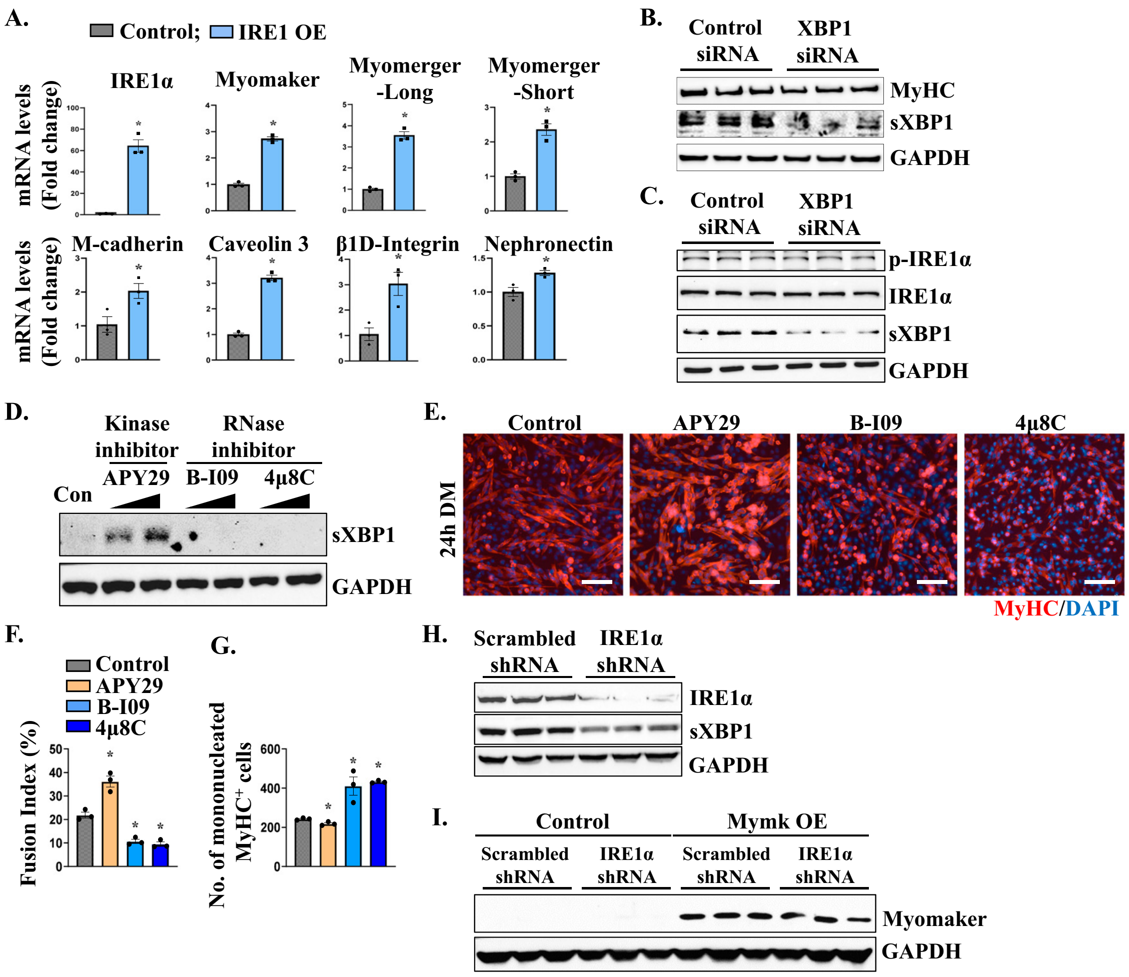

Figure EV5. Effect of IRE1α overexpression or its pharmacological inhibition on myoblast fusion.

(**A**) Relative mRNA levels of IRE1α, myomaker, myomerger-Long, myomerger-Short, M-cadherin, Caveolin-3, β1D-Integrin, and nephronectin in control and IRE1α overexpressing myoblast cultures. n = 3 (biological replicates) per group. Data information: Data are presented as mean ± SEM. *$p \leq 0.05$, values significantly different from control cultures analyzed by unpaired Student *t*-test. (**B**) Immunoblots presented here show the levels of MyHC, sXBP1, and GAPDH protein in control and XBP1 siRNA transfected cultures incubated in DM for 24 h. (**C**) Levels of p-IRE1α, IRE1α, sXBP1, and GAPDH protein in control and XBP1 knockdown cultures. (**D**) Levels of sXBP1 protein in control and APY29 (280 or 560 nM), B-I09 (1.23 or 2.46 μM), or 4 μ8C (4 or 8 μM) treated primary myoblast cultures. (**E**) Representative photomicrographs of myoblast cultures treated with vehicle alone or with APY29 (280 nM), B-I09 (1.23 μM), or 4 μ8C (4 μM) and incubated in DM for 24 h followed by immunostaining for MyHC protein and DAPI staining. Scale bar: 100 μm. (**F, G**) Quantification of (**F**) fusion index and (**G**) number of mononucleated MyHC$^+$ cells in cultures. *n* = 3 (biological replicates) per group. Data information: Data were presented as mean ± SEM. *$p \leq 0.05$, values significantly different from control cultures analyzed by unpaired Student *t*-test. (**H**) Immunoblots showing knockdown of IRE1α and levels of sXBP1 and GAPDH in cultures expressing IRE1α shRNA. (**I**) The immunoblot presented here shows levels of myomaker and GAPDH protein in cultures transfected with *Mymk* cDNA. Source data are available online for this figure.

 © The Author(s)
